# Comparative analysis of core genome MLST and SNP typing within a European *Salmonella* serovar Enteritidis outbreak

**DOI:** 10.1016/j.ijfoodmicro.2018.02.023

**Published:** 2018-06-02

**Authors:** Madison E. Pearce, Nabil-Fareed Alikhan, Timothy J. Dallman, Zhemin Zhou, Kathie Grant, Martin C.J. Maiden

**Affiliations:** a Department of Zoology, University of Oxford, Peter Medawar Building for Pathogen Research, South Parks Road, Oxford OX1 3SY, United Kingdom; b National Institute for Health Research, Health Protection Research Unit, Gastrointestinal Infections, University of Oxford, United Kingdom; c Warwick Medical School, University of Warwick, Coventry CV4 7AL, United Kingdom; d Public Health England, Gastrointestinal Bacteria Reference Unit, 61 Colindale Avenue, London NW9 5EQ, United Kingdom

**Keywords:** Core genome multilocus sequence typing (cgMLST), Single nucleotide polymorphisms (SNPs), Whole-genome sequencing (wgs), *Salmonella*, Outbreak, Phylogeny

## Abstract

Multi-country outbreaks of foodborne bacterial disease present challenges in their detection, tracking, and notification. As food is increasingly distributed across borders, such outbreaks are becoming more common. This increases the need for high-resolution, accessible, and replicable isolate typing schemes. Here we evaluate a core genome multilocus typing (cgMLST) scheme for the high-resolution reproducible typing of *Salmonella enterica* (*S. enterica*) isolates, by its application to a large European outbreak of *S. enterica* serovar Enteritidis. This outbreak had been extensively characterised using single nucleotide polymorphism (SNP)-based approaches. The cgMLST analysis was congruent with the original SNP-based analysis, the epidemiological data, and whole genome MLST (wgMLST) analysis. Combination of the cgMLST and epidemiological data confirmed that the genetic diversity among the isolates predated the outbreak, and was likely present at the infection source. There was consequently no link between country of isolation and genetic diversity, but the cgMLST clusters were congruent with date of isolation. Furthermore, comparison with publicly available Enteritidis isolate data demonstrated that the cgMLST scheme presented is highly scalable, enabling outbreaks to be contextualised within the *Salmonella* genus. The cgMLST scheme is therefore shown to be a standardised and scalable typing method, which allows *Salmonella* outbreaks to be analysed and compared across laboratories and jurisdictions.

## Highlights

• cgMLST is proposed as a universal typing scheme for *Salmonella*.
• cgMLST is congruent with SNP analyses and easier to implement across laboratories.
• Genomic data are consistent with the epidemiology of the outbreak.

## 1 Introduction

Members of the bacterial genus *Salmonella* are a major threat to human health, causing an estimated 80.3 million cases of foodborne gastroenteritis annually (Majowicz et al., 2010). Diarrheal and invasive non-typhoidal *Salmonella* (NTS) infections are responsible for the highest burden of all foodborne infections, causing an estimated loss of 4.07 million disability adjusted life years (DALYs) per year (Kirk et al., 2015). *Salmonella enterica* (*S. enterica*) serovar Enteritidis is the largest single cause of *Salmonella* infection globally, accounting for between 40% and 60% of human cases (Galanis et al., 2006; Hendriksen et al., 2011) and many foodborne disease outbreaks. This serovar is particularly prevalent in Africa, Europe, North America, and parts of Asia (Centers for Disease Control and Prevention (CDC), 2016; Galanis et al., 2006) and poultry and poultry products, including eggs, are the principal human infection source (Velge et al., 2005). Eggs contaminated by serovar Enteritidis phage type 14b caused a large outbreak of disease, comprising over 350 cases, from May to September 2014 in multiple countries including United Kingdom, Germany, France, Austria and Luxembourg (Dallman et al., 2016; Inns et al., 2015).

The 2014 outbreak exemplifies the public health challenges posed by multi-national disease outbreaks (Chatt et al., 2017; Deng et al., 2013; Kinross et al., 2014) that are a consequence of the widespread trade of food and other goods across borders (van Belkum et al., 2007). Such outbreaks present difficulties in their detection, description, and resolution, as most disease surveillance occurs at the national level (van Belkum et al., 2007), which makes the uniform characterisation of disease-associated isolates in different jurisdictions essential. The first broadly accepted *Salmonella* typing scheme, the Kauffmann-White scheme, was based on the serological detection of somatic and flagella antigens (Grimont and Weill, 2007). This scheme facilitated international communication and comparison due to annual updates and maintenance by the World Health Organization (WHO) coordinated reference laboratory, located at the Insitut Pasteur (Paris, France); however, serotyping does not always reflect the genetic relatedness of *S. enterica* lineages (Harbottle et al., 2006; Sangal et al., 2010). For example, the serovar Newport, is associated with several genetically distinct lineages, at least two of which have distinct antimicrobial resistance profiles and different host specificities (Harbottle et al., 2006; Sangal et al., 2010; Sukhnanand et al., 2005). In addition, although there are over 2500 serovars (Grimont and Weill, 2007), the scheme lacks the resolution necessary for outbreak detection and characterisation. Pulse-field gel electrophoresis (PFGE) fingerprinting provided increased resolution and achieved success with the PulseNet initiative (Gerner-Smidt et al., 2006), but these data were difficult to compare among laboratories (Harbottle et al., 2006) as they required highly standardised approaches (Swaminathan et al., 2001) and a global PFGE database proved too complex and costly to implement (Nadon et al., 2017).

Typing methods based on nucleotide sequence, such as multilocus sequence typing (MLST), have provided alternative approaches, which are scalable in resolution and the number of isolates to which they can be applied (Achtman et al., 2012; Maiden et al., 2013). Seven locus MLST sequence types (STs) can be generated with easily replicated protocols (Maiden et al., 1998) and correlate well with the majority of lineages and serovars by means of eBurst groups (eBGs) (Achtman et al., 2012). On the occasions that MLST is incapable of resolving genetically distinct lineages, this is a reflection of the genealogy of the species, as these lineages are often closely related or recombinant (Achtman et al., 2012). Furthermore, the ST and allele nomenclatures are internationally available and readily standardised using schemes hosted on web-based servers (Maiden et al., 1998). While seven locus MLST lacks the necessary resolution for the identification of distinct outbreaks caused by closely related bacterial variants, the arrival of high throughput sequencing technologies led to increasingly affordable and practical whole genome sequence (WGS) analyses, which allowed for high-resolution isolate characterisation (Bakker et al., 2011; Dallman et al., 2016; Inns et al., 2015; Quick et al., 2015). In turn this is facilitating the adoption of WGS analyses for routine surveillance (Ashton et al., 2016; Nadon et al., 2017).

One means of exploiting WGS data is the identification of single nucleotide polymorphisms (SNPs) that vary among isolates. SNPs can be highly informative markers, which are capable of revealing evolutionary histories of homogenous groups (Octavia and Lan, 2010) and detecting and tracing outbreaks (Bakker et al., 2011; Taylor et al., 2015). These are detected by comparing sequence data from isolates of interest with a reference genome and nucleotides that vary within the dataset are then recorded (Bakker et al., 2011). Due to inherent inaccuracies in single reads of high throughput sequencing technologies (Bakker et al., 2011; Sahl et al., 2015), quality assurance criteria, such as minimum coverage and distances allowed between SNPs, must be applied to ensure accuracy and consistency (Bakker et al., 2011; Sahl et al., 2015). Differences in these criteria, assembly pipelines and references used, can present difficulties in standardisation within and, particularly, among laboratories (Kaas et al., 2014; Pightling et al., 2014), complicating the establishment of a consistent nomenclature. Despite these difficulties, SNP-based analyses have been successful in resolving outbreaks, including the multi-country phage type 14b outbreak (Dallman et al., 2016; Inns et al., 2015) discussed here.

An alternative approach is to upscale the MLST concept (Maiden et al., 1998) to include many more loci (Maiden et al., 2013). An advantage of this approach is that, as with MLST, loci used in the schemes are readily maintained and shared among laboratories using online databases such as EnteroBase (http://EnteroBase.warwick.ac.uk) or PubMLST (https://pubmlst.org/) (Jolley and Maiden, 2010). It is possible to assemble schemes ranging from a small number of loci, such as conventional MLST (Maiden et al., 1998) and ribosomal MLST (rMLST) (Jolley et al., 2012a; Maiden et al., 2013), which uses the 53 ribosomal genes, up to the whole genome level: whole genome MLST (wgMLST) (Jolley et al., 2012b; Moura et al., 2016). In order to distinguish between the closely related isolates found within an outbreak (Hoffmann et al., 2014; Jackson et al., 2016), a large number of genes need to be included within a given scheme. Core genome MLST (cgMLST) schemes (Maiden et al., 2013) balance the number of loci used in a scheme with the maximum possible resolution, by including those loci present in the majority of isolates (ranging from 95% to 99% (Bratcher et al., 2014; Moura et al., 2016)) in a given grouping of bacteria (Bratcher et al., 2014; Moura et al., 2016; van Tonder et al., 2014). Ideally these genes reflect the true genealogy within the species and do not change presence over time (Moura et al., 2016), which makes them forward and backward compatible (Moran-Gilad et al., 2015). To encourage this, elements not under strict selection pressures, such as repetitive genes and pseudogenes should be excluded during development (Moura et al., 2016). Previous studies within other bacterial species have shown SNPs and cgMLST to be congruent (Hyun et al., 2014; Kohl et al., 2014). As with MLST and rMLST, cgMLST can form the basis of a stable, reference free, internationally curated nomenclature scheme accessed via databases, that permit global epidemiology and other analyses (Bratcher et al., 2014; Moura et al., 2016).

For the purposes of this work, wgMLST is defined as a non-redundant set of genes that are present across a set of genomes representing a species, akin to a pan-genome. Consequently, a wgMLST scheme includes a greater number of genes and may also include highly variable elements such as repetitive genes and pseudogenes, if they are present in any included genome (Moura et al., 2016).

Here we validate the application of cgMLST for the characterisation of international outbreaks of serovar Enteritidis disease by a reanalysis of the European phage type 14b outbreak and comparison of the results previously obtained by SNP analyses. We show that cgMLST is a viable alternative high-resolution analysis approach, which is highly reproducible and scalable. Furthermore, we demonstrate that cgMLST can be readily implemented in laboratories that only have access to web-based bioinformatics analysis tools, which makes it of particular utility in the resolution of multi-country disease outbreaks.

## 2 Materials and methods

### 2.1 Data sources

The outbreak dataset analysed here includes sequenced reads of isolates described previously (Dallman et al., 2016). Sequence data were retrieved from the Sequence Read Archive (SRA) (https://www.ncbi.nlm.nih.gov/sra) and assembled using the SPAdes (Bankevich et al., 2012) based assembly pipeline developed for the EnteroBase (http://EnteroBase.warwick.ac.uk) database (Table S1).

### 2.2 Datasets

All comparative analyses were performed using isolates from the multi-country European outbreak of phage type 14b serovar Enteritidis, described by Inns et al. (2015) and closely related non-outbreak isolates identified previously by Dallman et al. (2016). Several aspects of this outbreak make it particularly useful for comparing analytical approaches: it spanned several countries, occurred over several months, consisted of three distinct serovar Enteritidis clades associated with primary production and there was sub clustering of point source outbreaks. Furthermore, the availability of non-outbreak isolates that are closely related to those within the outbreak, allowed for further analyses to be performed. For all outbreak isolates, the country and year of isolation were known and for most the month of isolation was also available. The outbreak associated isolates were organised into three datasets (A–C, Table S1).

Dataset A: Consisted of the 530 isolates from the Dallman et al. study (Dallman et al. 2016); however, three of the isolates listed within the dataset: 53063, 45270 and 48189 had identical Short Read Archive (SRA) accessions as other isolates (SRR1969068, SRR1957842 and SRR1957842 respectively) and as such could not be identified within the SRA. These were excluded from the analysis, leaving a dataset of 527 isolates. This dataset contained both outbreak isolates and closely related non-outbreak isolates.
Dataset B: Consisted of the 401 isolates previously used to generate the SNP tree analysis in Dallman et al. (2016); however, 15 isolates were removed, as they were either duplicates or not possible to identify within the SRA, leaving 386 isolates within the analysis. This dataset contained only outbreak isolates.
Dataset C: Consisted of the 193 isolates from the UK part of the outbreak which had exposures associated with several restaurants and food vendors (Inns et al., 2015). Of these, eight isolates were mixed samples and a further six failed the quality controls within EnteroBase (http://EnteroBase.warwick.ac.uk), these fourteen isolates were discarded, leaving a dataset of 179 isolates.

### 2.3 Description of cgMLST and wgMLST

Whole genome MLST (wgMLST) and core genome MLST (cgMLST) schemes have been defined in EnteroBase, as standard genotyping methods for *Salmonella*, for improved discrimination of genotype as compared to 7-locus MLST and 53-locus rMLST schemes.

Construction of cgMLST and wgMLST schemes consisted of three stages. Firstly, coding sequences were compiled from 537 *Salmonella* genomes, including: 167 complete genomes in NCBI, 82 NCTC genomes from PacBio sequencing and 288 representatives for one genome per eBURST group (Achtman et al., 2012) (based on rMLST) within EnteroBase. The rMLST eBURST groups were clusters of genetically closely related isolates (Achtman et al., 2012) based on rMLST, a typing approach which uses the 53 ribosomal genes to identify and define species and their groupings (Jolley et al., 2012a; Maiden et al., 2013). The genomes from the eBURST groups encompassed the genomic diversity within the *Salmonella* genus and consisted of a total of 2,406,798 CDS, which were grouped into 75,864 gene clusters using Uclust (Edgar, 2010). In order to identify homologous regions within each genome, the centroid sequences of each clusters were aligned onto all 537 genomes using nucleotide BLAST, where a gene was considered present if a match covered >70% nucleotide identity over 50% of the length of the centroid sequences.

Paralogous genes (or paralogs) are homologous genes where a gene duplication event has occurred, followed by parallel evolution (Fitch, 1970). The sets of homologous regions with potential paralogs were identified if they were duplicated within any single genome. These regions were iteratively sub-clustered based on phylogenetic topology, firstly each set of sets of homologous regions were aligned together. Then the resulting alignment was used to generate a Maximum likelihood tree using FastTree (Price et al., 2010) and the ETE3 (Huerta-Cepas et al., 2016) python package was used to bipartition the tree to maximise the nucleotide diversity (at least 5%) between the subtrees. Each of the resultant subtrees was evaluated iteratively until no two regions came from the same genome in the same subtree, or the maximum inter-subtree diversity was <5%. Then the original set of homologous regions was replaced with all of its sub-trees.

After the division process, all the homologous sets were scored and ranked according to the summarised alignment scores of their homologous regions. Homologous sets were discarded if they had regions that overlapped with the regions within other sets that had greater scores. Finally, a complete set of 28,883 ‘pan genes’ was identified for the 537 genomes. This set was further refined to 21,065 clusters, after similar gene clusters were merged if genes shared over 70% amino acid similarity. From each cluster, a single representative with the greatest alignment score was chosen to create a wgMLST scheme for *Salmonella*. This removed potential non-specific matches to paralogs in the downstream typing procedure. 3258 *Salmonella* genomes, representing all rMLST STs in EnteroBase (up to May 2016), were typed using this novel scheme.

To generate the cgMLST scheme, a subset of wgMLST loci was selected based on three criteria: (1) the loci were present in over 98% (3193) of the genomes, (2) the coding frames for the loci were intact in over 94% (3063) of the genomes and (3) the number of alleles fell within the majority of all loci. This process yielded a total of 3002 loci, which formed the cgMLST scheme for *Salmonella* employed here.

### 2.4 Comparison of SNPs and cgMLST

SNPs and cgMLST were compared using Datasets A and B. SNP data was provided by Dallman et al. (2016) and cgMLST results were derived using the *Salmonella* cgMLST V2 scheme in EnteroBase (http://EnteroBase.warwick.ac.uk). Every new combination of alleles, was assigned a new cgMLST ST, including if a locus was missing in one isolate and present in another. Public Health England (PHE) use SNP addresses (Ashton et al., 2015) as unique identifiers within a given dataset. These are calculated using hierarchical single linkage at decreasing levels of genetic differences (250, 100, 50, 25, 10, 5 and 0 SNPs different) to identify epidemiologically significant clusters (Ashton et al., 2015). For the statistical comparison between cgMLST and SNP addresses the cgMLST STs were filtered for missing data and combined into single linkage clusters with one as a cut-off, which prevented missing loci from contributing to the variation of cgMLST STs. Comparison of cgMLST single linkage clusters and the PHE SNP addresses was carried out on Dataset A using Simpson's diversity index (Carriço et al., 2006) and the adjusted Wallace coefficient (Severiano et al., 2011). The Simpson's diversity index identifies statistically significant differences between counts of unique profiles generated by different typing methodologies. It has a value between 0 and 1, depending on the number of partitions created by a typing method and a significant difference between discriminatory abilities is determined by examining the value's 95% confidence intervals, to ensure there is no overlap. The adjusted Wallace coefficient is a quantitative measure of congruence, which calculates the statistical significance of similarities between partitions generated by different typing methods, while accounting for the possibility of agreement occurring through chance alone. It also has a value between 0 and 1 depending on the ability of a typing method to further subdivide others and accounts for 95% confidence intervals. This is to better ensure that differences in sub-division are not occurring by chance.

Further comparison of cgMLST and SNPs was performed on Dataset B using the tanglegram algorithm (Scornavacca et al., 2011), generated within Dendroscope 3 (Huson and Scornavacca, 2012). The tanglegram algorithm compares two phylogenetic networks by placing rooted trees side by side and drawing a straight line (or connector) between corresponding taxa (identified through identical tip labels). The algorithm minimises the number of crossings between connectors (Scornavacca et al., 2011), therefore if the two trees are identical no connectors will cross. Changes between the internal nodes of the phylogenies of the two trees can cause multiple short-range crosses, all in the same direction, between connectors; however, this demonstrates that clustering at the tips of the phylogeny remained the same. Distance matrices calculated for cgMLST and SNPs, respectively, were used in SplitsTree 4 (Huson and Bryant, 2006) to create the neighbour-joining trees necessary for the tanglegram algorithm. The neighbour-joining trees were then loaded into Dendroscope 3 (Huson and Scornavacca, 2012) and the tanglegram algorithm was applied.

### 2.5 Comparison of cgMLST and wgMLST: food traceback

Dataset C was used to compare cgMLST and wgMLST. Identification of all loci present within each isolate was performed using the wgMLST scheme, which is available within EnteroBase (http://EnteroBase.warwick.ac.uk). As with the comparison between cgMLST and SNPs, the comparison between cgMLST and wgMLST (Ashton et al., 2015) was carried out using Simpson's diversity index (Carriço et al., 2006) and the adjusted Wallace coefficient (Severiano et al., 2011). Minimum spanning trees were then created within EnteroBase using both cgMLST and wgMLST (Ribeiro-Gonçalves et al., 2016) and were edited using the GrapeTree function. These were annotated using the food traceback, which was provided by PHE, and a visual comparison was performed.

### 2.6 Geographic versus temporal distribution of the isolates using cgMLST

This analysis was performed using Dataset A. Minimum spanning trees were created within EnteroBase (http://EnteroBase.warwick.ac.uk) using cgMLST and edited using the GrapeTree function. These were then annotated using the sample date and country data from Dallman et al. (2016). For the temporal analysis, the minimum spanning tree was annotated using only the year of collection for isolates outside of the outbreak period (2012, 2015 and 2016). Isolates within the outbreak period were annotated using the month and year of collection, except in the instances where only the year of collection was available, in these cases only the year was used.

### 2.7 Placing the outbreak within the rest of Enteritidis using cgMLST

A search of EnteroBase was performed, using the built-in *Salmonella* In Silico Typing Resource (SISTR) (Yoshida et al., 2016), for genome records that were identified as serovar Enteritidis. This returned 8365 (Table S2) isolates, which passed the internal EnteroBase assembly checks, and were analysed using cgMLST to generate a minimum spanning tree. The minimum spanning tree was annotated using the seven most common rMLST STs within serovar Enteritidis and the remaining isolates were grouped together under the collective term ‘other’.

## 3 Results

### 3.1 Comparison of SNPs and cgMLST: tanglegram (Scornavacca et al., 2011)

The 527 unique isolates in Dataset A (Table S1) were resolved into 249 different sequence types using the PHE SNP address (Ashton et al., 2015) (Simpson's diversity index = 0.949; 95% CI: 0.937–0.961). In comparison, single linkage cgMLST clusters generated fewer profiles, resolving the dataset into 229 unique sequence types (Simpson's diversity index = 0.901; 95% confidence interval (CI): 0.879–0.923 (Carriço et al., 2006)) (P < 0.001). The SNP addresses therefore provided a greater resolution than cgMLST single linkage clusters (adjusted Wallace coefficient = 0.874; 95% CI: 0.808–0.942). Some cgMLST single linkage clusters, however, did further subdivide SNP addresses, although not as strongly (adjusted Wallace coefficient = 0.430; 95% CI: 0.350–0.507 (Severiano et al., 2011)) (P < 0.001). Unlike a SNP analysis, a cgMLST scheme will not include intergenic regions. Furthermore, only one allelic change will be counted when multiple nucleotide changes within the same gene. On the other hand, short insertions or deletions in the core genes are ignored by many SNP analyses but captured by a cgMLST scheme, because they change the sequences of the genes. The small differences between SNP and cgMLST analyses observed here will be due to a combination of these factors.

**Fig. 1:**
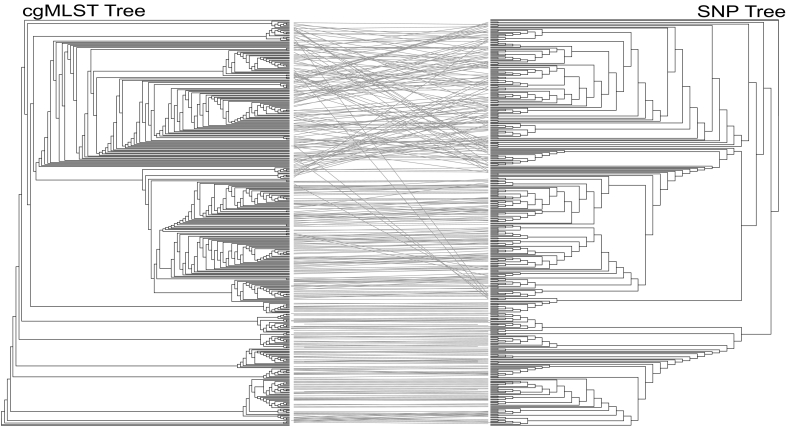
Comparison between whole genome SNP phylogeny and cgMLST in European Outbreak data. Tanglegram (Scornavacca et al., 2011) linking tips with the same label to each other via a straight line, produced within dendroscope 3 (Huson and Scornavacca, 2012) of 386 isolates from the *Salmonella* serovar Enteritidis PT14b outbreak. A SNP based neighbour-joining tree (right) is compared with one generated using cgMLST (left). Clustering within the two trees was mostly congruent, although the top section of the tree showed several inversions. These inversions were caused by differences in clustering at the deeper nodes, which were not as strongly supported. This led to the positioning of some isolates, which were clustered at the edge of one tree, moving to the centre of the other.

Dataset B (Table S1) isolates were used to generate a tanglegram for a visual comparison of cgMLST and SNPs, which showed good congruence between the two methods (Fig. 1). There were some minor differences in the two phylogenies, predominantly caused by inversions of clusters, which was explained by differences between the internal nodes, located deeper within the phylogenies. A visual comparison of the two neighbour-joining trees (Fig. S1) indicated that the majority of isolates were grouped into the same clusters, whether analysed with SNPs or cgMLST.

### 3.2 Geographic versus temporal distribution of the isolates using cgMLST

The majority of the outbreak occurred from May to September 2014, with sporadic cases persisting until December and closely related isolates occurred in 2012, 2015 and 2016. The outbreak was spread across multiple countries including: United Kingdom, Germany, France, Austria and Luxembourg (Dallman et al., 2016; Inns et al., 2015).

**Fig. 2:**
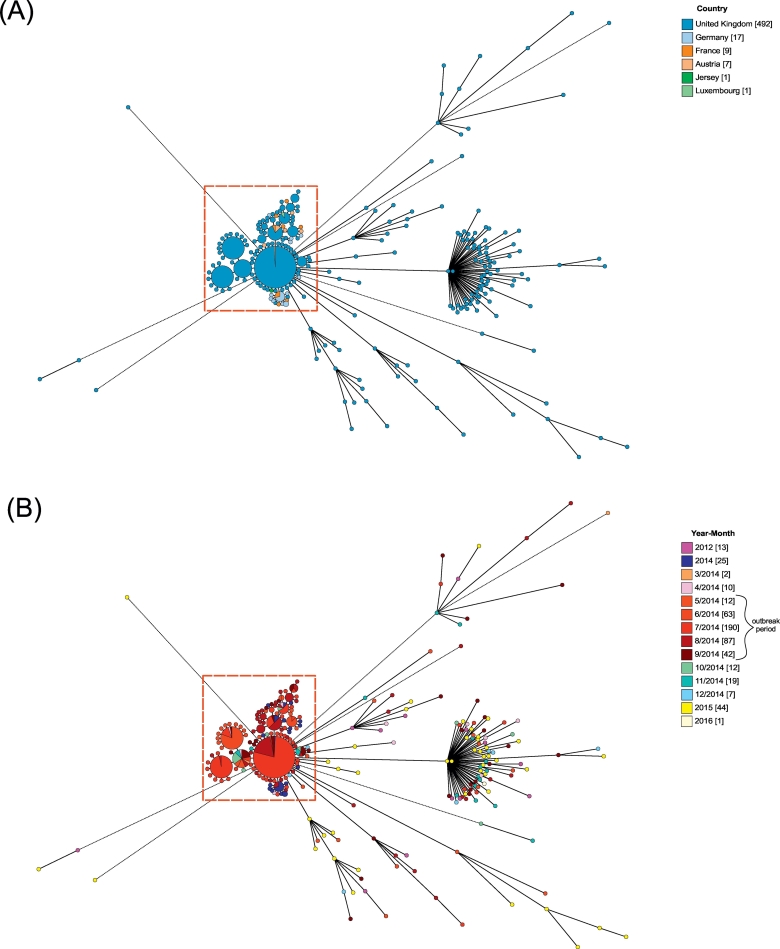
Core genome minimum spanning tree illustrating the spread of outbreak isolates based on country of origin (A) and time of collection (B). Minimum spanning tree for 527 *Salmonella* serovar Enteritidis isolates, from 2012 to 2016 generated from cgMLST data, using the 3002 locus cgMLST scheme, which is available within EnteroBase. Branches longer than 301 differences were proportionally shortened and are represented by dashed lines. The isolates within this analysis were from the Pt14b outbreak or were closely related to isolates from the outbreak. A: Annotated by country: United Kingdom, dark blue; Germany, light blue; France, orange; Austria, light orange; Jersey, dark green; and Luxembourg, green (Dallman et al., 2016). The majority of the outbreak (within the red box) fell within the top left cluster of the tree, while the rest of the tree was dominated by sporadic cases. This tree indicated that there was no relationship between the country of isolation and the cgMLST clusters, indicating that the diversity predated the outbreak. B: Annotated by year of isolation. Most outbreak-associated cases occurred from May to September 2014, with individual cases persisting until December. As the year of most interest the months of 2014 were individually labelled, if the information was available. The outbreak months of May to September were coloured on a gradient and the remaining months of 2014 were coloured independently, while the other isolates were grouped by year: 2012, 2015 and 2016. Most of the outbreak associated isolates were clustered in the top left of the tree (within the red box) with the tree otherwise dominated by sporadic cases. This topology indicated that there was a relationship between year of infection and genetic diversity, as isolates from 2012, 2015 and 2016 predominately occurred outside of the outbreak cluster. Groups of cgMLST types within the outbreak were frequently dominated by isolates from one or two months, suggesting that different outbreak strains were dominant at different times during the outbreak. (For interpretation of the references to colour in this figure legend, the reader is referred to the web version of this article.)

The Minimum spanning trees generated from Dataset A (Table S1) showed that the majority of outbreak isolates formed one large cluster, with the sporadic cases more dispersed through the remainder of the phylogeny (Fig. 2). However, there was no observable relationship between isolate clusters and country of origin (Fig. 2A), suggesting that diversity did not emerge during the outbreak but was present before the start of the outbreak, as was suggested from SNP analyses (Dallman et al., 2016).

When the phylogeny was analysed by the month and year of isolation, isolates from 2012, 2015 and 2016, which were closely related but did not belong to the outbreak, did not fall within the same genetic cluster as the outbreak isolates. These isolates were also diverse, suggesting that they were more genetically distant from the outbreak group and each other (Fig. 2B). Clusters of cgMLST types within the outbreak often contained isolates from only one or two months and when a cluster consisted of multiple months they were generally consecutive. This suggested that various closely related subtypes of serovar Enteritidis replaced each other over the course of the outbreak. This was consistent with the food traceback investigations, which suggested that there were multiple point source outbreaks within the United Kingdom (Dallman et al., 2016).

### 3.3 Comparison of cgMLST and wgMLST: food traceback

Dataset C (Table S1) was used to compare cgMLST and wgMLST. The 177 isolates had 177 unique wgMLST sequence types (Simpson's diversity index = 1.000; 95% CI: 1.000–1.000), compared with 137 cgMLST types (Simpson's diversity index = 0.981; 95% CI: 0.969–0.944 (Carriço et al., 2006)) (P < 0.05). Due to the larger number of distinct profiles obtained through wgMLST, it provided additional resolution compared to cgMLST (adjusted Wallace coefficient = 1.000; 95% CI: 1.000–1.000 (Severiano et al., 2011)). However, there was no statistically significant difference (P = 1.000) in the discriminatory ability of cgMLST and wgMLST.

**Fig. 3:**
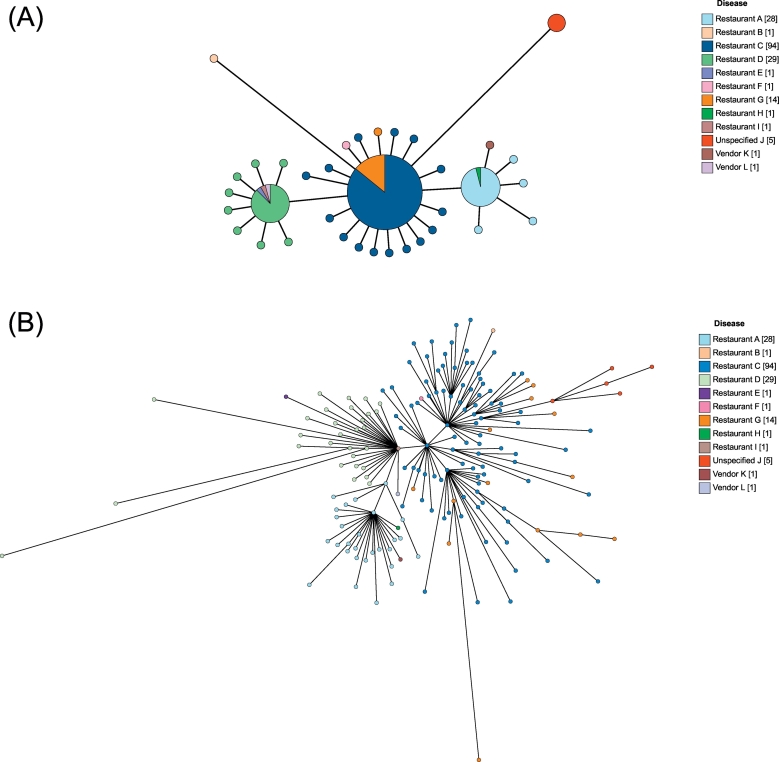
Core genome minimum spanning tree (A) and whole genome minimum spanning tree (B). A: minimum spanning tree of 177 unique isolates calculated using the cgMLST scheme, which consists of 3002 loci and is available in the EnteroBase database. B: minimum spanning tree of the 177 unique isolates calculated using the wgMLST scheme from EnteroBase, which is based on the full genomes of 537 complete or representative genomes (https://bitbucket.org/enterobase/enterobase-web/wiki/Salmonella%20Statistics) and is available in the EnteroBase database. The trees, generated using EnteroBase, were labelled with 12 different sources of infection from the *Salmonella* serovar Enteritidis Pt14b outbreak previously described in Inns et al. (2015) and showed that the isolates form several distinct clusters, predominantly consisting of multiple sources. Both trees showed the same isolates clustering together and were suggestive that cgMLST and wgMLST are congruent. However, as shown by the individual data points wgMLST was more discriminatory than cgMLST. All isolates from the same source clustered together, which suggested a single strain of infection per source.

These results were consistent with the results of mapping the available food traceback isolates onto minimal spanning trees (Fig. 3). Both approaches (Fig. 3A and B) grouped the isolates into the identical clusters of: restaurants A and H and vendor K; restaurants D, E, I and vendor L; restaurants B, C, F and G and unspecified J. Neither approach distinguished isolates on geographical source suggesting that there was no relationship between place of isolation and genetic clustering of the isolates. Furthermore, when compared with the original food traceback (Inns et al., 2015) there was no relationship between clusters and the wholesalers supplying the sources, suggesting that any diversity within the isolates was generated at the source, before the outbreak began.

### 3.4 Placing the outbreak within the rest of Enteritidis using cgMLST

**Fig. 4:**
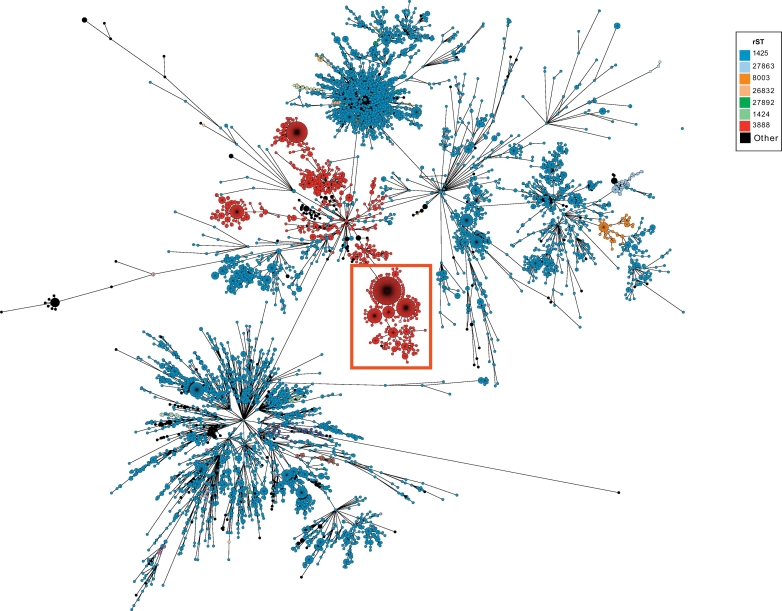
Core genome minimum spanning tree placing the outbreak within the rest of serovar Enteritidis. Minimum spanning tree, generated using EnteroBase, including genome data for all serovar Enteritidis (9380) and PT14b outbreak isolates within the database as of December 2016. Each node, whose size is proportional to the number of isolates, corresponds to a single sequence type profile based on 3002 core genes (cgMLSTv2, as described in EnteroBase). Nodes are colour-coded according to sequence types defined by rMLST. The Pt14b outbreak isolates (boxed in red) from a unique cluster within serovar Enteritidis diversity. All nodes are linked (black lines) sequentially to the most closely related node with the highest number of cgMLST alleles in common. (For interpretation of the references to colour in this figure legend, the reader is referred to the web version of this article.)

Beyond being able to resolve outbreaks, cgMLST schemes are also capable of placing an outbreak into a wider context. As they use a predefined set of loci, it is possible to show the outbreak in the context of other *Salmonella* isolates, for example using all of the serovar Enteritidis isolates available within the EnteroBase database (Fig. 4). Annotation of the isolates using rMLST showed that all outbreak isolates belonged to rMLST 3888. Despite the increased number of Enteritidis isolates, the outbreak formed an identifiable cluster within the minimal spanning tree.

## 4 Discussion

WGS technologies provide powerful data collection and analysis techniques that can be implemented to tackle the challenges presented by the globalisation of food production and distribution. The continual transportation of foods, and therefore pathogens, across boarders requires routine high-resolution surveillance and outbreak detection methods. For the characterisation of bacterial isolates, nucleotide sequence based technologies have several potential advantages, including high throughput, reproducibility and sensitivity (Bakker et al., 2011; Dallman et al., 2016; Quick et al., 2015). For effective implementation within a public health setting, uniform nomenclatures and protocols need to be adopted. Databases such as PubMLST.org (Jolley and Maiden, 2010) and EnteroBase (http://EnteroBase.warwick.ac.uk) can facilitate global communication and collaboration by providing widely accessible nomenclature servers for multiple hierarchical MLST schemes (Maiden et al., 2013), which can be used to characterise isolates, grouping and discriminating between them as required for the epidemiological application (Nadon et al., 2017).

Foodborne disease outbreaks can be detected via a top-down approach, where a common source is determined, such as a restaurant or specific food product and then isolates relating to that source can be analysed. Another approach is bottom-up, where isolates are found to be sufficiently similar that they are deemed likely to be a consequence of infection from a common source, which may be identified by epidemiological investigation. While a top-down approach is easily analysed by many different typing systems, a bottom-up approach can be more difficult, especially when more than one country is involved. An advantage of gene-by-gene approaches such as MLST, rMLST and cgMLST, is that they can be employed to make rapid comparisons of large numbers of isolates (Maiden et al., 2013). Progressively increasing the resolution of these approaches, by increasing the number of loci used, allows for the identification of previously undetected disease clusters. The cgMLST schemes enable high resolution within a species or genus which can also be used as the basis of nomenclature (Maiden et al., 2013; Moura et al., 2016) and the usefulness of such schemes for investigation and description of outbreaks, has previously been demonstrated (Hyun et al., 2014; Kohl et al., 2014).

In cgMLST a set of loci, 3002 for the *Salmonella* scheme described here, is used for gene-by-gene comparisons of assembled bacterial isolates. These schemes summarise the diversity present within the nucleotide sequences of these loci, as each distinct sequence at every locus is identified as a variant. Using alleles as the basis of a comparison, instead of nucleotide sequences, accounts for differences due to single evolutionary events that introduce many polymorphisms, such as horizontal gene transfer (Moura et al., 2016). An advantage of the allelic approach is that loci which differ within a comparison are obvious and the nucleotide sequences can still be accessed when necessary for any further analyses. Within this scheme only coding sequences are included; however, this is not dissimilar to SNP based analyses as polymorphisms within intergenic regions are often excluded, due to low coverage or genomic complexity (Bakker et al., 2011; Sahl et al., 2015). While the exclusion of the accessory genome may lead to a decrease in resolution, it enables direct comparison with all members for which the scheme was established (Pightling et al., 2014).

Isolates from the serovar Enteritidis Pt14b outbreak which clustered together when analysed with cgMLST were highly congruent with clusters obtained by SNP analysis. All clusters contained identical sets of isolates; however, there were some differences between the cgMLST and SNP analyses when more distant relationships among the clusters were examined. The differences between the approaches are likely due to the differences between the data points the two approaches analyse. This demonstrates that cgMLST provides a standardised approach, which is capable of high resolution and reliable isolate characterisation, even though the accessory genome and intergenic regions are not included. The cgMLST approach also does not exclude analysis with SNP-based approaches, if required.

The cgMLST types can be visualised with a variety of tools widely available on the internet, a number of which have been incorporated into the EnteroBase website. As the two principal features of this outbreak were date and location of isolation, these were used for diagram annotation. The cgMLST analyses supported the conclusions established by the SNP analyses that there is no relationship between country of isolation and the genetic spread of the isolates and that the diversity within the outbreak was generated at the source (Dallman et al., 2016). Additionally, data presented by Inns et al. (2015) demonstrated that cases occurred concurrently across the UK over the outbreak period, further supporting this conclusion.

The clustering of isolates by cgMLST type suggested that there was a relationship between the date of isolation and the spread of the cgMLST types. The most prevalent cgMLST types varied over the course of the outbreak, as dominant strains were replaced, approximately at monthly intervals. This observation was further supported by the epidemiological data (Inns et al., 2015), which showed several ‘peaks’ in incidence across the outbreak, at least in part caused by point-source outbreaks (Dallman et al., 2016), and a decreasing ‘tail’ of cases at the end of the outbreak. Throughout the outbreak period, multiple strains were in continual circulation, as several cgMLST types which occurred at the start of the outbreak initially declined in prevalence but later reoccurred.

Restaurants and vendors were found to be contaminated with genotypes that clustered together when analysed with cgMLST; however, there was no known relationship between them, such as the wholesalers who supplied them (Inns et al., 2015). Conversely, one wholesaler supplied to multiple restaurants and vendors but the cgMLST types associated with these businesses did not cluster together in the phylogenetic analyses. Each time a restaurant or vendor was contaminated, it was with the same cgMLST type, suggesting that these locations were only contaminated once and from a single source. These conclusions are consistent with of the epidemiological data (Chatt et al., 2017; Dallman et al., 2016; Inns et al., 2015) and demonstrate that cgMLST can be used to make epidemiologically relevant inferences.

When isolates are closely related, they will also have parts of the accessory genome in common with each other. This can be captured by a wgMLST analysis. As there is a current lack of standardisation (Maiden et al., 2013), this paper uses the scheme provided by EnteroBase, which defines wgMLST based on 537 complete or representative genomes (“Enterobase-web,” 2017). In this wgMLST scheme, all shared genes of the given dataset are compared. Although this analysis is potentially capable of providing more resolution among isolates than cgMLST, by containing more loci, a wgMLST scheme is likely to contain pseudogenes and paralogous genes due to the inclusion of all shared genes within a dataset. The inclusion of pseudogenes and paralogous genes in such comparisons can generate spurious differences potentially leading to the inaccurate clustering of isolates in the analysis. Pseudogenes resemble functional genes but are inactive, whereas duplicated genes are copies of functional genes. As such, they are often subject to different evolutionary constraints and may acquire mutations faster (Kuo and Ochman, 2010; Li et al., 2003), which can lead to the appearance of greater divergence and misleading local relationships (Li et al., 2003). Such differences may explain some of the inconsistencies among the cgMSLT and wgMLST analyses described here, where the isolates from restaurants D, E, I and vendor L were observed to be more closely related to those from restaurants B, C, F and G with cgMLST (Fig. 3A), while the same group of isolates was more closely related to the isolates from restaurants A, H and vendor K by wgMLST (Fig. 3B). The clustering of the isolates does not always relate to important genealogical changes and variations between the two approaches may be caused by stochastic changes, within highly variable regions, which do not relate to disease or fitness (Zhou et al., 2013). For these reasons a scheme that includes every possible gene in a dataset is not useful for surveillance purposes.

The cgMLST scheme was very effective in relating the outbreak isolates to the known diversity of *S. enterica* serovar Enteritidis. Using cgMLST it was possible to place the outbreak within the context of all known Enteritidis genomes that were available within EnteroBase. It is also possible to examine isolates based on other criteria, such as country or year of origin, enabling the investigation of trends and relationships that may otherwise not be immediately apparent and to identify closely related non-outbreak isolates within the database. This enables epidemiological inferences and investigations to be made, such as establishing the length of time for which a given genotype persists. For example, an outbreak of serovar Enteritidis phage type 8, linked to feeder mice was detected by PHE, through routine SNP analysis of whole genome sequences (De Pinna, 2016). Due to the low levels of isolates which were continually being fed into the population, this outbreak would not have been detected without routine data surveillance (De Pinna, 2016). The widespread adoption of the cgMLST approach will enable the identification of such relationships at the global level and over time.

One improvement of the *Salmonella* cgMLST scheme would be the implementation of a formal, standardised clustering system, to enable the sorting of cgMLST profiles into closely related groups, such as clonal complex. This is necessary for enabling the easy communication of an outbreak between laboratories, as cgMLST types are too discriminatory and the current implementation includes missing alleles. One way to do this would be to adopt a similar approach to that used by PHE with SNP typing (Ashton et al., 2015) and create ‘cgMLST addresses’ which are based on single linkage clustering to form a hierarchy of relatedness (Ashton et al., 2015).

## 5 Conclusions

This work demonstrates that the cgMLST scheme presented has sufficient resolution to detect a multi-country disease outbreak caused by very closely related strains of serovar Enteritidis, and to identify sub-structure within the isolates obtained during the outbreak. The cgMLST analyses were congruent with the wgMLST analyses and previously used SNP analyses, but the cgMLST scheme has the advantage of being readily and consistently applied in different laboratories and jurisdictions as it uses a consistent set of conserved loci and allele designations. The analyses can be undertaken with more or fewer isolates making analyses performed with cgMLST both replicable and forwards and backward compatible. As the core genome is common to all members of the species, it is possible to rapidly locate outbreak isolates in the context of the known diversity of serovar Enteritidis. Finally, the availability of web-based analyses platforms enables these high-resolution analyses to be conducted with minimal requirements for locally installed bioinformatics infrastructure.

The following are the supplementary data related to this article.

Supplementary Fig. 1 Core genome neighbour-joining tree (A) and single nucleotide polymorphism neighbour-joining tree (B).
A: neighbour-joining tree of the 386 isolates used to create the Tanglegram (Fig. 1) calculated using the cgMLST scheme which consists of 3002 loci and is available in the EnteroBase database. The tree was drawn using SplitsTree4 (Huson and Bryant, 2006).
B: neighbour-joining tree of the 386 isolates used to create the Tanglegram (Fig. 1) calculated using the PHE SNP pipeline. The tree was drawn using SplitsTree4 (Huson and Bryant, 2006).
The trees, drawn within SplitsTree4 (Huson and Bryant, 2006) were coloured based on closely related groups of isolates. The overall topology of the trees and the majority of the clusters, demonstrated strong congruence between SNP and cgMLST based approaches. Minor differences were observed, such as the purple group was split into four clusters via SNPs and only two via cgMLST, none of which were directly congruent between the two methods. However, all isolates within the purple clusters were more closely related to each other than their neighbours. There were also two isolates, split into the red and purple groups by SNPs, which were very closely related via cgMLST. Despite these exceptions the rest of the isolates fell into highly congruent clusters, which suggested strong similarities between the two methods. Image 1

Supplementary Table 1 Additional data for the 535 *Salmonella* serovar Enteritidis isolates involved in the PT14b outbreak and used in this analysis.
Table including detailed metadata of all of the PT14b isolates involved within these analyses and details the datasets we used for each analysis, enabling readers to look-up and gain further knowledge of specific isolates and allows for these analyses to be repeated. Supplementary Table 1

Supplementary Table 2 Additional data for the 8965 *Salmonella* serovar Enteritidis isolates used to generate Fig. 4.
Table including detailed metadata of all of the serovar Enteritidis isolates used to generate Fig. 4, making the dataset easily available and enabling replication of this analysis. Supplementary Table 2

## Conflict of interest

None to declare.

## References

[bb0005] Achtman M., Wain J., Weill F.-X., Nair S., Zhou Z., Sangal V., Krauland M.G., Hale J.L., Harbottle H., Uesbeck A., Dougan G., Harrison L.H., Brisse S. (2012). Multilocus sequence typing as a replacement for serotyping in *Salmonella enterica*. PLoS Pathog..

[bb0010] Ashton P.M., Nair S., Peters T., Tewolde R., Day M., Doumith M., Green J., Jenkins C., Underwood A., Arnold C., Pinna E., Dallman T., Grant K. (2015). Revolutionising public health reference microbiology using whole genome sequencing: *Salmonella* as an exemplar. bioRxiv.

[bb0015] Ashton P.M., Nair S., Peters T.M., Bale J.A., Powell D.G., Painset A., Tewolde R., Schaefer U., Jenkins C., Dallman T.J., de Pinna E.M., Grant K.A., Salmonella Whole Genome Sequencing Implementation Group (2016). Identification of *Salmonella* for public health surveillance using whole genome sequencing. PeerJ.

[bb0020] Bakker H.C., Switt A.I.M., Cummings C.A., Hoelzer K., Degoricija L., Rodriguez-Rivera L.D., Wright E.M., Fang R., Davis M., Root T., Schoonmaker-Bopp D., Musser K.A., Villamil E., Waechter H., Kornstein L., Furtado M.R., Wiedmann M. (2011). A whole-genome single nucleotide polymorphism-based approach to trace and identify outbreaks linked to a common *Salmonella enterica* subsp. *enterica* serovar Montevideo pulsed-field gel electrophoresis type. Appl. Environ. Microbiol..

[bb0025] Bankevich A., Nurk S., Antipov D., Gurevich A.A., Dvorkin M., Kulikov A.S., Lesin V.M., Nikolenko S.I., Pham S., Prjibelski A.D., Pyshkin A.V., Sirotkin A.V., Vyahhi N., Tesler G., Alekseyev M.A., Pevzner P.A. (2012). SPAdes: a new genome assembly algorithm and its applications to single-cell sequencing. J. Comput. Biol..

[bb0030] Bratcher H.B., Corton C., Jolley K.A., Parkhill J., Maiden M.C. (2014). A gene-by-gene population genomics platform: de novo assembly, annotation and genealogical analysis of 108 representative *Neisseria meningitidis* genomes. BMC Genomics.

[bb0035] Carriço J.A., Silva-Costa C., Melo-Cristino J., Pinto F.R., De Lencastre H., Almeida J.S., Ramirez M. (2006). Illustration of a common framework for relating multiple typing methods by application to macrolide-resistant *Streptococcus pyogenes*. J. Clin. Microbiol..

[bb0040] Centers for Disease Control and Prevention (CDC) (2016). National *Salmonella* Surveillance Annual Report, 2013.

[bb0045] Chatt C., Nicholds-Trainor D., Scrivener A., Suleman S., Harvey M., Dallman T., Hawker J., Sibal B. (2017). Outbreak of *Salmonella* Enteritidis PT14b gastroenteritis at a restaurant in England: the use of molecular typing to achieve a successful prosecution. Public Health.

[bb0050] Dallman T., Inns T., Jombart T., Ashton P., Loman N., Chatt C., Messelhaeusser U., Rabsch W., Simon S., Nikisins S., Bernard H., le Hello S., Jourdan da-Silva N., Kornschober C., Mossong J., Hawkey P., de Pinna E., Grant K., Cleary P. (2016). Phylogenetic structure of European *Salmonella* Enteritidis outbreak correlates with national and international egg distribution network. Microb. Genomics.

[bb0055] De Pinna E. (2016). Use of WGS for Typing *Salmonella* at PHE.

[bb0060] Deng X., Salazar J.K., Frezet S., Maccannell D., Ribot E.M., Fields P.I., Fricke W.F., Zhang W. (2013). Genome sequence of *Salmonella enterica* serotype Tennessee strain CDC07-0191, implicated in the 2006–2007 multistate food-borne outbreak linked to peanut butter in the United States. Genome Announc..

[bb0065] Edgar R.C. (2010). Search and clustering orders of magnitude faster than BLAST. Bioinformatics.

[bb0070] Enterobase-web (2017). https://bitbucket.org/enterobase/enterobase-web/wiki/Salmonella%20Statistics.

[bb0075] Fitch W.M. (1970). Distinguishing homologous from analogous proteins. Syst. Zool..

[bb0080] Galanis E., Lo Fo Wong D.M.A., Patrick M.E., Binsztein N., Cieslik A., Chalermchikit T., Aidara-Kane A., Ellis A., Angulo F.J., Wegener H.C. (2006). Web-based surveillance and global *Salmonella* distribution, 2000–2002. Emerg. Infect. Dis..

[bb0085] Gerner-Smidt P., Hise K., Kincaid J., Hunter S., Rolando S., Hyytiä-Trees E., Ribot E.M., Swaminathan B., Taskforce P. (2006). PulseNet USA: a five-year update. Foodborne Pathog. Dis..

[bb0090] Grimont P.A.D., Weill F.-X. (2007). Antigenic Formulae of the *Salmonella* Serovars.

[bb0095] Harbottle H., White D.G., McDermott P.F., Walker R.D., Zhao S. (2006). Comparison of multilocus sequence typing, pulsed-field gel electrophoresis, and antimicrobial susceptibility typing for characterization of *Salmonella enterica* serotype Newport isolates. J. Clin. Microbiol..

[bb0100] Hendriksen R.S., Vieira A.R., Karlsmose S., Lo Fo Wong D.M.A., Jensen A.B., Wegener H.C., Aarestrup F.M. (2011). Global monitoring of *Salmonella* serovar distribution from the World Health Organization Global Foodborne Infections Network Country Data Bank: results of quality assured laboratories from 2001 to 2007. Foodborne Pathog. Dis..

[bb0105] Hoffmann M., Zhao S., Pettengill J., Luo Y., Monday S.R., Abbott J., Ayers S.L., Cinar H.N., Muruvanda T., Li C., Allard M.W., Whichard J., Meng J., Brown E.W., McDermott P.F. (2014). Comparative genomic analysis and virulence differences in closely related *Salmonella enterica* serotype Heidelberg isolates from humans, retailmeats, and animals. Genome Biol. Evol..

[bb0110] Huerta-Cepas J., Serra F., Bork P. (2016). ETE 3: reconstruction, analysis, and visualization of phylogenomic data. Mol. Biol. Evol..

[bb0115] Huson D.H., Bryant D. (2006). Application of phylogenetic networks in evolutionary studies. Mol. Biol. Evol..

[bb0120] Huson D.H., Scornavacca C. (2012). Dendroscope 3: an interactive tool for rooted phylogenetic trees and networks. Syst. Biol..

[bb0125] Hyun J., Seon Y., Sik J., Geun S., Kenneth S. (2014). Clonality and resistome analysis of KPC-producing *Klebsiella pneumoniae* strain isolated in Korea using whole genome sequencing. Biomed. Res. Int..

[bb0130] Inns T., Lane C., Peters T., Dallman T., Chatt C., McFarland N., Crook P., Bishop T., Edge J., Hawker J., Elson R., Neal K., Adak G.K., Cleary P., Outbreak Control Team (2015). A multi-country *Salmonella* Enteritidis phage type 14b outbreak associated with eggs from a German producer: “near real-time” application of whole genome sequencing and food chain investigations, United Kingdom, May to September 2014. Euro Surveill..

[bb0135] Jackson B.R., Tarr C., Strain E., Jackson K.A., Conrad A., Carleton H., Katz L.S., Stroika S., Gould L.H., Mody R.K., Silk B.J., Beal J., Chen Y., Timme R., Doyle M., Fields A., Wise M., Tillman G., Defibaugh-Chavez S., Kucerova Z., Sabol A., Roache K., Trees E., Simmons M., Wasilenko J., Kubota K., Pouseele H., Klimke W., Besser J., Brown E., Allard M., Gerner-Smidt P. (2016). Implementation of nationwide real-time whole-genome sequencing to enhance listeriosis outbreak detection and investigation. Clin. Infect. Dis..

[bb0140] Jolley K.A., Maiden M.C.J. (2010). BIGSdb: scalable analysis of bacterial genome variation at the population level. BMC Bioinf..

[bb0145] Jolley K.A., Bliss C.M., Bennett J.S., Bratcher H.B., Brehony C., Colles F.M., Wimalarathna H., Harrison O.B., Sheppard S.K., Cody A.J., Maiden M.C.J. (2012). Ribosomal multilocus sequence typing: universal characterization of bacteria from domain to strain. Microbiology.

[bb0150] Jolley K.A., Hill D.M.C., Bratcher H.B., Harrison O.B., Feavers I.M., Parkhill J., Maiden M.C.J. (2012). Resolution of a meningococcal disease outbreak from whole-genome sequence data with rapid Web-based analysis methods. J. Clin. Microbiol..

[bb0155] Kaas R.S., Leekitcharoenphon P., Aarestrup F.M., Lund O. (2014). Solving the problem of comparing whole bacterial genomes across different sequencing platforms. PLoS One.

[bb0160] Kinross P., van Alphen L., Martinez Urtaza J., Struelens M., Takkinen J., Coulombier D., Mäkelä P., Bertrand S., Mattheus W., Schmid D., Kanitz E., Rücker V., Krisztalovics K., Pászti J., Szögyényi Z., Lancz Z., Rabsch W., Pfefferkorn B., Hiller P., Mooijman K., Gossner C. (2014). Multidisciplinary investigation of a multicountry outbreak of *Salmonella* Stanley infections associated with Turkey meat in the European Union, August 2011 to January 2013. Eur. Secur..

[bb0165] Kirk M.D., Pires S.M., Black R.E., Caipo M., Crump J.A., Devleesschauwer B., Döpfer D., Fazil A., Fischer-Walker C.L., Hald T., Hall A.J., Keddy K.H., Lake R.J., Lanata C.F., Torgerson P.R., Havelaar A.H., Angulo F.J. (2015). World Health Organization estimates of the global and regional disease burden of 22 foodborne bacterial, protozoal, and viral diseases, 2010: a data synthesis. PLoS Med..

[bb0170] Kohl T.A., Diel R., Harmsen D., Rothgänger J., Meywald Walter K., Merker M., Weniger T., Niemann S. (2014). Whole-genome-based *Mycobacterium tuberculosis* surveillance: a standardized, portable, and expandable approach. J. Clin. Microbiol..

[bb0175] Kuo C.-H., Ochman H. (2010). The extinction dynamics of bacterial pseudogenes. PLoS Genet..

[bb0180] Li L., Stoeckert C.J., Roos D.S. (2003). OrthoMCL: identification of ortholog groups for eukaryotic genomes. Genome Res..

[bb0185] Maiden M.C., Bygraves J.A., Feil E., Morelli G., Russell J.E., Urwin R., Zhang Q., Zhou J., Zurth K., Caugant D.A., Feavers I.M., Achtman M., Spratt B.G. (1998). Multilocus sequence typing: a portable approach to the identification of clones within populations of pathogenic microorganisms. Proc. Natl. Acad. Sci. U. S. A..

[bb0190] Maiden M.C.J., Jansen van Rensburg M.J., Bray J.E., Earle S.G., Ford S.A., Jolley K.A., McCarthy N.D. (2013). MLST revisited: the gene-by-gene approach to bacterial genomics. Nat. Rev. Microbiol..

[bb0195] Majowicz S.E., Musto J., Scallan E., Angulo F.J., Kirk M., O'Brien S.J., Jones T.F., Fazil A., Hoekstra R.M. (2010). The global burden of nontyphoidal *Salmonella* gastroenteritis. Clin. Infect. Dis..

[bb0200] Moran-Gilad J., Prior K., Yakunin E., Harrison T.G., Underwood A., Lazarovitch T., Valinsky L., Lück C., Krux F., Agmon V., Grotto I., Harmsen D. (2015). Design and application of a core genome multilocus sequence typing scheme for investigation of Legionnaires' disease incidents. Eur. Secur..

[bb0205] Moura A., Criscuolo A., Pouseele H., Maury M.M., Leclercq A., Tarr C., Björkman J.T., Dallman T., Reimer A., Enouf V., Larsonneur E., Carleton H., Bracq-Dieye H., Katz L.S., Jones L., Touchon M., Tourdjman M., Walker M., Stroika S., Cantinelli T., Chenal-Francisque V., Kucerova Z., Rocha E.P.C., Nadon C., Grant K., Nielsen E.M., Pot B., Gerner-Smidt P., Lecuit M., Brisse S. (2016). Whole genome-based population biology and epidemiological surveillance of *Listeria monocytogenes*. Nat. Microbiol..

[bb0210] Nadon C., Van Walle I., Gerner-Smidt P., Campos J., Chinen I., Concepcion-Acevedo J., Gilpin B., Smith A.M., Man Kam K., Perez E., Trees E., Kubota K., Takkinen J., Nielsen E.M., Carleton H., FWD-NEXT Expert Panel (2017). PulseNet International: vision for the implementation of whole genome sequencing (WGS) for global food-borne disease surveillance. Euro Surveill..

[bb0215] Octavia S., Lan R. (2010). Single nucleotide polymorphism typing of global *Salmonella enterica* serovar Typhi isolates by use of a hairpin primer real-time PCR assay. J. Clin. Microbiol..

[bb0220] Pightling A.W., Petronella N., Pagotto F. (2014). Choice of reference sequence and assembler for alignment of *Listeria monocytogenes* short-read sequence data greatly influences rates of error in SNP analyses. PLoS One.

[bb0225] Price M.N., Dehal P.S., Arkin A.P. (2010). FastTree 2 – approximately maximum-likelihood trees for large alignments. PLoS One.

[bb0230] Quick J., Ashton P., Calus S., Chatt C., Gossain S., Hawker J., Nair S., Neal K., Nye K., Peters T., De Pinna E., Robinson E., Struthers K., Webber M., Catto A., Dallman T.J., Hawkey P., Loman N.J. (2015). Rapid draft sequencing and real-time nanopore sequencing in a hospital outbreak of *Salmonella*. Genome Biol..

[bb0235] Ribeiro-Gonçalves B., Francisco A.P., Vaz C., Ramirez M., Carriço J.A. (2016). PHYLOViZ Online: web-based tool for visualization, phylogenetic inference, analysis and sharing of minimum spanning trees. Nucleic Acids Res..

[bb0240] Sahl J.W., Schupp J.M., Rasko D.A., Colman R.E., Foster J.T., Keim P. (2015). Phylogenetically typing bacterial strains from partial SNP genotypes observed from direct sequencing of clinical specimen metagenomic data. Genome Med..

[bb0245] Sangal V., Harbottle H., Mazzoni C.J., Helmuth R., Guerra B., Didelot X., Paglietti B., Rabsch W., Brisse S., Weill F.-X., Roumagnac P., Achtman M. (2010). Evolution and population structure of *Salmonella enterica* serovar Newport. J. Bacteriol..

[bb0250] Scornavacca C., Zickmann F., Huson D.H. (2011). Tanglegrams for rooted phylogenetic trees and networks. Bioinformatics.

[bb0255] Severiano A., Pinto F.R., Ramirez M., Carriço J.A. (2011). Adjusted Wallace coefficient as a measure of congruence between typing methods. J. Clin. Microbiol..

[bb0260] Sukhnanand S., Alcaine S., Warnick L.D., Su W.-L., Hof J., Craver M.P.J., McDonough P., Boor K.J., Wiedmann M. (2005). DNA sequence-based subtyping and evolutionary analysis of selected *Salmonella enterica* serotypes. J. Clin. Microbiol..

[bb0265] Swaminathan B., Barrett T.J., Hunter S.B., Tauxe R.V., PulseNet Task C.D.C., Force C.P.T. (2001). PulseNet: the molecular subtyping network for foodborne bacterial disease surveillance, United States. Emerg. Infect. Dis..

[bb0270] Taylor A.J., Lappi V., Wolfgang W.J., Lapierre P., Palumbo M.J., Medus C., Boxrud D. (2015). Characterization of foodborne outbreaks of *Salmonella enterica* serovar Enteritidis with whole-genome sequencing single nucleotide polymorphism-based analysis for surveillance and outbreak detection. J. Clin. Microbiol..

[bb0275] van Belkum A., Tassios P.T., Dijkshoorn L., Haeggman S., Cookson B., Fry N.K., Fussing V., Green J., Feil E., Gerner-Smidt P., Brisse S., Struelens M. (2007). Guidelines for the validation and application of typing methods for use in bacterial epidemiology. Clin. Microbiol. Infect..

[bb0280] van Tonder A.J., Mistry S., Bray J.E., Hill D.M.C., Cody A.J., Farmer C.L., Klugman K.P., von Gottberg A., Bentley S.D., Parkhill J., Jolley K.A., Maiden M.C.J., Brueggemann A.B. (2014). Defining the estimated core genome of bacterial populations using a Bayesian decision model. PLoS Comput. Biol..

[bb0285] Velge P., Cloeckaert A., Barrow P. (2005). Emergence of *Salmonella* epidemics: the problems related to *Salmonella enterica* serotype Enteritidis and multiple antibiotic resistance in other major serotypes. Vet. Res..

[bb0290] Yoshida C.E., Kruczkiewicz P., Laing C.R., Lingohr E.J., Gannon V.P.J., Nash J.H.E., Taboada E.N. (2016). The *Salmonella* in silico typing resource (SISTR): an open web-accessible tool for rapidly typing and subtyping draft *Salmonella* genome assemblies. PLoS One.

[bb0295] Zhou Z., McCann A., Litrup E., Murphy R., Cormican M., Fanning S., Brown D., Guttman D.S., Brisse S., Achtman M. (2013). Neutral genomic microevolution of a recently emerged pathogen, *Salmonella enterica* serovar Agona. PLoS Genet..

