# Supplementary figures and images for: Comparative analysis of core genome MLST and SNP typing within a European Salmonella serovar Enteritidis outbreak
Source: Int J Food Microbiol. 2018 Jun 2;274:1–11. doi: 10.1016/j.ijfoodmicro.2018.02.023 (PMC5899760; doi:10.1016/j.ijfoodmicro.2018.02.023)

A (cgMLST)

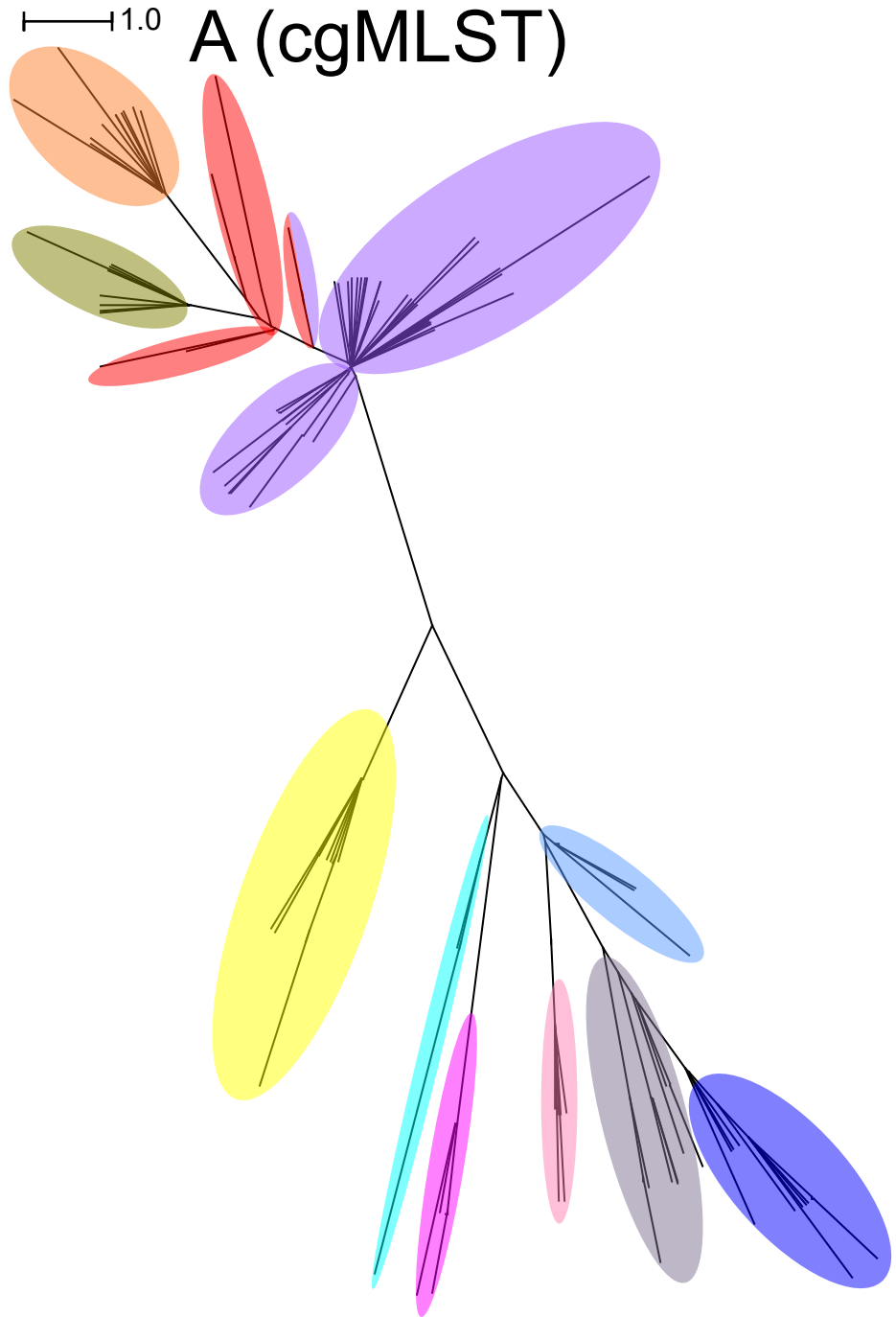

B (SNPs)

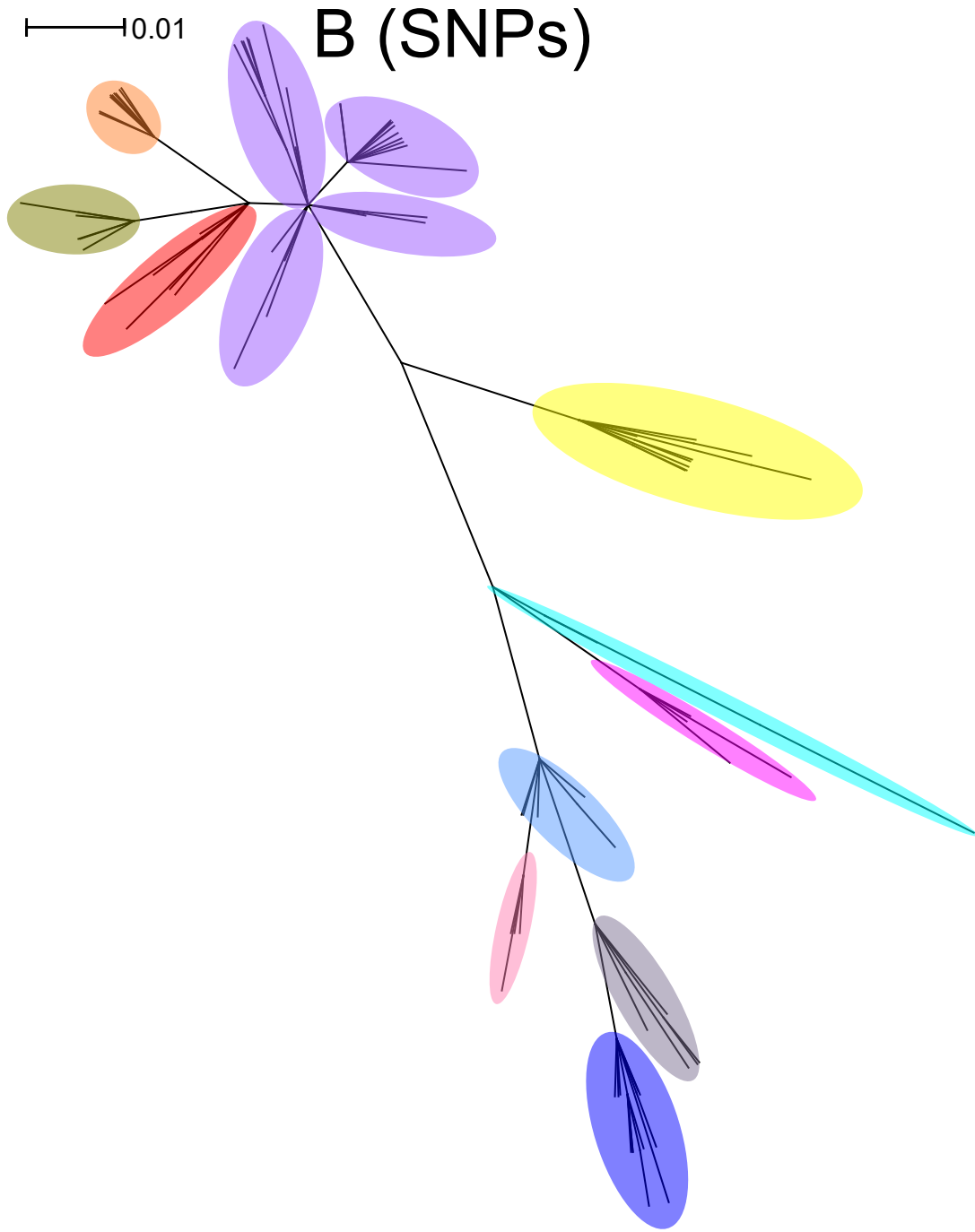

Supplement: Supplementary Fig. 1 — Core genome neighbour-joining tree (A) and single nucleotide polymorphism neighbour-joining tree (B). A: neighbour-joining tree of the 386 isolates used to create the Tanglegram (Fig. 1) calculated using the cgMLST scheme which consists of 3002 loci and is available in the EnteroBase database. The tree was drawn using SplitsTree4 (Huson and Bryant, 2006). B: neighbour-joining tree of the 386 isolates used to create the Tanglegram (Fig. 1) calculated using the PHE SNP pipeline. The tree was drawn using SplitsTree4 (Huson and Bryant, 2006). The trees, drawn within SplitsTree4 (Huson and Bryant, 2006) were coloured based on closely related groups of isolates. The overall topology of the trees and the majority of the clusters, demonstrated strong congruence between SNP and cgMLST based approaches. Minor differences were observed, such as the purple group was split into four clusters via SNPs and only two via cgMLST, none of which were directly congruent between the two methods. However, all isolates within the purple clusters were more closely related to each other than their neighbours. There were also two isolates, split into the red and purple groups by SNPs, which were very closely related via cgMLST. Despite these exceptions the rest of the isolates fell into highly congruent clusters, which suggested strong similarities between the two methods. [file mmc1.pdf]
