# Supplementary material for: Comparative analysis of core genome MLST and SNP typing within a European Salmonella serovar Enteritidis outbreak
Source: Int J Food Microbiol. 2018 Jun 2;274:1–11. doi: 10.1016/j.ijfoodmicro.2018.02.023 (PMC5899760; doi:10.1016/j.ijfoodmicro.2018.02.023)
Supplement: Supplementary Table 1 — Additional data for the 535 Salmonella serovar Enteritidis isolates involved in the PT14b outbreak and used in this analysis. Table including detailed metadata of all of the PT14b isolates involved within these analyses and details the datasets we used for each analysis, enabling readers to look-up and gain further knowledge of specific isolates and allows for these analyses to be repeated. [file mmc2.pdf]

Enterobase Barcode	Name	Accession number	Collection Year	Collection Month	Collection Day	Continent	Country	Serovar	ST	SNP Addresses	dataset A	dataset B	dataset C
SAL_CA4860AA	34199	SRR1965044	2014	7	24	Europe	United Kingdom	Enteritidis	2066	1.2.3.38.38.38.367	✓	✓	✓
SAL_CA4812AA	40793	SRR1965094	2014	8	12	Europe	United Kingdom	Enteritidis	26206	1.2.3.38.38.38.395	✓	✓	✓
SAL_CA4740AA	40799	SRR1965168	2014	8	15	Europe	United Kingdom	Enteritidis	26270	1.2.3.38.38.38.367	✓	✓	✓
SAL_CA4651AA	25341	SRR1965266	2014	6	25	Europe	United Kingdom	Enteritidis	26347	1.2.3.38.38.38.38	✓	✓	✓
SAL_CA4566AA	50361	SRR1965353	2014	9	4	Europe	United Kingdom	Enteritidis	2066	1.2.3.38.38.38.413	✓	✓	✓
SAL_CA4560AA	38361	SRR1965359	2014	7	28	Europe	United Kingdom	Enteritidis	2066	1.2.3.38.38.38.367	✓	✓	✓
SAL_CA4550AA	38314	SRR1965369	2014	7	29	Europe	United Kingdom	Enteritidis	26438	1.2.3.38.38.38.367	✓	✓	✓
SAL_CA4544AA	32523	SRR1965375	2014	7	15	Europe	United Kingdom	Enteritidis	7153	1.2.3.38.38.38.393	✓	✓	✓
SAL_CA4510AA	38420	SRR1965410	2014	7	29	Europe	United Kingdom	Enteritidis	26468	1.2.3.38.38.38.413	✓	✓	✓
SAL_CA4496AA	37823	SRR1965424	2014	7	30	Europe	United Kingdom	Enteritidis	26481	1.2.3.38.38.38.393	✓	✓	✓
SAL_CA4462AA	38418	SRR1965458	2014	7	30	Europe	United Kingdom	Enteritidis	26511	1.2.3.38.38.38.413	✓	✓	✓
SAL_CA4457AA	38311	SRR1965464	2014	7	29	Europe	United Kingdom	Enteritidis	26516	1.2.3.38.38.38.367	✓	✓	✓
SAL_CA4417AA	37832	SRR1965506	2014	7	31	Europe	United Kingdom	Enteritidis	26550	1.2.3.38.38.38.2451	✓	✓	✓
SAL_CA4391AA	37819	SRR1965533	2014	7	29	Europe	United Kingdom	Enteritidis	2066	1.2.3.38.38.38.413	✓	✓	✓
SAL_CA4375AA	21777	SRR1965549	2014	6	12	Europe	United Kingdom	Enteritidis	26585	1.2.3.38.38.38.38	✓	✓	✓
SAL_CA4343AA	38766	SRR1965590	2014	8	5	Europe	United Kingdom	Enteritidis	2066	1.2.3.38.38.38.367	✓	✓	✓
SAL_CA4339AA	38426	SRR1965596	2014	7	28	Europe	United Kingdom	Enteritidis	26616	1.2.3.38.38.38.367	✓	✓	✓
SAL_CA4312AA	38421	SRR1965623	2014	7	29	Europe	United Kingdom	Enteritidis	26639	1.2.3.38.38.38.413	✓	✓	✓
SAL_CA4259AA	31929	SRR1965679	2014	7	21	Europe	United Kingdom	Enteritidis	26685	1.2.3.38.38.38.367	✓	✓	✓
SAL_CA4247AA	38425	SRR1965692	2014	7	29	Europe	United Kingdom	Enteritidis	26696	1.2.3.38.38.38.1072	✓	✓	✓
SAL_CA4240AA	37828	SRR1965700	2014	7	28	Europe	United Kingdom	Enteritidis	26702	1.2.3.38.38.38.367	✓	✓	✓
SAL_CA4176AA	36522	SRR1965772	2014	7	23	Europe	United Kingdom	Enteritidis	26757	1.2.3.38.38.38.2621	✓	✓	✓
SAL_CA4151AA	37808	SRR1965800	2014	7	31	Europe	United Kingdom	Enteritidis	7612	1.2.3.38.38.38.367	✓	✓	✓
SAL_CA4000AA	34176	SRR1965958	2014	7	21	Europe	United Kingdom	Enteritidis	26909	1.2.3.38.38.38.393	✓	✓	✓
SAL_CA3971AA	31976	SRR1965987	2014	7	23	Europe	United Kingdom	Enteritidis	26936	1.2.3.38.38.38.393	✓	✓	✓
SAL_CA3877AA	32462	SRR1966087	2014	7	15	Europe	United Kingdom	Enteritidis	27020	1.2.3.38.38.38.367	✓	✓	✓
SAL_CA3860AA	31973	SRR1966105	2014	7	22	Europe	United Kingdom	Enteritidis	27036	1.2.3.38.38.38.393	✓	✓	✓
SAL_CA3836AA	37831	SRR1966129	2014	7	30	Europe	United Kingdom	Enteritidis	27057	1.2.3.38.38.38.367	✓	✓	✓
SAL_CA3814AA	31967	SRR1966151	2014	7	26	Europe	United Kingdom	Enteritidis	2066	1.2.3.38.38.38.367	✓	✓	✓
SAL_CA3772AA	39453	SRR1966194	2014	7	29	Europe	United Kingdom	Enteritidis	27108	1.2.3.38.38.38.367	✓	✓	✓
SAL_CA3742AA	46580	SRR1966225	2014	7	17	Europe	United Kingdom	Enteritidis	27137	1.2.3.38.38.38.367	✓	✓	✓
SAL_CA3727AA	31980	SRR1966240	2014	7	25	Europe	United Kingdom	Enteritidis	2066	1.2.3.38.38.38.367	✓	✓	✓
SAL_CA3628AA	34178	SRR1966339	2014	7	17	Europe	United Kingdom	Enteritidis	26229	1.2.3.38.38.38.393	✓	✓	✓
SAL_CA3618AA	36521	SRR1966349	2014	7	24	Europe	United Kingdom	Enteritidis	7612	1.2.3.38.38.38.367	✓	✓	✓
SAL_CA3584AA	46585	SRR1966383	2014	7	18	Europe	United Kingdom	Enteritidis	27283	1.2.3.38.38.38.393	✓	✓	✓
SAL_CA3580AA	31928	SRR1966387	2014	7	21	Europe	United Kingdom	Enteritidis	27287	1.2.3.38.38.38.367	✓	✓	✓
SAL_CA3518AA	21778	SRR1966448	2014	6	12	Europe	United Kingdom	Enteritidis	674	1.2.3.38.38.38.38	✓	✓	✓
SAL_CA3488AA	68909	SRR1966478	2014	6	24	Europe	United Kingdom	Enteritidis	27371	1.2.3.38.38.38.38	✓	✓	✓
SAL_CA3480AA	38318	SRR1966486	2014	7	28	Europe	United Kingdom	Enteritidis	27379	1.2.3.38.38.38.367	✓	✓	✓
SAL_CA3459AA	37812	SRR1966507	2014	7	31	Europe	United Kingdom	Enteritidis	27399	1.2.3.38.38.38.367	✓	✓	✓
SAL_CA3413AA	31922	SRR1966554	2014	7	21	Europe	United Kingdom	Enteritidis	7612	1.2.3.38.38.38.367	✓	✓	✓
SAL_CA3385AA	23473	SRR1966582	2014	6	13	Europe	United Kingdom	Enteritidis	27459	1.2.3.38.38.38.1116	✓	✓	✓
SAL_CA3363AA	36498	SRR1966605	2014	7	28	Europe	United Kingdom	Enteritidis	27476	1.2.3.38.38.38.367	✓	✓	✓
SAL_CA3279AA	39455	SRR1966692	2014	7	30	Europe	United Kingdom	Enteritidis	27552	1.2.3.38.38.38.367	✓	✓	✓
SAL_CA3247AA	34194	SRR1966724	2014	7	24	Europe	United Kingdom	Enteritidis	7153	1.2.3.38.38.38.393	✓	✓	✓

SAL_CA3216AA	38316	SRR1966755	2014	7	28	Europe	United Kingdom	Enteritidis	27608	1.2.3.38.38.38.367	✓	✓	✓
SAL_CA3141AA	32459	SRR1966830	2014	7	17	Europe	United Kingdom	Enteritidis	2066	1.2.3.38.38.38.367	✓	✓	✓
SAL_CA3120AA	34189	SRR1966851	2014	7	25	Europe	United Kingdom	Enteritidis	27688	1.2.3.38.38.38.367	✓	✓	✓
SAL_CA3048AA	37833	SRR1966923	2014	7	30	Europe	United Kingdom	Enteritidis	27750	1.2.3.38.38.38.367	✓	✓	✓
SAL_CA3034AA	36525	SRR1966937	2014	7	24	Europe	United Kingdom	Enteritidis	27763	1.2.3.38.38.38.393	✓	✓	✓
SAL_CA3002AA	38313	SRR1966969	2014	7	28	Europe	United Kingdom	Enteritidis	27792	1.2.3.38.38.38.367	✓	✓	✓
SAL_CA3000AA	38319	SRR1966971	2014	7	28	Europe	United Kingdom	Enteritidis	27794	1.2.3.38.38.38.393	✓	✓	✓
SAL_CA2969AA	36501	SRR1967002	2014	7	17	Europe	United Kingdom	Enteritidis	27822	1.2.3.38.38.38.393	✓	✓	✓
SAL_CA2943AA	38419	SRR1967028	2014	7	29	Europe	United Kingdom	Enteritidis	27844	1.2.3.38.38.38.413	✓	✓	✓
SAL_CA2932AA	46591	SRR1967039	2014	7	18	Europe	United Kingdom	Enteritidis	27852	1.2.3.38.38.38.367	✓	✓	✓
SAL_CA2891AA	34186	SRR1967080	2014	7	25	Europe	United Kingdom	Enteritidis	2066	1.2.3.38.38.38.367	✓	✓	✓
SAL_CA2886AA	32472	SRR1967085	2014	7	18	Europe	United Kingdom	Enteritidis	2066	1.2.3.38.38.38.367	✓	✓	✓
SAL_CA2882AA	36524	SRR1967089	2014	7	22	Europe	United Kingdom	Enteritidis	27888	1.2.3.38.38.38.367	✓	✓	✓
SAL_CA2872AA	21776	SRR1967099	2014	6	3	Europe	United Kingdom	Enteritidis	27897	1.2.3.38.38.38.38	✓	✓	✓
SAL_CA2811AA	23472	SRR1967160	2014	6	13	Europe	United Kingdom	Enteritidis	27950	1.2.3.38.38.38.38	✓	✓	✓
SAL_CA2801AA	37807	SRR1967170	2014	7	28	Europe	United Kingdom	Enteritidis	27959	1.2.3.38.38.38.2542	✓	✓	✓
SAL_CA2797AA	34185	SRR1967174	2014	7	22	Europe	United Kingdom	Enteritidis	7153	1.2.3.38.38.38.393	✓	✓	✓
SAL_CA2792AA	39450	SRR1967179	2014	7	23	Europe	United Kingdom	Enteritidis	27967	1.2.3.38.38.38.367	✓	✓	✓
SAL_CA2786AA	34196	SRR1967185	2014	7	25	Europe	United Kingdom	Enteritidis	27973	1.2.3.38.38.38.367	✓	✓	✓
SAL_CA2743AA	21770	SRR1967228	2014	6	6	Europe	United Kingdom	Enteritidis	674	1.2.3.38.38.38.38	✓	✓	✓
SAL_CA2688AA	24040	SRR1967283	2014	6	20	Europe	United Kingdom	Enteritidis	28055	1.2.3.38.38.38.604	✓	✓	✓
SAL_CA2685AA	34197	SRR1967286	2014	7	24	Europe	United Kingdom	Enteritidis	2066	1.2.3.38.38.38.367	✓	✓	✓
SAL_CA2664AA	24986	SRR1967307	2014	6	28	Europe	United Kingdom	Enteritidis	28076	1.2.3.38.38.38.472	✓	✓	✓
SAL_CA2593AA	38317	SRR1967378	2014	7	28	Europe	United Kingdom	Enteritidis	28141	1.2.3.38.38.38.367	✓	✓	✓
SAL_CA2588AA	34184	SRR1967383	2014	7	22	Europe	United Kingdom	Enteritidis	28146	1.2.3.38.38.38.393	✓	✓	✓
SAL_CA2509AA	32463	SRR1967462	2014	7	17	Europe	United Kingdom	Enteritidis	28214	1.2.3.38.38.38.367	✓	✓	✓
SAL_CA2492AA	37809	SRR1967479	2014	7	29	Europe	United Kingdom	Enteritidis	7612	1.2.3.38.38.38.367	✓	✓	✓
SAL_CA2423AA	31923	SRR1967548	2014	7	23	Europe	United Kingdom	Enteritidis	28289	1.2.3.38.38.38.367	✓	✓	✓
SAL_CA2371AA	46589	SRR1967600	2014	7	17	Europe	United Kingdom	Enteritidis	28335	1.2.3.38.38.38.367	✓	✓	✓
SAL_CA2362AA	36464	SRR1967609	2014	7	25	Europe	United Kingdom	Enteritidis	2066	1.2.3.38.38.38.367	✓	✓	✓
SAL_CA2205AA	38371	SRR1967766	2014	7	28	Europe	United Kingdom	Enteritidis	28478	1.2.3.38.38.38.367	✓	✓	✓
SAL_CA2200AA	36526	SRR1967771	2014	7	28	Europe	United Kingdom	Enteritidis	2066	1.2.3.38.38.38.367	✓	✓	✓
SAL_CA2199AA	36531	SRR1967772	2014	7	29	Europe	United Kingdom	Enteritidis	28483	1.2.3.38.38.38.2321	✓	✓	✓
SAL_CA2191AA	24038	SRR1967780	2014	6	23	Europe	United Kingdom	Enteritidis	28491	1.2.3.38.38.38.472	✓	✓	✓
SAL_CA2184AA	31974	SRR1967787	2014	7	25	Europe	United Kingdom	Enteritidis	28497	1.2.3.38.38.38.393	✓	✓	✓
SAL_CA2113AA	31968	SRR1967858	2014	7	26	Europe	United Kingdom	Enteritidis	28559	1.2.3.38.38.38.367	✓	✓	✓
SAL_CA2084AA	38332	SRR1967887	2014	7	25	Europe	United Kingdom	Enteritidis	2066	1.2.3.38.38.38.1809	✓	✓	✓
SAL_CA1889AA	32466	SRR1967989	2014	7	18	Europe	United Kingdom	Enteritidis	28675	1.2.3.38.38.38.393	✓	✓	✓
SAL_CA1784AA	37810	SRR1968043	2014	7	29	Europe	United Kingdom	Enteritidis	28721	1.2.3.38.38.38.367	✓	✓	✓
SAL_CA1771AA	38417	SRR1968051	2014	7	28	Europe	United Kingdom	Enteritidis	28729	1.2.3.38.38.38.413	✓	✓	✓
SAL_CA1654AA	37827	SRR1968120	2014	7	24	Europe	United Kingdom	Enteritidis	28815	1.2.3.38.38.38.367	✓	✓	✓
SAL_CA1478AA	31917	SRR1968213	2014	7	18	Europe	United Kingdom	Enteritidis	28978	1.2.3.38.38.38.668	✓	✓	✓
SAL_CA1253AA	46590	SRR1968332	2014	7	17	Europe	United Kingdom	Enteritidis	29082	1.2.3.38.38.38.367	✓	✓	✓
SAL_CA1071AA	46579	SRR1968486	2014	7	17	Europe	United Kingdom	Enteritidis	29224	1.2.3.38.38.38.367	✓	✓	✓
SAL_CA0907AA	40235	SRR1968516	2014	8	12	Europe	United Kingdom	Enteritidis	29251	1.2.3.38.38.38.413	✓	✓	✓
SAL_CA0885AA	37817	SRR1968529	2014	7	26	Europe	United Kingdom	Enteritidis	27851	1.2.3.38.38.38.367	✓	✓	✓

SAL_CA0837AA	37825	SRR1968556	2014	7	28	Europe	United Kingdom	Enteritidis	29305	1.2.3.38.38.38.808	✓	✓	✓
SAL_CA0821AA	31972	SRR1968564	2014	7	25	Europe	United Kingdom	Enteritidis	2066	1.2.3.38.38.38.367	✓	✓	✓
SAL_CA0770AA	38422	SRR1968589	2014	7	25	Europe	United Kingdom	Enteritidis	29363	1.2.3.38.38.38.413	✓	✓	✓
SAL_CA0763AA	34188	SRR1968592	2014	7	25	Europe	United Kingdom	Enteritidis	29369	1.2.3.38.38.38.367	✓	✓	✓
SAL_CA0694AA	37813	SRR1968627	2014	7	31	Europe	United Kingdom	Enteritidis	29430	1.2.3.38.38.38.367	✓	✓	✓
SAL_CA0644AA	38344	SRR1968654	2014	7	28	Europe	United Kingdom	Enteritidis	26229	1.2.3.38.38.38.393	✓	✓	✓
SAL_CA0472AA	31966	SRR1968746	2014	7	25	Europe	United Kingdom	Enteritidis	29612	1.2.3.38.38.38.367	✓	✓	✓
SAL_CA0378AA	37811	SRR1968796	2014	7	28	Europe	United Kingdom	Enteritidis	29695	1.2.3.38.38.38.367	✓	✓	✓
SAL_CA0306AA	38325	SRR1968832	2014	7	30	Europe	United Kingdom	Enteritidis	29759	1.2.3.38.38.38.367	✓	✓	✓
SAL_CA0244AA	23462	SRR1968861	2014	6	10	Europe	United Kingdom	Enteritidis	29811	1.2.3.38.38.38.38	✓	✓	✓
SAL_CA0199AA	41984	SRR1968882	2014	8	14	Europe	United Kingdom	Enteritidis	29839	1.2.3.38.38.38.2248	✓	✓	✓
SAL_CA0163AA	34175	SRR1968899	2014	7	22	Europe	United Kingdom	Enteritidis	29860	1.2.3.38.38.38.537	✓	✓	✓
SAL_CA0150AA	34177	SRR1968906	2014	7	22	Europe	United Kingdom	Enteritidis	7153	1.2.3.38.38.38.393	✓	✓	✓
SAL_CA0106AA	38369	SRR1968929	2014	7	30	Europe	United Kingdom	Enteritidis	29905	1.2.3.38.38.38.367	✓	✓	✓
SAL_BA9962AA	32471	SRR1969002	2014	7	20	Europe	United Kingdom	Enteritidis	30022	1.2.3.38.38.38.367	✓	✓	✓
SAL_BA9960AA	23493	SRR1969003	2014	6	19	Europe	United Kingdom	Enteritidis	30024	1.2.3.38.38.38.38	✓	✓	✓
SAL_BA9938AA	24039	SRR1969015	2014	6	23	Europe	United Kingdom	Enteritidis	30043	1.2.3.38.38.38.472	✓	✓	✓
SAL_BA9923AA	36395	SRR1969024	2014	8	3	Europe	United Kingdom	Enteritidis	30055	1.2.3.38.38.38.367	✓	✓	✓
SAL_BA9834AA	46586	SRR1969069	2014	7	14	Europe	United Kingdom	Enteritidis	30125	1.2.3.38.38.38.393	✓	✓	✓
SAL_BA9822AA	34181	SRR1969076	2014	7	22	Europe	United Kingdom	Enteritidis	30135	1.2.3.38.38.38.393	✓	✓	✓
SAL_BA9738AA	36518	SRR1969134	2014	7	23	Europe	United Kingdom	Enteritidis	2066	1.2.3.38.38.38.367	✓	✓	✓
SAL_BA9594AA	34179	SRR1969206	2014	7	21	Europe	United Kingdom	Enteritidis	30306	1.2.3.38.38.38.393	✓	✓	✓
SAL_BA9466AA	39452	SRR1969276	2014	7	28	Europe	United Kingdom	Enteritidis	30409	1.2.3.38.38.38.367	✓	✓	✓
SAL_BA9440AA	38315	SRR1969298	2014	7	28	Europe	United Kingdom	Enteritidis	29305	1.2.3.38.38.38.367	✓	✓	✓
SAL_BA9436AA	37798	SRR1969301	2014	7	28	Europe	United Kingdom	Enteritidis	30436	1.2.3.38.38.38.367	✓	✓	✓
SAL_BA9403AA	39454	SRR1969334	2014	7	28	Europe	United Kingdom	Enteritidis	2066	1.2.3.38.38.38.367	✓	✓	✓
SAL_BA9397AA	26641	SRR1969340	2014	6	26	Europe	United Kingdom	Enteritidis	26971	1.2.3.38.38.38.472	✓	✓	✓
SAL_BA9388AA	27771	SRR1969349	2014	6	26	Europe	United Kingdom	Enteritidis	30473	1.2.3.38.38.38.472	✓	✓	✓
SAL_BA9365AA	46583	SRR1969372	2014	7	14	Europe	United Kingdom	Enteritidis	30496	1.2.3.38.38.38.393	✓	✓	✓
SAL_BA9337AA	37834	SRR1969400	2014	7	31	Europe	United Kingdom	Enteritidis	30520	1.2.3.38.38.38.367	✓	✓	✓
SAL_BA9321AA	38779	SRR1969416	2014	8	7	Europe	United Kingdom	Enteritidis	2066	1.2.3.38.38.38.413	✓	✓	✓
SAL_BA9293AA	21783	SRR1969436	2014	6	5	Europe	United Kingdom	Enteritidis	30553	1.2.3.38.38.38.38	✓	✓	✓
SAL_BA9114AA	21779	SRR1969526	2014	6	12	Europe	United Kingdom	Enteritidis	674	1.2.3.38.38.38.38	✓	✓	✓
SAL_BA8951AA	36530	SRR1969546	2014	7	28	Europe	United Kingdom	Enteritidis	2066	1.2.3.38.38.38.367	✓	✓	✓
SAL_BA8852AA	32460	SRR1969597	2014	7	16	Europe	United Kingdom	Enteritidis	30907	1.2.3.38.38.38.367	✓	✓	✓
SAL_BA8844AA	31652	SRR1969601	2014	7	9	Europe	United Kingdom	Enteritidis	30913	1.2.3.38.38.38.393	✓	✓	✓
SAL_BA8588AA	37799	SRR1969732	2014	8	1	Europe	United Kingdom	Enteritidis	31130	1.2.3.38.38.38.367	✓	✓	✓
SAL_BA8536AA	36516	SRR1969758	2014	7	21	Europe	United Kingdom	Enteritidis	27476	1.2.3.38.38.38.367	✓	✓	✓
SAL_BA8449AA	37829	SRR1969802	2014	7	28	Europe	United Kingdom	Enteritidis	28289	1.2.3.38.38.38.367	✓	✓	✓
SAL_BA8444AA	37815	SRR1969805	2014	7	30	Europe	United Kingdom	Enteritidis	28289	1.2.3.38.38.38.367	✓	✓	✓
SAL_BA8378AA	37826	SRR1969838	2014	7	28	Europe	United Kingdom	Enteritidis	2066	1.2.3.38.38.38.367	✓	✓	✓
SAL_BA8205AA	34204	SRR1969924	2014	7	28	Europe	United Kingdom	Enteritidis	31423	1.2.3.38.38.38.1595	✓	✓	✓
SAL_BA8150AA	32467	SRR1969951	2014	7	19	Europe	United Kingdom	Enteritidis	31456	1.2.3.38.38.38.703	✓	✓	✓
SAL_BA7885AA	38410	SRR1970082	2014	8	1	Europe	United Kingdom	Enteritidis	31578	1.2.3.38.38.38.483	✓	✓	✓
SAL_BA7786AA	38349	SRR1970133	2014	7	27	Europe	United Kingdom	Enteritidis	31658	1.2.3.38.38.38.1378	✓	✓	✓
SAL_BA7695AA	34182	SRR1970179	2014	7	24	Europe	United Kingdom	Enteritidis	31704	1.2.3.38.38.38.367	✓	✓	✓

SAL_BA7689AA	37830	SRR1970182	2014	7	30	Europe	United Kingdom	Enteritidis	31707	1.2.3.38.38.38.367	✓	✓	✓
SAL_BA7611AA	31927	SRR1970220	2014	7	22	Europe	United Kingdom	Enteritidis	2066	1.2.3.38.38.38.367	✓	✓	✓
SAL_BA7600AA	46584	SRR1970226	2014	7	17	Europe	United Kingdom	Enteritidis	31754	1.2.3.38.38.38.393	✓	✓	✓
SAL_BA7479AA	29391	SRR1970288	2014	7	14	Europe	United Kingdom	Enteritidis	31812	1.2.3.38.38.38.393	✓	✓	✓
SAL_BA7469AA	37824	SRR1970294	2014	7	27	Europe	United Kingdom	Enteritidis	2066	1.2.3.38.38.38.367	✓	✓	✓
SAL_BA7461AA	38345	SRR1970302	2014	7	28	Europe	United Kingdom	Enteritidis	7153	1.2.3.38.38.38.393	✓	✓	✓
SAL_BA7442AA	46581	SRR1970321	2014	7	18	Europe	United Kingdom	Enteritidis	31840	1.2.3.38.38.38.367	✓	✓	✓
SAL_EA8049AA	18557	SRR3048580	2014	6	6	Europe	United Kingdom	Enteritidis	6818	1.2.3.38.38.38.38	✓	✓	✓
SAL_EA8008AA	56935	SRR3048716	2014	8	1	Europe	United Kingdom	Enteritidis	2066	1.2.3.38.38.38.413	✓	✓	✓
SAL_EA7962AA	21810	SRR3048785	2014	6	6	Europe	United Kingdom	Enteritidis	6902	1.2.3.38.38.38.38	✓	✓	✓
SAL_EA7824AA	29970	SRR3048933	2014	7	21	Europe	United Kingdom	Enteritidis	7033	1.2.3.38.38.38.367	✓	✓	✓
SAL_EA7699AA	29280	SRR3049070	2014	7	11	Europe	United Kingdom	Enteritidis	7153	1.2.3.38.38.38.393	✓	✓	✓
SAL_EA7675AA	21117	SRR3049094	2014	6	4	Europe	United Kingdom	Enteritidis	7177	1.2.3.38.38.38.38	✓	✓	✓
SAL_EA7665AA	20909	SRR3049104	2014	4	6	Europe	United Kingdom	Enteritidis	674	1.2.3.38.38.38.38	✓	✓	✓
SAL_EA7540AA	21868	SRR3049229	2014	6	11	Europe	United Kingdom	Enteritidis	7309	1.2.3.38.38.38.2660	✓	✓	✓
SAL_EA7504AA	18555	SRR3049265	2014	6	6	Europe	United Kingdom	Enteritidis	7344	1.2.3.38.38.38.38	✓	✓	✓
SAL_EA7467AA	21116	SRR3049304	2014	6	4	Europe	United Kingdom	Enteritidis	7381	1.2.3.38.38.38.1775	✓	✓	✓
SAL_EA7408AA	18554	SRR3049366	2014	6	6	Europe	United Kingdom	Enteritidis	7435	1.2.3.38.38.38.38	✓	✓	✓
SAL_EA7367AA	29281	SRR3049409	2014	7	11	Europe	United Kingdom	Enteritidis	7473	1.2.3.38.38.38.393	✓	✓	✓
SAL_EA7334AA	20925	SRR3049443	2014	6	6	Europe	United Kingdom	Enteritidis	674	1.2.3.38.38.38.38	✓	✓	✓
SAL_EA7277AA	20926	SRR3049503	2014	6	7	Europe	United Kingdom	Enteritidis	7555	1.2.3.38.38.38.2103	✓	✓	✓
SAL_EA7237AA	20927	SRR3049543	2014	6	8	Europe	United Kingdom	Enteritidis	7593	1.2.3.38.38.38.38	✓	✓	✓
SAL_EA7167AA	34190	SRR3049621	2014	7	24	Europe	United Kingdom	Enteritidis	7660	1.2.3.38.38.38.367	✓	✓	✓
SAL_EA7135AA	21843	SRR3049661	2014	6	1	Europe	United Kingdom	Enteritidis	674	1.2.3.38.38.38.38	✓	✓	✓
SAL_EA7086AA	29972	SRR3049720	2014	7	21	Europe	United Kingdom	Enteritidis	7736	1.2.3.38.38.38.367	✓	✓	✓
SAL_EA7083AA	21844	SRR3049723	2014	6	10	Europe	United Kingdom	Enteritidis	7739	1.2.3.38.38.38.38	✓	✓	✓
SAL_EA6989AA	21114	SRR3049844	2014	6	4	Europe	United Kingdom	Enteritidis	7827	1.2.3.38.38.38.38	✓	✓	✓
SAL_EA6943AA	21113	SRR3049901	2014	6	4	Europe	United Kingdom	Enteritidis	7870	1.2.3.38.38.38.1449	✓	✓	✓
SAL_EA6911AA	21815	SRR3049937	2014	6	9	Europe	United Kingdom	Enteritidis	7901	1.2.3.38.38.38.38	✓	✓	✓
SAL_EA6910AA	21115	SRR3049939	2014	6	4	Europe	United Kingdom	Enteritidis	7902	1.2.3.38.38.38.38	✓	✓	✓
SAL_EA9314AA	38368	SRR3120716	2014	7	31	Europe	United Kingdom	Enteritidis	5791	1.2.3.38.38.38.367	✓	✓	✓
SAL_CA2175AA	21767	SRR1967796	2014	6	4	Europe	United Kingdom	Enteritidis	28504	1.2.3.38.38.38.38	✓		✓
SAL_CA4703AA	31926	SRR1965207	2014	7		Europe	United Kingdom	Enteritidis	26303				✓
SAL_CA2567AA	36499	SRR1967404	2014	7		Europe	United Kingdom	Enteritidis	28167				✓
SAL_CA2505AA	38312	SRR1967466	2014	8		Europe	United Kingdom	Enteritidis	28218				✓
SAL_CA0560AA	31924	SRR1968701	2014	7		Europe	United Kingdom	Enteritidis	29540				✓
SAL_CA0240AA	31930	SRR1968863	2014	7		Europe	United Kingdom	Enteritidis	29815				✓
SAL_BA7675AA	57104	SRR1970189	2014	8		Europe	United Kingdom	Enteritidis	31713				✓
SAL_EA7611AA	21813	SRR3049158				Europe	United Kingdom	Enteritidis	7238				✓
SAL_EA9317AA	36461	SRR3120711				Europe	United Kingdom	Enteritidis	5789				✓
SAL_CA6794AA	44683	SRR1957774	2014	8	29	Europe	United Kingdom	Enteritidis	24493	1.2.3.38.38.38.1469	✓	✓	
SAL_CA6754AA	45262	SRR1957814	2014	9	1	Europe	United Kingdom	Enteritidis	24533	1.2.3.38.38.38.461	✓	✓	
SAL_CA6748AA	49751	SRR1957820	2014	9	15	Europe	United Kingdom	Enteritidis	24538	1.2.3.38.38.38.2163	✓	✓	
SAL_CA6726AA	62582	SRR1957842	2014	9	2	Europe	United Kingdom	Enteritidis	24558	1.2.3.38.38.38.1404	✓	✓	
SAL_CA6716AA	62574	SRR1957852	2014	9	1	Europe	United Kingdom	Enteritidis	24568	1.2.3.38.38.38.461	✓	✓	
SAL_CA6691AA	62581	SRR1957877	2014	8	27	Europe	United Kingdom	Enteritidis	24591	1.2.3.38.38.38.550	✓	✓	

SAL_CA6680AA	48262	SRR1957888	2014	9	5	Europe	United Kingdom	Enteritidis	24601	1.2.3.38.38.38.367	✓	✓
SAL_CA6582AA	46118	SRR1957986	2014	8	20	Europe	United Kingdom	Enteritidis	24693	1.2.3.38.38.38.550	✓	✓
SAL_CA6557AA	58510	SRR1958011	2014	10	2	Europe	United Kingdom	Enteritidis	7317	1.2.3.38.38.38.655	✓	✓
SAL_CA6541AA	46085	SRR1958028	2014	8	19	Europe	United Kingdom	Enteritidis	24732	1.2.3.38.38.38.550	✓	✓
SAL_CA6536AA	50109	SRR1958033	2014	9	16	Europe	United Kingdom	Enteritidis	24738	1.2.3.38.38.38.787	✓	✓
SAL_CA6517AA	57623	SRR1958052	2014	8	8	Europe	United Kingdom	Enteritidis	2066	1.2.3.38.38.38.367	✓	✓
SAL_CA6439AA	48206	SRR1958131	2014	9	4	Europe	United Kingdom	Enteritidis	24824	1.2.3.38.38.38.550	✓	✓
SAL_CA6369AA	50158	SRR1958208	2014	9	20	Europe	United Kingdom	Enteritidis	2066	1.2.3.38.38.38.413	✓	✓
SAL_CA6358AA	57641	SRR1958219	2014	9	26	Europe	United Kingdom	Enteritidis	7317	1.2.3.38.38.38.655	✓	✓
SAL_CA6305AA	62610	SRR1958272	2014	10	30	Europe	United Kingdom	Enteritidis	24946	1.2.3.38.38.38.653	✓	✓
SAL_CA6297AA	66845	SRR1958280	2014	10	13	Europe	United Kingdom	Enteritidis	7317	1.2.3.38.38.38.544	✓	✓
SAL_CA6117AA	62554	SRR1958460	2014	8	24	Europe	United Kingdom	Enteritidis	25112	1.2.3.38.38.38.550	✓	✓
SAL_CA6073AA	60382	SRR1958504	2014	10	7	Europe	United Kingdom	Enteritidis	7317	1.2.3.38.38.38.2034	✓	✓
SAL_CA6031AA	46156	SRR1958546	2014	8	19	Europe	United Kingdom	Enteritidis	25187	1.2.3.38.38.38.550	✓	✓
SAL_CA5963AA	62556	SRR1958614	2014	8	29	Europe	United Kingdom	Enteritidis	25250	1.2.3.38.38.38.1151	✓	✓
SAL_CA5901AA	60074	SRR1958676	2014	10	9	Europe	United Kingdom	Enteritidis	7317	1.2.3.38.38.38.655	✓	✓
SAL_CA5598AA	69743	SRR1960032	2014	11	13	Europe	United Kingdom	Enteritidis	25562	1.2.3.38.38.38.484	✓	✓
SAL_CA5222AA	74309	SRR1963071	2014	11	27	Europe	United Kingdom	Enteritidis	7317	1.2.3.38.38.38.458	✓	✓
SAL_CA5115AA	69699	SRR1963272	2014	11	12	Europe	United Kingdom	Enteritidis	24946	1.2.3.38.38.38.653	✓	✓
SAL_CA4837AA	34180	SRR1965068	2014	7	17	Europe	United Kingdom	Enteritidis	26182	1.2.3.38.38.38.624	✓	✓
SAL_CA4792AA	57102	SRR1965114	2014	8	11	Europe	United Kingdom	Enteritidis	26222	1.2.3.38.38.38.367	✓	✓
SAL_CA4785AA	32476	SRR1965122	2014	7	17	Europe	United Kingdom	Enteritidis	26229	1.2.3.38.38.38.393	✓	✓
SAL_CA4697AA	50356	SRR1965213	2014	9	5	Europe	United Kingdom	Enteritidis	26308	1.2.3.38.38.38.624	✓	✓
SAL_CA4642AA	53110	SRR1965275	2014	9	23	Europe	United Kingdom	Enteritidis	26355	1.2.3.38.38.38.354	✓	✓
SAL_CA4605AA	21785	SRR1965313	2014	6	3	Europe	United Kingdom	Enteritidis	26390	1.2.3.38.38.38.38	✓	✓
SAL_CA4591AA	52954	SRR1965327	2014	9	25	Europe	United Kingdom	Enteritidis	26402	1.2.3.38.38.38.655	✓	✓
SAL_CA4580AA	37759	SRR1965339	2014	7	28	Europe	United Kingdom	Enteritidis	514	1.2.3.38.38.38.570	✓	✓
SAL_CA4509AA	65448	SRR1965411	2014	10	3	Europe	United Kingdom	Enteritidis	26469	1.2.3.38.38.38.2246	✓	✓
SAL_CA4476AA	40327	SRR1965444	2014	8	18	Europe	United Kingdom	Enteritidis	26498	1.2.3.38.38.38.550	✓	✓
SAL_CA4438AA	24053	SRR1965483	2014	6	19	Europe	United Kingdom	Enteritidis	26531	1.2.3.38.38.38.1166	✓	✓
SAL_CA4437AA	43431	SRR1965484	2014	8	29	Europe	United Kingdom	Enteritidis	26532	1.2.3.38.38.38.458	✓	✓
SAL_CA4427AA	38309	SRR1965496	2014	7	28	Europe	United Kingdom	Enteritidis	26542	1.2.3.38.38.38.413	✓	✓
SAL_CA4394AA	37778	SRR1965530	2014	8	1	Europe	United Kingdom	Enteritidis	7612	1.2.3.38.38.38.367	✓	✓
SAL_CA4361AA	31977	SRR1965563	2014	4	22	Europe	United Kingdom	Enteritidis	514	1.2.3.38.38.38.790	✓	✓
SAL_CA4317AA	36473	SRR1965618	2014	7	21	Europe	United Kingdom	Enteritidis	26634	1.2.3.38.38.38.746	✓	✓
SAL_CA4268AA	36506	SRR1965669	2014	7	24	Europe	United Kingdom	Enteritidis	26678	1.2.3.38.38.38.461	✓	✓
SAL_CA4213AA	37062	SRR1965730	2014	8	6	Europe	United Kingdom	Enteritidis	26723	1.2.3.38.38.38.1201	✓	✓
SAL_CA4209AA	65449	SRR1965735	2014	8	1	Europe	United Kingdom	Enteritidis	26727	1.2.3.38.38.38.413	✓	✓
SAL_CA4203AA	40269	SRR1965742	2014	8	9	Europe	United Kingdom	Enteritidis	26733	1.2.3.38.38.38.1162	✓	✓
SAL_CA4194AA	46009	SRR1965753	2014	8	9	Europe	United Kingdom	Enteritidis	25112	1.2.3.38.38.38.550	✓	✓
SAL_CA4174AA	65379	SRR1965774	2014	9	29	Europe	United Kingdom	Enteritidis	26759	1.2.3.38.38.38.728	✓	✓
SAL_CA4165AA	57112	SRR1965784	2014	8	1	Europe	United Kingdom	Enteritidis	26768	1.2.3.38.38.38.413	✓	✓
SAL_CA4088AA	31952	SRR1965867	2014	7	24	Europe	United Kingdom	Enteritidis	512	1.2.3.38.38.38.550	✓	✓
SAL_CA4077AA	91893	SRR1965879	2014	8	1	Europe	United Kingdom	Enteritidis	26844	1.2.3.38.38.38.413	✓	✓
SAL_CA4061AA	31969	SRR1965895	2014	7	25	Europe	United Kingdom	Enteritidis	26858	1.2.3.38.38.38.624	✓	✓
SAL_CA3956AA	23500	SRR1966002	2014	6	17	Europe	United Kingdom	Enteritidis	26951	1.2.3.38.38.38.949	✓	✓

SAL_CA3943AA	39486	SRR1966020	2014	8	4	Europe	United Kingdom	Enteritidis	26963	1.2.3.38.38.38.1184	✓	✓
SAL_CA3934AA	26644	SRR1966029	2014	6	30	Europe	United Kingdom	Enteritidis	26971	1.2.3.38.38.38.472	✓	✓
SAL_CA3888AA	37835	SRR1966076	2014	7	28	Europe	United Kingdom	Enteritidis	27010	1.2.3.38.38.38.1127	✓	✓
SAL_CA3887AA	36504	SRR1966077	2014	7	23	Europe	United Kingdom	Enteritidis	27011	1.2.3.38.38.38.544	✓	✓
SAL_CA3862AA	25260	SRR1966102	2014	7	7	Europe	United Kingdom	Enteritidis	27034	1.2.3.38.38.38.550	✓	✓
SAL_CA3715AA	34187	SRR1966252	2014	7	25	Europe	United Kingdom	Enteritidis	27163	1.2.3.38.38.38.367	✓	✓
SAL_CA3678AA	32477	SRR1966289	2014	7	17	Europe	United Kingdom	Enteritidis	27198	1.2.3.38.38.38.393	✓	✓
SAL_CA3660AA	40794	SRR1966307	2014	8	12	Europe	United Kingdom	Enteritidis	27214	1.2.3.38.38.38.478	✓	✓
SAL_CA3622AA	57113	SRR1966345	2014	8	5	Europe	United Kingdom	Enteritidis	27248	1.2.3.38.38.38.413	✓	✓
SAL_CA3616AA	5355	SRR1966351	2014	4	2	Europe	United Kingdom	Enteritidis	7317	1.3.154.272.272.272.272	✓	✓
SAL_CA3519AA	39449	SRR1966447	2014	7	21	Europe	United Kingdom	Enteritidis	27345	1.2.3.38.38.38.367	✓	✓
SAL_CA3484AA	21781	SRR1966482	2014	6	2	Europe	United Kingdom	Enteritidis	27375	1.2.3.38.38.38.729	✓	✓
SAL_CA3475AA	37822	SRR1966491	2014	7	30	Europe	United Kingdom	Enteritidis	27384	1.2.3.38.38.38.393	✓	✓
SAL_CA3464AA	36392	SRR1966502	2014	8	4	Europe	United Kingdom	Enteritidis	27394	1.2.3.38.38.38.488	✓	✓
SAL_CA3431AA	68972	SRR1966536	2014	11	11	Europe	United Kingdom	Enteritidis	7317	1.2.3.38.38.38.2646	✓	✓
SAL_CA3345AA	56112	SRR1966625	2014	9	12	Europe	United Kingdom	Enteritidis	27492	1.2.3.38.38.38.354	✓	✓
SAL_CA3342AA	73134	SRR1966628	2014	12	1	Europe	United Kingdom	Enteritidis	27495	1.2.3.38.38.38.2129	✓	✓
SAL_CA3286AA	32470	SRR1966685	2014	7	17	Europe	United Kingdom	Enteritidis	27546	1.2.3.38.38.38.367	✓	✓
SAL_CA3256AA	38772	SRR1966715	2014	8	6	Europe	United Kingdom	Enteritidis	27571	1.2.3.38.38.38.413	✓	✓
SAL_CA3226AA	37111	SRR1966745	2014	8	6	Europe	United Kingdom	Enteritidis	27599	1.2.3.38.38.38.1431	✓	✓
SAL_CA3113AA	21786	SRR1966858	2014	6	3	Europe	United Kingdom	Enteritidis	674	1.2.3.38.38.38.38	✓	✓
SAL_CA3060AA	73221	SRR1966911	2014	12	8	Europe	United Kingdom	Enteritidis	27738	1.2.3.38.38.38.970	✓	✓
SAL_CA3043AA	34171	SRR1966928	2014	7	24	Europe	United Kingdom	Enteritidis	27755	1.2.3.38.38.38.2245	✓	✓
SAL_CA3036AA	14128	SRR1966935	2014	5	22	Europe	United Kingdom	Enteritidis	27761	1.2.3.38.38.38.949	✓	✓
SAL_CA2987AA	27067	SRR1966984	2014	7	7	Europe	United Kingdom	Enteritidis	674	1.2.3.38.38.38.38	✓	✓
SAL_CA2933AA	37814	SRR1967038	2014	7	30	Europe	United Kingdom	Enteritidis	27851	1.2.3.38.38.38.367	✓	✓
SAL_CA2925AA	46582	SRR1967046	2014	7	16	Europe	United Kingdom	Enteritidis	27858	1.2.3.38.38.38.393	✓	✓
SAL_CA2918AA	34198	SRR1967053	2014	7	24	Europe	United Kingdom	Enteritidis	27863	1.2.3.38.38.38.1153	✓	✓
SAL_CA2895AA	52972	SRR1967076	2014	9	26	Europe	United Kingdom	Enteritidis	2066	1.2.3.38.38.38.413	✓	✓
SAL_CA2850AA	65390	SRR1967121	2014	9	12	Europe	United Kingdom	Enteritidis	24946	1.2.3.38.38.38.653	✓	✓
SAL_CA2840AA	31611	SRR1967131	2014	7	14	Europe	United Kingdom	Enteritidis	27925	1.2.3.38.38.38.394	✓	✓
SAL_CA2802AA	61859	SRR1967169	2014	10	10	Europe	United Kingdom	Enteritidis	27958	1.2.3.38.38.38.571	✓	✓
SAL_CA2784AA	34202	SRR1967187	2014	7	21	Europe	United Kingdom	Enteritidis	27975	1.2.3.38.38.38.393	✓	✓
SAL_CA2755AA	32525	SRR1967216	2014	7	18	Europe	United Kingdom	Enteritidis	28000	1.2.3.38.38.38.367	✓	✓
SAL_CA2710AA	38411	SRR1967261	2014	7	30	Europe	United Kingdom	Enteritidis	28037	1.2.3.38.38.38.544	✓	✓
SAL_CA2687AA	39515	SRR1967284	2014	8	11	Europe	United Kingdom	Enteritidis	28056	1.2.3.38.38.38.550	✓	✓
SAL_CA2650AA	40324	SRR1967321	2014	8	18	Europe	United Kingdom	Enteritidis	28090	1.2.3.38.38.38.550	✓	✓
SAL_CA2577AA	57103	SRR1967394	2014	7	31	Europe	United Kingdom	Enteritidis	28157	1.2.3.38.38.38.413	✓	✓
SAL_CA2549AA	14099	SRR1967422	2014	5	21	Europe	United Kingdom	Enteritidis	28182	1.2.3.38.38.38.949	✓	✓
SAL_CA2493AA	36476	SRR1967478	2014	7	21	Europe	United Kingdom	Enteritidis	7612	1.2.3.38.38.38.746	✓	✓
SAL_CA2461AA	38718	SRR1967510	2014	8	7	Europe	United Kingdom	Enteritidis	28254	1.2.3.38.38.38.1288	✓	✓
SAL_CA2438AA	32375	SRR1967533	2014	7	18	Europe	United Kingdom	Enteritidis	7153	1.2.3.38.38.38.393	✓	✓
SAL_CA2431AA	24987	SRR1967540	2014	6	28	Europe	United Kingdom	Enteritidis	28283	1.2.3.38.38.38.472	✓	✓
SAL_CA2374AA	57130	SRR1967597	2014	9	30	Europe	United Kingdom	Enteritidis	28332	1.2.3.38.38.38.653	✓	✓
SAL_CA2346AA	36385	SRR1967625	2014	8	4	Europe	United Kingdom	Enteritidis	7317	1.2.3.38.38.38.544	✓	✓
SAL_CA2242AA	32464	SRR1967729	2014	7	17	Europe	United Kingdom	Enteritidis	28445	1.2.3.38.38.38.544	✓	✓

SAL_CA2170AA	50360	SRR1967801	2014	9	3	Europe	United Kingdom	Enteritidis	28509	1.2.3.38.38.38.653	✓	✓
SAL_CA1887AA	39447	SRR1967990	2014	7	18	Europe	United Kingdom	Enteritidis	28676	1.2.3.38.38.38.570	✓	✓
SAL_CA1882AA	36394	SRR1967993	2014	8	4	Europe	United Kingdom	Enteritidis	2066	1.2.3.38.38.38.367	✓	✓
SAL_CA1871AA	37006	SRR1967999	2014	8	3	Europe	United Kingdom	Enteritidis	28684	1.2.3.38.38.38.653	✓	✓
SAL_CA1747AA	42171	SRR1968063	2014	8	26	Europe	United Kingdom	Enteritidis	28739	1.2.3.38.38.38.1544	✓	✓
SAL_CA1735AA	65373	SRR1968069	2014	10	2	Europe	United Kingdom	Enteritidis	28744	1.2.3.38.38.38.790	✓	✓
SAL_CA1318AA	25320	SRR1968299	2014	6	24	Europe	United Kingdom	Enteritidis	29050	1.2.3.38.38.38.472	✓	✓
SAL_CA1311AA	9310	SRR1968303	2014	4	23	Europe	United Kingdom	Enteritidis	29054	1.3.6.6.6.6.6	✓	✓
SAL_CA1299AA	40273	SRR1968309	2014	8	12	Europe	United Kingdom	Enteritidis	29060	1.2.3.38.38.38.1151	✓	✓
SAL_CA1278AA	57111	SRR1968319	2014	8	1	Europe	United Kingdom	Enteritidis	29070	1.2.3.38.38.38.1365	✓	✓
SAL_CA1258AA	31965	SRR1968329	2014	7	25	Europe	United Kingdom	Enteritidis	2066	1.2.3.38.38.38.367	✓	✓
SAL_CA1168AA	13389	SRR1968417	2014	5	12	Europe	United Kingdom	Enteritidis	29158	1.2.3.38.38.38.962	✓	✓
SAL_CA1162AA	34203	SRR1968423	2014	7	21	Europe	United Kingdom	Enteritidis	29164	1.2.3.38.38.38.393	✓	✓
SAL_CA1093AA	31975	SRR1968475	2014	7	25	Europe	United Kingdom	Enteritidis	29212	1.2.3.38.38.38.1903	✓	✓
SAL_CA0903AA	40224	SRR1968520	2014	8	13	Europe	United Kingdom	Enteritidis	29255	1.2.3.38.38.38.413	✓	✓
SAL_CA0844AA	36364	SRR1968553	2014	7	30	Europe	United Kingdom	Enteritidis	514	1.2.3.38.38.38.910	✓	✓
SAL_CA0741AA	65434	SRR1968603	2014	9	30	Europe	United Kingdom	Enteritidis	7317	1.2.3.38.38.38.655	✓	✓
SAL_CA0720AA	40274	SRR1968614	2014	8	11	Europe	United Kingdom	Enteritidis	29406	1.2.3.38.38.38.766	✓	✓
SAL_CA0650AA	25266	SRR1968651	2014	7	1	Europe	United Kingdom	Enteritidis	512	1.2.3.38.38.38.550	✓	✓
SAL_CA0634AA	36475	SRR1968659	2014	7	21	Europe	United Kingdom	Enteritidis	29482	1.2.3.38.38.38.746	✓	✓
SAL_CA0607AA	31656	SRR1968675	2014	7	10	Europe	United Kingdom	Enteritidis	29502	1.2.3.38.38.38.533	✓	✓
SAL_CA0461AA	46011	SRR1968755	2014	8	9	Europe	United Kingdom	Enteritidis	2066	1.2.3.38.38.38.367	✓	✓
SAL_CA0414AA	36477	SRR1968778	2014	7	21	Europe	United Kingdom	Enteritidis	2066	1.2.3.38.38.38.367	✓	✓
SAL_CA0401AA	37037	SRR1968785	2014	8		Europe	France	Enteritidis	29673	1.2.3.38.38.38.1098	✓	✓
SAL_CA0389AA	23057	SRR1968791	2014	6	7	Europe	United Kingdom	Enteritidis	29684	1.2.3.38.38.38.413	✓	✓
SAL_CA0358AA	21782	SRR1968806	2014	6	2	Europe	United Kingdom	Enteritidis	29714	1.2.3.38.38.38.38	✓	✓
SAL_CA0356AA	13835	SRR1968807	2014	5	15	Europe	United Kingdom	Enteritidis	29715	1.1.2.1074.1763.2074.3174	✓	✓
SAL_CA0161AA	37800	SRR1968900	2014	7	29	Europe	United Kingdom	Enteritidis	29862	1.2.3.38.38.38.367	✓	✓
SAL_CA0138AA	40795	SRR1968912	2014	8	15	Europe	United Kingdom	Enteritidis	25250	1.2.3.38.38.38.766	✓	✓
SAL_CA0102AA	37036	SRR1968931	2014	8		Europe	France	Enteritidis	551	1.2.3.38.38.38.600	✓	✓
SAL_CA0022AA	31623	SRR1968972	2014	7	9	Europe	United Kingdom	Enteritidis	29978	1.2.3.38.38.38.38	✓	✓
SAL_BA9958AA	27797	SRR1969004	2014	6	27	Europe	United Kingdom	Enteritidis	30026	1.2.3.38.38.38.38	✓	✓
SAL_BA9836AA	57110	SRR1969068	2014	8	1	Europe	United Kingdom	Enteritidis	30124	1.2.3.38.38.38.413	✓	✓
SAL_BA9661AA	32478	SRR1969172	2014	7	17	Europe	United Kingdom	Enteritidis	30253	1.2.3.38.38.38.393	✓	✓
SAL_BA9625AA	39514	SRR1969190	2014	8	14	Europe	United Kingdom	Enteritidis	30280	1.2.3.38.38.38.413	✓	✓
SAL_BA9621AA	65125	SRR1969192	2014	11	4	Europe	United Kingdom	Enteritidis	30284	1.2.3.38.38.38.653	✓	✓
SAL_BA9589AA	43427	SRR1969209	2014	8	28	Europe	United Kingdom	Enteritidis	30311	1.2.3.38.38.38.461	✓	✓
SAL_BA9550AA	32550	SRR1969229	2014	7	18	Europe	United Kingdom	Enteritidis	30344	1.2.3.38.38.38.393	✓	✓
SAL_BA9457AA	41995	SRR1969283	2014	8	20	Europe	United Kingdom	Enteritidis	30417	1.2.3.38.38.38.413	✓	✓
SAL_BA9372AA	46593	SRR1969365	2014	7	15	Europe	United Kingdom	Enteritidis	30489	1.2.3.38.38.38.1608	✓	✓
SAL_BA9351AA	37038	SRR1969386	2014	8		Europe	France	Enteritidis	30506	1.2.3.38.38.38.2314	✓	✓
SAL_BA9138AA	39506	SRR1969515	2014	8	8	Europe	United Kingdom	Enteritidis	30689	1.2.3.38.38.38.367	✓	✓
SAL_BA9133AA	23082	SRR1969517	2014	6	8	Europe	United Kingdom	Enteritidis	30694	1.2.3.38.38.38.1315	✓	✓
SAL_BA8887AA	38428	SRR1969578	2014	7	29	Europe	United Kingdom	Enteritidis	30879	1.2.3.38.38.38.550	✓	✓
SAL_BA8669AA	36528	SRR1969692	2014	7	28	Europe	United Kingdom	Enteritidis	31057	1.2.3.38.38.38.436	✓	✓
SAL_BA8636AA	32475	SRR1969708	2014	7	17	Europe	United Kingdom	Enteritidis	31088	1.2.3.38.38.38.2060	✓	✓

SAL_BA8627AA	37035	SRR1969713	2014	8		Europe	France	Enteritidis	31096	1.2.3.38.38.38.673	✓	✓
SAL_BA8576AA	31612	SRR1969738	2014	7	14	Europe	United Kingdom	Enteritidis	31141	1.2.3.38.38.38.394	✓	✓
SAL_BA8545AA	31613	SRR1969753	2014	7	14	Europe	United Kingdom	Enteritidis	31165	1.2.3.38.38.38.38	✓	✓
SAL_BA8376AA	36474	SRR1969839	2014	7	21	Europe	United Kingdom	Enteritidis	2066	1.2.3.38.38.38.367	✓	✓
SAL_BA8364AA	37007	SRR1969845	2014	8	1	Europe	United Kingdom	Enteritidis	31298	1.2.3.38.38.38.393	✓	✓
SAL_BA8273AA	38430	SRR1969891	2014	8	1	Europe	Jersey	Enteritidis	31366	1.2.3.38.38.38.367	✓	✓
SAL_BA8265AA	25225	SRR1969895	2014	7	28	Europe	United Kingdom	Enteritidis	31374	1.2.3.38.38.38.472	✓	✓
SAL_BA8197AA	41991	SRR1969928	2014	8	19	Europe	United Kingdom	Enteritidis	31431	1.2.3.38.38.38.550	✓	✓
SAL_BA8189AA	23507	SRR1969932	2014	6	19	Europe	United Kingdom	Enteritidis	31439	1.2.3.38.38.38.962	✓	✓
SAL_BA8017AA	38773	SRR1970014	2014	8	7	Europe	United Kingdom	Enteritidis	31511	1.2.3.38.38.38.413	✓	✓
SAL_BA7794AA	37806	SRR1970129	2014	7	30	Europe	United Kingdom	Enteritidis	31654	1.2.3.38.38.38.1162	✓	✓
SAL_BA7770AA	39446	SRR1970141	2014	7	12	Europe	United Kingdom	Enteritidis	31668	1.2.3.38.38.38.393	✓	✓
SAL_BA7725AA	31981	SRR1970163	2014	7	25	Europe	United Kingdom	Enteritidis	2066	1.2.3.38.38.38.367	✓	✓
SAL_EA8050AA	21845	SRR3048579	2014	6	10	Europe	United Kingdom	Enteritidis	6817	1.2.3.38.38.38.38	✓	✓
SAL_EA7904AA	43909	SRR3048844	2014	8	21	Europe	United Kingdom	Enteritidis	6957	1.2.3.38.38.38.413	✓	✓
SAL_EA7808AA	46125	SRR3048951	2014	8	23	Europe	United Kingdom	Enteritidis	7049	1.2.3.38.38.38.550	✓	✓
SAL_EA7763AA	21132	SRR3048999	2014	5	31	Europe	United Kingdom	Enteritidis	7091	1.2.3.38.38.38.2669	✓	✓
SAL_EA7752AA	58576	SRR3049014	2014	9	28	Europe	United Kingdom	Enteritidis	7102	1.2.3.38.38.38.1418	✓	✓
SAL_EA7702AA	53036	SRR3049067	2014	9	23	Europe	United Kingdom	Enteritidis	674	1.2.3.38.38.38.38	✓	✓
SAL_EA7701AA	56991	SRR3049068	2014	9	30	Europe	United Kingdom	Enteritidis	7151	1.2.3.38.38.38.2244	✓	✓
SAL_EA7677AA	21119	SRR3049092	2014	6	4	Europe	United Kingdom	Enteritidis	7175	1.2.3.38.38.38.2483	✓	✓
SAL_EA7644AA	43884	SRR3049125	2014	8	13	Europe	United Kingdom	Enteritidis	7205	1.2.3.38.38.38.2366	✓	✓
SAL_EA7635AA	43930	SRR3049134	2014	8	14	Europe	United Kingdom	Enteritidis	7214	1.2.3.38.38.38.1795	✓	✓
SAL_EA7599AA	48161	SRR3049170	2014	8	24	Europe	United Kingdom	Enteritidis	7250	1.2.3.38.38.38.550	✓	✓
SAL_EA7582AA	43936	SRR3049187	2014	8	21	Europe	United Kingdom	Enteritidis	7267	1.2.3.38.38.38.1151	✓	✓
SAL_EA7578AA	21846	SRR3049191	2014	6	9	Europe	United Kingdom	Enteritidis	7271	1.2.3.38.38.38.38	✓	✓
SAL_EA7449AA	46155	SRR3049322	2014	8	27	Europe	United Kingdom	Enteritidis	7399	1.2.3.38.38.38.461	✓	✓
SAL_EA7409AA	45269	SRR3049365	2014	8	27	Europe	United Kingdom	Enteritidis	7434	1.2.3.38.38.38.550	✓	✓
SAL_EA7284AA	43925	SRR3049494	2014	8	13	Europe	United Kingdom	Enteritidis	7549	1.2.3.38.38.38.1351	✓	✓
SAL_EA7278AA	20929	SRR3049502	2014	6	3	Europe	United Kingdom	Enteritidis	7554	1.2.3.38.38.38.550	✓	✓
SAL_EA7267AA	43958	SRR3049513	2014	8	18	Europe	United Kingdom	Enteritidis	7565	1.2.3.38.38.38.550	✓	✓
SAL_EA7231AA	39517	SRR3049549	2014	8	12	Europe	United Kingdom	Enteritidis	7599	1.2.3.38.38.38.550	✓	✓
SAL_EA7216AA	29971	SRR3049565	2014	7	21	Europe	United Kingdom	Enteritidis	7612	1.2.3.38.38.38.367	✓	✓
SAL_EA7084AA	43960	SRR3049722	2014	8	18	Europe	United Kingdom	Enteritidis	7738	1.2.3.38.38.38.550	✓	✓
SAL_EA7071AA	18556	SRR3049743	2014	6	6	Europe	United Kingdom	Enteritidis	7750	1.2.3.38.38.38.38	✓	✓
SAL_EA7047AA	29308	SRR3049775	2014	7	15	Europe	United Kingdom	Enteritidis	7773	1.2.3.38.38.38.413	✓	✓
SAL_EA7041AA	25004	SRR3049783	2014	7	3	Europe	United Kingdom	Enteritidis	7779	1.2.3.38.38.38.550	✓	✓
SAL_EA7034AA	1514	SRR3049796	2014	3	25	Europe	United Kingdom	Enteritidis	7786	1.27.153.271.271.271.271	✓	✓
SAL_EA7020AA	43937	SRR3049811	2014	8	21	Europe	United Kingdom	Enteritidis	7799	1.2.3.38.38.38.550	✓	✓
SAL_EA6983AA	21120	SRR3049851	2014	6	2	Europe	United Kingdom	Enteritidis	7833	1.2.3.38.38.38.949	✓	✓
SAL_EA6940AA	21871	SRR3049904	2014	6	12	Europe	United Kingdom	Enteritidis	7873	1.2.3.38.38.38.807	✓	✓
SAL_EA9321AA	43927	SRR3120707	2014	8	17	Europe	United Kingdom	Enteritidis	5788	1.2.3.38.38.38.515	✓	✓
SAL_FA4108AA	21807	SRR3323014	2014	6	8	Europe	United Kingdom	Enteritidis	674	1.2.3.38.38.38.38	✓	✓
SAL_FA5451AA	21809	SRR3401403	2014	6	8	Europe	United Kingdom	Enteritidis	674	1.2.3.38.38.38.38	✓	✓
SAL_FA5597AA	05224	SRR3410208	2014			Europe	Germany	Enteritidis	545	1.2.3.38.38.38.686	✓	✓
SAL_FA5596AA	05225	SRR3410209	2014			Europe	Germany	Enteritidis	546	1.2.3.38.38.38.687	✓	✓

SAL_FA5595AA	05226	SRR3410210	2014			Europe	Germany	Enteritidis	547	1.2.3.38.38.38.2115	✓	✓
SAL_FA5594AA	05227	SRR3410211	2014			Europe	Germany	Enteritidis	548	1.2.3.38.38.38.685	✓	✓
SAL_FA5593AA	05795	SRR3410212	2014			Europe	Germany	Enteritidis	549	1.2.3.38.38.38.2801	✓	✓
SAL_FA5592AA	05946	SRR3410213	2014			Europe	Germany	Enteritidis	550	1.2.3.38.38.38.2799	✓	✓
SAL_FA5591AA	06012	SRR3410214	2014			Europe	Germany	Enteritidis	551	1.2.3.38.38.38.2798	✓	✓
SAL_FA5590AA	06175	SRR3410215	2014			Europe	Germany	Enteritidis	551	1.2.3.38.38.38.2800	✓	✓
SAL_FA5589AA	06388	SRR3410216	2014			Europe	Germany	Enteritidis	552	1.2.3.38.38.38.2797	✓	✓
SAL_FA5634AA	37045	SRR3417493	2014			Europe	Austria	Enteritidis	511	1.2.3.38.38.38.1757	✓	✓
SAL_FA5633AA	37046	SRR3417494	2014			Europe	Austria	Enteritidis	512	1.2.3.38.38.38.357	✓	✓
SAL_FA5632AA	37047	SRR3417495	2014			Europe	Austria	Enteritidis	513	1.2.3.38.38.38.413	✓	✓
SAL_FA5631AA	45605	SRR3417496	2014			Europe	Luxembourg	Enteritidis	514	1.2.3.38.38.38.790	✓	✓
SAL_FA5630AA	49691	SRR3417497	2014			Europe	Austria	Enteritidis	515	1.2.3.38.38.38.1111	✓	✓
SAL_FA5629AA	49692	SRR3417498	2014			Europe	Austria	Enteritidis	516	1.2.3.38.38.38.357	✓	✓
SAL_FA5628AA	49693	SRR3417499	2014			Europe	Austria	Enteritidis	517	1.2.3.38.38.38.357	✓	✓
SAL_FA5627AA	50104	SRR3417500	2014			Europe	Austria	Enteritidis	518	1.2.3.38.38.38.790	✓	✓
SAL_FA5626AA	53973	SRR3417501	2014			Europe	Germany	Enteritidis	519	1.2.3.38.38.38.1394	✓	✓
SAL_FA5625AA	53996	SRR3417502	2014			Europe	Germany	Enteritidis	520	1.2.3.38.38.38.1255	✓	✓
SAL_FA5624AA	54008	SRR3417503	2014			Europe	Germany	Enteritidis	521	1.2.3.38.38.38.533	✓	✓
SAL_FA5623AA	54014	SRR3417504	2014			Europe	Germany	Enteritidis	522	1.2.3.38.38.38.1555	✓	✓
SAL_FA5622AA	54038	SRR3417505	2014			Europe	Germany	Enteritidis	523	1.2.3.38.38.38.1555	✓	✓
SAL_FA5621AA	54047	SRR3417506	2014			Europe	Germany	Enteritidis	524	1.2.3.38.38.38.1438	✓	✓
SAL_FA5620AA	54053	SRR3417507	2014			Europe	Germany	Enteritidis	525	1.2.3.38.38.38.1255	✓	✓
SAL_FA5619AA	54059	SRR3417508	2014			Europe	Germany	Enteritidis	526	1.2.3.38.38.38.2623	✓	✓
SAL_EA8419AA	201405757	traces-0foIqty	2014	7	2	Europe	France	Enteritidis	555		✓	✓
SAL_EA8417AA	201405861	traces-0hvfoaj	2014	7	18	Europe	France	Enteritidis	556		✓	✓
SAL_EA8112AA	201405760	traces-0JiMSsi	2014	7	23	Europe	France	Enteritidis	512		✓	✓
SAL_EA8113AA	201405756	traces-0qWOdHp	2014	7	21	Europe	France	Enteritidis	554		✓	✓
SAL_EA8416AA	201405122	traces-0wRGnlX	2014	7	19	Europe	France	Enteritidis	553		✓	✓
SAL_DA1790AA	H124680351	SRR1635097	2012	11	7	Europe	United Kingdom	Enteritidis	20195	1.1.185.343.343.343.343	✓	
SAL_DA1773AA	H123940649	SRR1635114	2012	9	21	Europe	United Kingdom	Enteritidis	20211	1.6.11.13.333.333.333	✓	
SAL_DA1151AA	H121440322	SRR1645334	2012	3	26	Europe	United Kingdom	Enteritidis	20755	13.29.164.288.288.288.288	✓	
SAL_DA1070AA	H123740519	SRR1645445	2012	9	3	Europe	United Kingdom	Enteritidis	20833	1.1.176.315.315.315.812	✓	
SAL_DA0883AA	H123580521	SRR1645782	2012	8	22	Europe	United Kingdom	Enteritidis	21002	1.1.177.321.321.321.321	✓	
SAL_DA0881AA	H123480474	SRR1645784	2012	8	21	Europe	United Kingdom	Enteritidis	21004	3.4.49.318.318.318.318	✓	
SAL_DA0796AA	H122540669	SRR1645877	2012	6	16	Europe	United Kingdom	Enteritidis	21083	1.5.170.299.299.299.299	✓	
SAL_DA0775AA	H122280366	SRR1645898	2012	5	29	Europe	United Kingdom	Enteritidis	21103	1.5.166.294.294.294.294	✓	
SAL_DA0563AA	H122420304	SRR1646133	2012	6	7	Europe	United Kingdom	Enteritidis	21303	1.1.168.297.297.297.297	✓	
SAL_DA0480AA	H120540611	SRR1646243	2012	2	1	Europe	United Kingdom	Enteritidis	21378	1.1.162.286.286.286.286	✓	
SAL_DA0447AA	H123980391	SRR1646278	2012	9	17	Europe	United Kingdom	Enteritidis	21407	1.11.181.335.335.335.335	✓	
SAL_CA6836AA	44755	SRR1957732	2014	9	10	Europe	United Kingdom	Enteritidis	24457	10.20.81.133.133.133.133	✓	
SAL_CA6759AA	44692	SRR1957809	2014	9	8	Europe	United Kingdom	Enteritidis	15673	1.5.79.129.129.129.129	✓	
SAL_CA6715AA	49744	SRR1957853	2014	9	23	Europe	United Kingdom	Enteritidis	24569	1.21.92.155.155.155.155	✓	
SAL_CA6705AA	46132	SRR1957863	2014	8	29	Europe	United Kingdom	Enteritidis	24578	9.16.68.108.144.144.144	✓	
SAL_CA6690AA	62560	SRR1957878	2014	9	5	Europe	United Kingdom	Enteritidis	24592	3.4.8.205.205.205.205	✓	
SAL_CA6635AA	62551	SRR1957933	2014	9	3	Europe	United Kingdom	Enteritidis	24643	1.5.112.204.204.204.204	✓	
SAL_CA6449AA	66859	SRR1958121	2014	10	24	Europe	United Kingdom	Enteritidis	24816	1.24.129.235.235.235.235	✓	

SAL_CA6418AA	48243	SRR1958152	2014	9	15	Europe	United Kingdom	Enteritidis	24844	1.1.2.149.149.149.149	✓
SAL_CA6406AA	46128	SRR1958164	2014	8	29	Europe	United Kingdom	Enteritidis	24854	1.1.88.143.143.143.143	✓
SAL_CA6252AA	54060	SRR1958325	2014	9	25	Europe	United Kingdom	Enteritidis	24992	1.1.101.174.174.174.174	✓
SAL_CA5999AA	50150	SRR1958578	2014	9	24	Europe	United Kingdom	Enteritidis	25218	1.1.2.43.43.380.2290	✓
SAL_CA5913AA	46122	SRR1958664	2014	8	29	Europe	United Kingdom	Enteritidis	25292	1.1.87.142.142.142.142	✓
SAL_CA5850AA	80307	SRR1959222	2015	1	13	Europe	United Kingdom	Enteritidis	25345	1.1.202.526.680.735.915	✓
SAL_CA5846AA	70657	SRR1959226	2014	11	27	Europe	United Kingdom	Enteritidis	25347	1.25.140.251.251.251.251	✓
SAL_CA5734AA	73611	SRR1959386	2014	12	9	Europe	United Kingdom	Enteritidis	25442	1.1.151.268.268.268.268	✓
SAL_CA5678AA	71396	SRR1959443	2012	11	6	Europe	United Kingdom	Enteritidis	25491	1.26.144.258.258.258.258	✓
SAL_CA5654AA	68894	SRR1959468	2014	10	23	Europe	United Kingdom	Enteritidis	25510	1.1.136.244.244.244.244	✓
SAL_CA5566AA	71409	SRR1960073	2012	11	6	Europe	United Kingdom	Enteritidis	25587	1.7.146.262.262.262.262	✓
SAL_CA5507AA	78695	SRR1960166	2014	12	24	Europe	United Kingdom	Enteritidis	25635	1.1.222.1001.1587.1831.2556	✓
SAL_CA5446AA	73639	SRR1960236	2014	12	10	Europe	United Kingdom	Enteritidis	25687	1.21.152.270.270.270.270	✓
SAL_CA5334AA	69702	SRR1960378	2014	11	19	Europe	United Kingdom	Enteritidis	25786	1.1.138.247.247.247.247	✓
SAL_CA5315AA	80363	SRR1960665	2015	1	14	Europe	United Kingdom	Enteritidis	25798	1.5.205.604.822.905.1161	✓
SAL_CA5302AA	80341	SRR1960928	2015	1	13	Europe	United Kingdom	Enteritidis	25808	1.5.201.507.639.686.839	✓
SAL_CA5285AA	73535	SRR1961505	2014	9	19	Europe	United Kingdom	Enteritidis	50869	1.1.148.264.264.264.264	✓
SAL_CA5166AA	69142	SRR1963148	2014	11	13	Europe	United Kingdom	Enteritidis	25919	3.4.137.245.245.245.245	✓
SAL_CA5161AA	70664	SRR1963220	2014	11	27	Europe	United Kingdom	Enteritidis	25922	3.4.141.253.253.253.253	✓
SAL_CA5118AA	70666	SRR1963269	2014	11	27	Europe	United Kingdom	Enteritidis	25955	1.1.105.178.254.254.254	✓
SAL_CA4968AA	73554	SRR1963454	2014	9	22	Europe	United Kingdom	Enteritidis	26077	1.1.150.266.266.266.266	✓
SAL_CA4588AA	32527	SRR1965330	2014	7	23	Europe	United Kingdom	Enteritidis	26405	1.1.53.76.76.76.76	✓
SAL_CA4559AA	45606	SRR1965360	2014	9	9	Europe	United Kingdom	Enteritidis	26429	1.5.83.136.136.136.136	✓
SAL_CA4431AA	41987	SRR1965492	2014	8	27	Europe	United Kingdom	Enteritidis	26538	1.15.61.118.118.118.118	✓
SAL_CA4372AA	13360	SRR1965552	2014	4	15	Europe	United Kingdom	Enteritidis	26588	1.1.2.360.368.369.3150	✓
SAL_CA4299AA	99068	SRR1965636	2015	3	27	Europe	United Kingdom	Enteritidis	25745	1.1.204.580.786.862.1101	✓
SAL_CA4228AA	67566	SRR1965713	2014	11	11	Europe	United Kingdom	Enteritidis	26712	1.1.130.237.237.237.237	✓
SAL_CA4214AA	68636	SRR1965729	2014	10	13	Europe	United Kingdom	Enteritidis	7317	1.2.3.38.38.38.544	✓
SAL_CA4168AA	53007	SRR1965781	2014	10	1	Europe	United Kingdom	Enteritidis	26765	8.14.59.168.168.168.168	✓
SAL_CA4045AA	68692	SRR1965912	2014	11	12	Europe	United Kingdom	Enteritidis	26868	1.1.134.242.242.242.242	✓
SAL_CA4020AA	96239	SRR1965938	2015	3	16	Europe	United Kingdom	Enteritidis	26890	1.1.208.623.859.955.1226	✓
SAL_CA4010AA	9245	SRR1965948	2014	4	23	Europe	United Kingdom	Enteritidis	26899	1.2.3.38.38.38.1098	✓
SAL_CA3995AA	83318	SRR1965963	2015	1	21	Europe	United Kingdom	Enteritidis	26913	4.31.193.423.488.506.568	✓
SAL_CA3908AA	55582	SRR1966056	2014	9	19	Europe	United Kingdom	Enteritidis	26991	3.4.104.177.177.177.177	✓
SAL_CA3880AA	14875	SRR1966084	2014	5	20	Europe	United Kingdom	Enteritidis	27017	1.7.14.16.16.16.16	✓
SAL_CA3771AA	9286	SRR1966195	2014	5	1	Europe	United Kingdom	Enteritidis	27109	1.1.1.1.1.1.1	✓
SAL_CA3761AA	9464	SRR1966205	2014	4	29	Europe	United Kingdom	Enteritidis	27119	1.1.4.9.9.9.9	✓
SAL_CA3373AA	51262	SRR1966595	2014	9	17	Europe	United Kingdom	Enteritidis	27468	1.1.2.167.167.167.167	✓
SAL_CA3359AA	83588	SRR1966609	2015	1	27	Europe	United Kingdom	Enteritidis	27480	1.1.198.484.603.644.780	✓
SAL_CA3248AA	65019	SRR1966723	2014	11	4	Europe	United Kingdom	Enteritidis	27579	1.1.124.226.226.226.226	✓
SAL_CA3045AA	38414	SRR1966926	2014	8	6	Europe	United Kingdom	Enteritidis	27753	1.12.64.98.98.98.98	✓
SAL_CA2965AA	73223	SRR1967006	2014	12	10	Europe	United Kingdom	Enteritidis	27825	1.1.147.263.263.263.263	✓
SAL_CA2952AA	63914	SRR1967019	2014	11	5	Europe	United Kingdom	Enteritidis	27838	1.1.119.217.217.217.217	✓
SAL_CA2914AA	12185	SRR1967057	2014	5	13	Europe	United Kingdom	Enteritidis	27866	1.5.9.11.11.11.11	✓
SAL_CA2838AA	68690	SRR1967133	2014	11	12	Europe	United Kingdom	Enteritidis	27927	1.1.2.240.240.240.240	✓
SAL_CA2661AA	38415	SRR1967310	2014	8	6	Europe	United Kingdom	Enteritidis	28079	1.1.30.99.99.99.99	✓

SAL_CA2636AA	14895	SRR1967335	2014	5	21	Europe	United Kingdom	Enteritidis	28104	4.8.15.17.17.17.17	✓
SAL_CA2445AA	9182	SRR1967526	2014	5	7	Europe	United Kingdom	Enteritidis	28270	1.2.3.18.278.278.278	✓
SAL_CA2190AA	7410	SRR1967781	2014	4	29	Europe	United Kingdom	Enteritidis	28492	1.1.157.276.276.276.276	✓
SAL_CA1919AA	65407	SRR1967973	2014	9	17	Europe	United Kingdom	Enteritidis	28661	1.1.127.233.233.233.233	✓
SAL_CA1781AA	95017	SRR1968045	2015	3	11	Europe	United Kingdom	Enteritidis	28723	1.1.212.724.1061.1193.1591	✓
SAL_CA1709AA	45579	SRR1968089	2014	9	9	Europe	United Kingdom	Enteritidis	28762	1.11.82.135.135.135.135	✓
SAL_CA1644AA	9263	SRR1968125	2014	4	24	Europe	United Kingdom	Enteritidis	28825	1.5.159.280.280.280.280	✓
SAL_CA1631AA	34201	SRR1968131	2014	7	29	Europe	United Kingdom	Enteritidis	28838	3.4.8.81.81.81.81	✓
SAL_CA1499AA	38412	SRR1968202	2014	8	6	Europe	United Kingdom	Enteritidis	28959	1.1.63.97.97.97.97	✓
SAL_CA1480AA	31651	SRR1968212	2014	7	15	Europe	United Kingdom	Enteritidis	28976	1.1.34.74.74.74.74	✓
SAL_CA1304AA	27973	SRR1968306	2014	7	17	Europe	United Kingdom	Enteritidis	29057	1.1.44.59.59.59.59	✓
SAL_CA1186AA	40280	SRR1968399	2014	8	19	Europe	United Kingdom	Enteritidis	29141	1.5.69.111.111.111.111	✓
SAL_CA1179AA	39508	SRR1968406	2014	8	18	Europe	United Kingdom	Enteritidis	29147	1.11.48.106.106.106.106	✓
SAL_CA0914AA	23508	SRR1968511	2014	6	25	Europe	United Kingdom	Enteritidis	29244	1.1.38.47.47.47.47	✓
SAL_CA0512AA	37836	SRR1968725	2014	8	5	Europe	United Kingdom	Enteritidis	29580	3.13.56.93.93.93.93	✓
SAL_CA0496AA	40366	SRR1968732	2014	8	28	Europe	United Kingdom	Enteritidis	29594	1.5.72.115.115.115.115	✓
SAL_CA0475AA	34191	SRR1968743	2014	7	29	Europe	United Kingdom	Enteritidis	29609	1.12.55.79.79.79.79	✓
SAL_CA0411AA	68689	SRR1968780	2014	11	12	Europe	United Kingdom	Enteritidis	29663	1.1.131.239.239.239.239	✓
SAL_BA9681AA	83706	SRR1969162	2014	12	4	Europe	United Kingdom	Enteritidis	25680	1.5.192.373.396.403.786	✓
SAL_BA9491AA	68696	SRR1969258	2014	11	13	Europe	United Kingdom	Enteritidis	25666	1.1.135.243.243.243.243	✓
SAL_BA9456AA	63932	SRR1969284	2014	11	6	Europe	United Kingdom	Enteritidis	30418	1.1.122.221.221.221.221	✓
SAL_BA9448AA	12110	SRR1969291	2014	5	8	Europe	United Kingdom	Enteritidis	30426	1.2.3.38.38.38.949	✓
SAL_BA9347AA	13413	SRR1969390	2014	5	16	Europe	United Kingdom	Enteritidis	30510	1.2.3.151.151.151.3167	✓
SAL_BA9112AA	45610	SRR1969527	2014	9	9	Europe	United Kingdom	Enteritidis	30712	1.1.2.137.137.137.137	✓
SAL_BA8867AA	85758	SRR1969589	2015	2	9	Europe	United Kingdom	Enteritidis	27533	1.1.200.491.619.660.802	✓
SAL_BA8861AA	6446	SRR1969592	2014	4	17	Europe	United Kingdom	Enteritidis	30900	1.1.156.275.275.275.275	✓
SAL_BA8735AA	53129	SRR1969657	2014	9	29	Europe	United Kingdom	Enteritidis	31001	1.1.2.173.173.173.173	✓
SAL_BA8599AA	39476	SRR1969727	2014	8	15	Europe	United Kingdom	Enteritidis	31119	1.11.67.104.104.104.104	✓
SAL_BA8493AA	94103	SRR1969779	2015	3	5	Europe	United Kingdom	Enteritidis	31207	1.5.209.638.889.994.1289	✓
SAL_BA8372AA	53113	SRR1969841	2014	9	29	Europe	United Kingdom	Enteritidis	31296	1.1.99.172.172.172.172	✓
SAL_BA7987AA	40348	SRR1970029	2014	8	28	Europe	United Kingdom	Enteritidis	31527	3.17.71.114.114.114.114	✓
SAL_BA7790AA	32392	SRR1970131	2014	7	25	Europe	United Kingdom	Enteritidis	31656	1.1.52.75.75.75.75	✓
SAL_BA7768AA	63923	SRR1970142	2014	11	6	Europe	United Kingdom	Enteritidis	31669	8.23.121.220.220.220.220	✓
SAL_BA7739AA	95025	SRR1970156	2015	3	11	Europe	United Kingdom	Enteritidis	31680	17.38.223.1020.1637.1891.2642	✓
SAL_EA7787AA	21091	SRR3048975	2014	6	3	Europe	United Kingdom	Enteritidis	7069	1.11.31.37.37.37.37	✓
SAL_EA7715AA	99074	SRR3049054	2014	3	27	Europe	United Kingdom	Enteritidis	7139	15.32.196.474.586.621.745	✓
SAL_EA7708AA	20903	SRR3049061	2014	6	5	Europe	United Kingdom	Enteritidis	7146	1.1.26.31.31.31.31	✓
SAL_EA7529AA	18577	SRR3049240	2014	6	6	Europe	United Kingdom	Enteritidis	7319	4.9.21.24.24.24.24	✓
SAL_EA7524AA	29960	SRR3049245	2014	7	23	Europe	United Kingdom	Enteritidis	7324	1.1.191.358.363.364.366	✓
SAL_EA7420AA	53076	SRR3049354	2014	9	30	Europe	United Kingdom	Enteritidis	7424	1.1.98.171.171.171.171	✓
SAL_EA7407AA	18531	SRR3049367	2014	6	4	Europe	United Kingdom	Enteritidis	7436	1.1.229.1034.1684.1954.2823	✓
SAL_EA7045AA	21065	SRR3049778	2014	6	3	Europe	United Kingdom	Enteritidis	7775	1.10.28.33.33.33.33	✓
SAL_FA3983AA	185901	SRR3284689	2015	11	3	Europe	United Kingdom	Enteritidis	1957	1.1.242.1065.1746.2051.3585	✓
SAL_FA3963AA	166245	SRR3284721	2015	9	23	Europe	United Kingdom	Enteritidis	1976	1.2.270.1145.1898.2264.3626	✓
SAL_FA3948AA	134013	SRR3284736	2015	7	15	Europe	United Kingdom	Enteritidis	1989	1.1.245.1070.1756.2063.3141	✓
SAL_FA3913AA	197074	SRR3284773	2015	12	4	Europe	United Kingdom	Enteritidis	2021	1.1.47.214.1658.2500.4224	✓

SAL_FA3844AA	183662	SRR3285109	2015	10	27	Europe	United Kingdom	Enteritidis	2080	1.1.228.1033.1681.1946.4002	✓
SAL_FA3840AA	206728	SRR3285244	2016	1	12	Europe	United Kingdom	Enteritidis	2084	1.1.2.197.544.2403.4325	✓
SAL_FA3831AA	133214	SRR3285283	2015	7	14	Europe	United Kingdom	Enteritidis	2092	1.1.2.1066.1749.2054.3119	✓
SAL_FA3755AA	146116	SRR3285416	2015	8	12	Europe	United Kingdom	Enteritidis	2156	1.3.249.1082.1788.2113.3276	✓
SAL_FA3710AA	180813	SRR3285462	2015	9	10	Europe	United Kingdom	Enteritidis	2196	1.2.262.1137.1888.2254.3602	✓
SAL_FA3702AA	170334	SRR3285470	2015	9	17	Europe	United Kingdom	Enteritidis	2203	1.27.225.1150.1907.2277.3665	✓
SAL_FA3634AA	104253	SRR3286602	2015	4	20	Europe	United Kingdom	Enteritidis	2262	1.3.224.1021.1652.1906.2677	✓
SAL_FA3509AA	122682	SRR3286836	2015	6	12	Europe	United Kingdom	Enteritidis	2367	1.1.236.1049.1714.1998.2934	✓
SAL_FA3491AA	122699	SRR3286863	2015	6	15	Europe	United Kingdom	Enteritidis	2381	1.1.237.1051.1716.2001.2940	✓
SAL_FA3487AA	120575	SRR3286870	2015	4	30	Europe	United Kingdom	Enteritidis	2385	1.1.234.1045.1708.1982.2900	✓
SAL_FA3479AA	119502	SRR3286886	2015	6	2	Europe	United Kingdom	Enteritidis	2392	1.1.233.1044.1704.1976.2889	✓
SAL_FA7330AA	143553	SRR3585347	2015	8	10	Europe	United Kingdom	Enteritidis	40253	1.1.250.1085.1794.2122.3293	✓
SAL_FA7329AA	152413	SRR3585348	2015	8	25	Europe	United Kingdom	Enteritidis	40254	1.1.256.1101.1827.2165.3375	✓
SAL_FA7327AA	164715	SRR3585351	2015	9	21	Europe	United Kingdom	Enteritidis	40256	1.3.263.1128.1873.2229.3531	✓
SAL_FA7326AA	134034	SRR3585352	2015	7	8	Europe	United Kingdom	Enteritidis	40257	1.1.242.1065.1746.2051.3115	✓
SAL_FA7325AA	137147	SRR3585354	2015	7	23	Europe	United Kingdom	Enteritidis	40258	4.44.246.1071.1757.2064.3146	✓
SAL_FA7324AA	179844	SRR3585355	2015	10	23	Europe	United Kingdom	Enteritidis	40259	1.1.277.1177.1995.2391.3912	✓
SAL_FA7323AA	154869	SRR3585356	2015	9	1	Europe	United Kingdom	Enteritidis	40260	1.47.260.1114.1845.2190.3444	✓
SAL_FA7322AA	120578	SRR3585357	2015	6	2	Europe	United Kingdom	Enteritidis	40261	1.3.235.1046.1710.1985.2905	✓
SAL_FA7321AA	153189	SRR3585358	2015	8	27	Europe	United Kingdom	Enteritidis	40262	4.45.257.1106.1833.2173.3394	✓
SAL_FA7320AA	160846	SRR3585359	2015	9	14	Europe	United Kingdom	Enteritidis	40263	4.31.264.1129.1875.2231.3537	✓
SAL_FA7319AA	160806	SRR3585360	2015	9	10	Europe	United Kingdom	Enteritidis	40264	1.2.262.1118.1850.2199.3462	✓
SAL_FA7318AA	131456	SRR3585361	2015	7	8	Europe	United Kingdom	Enteritidis	40265	3.4.241.1063.1740.2042.3081	✓
SAL_FA7317AA	178732	SRR3585363	2015	10	13	Europe	United Kingdom	Enteritidis	40266	1.5.276.1175.1987.2378.3879	✓
SAL_FA7316AA	183605	SRR3585364	2015	11	2	Europe	United Kingdom	Enteritidis	40267	1.5.279.1188.2016.2426.4013	✓
SAL_FA7315AA	190569	SRR3585365	2015	11	24	Europe	United Kingdom	Enteritidis	40268	20.53.283.1206.2050.2481.4147	✓
SAL_FA7314AA	185894	SRR3585367	2015	11	11	Europe	United Kingdom	Enteritidis	40269	1.1.280.1189.2017.2431.4023	✓
SAL_FA7313AA	168519	SRR3585375	2015	9	25	Europe	United Kingdom	Enteritidis	40270	4.50.271.1154.1912.2284.3677	✓
SAL_FA7312AA	110976	SRR3585376	2015	5	11	Europe	United Kingdom	Enteritidis	2345	1.1.228.1033.1681.1946.2785	✓
SAL_FA7311AA	193289	SRR3585377	2015	12	2	Europe	United Kingdom	Enteritidis	40271	13.52.281.1202.2042.2470.4123	✓
