# Supplementary material for: Comparative analysis of core genome MLST and SNP typing within a European Salmonella serovar Enteritidis outbreak
Source: Int J Food Microbiol. 2018 Jun 2;274:1–11. doi: 10.1016/j.ijfoodmicro.2018.02.023 (PMC5899760; doi:10.1016/j.ijfoodmicro.2018.02.023)
Supplement: Supplementary Table 2 — Additional data for the 8965 Salmonella serovar Enteritidis isolates used to generate Fig. 4. Table including detailed metadata of all of the serovar Enteritidis isolates used to generate Fig. 4, making the dataset easily available and enabling replication of this analysis. [file mmc3.pdf]

Enterobase Barcode	Name	Accession No.	Collection Year	Collection Month	Collection Day	Continent	Country	Serovar (given)	SISTR serovar (predicted)	rMLST	cgMLST
SAL_AA7056AA	PNUSAS000496	SRR2183035	2015	4		North America	United States	Enteritidis	Enteritidis	1425	39551
SAL_AA7057AA	PNUSAS000495	SRR2183034	2015	4		North America	United States	Enteritidis	Enteritidis	1425	17527
SAL_AA7058AA	PNUSAS000494	SRR2183033	2015	3		North America	United States	Enteritidis	Enteritidis	1425	12260
SAL_AA7083AA	PNUSAS000493	SRR2182938				North America	United States	Enteritidis (Predicted)	Enteritidis	27863	39529
SAL_AA7084AA	PNUSAS000492	SRR2182937	2015	5		North America	United States	Enteritidis	Enteritidis	27863	39528
SAL_AA7085AA	PNUSAS000491	SRR2182936	2015	5		North America	United States	Enteritidis	Enteritidis	1425	39527
SAL_AA7089AA	PNUSAS000363	SRR2182932				North America	United States	Enteritidis	Enteritidis	1425	39523
SAL_AA7092AA	PNUSAS000362	SRR2182929				North America	United States	Enteritidis (Predicted)	Enteritidis	1425	39520
SAL_AA7093AA	PNUSAS000361	SRR2182925				North America	United States	Enteritidis	Enteritidis	1425	39519
SAL_AA7106AA	PNUSAS000511	SRR2182912				North America	United States	Enteritidis (Predicted)	Enteritidis	1425	39508
SAL_AA7128AA	PNUSAS000488	SRR2182889	2015	5		North America	United States	Enteritidis	Enteritidis	1425	39488
SAL_AA7129AA	PNUSAS000486	SRR2182888				North America	United States	Enteritidis (Predicted)	Enteritidis	1425	39487
SAL_AA7130AA	PNUSAS000485	SRR2182887				North America	United States	Enteritidis (Predicted)	Enteritidis	1425	39486
SAL_AA7131AA	PNUSAS000448	SRR2182886				North America	United States	Enteritidis	Enteritidis	1425	2668
SAL_AA7133AA	PNUSAS000484	SRR2182884	2015	4		North America	United States	Enteritidis	Enteritidis	1425	1808
SAL_AA7161AA	PNUSAS000416	SRR2182784				North America	United States	Enteritidis (Predicted)	Enteritidis	1425	39467
SAL_AA7162AA	PNUSAS000415	SRR2182783				North America	United States	Enteritidis (Predicted)	Enteritidis	1425	35024
SAL_AA7163AA	PNUSAS000414	SRR2182782				North America	United States	Enteritidis (Predicted)	Enteritidis	8003	39466
SAL_AA7164AA	PNUSAS000421	SRR2182781				North America	United States	Enteritidis (Predicted)	Enteritidis	1425	39465
SAL_AA7165AA	PNUSAS000420	SRR2069383				North America	United States	Enteritidis (Predicted)	Enteritidis	1425	39464
SAL_AA7166AA	PNUSAS000419	SRR2182779				North America	United States	Enteritidis (Predicted)	Enteritidis	1425	39463
SAL_AA7167AA	PNUSAS000418	SRR2182778				North America	United States	Enteritidis (Predicted)	Enteritidis	1425	39462
SAL_AA7168AA	PNUSAS000417	SRR2069380				North America	United States	Enteritidis (Predicted)	Enteritidis	1425	39461
SAL_AA7169AA	PNUSAS000410	SRR2182776				North America	United States	Enteritidis (Predicted)	Enteritidis	1425	39460
SAL_AA7170AA	NY57462994	SRR2157023	2015	7		North America	United States	Enteritidis	Enteritidis	1425	39459
SAL_AA7171AA	NY57462933	SRR2157022	2015	7		North America	United States	Enteritidis	Enteritidis	1425	39457
SAL_AA7172AA	NY57379802	SRR2157021	2015	7		North America	United States	Enteritidis	Enteritidis	1425	39458
SAL_AA7173AA	NY57379782	SRR2157020	2015	7		North America	United States	Enteritidis	Enteritidis	1425	39456
SAL_AA7174AA	NY57379764	SRR2157019	2015	7		North America	United States	Enteritidis	Enteritidis	1425	39455
SAL_AA7175AA	NY57379479	SRR2157018	2015	7		North America	United States	Enteritidis	Enteritidis	1425	39454
SAL_AA7176AA	NY57269529	SRR2157017	2015	6		North America	United States	Enteritidis	Enteritidis	1425	39453
SAL_AA7177AA	NY57269440	SRR2157016	2015	6		North America	United States	Enteritidis	Enteritidis	1425	39452
SAL_AA7178AA	NY57463012	SRR2157015	2015	7		North America	United States	Enteritidis	Enteritidis	1425	39451
SAL_AA7179AA	NY57194058	SRR2157014	2015	7		North America	United States	Enteritidis	Enteritidis	1425	39450
SAL_AA7180AA	NY57194005	SRR2157013	2015	6		North America	United States	Enteritidis	Enteritidis	1425	39449
SAL_AA7181AA	NY57269402	SRR2135520	2015	7		North America	United States	Enteritidis	Enteritidis	1425	39448
SAL_AA7182AA	NY57194181	SRR2135519	2015	7		North America	United States	Enteritidis	Enteritidis	1425	36786
SAL_AA7183AA	NY57194031	SRR2135518	2015	6		North America	United States	Enteritidis	Enteritidis	1425	39447
SAL_AA7184AA	NY57193981	SRR2135517	2015	7		North America	United States	Enteritidis	Enteritidis	1425	39446
SAL_AA7185AA	NY57193923	SRR2135516	2015	6		North America	United States	Enteritidis	Enteritidis	1425	39445
SAL_AA7186AA	NY57120837	SRR2134757	2015	6		North America	United States	Enteritidis	Enteritidis	1425	4151
SAL_AA7187AA	NY57120805	SRR2134756	2015	7		North America	United States	Enteritidis	Enteritidis	1425	39444
SAL_AA7188AA	NY57080271	SRR2134755	2015	6		North America	United States	Enteritidis	Enteritidis	1425	35685
SAL_AA7189AA	NY57079882	SRR2134754	2015	6		North America	United States	Enteritidis	Enteritidis	1425	4151
SAL_AA7190AA	NY57079867	SRR2134753	2015	6		North America	United States	Enteritidis	Enteritidis	1425	39443
SAL_AA7191AA	NY57079789	SRR2134752	2015	6		North America	United States	Enteritidis	Enteritidis	1425	35158
SAL_AA7193AA	NY57016525	SRR2134750	2015	6		North America	United States	Enteritidis	Enteritidis	1425	38200
SAL_AA7199AA	NY56962591	SRR2103102	2015	6		North America	United States	Enteritidis	Enteritidis	1425	39438
SAL_AA7200AA	NY56916987	SRR2103101	2015	6		North America	United States	Enteritidis	Enteritidis	1425	39437
SAL_AA7201AA	NY56916888	SRR2103100	2015	6		North America	United States	Enteritidis	Enteritidis	1425	39417
SAL_AA7202AA	NY56916864	SRR2103099	2015	6		North America	United States	Enteritidis	Enteritidis	1425	39436
SAL_AA7203AA	NY56916781	SRR2103098	2015	6		North America	United States	Enteritidis	Enteritidis	8003	39435
SAL_AA7204AA	NY56104917	SRR2058336	2015	4		North America	United States	Enteritidis	Enteritidis	1425	39434
SAL_AA7205AA	NY56104253	SRR2058335	2015	5		North America	United States	Enteritidis	Enteritidis	1425	2067
SAL_AA7206AA	NY56085813	SRR2058334	2015	4		North America	United States	Enteritidis	Enteritidis	1425	35024
SAL_AA7207AA	NY56085744	SRR2058333	2015	5		North America	United States	Enteritidis	Enteritidis	1425	39433
SAL_AA7208AA	NY55718321	SRR2046762	2015	4		North America	United States	Enteritidis	Enteritidis	1425	39415
SAL_AA7209AA	NY56058490	SRR2046761	2015	4		North America	United States	Enteritidis	Enteritidis	1425	39432

SAL_AA7210AA	NY56021291	SRR2046760	2015	5	North America	United States	Enteritidis	Enteritidis	27863	39431
SAL_AA7211AA	NY56021276	SRR2046759	2015	5	North America	United States	Enteritidis	Enteritidis	1425	39430
SAL_AA7212AA	NY56021260	SRR2046757	2015	5	North America	United States	Enteritidis	Enteritidis	1425	39429
SAL_AA7213AA	NY55960920	SRR2046756	2015	4	North America	United States	Enteritidis	Enteritidis	1425	39428
SAL_AA7214AA	NY55960901	SRR2046755	2015	5	North America	United States	Enteritidis	Enteritidis	1425	39427
SAL_AA7215AA	NY55959906	SRR2046754	2015	4	North America	United States	Enteritidis	Enteritidis	1425	39426
SAL_AA7216AA	NY55957804	SRR2046753	2015	4	North America	United States	Enteritidis	Enteritidis	1425	39425
SAL_AA7217AA	NY55904079	SRR2038211	2015	4	North America	United States	Enteritidis	Enteritidis	1425	34170
SAL_AA7218AA	NY55904057	SRR2038209	2015	5	North America	United States	Enteritidis	Enteritidis	1425	35024
SAL_AA7219AA	NY55904042	SRR2038208	2015	5	North America	United States	Enteritidis	Enteritidis	1425	39424
SAL_AA7220AA	NY55904150	SRR2038207	2015	4	North America	United States	Enteritidis	Enteritidis	1425	39423
SAL_AA7221AA	NY55904129	SRR2038205	2015	4	North America	United States	Enteritidis	Enteritidis	1425	39422
SAL_AA7222AA	NY55904116	SRR2038204	2015	4	North America	United States	Enteritidis	Enteritidis	1425	39421
SAL_AA7223AA	NY55905266	SRR2038203	2015	4	North America	United States	Enteritidis	Enteritidis	1425	39420
SAL_AA7224AA	NY55843228	SRR2038202	2015	4	North America	United States	Enteritidis	Enteritidis	1425	39419
SAL_AA7225AA	NY55904096	SRR2038201	2015	4	North America	United States	Enteritidis	Enteritidis	1425	39418
SAL_AA7226AA	NY55841185	SRR2038200	2015	4	North America	United States	Enteritidis	Enteritidis	1425	10271
SAL_AA7227AA	NY55841159	SRR2038199	2015	4	North America	United States	Enteritidis	Enteritidis	1425	39417
SAL_AA7228AA	NY55775996	SRR2038193	2015	4	North America	United States	Enteritidis	Enteritidis	1425	39416
SAL_AA7229AA	NY55724498	SRR2038192	2015	4	North America	United States	Enteritidis	Enteritidis	1425	39411
SAL_AA7230AA	NY55718253	SRR2038191	2015	4	North America	United States	Enteritidis	Enteritidis	1425	39415
SAL_AA7231AA	NY55726209	SRR2038190	2015	4	North America	United States	Enteritidis	Enteritidis	1425	2668
SAL_AA7232AA	NY55600339	SRR2015103	2015	4	North America	United States	Enteritidis	Enteritidis	1425	39414
SAL_AA7233AA	NY55655771	SRR2015102	2015	4	North America	United States	Enteritidis	Enteritidis	26832	39413
SAL_AA7234AA	NY55655753	SRR2015101	2015	4	North America	United States	Enteritidis	Enteritidis	1425	39412
SAL_AA7235AA	NY55655740	SRR2015100	2015	4	North America	United States	Enteritidis	Enteritidis	1425	39411
SAL_AA7236AA	NY55601119	SRR2015099	2015	4	North America	United States	Enteritidis	Enteritidis	1425	39410
SAL_AA7237AA	NY55601028	SRR2015098	2015	4	North America	United States	Enteritidis	Enteritidis	1425	39409
SAL_AA7238AA	NY55524586	SRR2009035	2015	4	North America	United States	Enteritidis	Enteritidis	27892	39408
SAL_AA7239AA	NY55524554	SRR2009034	2015	4	North America	United States	Enteritidis	Enteritidis	1425	39407
SAL_AA7240AA	NY55460189	SRR2009033	2015	4	North America	United States	Enteritidis	Enteritidis	1425	39406
SAL_AA7241AA	NY55460127	SRR2009032	2015	3	North America	United States	Enteritidis	Enteritidis	1425	39405
SAL_AA7242AA	NY55460091	SRR2009031	2015	4	North America	United States	Enteritidis	Enteritidis	1425	39404
SAL_AA7243AA	NY55460078	SRR2009020	2015	3	North America	United States	Enteritidis	Enteritidis	1425	39403
SAL_AA7244AA	NY55460066	SRR2009019	2015	4	North America	United States	Enteritidis	Enteritidis	1425	39402
SAL_AA7245AA	NY55460057	SRR2009018	2015	4	North America	United States	Enteritidis	Enteritidis	1425	39401
SAL_AA7246AA	NY55524779	SRR2009017	2015	4	North America	United States	Enteritidis	Enteritidis	1425	25754
SAL_AA7247AA	NY55460048	SRR2009016	2015	4	North America	United States	Enteritidis	Enteritidis	1425	39400
SAL_AA7248AA	NY55460039	SRR2009015	2015	4	North America	United States	Enteritidis	Enteritidis	1425	39399
SAL_AA7249AA	NY55361901	SRR2007821	2015	3	North America	United States	Enteritidis	Enteritidis	1425	39398
SAL_AA7250AA	NY55380215	SRR2007820	2015	4	North America	United States	Enteritidis	Enteritidis	1425	39397
SAL_AA7251AA	NY55380204	SRR2007819	2015	3	North America	United States	Enteritidis	Enteritidis	1425	39397
SAL_AA7252AA	NY55380194	SRR2007818	2015	3	North America	United States	Enteritidis	Enteritidis	1425	39396
SAL_AA7253AA	NY55362073	SRR2007817	2015	3	North America	United States	Enteritidis	Enteritidis	1425	2323
SAL_AA7254AA	NY55362061	SRR2007816	2015	3	North America	United States	Enteritidis	Enteritidis	1425	39395
SAL_AA7255AA	NY55362024	SRR2007815	2015	3	North America	United States	Enteritidis	Enteritidis	1425	39394
SAL_AA7256AA	NY55362012	SRR2007814	2015	3	North America	United States	Enteritidis	Enteritidis	1425	39393
SAL_AA7257AA	NY_55306147	SRR1979448	2015	3	North America	United States	Enteritidis	Enteritidis	1425	39392
SAL_AA7258AA	NY_55263483	SRR1979447	2015	2	North America	United States	Enteritidis	Enteritidis	1425	39391
SAL_AA7259AA	NY_55235726	SRR1979446	2015	3	North America	United States	Enteritidis	Enteritidis	1425	39390
SAL_AA7696AA	S038T	ERR703076					Typhimurium	Enteritidis	1424	38996
SAL_AA7724AA	S373S-sc-2004074	ERR702993					Typhimurium	Enteritidis	1424	41461
SAL_AA7750AA	S272T	ERR703055					Typhimurium	Enteritidis	1424	39115
SAL_AA7912AA	S102T	ERR703025					Typhimurium	Enteritidis	1424	38996
SAL_AA8060AA	Typhimurium	ERR702719					Typhimurium	Enteritidis	1424	41254
SAL_AA8383AA	21115_H14242077501-2	ERR653992	2014	6	Europe	United Kingdom	Enteritidis	Enteritidis	3888	38714
SAL_AA8384AA	21114_H14242077401-2	ERR653991	2014	6	Europe	United Kingdom	Enteritidis	Enteritidis	3888	38713
SAL_AA8385AA	21113_H14242077301-2	ERR653990	2014	6	Europe	United Kingdom	Enteritidis	Enteritidis	3888	38712
SAL_AA8392AA	20927_H14244069301-1	ERR653989	2014	6	Europe	United Kingdom	Enteritidis	Enteritidis	3888	38709

SAL_AA8395AA	H142580393	ERR653333	2014	6	Europe	United Kingdom	Enteritidis	Enteritidis	3888	38706
SAL_AA8396AA	908767	ERR653331	2014	6	Europe	United Kingdom	Enteritidis (Predicted)	Enteritidis	1425	38705
SAL_AA8399AA	907828	ERR653315	2014	6	Europe	United Kingdom	Enteritidis	Enteritidis	3888	38703
SAL_AA8400AA	H142580394	ERR653334	2014	6	Europe	United Kingdom	Enteritidis	Enteritidis	3888	38702
SAL_AA8401AA	908735	ERR653329	2014	6	Europe	United Kingdom	Enteritidis (Predicted)	Enteritidis	3888	38701
SAL_AA8403AA	908175	ERR653323	2014	6	Europe	United Kingdom	Enteritidis	Enteritidis	3888	38700
SAL_AA8404AA	908004	ERR653319	2014	6	Europe	United Kingdom	Enteritidis	Enteritidis	3888	38699
SAL_AA8405AA	907985	ERR653318	2014	6	Europe	United Kingdom	Enteritidis	Enteritidis	3888	38698
SAL_AA8406AA	H142480277	ERR653332	2014	6	Europe	United Kingdom	Enteritidis	Enteritidis	3888	38697
SAL_AA8407AA	908736	ERR653330	2014	6	Europe	United Kingdom	Enteritidis (Predicted)	Enteritidis	-1700	38696
SAL_AA8409AA	908430	ERR653327	2014	6	Europe	United Kingdom	Enteritidis (Predicted)	Enteritidis	3888	38695
SAL_AA8410AA	908390	ERR653325	2014	6	Europe	United Kingdom	Enteritidis	Enteritidis	3888	38694
SAL_AA8411AA	908151	ERR653322	2014	6	Europe	United Kingdom	Enteritidis	Enteritidis	3888	38693
SAL_AA8412AA	908127	ERR776485	2014	6	Europe	United Kingdom	Enteritidis (Predicted)	Enteritidis	1425	38692
SAL_AA8413AA	908108	ERR653309	2014	6	Europe	United Kingdom	Enteritidis (Predicted)	Enteritidis	1425	38691
SAL_AA8415AA	907774	ERR653305	2014	6	Europe	United Kingdom	Enteritidis (Predicted)	Enteritidis	3888	38690
SAL_AA8416AA	907757	ERR653283	2014	6	Europe	United Kingdom	Enteritidis	Enteritidis	3888	674
SAL_AA8417AA	907724	ERR653282	2014	6	Europe	United Kingdom	Enteritidis	Enteritidis	3888	38689
SAL_AA8418AA	907678	ERR653281	2014	6	Europe	United Kingdom	Enteritidis	Enteritidis	3888	38688
SAL_AA8419AA	907608	ERR653279	2014	6	Europe	United Kingdom	Enteritidis	Enteritidis	3888	7381
SAL_AA8420AA	907603	ERR653278	2014	6	Europe	United Kingdom	Enteritidis	Enteritidis	3888	38687
SAL_AA8421AA	907568	ERR653277	2014	6	Europe	United Kingdom	Enteritidis	Enteritidis	3888	674
SAL_AA8422AA	907474	ERR653273	2014	6	Europe	United Kingdom	Enteritidis	Enteritidis	3888	38686
SAL_AA8423AA	907462	ERR653290	2014	6	Europe	United Kingdom	Enteritidis	Enteritidis	3888	38685
SAL_AA8424AA	906014	ERR653287	2014	6	Europe	United Kingdom	Enteritidis (Predicted)	Enteritidis	1425	38684
SAL_AA8426AA	907659	ERR653301	2014	6	Europe	United Kingdom	Enteritidis	Enteritidis	3888	38683
SAL_AA8427AA	907812	ERR653285	2014	6	Europe	United Kingdom	Enteritidis	Enteritidis	3888	674
SAL_AA8428AA	907789	ERR653284	2014	6	Europe	United Kingdom	Enteritidis	Enteritidis	3888	38682
SAL_AA8429AA	907648	ERR653280	2014	6	Europe	United Kingdom	Enteritidis	Enteritidis	3888	674
SAL_AA8431AA	907555	ERR653276	2014	6	Europe	United Kingdom	Enteritidis	Enteritidis	3888	674
SAL_AA8432AA	907525	ERR653275	2014	6	Europe	United Kingdom	Enteritidis	Enteritidis	3888	674
SAL_AA8433AA	907513	ERR653293	2014	6	Europe	United Kingdom	Enteritidis (Predicted)	Enteritidis	3888	38679
SAL_AA8434AA	907479	ERR653274	2014	6	Europe	United Kingdom	Enteritidis	Enteritidis	3888	38680
SAL_AA8435AA	907452	ERR653272	2014	6	Europe	United Kingdom	Enteritidis	Enteritidis	3888	674
SAL_AA8436AA	907378	ERR653271	2014	6	Europe	United Kingdom	Enteritidis	Enteritidis	3888	674
SAL_AA8437AA	112797	ERR653270	2014	6	Europe	United Kingdom	Enteritidis	Enteritidis	3888	38678
SAL_AA8727AA	SE1385_06-sc-2013-07-15T1	ERR357437					Enteritidis	Enteritidis	1425	38487
SAL_AA8728AA	SE1205_88-sc-2013-07-15T1	ERR353350					Enteritidis	Enteritidis	1425	38486
SAL_AA8729AA	SE1638_90-sc-2013-07-15T1	ERR357435					Enteritidis	Enteritidis	1425	38485
SAL_AA8730AA	SEAlbis_72-sc-2013-07-15T1	ERR353348					Enteritidis	Enteritidis	1425	38484
SAL_AA8731AA	SE1224_05-sc-2013-07-15T1	ERR353347					Enteritidis	Enteritidis	1425	38483
SAL_AA8732AA	SE736_95-sc-2013-07-15T10	ERR357432					Enteritidis	Enteritidis	1425	38482
SAL_AA8733AA	SE42_13-sc-2013-07-15T10:	ERR353345					Enteritidis	Enteritidis	1425	38481
SAL_AA8734AA	SE1347_11-sc-2013-07-15T1	ERR357430					Enteritidis	Enteritidis	1425	38480
SAL_AA8810AA	3094/3	ERR352731					Typhi	Enteritidis	3776	38428
SAL_AA8812AA	3524/3	ERR352598					Typhi	Enteritidis	3776	33525
SAL_AA9076AA	2010K_0627-sc-2013-04-23T	ERR352173					Enteritidis	Enteritidis	1424	38207
SAL_AA9087AA	NY57819720	SRR2176357	2015	7	North America	United States	Enteritidis	Enteritidis	1425	38200
SAL_AA9088AA	NY57819039	SRR2176356	2015	7	North America	United States	Enteritidis	Enteritidis	1425	36786
SAL_AA9089AA	NY57819028	SRR2176282	2015	7	North America	United States	Enteritidis	Enteritidis	1425	38199
SAL_AA9090AA	NY57720350	SRR2176160	2015	7	North America	United States	Enteritidis	Enteritidis	1425	10152
SAL_AA9091AA	NY57720235	SRR2176159	2015	7	North America	United States	Enteritidis	Enteritidis	1425	38198
SAL_AA9092AA	NY57720071	SRR2176158	2015	7	North America	United States	Enteritidis	Enteritidis	1425	38197
SAL_AA9093AA	NY57594935	SRR2176157	2015	7	North America	United States	Enteritidis	Enteritidis	1425	38196
SAL_AA9094AA	NY57594647	SRR2176156	2015	7	North America	United States	Enteritidis	Enteritidis	1425	38195
SAL_AA9095AA	NY57593952	SRR2176155	2015	7	North America	United States	Enteritidis	Enteritidis	1425	38194
SAL_AA9205AA	H0170036601-sc-1875030	ERR501637					Enteritidis	Enteritidis	1424	41007
SAL_AA9206AA	D7795-sc-1875029	ERR501636					Enteritidis	Enteritidis	1424	41007
SAL_AA9209AA	H0_9180_0482-sc-1875026	ERR501633					Enteritidis	Enteritidis	1424	41007

SAL_AA9660AA	SARB18_FB	ERR424912	1988	North America	United States	Enteritidis	Enteritidis	8183	37747
SAL_AA9663AA	LH_M296	ERR424909	2001	North America	United States	Enteritidis	Enteritidis	1425	37745
SAL_AA9669AA	MZ0630	ERR424903				Enteritidis	Enteritidis	1425	34011
SAL_AA9770AA	UY0144-sc-2013-10-08T14:2	ERR405726				Enteritidis (Predicted)	Enteritidis	26042	37646
SAL_AA9774AA	UY0140-sc-2013-10-08T14:2	ERR405722				Enteritidis (Predicted)	Enteritidis	1425	37642
SAL_AA9806AA	UY050-sc-2013-10-08T14:24	ERR405536				Enteritidis (Predicted)	Enteritidis	1425	37614
SAL_AA9807AA	UY049-sc-2013-10-08T14:24	ERR405535				Enteritidis (Predicted)	Enteritidis	26042	37613
SAL_AA9808AA	UY048-sc-2013-10-08T14:24	ERR405439				Enteritidis (Predicted)	Enteritidis	26042	37612
SAL_AA9809AA	UY047-sc-2013-10-08T14:24	ERR405533				Enteritidis (Predicted)	Enteritidis	26042	37611
SAL_AA9810AA	UY046-sc-2013-10-08T14:24	ERR405532				Enteritidis (Predicted)	Enteritidis	26042	37610
SAL_AA9811AA	UY045-sc-2013-10-08T14:24	ERR405436				Enteritidis (Predicted)	Enteritidis	26042	37610
SAL_AA9812AA	UY044-sc-2013-10-08T14:24	ERR405435				Enteritidis (Predicted)	Enteritidis	26042	37609
SAL_AA9813AA	UY043-sc-2013-10-08T14:24	ERR405529				Enteritidis (Predicted)	Enteritidis	26042	37608
SAL_AA9826AA	UY041-sc-2013-10-08T14:24	ERR405527				Enteritidis (Predicted)	Enteritidis	1425	37597
SAL_AA9827AA	UY040-sc-2013-10-08T14:24	ERR405526				Enteritidis (Predicted)	Enteritidis	26042	37596
SAL_AA9828AA	UY039-sc-2013-10-08T14:24	ERR405525				Enteritidis (Predicted)	Enteritidis	26042	37595
SAL_AA9829AA	UY038-sc-2013-10-08T14:24	ERR405429				Enteritidis (Predicted)	Enteritidis	26042	37594
SAL_AA9830AA	UY037-sc-2013-10-08T14:24	ERR405523				Enteritidis (Predicted)	Enteritidis	1425	37593
SAL_AA9831AA	UY036-sc-2013-10-08T14:24	ERR405522				Enteritidis (Predicted)	Enteritidis	1425	37592
SAL_AA9832AA	UY035-sc-2013-10-08T14:24	ERR405521				Enteritidis (Predicted)	Enteritidis	1425	37591
SAL_AA9833AA	UY034-sc-2013-10-08T14:24	ERR405520				Enteritidis (Predicted)	Enteritidis	1425	37590
SAL_AA9834AA	UY033-sc-2013-10-08T14:24	ERR405424				Enteritidis (Predicted)	Enteritidis	1425	37589
SAL_AA9847AA	UY031-sc-2013-10-08T14:24	ERR405517				Enteritidis (Predicted)	Enteritidis	1425	37578
SAL_AA9848AA	UY030-sc-2013-10-08T14:24	ERR405421				Enteritidis (Predicted)	Enteritidis	26042	37577
SAL_AA9849AA	UY029-sc-2013-10-08T14:24	ERR405515				Enteritidis (Predicted)	Enteritidis	26042	37576
SAL_AA9850AA	UY028-sc-2013-10-08T14:24	ERR405419				Enteritidis (Predicted)	Enteritidis	1425	37575
SAL_AA9851AA	UY027-sc-2013-10-08T14:24	ERR405513				Enteritidis (Predicted)	Enteritidis	1425	37574
SAL_AA9852AA	UY026-sc-2013-10-08T14:24	ERR405512				Enteritidis (Predicted)	Enteritidis	26042	37573
SAL_AA9853AA	UY025-sc-2013-10-08T14:24	ERR405416				Enteritidis (Predicted)	Enteritidis	26042	37572
SAL_AA9854AA	UY024-sc-2013-10-08T14:24	ERR405510				Enteritidis (Predicted)	Enteritidis	1425	37571
SAL_AA9855AA	UY023-sc-2013-10-08T14:24	ERR405509				Enteritidis (Predicted)	Enteritidis	26042	37570
SAL_AA9856AA	UY022-sc-2013-10-08T14:24	ERR405413				Enteritidis (Predicted)	Enteritidis	1425	37569
SAL_AA9857AA	UY021-sc-2013-10-08T14:24	ERR405507				Enteritidis (Predicted)	Enteritidis	1425	37568
SAL_AA9863AA	UY0191-sc-2013-10-08T14:2	ERR405677				Enteritidis (Predicted)	Enteritidis	1425	37564
SAL_AA9869AA	UY020-sc-2013-10-08T14:24	ERR405506				Enteritidis (Predicted)	Enteritidis	1425	37560
SAL_AA9870AA	UY019-sc-2013-10-08T14:24	ERR405505				Enteritidis (Predicted)	Enteritidis	1425	37559
SAL_AA9871AA	UY018-sc-2013-10-08T14:24	ERR405504				Enteritidis (Predicted)	Enteritidis	1425	37558
SAL_AA9872AA	UY017-sc-2013-10-08T14:24	ERR405503				Enteritidis (Predicted)	Enteritidis	1425	37557
SAL_AA9873AA	UY016-sc-2013-10-08T14:24	ERR405502				Enteritidis (Predicted)	Enteritidis	1425	37556
SAL_AA9874AA	UY015-sc-2013-10-08T12:35	ERR405406				Enteritidis (Predicted)	Enteritidis	1425	37555
SAL_AA9875AA	UY014-sc-2013-10-08T12:42	ERR405500				Enteritidis (Predicted)	Enteritidis	1425	37554
SAL_AA9876AA	UY013-sc-2013-10-08T12:42	ERR405499				Enteritidis (Predicted)	Enteritidis	1425	37553
SAL_AA9877AA	UY012-sc-2013-10-08T12:36	ERR405403				Enteritidis (Predicted)	Enteritidis	1425	37552
SAL_AA9878AA	UY011-sc-2013-10-08T12:35	ERR405497				Enteritidis (Predicted)	Enteritidis	1425	37551
SAL_AA9879AA	UY010-sc-2013-10-08T12:35	ERR405496				Enteritidis (Predicted)	Enteritidis	1425	37550
SAL_AA9891AA	UY009-sc-2013-10-08T12:35	ERR405495				Enteritidis (Predicted)	Enteritidis	1425	37538
SAL_AA9892AA	UY008-sc-2013-10-08T12:35	ERR405494				Enteritidis (Predicted)	Enteritidis	1425	37538
SAL_AA9893AA	UY007-sc-2013-10-08T12:34	ERR405493				Enteritidis (Predicted)	Enteritidis	26042	37537
SAL_AA9894AA	UY006-sc-2013-10-08T12:35	ERR405492				Enteritidis (Predicted)	Enteritidis	26042	37536
SAL_AA9895AA	UY005-sc-2013-10-08T12:35	ERR405491				Enteritidis (Predicted)	Enteritidis	26042	37535
SAL_AA9896AA	UY004-sc-2013-10-08T12:35	ERR405490				Enteritidis (Predicted)	Enteritidis	26042	37532
SAL_AA9897AA	UY003-sc-2013-10-08T12:34	ERR405394				Enteritidis (Predicted)	Enteritidis	26042	37534
SAL_AA9898AA	UY002-sc-2013-10-08T12:34	ERR405488				Enteritidis (Predicted)	Enteritidis	26042	37533
SAL_AA9899AA	UY001-sc-2013-10-08T11:53	ERR405487				Enteritidis (Predicted)	Enteritidis	26042	37532
SAL_AA9902AA	UY0174-sc-2013-10-08T14:2	ERR405660				Enteritidis (Predicted)	Enteritidis	26042	37529
SAL_AA9904AA	UY0172-sc-2013-10-08T14:2	ERR405658				Enteritidis (Predicted)	Enteritidis	1425	37527
SAL_AA9955AA	UY070-sc-2013-10-08T14:25	ERR405556				Enteritidis (Predicted)	Enteritidis	1425	37477
SAL_BA0004AA	KeN136	ERR387691				Enteritidis	Enteritidis	3801	37435
SAL_BA0005AA	KeN134	ERR387690				Enteritidis	Enteritidis	1425	37434

SAL_BA0006AA	KeN133	ERR387689	Enteritidis	Enteritidis	1425	37433
SAL_BA0007AA	KeN132	ERR387688	Enteritidis	Enteritidis	1425	37432
SAL_BA0008AA	KeN130	ERR387687	Enteritidis	Enteritidis	1425	37431
SAL_BA0009AA	KeN129	ERR387686	Enteritidis	Enteritidis	1425	37430
SAL_BA0010AA	KeN128	ERR387685	Enteritidis	Enteritidis	1425	37429
SAL_BA0011AA	KeN126	ERR387684	Enteritidis	Enteritidis	1425	37428
SAL_BA0012AA	KeN125	ERR387683	Enteritidis	Enteritidis	1425	41258
SAL_BA0013AA	KeN124	ERR387682	Enteritidis	Enteritidis	8469	41257
SAL_BA0014AA	KeN123	ERR387681	Enteritidis	Enteritidis	1425	41256
SAL_BA0015AA	KeN117	ERR387680	Enteritidis	Enteritidis	1424	37427
SAL_BA0016AA	KeN116	ERR387679	Enteritidis	Enteritidis	1425	41255
SAL_BA0017AA	KeN65	ERR387678	Enteritidis	Enteritidis	1425	41501
SAL_BA0018AA	KeN62	ERR387677	Enteritidis	Enteritidis	1425	41289
SAL_BA0019AA	KeN51	ERR387676	Enteritidis	Enteritidis	1425	41492
SAL_BA0021AA	KeN31	ERR387674	Enteritidis	Enteritidis	1425	37426
SAL_BA0022AA	KeN29	ERR387673	Enteritidis	Enteritidis	1425	41287
SAL_BA0024AA	KeN27	ERR387671	Enteritidis	Enteritidis	1425	37424
SAL_BA0025AA	KeN24	ERR387670	Enteritidis	Enteritidis	1425	37423
SAL_BA0026AA	KeN23	ERR387669	Enteritidis	Enteritidis	1425	37423
SAL_BA0027AA	KeN21	ERR387668	Enteritidis	Enteritidis	1425	41286
SAL_BA0032AA	KeN9	ERR387663	Enteritidis	Enteritidis	1425	41285
SAL_BA0034AA	KeN3	ERR387661	Enteritidis	Enteritidis	1425	41284
SAL_BA0035AA	KeN1	ERR387660	Enteritidis	Enteritidis	1425	41283
SAL_BA0044AA	KeN39	ERR387651	Enteritidis	Enteritidis	1425	41282
SAL_BA0046AA	KeN143	ERR387723	Enteritidis	Enteritidis	1425	37419
SAL_BA0074AA	KeN149	ERR387695	Enteritidis	Enteritidis	1425	41281
SAL_BA0075AA	KeN142	ERR387694	Enteritidis	Enteritidis	1425	37397
SAL_BA0078AA	J55-sc-2013-05-22T09:56:54	ERR374248	Enteritidis	Enteritidis	1425	37395
SAL_BA0080AA	W24-sc-2013-05-22T09:57:3	ERR374246	Enteritidis	Enteritidis	1425	37393
SAL_BA0081AA	W4-sc-2013-05-22T09:57:33	ERR374245	Enteritidis	Enteritidis	1425	37392
SAL_BA0083AA	P177-sc-2013-05-22T09:57:0	ERR374243	Enteritidis	Enteritidis	7944	37391
SAL_BA0084AA	W19-sc-2013-05-22T09:57:3	ERR374242	Enteritidis	Enteritidis	1425	37390
SAL_BA0085AA	T129-sc-2013-05-22T09:57:2	ERR374241	Enteritidis	Enteritidis	1425	37389
SAL_BA0086AA	I15-sc-2013-05-22T09:56:47	ERR374240	Enteritidis	Enteritidis	1425	37388
SAL_BA0087AA	S56-sc-2013-05-22T09:57:14	ERR374239	Enteritidis	Enteritidis	1425	37387
SAL_BA0088AA	S85-sc-2013-05-22T09:57:17	ERR374238	Enteritidis	Enteritidis	1425	37386
SAL_BA0089AA	X25-sc-2013-05-22T09:57:25	ERR374237	Enteritidis	Enteritidis	7944	37385
SAL_BA0090AA	V91-sc-2013-05-22T09:57:29	ERR374236	Enteritidis	Enteritidis	1425	37376
SAL_BA0091AA	S4-sc-2013-05-22T09:57:12Z	ERR374235	Enteritidis	Enteritidis	1425	37384
SAL_BA0092AA	S76-sc-2013-05-22T09:57:15	ERR374234	Enteritidis	Enteritidis	1425	37383
SAL_BA0093AA	R74-sc-2013-05-22T09:57:13	ERR374233	Enteritidis	Enteritidis	1425	37382
SAL_BA0094AA	W42-sc-2013-05-22T09:57:3	ERR374232	Enteritidis	Enteritidis	1425	37381
SAL_BA0095AA	X41-sc-2013-05-22T09:57:32	ERR374231	Enteritidis	Enteritidis	1425	37380
SAL_BA0096AA	V95-sc-2013-05-22T09:57:30	ERR374230	Enteritidis	Enteritidis	1425	37379
SAL_BA0097AA	W47-sc-2013-05-22T09:57:0	ERR374229	Enteritidis	Enteritidis	1425	37378
SAL_BA0098AA	I23-sc-2013-05-22T09:56:48	ERR374228	Enteritidis	Enteritidis	1425	37377
SAL_BA0099AA	X36-sc-2013-05-22T09:57:31	ERR374227	Enteritidis	Enteritidis	1425	37376
SAL_BA0100AA	T159-sc-2013-05-22T09:57:2	ERR374226	Enteritidis	Enteritidis	1425	37375
SAL_BA0101AA	R53-sc-2013-05-22T09:57:11	ERR374225	Enteritidis	Enteritidis	1425	41280
SAL_BA0102AA	D79-sc-2013-05-22T09:56:43	ERR374224	Enteritidis	Enteritidis	1425	37362
SAL_BA0103AA	V7-sc-2013-05-22T09:57:26Z	ERR374223	Enteritidis	Enteritidis	1425	37374
SAL_BA0104AA	T103-sc-2013-05-22T09:57:2	ERR374222	Enteritidis	Enteritidis	1425	37373
SAL_BA0105AA	J39-sc-2013-05-22T09:56:52	ERR374221	Enteritidis	Enteritidis	1425	37367
SAL_BA0106AA	V55-sc-2013-05-22T09:57:28	ERR374220	Enteritidis	Enteritidis	1425	37371
SAL_BA0107AA	T145-sc-2013-05-22T09:57:2	ERR374219	Enteritidis	Enteritidis	1425	37372
SAL_BA0108AA	J73-sc-2013-05-22T09:56:55	ERR374218	Enteritidis	Enteritidis	1425	37370
SAL_BA0109AA	Q76-sc-2013-05-22T09:57:04	ERR374217	Enteritidis	Enteritidis	1425	37369
SAL_BA0110AA	V39-sc-2013-05-22T09:57:27	ERR374216	Enteritidis	Enteritidis	1425	37368
SAL_BA0111AA	J35-sc-2013-05-22T09:56:51	ERR374215	Enteritidis	Enteritidis	1425	37367

SAL_BA0112AA	I74-sc-2013-05-22T09:56:50	ERR374214	Enteritidis	Enteritidis	1425	37366
SAL_BA0113AA	P52-sc-2013-05-22T09:56:56	ERR374213	Enteritidis	Enteritidis	1425	37365
SAL_BA0114AA	S90-sc-2013-05-22T09:57:18	ERR374212	Enteritidis	Enteritidis	1425	37364
SAL_BA0115AA	T46-sc-2013-05-22T09:57:20	ERR374211	Enteritidis	Enteritidis	1425	37363
SAL_BA0116AA	I60-sc-2013-05-22T09:56:49	ERR374210	Enteritidis	Enteritidis	1425	37362
SAL_BA0117AA	P115-sc-2013-05-22T09:56:5	ERR374209	Enteritidis	Enteritidis	1425	37361
SAL_BA0118AA	P83-sc-2013-05-22T09:56:57	ERR374208	Enteritidis	Enteritidis	1425	37360
SAL_BA0119AA	Q48-sc-2013-05-22T09:57:0	ERR374207	Enteritidis	Enteritidis	1425	37358
SAL_BA0120AA	17_65-sc-2013-06-10T14:58	ERR369421	Enteritidis	Enteritidis	1425	37359
SAL_BA0122AA	4_55-sc-2013-06-10T14:58:0	ERR369419	Enteritidis	Enteritidis	1425	37356
SAL_BA0123AA	201007599-sc-2013-06-10T1	ERR369418	Enteritidis	Enteritidis	1425	37355
SAL_BA0124AA	02_8821-sc-2013-06-10T14:	ERR369417	Enteritidis	Enteritidis	1425	37354
SAL_BA0125AA	201007105-sc-2013-06-10T1	ERR369416	Enteritidis	Enteritidis	1425	37353
SAL_BA0127AA	201008619-sc-2013-06-10T1	ERR369414	Enteritidis	Enteritidis	1425	37351
SAL_BA0128AA	04_7098-sc-2013-06-10T14:	ERR369413	Enteritidis	Enteritidis	1425	37350
SAL_BA0129AA	00_5185-sc-2013-06-10T14:	ERR369412	Enteritidis	Enteritidis	1425	37349
SAL_BA0131AA	3_58-sc-2013-06-10T14:58:0	ERR369410	Enteritidis	Enteritidis	1425	37347
SAL_BA0132AA	02_3090-sc-2013-06-10T14:	ERR369409	Enteritidis	Enteritidis	1425	37346
SAL_BA0133AA	02_3077-sc-2013-06-10T14:	ERR369408	Enteritidis	Enteritidis	1425	37345
SAL_BA0134AA	30_58-sc-2013-06-10T14:58	ERR369407	Enteritidis	Enteritidis	1425	37344
SAL_BA0135AA	04_6072-sc-2013-06-10T14:	ERR369406	Enteritidis	Enteritidis	1425	37343
SAL_BA0136AA	201004918-sc-2013-06-10T1	ERR369405	Enteritidis	Enteritidis	1425	37342
SAL_BA0137AA	01_4553-sc-2013-06-10T14:	ERR369404	Enteritidis	Enteritidis	1425	37341
SAL_BA0138AA	05_6321-sc-2013-06-10T14:	ERR369403	Enteritidis	Enteritidis	1425	37340
SAL_BA0139AA	98_10272-sc-2013-06-10T14	ERR369402	Enteritidis	Enteritidis	3842	37339
SAL_BA0141AA	03_0779-sc-2013-06-10T14:	ERR369400	Enteritidis	Enteritidis	1425	37337
SAL_BA0142AA	201008533-sc-2013-06-10T1	ERR369399	Enteritidis	Enteritidis	3888	37336
SAL_BA0143AA	3_66-sc-2013-06-10T14:58:2	ERR369398	Enteritidis	Enteritidis	1425	37335
SAL_BA0145AA	08_7427-sc-2013-06-10T14:	ERR369396	Enteritidis	Enteritidis	3888	37333
SAL_BA0147AA	34_61-sc-2013-06-10T14:58	ERR369394	Enteritidis	Enteritidis	7948	37331
SAL_BA0149AA	9_63-sc-2013-06-10T14:58:1	ERR369392	Enteritidis	Enteritidis	7939	37329
SAL_BA0150AA	11_66-sc-2013-06-10T14:58	ERR369391	Enteritidis	Enteritidis	1425	37328
SAL_BA0151AA	16_65-sc-2013-06-10T14:58	ERR369390	Enteritidis	Enteritidis	1425	37327
SAL_BA0152AA	03_2506-sc-2013-06-10T14:	ERR369389	Enteritidis	Enteritidis	1425	37326
SAL_BA0153AA	94_9599-sc-2013-06-10T14:	ERR369388	Enteritidis	Enteritidis	1425	37325
SAL_BA0154AA	04_5767-sc-2013-06-10T14:	ERR369387	Enteritidis	Enteritidis	1425	37324
SAL_BA0155AA	06_0237-sc-2013-06-10T14:	ERR369386	Enteritidis	Enteritidis	1425	37323
SAL_BA0156AA	06_6191-sc-2013-06-10T14:	ERR369385	Enteritidis	Enteritidis	1425	37322
SAL_BA0157AA	201008943-sc-2013-06-10T1	ERR369384	Enteritidis	Enteritidis	1425	37321
SAL_BA0158AA	7_55-sc-2013-06-10T14:58:0	ERR369383	Enteritidis	Enteritidis	7940	37320
SAL_BA0159AA	02_3084-sc-2013-06-10T14:	ERR369382	Enteritidis	Enteritidis	1425	37319
SAL_BA0162AA	201003883-sc-2013-06-10T1	ERR369379	Enteritidis	Enteritidis	7942	37316
SAL_BA0163AA	201301913-sc-2013-06-10T1	ERR369378	Enteritidis	Enteritidis	1425	37315
SAL_BA0164AA	16_57-sc-2013-06-10T14:57	ERR369377	Enteritidis	Enteritidis	3842	37314
SAL_BA0165AA	03_0448-sc-2013-06-10T14:	ERR369376	Enteritidis	Enteritidis	1425	37313
SAL_BA0166AA	SE85_1233-sc-2013-06-10T1	ERR369375	Enteritidis	Enteritidis	1424	37312
SAL_BA0167AA	00_2993-sc-2013-06-10T14:	ERR369374	Enteritidis	Enteritidis	7944	37311
SAL_BA0169AA	09_4553-sc-2013-06-10T14:	ERR369372	Enteritidis	Enteritidis	1425	37309
SAL_BA0170AA	00_7482-sc-2013-06-10T14:	ERR369371	Enteritidis	Enteritidis	1425	37308
SAL_BA0171AA	01_4533-sc-2013-06-10T14:	ERR369370	Enteritidis	Enteritidis	1425	37307
SAL_BA0172AA	201000446-sc-2013-06-10T1	ERR369369	Enteritidis	Enteritidis	1425	37306
SAL_BA0173AA	201005749-sc-2013-06-10T1	ERR369368	Enteritidis	Enteritidis	1425	37305
SAL_BA0174AA	34_58-sc-2013-06-10T14:58	ERR369367	Enteritidis	Enteritidis	1425	37304
SAL_BA0175AA	8_55-sc-2013-06-10T14:58:0	ERR369366	Enteritidis	Enteritidis	1425	37303
SAL_BA0176AA	94_8098-sc-2013-06-10T14:	ERR369365	Enteritidis	Enteritidis	1424	37302
SAL_BA0177AA	201009544-sc-2013-06-10T1	ERR369364	Enteritidis	Enteritidis	1425	37301
SAL_BA0179AA	94_8355-sc-2013-06-10T14:	ERR369362	Enteritidis	Enteritidis	1425	37299
SAL_BA0181AA	06_2966-sc-2013-06-10T14:	ERR369360	Enteritidis	Enteritidis	1425	37297
SAL_BA0182AA	06_6063-sc-2013-06-10T14:	ERR369359	Enteritidis	Enteritidis	1425	37296

SAL_BA0185AA	201101618-sc-2013-06-10T1	ERR369356					Enteritidis	Enteritidis	3888	37293
SAL_BA0186AA	93_2136-sc-2013-06-10T14:5	ERR369355					Enteritidis	Enteritidis	1425	37292
SAL_BA0187AA	94_4688-sc-2013-06-10T14:5	ERR369354					Enteritidis	Enteritidis	1424	37291
SAL_BA0188AA	17_60-sc-2013-06-10T14:58:	ERR369353					Enteritidis	Enteritidis	1425	37290
SAL_BA0189AA	93_5337-sc-2013-06-10T14:5	ERR369352					Enteritidis	Enteritidis	1425	37289
SAL_BA0192AA	20_55-sc-2013-06-10T14:58:	ERR369349					Enteritidis	Enteritidis	3842	37286
SAL_BA0193AA	201300358-sc-2013-06-10T1	ERR369348					Enteritidis	Enteritidis	1425	37285
SAL_BA0194AA	92_3273-sc-2013-06-10T14:5	ERR369347					Enteritidis	Enteritidis	7962	37284
SAL_BA0195AA	98_2244-sc-2013-06-10T14:5	ERR369346					Enteritidis	Enteritidis	1425	37283
SAL_BA0196AA	93_7053-sc-2013-06-10T14:5	ERR369345					Enteritidis	Enteritidis	1425	37282
SAL_BA0197AA	R42-sc-2013-05-22T09:57:1	ERR369344					Enteritidis	Enteritidis	1425	37281
SAL_BA0198AA	T27-sc-2013-05-22T09:57:19	ERR369343					Enteritidis	Enteritidis	7944	37280
SAL_BA0199AA	Q64-sc-2013-05-22T09:57:0	ERR369342					Enteritidis	Enteritidis	1425	37279
SAL_BA0200AA	P149-sc-2013-05-22T09:56:5	ERR369341					Enteritidis	Enteritidis	1425	37278
SAL_BA0544AA	3395/3	ERR357546					Typhi	Enteritidis	3776	37030
SAL_BA0608AA	3154/3	ERR357616					Typhi	Enteritidis	3776	33191
SAL_BA0639AA	3239/3	ERR357503					Typhi	Enteritidis	3776	33554
SAL_BA0705AA	15MN00384	SRR2175357	2015	8	North America	United States	Enteritidis	Enteritidis	26543	36912
SAL_BA0706AA	15MN00383	SRR2175356	2015	7	North America	United States	Enteritidis	Enteritidis	1425	36911
SAL_BA0720AA	15MN00385	SRR2175342	2015	8	North America	United States	Enteritidis	Enteritidis	1425	36903
SAL_BA0728AA	15MN00394	SRR2175334	2015	7	North America	United States	Enteritidis	Enteritidis	1425	14335
SAL_BA0809AA	FDA00001998	SRR2174134	2004	1	North America	United States	Enteritidis	Enteritidis	1425	9571
SAL_BA0841AA	FDA00002000	SRR2174099	2004	1	North America	United States	Enteritidis	Enteritidis	1425	36850
SAL_BA0864AA	CFSAN035291	SRR2174074	2012		North America	United States	Enteritidis	Enteritidis	1425	36838
SAL_BA0894AA	CFSAN035302	SRR2533373	2012		North America	United States	Enteritidis	Enteritidis	1425	36824
SAL_BA0948AA	CFSAN035289	SRR2533364	2012		North America	United States	Enteritidis	Enteritidis	1425	36801
SAL_BA0957AA	FDA00001999	SRR2173976	2004	1	North America	United States	Enteritidis	Enteritidis	1425	9571
SAL_BA0969AA	NY57719970	SRR2168974	2015	7	North America	United States	Enteritidis	Enteritidis	1425	36789
SAL_BA0970AA	NY57719739	SRR2168973	2015	7	North America	United States	Enteritidis	Enteritidis	1425	36788
SAL_BA0971AA	NY57594977	SRR2168972	2015	7	North America	United States	Enteritidis	Enteritidis	1425	36787
SAL_BA0972AA	NY57290959	SRR2168971	2015	7	North America	United States	Enteritidis	Enteritidis	1425	36786
SAL_BA0973AA	NY57269583	SRR2168970	2015	7	North America	United States	Enteritidis	Enteritidis	1425	36785
SAL_BA1041AA	CFSAN035309	SRR2163268	2012		North America	United States	Enteritidis	Enteritidis	26162	36733
SAL_BA1054AA	CFSAN035315	SRR2163249	2012		North America	United States	Enteritidis	Enteritidis	1425	36723
SAL_BA1055AA	CFSAN035308	SRR2163248	2012		North America	United States	Enteritidis	Enteritidis	1425	36722
SAL_BA1061AA	NY_55224659	SRR1964511	2015	3	North America	United States	Enteritidis	Enteritidis	1425	36718
SAL_BA1062AA	NY_55224652	SRR1964510	2015	3	North America	United States	Enteritidis	Enteritidis	1425	36717
SAL_BA1063AA	NY_55119574	SRR1964503	2015	3	North America	United States	Enteritidis	Enteritidis	1425	36716
SAL_BA1064AA	NY_55066753	SRR1951991	2015	3	North America	United States	Enteritidis	Enteritidis	1425	36715
SAL_BA1065AA	NY_55066704	SRR1951990	2015	3	North America	United States	Enteritidis	Enteritidis	1425	36714
SAL_BA1066AA	NY_55041710	SRR1951989	2015	3	North America	United States	Enteritidis	Enteritidis	1425	36713
SAL_BA1067AA	NY_55041702	SRR1951987	2015	3	North America	United States	Enteritidis	Enteritidis	1425	36712
SAL_BA1068AA	NY_55041601	SRR1951986	2015	3	North America	United States	Enteritidis	Enteritidis	1425	32855
SAL_BA1069AA	NY_55038755	SRR1951985	2015	3	North America	United States	Enteritidis	Enteritidis	1425	36711
SAL_BA1070AA	NY-swgs1629	SRR1944531	2015	3	North America	United States	Enteritidis	Enteritidis	1425	10271
SAL_BA1071AA	NY-swgs1628	SRR1944530	2015	2	North America	United States	Enteritidis	Enteritidis	1425	36710
SAL_BA1072AA	NY-swgs1627	SRR1944529	2015	2	North America	United States	Enteritidis	Enteritidis	1425	36709
SAL_BA1073AA	NY-swgs1626	SRR1944528	2015	2	North America	United States	Enteritidis	Enteritidis	3888	24864
SAL_BA1074AA	NY-swgs1624	SRR1917116	2015	2	North America	United States	Enteritidis	Enteritidis	1425	36708
SAL_BA1075AA	NY-swgs1623	SRR1917115	2015	2	North America	United States	Enteritidis	Enteritidis	1425	36707
SAL_BA1076AA	NY-swgs1622	SRR1917114	2015	2	North America	United States	Enteritidis	Enteritidis	28557	36706
SAL_BA1077AA	NY-swgs1621	SRR1917113	2015	2	North America	United States	Enteritidis	Enteritidis	1425	36700
SAL_BA1078AA	NY-swgs1620	SRR1917112	2015	2	North America	United States	Enteritidis	Enteritidis	1425	36705
SAL_BA1079AA	NY-swgs1619	SRR1917111	2015	2	North America	United States	Enteritidis	Enteritidis	1425	36704
SAL_BA1080AA	NY-swgs1618	SRR1917110	2015	2	North America	United States	Enteritidis	Enteritidis	1425	36703
SAL_BA1081AA	NY-swgs1617	SRR1917109	2015	2	North America	United States	Enteritidis	Enteritidis	1425	36702
SAL_BA1082AA	NY-swgs1625	SRR1917108	2015	2	North America	United States	Enteritidis	Enteritidis	1425	1201
SAL_BA1083AA	NY-swgs1616	SRR1917106	2015	2	North America	United States	Enteritidis	Enteritidis	1425	36701
SAL_BA1084AA	NY-swgs1615	SRR1917104	2015	2	North America	United States	Enteritidis	Enteritidis	1425	36700

SAL_BA1085AA	NY-swgs1612	SRR1910382	2015	2	North America	United States	Enteritidis	Enteritidis	1425	36699
SAL_BA1086AA	NY-swgs1611	SRR1910380	2015	2	North America	United States	Enteritidis	Enteritidis	1425	36698
SAL_BA1087AA	NY-swgs1610	SRR1910379	2015	2	North America	United States	Enteritidis	Enteritidis	1425	26097
SAL_BA1088AA	NY-swgs1609	SRR1910378	2015	1	North America	United States	Enteritidis	Enteritidis	1425	36697
SAL_BA1089AA	NY-swgs1608	SRR1910376	2015	2	North America	United States	Enteritidis	Enteritidis	1425	36696
SAL_BA1090AA	NY-swgs1607	SRR1910374	2013	2	North America	United States	Enteritidis	Enteritidis	27921	36695
SAL_BA1091AA	NY-swgs1606	SRR1910372	2012	8	North America	United States	Enteritidis	Enteritidis	27921	36694
SAL_BA1092AA	NY-swgs1605	SRR1910371	2011	9	North America	United States	Enteritidis	Enteritidis	1425	36693
SAL_BA1093AA	NY-swgs1614	SRR1910370	2015	2	North America	United States	Enteritidis	Enteritidis	8003	12205
SAL_BA1094AA	NY-swgs1613	SRR1910369	2015	2	North America	United States	Enteritidis	Enteritidis	1425	36692
SAL_BA1095AA	NY-swgs1604	SRR1910368	2011	6	North America	United States	Enteritidis	Enteritidis	1425	36691
SAL_BA1096AA	NY-swgs1603	SRR1910367	2010	8	North America	United States	Enteritidis	Enteritidis	1425	36690
SAL_BA1097AA	NY-swgs1602	SRR1813479	2015	2	North America	United States	Enteritidis	Enteritidis	1425	16644
SAL_BA1098AA	NY-swgs1601	SRR1813478	2015	2	North America	United States	Enteritidis	Enteritidis	1425	36686
SAL_BA1099AA	NY-swgs1600	SRR1813477	2015	2	North America	United States	Enteritidis	Enteritidis	1425	36689
SAL_BA1100AA	NY-swgs1599	SRR1813475	2015	2	North America	United States	Enteritidis	Enteritidis	1425	36688
SAL_BA1101AA	NY-swgs1598	SRR1813474	2015	2	North America	United States	Enteritidis	Enteritidis	1425	36687
SAL_BA1102AA	NY-swgs1597	SRR1813473	2015	1	North America	United States	Enteritidis	Enteritidis	1425	36686
SAL_BA1103AA	NY-swgs1596	SRR1813472	2015	1	North America	United States	Enteritidis	Enteritidis	1425	36685
SAL_BA1104AA	NY-swgs1595	SRR1806280	2015	1	North America	United States	Enteritidis	Enteritidis	1425	36684
SAL_BA1105AA	NY-swgs1594	SRR1806279	2015	1	North America	United States	Enteritidis	Enteritidis	1425	36683
SAL_BA1106AA	NY-swgs1593	SRR1806278	2015	1	North America	United States	Enteritidis	Enteritidis	1425	36682
SAL_BA1107AA	NY-swgs1592	SRR1806277	2015	1	North America	United States	Enteritidis	Enteritidis	1425	36681
SAL_BA1108AA	NY-swgs1591	SRR1796045	2015	1	North America	United States	Enteritidis	Enteritidis	1425	36680
SAL_BA1109AA	NY-swgs1590	SRR1796044	2015	1	North America	United States	Enteritidis	Enteritidis	27924	36679
SAL_BA1110AA	NY-swgs1589	SRR1796043	2015	1	North America	United States	Enteritidis	Enteritidis	1425	36678
SAL_BA1111AA	NY-swgs1588	SRR1796042	2015	1	North America	United States	Enteritidis	Enteritidis	1425	36677
SAL_BA1112AA	NY-swgs1587	SRR1778062	2014	12	North America	United States	Enteritidis	Enteritidis	1425	36676
SAL_BA1113AA	NY-swgs1586	SRR1778060	2014	12	North America	United States	Enteritidis	Enteritidis	1425	36675
SAL_BA1114AA	NY-swgs1585	SRR1778054	2014	12	North America	United States	Enteritidis	Enteritidis	26849	36674
SAL_BA1115AA	NY-swgs1584	SRR1770487	2014	12	North America	United States	Enteritidis	Enteritidis	1425	36673
SAL_BA1116AA	NY-swgs1583	SRR1770486	2014	12	North America	United States	Enteritidis	Enteritidis	1425	21923
SAL_BA1117AA	NY-swgs1582	SRR1770485	2014	12	North America	United States	Enteritidis	Enteritidis	8003	36672
SAL_BA1118AA	NY-swgs1581	SRR1770484	2014	12	North America	United States	Enteritidis	Enteritidis	1425	36671
SAL_BA1119AA	NY-swgs1580	SRR1752283	2014	12	North America	United States	Enteritidis	Enteritidis	1425	36670
SAL_BA1120AA	NY-swgs1579	SRR1752282	2014	12	North America	United States	Enteritidis	Enteritidis	1425	36669
SAL_BA1121AA	NY-swgs1578	SRR1752281	2014	12	North America	United States	Enteritidis	Enteritidis	1425	36668
SAL_BA1122AA	NY-swgs1577	SRR1752112	2014	12	North America	United States	Enteritidis	Enteritidis	1425	36667
SAL_BA1123AA	NY-swgs1576	SRR1752111	2014	12	North America	United States	Enteritidis	Enteritidis	1425	36666
SAL_BA1124AA	NY-swgs1575	SRR1752110	2014	12	North America	United States	Enteritidis	Enteritidis	1425	36665
SAL_BA1125AA	NY-swgs1574	SRR1752109	2014	12	North America	United States	Enteritidis	Enteritidis	1425	36664
SAL_BA1126AA	NY-swgs1573	SRR1752108	2014	11	North America	United States	Enteritidis	Enteritidis	1425	36663
SAL_BA1127AA	NY-swgs1572	SRR1752107	2014	11	North America	United States	Enteritidis	Enteritidis	1425	36662
SAL_BA1128AA	NY-swgs1571	SRR1752106	2014	12	North America	United States	Enteritidis	Enteritidis	1425	36661
SAL_BA1129AA	NY-swgs1570	SRR1737751	2014	12	North America	United States	Enteritidis	Enteritidis	1425	36660
SAL_BA1130AA	NY-swgs1569	SRR1737750	2014	11	North America	United States	Enteritidis	Enteritidis	1425	36659
SAL_BA1131AA	NY-swgs1568	SRR1737749	2014	11	North America	United States	Enteritidis	Enteritidis	1425	21858
SAL_BA1132AA	NY-swgs1567	SRR1737748	2014	11	North America	United States	Enteritidis	Enteritidis	1425	36658
SAL_BA1133AA	NY-swgs1566	SRR1732683	2014	11	North America	United States	Enteritidis	Enteritidis	1425	36657
SAL_BA1134AA	NY-swgs1565	SRR1732682	2014	11	North America	United States	Enteritidis	Enteritidis	27929	36656
SAL_BA1135AA	NY-swgs1564	SRR1732681	2014	11	North America	United States	Enteritidis	Enteritidis	1425	21858
SAL_BA1136AA	NY-swgs1563	SRR1732680	2014	11	North America	United States	Enteritidis	Enteritidis	1425	36655
SAL_BA1137AA	NY-swgs1562	SRR1732679	2014	11	North America	United States	Enteritidis	Enteritidis	1425	21858
SAL_BA1138AA	NY-swgs1561	SRR1732678	2014	10	North America	United States	Enteritidis	Enteritidis	1425	36654
SAL_BA1139AA	NY-swgs1560	SRR1732677	2014	11	North America	United States	Enteritidis	Enteritidis	1425	21858
SAL_BA1140AA	NY-swgs1559	SRR1732676	2014	11	North America	United States	Enteritidis	Enteritidis	1425	36653
SAL_BA1141AA	NY-swgs1558	SRR1732675	2014	11	North America	United States	Enteritidis	Enteritidis	1425	36652
SAL_BA1147AA	FDA00002138	SRR2156630	2004	8	North America	United States	Enteritidis	Enteritidis	1425	36604
SAL_BA1157AA	FDA00002137	SRR2156618	2004	8	North America	United States	Enteritidis	Enteritidis	1425	36641

SAL_BA1162AA	FDA00002140	SRR2156609	2004	8	North America	United States	Enteritidis	Enteritidis	1425	36635
SAL_BA1165AA	FDA00002133	SRR2156604	2004	8	North America	United States	Enteritidis	Enteritidis	1425	36634
SAL_BA1168AA	CFSAN032970	SRR2156601	2012		North America	United States	Enteritidis	Enteritidis	1425	36631
SAL_BA1178AA	CFSAN034232	SRR2156578	2013		North America	United States	Enteritidis	Enteritidis	1425	36622
SAL_BA1191AA	FDA00002141	SRR2156517	2004	8	North America	United States	Enteritidis	Enteritidis	1425	36610
SAL_BA1197AA	FDA00002139	SRR2156507	2004	8	North America	United States	Enteritidis	Enteritidis	1425	36604
SAL_BA1218AA	15MN00308	SRR2153476	2015	7	North America	United States	Enteritidis	Enteritidis	27901	36585
SAL_BA1219AA	15MN00305	SRR2153475	2015	7	North America	United States	Enteritidis	Enteritidis	1425	36584
SAL_BA1220AA	15MN00306	SRR2669599	2015	7	North America	United States	Enteritidis	Enteritidis	8003	36583
SAL_BA1221AA	15MN00307	SRR2669600	2015	7	North America	United States	Enteritidis	Enteritidis	1425	36582
SAL_BA1222AA	15MN00303	SRR2153466	2015	7	North America	United States	Enteritidis	Enteritidis	26832	36581
SAL_BA1223AA	15MN00304	SRR2153465	2015	7	North America	United States	Enteritidis	Enteritidis	27904	36580
SAL_BA1224AA	15MN00302	SRR2153464	2015	7	North America	United States	Enteritidis	Enteritidis	1425	36579
SAL_BA1225AA	15MN00300	SRR2153463	2015	7	North America	United States	Enteritidis	Enteritidis	1425	36578
SAL_BA1226AA	15MN00371	SRR2153212	2015	7	North America	United States	Enteritidis	Enteritidis	1425	11501
SAL_BA1230AA	15MN00380	SRR2153208	2015	7	North America	United States	Enteritidis	Enteritidis	1425	36574
SAL_BA1231AA	15MN00373	SRR2153207	2015	7	North America	United States	Enteritidis	Enteritidis	1425	12252
SAL_BA1232AA	15MN00372	SRR2153206	2015	7	North America	United States	Enteritidis	Enteritidis	1425	8925
SAL_BA1233AA	15MN00370	SRR2153205	2015	7	North America	United States	Enteritidis	Enteritidis	1425	8925
SAL_BA1237AA	CFSAN035276	SRR2153016	2012		North America	United States	Enteritidis	Enteritidis	1425	36571
SAL_BA1247AA	CFSAN035272	SRR2153005	2013		North America	United States	Enteritidis	Enteritidis	1425	36561
SAL_BA1252AA	CFSAN035279	SRR2153000	2013		North America	United States	Enteritidis	Enteritidis	26162	36557
SAL_BA1289AA	FDA00002147	SRR2143488	2004	8	North America	United States	Enteritidis	Enteritidis	1425	36497
SAL_BA1295AA	FDA00002150	SRR2143478	2004	8	North America	United States	Enteritidis	Enteritidis	1425	36528
SAL_BA1307AA	FDA00002145	SRR2143460	2004	8	North America	United States	Enteritidis	Enteritidis	1425	36503
SAL_BA1308AA	FDA00002143	SRR2143459	2004	8	North America	United States	Enteritidis	Enteritidis	1425	36496
SAL_BA1313AA	FDA00002149	SRR2143451	2004	8	North America	United States	Enteritidis	Enteritidis	1425	36514
SAL_BA1326AA	FDA00002148	SRR2143417	2004	8	North America	United States	Enteritidis	Enteritidis	1425	36504
SAL_BA1327AA	FDA00002146	SRR2143416	2004	8	North America	United States	Enteritidis	Enteritidis	1425	36503
SAL_BA1333AA	FDA00002144	SRR2143360	2004	8	North America	United States	Enteritidis	Enteritidis	1425	36497
SAL_BA1334AA	FDA00002142	SRR2143359	2004	8	North America	United States	Enteritidis	Enteritidis	1425	36496
SAL_BA1336AA	15MN00362	SRR2140791	2015	6	North America	United States	Enteritidis	Enteritidis	1425	8925
SAL_BA1337AA	15MN00361	SRR2140790	2015	6	North America	United States	Enteritidis	Enteritidis	1425	8925
SAL_BA1338AA	15MN00346	SRR2140789	2015	7	North America	United States	Enteritidis	Enteritidis	26832	36494
SAL_BA1339AA	15MN00345	SRR2140788	2015	7	North America	United States	Enteritidis	Enteritidis	27944	36493
SAL_BA1340AA	15MN00342	SRR2140787	2015	7	North America	United States	Enteritidis	Enteritidis	1425	36492
SAL_BA1341AA	15MN00344	SRR2140786	2015	7	North America	United States	Enteritidis	Enteritidis	1425	36488
SAL_BA1344AA	15MN00343	SRR2140781	2015	7	North America	United States	Enteritidis	Enteritidis	1425	36489
SAL_BA1345AA	15MN00341	SRR2140780	2015	7	North America	United States	Enteritidis	Enteritidis	1425	36488
SAL_BA1346AA	15MN00340	SRR2140779	2015	7	North America	United States	Enteritidis	Enteritidis	1425	36487
SAL_BA1406AA	WAPHL_SAL-A00917	SRR2133398	2015	6	North America	United States	Enteritidis	Enteritidis	1425	6712
SAL_BA1409AA	15MN00320	SRR2133351	2015	7	North America	United States	Enteritidis	Enteritidis	1425	36319
SAL_BA1410AA	15MN00260	SRR2669605	2015	6	North America	United States	Enteritidis	Enteritidis	1425	36431
SAL_BA1412AA	WAPHL_SAL-A00916	SRR2133348	2015	6	North America	United States	Enteritidis	Enteritidis	1425	36438
SAL_BA1417AA	15MN00323	SRR2133340	2015	7	North America	United States	Enteritidis	Enteritidis	1425	36433
SAL_BA1418AA	WAPHL_SAL-A00911	SRR2133339	2015	6	North America	United States	Enteritidis	Enteritidis	1425	36410
SAL_BA1420AA	15MN00256	SRR2669604	2015	6	North America	United States	Enteritidis	Enteritidis	1425	36431
SAL_BA1433AA	15MN00329	SRR2133212	2015	6	North America	United States	Enteritidis	Enteritidis	26832	35856
SAL_BA1434AA	15MN00328	SRR2133211	2015	6	North America	United States	Enteritidis	Enteritidis	26832	35856
SAL_BA1435AA	WAPHL_SAL-A00912	SRR2133209	2015	6	North America	United States	Enteritidis	Enteritidis	1425	36410
SAL_BA1436AA	15MN00301	SRR2133208	2015	7	North America	United States	Enteritidis	Enteritidis	1425	36421
SAL_BA1441AA	WAPHL_SAL-A00915	SRR2133195	2015	6	North America	United States	Enteritidis	Enteritidis	1425	6712
SAL_BA1450AA	15MN00321	SRR2133171	2015	7	North America	United States	Enteritidis	Enteritidis	1425	23898
SAL_BA1451AA	WAPHL_SAL-A00914	SRR2133170	2015	6	North America	United States	Enteritidis	Enteritidis	1425	6712
SAL_BA1453AA	WAPHL_SAL-A00913	SRR2133163	2015	6	North America	United States	Enteritidis	Enteritidis	1425	36410
SAL_BA1455AA	15MN00241	SRR2669598	2015	6	North America	United States	Enteritidis	Enteritidis	1425	8925
SAL_BA1465AA	FSIS1503212	SRR2132200	2014		North America	United States	Enteritidis	Enteritidis	1425	36402
SAL_BA1495AA	FSIS1501004	SRR2131787	2015		North America	United States	Enteritidis	Enteritidis	1425	36376
SAL_BA1541AA	FSIS1501001	SRR2126759	2015		North America	United States	Enteritidis	Enteritidis	1425	36336

SAL_BA1544AA	FSIS1501000	SRR2126731	2015		North America	United States	Enteritidis	Enteritidis	1425	36333
SAL_BA1547AA	FSIS1500998	SRR2126722	2015		North America	United States	Enteritidis	Enteritidis	1425	36330
SAL_BA1553AA	FSIS1500980	SRR2126291	2015		North America	United States	Enteritidis	Enteritidis	1425	8406
SAL_BA1555AA	FSIS1500982	SRR2126289	2015		North America	United States	Enteritidis	Enteritidis	1425	36323
SAL_BA1559AA	15MN00319	SRR2125971	2015	7	North America	United States	Enteritidis	Enteritidis	1425	36319
SAL_BA1570AA	FSIS1501381	SRR2125865	2015		North America	United States	Enteritidis	Enteritidis	1425	36309
SAL_BA1580AA	FSIS1500513	SRR2125686	2014		North America	United States	Enteritidis	Enteritidis	1425	36300
SAL_BA1581AA	FSIS1500514	SRR2125685	2014		North America	United States	Enteritidis	Enteritidis	1425	36299
SAL_BA1582AA	FSIS1500516	SRR2125623	2014		North America	United States	Enteritidis	Enteritidis	1425	36298
SAL_BA1583AA	FSIS1500511	SRR2125620	2014		North America	United States	Enteritidis	Enteritidis	1425	36297
SAL_BA1584AA	FSIS1500512	SRR2125618	2014		North America	United States	Enteritidis	Enteritidis	1425	36297
SAL_BA1585AA	FSIS1500515	SRR2125617	2014		North America	United States	Enteritidis	Enteritidis	1425	36296
SAL_BA1617AA	FDA00001398	SRR2124539	2009	11	Asia	China	Enteritidis	Enteritidis	1425	36275
SAL_BA1619AA	CFSAN030066	SRR2124535	2012		North America	United States	Enteritidis	Enteritidis	1425	36273
SAL_BA1644AA	15MN00245	SRR2088737	2015	6	North America	United States	Enteritidis	Enteritidis	1425	36250
SAL_BA1645AA	CFSAN032958	SRR2124468	2012		North America	United States	Enteritidis	Enteritidis	1425	36249
SAL_BA1688AA	CFSAN030097	SRR2124395	2012		North America	United States	Enteritidis	Enteritidis	1425	36209
SAL_BA1718AA	CFSAN030086	SRR2124343	2012		North America	United States	Enteritidis	Enteritidis	26162	36182
SAL_BA1721AA	CFSAN030100	SRR2124336	2012		North America	United States	Enteritidis	Enteritidis	1425	36180
SAL_BA1728AA	CFSAN030067	SRR2124324	2012		North America	United States	Enteritidis	Enteritidis	1425	36174
SAL_BA1732AA	CFSAN030081	SRR2124315	2012		North America	United States	Enteritidis	Enteritidis	1425	22973
SAL_BA1782AA	15MN00286	SRR2106118	2015	7	North America	United States	Enteritidis	Enteritidis	1425	36130
SAL_BA1783AA	15MN00285	SRR2106117	2015	7	North America	United States	Enteritidis	Enteritidis	1425	36129
SAL_BA1787AA	15MN00288	SRR2106113	2015	7	North America	United States	Enteritidis	Enteritidis	1425	36126
SAL_BA1788AA	15MN00287	SRR2106112	2015	7	North America	United States	Enteritidis	Enteritidis	1425	36125
SAL_BA1794AA	15MN00268	SRR2106106	2015	7	North America	United States	Enteritidis	Enteritidis	1425	36119
SAL_BA1807AA	2015K-0492	SRR2106066			North America	United States	Enteritidis (Predicted)	Enteritidis	26166	35888
SAL_BA1841AA	FDA00001023	SRR2104599	2007	2	North America	United States	Tennessee	Enteritidis	1425	36086
SAL_BA1894AA	15MN00262	SRR2102499	2015	6	North America	United States	Enteritidis	Enteritidis	1425	8925
SAL_BA1897AA	15MN00255	SRR2102494	2015	6	North America	United States	Enteritidis	Enteritidis	26832	35856
SAL_BA1913AA	15MN00264	SRR2102475	2015	6	North America	United States	Enteritidis	Enteritidis	1425	36030
SAL_BA1915AA	15MN00266	SRR2102471	2015	7	North America	United States	Enteritidis	Enteritidis	1425	36028
SAL_BA1937AA	15MN00267	SRR2102442	2015	7	North America	United States	Enteritidis	Enteritidis	1425	36008
SAL_BA1946AA	15MN00263	SRR2102433	2015	6	North America	United States	Enteritidis	Enteritidis	1425	8925
SAL_BA1948AA	15MN00261	SRR2102430	2015	6	North America	United States	Enteritidis	Enteritidis	26832	36000
SAL_BA1956AA	15MN00265	SRR2102420	2015	6	North America	United States	Enteritidis	Enteritidis	1425	35993
SAL_BA1970AA	15MN00276	SRR2102400	2015	7	North America	United States	Enteritidis	Enteritidis	1425	35982
SAL_BA1984AA	FSIS1502619	SRR2102080	2015		North America	United States	Enteritidis	Enteritidis	1425	35968
SAL_BA1985AA	FSIS1502618	SRR2102061	2015		North America	United States	Enteritidis	Enteritidis	1425	35967
SAL_BA1986AA	FSIS1502617	SRR2102059	2015		North America	United States	Enteritidis	Enteritidis	1425	35966
SAL_BA1987AA	FSIS1502616	SRR2102058	2015		North America	United States	Enteritidis	Enteritidis	1425	35965
SAL_BA2011AA	ADRDL-14-11974	SRR2096607	2014	6	North America	United States	Enteritidis	Enteritidis	1425	35945
SAL_BA2027AA	15MN00243	SRR2096593	2015	6	North America	United States	Enteritidis	Enteritidis	1425	35931
SAL_BA2047AA	15MN00247	SRR2096550	2015	6	North America	United States	Enteritidis	Enteritidis	26832	35914
SAL_BA2075AA	2015K-0494	SRR2095395			North America	United States	Enteritidis (Predicted)	Enteritidis	26166	35888
SAL_BA2076AA	2015K-0493	SRR2095394			North America	United States	Enteritidis (Predicted)	Enteritidis	26166	35891
SAL_BA2077AA	2015K-0491	SRR2095393			North America	United States	Enteritidis (Predicted)	Enteritidis	26166	35890
SAL_BA2078AA	2015K-0490	SRR2095392			North America	United States	Enteritidis (Predicted)	Enteritidis	26166	35889
SAL_BA2079AA	2015K-0489	SRR2095391			North America	United States	Enteritidis (Predicted)	Enteritidis	26166	35888
SAL_BA2080AA	2015K-0488	SRR2095390			North America	United States	Enteritidis (Predicted)	Enteritidis	1425	35887
SAL_BA2081AA	2015K-0487	SRR2095389			North America	United States	Enteritidis (Predicted)	Enteritidis	26166	35886
SAL_BA2082AA	2015K-0486	SRR2095388			North America	United States	Enteritidis (Predicted)	Enteritidis	26166	35885
SAL_BA2085AA	NY56857190	SRR2094564	2015	6	North America	United States	Enteritidis	Enteritidis	1425	35882
SAL_BA2086AA	NY56857138	SRR2094563	2015	6	North America	United States	Enteritidis	Enteritidis	1425	35881
SAL_BA2087AA	NY56857097	SRR2094562	2015	6	North America	United States	Enteritidis	Enteritidis	1425	35880
SAL_BA2088AA	NY56761923	SRR2094561	2015	6	North America	United States	Enteritidis	Enteritidis	1425	35879
SAL_BA2089AA	NY56761711	SRR2094560	2015	6	North America	United States	Enteritidis	Enteritidis	1425	35878
SAL_BA2090AA	NY56761665	SRR2094559	2015	6	North America	United States	Enteritidis	Enteritidis	1425	35877
SAL_BA2096AA	FSIS1501027	SRR2093805	2015		North America	United States	Enteritidis	Enteritidis	1425	35871

SAL_BA2101AA	S14FP01642	SRR2088897	2014	4		Europe	Belgium	Enteritidis	Enteritidis	1425	35869
SAL_BA2102AA	S14FP01640	SRR2088896	2014	4		Europe	Belgium	Enteritidis	Enteritidis	1425	35868
SAL_BA2103AA	S14BD01753	SRR2088895	2014	5		Europe	Belgium	Enteritidis	Enteritidis	1425	35867
SAL_BA2104AA	S14BD01672	SRR2088894	2014	4		Europe	Belgium	Enteritidis	Enteritidis	1425	35866
SAL_BA2105AA	S14BD01605	SRR2088893	2014	4		Europe	Belgium	Enteritidis	Enteritidis	1425	35865
SAL_BA2110AA	15MN00242	SRR2088736	2015	6		North America	United States	Enteritidis	Enteritidis	1425	35860
SAL_BA2111AA	15MN00239	SRR2906162	2015	6		North America	United States	Enteritidis	Enteritidis	1425	35859
SAL_BA2112AA	15MN00240	SRR2088733	2015	6		North America	United States	Enteritidis	Enteritidis	1425	35858
SAL_BA2113AA	15MN00259	SRR2088731	2015	6		North America	United States	Enteritidis	Enteritidis	1425	35855
SAL_BA2114AA	15MN00254	SRR2088730	2015	6		North America	United States	Enteritidis	Enteritidis	26832	35857
SAL_BA2115AA	15MN00253	SRR2088729	2015	6		North America	United States	Enteritidis	Enteritidis	26832	35856
SAL_BA2116AA	15MN00252	SRR2088728	2015	6		North America	United States	Enteritidis	Enteritidis	1425	35855
SAL_BA2117AA	15MN00251	SRR2088727	2015	6		North America	United States	Enteritidis	Enteritidis	26832	35854
SAL_BA2118AA	15MN00244	SRR2088726	2015	6		North America	United States	Enteritidis	Enteritidis	1425	35853
SAL_BA2119AA	15MN00238	SRR2088725	2015	6		North America	United States	Enteritidis	Enteritidis	27944	35852
SAL_BA2120AA	15MN00257	SRR2088724	2015	6		North America	United States	Enteritidis	Enteritidis	1425	35851
SAL_BA2166AA	CFSAN034151	SRR2086995	2013			North America	United States	Enteritidis	Enteritidis	1425	35812
SAL_BA2167AA	CFSAN034136	SRR2086994	2012			North America	United States	Enteritidis	Enteritidis	1425	35811
SAL_BA2168AA	CFSAN034135	SRR2086993	2012			North America	United States	Enteritidis	Enteritidis	1425	35810
SAL_BA2173AA	CFSAN034140	SRR2086988	2012			North America	United States	Enteritidis	Enteritidis	28569	35807
SAL_BA2192AA	CFSAN034133	SRR2086968	2012	11	5	North America	United States	Enteritidis	Enteritidis	1425	35789
SAL_BA2212AA	FDA00001199	SRR2086597	2008	2		Asia	Sri Lanka	Enteritidis	Enteritidis	1425	35775
SAL_BA2231AA	FDA00001212	SRR2086576	2008	3		Asia	India	Enteritidis	Enteritidis	1425	35760
SAL_BA2250AA	FDA00004465	SRR2086559	2008	2		Asia	China	Enteritidis	Enteritidis	1425	35744
SAL_BA2304AA	NY56657591	SRR2085497	2015	6		North America	United States	Enteritidis	Enteritidis	1425	35692
SAL_BA2306AA	NY56657578	SRR2085496	2015	6		North America	United States	Enteritidis	Enteritidis	1425	35690
SAL_BA2308AA	NY56657565	SRR2085495	2015	6		North America	United States	Enteritidis	Enteritidis	1425	35688
SAL_BA2310AA	NY56657552	SRR2085494	2015	6		North America	United States	Enteritidis	Enteritidis	1425	10156
SAL_BA2312AA	NY56582447	SRR2085493	2015	6		North America	United States	Enteritidis	Enteritidis	1425	35685
SAL_BA2314AA	NY56582209	SRR2085492	2015	6		North America	United States	Enteritidis	Enteritidis	1425	35683
SAL_BA2316AA	NY56581629	SRR2085491	2015	6		North America	United States	Enteritidis	Enteritidis	1425	35681
SAL_BA2318AA	NY56581512	SRR2085490	2015	5		North America	United States	Enteritidis	Enteritidis	1425	35679
SAL_BA2321AA	NY56581488	SRR2085489	2015	5		North America	United States	Enteritidis	Enteritidis	1425	35676
SAL_BA2322AA	NY56581414	SRR2085488	2015	6		North America	United States	Enteritidis	Enteritidis	1425	35675
SAL_BA2331AA	2015K-0436	SRR2082996				North America	United States	Enteritidis (Predicted)	Enteritidis	1425	33607
SAL_BA2333AA	2015K-0435	SRR2082995	2015	3		North America	United States		Enteritidis	1425	6087
SAL_BA2336AA	2015K-0434	SRR2082994	2015	3		North America	United States		Enteritidis	1425	35665
SAL_BA2338AA	2015K-0433	SRR2082992				North America	United States	Enteritidis (Predicted)	Enteritidis	1425	35663
SAL_BA2381AA	15MN00234	SRR2082947	2015	6		North America	United States	Enteritidis	Enteritidis	1425	12244
SAL_BA2387AA	15MN00232	SRR2082940	2015	6		North America	United States	Enteritidis	Enteritidis	1425	35633
SAL_BA2392AA	15MN00229	SRR3662621	2015	6		North America	United States	Enteritidis	Enteritidis	1425	35629
SAL_BA2405AA	AZ_TG68272	SRR2082859	2010			North America	United States	Enteritidis	Enteritidis	1425	35616
SAL_BA2413AA	15MN00233	SRR2082853	2015	6		North America	United States	Enteritidis	Enteritidis	1425	12244
SAL_BA2421AA	15MN00228	SRR3662622	2015	6		North America	United States	Enteritidis	Enteritidis	1425	35602
SAL_BA2437AA	15MN00237	SRR2082830	2015	6		North America	United States	Enteritidis	Enteritidis	1425	35589
SAL_BA2497AA	3302/3	ERR349624						Typhi	Enteritidis	3776	35552
SAL_BA2513AA	CFSAN034131	SRR2078917	2012	11	5	North America	United States	Enteritidis	Enteritidis	28569	35543
SAL_BA2538AA	NY56425384	SRR2078828	2015	5		North America	United States	Enteritidis	Enteritidis	1425	35524
SAL_BA2540AA	NY56423955	SRR2078827	2015	5		North America	United States	Enteritidis	Enteritidis	1425	35522
SAL_BA2542AA	NY56423922	SRR2078826	2015	5		North America	United States	Enteritidis	Enteritidis	1425	35520
SAL_BA2544AA	NY56423894	SRR2078825	2015	5		North America	United States	Enteritidis	Enteritidis	1425	35518
SAL_BA2583AA	3540/3	ERR349438						Typhi	Enteritidis	3776	33191
SAL_BA2607AA	ADRDL-14-8130	SRR2077391	2014	4		North America	United States	Enteritidis	Enteritidis	1425	12238
SAL_BA2717AA	CFSAN032967	SRR2075298	2012			North America	United States	Enteritidis	Enteritidis	1425	35370
SAL_BA2819AA	CFSAN032968	SRR2075104	2012			North America	United States	Enteritidis	Enteritidis	1425	35287
SAL_BA2879AA	CFSAN032959	SRR2075048	2012			North America	United States	Enteritidis	Enteritidis	1425	35240
SAL_BA2910AA	CFSAN032964	SRR2075011	2012			North America	United States	Enteritidis	Enteritidis	1425	35213
SAL_BA2916AA	CFSAN033541	SRR2075005	2013			North America	United States	Enteritidis	Enteritidis	1425	35207
SAL_BA2923AA	CFSAN033543	SRR2074998	2012			North America	United States	Enteritidis	Enteritidis	1425	35200

SAL_BA2951AA	NY56360928	SRR2072702	2015	5	North America	United States	Enteritidis	Enteritidis	1425	35180
SAL_BA2953AA	NY56360838	SRR2072700	2015	5	North America	United States	Enteritidis	Enteritidis	1425	35179
SAL_BA2955AA	NY56360751	SRR2072698	2015	5	North America	United States	Enteritidis	Enteritidis	1425	35178
SAL_BA2957AA	NY56316315	SRR2072696	2015	4	North America	United States	Enteritidis	Enteritidis	1425	35177
SAL_BA2958AA	NY56300061	SRR2072695	2015	5	North America	United States	Enteritidis	Enteritidis	1425	35176
SAL_BA2960AA	NY56299973	SRR2072694	2015	5	North America	United States	Enteritidis	Enteritidis	1425	35175
SAL_BA2963AA	NY56299887	SRR2072692	2015	5	North America	United States	Enteritidis	Enteritidis	1425	35174
SAL_BA2965AA	NY56299851	SRR2072691	2015	5	North America	United States	Enteritidis	Enteritidis	1425	35173
SAL_BA2967AA	NY56299818	SRR2072689	2015	5	North America	United States	Enteritidis	Enteritidis	1425	35172
SAL_BA2968AA	NY56296697	SRR2072687	2015	5	North America	United States	Enteritidis	Enteritidis	1425	35171
SAL_BA2970AA	15MN00226	SRR2072493	2015	6	North America	United States	Enteritidis	Enteritidis	1425	34519
SAL_BA2972AA	15MN00224	SRR2072492	2015	6	North America	United States	Enteritidis	Enteritidis	8003	35169
SAL_BA2974AA	15MN00223	SRR2072491	2015	6	North America	United States	Enteritidis	Enteritidis	1425	8925
SAL_BA2976AA	15MN00222	SRR2072490	2015	6	North America	United States	Enteritidis	Enteritidis	1425	35166
SAL_BA2981AA	15MN00227	SRR2072485	2015	6	North America	United States	Enteritidis	Enteritidis	8003	35161
SAL_BA2982AA	15MN00225	SRR2072484	2015	6	North America	United States	Enteritidis	Enteritidis	1425	35160
SAL_BA2984AA	15MN00221	SRR2072483	2015	6	North America	United States	Enteritidis	Enteritidis	1425	35158
SAL_BA3004AA	NY_FSL S10-1644	SRR1002831	2011	10	North America	United States	Enteritidis	Enteritidis	7801	35137
SAL_BA3017AA	NY_FSL S10-1623	SRR1002825	2011	9	North America	United States	Enteritidis	Enteritidis	7801	35137
SAL_BA3028AA	NY-N14325	SRR2070984	2007		North America	United States	Enteritidis	Enteritidis	1425	35129
SAL_BA3062AA	NY56300870	SRR2070870			North America	United States	Enteritidis	Enteritidis	1425	35098
SAL_BA3064AA	NY56300851	SRR2070869			North America	United States	Enteritidis	Enteritidis	1425	35098
SAL_BA3066AA	NY56300821	SRR2070868			North America	United States	Enteritidis	Enteritidis	1425	35098
SAL_BA3072AA	PNUSAS000413	SRR2192239			North America	United States	Enteritidis (Predicted)	Enteritidis	1425	35093
SAL_BA3074AA	PNUSAS000412	SRR2192237			North America	United States	Enteritidis (Predicted)	Enteritidis	1425	35091
SAL_BA3076AA	PNUSAS000411	SRR2192194			North America	United States	Enteritidis (Predicted)	Enteritidis	1425	35089
SAL_BA3078AA	PNUSAS000459	SRR2194018	2015	4	North America	United States	Enteritidis	Enteritidis	1425	35087
SAL_BA3105AA	FSIS1501015	SRR2068054	2015		North America	United States	Enteritidis	Enteritidis	1425	35060
SAL_BA3111AA	FSIS1501012	SRR2067988	2015		North America	United States	Enteritidis	Enteritidis	1425	35054
SAL_BA3122AA	NY56250649	SRR2067480	2015	5	North America	United States	Enteritidis	Enteritidis	1425	35044
SAL_BA3124AA	NY56250681	SRR2067479	2015	5	North America	United States	Enteritidis	Enteritidis	1425	35042
SAL_BA3126AA	NY56250668	SRR2067477	2015	5	North America	United States	Enteritidis	Enteritidis	1425	35040
SAL_BA3128AA	NY56250636	SRR2067476	2015	5	North America	United States	Enteritidis	Enteritidis	1425	35038
SAL_BA3130AA	NY56207015	SRR2067262	2015	5	North America	United States	Enteritidis	Enteritidis	1425	2668
SAL_BA3132AA	NY56207004	SRR2066774	2015	5	North America	United States	Enteritidis	Enteritidis	1425	35035
SAL_BA3134AA	NY56205726	SRR2066432	2015	5	North America	United States	Enteritidis	Enteritidis	1425	35033
SAL_BA3136AA	NY56161314	SRR2065999	2015	5	North America	United States	Enteritidis	Enteritidis	1425	35032
SAL_BA3138AA	NY56250603	SRR2064145	2015	5	North America	United States	Enteritidis	Enteritidis	1425	35028
SAL_BA3140AA	NY56250590	SRR2064144	2015	5	North America	United States	Enteritidis	Enteritidis	1425	2668
SAL_BA3142AA	NY56250555	SRR2064143	2015	5	North America	United States	Enteritidis	Enteritidis	1425	35027
SAL_BA3144AA	NY56206993	SRR2064142	2015	4	North America	United States	Enteritidis	Enteritidis	1425	35024
SAL_BA3146AA	NY56206572	SRR2064141	2015	5	North America	United States	Enteritidis	Enteritidis	28574	35022
SAL_BA3148AA	NY56205781	SRR2064140	2015	5	North America	United States	Enteritidis	Enteritidis	1425	35020
SAL_BA3150AA	NY56205739	SRR2064139	2015	5	North America	United States	Enteritidis	Enteritidis	1425	10271
SAL_BA3152AA	NY56161301	SRR2064136	2015	5	North America	United States	Enteritidis	Enteritidis	1425	35017
SAL_BA3154AA	NY56250709	SRR2064129	2015	5	North America	United States	Enteritidis	Enteritidis	1425	35016
SAL_BA3166AA	NY56250694	SRR2064123	2015	5	North America	United States	Enteritidis	Enteritidis	1425	35004
SAL_BA3170AA	NY56161285	SRR2064121	2015	5	North America	United States	Enteritidis	Enteritidis	1425	35001
SAL_BA3180AA	NY56161250	SRR2064115	2015	5	North America	United States	Enteritidis	Enteritidis	1425	34991
SAL_BA3186AA	15MN00210	SRR2062507	2015	6	North America	United States	Enteritidis	Enteritidis	1425	11501
SAL_BA3188AA	15MN00209	SRR2062506	2015	6	North America	United States	Enteritidis	Enteritidis	1425	8925
SAL_BA3190AA	15MN00213	SRR2062505	2015	6	North America	United States	Enteritidis	Enteritidis	1425	34983
SAL_BA3192AA	15MN00212	SRR2062504	2015	6	North America	United States	Enteritidis	Enteritidis	1425	34981
SAL_BA3194AA	15MN00211	SRR2062503	2015	6	North America	United States	Enteritidis	Enteritidis	1425	34979
SAL_BA3196AA	15MN00208	SRR2062502	2015	5	North America	United States	Enteritidis	Enteritidis	1425	34977
SAL_BA3215AA	PNUSAS000453	SRR2193691			North America	United States	Enteritidis (Predicted)	Enteritidis	1425	34959
SAL_BA3217AA	PNUSAS000452	SRR2193690			North America	United States	Enteritidis (Predicted)	Enteritidis	1425	34957
SAL_BA3220AA	PNUSAS000451	SRR2193689			North America	United States	Enteritidis (Predicted)	Enteritidis	1425	34954
SAL_BA3222AA	PNUSAS000450	SRR2193688			North America	United States	Enteritidis (Predicted)	Enteritidis	1425	34952

SAL_BA3224AA	PNUSAS000449	SRR2058220			North America	United States	Enteritidis (Predicted)	Enteritidis	1425	34950
SAL_BA3241AA	2010K_0600-sc-2013-04-23T	ERR341114					Typhimurium	Enteritidis	1424	34939
SAL_BA3253AA	2010K_0610-sc-2013-04-23T	ERR341102					Enteritidis	Enteritidis	1424	34914
SAL_BA3254AA	2010K_0618-sc-2013-04-23T	ERR341101					Enteritidis	Enteritidis	1424	41278
SAL_BA3256AA	2010K_0549-sc-2013-04-23T	ERR341099					Enteritidis	Enteritidis	1424	41277
SAL_BA3257AA	2010K_0465-sc-2013-04-23T	ERR341098					Enteritidis	Enteritidis	40441	41276
SAL_BA3285AA	2010K_0577-sc-2013-04-23T	ERR341069					Typhimurium	Enteritidis	1424	34914
SAL_BA3432AA	2015K-0316	SRR2578266	2015	2	North America	United States	Enteritidis	Enteritidis	1425	2067
SAL_BA3433AA	2015K-0315	SRR2578267	2015	3	North America	United States	Enteritidis	Enteritidis	1425	25638
SAL_BA3434AA	2015K-0313	SRR2578270	2015	3	North America	United States	Enteritidis	Enteritidis	1425	2067
SAL_BA3443AA	15MN00206	SRR2056006	2015	5	North America	United States	Enteritidis	Enteritidis	1425	11501
SAL_BA3444AA	15MN00204	SRR2056005	2015	5	North America	United States	Enteritidis	Enteritidis	1425	34803
SAL_BA3445AA	15MN00198	SRR2056004	2015	5	North America	United States	Enteritidis	Enteritidis	1425	11501
SAL_BA3446AA	15MN00197	SRR2056003	2015	5	North America	United States	Enteritidis	Enteritidis	1425	34170
SAL_BA3447AA	15MN00207	SRR2056002	2015	5	North America	United States	Enteritidis	Enteritidis	1425	34802
SAL_BA3448AA	15MN00205	SRR2056001	2015	5	North America	United States	Enteritidis	Enteritidis	1425	34801
SAL_BA3449AA	15MN00203	SRR2056000	2015	5	North America	United States	Enteritidis	Enteritidis	1425	34799
SAL_BA3451AA	NY-N14221	SRR2054615	2007		North America	United States	Enteritidis	Enteritidis	1425	34798
SAL_BA3452AA	NY-N14220	SRR2054614	2007		North America	United States	Enteritidis	Enteritidis	1425	34797
SAL_BA3479AA	15MN00172	SRR2054262	2015	5	North America	United States	Enteritidis	Enteritidis	1425	34520
SAL_BA3483AA	15MN00175	SRR2054257	2015	5	North America	United States	Enteritidis	Enteritidis	1425	34774
SAL_BA3512AA	15MN00176	SRR2054221	2015	5	North America	United States	Enteritidis	Enteritidis	1425	34753
SAL_BA3513AA	15MN00174	SRR2054220	2015	4	North America	United States	Enteritidis	Enteritidis	1425	34752
SAL_BA3571AA	15MN00173	SRR2054137	2015	5	North America	United States	Enteritidis	Enteritidis	1425	34704
SAL_BA3679AA	2015K-0363	SRR2571583			North America	United States	Enteritidis (Predicted)	Enteritidis	1425	34621
SAL_BA3681AA	2015K-0361	SRR2571585			North America	United States	Enteritidis (Predicted)	Enteritidis	1425	4169
SAL_BA3683AA	2015K-0362	SRR2571582	2015	4	North America	United States	Enteritidis	Enteritidis	1425	4169
SAL_BA3685AA	2015K-0360	SRR2571581	2015	4	North America	United States	Enteritidis	Enteritidis	1425	4169
SAL_BA3687AA	2015K-0359	SRR2571586	2015	4	North America	United States	Enteritidis	Enteritidis	1425	4169
SAL_BA3689AA	2015K-0357	SRR2571587	2015	5	North America	United States	Enteritidis	Enteritidis	1425	4169
SAL_BA3691AA	2015K-0358	SRR2571584	2015	4	North America	United States	Enteritidis	Enteritidis	1425	4169
SAL_BA3700AA	2015K-0334	SRR2577440			North America	United States	Enteritidis (Predicted)	Enteritidis	26543	34607
SAL_BA3702AA	2015K-0333	SRR2577308			North America	United States	Enteritidis (Predicted)	Enteritidis	26543	34605
SAL_BA3703AA	2015K-0332	SRR2577307			North America	United States	Enteritidis (Predicted)	Enteritidis	26543	34604
SAL_BA3709AA	2015K-0314	SRR2051445			North America	United States	Enteritidis (Predicted)	Enteritidis	1425	34600
SAL_BA3712AA	2015K-0307	SRR2051443			North America	United States	Enteritidis (Predicted)	Enteritidis	1425	25745
SAL_BA3781AA	672456-sc-2013-04-16T07:3	ERR338971					Enteritidis	Enteritidis	1425	41294
SAL_BA3783AA	649762-sc-2013-04-16T07:3	ERR338970					Enteritidis	Enteritidis	1425	41293
SAL_BA3787AA	645902-sc-2013-04-16T07:3	ERR338968					Enteritidis	Enteritidis	3888	41292
SAL_BA3789AA	645899-sc-2013-04-16T07:3	ERR338967					Enteritidis	Enteritidis	3888	41292
SAL_BA3794AA	672461-sc-2013-04-16T07:3	ERR338965					Enteritidis	Enteritidis	3888	41036
SAL_BA3796AA	658910-sc-2013-04-16T07:3	ERR338964					Enteritidis	Enteritidis	3888	41291
SAL_BA3804AA	651579-sc-2013-04-16T07:3	ERR338960					Enteritidis	Enteritidis	1425	41290
SAL_BA3808AA	672246-sc-2013-04-16T07:3	ERR338958					Enteritidis	Enteritidis	3888	41036
SAL_BA3810AA	655136-sc-2013-04-16T07:3	ERR338957					Enteritidis	Enteritidis	3888	41443
SAL_BA3812AA	649763-sc-2013-04-16T07:3	ERR338956					Enteritidis	Enteritidis	1425	34533
SAL_BA3813AA	682965-sc-2013-04-16T07:3	ERR338955					Enteritidis	Enteritidis	3888	34531
SAL_BA3828AA	383167-sc-2013-04-16T07:3	ERR338948					Enteritidis	Enteritidis	1425	34419
SAL_BA3832AA	333519-sc-2013-04-16T07:3	ERR338946					Enteritidis	Enteritidis	1425	41427
SAL_BA3836AA	466244-sc-2013-04-16T07:3	ERR338944					Enteritidis	Enteritidis	1425	41426
SAL_BA3838AA	1025081-sc-2013-04-16T07:	ERR338943					Enteritidis	Enteritidis	3815	34524
SAL_BA3840AA	384986-sc-2013-04-16T07:3	ERR338942					Enteritidis	Enteritidis	1425	34419
SAL_BA3844AA	1025080-sc-2013-04-16T07:	ERR338940					Enteritidis	Enteritidis	3815	41314
SAL_BA3846AA	348816-sc-2013-04-16T07:3	ERR338939					Enteritidis	Enteritidis	1425	34404
SAL_BA3848AA	249299-sc-2013-04-16T07:3	ERR338938					Enteritidis	Enteritidis	1425	34412
SAL_BA3851AA	236888-sc-2013-04-16T07:3	ERR338937					Enteritidis	Enteritidis	1425	41313
SAL_BA3853AA	171403-sc-2013-04-16T07:3	ERR338936					Enteritidis	Enteritidis	1425	41312
SAL_BA3854AA	15MN00171	SRR2047362	2015	5	North America	United States	Enteritidis	Enteritidis	1425	34520
SAL_BA3856AA	15MN00170	SRR2047361	2015	5	North America	United States	Enteritidis	Enteritidis	1425	34519

SAL_BA3857AA	238577-sc-2013-04-16T07:3	ERR338934					Enteritidis	Enteritidis	1425	34393
SAL_BA3858AA	15MN00169	SRR2047360	2015	5	North America	United States	Enteritidis	Enteritidis	27901	34518
SAL_BA3859AA	15MN00165	SRR2047359	2015	5	North America	United States	Enteritidis	Enteritidis	1425	2323
SAL_BA3861AA	318064-sc-2013-04-16T07:3	ERR338932					Enteritidis	Enteritidis	1425	41311
SAL_BA3862AA	15MN00164	SRR2047358	2015	5	North America	United States	Enteritidis	Enteritidis	1425	34517
SAL_BA3864AA	15MN00163	SRR2047357	2015	5	North America	United States	Enteritidis	Enteritidis	1425	34516
SAL_BA3865AA	586365-sc-2013-04-16T07:3	ERR338930					Enteritidis	Enteritidis	1425	41310
SAL_BA3873AA	201566-sc-2013-04-16T07:3	ERR338926					Enteritidis	Enteritidis	1425	41309
SAL_BA3881AA	596719-sc-2013-04-16T07:3	ERR338922					Enteritidis	Enteritidis	1425	34512
SAL_BA3883AA	575404-sc-2013-04-16T07:3	ERR338921					Enteritidis	Enteritidis	1425	34500
SAL_BA3885AA	177883-sc-2013-04-16T07:3	ERR338920					Enteritidis	Enteritidis	1425	34510
SAL_BA3887AA	360688-sc-2013-04-16T07:3	ERR338919					Enteritidis	Enteritidis	1425	34399
SAL_BA3894AA	511112-sc-2013-04-16T07:3	ERR338916					Enteritidis	Enteritidis	3888	41308
SAL_BA3896AA	512103-sc-2013-04-16T07:3	ERR338915					Enteritidis	Enteritidis	3888	34369
SAL_BA3901AA	238661-sc-2013-04-16T07:3	ERR338912					Enteritidis	Enteritidis	1425	34354
SAL_BA3905AA	462913-sc-2013-04-16T07:3	ERR338910					Enteritidis	Enteritidis	1425	41307
SAL_BA3908AA	236885-sc-2013-04-16T07:3	ERR338908					Enteritidis	Enteritidis	1425	41306
SAL_BA3914AA	463134-sc-2013-04-16T07:3	ERR338905					Enteritidis	Enteritidis	1425	34500
SAL_BA3931AA	CFSAN032557	SRR2047316	2014	5	North America	United States	Enteritidis (Predicted)	Enteritidis	1425	34488
SAL_BA3939AA	CFSAN032553	SRR2047312	2014	5	North America	United States	Enteritidis (Predicted)	Enteritidis	27901	34482
SAL_BA3941AA	CFSAN032549	SRR2047311	2014	5	North America	United States	Enteritidis (Predicted)	Enteritidis	1425	34480
SAL_BA3945AA	CFSAN032550	SRR2047309	2014	5	North America	United States	Enteritidis (Predicted)	Enteritidis	1425	34476
SAL_BA3956AA	CFSAN032543	SRR2047304	2014	5	North America	United States	Enteritidis (Predicted)	Enteritidis	1425	34467
SAL_BA3967AA	CFSAN032539	SRR2047299	2014	5	North America	United States	Enteritidis (Predicted)	Enteritidis	1425	34458
SAL_BA3973AA	IEH-NGS-SAL-02822	SRR2047296	2014	5	North America	United States	Enteritidis (Predicted)	Enteritidis	1425	34453
SAL_BA3979AA	CFSAN032347	SRR2047293	2014	5	North America	United States	Enteritidis (Predicted)	Enteritidis	1425	34448
SAL_BA3983AA	WAPHL_SAL-A00780	SRR2047291	2006		North America	United States	Enteritidis	Enteritidis	1425	34445
SAL_BA3990AA	PNUSAS000399	SRR2043725	2015	4	North America	United States	Enteritidis	Enteritidis	1425	34439
SAL_BA3992AA	PNUSAS000398	SRR2043724	2015	4	North America	United States	Enteritidis	Enteritidis	1425	34438
SAL_BA3994AA	PNUSAS000397	SRR2043723	2015	4	North America	United States	Enteritidis	Enteritidis	1425	34437
SAL_BA4015AA	250909-sc-2013-04-16T07:3	ERR338318					Enteritidis	Enteritidis	1425	34419
SAL_BA4019AA	472274-sc-2013-04-16T07:3	ERR338316					Enteritidis	Enteritidis	1425	34500
SAL_BA4021AA	317576-sc-2013-04-16T07:3	ERR338315					Enteritidis	Enteritidis	1425	34415
SAL_BA4023AA	496155-sc-2013-04-16T07:3	ERR338314					Enteritidis	Enteritidis	3888	34369
SAL_BA4025AA	249309-sc-2013-04-16T07:3	ERR338313					Enteritidis	Enteritidis	1425	34412
SAL_BA4027AA	569257-sc-2013-04-16T07:3	ERR338312					Enteritidis	Enteritidis	3888	34410
SAL_BA4029AA	380425-sc-2013-04-16T07:3	ERR338311					Enteritidis	Enteritidis	1425	34408
SAL_BA4030AA	319184-sc-2013-04-16T07:3	ERR338310					Enteritidis	Enteritidis	1425	41305
SAL_BA4036AA	348823-sc-2013-04-16T07:3	ERR338307					Enteritidis	Enteritidis	1425	34404
SAL_BA4038AA	PNUSAS000360	SRR2043701			North America	United States	Enteritidis	Enteritidis	1425	34403
SAL_BA4039AA	492225-sc-2013-04-16T07:3	ERR338306					Enteritidis	Enteritidis	3888	34381
SAL_BA4040AA	PNUSAS000359	SRR2043700			North America	United States	Enteritidis	Enteritidis	1425	34402
SAL_BA4043AA	570523-sc-2013-04-16T07:3	ERR338304					Enteritidis	Enteritidis	1425	34399
SAL_BA4045AA	533511-sc-2013-04-16T07:3	ERR338303					Enteritidis	Enteritidis	1425	34397
SAL_BA4047AA	358520-sc-2013-04-16T07:3	ERR338302					Enteritidis	Enteritidis	1425	41304
SAL_BA4050AA	254602-sc-2013-04-16T07:3	ERR338301					Enteritidis	Enteritidis	1425	41425
SAL_BA4051AA	204225-sc-2013-04-16T07:3	ERR338300					Enteritidis	Enteritidis	1425	41309
SAL_BA4053AA	238579-sc-2013-04-16T07:3	ERR338299					Enteritidis	Enteritidis	1425	34393
SAL_BA4054AA	PNUSAS000352	SRR2043693	2015	4	North America	United States	Enteritidis	Enteritidis	1425	34392
SAL_BA4057AA	290914-sc-2013-04-16T07:3	ERR338297					Enteritidis	Enteritidis	1425	34390
SAL_BA4059AA	626285-sc-2013-04-16T07:3	ERR338296					Enteritidis	Enteritidis	1425	34380
SAL_BA4061AA	485533-sc-2013-04-16T07:3	ERR338295					Enteritidis	Enteritidis	1425	34386
SAL_BA4063AA	459755-sc-2013-04-16T07:3	ERR338294					Enteritidis	Enteritidis	3888	34384
SAL_BA4066AA	283957-sc-2013-04-16T07:3	ERR338293					Enteritidis	Enteritidis	1425	41500
SAL_BA4068AA	119691-sc-2013-04-16T07:3	ERR338292					Enteritidis	Enteritidis	1425	41320
SAL_BA4070AA	468192-sc-2013-04-16T07:3	ERR338291					Enteritidis	Enteritidis	1425	34382
SAL_BA4072AA	684397-sc-2013-04-16T07:3	ERR338290					Enteritidis	Enteritidis	3888	34369
SAL_BA4073AA	2015K-0280	SRR2043477			North America	United States	Enteritidis (Predicted)	Enteritidis	1425	21858
SAL_BA4074AA	492221-sc-2013-04-16T07:3	ERR338289					Enteritidis	Enteritidis	3888	34381

SAL_BA4075AA	2015K-0278	SRR2043476			North America	United States	Enteritidis (Predicted)	Enteritidis	1425	21858
SAL_BA4076AA	626279-sc-2013-04-16T07:3	ERR338288					Enteritidis	Enteritidis	1425	34380
SAL_BA4077AA	2015K-0279	SRR2043475	2014	10	North America	United States	Enteritidis (Predicted)	Enteritidis	1425	34379
SAL_BA4078AA	236817-sc-2013-04-16T07:3	ERR338287					Enteritidis	Enteritidis	1425	34378
SAL_BA4079AA	2015K-0277	SRR2043474	2014	11	North America	United States	Enteritidis (Predicted)	Enteritidis	1425	34377
SAL_BA4080AA	468205-sc-2013-04-16T07:3	ERR338286					Enteritidis	Enteritidis	1425	34376
SAL_BA4082AA	245880-sc-2013-04-16T07:3	ERR338285					Enteritidis	Enteritidis	1425	34374
SAL_BA4083AA	CFSAN032367	SRR2043313	2014	6	North America	United States	Enteritidis (Predicted)	Enteritidis	1425	34373
SAL_BA4085AA	481587-sc-2013-04-16T07:3	ERR338284					Enteritidis	Enteritidis	1425	41498
SAL_BA4087AA	172848-sc-2013-04-16T07:3	ERR338283					Enteritidis	Enteritidis	1425	34370
SAL_BA4089AA	684394-sc-2013-04-16T07:3	ERR338282					Enteritidis	Enteritidis	3888	34369
SAL_BA4091AA	570890-sc-2013-04-16T07:3	ERR338281					Enteritidis	Enteritidis	3888	34367
SAL_BA4092AA	CFSAN032394	SRR2043308	2014	6	North America	United States	Enteritidis (Predicted)	Enteritidis	1425	34366
SAL_BA4093AA	631484-sc-2013-04-16T07:3	ERR338280					Enteritidis	Enteritidis	1425	41497
SAL_BA4095AA	247274-sc-2013-04-16T07:3	ERR338279					Enteritidis	Enteritidis	1425	34419
SAL_BA4097AA	180211-sc-2013-04-16T07:3	ERR338278					Enteritidis	Enteritidis	1425	41322
SAL_BA4102AA	CFSAN032354	SRR2043303	2014	5	North America	United States	Enteritidis (Predicted)	Enteritidis	27901	34361
SAL_BA4103AA	1029414-sc-2013-04-16T07:	ERR338275					Enteritidis	Enteritidis	1425	34360
SAL_BA4104AA	IEH-NGS-SAL-02819	SRR2043302	2014	6	North America	United States	Enteritidis (Predicted)	Enteritidis	1425	34359
SAL_BA4105AA	508751-sc-2013-04-16T07:3	ERR338274					Enteritidis	Enteritidis	3888	41308
SAL_BA4107AA	584640-sc-2013-04-16T07:3	ERR338273					Enteritidis	Enteritidis	1425	41310
SAL_BA4108AA	CFSAN032382	SRR2043300	2014	6	North America	United States	Enteritidis (Predicted)	Enteritidis	1425	34357
SAL_BA4109AA	624887-sc-2013-04-16T07:3	ERR338272					Enteritidis	Enteritidis	3888	41321
SAL_BA4111AA	120673-sc-2013-04-16T07:3	ERR338271					Enteritidis	Enteritidis	1425	41320
SAL_BA4112AA	CFSAN032366	SRR2043298	2014	6	North America	United States	Enteritidis (Predicted)	Enteritidis	1425	34355
SAL_BA4113AA	238663-sc-2013-04-16T07:3	ERR338270					Enteritidis	Enteritidis	1425	34354
SAL_BA4119AA	454790-sc-2013-04-16T07:3	ERR338267					Enteritidis	Enteritidis	3888	41319
SAL_BA4121AA	503944-sc-2013-04-16T07:3	ERR338266					Enteritidis	Enteritidis	1425	41316
SAL_BA4123AA	584643-sc-2013-04-16T07:3	ERR338265					Enteritidis	Enteritidis	1425	41318
SAL_BA4125AA	616854-sc-2013-04-16T07:3	ERR338264					Enteritidis	Enteritidis	3888	41315
SAL_BA4131AA	332287-sc-2013-04-16T07:3	ERR338261					Enteritidis	Enteritidis	1425	41317
SAL_BA4133AA	332662-sc-2013-04-16T07:3	ERR338260					Enteritidis	Enteritidis	1425	41317
SAL_BA4137AA	503933-sc-2013-04-16T07:3	ERR338258					Enteritidis	Enteritidis	1425	41316
SAL_BA4140AA	CFSAN032393	SRR2043283	2014	6	North America	United States	Enteritidis (Predicted)	Enteritidis	1425	34341
SAL_BA4141AA	616851-sc-2013-04-16T07:3	ERR338256					Enteritidis	Enteritidis	3888	41315
SAL_BA4144AA	CFSAN032390	SRR2043281	2014	6	North America	United States	Enteritidis (Predicted)	Enteritidis	1425	32445
SAL_BA4145AA	283954-sc-2013-04-16T07:3	ERR338254					Enteritidis	Enteritidis	1425	34419
SAL_BA4146AA	CFSAN032384	SRR2043280	2014	6	North America	United States	Enteritidis (Predicted)	Enteritidis	1425	34339
SAL_BA4147AA	361592-sc-2013-04-16T07:3	ERR338253					Enteritidis	Enteritidis	1425	34399
SAL_BA4149AA	482916-sc-2013-04-16T07:3	ERR338252					Enteritidis	Enteritidis	1425	41307
SAL_BA4154AA	CFSAN032351	SRR2043276	2014	5	North America	United States	Enteritidis (Predicted)	Enteritidis	28592	34334
SAL_BA4207AA	A134	ERR338066					Typhi	Enteritidis	7802	31241
SAL_BA4213AA	A129	ERR338063					Typhi	Enteritidis	7802	34305
SAL_BA4221AA	A124	ERR338059					Typhi	Enteritidis	1425	34295
SAL_BA4226AA	A120	ERR338056					Typhi	Enteritidis	7802	34296
SAL_BA4227AA	CFSAN032344	SRR2039250	2014	5	North America	United States	Enteritidis (Predicted)	Enteritidis	1425	32809
SAL_BA4228AA	A119	ERR338055					Typhi	Enteritidis	1425	34295
SAL_BA4230AA	A117	ERR338054					Typhi	Enteritidis	7802	31241
SAL_BA4246AA	CFSAN032325	SRR2039241	2014	5	North America	United States	Enteritidis (Predicted)	Enteritidis	1425	34280
SAL_BA4261AA	CFSAN032307	SRR2039158	2014	5	North America	United States	Enteritidis (Predicted)	Enteritidis	1425	34266
SAL_BA4263AA	CFSAN032304	SRR2039157	2014	5	North America	United States	Enteritidis (Predicted)	Enteritidis	1425	34264
SAL_BA4270AA	CFSAN032306	SRR2039153	2014	5	North America	United States	Enteritidis (Predicted)	Enteritidis	1425	32809
SAL_BA4295AA	368908	ERR338022					Typhi	Enteritidis	1425	34241
SAL_BA4302AA	CFSAN032300	SRR2039137	2014	5	North America	United States	Enteritidis (Predicted)	Enteritidis	1425	34234
SAL_BA4338AA	CFSAN032305	SRR2039119	2014	5	North America	United States	Enteritidis (Predicted)	Enteritidis	1425	34207
SAL_BA4382AA	15MN00153	SRR2035447	2015	4	North America	United States	Enteritidis	Enteritidis	1425	34173
SAL_BA4385AA	15MN00156	SRR2035444	2015	5	North America	United States	Enteritidis	Enteritidis	1425	34170
SAL_BA4387AA	15MN00155	SRR2035443	2015	5	North America	United States	Enteritidis	Enteritidis	1425	34168
SAL_BA4389AA	15MN00152	SRR2035441	2015	4	North America	United States	Enteritidis	Enteritidis	1425	34167

SAL_BA4397AA	15MN00147	SRR2035434	2015	4	North America	United States	Enteritidis	Enteritidis	1425	34161
SAL_BA4399AA	IEH-NGS-SAL-02708	SRR2035433	2014	5	North America	United States	Enteritidis (Predicted)	Enteritidis	1425	34159
SAL_BA4401AA	15MN00154	SRR2035430	2015	5	North America	United States	Enteritidis	Enteritidis	1425	11501
SAL_BA4403AA	15MN00067	SRR2035429	2015	2	North America	United States	Enteritidis	Enteritidis	1425	34156
SAL_BA4405AA	15MN00146	SRR2035428	2015	4	North America	United States	Enteritidis	Enteritidis	1425	34155
SAL_BA4409AA	CFSAN031253	SRR2035426	2014	5	North America	United States	Enteritidis (Predicted)	Enteritidis	1425	34151
SAL_BA4411AA	CFSAN031250	SRR2035425	2014	5	North America	United States	Enteritidis (Predicted)	Enteritidis	1425	34149
SAL_BA4414AA	15MN00144	SRR2035412	2015	4	North America	United States	Enteritidis	Enteritidis	27901	34146
SAL_BA4416AA	CFSAN032297	SRR2035411	2014	5	North America	United States	Enteritidis (Predicted)	Enteritidis	1425	34145
SAL_BA4418AA	CFSAN031260	SRR2035410	2014	5	North America	United States	Enteritidis (Predicted)	Enteritidis	1425	34143
SAL_BA4422AA	15MN00158	SRR2035408	2015	5	North America	United States	Enteritidis	Enteritidis	1425	17527
SAL_BA4424AA	15MN00157	SRR2035407	2015	5	North America	United States	Enteritidis	Enteritidis	1425	17527
SAL_BA4426AA	15MN00149	SRR2035406	2015	4	North America	United States	Enteritidis	Enteritidis	1425	34138
SAL_BA4428AA	15MN00148	SRR2035405	2015	4	North America	United States	Enteritidis	Enteritidis	1425	34136
SAL_BA4430AA	15MN00145	SRR2035401	2015	4	North America	United States	Enteritidis	Enteritidis	1425	34134
SAL_BA4442AA	CFSAN031259	SRR2035394	2014	5	North America	United States	Enteritidis (Predicted)	Enteritidis	1425	34123
SAL_BA4445AA	CFSAN031249	SRR2035391	2014	5	North America	United States	Enteritidis (Predicted)	Enteritidis	1425	34120
SAL_BA4451AA	CFSAN032298	SRR2035387	2014	5	North America	United States	Enteritidis (Predicted)	Enteritidis	1425	34114
SAL_BA4538AA	MM_SW824_14_2	ERR197441					Enteritidis	Enteritidis	28601	34068
SAL_BA4540AA	MM_SW824_14_1	ERR197440					Enteritidis	Enteritidis	1425	34067
SAL_BA4542AA	MM_SW824_14	ERR197439					Enteritidis	Enteritidis	1425	34066
SAL_BA4544AA	MM_SW824_10	ERR197438					Enteritidis	Enteritidis	1425	34065
SAL_BA4545AA	MM_SW824_7	ERR197437					Enteritidis	Enteritidis	1425	34064
SAL_BA4547AA	MM_SW824_4	ERR197436					Enteritidis	Enteritidis	1425	34062
SAL_BA4550AA	MM_SW824_1	ERR197435					Enteritidis	Enteritidis	1425	34061
SAL_BA4552AA	MM_SW824_0	ERR197434					Enteritidis	Enteritidis	1425	34022
SAL_BA4554AA	MM_PT4_14_2	ERR197433					Enteritidis	Enteritidis	1425	34055
SAL_BA4555AA	MM_PT4_14_1	ERR197432					Enteritidis	Enteritidis	1425	34055
SAL_BA4557AA	MM_PT4_14	ERR197431					Enteritidis	Enteritidis	1425	34055
SAL_BA4559AA	MM_PT4_10	ERR197430					Enteritidis	Enteritidis	1425	34055
SAL_BA4561AA	MM_PT4_7	ERR197429					Enteritidis	Enteritidis	1425	34055
SAL_BA4563AA	MM_PT4_4	ERR197428					Enteritidis	Enteritidis	1425	34055
SAL_BA4565AA	MM_PT4_1	ERR197427					Enteritidis	Enteritidis	1425	34055
SAL_BA4567AA	MM_PT4_0	ERR197426					Enteritidis	Enteritidis	1425	34011
SAL_BA4587AA	LB_SW824_14_2	ERR197416					Enteritidis	Enteritidis	1425	34039
SAL_BA4589AA	LB_SW824_14_1	ERR197415					Enteritidis	Enteritidis	1425	34037
SAL_BA4591AA	LB_SW824_14	ERR197414					Enteritidis	Enteritidis	1425	34022
SAL_BA4596AA	LB_SW824_10	ERR197412					Enteritidis	Enteritidis	1425	34031
SAL_BA4598AA	LB_SW824_7	ERR197411					Enteritidis	Enteritidis	1425	34029
SAL_BA4601AA	LB_SW824_4	ERR197410					Enteritidis	Enteritidis	1425	34026
SAL_BA4603AA	LB_SW824_1	ERR197409					Enteritidis	Enteritidis	1425	34024
SAL_BA4604AA	CFSAN031220	SRR2029478	2014	4	North America	United States	Enteritidis (Predicted)	Enteritidis	1425	34023
SAL_BA4605AA	LB_SW824_0	ERR197408					Enteritidis	Enteritidis	1425	34022
SAL_BA4606AA	CFSAN031221	SRR2029477	2014	5	North America	United States	Enteritidis (Predicted)	Enteritidis	1425	34021
SAL_BA4607AA	LB_PT4_14_2	ERR197407					Enteritidis	Enteritidis	1425	34011
SAL_BA4609AA	LB_PT4_14_1	ERR197406					Enteritidis	Enteritidis	1425	34011
SAL_BA4610AA	IEH-NGS-SAL-02674	SRR2029475	2014	4	North America	United States	Enteritidis (Predicted)	Enteritidis	-1739	34019
SAL_BA4611AA	LB_PT4_14	ERR197405					Enteritidis	Enteritidis	1425	34011
SAL_BA4613AA	LB_PT4_13	ERR197404					Enteritidis	Enteritidis	1425	34017
SAL_BA4615AA	LB_PT4_10	ERR197403					Enteritidis	Enteritidis	1425	34011
SAL_BA4617AA	LB_PT4_7	ERR197402					Enteritidis	Enteritidis	1425	34011
SAL_BA4618AA	IEH-NGS-SAL-02670	SRR2029471	2014	4	North America	United States	Enteritidis (Predicted)	Enteritidis	1425	34014
SAL_BA4620AA	LB_PT4_4	ERR197401					Enteritidis	Enteritidis	1425	34011
SAL_BA4622AA	LB_PT4_1	ERR197400					Enteritidis	Enteritidis	1425	34011
SAL_BA4623AA	CFSAN031212	SRR2029468	2014	5	North America	United States	Enteritidis (Predicted)	Enteritidis	1425	32445
SAL_BA4624AA	LB_PT4_0	ERR197399					Enteritidis	Enteritidis	1425	34011
SAL_BA4631AA	CFSAN031207	SRR2029464	2014	4	North America	United States	Enteritidis (Predicted)	Enteritidis	1425	34005
SAL_BA4638AA	PNUSAS000351	SRR2025325	2015	4	North America	United States	Enteritidis	Enteritidis	1425	34000
SAL_BA4640AA	PNUSAS000350	SRR2025324			North America	United States	Enteritidis (Predicted)	Enteritidis	1425	10874

SAL_BA4642AA	PNUSAS000349	SRR2025323			North America	United States	Enteritidis (Predicted)	Enteritidis	1425	2067
SAL_BA4644AA	PNUSAS000347	SRR2025321			North America	United States	Enteritidis (Predicted)	Enteritidis	1425	33997
SAL_BA4646AA	PNUSAS000346	SRR2025320			North America	United States	Enteritidis (Predicted)	Enteritidis	1425	33996
SAL_BA4648AA	PNUSAS000345	SRR2025319			North America	United States	Enteritidis (Predicted)	Enteritidis	1425	16644
SAL_BA4650AA	PNUSAS000344	SRR2025318			North America	United States	Enteritidis (Predicted)	Enteritidis	1425	25745
SAL_BA4652AA	PNUSAS000343	SRR2025317			North America	United States	Enteritidis (Predicted)	Enteritidis	1425	33993
SAL_BA4653AA	PNUSAS000342	SRR2025316			North America	United States	Enteritidis (Predicted)	Enteritidis	1425	33992
SAL_BA4657AA	PNUSAS000339	SRR2025314	2015	4	North America	United States	Enteritidis	Enteritidis	1425	33988
SAL_BA4659AA	PNUSAS000340	SRR2025313	2015	4	North America	United States	Enteritidis	Enteritidis	1425	33986
SAL_BA4662AA	PNUSAS000337	SRR2025311			North America	United States	Enteritidis (Predicted)	Enteritidis	1425	33984
SAL_BA4664AA	PNUSAS000335	SRR2025309			North America	United States	Enteritidis (Predicted)	Enteritidis	7801	25753
SAL_BA4666AA	PNUSAS000336	SRR2025308			North America	United States	Enteritidis (Predicted)	Enteritidis	1425	12315
SAL_BA4668AA	PNUSAS000334	SRR2025307			North America	United States	Enteritidis (Predicted)	Enteritidis	1425	1808
SAL_BA4680AA	PNUSAS000280	SRR2025301			North America	United States	Enteritidis (Predicted)	Enteritidis	1425	33973
SAL_BA4688AA	IEH_NGS_SAL_00292	SRR961991	2008	8	North America	United States	Enteritidis (Predicted)	Enteritidis	1425	33967
SAL_BA4694AA	IEH_NGS_SAL_00207	SRR961988	2008	3	North America	United States	Enteritidis (Predicted)	Enteritidis	1425	33961
SAL_BA4731AA	MDH-2014-00842	SRR1514744	2014	7	North America	United States	Enteritidis	Enteritidis	1425	33930
SAL_BA4733AA	MDH-2014-00841	SRR1514726	2014	7	North America	United States	Enteritidis	Enteritidis	1425	12265
SAL_BA4735AA	CFSAN031197	SRR2017888	2014	4	North America	United States	Enteritidis (Predicted)	Enteritidis	1425	33927
SAL_BA4743AA	NY-N13666	SRR2017884	2007		North America	United States	Enteritidis	Enteritidis	1425	30959
SAL_BA4758AA	CFSAN031190	SRR2017876	2014	5	North America	United States	Enteritidis (Predicted)	Enteritidis	1425	33909
SAL_BA4793AA	CFSAN031192	SRR2017855	2014	4	North America	United States	Enteritidis (Predicted)	Enteritidis	1425	16630
SAL_BA4799AA	IEH-NGS-SAL-02639	SRR2017852	2014	4	North America	United States	Enteritidis (Predicted)	Enteritidis	1425	33886
SAL_BA4847AA	CFSAN022634	SRR2016682			North America	United States	Enteritidis	Enteritidis	1425	33856
SAL_BA4861AA	IEH-NGS-SAL-02617	SRR2015924	2014	4	North America	United States	Enteritidis (Predicted)	Enteritidis	1425	33843
SAL_BA4875AA	A147	ERR331213					Typhi	Enteritidis	7802	41035
SAL_BA4881AA	CFSAN031177	SRR2015824	2014	4	North America	United States	Enteritidis (Predicted)	Enteritidis	1425	33829
SAL_BA4982AA	CFSAN031164	SRR2015687	2014	4	North America	United States	Enteritidis (Predicted)	Enteritidis	1425	33742
SAL_BA5006AA	VA-WGS-00435	SRR2015664	2014	1	North America	United States	Enteritidis	Enteritidis	1425	33718
SAL_BA5012AA	CFSAN024486	SRR2015661	2009	11	North America	Mexico	Berta	Enteritidis	1425	33712
SAL_BA5091AA	PNUSAS000306	SRR2015095			North America	United States	Enteritidis	Enteritidis	1425	33645
SAL_BA5093AA	PNUSAS000307	SRR2015094			North America	United States	Enteritidis	Enteritidis	1425	33643
SAL_BA5095AA	PNUSAS000305	SRR2015093			North America	United States	Enteritidis	Enteritidis	1425	33641
SAL_BA5099AA	PNUSAS000304	SRR2015090			North America	United States	Enteritidis	Enteritidis	1425	33637
SAL_BA5101AA	PNUSAS000299	SRR2015089	2015	4	North America	United States	Enteritidis	Enteritidis	1425	33634
SAL_BA5105AA	PNUSAS000294	SRR2015087	2015	4	North America	United States	Enteritidis	Enteritidis	8003	12492
SAL_BA5107AA	PNUSAS000293	SRR2015086	2015	4	North America	United States	Enteritidis	Enteritidis	1425	17527
SAL_BA5109AA	PNUSAS000292	SRR2015085	2015	4	North America	United States	Enteritidis	Enteritidis	1425	33629
SAL_BA5111AA	PNUSAS000290	SRR2015084	2015	4	North America	United States	Enteritidis	Enteritidis	1425	33628
SAL_BA5113AA	PNUSAS000291	SRR2015083			North America	United States	Enteritidis (Predicted)	Enteritidis	1425	33627
SAL_BA5115AA	PNUSAS000289	SRR2015082			North America	United States	Enteritidis (Predicted)	Enteritidis	1425	25745
SAL_BA5117AA	PNUSAS000288	SRR2015081			North America	United States	Enteritidis (Predicted)	Enteritidis	1425	33625
SAL_BA5119AA	PNUSAS000287	SRR2015080	2015	3	North America	United States	Enteritidis	Enteritidis	28616	33623
SAL_BA5122AA	PNUSAS000286	SRR2015079	2015	3	North America	United States	Enteritidis	Enteritidis	1425	2067
SAL_BA5123AA	PNUSAS000283	SRR2015078	2015	3	North America	United States	Enteritidis	Enteritidis	1425	33620
SAL_BA5126AA	PNUSAS000281	SRR2015077	2015	3	North America	United States	Enteritidis	Enteritidis	1425	33617
SAL_BA5127AA	PNUSAS000282	SRR2015076	2015	3	North America	United States	Enteritidis	Enteritidis	1425	33616
SAL_BA5129AA	PNUSAS000279	SRR2015075	2015	1	North America	United States	Enteritidis	Enteritidis	1425	33615
SAL_BA5131AA	PNUSAS000278	SRR2015074			North America	United States	Enteritidis (Predicted)	Enteritidis	1425	33614
SAL_BA5133AA	PNUSAS000277	SRR2015073	2015	1	North America	United States	Enteritidis	Enteritidis	1425	33612
SAL_BA5135AA	PNUSAS000276	SRR2015072	2015	3	North America	United States	Enteritidis	Enteritidis	1425	33610
SAL_BA5137AA	PNUSAS000275	SRR2015071	2015	3	North America	United States	Enteritidis	Enteritidis	1425	2323
SAL_BA5139AA	PNUSAS000274	SRR2015070	2015	3	North America	United States	Enteritidis	Enteritidis	1425	2323
SAL_BA5141AA	PNUSAS000273	SRR2015069	2015	3	North America	United States	Enteritidis	Enteritidis	1425	33607
SAL_BA5142AA	PNUSAS000272	SRR2015068	2015	3	North America	United States	Enteritidis	Enteritidis	1425	33607
SAL_BA5144AA	PNUSAS000270	SRR2015067	2015	3	North America	United States	Enteritidis	Enteritidis	1425	33606
SAL_BA5146AA	PNUSAS000269	SRR2015066	2015	3	North America	United States	Enteritidis	Enteritidis	1425	33604
SAL_BA5147AA	PNUSAS000268	SRR2015065	2015	3	North America	United States	Enteritidis	Enteritidis	1425	33603
SAL_BA5149AA	PNUSAS000267	SRR2015064	2015	3	North America	United States	Enteritidis	Enteritidis	1425	33602

SAL_BA5156AA	PNUSAS000233	SRR2015060	2015	3	North America	United States	Enteritidis	Enteritidis	28617	33597
SAL_BA5177AA	1020983-sc-2012-11-09T12:2	ERR235380					Enteritidis (Predicted)	Enteritidis	1424	33574
SAL_BA5179AA	34173-sc-2012-11-09T12:24:	ERR235320					Enteritidis (Predicted)	Enteritidis	3776	33579
SAL_BA5180AA	34083-sc-2012-11-09T12:24:	ERR235319					Enteritidis (Predicted)	Enteritidis	3776	33186
SAL_BA5181AA	33993-sc-2012-11-09T12:24:	ERR235318					Enteritidis (Predicted)	Enteritidis	3776	33184
SAL_BA5182AA	33943-sc-2012-11-09T12:24:	ERR235317					Enteritidis (Predicted)	Enteritidis	3776	33578
SAL_BA5183AA	33923-sc-2012-11-09T12:24:	ERR235316					Enteritidis (Predicted)	Enteritidis	3776	33577
SAL_BA5184AA	33913-sc-2012-11-09T12:24:	ERR235315					Enteritidis (Predicted)	Enteritidis	3776	33191
SAL_BA5185AA	33893-sc-2012-11-09T12:24:	ERR235314					Enteritidis (Predicted)	Enteritidis	3776	33191
SAL_BA5186AA	33873-sc-2012-11-09T12:24:	ERR235313					Enteritidis (Predicted)	Enteritidis	3776	33191
SAL_BA5187AA	33713-sc-2012-11-09T12:24:	ERR235312					Enteritidis (Predicted)	Enteritidis	3776	33576
SAL_BA5188AA	33703-sc-2012-11-09T12:24:	ERR235311					Enteritidis (Predicted)	Enteritidis	3776	33575
SAL_BA5189AA	33693-sc-2012-11-09T12:24:	ERR235310					Enteritidis (Predicted)	Enteritidis	3776	33184
SAL_BA5191AA	A13216-sc-2012-11-09T12:2	ERR235140					Enteritidis (Predicted)	Enteritidis	1424	33574
SAL_BA5192AA	D3421-sc-2012-11-09T12:21	ERR235139					Enteritidis (Predicted)	Enteritidis	1424	33573
SAL_BA5193AA	A46872-sc-2012-11-09T12:2	ERR235138					Enteritidis (Predicted)	Enteritidis	1425	33572
SAL_BA5196AA	A49455-sc-2012-11-09T12:2	ERR235135					Enteritidis (Predicted)	Enteritidis	1425	33569
SAL_BA5198AA	D7378-sc-2012-11-09T12:21	ERR235133					Enteritidis (Predicted)	Enteritidis	1424	33264
SAL_BA5199AA	A6379-sc-2012-11-09T12:21	ERR235132					Enteritidis (Predicted)	Enteritidis	1424	33200
SAL_BA5200AA	548-sc-2012-11-09T12:21:12	ERR235131					Enteritidis (Predicted)	Enteritidis	1425	33257
SAL_BA5202AA	D3419-sc-2012-11-09T12:21	ERR235129					Enteritidis (Predicted)	Enteritidis	1424	33567
SAL_BA5215AA	33673-sc-2012-11-09T12:24:	ERR235308					Enteritidis (Predicted)	Enteritidis	3776	33557
SAL_BA5216AA	33663-sc-2012-11-09T12:24:	ERR235307					Enteritidis (Predicted)	Enteritidis	3776	33556
SAL_BA5217AA	33653-sc-2012-11-09T12:24:	ERR235306					Enteritidis (Predicted)	Enteritidis	3776	33184
SAL_BA5218AA	33623-sc-2012-11-09T12:24:	ERR235305					Enteritidis (Predicted)	Enteritidis	3776	33540
SAL_BA5219AA	33523-sc-2012-11-09T12:24:	ERR235304					Enteritidis (Predicted)	Enteritidis	3776	33184
SAL_BA5220AA	33513-sc-2012-11-09T12:23:	ERR235303					Enteritidis (Predicted)	Enteritidis	3776	33191
SAL_BA5221AA	33453-sc-2012-11-09T12:23:	ERR235302					Enteritidis (Predicted)	Enteritidis	3776	33184
SAL_BA5222AA	33393-sc-2012-11-09T12:23:	ERR235301					Enteritidis (Predicted)	Enteritidis	3776	33555
SAL_BA5223AA	33078-sc-2012-11-09T12:23:	ERR235300					Enteritidis (Predicted)	Enteritidis	3776	33554
SAL_BA5224AA	33033-sc-2012-11-09T12:23:	ERR235299					Enteritidis (Predicted)	Enteritidis	3776	33186
SAL_BA5225AA	33013-sc-2012-11-09T12:23:	ERR235298					Enteritidis (Predicted)	Enteritidis	3776	33553
SAL_BA5226AA	32853-sc-2012-11-09T12:23:	ERR235297					Enteritidis (Predicted)	Enteritidis	3776	33507
SAL_BA5227AA	A30023-sc-2012-11-09T12:2	ERR235128					Enteritidis (Predicted)	Enteritidis	1425	33552
SAL_BA5228AA	A28666-sc-2012-11-09T12:2	ERR235127					Enteritidis (Predicted)	Enteritidis	1425	33551
SAL_BA5229AA	A58824-sc-2012-11-09T12:2	ERR235126					Enteritidis (Predicted)	Enteritidis	1424	41355
SAL_BA5230AA	A51300-sc-2012-11-09T12:2	ERR235125					Enteritidis (Predicted)	Enteritidis	1425	33550
SAL_BA5232AA	A7830-sc-2012-11-09T12:21	ERR235123					Enteritidis (Predicted)	Enteritidis	1424	33291
SAL_BA5235AA	D6437-sc-2012-11-09T12:21	ERR235120					Enteritidis (Predicted)	Enteritidis	1424	33548
SAL_BA5236AA	D5293-sc-2012-11-09T12:21	ERR235119					Enteritidis (Predicted)	Enteritidis	1424	33547
SAL_BA5242AA	35713-sc-2012-11-09T12:24:	ERR235362					Enteritidis (Predicted)	Enteritidis	3776	33541
SAL_BA5243AA	35723-sc-2012-11-09T12:24:	ERR235361					Enteritidis (Predicted)	Enteritidis	3776	33540
SAL_BA5244AA	35773-sc-2012-11-09T12:24:	ERR235360					Enteritidis (Predicted)	Enteritidis	3776	33184
SAL_BA5245AA	35693-sc-2012-11-09T12:24:	ERR235359					Enteritidis (Predicted)	Enteritidis	3776	33539
SAL_BA5246AA	36103-sc-2012-11-09T12:24:	ERR235358					Enteritidis (Predicted)	Enteritidis	3776	33538
SAL_BA5247AA	36163-sc-2012-11-09T12:24:	ERR235357					Enteritidis (Predicted)	Enteritidis	3776	33537
SAL_BA5248AA	36173-sc-2012-11-09T12:24:	ERR235356					Enteritidis (Predicted)	Enteritidis	3776	33525
SAL_BA5249AA	36183-sc-2012-11-09T12:24:	ERR235355					Enteritidis (Predicted)	Enteritidis	3776	33184
SAL_BA5250AA	32863-sc-2012-11-09T12:23:	ERR235296					Enteritidis (Predicted)	Enteritidis	3776	33536
SAL_BA5251AA	32633-sc-2012-11-09T12:23:	ERR235295					Enteritidis (Predicted)	Enteritidis	3776	33194
SAL_BA5252AA	32523-sc-2012-11-09T12:23:	ERR235294					Enteritidis (Predicted)	Enteritidis	3776	33535
SAL_BA5253AA	32963-sc-2012-11-09T12:23:	ERR235293					Enteritidis (Predicted)	Enteritidis	3776	33534
SAL_BA5254AA	32013-sc-2012-11-09T12:23:	ERR235292					Enteritidis (Predicted)	Enteritidis	3776	33533
SAL_BA5255AA	32363-sc-2012-11-09T12:23:	ERR235291					Enteritidis (Predicted)	Enteritidis	3776	33196
SAL_BA5256AA	31353-sc-2012-11-09T12:23:	ERR235290					Enteritidis (Predicted)	Enteritidis	3776	33191
SAL_BA5262AA	36313-sc-2012-11-09T12:24:	ERR235354					Enteritidis (Predicted)	Enteritidis	3776	33529
SAL_BA5263AA	36343-sc-2012-11-09T12:24:	ERR235353					Enteritidis (Predicted)	Enteritidis	3776	33528
SAL_BA5264AA	36723-sc-2012-11-09T12:24:	ERR235352					Enteritidis (Predicted)	Enteritidis	3776	33507
SAL_BA5265AA	36803-sc-2012-11-09T12:24:	ERR235351					Enteritidis (Predicted)	Enteritidis	3776	33527

SAL_BA5266AA	35563-sc-2012-11-09T12:24:	ERR235350					Enteritidis (Predicted)	Enteritidis	3776	33196
SAL_BA5267AA	35473-sc-2012-11-09T12:24:	ERR235349					Enteritidis (Predicted)	Enteritidis	3776	33526
SAL_BA5268AA	35443-sc-2012-11-09T12:24:	ERR235348					Enteritidis (Predicted)	Enteritidis	3776	33525
SAL_BA5269AA	35433-sc-2012-11-09T12:24:	ERR235347					Enteritidis (Predicted)	Enteritidis	3776	33191
SAL_BA5270AA	35263-sc-2012-11-09T12:24:	ERR235346					Enteritidis (Predicted)	Enteritidis	3776	33524
SAL_BA5271AA	35223-sc-2012-11-09T12:24:	ERR235345					Enteritidis (Predicted)	Enteritidis	3776	33523
SAL_BA5272AA	35213-sc-2012-11-09T12:24:	ERR235344					Enteritidis (Predicted)	Enteritidis	3776	33523
SAL_BA5273AA	35203-sc-2012-11-09T12:24:	ERR235343					Enteritidis (Predicted)	Enteritidis	3776	33522
SAL_BA5287AA	34933-sc-2012-11-09T12:24:	ERR235341					Enteritidis (Predicted)	Enteritidis	3776	33510
SAL_BA5288AA	34893-sc-2012-11-09T12:24:	ERR235340					Enteritidis (Predicted)	Enteritidis	3776	33191
SAL_BA5289AA	34873-sc-2012-11-09T12:24:	ERR235339					Enteritidis (Predicted)	Enteritidis	3776	33509
SAL_BA5290AA	34863-sc-2012-11-09T12:24:	ERR235338					Enteritidis (Predicted)	Enteritidis	3776	33191
SAL_BA5291AA	34853-sc-2012-11-09T12:24:	ERR235337					Enteritidis (Predicted)	Enteritidis	3776	33184
SAL_BA5292AA	34683-sc-2012-11-09T12:24:	ERR235336					Enteritidis (Predicted)	Enteritidis	3776	33189
SAL_BA5293AA	34563-sc-2012-11-09T12:24:	ERR235335					Enteritidis (Predicted)	Enteritidis	3776	33508
SAL_BA5294AA	34503-sc-2012-11-09T12:24:	ERR235334					Enteritidis (Predicted)	Enteritidis	3776	33507
SAL_BA5302AA	A53420-sc-2012-11-09T12:2	ERR235265					Enteritidis (Predicted)	Enteritidis	1424	33500
SAL_BA5312AA	PNUSAS000211	SRR2015054	2015	2	North America	United States	Enteritidis	Enteritidis	1425	1808
SAL_BA5314AA	PNUSAS000210	SRR2015053	2015	2	North America	United States	Enteritidis	Enteritidis	1425	33492
SAL_BA5316AA	PNUSAS000209	SRR2015052	2015	1	North America	United States	Enteritidis	Enteritidis	1425	1808
SAL_BA5318AA	PNUSAS000203	SRR2015051	2014	12	North America	United States	Enteritidis	Enteritidis	1425	2668
SAL_BA5391AA	3493-sc-2012-11-09T12:20:5	ERR235117					Enteritidis (Predicted)	Enteritidis	3745	33435
SAL_BA5393AA	A32229-sc-2012-11-09T12:2	ERR235116					Enteritidis (Predicted)	Enteritidis	1425	33433
SAL_BA5395AA	A55767-sc-2012-11-09T12:2	ERR235115					Enteritidis (Predicted)	Enteritidis	1424	33284
SAL_BA5397AA	A54341-sc-2012-11-09T12:2	ERR235114					Enteritidis (Predicted)	Enteritidis	1424	33283
SAL_BA5400AA	D6007-sc-2012-11-09T12:20	ERR235113					Enteritidis (Predicted)	Enteritidis	1424	33428
SAL_BA5402AA	A17787-sc-2012-11-09T12:2	ERR235112					Enteritidis (Predicted)	Enteritidis	1424	33427
SAL_BA5404AA	253-sc-2012-11-09T12:20:53	ERR235111					Enteritidis (Predicted)	Enteritidis	1425	33236
SAL_BA5406AA	D7419-sc-2012-11-09T12:20	ERR235110					Enteritidis (Predicted)	Enteritidis	1424	33424
SAL_BA5408AA	A34142-sc-2012-11-09T12:2	ERR235109					Enteritidis (Predicted)	Enteritidis	1424	33422
SAL_BA5410AA	A40882-sc-2012-11-09T12:2	ERR235108					Enteritidis (Predicted)	Enteritidis	1425	33421
SAL_BA5412AA	A53601-sc-2012-11-09T12:2	ERR235107					Enteritidis (Predicted)	Enteritidis	1424	33419
SAL_BA5414AA	A28721-sc-2012-11-09T12:2	ERR235106					Enteritidis (Predicted)	Enteritidis	1424	33418
SAL_BA5483AA	15MN00142	SRR2005756	2015	4	North America	United States	Enteritidis	Enteritidis	27892	5650
SAL_BA5523AA	FDA00001081	SRR2002724	2007	2	Europe	Hungary	Enteritidis	Enteritidis	1425	33330
SAL_BA5549AA	A39959-sc-2012-11-09T12:2	ERR235105					Enteritidis (Predicted)	Enteritidis	1424	33266
SAL_BA5552AA	WAPHL_SAL-A00766	SRR2002487	2006		North America	United States	Enteritidis	Enteritidis	1425	33308
SAL_BA5556AA	101-sc-2012-11-09T12:20:43	ERR235102					Enteritidis (Predicted)	Enteritidis	3858	33305
SAL_BA5566AA	1971-sc-2012-11-09T12:22:1	ERR235195					Enteritidis (Predicted)	Enteritidis	1425	33295
SAL_BA5568AA	A41750-sc-2012-11-09T12:2	ERR235194					Enteritidis (Predicted)	Enteritidis	1425	33293
SAL_BA5570AA	A35261-sc-2012-11-09T12:2	ERR235193					Enteritidis (Predicted)	Enteritidis	1424	33291
SAL_BA5572AA	A45427-sc-2012-11-09T12:2	ERR235192					Enteritidis (Predicted)	Enteritidis	1425	33289
SAL_BA5574AA	A36950-sc-2012-11-09T12:2	ERR235191					Enteritidis (Predicted)	Enteritidis	1424	33287
SAL_BA5576AA	A53843-sc-2012-11-09T12:2	ERR235190					Enteritidis (Predicted)	Enteritidis	1424	33251
SAL_BA5578AA	A7828-sc-2012-11-09T12:22	ERR235189					Enteritidis (Predicted)	Enteritidis	1424	33284
SAL_BA5579AA	D17739-sc-2012-11-09T12:2	ERR235188					Enteritidis (Predicted)	Enteritidis	1424	33283
SAL_BA5581AA	8323-sc-2012-11-09T12:22:0	ERR235187					Enteritidis (Predicted)	Enteritidis	3745	33281
SAL_BA5583AA	A53742-sc-2012-11-09T12:2	ERR235186					Enteritidis (Predicted)	Enteritidis	1424	33279
SAL_BA5587AA	A36678-sc-2012-11-09T12:2	ERR235184					Enteritidis (Predicted)	Enteritidis	1425	33275
SAL_BA5589AA	A53811-sc-2012-11-09T12:2	ERR235183					Enteritidis (Predicted)	Enteritidis	1424	33273
SAL_BA5594AA	A51073-sc-2012-11-09T12:2	ERR235181					Enteritidis (Predicted)	Enteritidis	1425	33257
SAL_BA5596AA	9486-sc-2012-11-09T12:21:5	ERR235180					Enteritidis (Predicted)	Enteritidis	3858	33269
SAL_BA5601AA	D16245-sc-2012-11-09T12:2	ERR235178					Enteritidis (Predicted)	Enteritidis	1424	33266
SAL_BA5603AA	A31878-sc-2012-11-09T12:2	ERR235177					Enteritidis (Predicted)	Enteritidis	1424	33264
SAL_BA5605AA	A46277-sc-2012-11-09T12:2	ERR235176					Enteritidis (Predicted)	Enteritidis	1424	33262
SAL_BA5607AA	A36950-sc-2012-11-09T12:2	ERR235175					Enteritidis (Predicted)	Enteritidis	1425	33260
SAL_BA5609AA	D54684-sc-2012-11-09T12:2	ERR235174					Enteritidis (Predicted)	Enteritidis	1425	33259
SAL_BA5612AA	A54059-sc-2012-11-09T12:2	ERR235173					Enteritidis (Predicted)	Enteritidis	1425	33257
SAL_BA5616AA	7909-sc-2012-11-09T12:21:4	ERR235171					Enteritidis (Predicted)	Enteritidis	1425	33253

SAL_BA5618AA	D11119-sc-2012-11-09T12:2	ERR235170					Enteritidis (Predicted)	Enteritidis	1424	33251
SAL_BA5620AA	A9215-sc-2012-11-09T12:21	ERR235169					Enteritidis (Predicted)	Enteritidis	1424	33249
SAL_BA5622AA	A31243-sc-2012-11-09T12:2	ERR235168					Enteritidis (Predicted)	Enteritidis	1425	33247
SAL_BA5624AA	A53016-sc-2012-11-09T12:2	ERR235167					Enteritidis (Predicted)	Enteritidis	1424	33245
SAL_BA5626AA	A56756-sc-2012-11-09T12:2	ERR235166					Enteritidis (Predicted)	Enteritidis	1425	33243
SAL_BA5629AA	A54595-sc-2012-11-09T12:2	ERR235165					Enteritidis (Predicted)	Enteritidis	1425	33240
SAL_BA5633AA	A51583-sc-2012-11-09T12:2	ERR235163					Enteritidis (Predicted)	Enteritidis	1425	33236
SAL_BA5636AA	6567-sc-2012-11-09T12:21:4	ERR235161					Enteritidis (Predicted)	Enteritidis	3815	33233
SAL_BA5638AA	A12683-sc-2012-11-09T12:2	ERR235160					Enteritidis (Predicted)	Enteritidis	1425	33232
SAL_BA5640AA	D3558-sc-2012-11-09T12:21	ERR235159					Enteritidis (Predicted)	Enteritidis	1424	33231
SAL_BA5642AA	A30782-sc-2012-11-09T12:2	ERR235158					Enteritidis (Predicted)	Enteritidis	1424	41495
SAL_BA5645AA	15MN00141	SRR1998979	2015	4	North America	United States	Enteritidis	Enteritidis	1425	33228
SAL_BA5646AA	A57601-sc-2012-11-09T12:2	ERR235156					Enteritidis (Predicted)	Enteritidis	1425	41336
SAL_BA5648AA	D43980-sc-2012-11-09T12:2	ERR235155					Enteritidis (Predicted)	Enteritidis	1424	33226
SAL_BA5650AA	A59040-sc-2012-11-09T12:2	ERR235154					Enteritidis (Predicted)	Enteritidis	1424	33220
SAL_BA5651AA	15MN00140	SRR1998974	2015	4	North America	United States	Enteritidis	Enteritidis	1425	33224
SAL_BA5654AA	34493-sc-2012-11-09T12:24:	ERR235333					Enteritidis (Predicted)	Enteritidis	3776	33222
SAL_BA5656AA	A9086-sc-2012-11-09T12:21	ERR235152					Enteritidis (Predicted)	Enteritidis	1424	33220
SAL_BA5658AA	3857-sc-2012-11-09T12:21:3	ERR235151					Enteritidis (Predicted)	Enteritidis	1425	33218
SAL_BA5659AA	CFSAN032481	SRR1998969	2006	5	South America	Argentina	Enteritidis	Enteritidis	1425	33217
SAL_BA5660AA	A12756-sc-2012-11-09T12:2	ERR235150					Enteritidis (Predicted)	Enteritidis	1424	33216
SAL_BA5662AA	A17698-sc-2012-11-09T12:2	ERR235149					Enteritidis (Predicted)	Enteritidis	1424	33214
SAL_BA5663AA	CFSAN032485	SRR1998964	2006	1	South America	Argentina	Enteritidis	Enteritidis	1425	33213
SAL_BA5664AA	A46756-sc-2012-11-09T12:2	ERR235148					Enteritidis (Predicted)	Enteritidis	1425	33212
SAL_BA5665AA	15MN00133	SRR1998961	2015	4	North America	United States	Enteritidis	Enteritidis	1425	33211
SAL_BA5666AA	A34057-sc-2012-11-09T12:2	ERR235147					Enteritidis (Predicted)	Enteritidis	1425	33210
SAL_BA5668AA	A59024-sc-2012-11-09T12:2	ERR235146					Enteritidis (Predicted)	Enteritidis	1425	33208
SAL_BA5669AA	15MN00139	SRR1998948	2015	4	North America	United States	Enteritidis	Enteritidis	28627	33207
SAL_BA5670AA	A48460-sc-2012-11-09T12:2	ERR235145					Enteritidis (Predicted)	Enteritidis	1425	33206
SAL_BA5671AA	CFSAN032487	SRR1998947	2008	4	South America	Argentina	Enteritidis	Enteritidis	1425	33205
SAL_BA5673AA	CFSAN032486	SRR1998946	2009	2	South America	Argentina	Enteritidis	Enteritidis	1425	33203
SAL_BA5675AA	CFSAN032482	SRR1998945	2006	5	South America	Argentina	Enteritidis	Enteritidis	1425	33201
SAL_BA5676AA	A7369-sc-2012-11-09T12:21	ERR235142					Enteritidis (Predicted)	Enteritidis	1424	33200
SAL_BA5677AA	15MN00138	SRR1998943	2015	4	North America	United States	Enteritidis	Enteritidis	1425	33199
SAL_BA5678AA	2702-sc-2012-11-09T12:21:2	ERR235141					Enteritidis (Predicted)	Enteritidis	3858	33198
SAL_BA5679AA	CFSAN032488	SRR1998938	2008	4	South America	Argentina	Enteritidis	Enteritidis	1425	33197
SAL_BA5680AA	34483-sc-2012-11-09T12:24:	ERR235332					Enteritidis (Predicted)	Enteritidis	3776	33196
SAL_BA5681AA	CFSAN030852	SRR1997833	2012		North America	United States	Enteritidis	Enteritidis	1425	33195
SAL_BA5683AA	CFSAN030835	SRR1997832	2012		North America	United States	Enteritidis	Enteritidis	1425	33187
SAL_BA5684AA	34443-sc-2012-11-09T12:24:	ERR235330					Enteritidis (Predicted)	Enteritidis	3776	33194
SAL_BA5686AA	34423-sc-2012-11-09T12:24:	ERR235329					Enteritidis (Predicted)	Enteritidis	3776	33193
SAL_BA5688AA	34413-sc-2012-11-09T12:24:	ERR235328					Enteritidis (Predicted)	Enteritidis	3776	33191
SAL_BA5690AA	34403-sc-2012-11-09T12:24:	ERR235327					Enteritidis (Predicted)	Enteritidis	3776	33184
SAL_BA5692AA	34393-sc-2012-11-09T12:24:	ERR235326					Enteritidis (Predicted)	Enteritidis	3776	33189
SAL_BA5694AA	34253-sc-2012-11-09T12:24:	ERR235325					Enteritidis (Predicted)	Enteritidis	3776	33181
SAL_BA5695AA	CFSAN030839	SRR1997826	2012		North America	United States	Enteritidis	Enteritidis	1425	33187
SAL_BA5696AA	34243-sc-2012-11-09T12:24:	ERR235324					Enteritidis (Predicted)	Enteritidis	3776	33186
SAL_BA5697AA	CFSAN030827	SRR1997825	2012		North America	United States	Enteritidis	Enteritidis	1425	33185
SAL_BA5698AA	34233-sc-2012-11-09T12:24:	ERR235323					Enteritidis (Predicted)	Enteritidis	3776	33184
SAL_BA5699AA	CFSAN030823	SRR1997824	2012		North America	United States	Enteritidis	Enteritidis	1425	33183
SAL_BA5700AA	CFSAN030818	SRR1997823	2012		North America	United States	Enteritidis	Enteritidis	28629	33182
SAL_BA5701AA	34223-sc-2012-11-09T12:24:	ERR235322					Enteritidis (Predicted)	Enteritidis	3776	33181
SAL_BA5702AA	CFSAN030816	SRR1997822	2012		North America	United States	Enteritidis	Enteritidis	1425	33180
SAL_BA5703AA	34193-sc-2012-11-09T12:24:	ERR235321					Enteritidis (Predicted)	Enteritidis	3776	33179
SAL_BA5704AA	CFSAN030809	SRR1997821	2012		North America	United States	Enteritidis	Enteritidis	1425	33178
SAL_BA5733AA	IEH-NGS-SAL-02584	SRR1997806	2014	4	North America	United States	Enteritidis (Predicted)	Enteritidis	1425	33150
SAL_BA5735AA	CFSAN031129	SRR1997805	2014	4	North America	United States	Enteritidis (Predicted)	Enteritidis	1425	33148
SAL_BA5741AA	CFSAN031153	SRR1997802	2014	4	North America	United States	Enteritidis (Predicted)	Enteritidis	1425	33142
SAL_BA5751AA	IEH-NGS-SAL-02574	SRR1997797	2014	4	North America	United States	Enteritidis (Predicted)	Enteritidis	1425	33135

SAL_BA5767AA	M273 / NCTC20088	ERR230453	1938	9	7			Moscow	Enteritidis	3842	33119
SAL_BA5778AA	CFSAN028988	SRR1996245	2014	4		North America	United States	Enteritidis (Predicted)	Enteritidis	1425	33109
SAL_BA5801AA	CFSAN028955	SRR1996232	2014	4		North America	United States	Enteritidis (Predicted)	Enteritidis	27901	33086
SAL_BA5811AA	CFSAN028927	SRR1996227	2014	4		North America	United States	Enteritidis (Predicted)	Enteritidis	1425	33076
SAL_BA5815AA	CFSAN031112	SRR1996224	2014	4		North America	United States	Enteritidis (Predicted)	Enteritidis	1425	33072
SAL_BA5823AA	CFSAN028963	SRR1996219	2014	4		North America	United States	Enteritidis (Predicted)	Enteritidis	1425	33066
SAL_BA5827AA	CFSAN028983	SRR1996217	2014	4		North America	United States	Enteritidis (Predicted)	Enteritidis	1425	33062
SAL_BA5833AA	IEH-NGS-SAL-02110	SRR1996214	2014	4		North America	United States	Enteritidis (Predicted)	Enteritidis	1425	33056
SAL_BA5835AA	CFSAN028971	SRR1996213	2014	4		North America	United States	Enteritidis (Predicted)	Enteritidis	1425	33054
SAL_BA5843AA	IEH-NGS-SAL-02581	SRR1996209	2014	4		North America	United States	Enteritidis (Predicted)	Enteritidis	1425	33046
SAL_BA5867AA	CFSAN028985	SRR1996194	2014	4		North America	United States	Enteritidis (Predicted)	Enteritidis	1425	33024
SAL_BA5876AA	IEH-NGS-SAL-02119	SRR1996189	2014	4		North America	United States	Enteritidis (Predicted)	Enteritidis	1425	33015
SAL_BA5880AA	M2 / NCTC20002	ERR230374	1925	8	4			Enteritidis	Enteritidis	1425	33011
SAL_BA5885AA	FDA00001126	SRR1996185	2007	6		Asia	China	Enteritidis	Enteritidis	1425	33006
SAL_BA5899AA	IEH-NGS-SAL-02075	SRR1996178	2014	4		North America	United States	Enteritidis (Predicted)	Enteritidis	1425	32998
SAL_BA5922AA	CFSAN028970	SRR1996167	2014	4		North America	United States	Enteritidis (Predicted)	Enteritidis	1425	32986
SAL_BA5960AA	CFSAN028992	SRR1996145	2014	4		North America	United States	Enteritidis (Predicted)	Enteritidis	1425	32967
SAL_BA5962AA	CFSAN028997	SRR1996144	2014	4		North America	United States	Enteritidis (Predicted)	Enteritidis	1425	32966
SAL_BA5982AA	CFSAN029002	SRR1996134	2014	4		North America	United States	Enteritidis (Predicted)	Enteritidis	1425	32957
SAL_BA6014AA	IEH-NGS-SAL-02135	SRR1995741	2014	4		North America	United States	Enteritidis (Predicted)	Enteritidis	27904	32942
SAL_BA6022AA	IEH-NGS-SAL-02116	SRR1995737	2014	4		North America	United States	Enteritidis (Predicted)	Enteritidis	1425	32938
SAL_BA6024AA	CFSAN031148	SRR1995564	2014	4		North America	United States	Enteritidis (Predicted)	Enteritidis	1425	32937
SAL_BA6034AA	CFSAN029004	SRR1995558	2014	4		North America	United States	Enteritidis (Predicted)	Enteritidis	1425	32932
SAL_BA6066AA	IEH-NGS-SAL-02593	SRR1995539	2014	4		North America	United States	Enteritidis (Predicted)	Enteritidis	1425	32917
SAL_BA6068AA	IEH-NGS-SAL-02113	SRR1995538	2014	4		North America	United States	Enteritidis (Predicted)	Enteritidis	1425	32916
SAL_BA6092AA	CFSAN028979	SRR1995525	2014	4		North America	United States	Enteritidis (Predicted)	Enteritidis	1425	32906
SAL_BA6098AA	CFSAN028986	SRR1995522	2014	4		North America	United States	Enteritidis (Predicted)	Enteritidis	1425	32903
SAL_BA6100AA	CFSAN031147	SRR1995521	2014	4		North America	United States	Enteritidis (Predicted)	Enteritidis	1425	32901
SAL_BA6112AA	CFSAN028962	SRR1995515	2014	4		North America	United States	Enteritidis (Predicted)	Enteritidis	1425	32892
SAL_BA6116AA	CFSAN028982	SRR1995513	2014	4		North America	United States	Enteritidis (Predicted)	Enteritidis	1425	32889
SAL_BA6126AA	CFSAN028999	SRR1995508	2014	4		North America	United States	Enteritidis (Predicted)	Enteritidis	1425	32880
SAL_BA6130AA	CFSAN028952	SRR1995506	2014	4		North America	United States	Enteritidis (Predicted)	Enteritidis	1425	32878
SAL_BA6133AA	CFSAN031120	SRR1995504	2014	4		North America	Canada	Enteritidis (Predicted)	Enteritidis	1425	32875
SAL_BA6149AA	CFSAN028998	SRR1995496	2014	4		North America	United States	Enteritidis (Predicted)	Enteritidis	1425	32862
SAL_BA6155AA	15MN00124	SRR1988508	2015	3		North America	United States	Enteritidis	Enteritidis	1425	32858
SAL_BA6158AA	15MN00097	SRR1988507	2015	3		North America	United States	Enteritidis	Enteritidis	1425	32855
SAL_BA6166AA	15MN00125	SRR1988503	2015	3		North America	United States	Enteritidis	Enteritidis	1425	32850
SAL_BA6171AA	CFSAN031107	SRR1988500	2014	4		North America	United States	Enteritidis (Predicted)	Enteritidis	1425	32846
SAL_BA6175AA	CFSAN031093	SRR1988498	2014	4		North America	United States	Enteritidis (Predicted)	Enteritidis	1425	32842
SAL_BA6179AA	15MN00132	SRR1988496	2015	4		North America	United States	Enteritidis	Enteritidis	1425	32838
SAL_BA6181AA	15MN00131	SRR1988495	2015	4		North America	United States	Enteritidis	Enteritidis	1425	32836
SAL_BA6182AA	15MN00122	SRR1988494	2015	3		North America	United States	Enteritidis	Enteritidis	1425	32835
SAL_BA6184AA	15MN00128	SRR1988493	2015	3		North America	United States	Enteritidis	Enteritidis	1425	32833
SAL_BA6186AA	15MN00112	SRR1988492	2015	4		North America	United States	Enteritidis	Enteritidis	1425	32831
SAL_BA6188AA	15MN00108	SRR1988491	2015	3		North America	United States	Enteritidis	Enteritidis	1425	32829
SAL_BA6209AA	IEH-NGS-SAL-02561	SRR1988480	2014	4		North America	United States	Enteritidis (Predicted)	Enteritidis	1425	32809
SAL_BA6217AA	15MN00127	SRR1988476	2015	3		North America	United States	Enteritidis	Enteritidis	1425	32801
SAL_BA6226AA	CFSAN031087	SRR1988471	2014	4		North America	United States	Enteritidis (Predicted)	Enteritidis	1425	32795
SAL_BA6228AA	15MN00120	SRR1988470	2015	3		North America	United States	Enteritidis	Enteritidis	1425	32793
SAL_BA6247AA	15MN00106	SRR1988452	2015	3		North America	United States	Enteritidis	Enteritidis	1425	32777
SAL_BA6249AA	15MN00099	SRR1988450	2015	3		North America	United States	Enteritidis	Enteritidis	1425	25745
SAL_BA6266AA	CFSAN030770	SRR1982236	2014	5		North America	United States	Enteritidis (Predicted)	Enteritidis	1425	32768
SAL_BA6270AA	15MN00111	SRR1982234	2015	3		North America	United States	Enteritidis	Enteritidis	1425	32764
SAL_BA6291AA	CFSAN030753	SRR1982222	2014	5		North America	United States	Enteritidis (Predicted)	Enteritidis	1425	32749
SAL_BA6293AA	CFSAN030786	SRR1982221	2014	5		North America	United States	Enteritidis (Predicted)	Enteritidis	1425	32445
SAL_BA6305AA	IEH-NGS-SAL-02532	SRR1982212	2014	5		North America	United States	Enteritidis (Predicted)	Enteritidis	1425	32737
SAL_BA6324AA	15MN00126	SRR1982202	2015	3		North America	United States	Enteritidis	Enteritidis	1425	32723
SAL_BA6328AA	15MN00135	SRR1982197	2015	4		North America	United States	Enteritidis	Enteritidis	1425	25745
SAL_BA6344AA	15MN00088	SRR1982188	2015	2		North America	United States	Enteritidis	Enteritidis	1425	32705

SAL_BA6351AA	15MN00110	SRR1982184	2015	4	North America	United States	Enteritidis	Enteritidis	1425	32699
SAL_BA6359AA	15MN00070	SRR1982180	2015	2	North America	United States	Enteritidis	Enteritidis	1425	2668
SAL_BA6372AA	09:MF0001194R	ERR161896					Enteritidis (Predicted)	Enteritidis	27167	32685
SAL_BA6373AA	CFSAN031084	SRR1982170	2014	4	North America	United States	Enteritidis (Predicted)	Enteritidis	1425	32684
SAL_BA6374AA	CFSAN030769	SRR1982169	2014	5	North America	United States	Enteritidis (Predicted)	Enteritidis	1425	32683
SAL_BA6386AA	15MN00105	SRR1982162	2015	3	North America	United States	Enteritidis	Enteritidis	1425	32672
SAL_BA6390AA	CFSAN030777	SRR1982159	2014	5	North America	United States	Enteritidis (Predicted)	Enteritidis	1425	32668
SAL_BA6394AA	15MN00071	SRR1982157	2015	2	North America	United States	Enteritidis	Enteritidis	1425	32664
SAL_BA6396AA	15MN00107	SRR1982156	2015	3	North America	United States	Enteritidis	Enteritidis	1425	12315
SAL_BA6397AA	15MN00098	SRR1982155	2015	3	North America	United States	Enteritidis	Enteritidis	28643	32662
SAL_BA6399AA	15MN00091	SRR1982154	2015	3	North America	United States	Enteritidis	Enteritidis	1425	32660
SAL_BA6403AA	15MN00089	SRR1982152	2015	3	North America	United States	Enteritidis	Enteritidis	1425	32658
SAL_BA6408AA	15MN00121	SRR1982149	2015	3	North America	United States	Enteritidis	Enteritidis	1425	32653
SAL_BA6420AA	15MN00109	SRR1982142	2015	4	North America	United States	Enteritidis	Enteritidis	1425	32643
SAL_BA6433AA	FLUFL-1483	SRR1982134	2003	6	North America	United States	Enteritidis (Predicted)	Enteritidis	1425	32632
SAL_BA6436AA	CFSAN030752	SRR1982131	2014	5	North America	United States	Enteritidis (Predicted)	Enteritidis	1425	32630
SAL_BA6440AA	15MN00137	SRR1982128	2015	4	North America	United States	Enteritidis	Enteritidis	1425	32627
SAL_BA6449AA	15MN00092	SRR1982124	2015	3	North America	United States	Enteritidis	Enteritidis	1425	32619
SAL_BA6451AA	15MN00068	SRR1982123	2015	2	North America	United States	Enteritidis	Enteritidis	1425	32617
SAL_BA6455AA	15MN00123	SRR1982120	2015	3	North America	United States	Enteritidis	Enteritidis	1425	32614
SAL_BA6457AA	15MN00100	SRR1982119	2015	3	North America	United States	Enteritidis	Enteritidis	1425	32613
SAL_BA6463AA	CFSAN030754	SRR1982116	2014	5	North America	United States	Enteritidis (Predicted)	Enteritidis	1425	32607
SAL_BA6465AA	CFSAN030751	SRR1982115	2014	5	North America	United States	Enteritidis (Predicted)	Enteritidis	1425	32605
SAL_BA6475AA	CFSAN030790	SRR1982056	2014	5	North America	United States	Enteritidis (Predicted)	Enteritidis	1425	32599
SAL_BA6478AA	15MN00134	SRR1982053	2015	4	North America	United States	Enteritidis	Enteritidis	1425	32596
SAL_BA6482AA	CFSAN030791	SRR1982051	2014	5	North America	United States	Enteritidis (Predicted)	Enteritidis	1425	32592
SAL_BA6484AA	IEH-NGS-SAL-02531	SRR1982049	2014	5	North America	United States	Enteritidis (Predicted)	Enteritidis	1425	32590
SAL_BA6486AA	IEH-NGS-SAL-02524	SRR1982048	2014	5	North America	United States	Enteritidis (Predicted)	Enteritidis	1425	32588
SAL_BA6488AA	15MN00104	SRR1982046	2015	3	North America	United States	Enteritidis	Enteritidis	1425	32586
SAL_BA6495AA	IEH-NGS-SAL-02536	SRR1982035	2014	5	North America	United States	Enteritidis (Predicted)	Enteritidis	1425	32579
SAL_BA6501AA	15MN00136	SRR1982031	2015	4	North America	United States	Enteritidis	Enteritidis	1425	25745
SAL_BA6502AA	15MN00130	SRR1982030	2015	4	North America	United States	Enteritidis	Enteritidis	1425	25754
SAL_BA6545AA	15MN00069	SRR1980625	2015	2	North America	United States	Enteritidis	Enteritidis	1425	22169
SAL_BA6569AA	15MN00090	SRR1980609	2015	2	North America	United States	Enteritidis	Enteritidis	1425	32517
SAL_BA6595AA	CFSAN030750	SRR1979268	2014	5	North America	United States	Enteritidis (Predicted)	Enteritidis	1425	32493
SAL_BA6616AA	CFSAN030743	SRR1976272	2014	5	North America	United States	Enteritidis (Predicted)	Enteritidis	1425	32475
SAL_BA6646AA	CFSAN030735	SRR1976254	2014	5	North America	United States	Enteritidis (Predicted)	Enteritidis	1425	32450
SAL_BA6654AA	IEH-NGS-SAL-02490	SRR1976249	2014	5	North America	United States	Enteritidis (Predicted)	Enteritidis	1425	32445
SAL_BA6656AA	IEH-NGS-SAL-02481	SRR1976248	2014	5	North America	United States	Enteritidis (Predicted)	Enteritidis	1425	32443
SAL_BA6658AA	IEH-NGS-SAL-02480	SRR1976247	2014	5	North America	United States	Enteritidis (Predicted)	Enteritidis	1425	32441
SAL_BA6662AA	IEH-NGS-SAL-02489	SRR1976241	2014	5	North America	United States	Enteritidis (Predicted)	Enteritidis	1425	32437
SAL_BA6666AA	IEH-NGS-SAL-02482	SRR1976239	2014	5	North America	United States	Enteritidis (Predicted)	Enteritidis	1425	32433
SAL_BA6676AA	09:MF0013408R	ERR160952						Enteritidis	1425	32425
SAL_BA6677AA	IEH-NGS-SAL-02477	SRR1976234	2014	5	North America	United States	Enteritidis (Predicted)	Enteritidis	1425	14319
SAL_BA6681AA	IEH-NGS-SAL-02471	SRR1976232	2014	5	North America	United States	Enteritidis (Predicted)	Enteritidis	1425	32421
SAL_BA6731AA	WAPHL_SAL-A00895	SRR1974121	2015	2	North America	United States	Enteritidis	Enteritidis	1425	32376
SAL_BA6733AA	CFSAN030268	SRR1974120	2014	4	North America	United States	Enteritidis (Predicted)	Enteritidis	1425	32374
SAL_BA6737AA	WAPHL_SAL-A00886	SRR1974114	2014	12	North America	United States	Enteritidis	Enteritidis	1425	32370
SAL_BA6771AA	WAPHL_SAL-A00733	SRR1974084	2006		North America	United States	Enteritidis	Enteritidis	1425	32340
SAL_BA6800AA	WAPHL_SAL-A00891	SRR1974026	2015	1	North America	United States	Enteritidis	Enteritidis	1425	32312
SAL_BA6897AA	CFSAN030267	SRR1973969	2014	4	North America	United States	Enteritidis (Predicted)	Enteritidis	1425	32222
SAL_BA6914AA	WAPHL_SAL-A00888	SRR1973913	2014	12	North America	United States	Enteritidis	Enteritidis	8003	32211
SAL_BA6941AA	WAPHL_SAL-A00887	SRR1973896	2014	12	North America	United States	Enteritidis	Enteritidis	1425	22715
SAL_BA6951AA	CFSAN030695	SRR1973888	2014	5	North America	United States	Enteritidis (Predicted)	Enteritidis	28670	32182
SAL_BA6959AA	IEH-NGS-SAL-02381	SRR1973883	2014	4	North America	United States	Enteritidis (Predicted)	Enteritidis	1425	32174
SAL_BA6978AA	CFSAN030276	SRR1973851	2014	4	North America	United States	Enteritidis (Predicted)	Enteritidis	1425	32157
SAL_BA6980AA	IEH-NGS-SAL-02454	SRR1973848	2014	5	North America	United States	Enteritidis (Predicted)	Enteritidis	1425	32155
SAL_BA6988AA	WAPHL_SAL-A00885	SRR1973841	2014	12	North America	United States	Enteritidis	Enteritidis	1425	32148
SAL_BA7036AA	CFSAN030292	SRR1973808	2014	4	North America	United States	Enteritidis (Predicted)	Enteritidis	1425	32111

SAL_BA7187AA	CFSAN030286	SRR1973798	2014	4		North America	United States	Enteritidis (Predicted)	Enteritidis	1425	32009
SAL_BA7196AA	IEH-NGS-SAL-02357	SRR1973792	2014	4		North America	United States	Enteritidis (Predicted)	Enteritidis	1425	32006
SAL_BA7204AA	WAPHL_SAL-A00890	SRR1973786	2015	1		North America	United States	Enteritidis	Enteritidis	1425	32003
SAL_BA7223AA	WAPHL_SAL-A00896	SRR1973776	2015	2		North America	United States	Enteritidis	Enteritidis	1425	31994
SAL_BA7241AA	IEH-NGS-SAL-02467	SRR1973761	2014	5		North America	United States	Enteritidis (Predicted)	Enteritidis	1425	31982
SAL_BA7262AA	CFSAN030279	SRR1973743	2014	4		North America	United States	Enteritidis (Predicted)	Enteritidis	1425	31970
SAL_BA7279AA	IEH-NGS-SAL-02458	SRR1973728	2014	5		North America	United States	Enteritidis (Predicted)	Enteritidis	28670	31962
SAL_BA7286AA	CFSAN030697	SRR1973717	2014	5		North America	United States	Enteritidis (Predicted)	Enteritidis	1425	31958
SAL_BA7292AA	WAPHL_SAL-A00892	SRR1973712	2015	1		North America	United States	Enteritidis	Enteritidis	1425	31956
SAL_BA7294AA	WAPHL_SAL-A00884	SRR1973711	2014	12		North America	United States	Enteritidis	Enteritidis	1425	31955
SAL_BA7301AA	CFSAN030709	SRR1973704	2014	5		North America	United States	Enteritidis (Predicted)	Enteritidis	1425	31950
SAL_BA7313AA	WAPHL_SAL-A00894	SRR1973698	2015	1		North America	United States	Enteritidis	Enteritidis	1425	31940
SAL_BA7315AA	CFSAN030295	SRR1973697	2014	4		North America	United States	Enteritidis (Predicted)	Enteritidis	1425	31938
SAL_BA7316AA	MF19031	ERR048521	2009			Europe	United Kingdom	Enteritidis	Enteritidis	1425	31937
SAL_BA7317AA	MF17654	ERR048520	2009			Europe	United Kingdom	Enteritidis	Enteritidis	1425	41328
SAL_BA7319AA	09:MF0017735R	ERR048519	2009			Europe	United Kingdom	Enteritidis	Enteritidis	1425	31935
SAL_BA7321AA	09:MF0021482R	ERR048518	2009			Europe	United Kingdom	Enteritidis	Enteritidis	1425	31934
SAL_BA7325AA	09:MF0018182R	ERR048516	2009			Europe	United Kingdom	Enteritidis	Enteritidis	1425	41327
SAL_BA7328AA	09:MF0011240R	ERR048514	2009			Europe	United Kingdom	Enteritidis	Enteritidis	1425	31931
SAL_BA7331AA	WAPHL_SAL-A00889	SRR1973686	2014	12		North America	United States	Enteritidis	Enteritidis	1425	31928
SAL_BA7332AA	09:MF0005148R	ERR048512	2009			Europe	United Kingdom	Enteritidis	Enteritidis	1425	31927
SAL_BA7334AA	09:MF0000575R	ERR048511	2009			Europe	United Kingdom	Enteritidis	Enteritidis	1425	31925
SAL_BA7340AA	08:MF0011943R	ERR048508	2008			Europe	United Kingdom	Enteritidis	Enteritidis	1425	31921
SAL_BA7351AA	05:MB7981	ERR048502	2005			Europe	United Kingdom	Enteritidis	Enteritidis	1425	31915
SAL_BA7359AA	CFSAN030294	SRR1973651	2014	4		North America	United States	Enteritidis (Predicted)	Enteritidis	1425	31910
SAL_BA7362AA	CFSAN030704	SRR1973643	2014	5		North America	United States	Enteritidis (Predicted)	Enteritidis	1425	31907
SAL_BA7393AA	PNUSAS000208	SRR1970632	2015	1		North America	United States	Enteritidis	Enteritidis	1425	31878
SAL_BA7394AA	PNUSAS000207	SRR1970631	2015	1		North America	United States	Enteritidis	Enteritidis	1425	31879
SAL_BA7395AA	PNUSAS000206	SRR1970630	2015	1		North America	United States	Enteritidis	Enteritidis	1425	31877
SAL_BA7396AA	PNUSAS000205	SRR1970629				North America	United States	Enteritidis (Predicted)	Enteritidis	1425	31876
SAL_BA7397AA	PNUSAS000204	SRR1970628				North America	United States	Enteritidis (Predicted)	Enteritidis	1425	31875
SAL_BA7398AA	PNUSAS000202	SRR1970627				North America	United States	Enteritidis (Predicted)	Enteritidis	1425	31874
SAL_BA7399AA	PNUSAS000198	SRR1970602	2015	2		North America	United States	Enteritidis	Enteritidis	1425	8621
SAL_BA7400AA	PNUSAS000197	SRR1970601	2015	2		North America	United States	Enteritidis	Enteritidis	1425	31873
SAL_BA7422AA	CFSAN030700	SRR1970578	2014	5		North America	United States	Enteritidis (Predicted)	Enteritidis	1425	31855
SAL_BA7439AA	56107	SRR1970324	2014	9		Europe	United Kingdom	Enteritidis	Enteritidis	1425	28844
SAL_BA7442AA	46581	SRR1970321	2014	7	18	Europe	United Kingdom	Enteritidis	Enteritidis	3888	31840
SAL_BA7443AA	53001	SRR1970320	2014	10		Europe	United Kingdom	Enteritidis	Enteritidis	1425	31839
SAL_BA7447AA	50850	SRR1970316	2014	9		Europe	United Kingdom	Enteritidis	Enteritidis	1425	24766
SAL_BA7461AA	38345	SRR1970302	2014	7	28	Europe	United Kingdom	Enteritidis	Enteritidis	3888	7153
SAL_BA7469AA	37824	SRR1970294	2014	7	27	Europe	United Kingdom	Enteritidis	Enteritidis	3888	2066
SAL_BA7472AA	3734	SRR1970291	2014	4		Europe	United Kingdom	Enteritidis	Enteritidis	1425	31815
SAL_BA7479AA	29391	SRR1970288	2014	7	14	Europe	United Kingdom	Enteritidis	Enteritidis	3888	31812
SAL_BA7489AA	21769	SRR1970283	2014	6		Europe	United Kingdom	Enteritidis	Enteritidis	1425	31807
SAL_BA7492AA	Salmonella_enteritidis_D247	ERR064832						Enteritidis	Enteritidis	1425	41323
SAL_BA7498AA	25000	SRR1970279	2014	7		Europe	United Kingdom	Enteritidis	Enteritidis	1425	31802
SAL_BA7499AA	38429	SRR1970278	2014	8		Europe	United Kingdom	Enteritidis	Enteritidis	1425	31801
SAL_BA7511AA	55589	SRR1970271	2014	9		Europe	United Kingdom	Enteritidis	Enteritidis	3888	31791
SAL_BA7588AA	80576	SRR1970232	2015	1		Europe	United Kingdom	Enteritidis	Enteritidis	1425	25808
SAL_BA7590AA	93574	SRR1970231	2015	3		Europe	United Kingdom	Enteritidis	Enteritidis	1425	6932
SAL_BA7598AA	12140	SRR1970227	2014	5		Europe	United Kingdom	Enteritidis	Enteritidis	1425	31755
SAL_BA7600AA	46584	SRR1970226	2014	7	17	Europe	United Kingdom	Enteritidis	Enteritidis	3888	31754
SAL_BA7602AA	25274	SRR1970225	2014	7		Europe	United Kingdom	Enteritidis	Enteritidis	1425	31753
SAL_BA7611AA	31927	SRR1970220	2014	7	22	Europe	United Kingdom	Enteritidis	Enteritidis	3888	2066
SAL_BA7620AA	9179	SRR1970216	2014	5		Europe	United Kingdom	Enteritidis	Enteritidis	1425	31744
SAL_BA7626AA	93573	SRR1970213	2015	3		Europe	United Kingdom	Enteritidis	Enteritidis	1425	31741
SAL_BA7630AA	63876	SRR1970211	2014	11		Europe	United Kingdom	Enteritidis	Enteritidis	1425	24879
SAL_BA7653AA	50862	SRR1970200	2014	9		Europe	United Kingdom	Enteritidis	Enteritidis	1425	31726
SAL_BA7675AA	57104	SRR1970189	2014	8		Europe	United Kingdom	Enteritidis	Enteritidis	3888	31713

SAL_BA7677AA	73051	SRR1970188	2014	11		Europe	United Kingdom	Enteritidis (Predicted)	Enteritidis	1425	31711
SAL_BA7685AA	63751	SRR1970184	2014	10		Europe	United Kingdom	Enteritidis	Enteritidis	1425	31583
SAL_BA7689AA	37830	SRR1970182	2014	7	30	Europe	United Kingdom	Enteritidis	Enteritidis	3888	31707
SAL_BA7695AA	34182	SRR1970179	2014	7	24	Europe	United Kingdom	Enteritidis	Enteritidis	3888	31704
SAL_BA7706AA	37116	SRR1970173	2014	8		Europe	United Kingdom	Enteritidis	Enteritidis	1425	31697
SAL_BA7725AA	31981	SRR1970163	2014	7	25	Europe	United Kingdom	Enteritidis	Enteritidis	3888	2066
SAL_BA7731AA	14896	SRR1970160	2014	5		Europe	United Kingdom	Enteritidis	Enteritidis	3888	31684
SAL_BA7737AA	91895	SRR1970157	2014	9		Europe	United Kingdom	Enteritidis	Enteritidis	3888	31681
SAL_BA7739AA	95025	SRR1970156	2015	3	11	Europe	United Kingdom	Enteritidis	Enteritidis	1425	31680
SAL_BA7752AA	62996	SRR1970150	2014	9		Europe	United Kingdom	Enteritidis	Enteritidis	1425	31676
SAL_BA7756AA	53122	SRR1970148	2014	9		Europe	United Kingdom	Enteritidis	Enteritidis	1425	25062
SAL_BA7766AA	31970	SRR1970143	2014	7		Europe	United Kingdom	Enteritidis	Enteritidis	1425	31670
SAL_BA7768AA	63923	SRR1970142	2014	11	6	Europe	United Kingdom	Enteritidis	Enteritidis	1425	31669
SAL_BA7770AA	39446	SRR1970141	2014	7	12	Europe	United Kingdom	Enteritidis	Enteritidis	3888	31668
SAL_BA7782AA	51253	SRR1970135	2014	9		Europe	United Kingdom	Enteritidis	Enteritidis	1425	31659
SAL_BA7786AA	38349	SRR1970133	2014	7	27	Europe	United Kingdom	Enteritidis	Enteritidis	3888	31658
SAL_BA7790AA	32392	SRR1970131	2014	7	25	Europe	United Kingdom	Enteritidis	Enteritidis	1425	31656
SAL_BA7792AA	61847	SRR1970130	2014	10		Europe	United Kingdom	Enteritidis	Enteritidis	3888	31655
SAL_BA7794AA	37806	SRR1970129	2014	7	30	Europe	United Kingdom	Enteritidis	Enteritidis	3888	31654
SAL_BA7798AA	89899	SRR1970127	2015	2		Europe	United Kingdom	Enteritidis	Enteritidis	1425	31651
SAL_BA7801AA	38774	SRR1970126	2014	8		Europe	United Kingdom	Enteritidis	Enteritidis	3888	31649
SAL_BA7808AA	63581	SRR1970123	2014	9		Europe	United Kingdom	Enteritidis	Enteritidis	1425	31642
SAL_BA7816AA	63737	SRR1970119	2014	10		Europe	United Kingdom	Enteritidis	Enteritidis	1425	31635
SAL_BA7824AA	61835	SRR1970115	2014	10		Europe	United Kingdom	Enteritidis	Enteritidis	1425	31629
SAL_BA7853AA	5924	SRR1970098	2014	4		Europe	United Kingdom	Enteritidis	Enteritidis	1425	31602
SAL_BA7855AA	9246	SRR1970097	2014	4		Europe	United Kingdom	Enteritidis	Enteritidis	1425	31600
SAL_BA7863AA	52994	SRR1970093	2014	10		Europe	United Kingdom	Enteritidis	Enteritidis	1425	31593
SAL_BA7873AA	68671	SRR1970088	2014	11		Europe	United Kingdom	Enteritidis	Enteritidis	27885	26868
SAL_BA7877AA	63704	SRR1970086	2014	10		Europe	United Kingdom	Enteritidis	Enteritidis	1425	31583
SAL_BA7885AA	38410	SRR1970082	2014	8	1	Europe	United Kingdom	Enteritidis	Enteritidis	3888	31578
SAL_BA7899AA	94261	SRR1970075	2015	3		Europe	United Kingdom	Enteritidis	Enteritidis	1425	2163
SAL_BA7907AA	57153	SRR1970071	2014	10		Europe	United Kingdom	Enteritidis	Enteritidis	1425	31569
SAL_BA7914AA	96919	SRR1970066	2015	3		Europe	United Kingdom	Enteritidis	Enteritidis	1425	31566
SAL_BA7920AA	39483	SRR1970062	2014	8		Europe	United Kingdom	Enteritidis	Enteritidis	1425	31564
SAL_BA7929AA	9463	SRR1970058	2014	4		Europe	United Kingdom	Enteritidis	Enteritidis	1425	31560
SAL_BA7941AA	76002	SRR1970052	2014	12		Europe	United Kingdom	Enteritidis	Enteritidis	1425	31553
SAL_BA7953AA	45589	SRR1970046	2014	9		Europe	United Kingdom	Enteritidis	Enteritidis	1425	31545
SAL_BA7957AA	25340	SRR1970044	2014	7		Europe	United Kingdom	Enteritidis	Enteritidis	1425	31541
SAL_BA7961AA	57158	SRR1970042	2014	10		Europe	United Kingdom	Enteritidis	Enteritidis	1425	31538
SAL_BA7972AA	34174	SRR1970036	2014	7		Europe	United Kingdom	Enteritidis	Enteritidis	3888	2007
SAL_BA7980AA	37107	SRR1970032	2014	8		Europe	United Kingdom	Enteritidis	Enteritidis	1425	31530
SAL_BA8003AA	23445	SRR1970021	2014	5		Europe	United Kingdom	Enteritidis	Enteritidis	26481	31519
SAL_BA8008AA	65127	SRR1970018	2014	11		Europe	United Kingdom	Enteritidis	Enteritidis	1425	24563
SAL_BA8017AA	38773	SRR1970014	2014	8	7	Europe	United Kingdom	Enteritidis	Enteritidis	3888	31511
SAL_BA8024AA	37804	SRR1970011	2014	8		Europe	United Kingdom	Enteritidis	Enteritidis	1425	31508
SAL_BA8054AA	68673	SRR1969997	2014	11		Europe	United Kingdom	Enteritidis	Enteritidis	1425	26123
SAL_BA8060AA	27796	SRR1969994	2014	7		Europe	United Kingdom	Enteritidis	Enteritidis	1425	31492
SAL_BA8068AA	39511	SRR1969990	2014	8		Europe	United Kingdom	Enteritidis	Enteritidis	1425	31487
SAL_BA8072AA	68688	SRR1969988	2014	11		Europe	United Kingdom	Enteritidis	Enteritidis	3888	2007
SAL_BA8078AA	68654	SRR1969985	2014	11		Europe	United Kingdom	Enteritidis	Enteritidis	1425	25341
SAL_BA8083AA	13400	SRR1969982	2014	5		Europe	United Kingdom	Enteritidis	Enteritidis	1425	31481
SAL_BA8086AA	91293	SRR1969981	2015	2		Europe	United Kingdom	Enteritidis	Enteritidis	1425	23956
SAL_BA8092AA	34193	SRR1969978	2014	7		Europe	United Kingdom	Enteritidis	Enteritidis	1425	31478
SAL_BA8097AA	38373	SRR1969976	2014	8		Europe	United Kingdom	Enteritidis	Enteritidis	1425	31477
SAL_BA8127AA	39487	SRR1969962	2014	8		Europe	United Kingdom	Enteritidis	Enteritidis	3888	31463
SAL_BA8133AA	63632	SRR1969959	2014	10		Europe	United Kingdom	Enteritidis	Enteritidis	1425	24879
SAL_BA8137AA	51257	SRR1969957	2014	9		Europe	United Kingdom	Enteritidis	Enteritidis	1425	27930
SAL_BA8150AA	32467	SRR1969951	2014	7	19	Europe	United Kingdom	Enteritidis	Enteritidis	3888	31456
SAL_BA8167AA	63762	SRR1969943	2014	10		Europe	United Kingdom	Enteritidis	Enteritidis	26142	31450

SAL_BA8173AA	83727	SRR1969940	2015	1		Europe	United Kingdom	Enteritidis	Enteritidis	1425	31447
SAL_BA8181AA	13385	SRR1969936	2014	5		Europe	United Kingdom	Enteritidis	Enteritidis	1425	31444
SAL_BA8183AA	12188	SRR1969935	2014	5		Europe	United Kingdom	Enteritidis	Enteritidis	1425	31443
SAL_BA8185AA	24988	SRR1969934	2014	7		Europe	United Kingdom	Enteritidis	Enteritidis	1425	31442
SAL_BA8187AA	27062	SRR1969933	2014	7		Europe	United Kingdom	Enteritidis	Enteritidis	3888	31441
SAL_BA8189AA	23507	SRR1969932	2014	6	19	Europe	United Kingdom	Enteritidis	Enteritidis	3888	31439
SAL_BA8197AA	41991	SRR1969928	2014	8	19	Europe	United Kingdom	Enteritidis	Enteritidis	3888	31431
SAL_BA8201AA	52969	SRR1969926	2014	10		Europe	United Kingdom	Enteritidis	Enteritidis	1425	31427
SAL_BA8203AA	13359	SRR1969925	2014	4		Europe	United Kingdom	Enteritidis	Enteritidis	1425	31425
SAL_BA8205AA	34204	SRR1969924	2014	7	28	Europe	United Kingdom	Enteritidis	Enteritidis	3888	31423
SAL_BA8209AA	31653	SRR1969922	2014	7		Europe	United Kingdom	Enteritidis	Enteritidis	1425	31419
SAL_BA8230AA	80604	SRR1969912	2015	1		Europe	United Kingdom	Enteritidis	Enteritidis	1425	31400
SAL_BA8241AA	AZ_TG68160	SRR953549	2009			North America	United States	Enteritidis	Enteritidis	1425	31394
SAL_BA8248AA	AZ_TG68132	SRR953545	2009			North America	United States	Enteritidis	Enteritidis	1425	31387
SAL_BA8252AA	AZ_TG68072	SRR953543	2008			North America	United States	Enteritidis	Enteritidis	1425	31385
SAL_BA8255AA	14538	SRR1969900	2014	5		Europe	United Kingdom	Enteritidis	Enteritidis	1425	31382
SAL_BA8261AA	9417	SRR1969897	2014	4		Europe	United Kingdom	Enteritidis	Enteritidis	3888	31376
SAL_BA8265AA	25225	SRR1969895	2014	7	28	Europe	United Kingdom	Enteritidis	Enteritidis	3888	31374
SAL_BA8266AA	46578	SRR1969894	2014	7		Europe	United Kingdom	Enteritidis	Enteritidis	1425	31373
SAL_BA8273AA	38430	SRR1969891	2014	8	1	Europe	Jersey	Enteritidis	Enteritidis	3888	31366
SAL_BA8283AA	9175	SRR1969886	2014	5		Europe	United Kingdom	Enteritidis	Enteritidis	1425	31358
SAL_BA8293AA	63933	SRR1969881	2014	11		Europe	United Kingdom	Enteritidis	Enteritidis	26544	31348
SAL_BA8298AA	46577	SRR1969878	2014	7		Europe	United Kingdom	Enteritidis	Enteritidis	1425	31344
SAL_BA8308AA	94097	SRR1969873	2015	3		Europe	United Kingdom	Enteritidis	Enteritidis	1425	31335
SAL_BA8310AA	94266	SRR1969872	2015	3		Europe	United Kingdom	Enteritidis	Enteritidis	1425	31334
SAL_BA8312AA	32394	SRR1969871	2014	7		Europe	United Kingdom	Enteritidis	Enteritidis	1425	31332
SAL_BA8317AA	52962	SRR1969869	2014	10		Europe	United Kingdom	Enteritidis	Enteritidis	1425	31327
SAL_BA8321AA	50859	SRR1969867	2014	9		Europe	United Kingdom	Enteritidis	Enteritidis	1425	31323
SAL_BA8330AA	3796	SRR1969862	2014	4		Europe	United Kingdom	Enteritidis	Enteritidis	3888	31315
SAL_BA8350AA	50848	SRR1969852	2014	9		Europe	United Kingdom	Enteritidis	Enteritidis	1425	31304
SAL_BA8354AA	80593	SRR1969850	2015	1		Europe	United Kingdom	Enteritidis	Enteritidis	1425	31301
SAL_BA8357AA	A376	ERR326643						Typhi	Enteritidis	1425	31278
SAL_BA8362AA	63578	SRR1969846	2014	10		Europe	United Kingdom	Enteritidis	Enteritidis	1425	31299
SAL_BA8364AA	37007	SRR1969845	2014	8	1	Europe	United Kingdom	Enteritidis	Enteritidis	3888	31298
SAL_BA8366AA	51287	SRR1969844	2014	9		Europe	United Kingdom	Enteritidis	Enteritidis	1425	24690
SAL_BA8372AA	53113	SRR1969841	2014	9	29	Europe	United Kingdom	Enteritidis	Enteritidis	28689	31296
SAL_BA8376AA	36474	SRR1969839	2014	7	21	Europe	United Kingdom	Enteritidis	Enteritidis	3888	2066
SAL_BA8378AA	37826	SRR1969838	2014	7	28	Europe	United Kingdom	Enteritidis	Enteritidis	3888	2066
SAL_BA8382AA	40790	SRR1969836	2014	8		Europe	United Kingdom	Enteritidis	Enteritidis	1425	31292
SAL_BA8387AA	83724	SRR1969834	2015	1		Europe	United Kingdom	Enteritidis	Enteritidis	1425	29703
SAL_BA8398AA	89903	SRR1969829	2015	2		Europe	United Kingdom	Enteritidis	Enteritidis	1425	31279
SAL_BA8399AA	A343	ERR326621						Typhi	Enteritidis	1425	31278
SAL_BA8417AA	13430	SRR1969819	2014	5		Europe	United Kingdom	Enteritidis	Enteritidis	1425	31265
SAL_BA8424AA	68685	SRR1969815	2014	11		Europe	United Kingdom	Enteritidis	Enteritidis	3888	25814
SAL_BA8430AA	27011	SRR1969812	2014	7		Europe	United Kingdom	Enteritidis	Enteritidis	1425	31258
SAL_BA8440AA	46592	SRR1969807	2014	7		Europe	United Kingdom	Enteritidis	Enteritidis	26142	31249
SAL_BA8444AA	37815	SRR1969805	2014	7	30	Europe	United Kingdom	Enteritidis	Enteritidis	3888	28289
SAL_BA8449AA	37829	SRR1969802	2014	7	28	Europe	United Kingdom	Enteritidis	Enteritidis	3888	28289
SAL_BA8450AA	A138	ERR326597						Typhi	Enteritidis	7802	31241
SAL_BA8462AA	63753	SRR1969795	2014	10		Europe	United Kingdom	Enteritidis	Enteritidis	1425	25859
SAL_BA8474AA	65112	SRR1969789	2014	11		Europe	United Kingdom	Enteritidis	Enteritidis	1425	31225
SAL_BA8476AA	27977	SRR1969788	2014	7		Europe	United Kingdom	Enteritidis	Enteritidis	1425	31223
SAL_BA8483AA	31905	SRR1969784	2014	7		Europe	United Kingdom	Enteritidis	Enteritidis	1425	31216
SAL_BA8493AA	94103	SRR1969779	2015	3	5	Europe	United Kingdom	Enteritidis	Enteritidis	1425	31207
SAL_BA8507AA	25271	SRR1969772	2014	7		Europe	United Kingdom	Enteritidis	Enteritidis	1425	31193
SAL_BA8526AA	36500	SRR1969763	2014	7		Europe	United Kingdom	Enteritidis	Enteritidis	3897	31178
SAL_BA8530AA	14131	SRR1969761	2014	5		Europe	United Kingdom	Enteritidis	Enteritidis	1425	31174
SAL_BA8533AA	36505	SRR1969759	2014	7		Europe	United Kingdom	Enteritidis	Enteritidis	3888	31171
SAL_BA8536AA	36516	SRR1969758	2014	7	21	Europe	United Kingdom	Enteritidis	Enteritidis	3888	27476

SAL_BA8538AA	68961	SRR1969757	2014	11		Europe	United Kingdom	Enteritidis	Enteritidis	1425	31169
SAL_BA8543AA	26665	SRR1969754	2014	7		Europe	United Kingdom	Enteritidis	Enteritidis	1425	31167
SAL_BA8545AA	31613	SRR1969753	2014	7	14	Europe	United Kingdom	Enteritidis	Enteritidis	3888	31165
SAL_BA8552AA	3739	SRR1969750	2014	4		Europe	United Kingdom	Enteritidis	Enteritidis	1425	31160
SAL_BA8565AA	57160	SRR1969744	2014	10		Europe	United Kingdom	Enteritidis	Enteritidis	1425	31151
SAL_BA8571AA	38360	SRR1969741	2014	8		Europe	United Kingdom	Enteritidis	Enteritidis	1425	31146
SAL_BA8573AA	40365	SRR1969740	2014	8		Europe	United Kingdom	Enteritidis	Enteritidis	1425	31144
SAL_BA8576AA	31612	SRR1969738	2014	7	14	Europe	United Kingdom	Enteritidis	Enteritidis	3888	31141
SAL_BA8580AA	85730	SRR1969736	2015	2		Europe	United Kingdom	Enteritidis	Enteritidis	1425	27079
SAL_BA8588AA	37799	SRR1969732	2014	8	1	Europe	United Kingdom	Enteritidis	Enteritidis	3888	31130
SAL_BA8596AA	CFSAN005656	SRR950678	2006	11		North America	United States	Enteritidis (Predicted)	Enteritidis	1425	31122
SAL_BA8599AA	39476	SRR1969727	2014	8	15	Europe	United Kingdom	Enteritidis	Enteritidis	1425	31119
SAL_BA8611AA	65329	SRR1969721	2014	9		Europe	United Kingdom	Enteritidis	Enteritidis	1425	27094
SAL_BA8613AA	12151	SRR1969720	2014	5		Europe	United Kingdom	Enteritidis	Enteritidis	1425	31108
SAL_BA8623AA	38327	SRR1969715	2014	8		Europe	United Kingdom	Enteritidis	Enteritidis	3888	31099
SAL_BA8625AA	63759	SRR1969714	2014	10		Europe	United Kingdom	Enteritidis	Enteritidis	1425	25087
SAL_BA8627AA	37035	SRR1969713	2014	8		Europe	France	Enteritidis	Enteritidis	3888	31096
SAL_BA8631AA	96955	SRR1969711	2015	3		Europe	United Kingdom	Enteritidis	Enteritidis	1425	31093
SAL_BA8636AA	32475	SRR1969708	2014	7	17	Europe	United Kingdom	Enteritidis	Enteritidis	3888	31088
SAL_BA8638AA	91268	SRR1969707	2015	2		Europe	United Kingdom	Enteritidis	Enteritidis	3888	31086
SAL_BA8661AA	45612	SRR1969696	2014	9		Europe	United Kingdom	Enteritidis	Enteritidis	1425	31064
SAL_BA8669AA	36528	SRR1969692	2014	7	28	Europe	United Kingdom	Enteritidis	Enteritidis	3888	31057
SAL_BA8674AA	9465	SRR1969689	2014	4		Europe	United Kingdom	Enteritidis	Enteritidis	1425	28464
SAL_BA8677AA	NY_IDR1300013484	SRR949508	2013	5		North America	United States	Enteritidis	Enteritidis	1425	31051
SAL_BA8678AA	NY_IDR1300012602	SRR949507	2013	4		North America	United States	Enteritidis	Enteritidis	1425	31050
SAL_BA8679AA	31940	SRR1969687	2014	7		Europe	United Kingdom	Enteritidis	Enteritidis	1425	31049
SAL_BA8680AA	NY_IDR1300011707	SRR949506	2013	4		North America	United States	Enteritidis	Enteritidis	1425	31047
SAL_BA8682AA	NY_IDR1300011220	SRR949505	2013	4		North America	United States	Enteritidis	Enteritidis	1425	31047
SAL_BA8684AA	NY_IDR1300011034	SRR949504	2013	4		North America	United States	Enteritidis	Enteritidis	1425	31045
SAL_BA8685AA	45607	SRR1969684	2014	9		Europe	United Kingdom	Enteritidis	Enteritidis	1425	31044
SAL_BA8686AA	NY_IDR1300010759	SRR949503	2013	4		North America	United States	Enteritidis	Enteritidis	1425	31043
SAL_BA8688AA	NY_IDR1300009147	SRR949502	2013	3		North America	United States	Enteritidis	Enteritidis	1425	31042
SAL_BA8690AA	NY_IDR1300008695	SRR949501	2013	3		North America	United States	Enteritidis	Enteritidis	1425	31040
SAL_BA8691AA	6462	SRR1969681	2014	4		Europe	United Kingdom	Enteritidis	Enteritidis	1425	31039
SAL_BA8693AA	NY_IDR1300008373	SRR949500	2013	3		North America	United States	Enteritidis	Enteritidis	1425	31037
SAL_BA8695AA	NY_IDR1300008146	SRR949499	2013	3		North America	United States	Enteritidis	Enteritidis	1425	31035
SAL_BA8698AA	67564	SRR1969677	2014	11		Europe	United Kingdom	Enteritidis	Enteritidis	3888	25602
SAL_BA8706AA	96958	SRR1969673	2015	3		Europe	United Kingdom	Enteritidis	Enteritidis	1425	31027
SAL_BA8710AA	31666	SRR1969671	2014	7		Europe	United Kingdom	Enteritidis	Enteritidis	3888	31024
SAL_BA8718AA	68960	SRR1969667	2014	11		Europe	United Kingdom	Enteritidis	Enteritidis	1425	413
SAL_BA8730AA	36388	SRR1969660	2014	8		Europe	United Kingdom	Enteritidis	Enteritidis	1425	31006
SAL_BA8735AA	53129	SRR1969657	2014	9	29	Europe	United Kingdom	Enteritidis	Enteritidis	1425	31001
SAL_BA8737AA	9325	SRR1969656	2014	4		Europe	United Kingdom	Enteritidis	Enteritidis	1425	30999
SAL_BA8742AA	85729	SRR1969653	2015	2		Europe	United Kingdom	Enteritidis	Enteritidis	1425	30995
SAL_BA8745AA	24057	SRR1969651	2014	6		Europe	United Kingdom	Enteritidis	Enteritidis	26434	30993
SAL_BA8786AA	NY_BAC0700002773	SRR949426	2007	3		North America	United States	Enteritidis	Enteritidis	1425	30959
SAL_BA8794AA	80558	SRR1969627	2015	1		Europe	United Kingdom	Enteritidis	Enteritidis	1425	30951
SAL_BA8802AA	53133	SRR1969623	2014	9		Europe	United Kingdom	Enteritidis	Enteritidis	1425	30944
SAL_BA8804AA	22254	SRR1969622	2014	6		Europe	United Kingdom	Enteritidis	Enteritidis	1425	30943
SAL_BA8808AA	50363	SRR1969620	2014	9		Europe	United Kingdom	Enteritidis	Enteritidis	1425	30940
SAL_BA8810AA	27064	SRR1969619	2014	7		Europe	United Kingdom	Enteritidis	Enteritidis	1425	30938
SAL_BA8811AA	NY_BAC0600005606	SRR949414	2006	7		North America	United States	Enteritidis	Enteritidis	1425	11422
SAL_BA8813AA	9285	SRR1969617	2014	5		Europe	United Kingdom	Enteritidis	Enteritidis	1425	30937
SAL_BA8822AA	96228	SRR1969613	2015	3		Europe	United Kingdom	Enteritidis	Enteritidis	3888	30930
SAL_BA8823AA	99784	SRR1969612	2015	3		Europe	United Kingdom	Enteritidis	Enteritidis	1425	2299
SAL_BA8826AA	27965	SRR1969611	2014	7		Europe	United Kingdom	Enteritidis	Enteritidis	3888	30927
SAL_BA8836AA	50359	SRR1969605	2014	9		Europe	United Kingdom	Enteritidis	Enteritidis	1425	30919
SAL_BA8844AA	31652	SRR1969601	2014	7	9	Europe	United Kingdom	Enteritidis	Enteritidis	3888	30913
SAL_BA8852AA	32460	SRR1969597	2014	7	16	Europe	United Kingdom	Enteritidis	Enteritidis	3888	30907

SAL_BA8859AA	83322	SRR1969593	2015	1		Europe	United Kingdom	Enteritidis	Enteritidis	1425	30901
SAL_BA8861AA	6446	SRR1969592	2014	4	17	Europe	United Kingdom	Enteritidis	Enteritidis	1425	30900
SAL_BA8867AA	85758	SRR1969589	2015	2	9	Europe	United Kingdom	Enteritidis	Enteritidis	1425	27533
SAL_BA8878AA	63585	SRR1969583	2014	10		Europe	United Kingdom	Enteritidis	Enteritidis	7811	24739
SAL_BA8887AA	38428	SRR1969578	2014	7	29	Europe	United Kingdom	Enteritidis	Enteritidis	3888	30879
SAL_BA8889AA	31655	SRR1969577	2014	7		Europe	United Kingdom	Enteritidis	Enteritidis	26142	30877
SAL_BA8899AA	9292	SRR1969572	2014	5		Europe	United Kingdom	Enteritidis	Enteritidis	1425	30870
SAL_BA8911AA	25215	SRR1969566	2014	7		Europe	United Kingdom	Enteritidis	Enteritidis	3888	30861
SAL_BA8947AA	41992	SRR1969548	2014	8		Europe	United Kingdom	Enteritidis	Enteritidis	1425	30827
SAL_BA8949AA	36466	SRR1969547	2014	7		Europe	United Kingdom	Enteritidis	Enteritidis	1425	30825
SAL_BA8951AA	36530	SRR1969546	2014	7	28	Europe	United Kingdom	Enteritidis	Enteritidis	3888	2066
SAL_BA8953AA	65431	SRR1969545	2014	10		Europe	United Kingdom	Enteritidis	Enteritidis	1425	30822
SAL_BA8968AA	5359	SRR1969538	2014	4		Europe	United Kingdom	Enteritidis	Enteritidis	1425	30810
SAL_BA8975AA	5356	SRR1969534	2014	4		Europe	United Kingdom	Enteritidis	Enteritidis	3888	30806
SAL_BA8977AA	56109	SRR1969533	2014	9		Europe	United Kingdom	Enteritidis	Enteritidis	1425	7550
SAL_BA9065AA	108_2012K_179-sc-2013-02-	ERR317078						Enteritidis	Enteritidis	1425	30754
SAL_BA9067AA	106_2011K_1681-sc-2013-0	ERR317076						Enteritidis	Enteritidis	1425	30753
SAL_BA9068AA	105_2011K_1654-sc-2013-0	ERR317075						Enteritidis	Enteritidis	7801	30752
SAL_BA9069AA	104_AM25928_B-sc-2013-0	ERR317074						Enteritidis	Enteritidis	1425	30751
SAL_BA9070AA	103_08_639-sc-2013-02-27T	ERR317073						Enteritidis	Enteritidis	1425	30750
SAL_BA9072AA	101_AM27283_B-sc-2013-0	ERR317071						Enteritidis	Enteritidis	1425	30748
SAL_BA9073AA	100_8_403-sc-2013-02-27T1	ERR317070						Enteritidis	Enteritidis	1425	30747
SAL_BA9074AA	99_CDC_H9556-sc-2013-02-	ERR317069						Enteritidis	Enteritidis	27569	16463
SAL_BA9076AA	S6669_03N-sc-2013-02-27T1	ERR317067						Enteritidis	Enteritidis	1425	30746
SAL_BA9077AA	S6669_03L-sc-2013-02-27T1	ERR317066						Enteritidis	Enteritidis	1425	30745
SAL_BA9078AA	S1750_06	ERR317065	2006					Enteritidis	Enteritidis	1425	30744
SAL_BA9079AA	S4726_04	ERR317064	2006			Europe	United Kingdom	Enteritidis	Enteritidis	1425	30743
SAL_BA9080AA	9508_211-sc-2013-02-27T10	ERR317063						Enteritidis	Enteritidis	1425	30742
SAL_BA9081AA	9508_195	ERR317062	1994			North America	United States	Enteritidis	Enteritidis	1425	30741
SAL_BA9082AA	EX4_PT21b	ERR317061	1996					Enteritidis	Enteritidis	1425	41044
SAL_BA9083AA	SED-sc-2013-02-27T10:26:5	ERR317060						Enteritidis	Enteritidis	1425	30739
SAL_BA9084AA	SEC-sc-2013-02-27T10:26:53	ERR317059						Enteritidis	Enteritidis	1425	30738
SAL_BA9085AA	SE A	ERR317058	1994			North America	United States	Enteritidis	Enteritidis	1425	30737
SAL_BA9087AA	RD84	ERR317056	2005			Europe	United Kingdom	Enteritidis	Enteritidis	1425	30736
SAL_BA9088AA	RD79-sc-2013-02-27T10:26:4	ERR317055						Enteritidis	Enteritidis	1425	30735
SAL_BA9089AA	RD76-sc-2013-02-27T10:26:4	ERR317054						Enteritidis	Enteritidis	1425	30734
SAL_BA9090AA	RD72-sc-2013-02-27T10:26:4	ERR317053						Enteritidis	Enteritidis	1425	30733
SAL_BA9091AA	RD57-sc-2013-02-27T10:26:4	ERR317052						Enteritidis	Enteritidis	7799	30732
SAL_BA9092AA	RD55-sc-2013-02-27T10:26:4	ERR317051						Enteritidis	Enteritidis	1425	30731
SAL_BA9093AA	RD54-sc-2013-02-27T10:26:4	ERR317050						Enteritidis	Enteritidis	7799	30730
SAL_BA9094AA	RD51-sc-2013-02-27T10:26:4	ERR317049						Enteritidis	Enteritidis	1425	30729
SAL_BA9095AA	RD50	ERR317048	2005			Europe	United Kingdom	Enteritidis	Enteritidis	1425	30728
SAL_BA9097AA	RD45-sc-2013-02-27T10:26:3	ERR317046						Enteritidis	Enteritidis	1425	30726
SAL_BA9098AA	RD46	ERR317045	2005			Europe	United Kingdom	Enteritidis	Enteritidis	1425	41043
SAL_BA9099AA	RD44-sc-2013-02-27T10:26:3	ERR317044						Enteritidis	Enteritidis	1425	30724
SAL_BA9100AA	RD42-sc-2013-02-27T10:26:3	ERR317043						Enteritidis	Enteritidis	1425	30724
SAL_BA9101AA	RD40-sc-2013-02-27T10:26:3	ERR317042						Enteritidis	Enteritidis	1425	30723
SAL_BA9102AA	RD36	ERR317041	2005			Europe	United Kingdom	Enteritidis	Enteritidis	1425	30722
SAL_BA9103AA	RD30-sc-2013-02-27T10:26:3	ERR317040						Enteritidis	Enteritidis	1425	30721
SAL_BA9104AA	RD26	ERR317039	2005			Europe	United Kingdom	Enteritidis	Enteritidis	1425	30720
SAL_BA9105AA	RD6-sc-2013-02-27T10:26:2	ERR317038						Enteritidis	Enteritidis	1425	30719
SAL_BA9106AA	RD3	ERR317037	2005			Europe	United Kingdom	Enteritidis	Enteritidis	1425	41042
SAL_BA9107AA	RD2-sc-2013-02-27T10:26:2	ERR317036						Enteritidis	Enteritidis	1425	30717
SAL_BA9109AA	P19-sc-2013-02-27T10:26:26	ERR317035						Enteritidis	Enteritidis	1425	30715
SAL_BA9111AA	P17	ERR317034	2007					Enteritidis	Enteritidis	1425	41041
SAL_BA9112AA	45610	SRR1969527	2014	9	9	Europe	United Kingdom	Enteritidis	Enteritidis	1425	30712
SAL_BA9113AA	P16-sc-2013-02-27T10:26:24	ERR317033						Enteritidis	Enteritidis	28706	30711
SAL_BA9114AA	21779	SRR1969526	2014	6	12	Europe	United Kingdom	Enteritidis	Enteritidis	3888	674
SAL_BA9115AA	P15-sc-2013-02-27T10:26:23	ERR317032						Enteritidis	Enteritidis	28706	41040

SAL_BA9117AA	P14-sc-2013-02-27T10:26:22	ERR317031						Enteritidis	Enteritidis	1425	41092
SAL_BA9118AA	P13-sc-2013-02-27T10:26:21	ERR317030						Enteritidis	Enteritidis	1425	30707
SAL_BA9120AA	P12-sc-2013-02-27T10:26:19	ERR317029						Enteritidis	Enteritidis	1425	30705
SAL_BA9122AA	P11-sc-2013-02-27T10:26:18	ERR317028						Enteritidis	Enteritidis	1425	30701
SAL_BA9124AA	P10-sc-2013-02-27T10:26:17	ERR317027						Enteritidis	Enteritidis	1425	30701
SAL_BA9126AA	P9-sc-2013-02-27T10:26:15Z	ERR317026						Enteritidis	Enteritidis	1425	30701
SAL_BA9128AA	P8-sc-2013-02-27T10:26:14Z	ERR317025						Enteritidis	Enteritidis	1425	41091
SAL_BA9130AA	P7-sc-2013-02-27T10:26:13Z	ERR317024						Enteritidis	Enteritidis	1425	41090
SAL_BA9132AA	P6-sc-2013-02-27T10:26:12Z	ERR317023						Enteritidis	Enteritidis	1425	41089
SAL_BA9133AA	23082	SRR1969517	2014	6	8	Europe	United Kingdom	Enteritidis	Enteritidis	3888	30694
SAL_BA9134AA	P5	ERR317022	2007					Enteritidis	Enteritidis	1425	41088
SAL_BA9135AA	P4-sc-2013-02-27T10:26:09Z	ERR317021						Enteritidis	Enteritidis	1425	30692
SAL_BA9136AA	65375	SRR1969516	2014	10		Europe	United Kingdom	Enteritidis	Enteritidis	1425	30691
SAL_BA9137AA	P2	ERR317020	2007					Enteritidis	Enteritidis	1425	30690
SAL_BA9138AA	39506	SRR1969515	2014	8	8	Europe	United Kingdom	Enteritidis	Enteritidis	3888	30689
SAL_BA9151AA	M204 / NCTC20341	ERR316979	1938	9	7			Enteritidis (Predicted)	Enteritidis	28710	30676
SAL_BA9162AA	M1 / NCTC20000	ERR316936	1925	8	4			Enteritidis (Predicted)	Enteritidis	1425	30665
SAL_BA9163AA	38777	SRR1969503	2014	8		Europe	United Kingdom	Enteritidis	Enteritidis	3888	30664
SAL_BA9169AA	75955	SRR1969500	2014	12		Europe	United Kingdom	Enteritidis	Enteritidis	1425	30660
SAL_BA9171AA	94095	SRR1969499	2015	3		Europe	United Kingdom	Enteritidis	Enteritidis	1425	30658
SAL_BA9181AA	68687	SRR1969494	2014	11		Europe	United Kingdom	Enteritidis	Enteritidis	1425	25685
SAL_BA9183AA	65440	SRR1969493	2014	10		Europe	United Kingdom	Enteritidis (Predicted)	Enteritidis	1425	25004
SAL_BA9189AA	72318	SRR1969490	2014	11		Europe	United Kingdom	Enteritidis	Enteritidis	1425	29320
SAL_BA9191AA	91277	SRR1969489	2015	2		Europe	United Kingdom	Enteritidis	Enteritidis	1425	30641
SAL_BA9200AA	84450	SRR1969484	2015	2		Europe	United Kingdom	Enteritidis	Enteritidis	1425	25914
SAL_BA9204AA	63916	SRR1969482	2014	11		Europe	United Kingdom	Enteritidis	Enteritidis	1425	30631
SAL_BA9226AA	25184	SRR1969471	2014	7		Europe	United Kingdom	Enteritidis	Enteritidis	1425	30610
SAL_BA9229AA	95045	SRR1969469	2015	3		Europe	United Kingdom	Enteritidis	Enteritidis	1425	30607
SAL_BA9246AA	9278	SRR1969460	2014	5		Europe	United Kingdom	Enteritidis	Enteritidis	1425	30595
SAL_BA9262AA	68698	SRR1969451	2014	11		Europe	United Kingdom	Enteritidis	Enteritidis	1425	25546
SAL_BA9266AA	75937	SRR1969449	2014	12		Europe	United Kingdom	Enteritidis	Enteritidis	1425	30580
SAL_BA9270AA	32396	SRR1969447	2014	7		Europe	United Kingdom	Enteritidis	Enteritidis	1425	30576
SAL_BA9273AA	84429	SRR1969445	2015	1		Europe	United Kingdom	Enteritidis	Enteritidis	1425	30573
SAL_BA9275AA	73126	SRR1969444	2014	12		Europe	United Kingdom	Enteritidis	Enteritidis	1425	30571
SAL_BA9277AA	50844	SRR1969443	2014	9		Europe	United Kingdom	Enteritidis	Enteritidis	1425	30569
SAL_BA9293AA	21783	SRR1969436	2014	6	5	Europe	United Kingdom	Enteritidis	Enteritidis	3888	30553
SAL_BA9303AA	53111	SRR1969431	2014	9		Europe	United Kingdom	Enteritidis	Enteritidis	1425	30545
SAL_BA9309AA	98251	SRR1969428	2015	3		Europe	United Kingdom	Enteritidis	Enteritidis	1425	30542
SAL_BA9310AA	38776	SRR1969427	2014	8		Europe	United Kingdom	Enteritidis	Enteritidis	26142	30541
SAL_BA9311AA	45620	SRR1969426	2014	9		Europe	United Kingdom	Enteritidis	Enteritidis	1425	30540
SAL_BA9313AA	86885	SRR1969424	2015	2		Europe	United Kingdom	Enteritidis	Enteritidis	1425	1434
SAL_BA9318AA	12171	SRR1969419	2014	5		Europe	United Kingdom	Enteritidis	Enteritidis	1425	30536
SAL_BA9321AA	38779	SRR1969416	2014	8	7	Europe	United Kingdom	Enteritidis	Enteritidis	3888	2066
SAL_BA9324AA	9308	SRR1969413	2014	5		Europe	United Kingdom	Enteritidis	Enteritidis	1425	30532
SAL_BA9326AA	69925	SRR1969411	2014	11		Europe	United Kingdom	Enteritidis	Enteritidis	1425	30530
SAL_BA9327AA	37033	SRR1969410	2014	8		Europe	United Kingdom	Enteritidis	Enteritidis	1425	30529
SAL_BA9334AA	27032	SRR1969403	2014	7		Europe	United Kingdom	Enteritidis	Enteritidis	1425	30522
SAL_BA9337AA	37834	SRR1969400	2014	7	31	Europe	United Kingdom	Enteritidis	Enteritidis	3888	30520
SAL_BA9338AA	38721	SRR1969399	2014	8		Europe	United Kingdom	Enteritidis	Enteritidis	1425	30519
SAL_BA9347AA	13413	SRR1969390	2014	5	16	Europe	United Kingdom	Enteritidis	Enteritidis	1425	30510
SAL_BA9349AA	39445	SRR1969388	2014	7		Europe	United Kingdom	Enteritidis	Enteritidis	1425	30508
SAL_BA9351AA	37038	SRR1969386	2014	8		Europe	France	Enteritidis	Enteritidis	3888	30506
SAL_BA9353AA	51258	SRR1969384	2014	9		Europe	United Kingdom	Enteritidis	Enteritidis	3888	30504
SAL_BA9365AA	46583	SRR1969372	2014	7	14	Europe	United Kingdom	Enteritidis	Enteritidis	3888	30496
SAL_BA9371AA	32517	SRR1969366	2014	7		Europe	United Kingdom	Enteritidis	Enteritidis	1425	30490
SAL_BA9372AA	46593	SRR1969365	2014	7	15	Europe	United Kingdom	Enteritidis	Enteritidis	3888	30489
SAL_BA9376AA	69903	SRR1969361	2014	11		Europe	United Kingdom	Enteritidis (Predicted)	Enteritidis	1425	30485
SAL_BA9378AA	25216	SRR1969359	2014	7		Europe	United Kingdom	Enteritidis	Enteritidis	1425	30483
SAL_BA9381AA	27966	SRR1969356	2014	7		Europe	United Kingdom	Enteritidis	Enteritidis	1425	30480

SAL_BA9384AA	25006	SRR1969353	2014	7		Europe	United Kingdom	Enteritidis	Enteritidis	3888	30477
SAL_BA9388AA	27771	SRR1969349	2014	6	26	Europe	United Kingdom	Enteritidis	Enteritidis	3888	30473
SAL_BA9389AA	94076	SRR1969348	2015	3		Europe	United Kingdom	Enteritidis	Enteritidis	1425	30472
SAL_BA9396AA	63882	SRR1969341	2014	11		Europe	United Kingdom	Enteritidis	Enteritidis	1425	30466
SAL_BA9397AA	26641	SRR1969340	2014	6	26	Europe	United Kingdom	Enteritidis	Enteritidis	3888	26971
SAL_BA9399AA	9185	SRR1969338	2014	5		Europe	United Kingdom	Enteritidis	Enteritidis	28716	30464
SAL_BA9400AA	51256	SRR1969337	2014	9		Europe	United Kingdom	Enteritidis	Enteritidis	3888	30463
SAL_BA9403AA	39454	SRR1969334	2014	7	28	Europe	United Kingdom	Enteritidis	Enteritidis	3888	2066
SAL_BA9405AA	39480	SRR1969332	2014	8		Europe	United Kingdom	Enteritidis	Enteritidis	1425	6917
SAL_BA9406AA	65053	SRR1969331	2014	11		Europe	United Kingdom	Enteritidis	Enteritidis	1425	24879
SAL_BA9409AA	50856	SRR1969328	2014	9		Europe	United Kingdom	Enteritidis	Enteritidis	1425	27930
SAL_BA9411AA	93568	SRR1969326	2015	3		Europe	United Kingdom	Enteritidis	Enteritidis	1425	30458
SAL_BA9422AA	25265	SRR1969315	2014	7		Europe	United Kingdom	Enteritidis	Enteritidis	3888	30449
SAL_BA9423AA	5879	SRR1969314	2014	4		Europe	United Kingdom	Enteritidis	Enteritidis	1425	30448
SAL_BA9426AA	27051	SRR1969311	2014	7		Europe	United Kingdom	Enteritidis	Enteritidis	3888	30446
SAL_BA9427AA	60166	SRR1969310	2014	10		Europe	United Kingdom	Enteritidis	Enteritidis	1425	30444
SAL_BA9428AA	5964	SRR1969309	2014	4		Europe	United Kingdom	Enteritidis	Enteritidis	1425	30443
SAL_BA9434AA	27976	SRR1969303	2014	7		Europe	United Kingdom	Enteritidis	Enteritidis	1425	30438
SAL_BA9435AA	9180	SRR1969302	2014	5		Europe	United Kingdom	Enteritidis	Enteritidis	1425	30437
SAL_BA9436AA	37798	SRR1969301	2014	7	28	Europe	United Kingdom	Enteritidis	Enteritidis	3888	30436
SAL_BA9440AA	38315	SRR1969298	2014	7	28	Europe	United Kingdom	Enteritidis	Enteritidis	3888	29305
SAL_BA9448AA	12110	SRR1969291	2014	5	8	Europe	United Kingdom	Enteritidis	Enteritidis	1425	30426
SAL_BA9456AA	63932	SRR1969284	2014	11	6	Europe	United Kingdom	Enteritidis	Enteritidis	1425	30418
SAL_BA9457AA	41995	SRR1969283	2014	8	20	Europe	United Kingdom	Enteritidis	Enteritidis	3888	30417
SAL_BA9458AA	13807	SRR1969282	2014	5		Europe	United Kingdom	Enteritidis	Enteritidis	1425	30416
SAL_BA9462AA	55540	SRR1969279	2014	9		Europe	United Kingdom	Enteritidis	Enteritidis	3888	3640
SAL_BA9466AA	39452	SRR1969276	2014	7	28	Europe	United Kingdom	Enteritidis	Enteritidis	3888	30409
SAL_BA9467AA	89999	SRR1969275	2015	2		Europe	United Kingdom	Enteritidis	Enteritidis	1425	30408
SAL_BA9468AA	63047	SRR1969274	2014	10		Europe	United Kingdom	Enteritidis	Enteritidis	1425	27396
SAL_BA9472AA	CFSAN002042	SRR900926						Enteritidis	Enteritidis	1425	30404
SAL_BA9473AA	42168	SRR1969271	2014	9		Europe	United Kingdom	Enteritidis	Enteritidis	1425	30403
SAL_BA9474AA	CFSAN002041	SRR900925				North America	United States	Enteritidis	Enteritidis	1425	30402
SAL_BA9482AA	41988	SRR1969266	2014	8		Europe	United Kingdom	Enteritidis	Enteritidis	3888	30397
SAL_BA9487AA	93531	SRR1969261	2015	3		Europe	United Kingdom	Enteritidis	Enteritidis	1425	30393
SAL_BA9489AA	9140	SRR1969259	2014	5		Europe	United Kingdom	Enteritidis	Enteritidis	1425	25142
SAL_BA9491AA	68696	SRR1969258	2014	11	13	Europe	United Kingdom	Enteritidis	Enteritidis	1425	25666
SAL_BA9505AA	Salmonella_ser__Enteritidis_	ERR226477						Enteritidis (Predicted)	Enteritidis	1425	30379
SAL_BA9517AA	Ke233-sc-2012-07-27T11:52:	ERR217471						Typhimurium	Enteritidis	1424	41359
SAL_BA9519AA	Ke232-sc-2012-07-27T11:52:	ERR217470						Enteritidis	Enteritidis	1424	30369
SAL_BA9524AA	23078	SRR1969242	2014	6		Europe	United Kingdom	Enteritidis	Enteritidis	1425	30364
SAL_BA9525AA	Ke219-sc-2012-07-27T11:51:	ERR217467						Enteritidis	Enteritidis	1425	30363
SAL_BA9532AA	96976	SRR1969238	2015	3		Europe	United Kingdom	Enteritidis	Enteritidis	1425	30358
SAL_BA9536AA	Ke205-sc-2012-07-27T11:51:	ERR217462						Enteritidis	Enteritidis	1424	30354
SAL_BA9538AA	Ke204-sc-2012-07-27T11:51:	ERR217461						Enteritidis	Enteritidis	1425	30318
SAL_BA9540AA	Ke199-sc-2012-07-27T11:51:	ERR217460						Enteritidis	Enteritidis	1424	30256
SAL_BA9545AA	63588	SRR1969231	2014	10		Europe	United Kingdom	Enteritidis	Enteritidis	1425	30348
SAL_BA9546AA	Ke196-sc-2012-07-27T11:51:	ERR217457						Enteritidis	Enteritidis	1424	30347
SAL_BA9550AA	32550	SRR1969229	2014	7	18	Europe	United Kingdom	Enteritidis	Enteritidis	3888	30344
SAL_BA9553AA	Ke189-sc-2012-07-27T11:51:	ERR217453						Enteritidis	Enteritidis	1425	30338
SAL_BA9556AA	76001	SRR1969226	2014	12		Europe	United Kingdom	Enteritidis	Enteritidis	1425	30339
SAL_BA9557AA	Ke187-sc-2012-07-27T11:51:	ERR217451						Enteritidis	Enteritidis	1425	30338
SAL_BA9560AA	38775	SRR1969224	2014	8		Europe	United Kingdom	Enteritidis	Enteritidis	1425	30335
SAL_BA9563AA	Ke181-sc-2012-07-27T11:51:	ERR217448						Enteritidis	Enteritidis	1424	30333
SAL_BA9565AA	Ke180-sc-2012-07-27T11:51:	ERR217447						Enteritidis	Enteritidis	1424	41357
SAL_BA9566AA	50833	SRR1969221	2014	9		Europe	United Kingdom	Enteritidis	Enteritidis	1425	30331
SAL_BA9570AA	90184	SRR1969219	2015	2		Europe	United Kingdom	Enteritidis	Enteritidis	1425	30328
SAL_BA9573AA	Ke172-sc-2012-07-27T11:51:	ERR217443						Enteritidis	Enteritidis	1424	30324
SAL_BA9576AA	Ke169-sc-2012-07-27T11:51:	ERR217442						Enteritidis	Enteritidis	1425	30322
SAL_BA9580AA	Ke164-sc-2012-07-27T11:51:	ERR217440						Enteritidis	Enteritidis	1425	30319

SAL_BA9581AA	Ke163-sc-2012-07-27T11:51:	ERR217439						Enteritidis	Enteritidis	1425	30318
SAL_BA9582AA	91269	SRR1969213	2015	2		Europe	United Kingdom	Enteritidis	Enteritidis	1425	7210
SAL_BA9584AA	Ke160-sc-2012-07-27T11:51:	ERR217438						Enteritidis	Enteritidis	1425	30316
SAL_BA9589AA	43427	SRR1969209	2014	8	28	Europe	United Kingdom	Enteritidis	Enteritidis	3888	30311
SAL_BA9591AA	Ke152-sc-2012-07-27T11:51:	ERR217435						Enteritidis	Enteritidis	1425	30309
SAL_BA9593AA	Ke151-sc-2012-07-27T11:51:	ERR217434						Enteritidis	Enteritidis	1424	30307
SAL_BA9594AA	34179	SRR1969206	2014	7	21	Europe	United Kingdom	Enteritidis	Enteritidis	3888	30306
SAL_BA9598AA	69884	SRR1969204	2014	11		Europe	United Kingdom	Enteritidis	Enteritidis	3888	30302
SAL_BA9599AA	Ke143-sc-2012-07-27T11:51:	ERR217431						Enteritidis	Enteritidis	1424	30301
SAL_BA9607AA	Ke122-sc-2012-07-27T11:51:	ERR217427						Enteritidis	Enteritidis	1424	30293
SAL_BA9609AA	Ke117-sc-2012-07-27T11:51:	ERR217426						Enteritidis	Enteritidis	1424	30292
SAL_BA9613AA	36520	SRR1969196	2014	8		Europe	United Kingdom	Enteritidis	Enteritidis	1425	30289
SAL_BA9615AA	9184	SRR1969195	2014	5		Europe	United Kingdom	Enteritidis	Enteritidis	1425	30288
SAL_BA9617AA	13432	SRR1969194	2014	5		Europe	United Kingdom	Enteritidis	Enteritidis	3888	30287
SAL_BA9621AA	65125	SRR1969192	2014	11	4	Europe	United Kingdom	Enteritidis	Enteritidis	3888	30284
SAL_BA9623AA	96977	SRR1969191	2015	3		Europe	United Kingdom	Enteritidis	Enteritidis	1425	30282
SAL_BA9625AA	39514	SRR1969190	2014	8	14	Europe	United Kingdom	Enteritidis	Enteritidis	3888	30280
SAL_BA9627AA	25261	SRR1969189	2014	7		Europe	United Kingdom	Enteritidis	Enteritidis	1425	30278
SAL_BA9630AA	14870	SRR1969187	2014	5		Europe	United Kingdom	Enteritidis	Enteritidis	1425	30275
SAL_BA9652AA	65429	SRR1969176	2014	9		Europe	United Kingdom	Enteritidis	Enteritidis	3888	7867
SAL_BA9653AA	Ke048-sc-2012-07-27T11:51:	ERR217405						Enteritidis	Enteritidis	1424	30259
SAL_BA9656AA	Ke044-sc-2012-07-27T11:51:	ERR217403						Enteritidis	Enteritidis	1424	30250
SAL_BA9658AA	Ke042-sc-2012-07-27T11:51:	ERR217402						Enteritidis	Enteritidis	1424	30256
SAL_BA9661AA	32478	SRR1969172	2014	7	17	Europe	United Kingdom	Enteritidis	Enteritidis	3888	30253
SAL_BA9664AA	Ke033-sc-2012-07-27T11:51:	ERR217399						Enteritidis	Enteritidis	1424	30250
SAL_BA9681AA	83706	SRR1969162	2014	12	4	Europe	United Kingdom	Enteritidis	Enteritidis	1425	25680
SAL_BA9691AA	Ke016-sc-2012-07-27T11:50:	ERR217386						Enteritidis	Enteritidis	1425	30230
SAL_BA9702AA	40791	SRR1969152	2014	8		Europe	United Kingdom	Enteritidis	Enteritidis	1425	30221
SAL_BA9704AA	86907	SRR1969151	2015	2		Europe	United Kingdom	Enteritidis	Enteritidis	1425	30220
SAL_BA9710AA	37115	SRR1969148	2014	8		Europe	United Kingdom	Enteritidis	Enteritidis	1425	30215
SAL_BA9723AA	Ke267-sc-2012-07-27T11:52:	ERR212519						Enteritidis	Enteritidis	1425	29967
SAL_BA9730AA	34168	SRR1969138	2014	7		Europe	United Kingdom	Enteritidis	Enteritidis	1425	30202
SAL_BA9736AA	31609	SRR1969135	2014	7		Europe	United Kingdom	Enteritidis	Enteritidis	1425	30199
SAL_BA9738AA	36518	SRR1969134	2014	7	23	Europe	United Kingdom	Enteritidis	Enteritidis	3888	2066
SAL_BA9740AA	31964	SRR1969133	2014	7		Europe	United Kingdom	Enteritidis	Enteritidis	1425	30197
SAL_BA9743AA	Ke491-sc-2012-07-27T11:54:	ERR212676						Enteritidis	Enteritidis	1424	30188
SAL_BA9747AA	Ke488-sc-2012-07-27T11:54:	ERR212673						Enteritidis	Enteritidis	1424	30191
SAL_BA9748AA	85733	SRR1969129	2015	2		Europe	United Kingdom	Enteritidis	Enteritidis	1425	27537
SAL_BA9749AA	Ke486-sc-2012-07-27T11:54:	ERR212671						Enteritidis	Enteritidis	1425	30190
SAL_BA9750AA	57109	SRR1969128	2014	8		Europe	United Kingdom	Enteritidis	Enteritidis	1425	30189
SAL_BA9751AA	Ke485-sc-2012-07-27T11:54:	ERR212670						Enteritidis	Enteritidis	1424	30188
SAL_BA9753AA	Ke484-sc-2012-07-27T11:54:	ERR212669						Enteritidis	Enteritidis	1424	30186
SAL_BA9758AA	36494	SRR1969124	2014	7		Europe	United Kingdom	Enteritidis	Enteritidis	1425	7771
SAL_BA9760AA	37011	SRR1969123	2014	8		Europe	United Kingdom	Enteritidis	Enteritidis	1425	30180
SAL_BA9766AA	53132	SRR1969120	2014	9		Europe	United Kingdom	Enteritidis	Enteritidis	1425	30175
SAL_BA9770AA	72265	SRR1969118	2014	12		Europe	United Kingdom	Enteritidis	Enteritidis	3888	3630
SAL_BA9773AA	38321	SRR1969116	2014	8		Europe	United Kingdom	Enteritidis	Enteritidis	1425	30171
SAL_BA9775AA	52964	SRR1969115	2014	9		Europe	United Kingdom	Enteritidis	Enteritidis	1425	30169
SAL_BA9779AA	50872	SRR1969113	2014	9		Europe	United Kingdom	Enteritidis	Enteritidis	1425	30165
SAL_BA9791AA	68695	SRR1969107	2014	11		Europe	United Kingdom	Enteritidis	Enteritidis	3888	30158
SAL_BA9799AA	38781	SRR1969103	2014	8		Europe	United Kingdom	Enteritidis	Enteritidis	1425	30152
SAL_BA9814AA	83590	SRR1969095	2015	1		Europe	United Kingdom	Enteritidis	Enteritidis	1425	29703
SAL_BA9819AA	27801	SRR1969078	2014	7		Europe	United Kingdom	Enteritidis	Enteritidis	1425	30138
SAL_BA9820AA	Ke448-sc-2012-07-27T11:53:	ERR212633						Enteritidis	Enteritidis	1424	30136
SAL_BA9822AA	34181	SRR1969076	2014	7	22	Europe	United Kingdom	Enteritidis	Enteritidis	3888	30135
SAL_BA9825AA	Ke445-sc-2012-07-27T11:53:	ERR212630						Enteritidis	Enteritidis	1425	30132
SAL_BA9834AA	46586	SRR1969069	2014	7	14	Europe	United Kingdom	Enteritidis	Enteritidis	3888	30125
SAL_BA9836AA	57110	SRR1969068	2014	8	1	Europe	United Kingdom	Enteritidis	Enteritidis	3888	30124
SAL_BA9844AA	13810	SRR1969064	2014	5		Europe	United Kingdom	Enteritidis	Enteritidis	1425	30119

SAL_BA9846AA	23503	SRR1969063	2014	6		Europe	United Kingdom	Enteritidis	Enteritidis	1425	30117
SAL_BA9847AA	Ke434-sc-2012-07-27T11:53:	ERR212619						Enteritidis	Enteritidis	1424	30116
SAL_BA9848AA	27794	SRR1969062	2014	7		Europe	United Kingdom	Enteritidis	Enteritidis	1425	30115
SAL_BA9850AA	40272	SRR1969061	2014	8		Europe	United Kingdom	Enteritidis	Enteritidis	3906	30113
SAL_BA9856AA	31657	SRR1969057	2014	7		Europe	United Kingdom	Enteritidis	Enteritidis	26543	30107
SAL_BA9872AA	Ke422-sc-2012-07-27T11:53:	ERR212607						Enteritidis	Enteritidis	1425	30096
SAL_BA9881AA	Ke417-sc-2012-07-27T11:53:	ERR212602						Enteritidis	Enteritidis	1424	30088
SAL_BA9882AA	65055	SRR1969043	2014	11		Europe	United Kingdom	Enteritidis	Enteritidis	1425	29851
SAL_BA9896AA	38330	SRR1969036	2014	8		Europe	United Kingdom	Enteritidis	Enteritidis	1425	30074
SAL_BA9897AA	Ke409-sc-2012-07-27T11:53:	ERR212594						Enteritidis	Enteritidis	1425	30073
SAL_BA9902AA	38767	SRR1969033	2014	8		Europe	United Kingdom	Enteritidis	Enteritidis	1425	20758
SAL_BA9907AA	84460	SRR1969031	2015	2		Europe	United Kingdom	Enteritidis	Enteritidis	1425	30066
SAL_BA9923AA	36395	SRR1969024	2014	8	3	Europe	United Kingdom	Enteritidis	Enteritidis	3888	30055
SAL_BA9933AA	34183	SRR1969018	2014	7		Europe	United Kingdom	Enteritidis	Enteritidis	3888	30047
SAL_BA9938AA	24039	SRR1969015	2014	6	23	Europe	United Kingdom	Enteritidis	Enteritidis	3888	30043
SAL_BA9946AA	14133	SRR1969011	2014	5		Europe	United Kingdom	Enteritidis	Enteritidis	3888	30037
SAL_BA9958AA	27797	SRR1969004	2014	6	27	Europe	United Kingdom	Enteritidis	Enteritidis	3888	30026
SAL_BA9960AA	23493	SRR1969003	2014	6	19	Europe	United Kingdom	Enteritidis	Enteritidis	3888	30024
SAL_BA9962AA	32471	SRR1969002	2014	7	20	Europe	United Kingdom	Enteritidis	Enteritidis	3888	30022
SAL_BA9970AA	38413	SRR1968998	2014	8		Europe	United Kingdom	Enteritidis	Enteritidis	1425	30015
SAL_BA9978AA	36495	SRR1968994	2014	7		Europe	United Kingdom	Enteritidis	Enteritidis	1425	30007
SAL_BA9986AA	26637	SRR1968990	2014	7		Europe	United Kingdom	Enteritidis	Enteritidis	1425	30001
SAL_BA9997AA	12131	SRR1968985	2014	5		Europe	United Kingdom	Enteritidis	Enteritidis	1425	29993
SAL_BA9999AA	31618	SRR1968984	2014	7		Europe	United Kingdom	Enteritidis	Enteritidis	1425	29992
SAL_CA0006AA	Ke304-sc-2012-07-27T11:52:	ERR212539						Enteritidis	Enteritidis	1425	29988
SAL_CA0009AA	46012	SRR1968978	2014	8		Europe	United Kingdom	Enteritidis	Enteritidis	1425	26972
SAL_CA0011AA	93572	SRR1968977	2015	3		Europe	United Kingdom	Enteritidis	Enteritidis	28005	26420
SAL_CA0014AA	46004	SRR1968976	2014	8		Europe	United Kingdom	Enteritidis	Enteritidis	1425	29985
SAL_CA0018AA	45998	SRR1968974	2014	8		Europe	United Kingdom	Enteritidis	Enteritidis	3888	29982
SAL_CA0020AA	36387	SRR1968973	2014	8		Europe	United Kingdom	Enteritidis	Enteritidis	1425	29980
SAL_CA0021AA	Ke292-sc-2012-07-27T11:52:	ERR212531						Enteritidis	Enteritidis	1425	29979
SAL_CA0022AA	31623	SRR1968972	2014	7	9	Europe	United Kingdom	Enteritidis	Enteritidis	3888	29978
SAL_CA0028AA	65047	SRR1968969	2014	11		Europe	United Kingdom	Enteritidis	Enteritidis	1425	25223
SAL_CA0029AA	Ke288-sc-2012-07-27T11:52:	ERR212527						Typhimurium	Enteritidis	1425	29972
SAL_CA0034AA	Ke276-sc-2012-07-27T11:52:	ERR212524						Enteritidis	Enteritidis	1425	29967
SAL_CA0079AA	29386	SRR1968943	2014	7		Europe	United Kingdom	Enteritidis	Enteritidis	1425	29927
SAL_CA0089AA	39482	SRR1968938	2014	8		Europe	United Kingdom	Enteritidis	Enteritidis	3888	29919
SAL_CA0094AA	63660	SRR1968935	2014	10		Europe	United Kingdom	Enteritidis	Enteritidis	1425	29914
SAL_CA0102AA	37036	SRR1968931	2014	8		Europe	France	Enteritidis	Enteritidis	3888	551
SAL_CA0106AA	38369	SRR1968929	2014	7	30	Europe	United Kingdom	Enteritidis	Enteritidis	3888	29905
SAL_CA0110AA	36390	SRR1968927	2014	8		Europe	United Kingdom	Enteritidis	Enteritidis	1425	29901
SAL_CA0112AA	99782	SRR1968926	2015	3		Europe	United Kingdom	Enteritidis	Enteritidis	3888	26603
SAL_CA0119AA	96972	SRR1968922	2015	3		Europe	United Kingdom	Enteritidis	Enteritidis	1425	7575
SAL_CA0125AA	40249	SRR1968919	2014	8		Europe	United Kingdom	Enteritidis	Enteritidis	26481	29890
SAL_CA0129AA	84447	SRR1968917	2015	2		Europe	United Kingdom	Enteritidis	Enteritidis	1425	2277
SAL_CA0138AA	40795	SRR1968912	2014	8	15	Europe	United Kingdom	Enteritidis	Enteritidis	3888	25250
SAL_CA0140AA	60098	SRR1968911	2014	10		Europe	United Kingdom	Enteritidis	Enteritidis	1425	29880
SAL_CA0150AA	34177	SRR1968906	2014	7	22	Europe	United Kingdom	Enteritidis	Enteritidis	3888	7153
SAL_CA0161AA	37800	SRR1968900	2014	7	29	Europe	United Kingdom	Enteritidis	Enteritidis	3888	29862
SAL_CA0163AA	34175	SRR1968899	2014	7	22	Europe	United Kingdom	Enteritidis	Enteritidis	3888	29860
SAL_CA0177AA	65054	SRR1968892	2014	11		Europe	United Kingdom	Enteritidis	Enteritidis	1425	29851
SAL_CA0184AA	32524	SRR1968889	2014	7		Europe	United Kingdom	Enteritidis	Enteritidis	1425	26210
SAL_CA0186AA	38783	SRR1968888	2014	8		Europe	United Kingdom	Enteritidis	Enteritidis	3888	29846
SAL_CA0188AA	99073	SRR1968887	2015	3		Europe	United Kingdom	Enteritidis	Enteritidis	1425	503
SAL_CA0199AA	41984	SRR1968882	2014	8	14	Europe	United Kingdom	Enteritidis	Enteritidis	3888	29839
SAL_CA0204AA	73137	SRR1968880	2014	12		Europe	United Kingdom	Enteritidis	Enteritidis	1425	6917
SAL_CA0229AA	45615	SRR1968869	2014	9		Europe	United Kingdom	Enteritidis	Enteritidis	1425	24676
SAL_CA0240AA	31930	SRR1968863	2014	7		Europe	United Kingdom	Enteritidis	Enteritidis	3888	29815
SAL_CA0244AA	23462	SRR1968861	2014	6	10	Europe	United Kingdom	Enteritidis	Enteritidis	3888	29811

SAL_CA0253AA	65126	SRR1968857	2014	11		Europe	United Kingdom	Enteritidis	Enteritidis	26481	29803
SAL_CA0257AA	21788	SRR1968855	2014	6		Europe	United Kingdom	Enteritidis	Enteritidis	3888	29800
SAL_CA0261AA	14132	SRR1968853	2014	5		Europe	United Kingdom	Enteritidis	Enteritidis	1425	29797
SAL_CA0266AA	13861	SRR1968851	2014	5		Europe	United Kingdom	Enteritidis	Enteritidis	1425	29794
SAL_CA0278AA	13859	SRR1968845	2014	5		Europe	United Kingdom	Enteritidis	Enteritidis	1425	29783
SAL_CA0292AA	12189	SRR1968839	2014	5		Europe	United Kingdom	Enteritidis	Enteritidis	1425	29771
SAL_CA0302AA	83302	SRR1968834	2015	1		Europe	United Kingdom	Enteritidis	Enteritidis	3888	3635
SAL_CA0306AA	38325	SRR1968832	2014	7	30	Europe	United Kingdom	Enteritidis	Enteritidis	3888	29759
SAL_CA0308AA	52978	SRR1968831	2014	10		Europe	United Kingdom	Enteritidis	Enteritidis	1425	29757
SAL_CA0311AA	65394	SRR1968829	2014	10		Europe	United Kingdom	Enteritidis	Enteritidis	1425	24726
SAL_CA0315AA	94090	SRR1968827	2015	3		Europe	United Kingdom	Enteritidis	Enteritidis	1425	29752
SAL_CA0317AA	38310	SRR1968826	2014	8		Europe	United Kingdom	Enteritidis	Enteritidis	1425	29750
SAL_CA0348AA	9281	SRR1968811	2014	5		Europe	United Kingdom	Enteritidis	Enteritidis	1425	29722
SAL_CA0356AA	13835	SRR1968807	2014	5	15	Europe	United Kingdom	Enteritidis	Enteritidis	3888	29715
SAL_CA0358AA	21782	SRR1968806	2014	6	2	Europe	United Kingdom	Enteritidis	Enteritidis	3888	29714
SAL_CA0368AA	56158	SRR1968801	2014	9		Europe	United Kingdom	Enteritidis	Enteritidis	3888	7867
SAL_CA0370AA	83725	SRR1968800	2015	1		Europe	United Kingdom	Enteritidis	Enteritidis	1425	29703
SAL_CA0378AA	37811	SRR1968796	2014	7	28	Europe	United Kingdom	Enteritidis	Enteritidis	3888	29695
SAL_CA0389AA	23057	SRR1968791	2014	6	7	Europe	United Kingdom	Enteritidis	Enteritidis	3888	29684
SAL_CA0399AA	65334	SRR1968786	2014	9		Europe	United Kingdom	Enteritidis	Enteritidis	1425	28868
SAL_CA0401AA	37037	SRR1968785	2014	8		Europe	France	Enteritidis	Enteritidis	3888	29673
SAL_CA0405AA	65050	SRR1968783	2014	11		Europe	United Kingdom	Enteritidis	Enteritidis	1425	29669
SAL_CA0407AA	65105	SRR1968782	2014	10		Europe	United Kingdom	Enteritidis	Enteritidis	3888	29667
SAL_CA0411AA	68689	SRR1968780	2014	11	12	Europe	United Kingdom	Enteritidis	Enteritidis	28536	29663
SAL_CA0413AA	40236	SRR1968779	2014	8		Europe	United Kingdom	Enteritidis	Enteritidis	1425	29661
SAL_CA0414AA	36477	SRR1968778	2014	7	21	Europe	United Kingdom	Enteritidis	Enteritidis	3888	2066
SAL_CA0417AA	23470	SRR1968777	2014	6		Europe	United Kingdom	Enteritidis	Enteritidis	1425	29658
SAL_CA0443AA	63902	SRR1968764	2014	11		Europe	United Kingdom	Enteritidis	Enteritidis	1425	29634
SAL_CA0451AA	38306	SRR1968760	2014	8		Europe	United Kingdom	Enteritidis	Enteritidis	1425	29628
SAL_CA0455AA	45617	SRR1968758	2014	9		Europe	United Kingdom	Enteritidis	Enteritidis	3888	29624
SAL_CA0461AA	46011	SRR1968755	2014	8	9	Europe	United Kingdom	Enteritidis	Enteritidis	3888	2066
SAL_CA0467AA	36393	SRR1968751	2014	8		Europe	United Kingdom	Enteritidis	Enteritidis	1425	29617
SAL_CA0472AA	31966	SRR1968746	2014	7	25	Europe	United Kingdom	Enteritidis	Enteritidis	3888	29612
SAL_CA0473AA	6447	SRR1968745	2014	4		Europe	United Kingdom	Enteritidis	Enteritidis	1425	29611
SAL_CA0475AA	34191	SRR1968743	2014	7	29	Europe	United Kingdom	Enteritidis	Enteritidis	1425	29609
SAL_CA0492AA	60141	SRR1968734	2014	10		Europe	United Kingdom	Enteritidis	Enteritidis	1425	29598
SAL_CA0496AA	40366	SRR1968732	2014	8	28	Europe	United Kingdom	Enteritidis	Enteritidis	1425	29594
SAL_CA0502AA	68703	SRR1968729	2014	11		Europe	United Kingdom	Kisangani	Enteritidis	1425	29589
SAL_CA0505AA	94263	SRR1968728	2015	3		Europe	United Kingdom	Enteritidis	Enteritidis	1425	29586
SAL_CA0509AA	51242	SRR1968726	2014	9		Europe	United Kingdom	Enteritidis	Enteritidis	1425	24690
SAL_CA0544AA	50335	SRR1968709	2014	9		Europe	United Kingdom	Enteritidis	Enteritidis	3888	7867
SAL_CA0560AA	31924	SRR1968701	2014	7		Europe	United Kingdom	Enteritidis	Enteritidis	3888	29540
SAL_CA0570AA	36386	SRR1968696	2014	8		Europe	United Kingdom	Enteritidis	Enteritidis	1425	26244
SAL_CA0585AA	14540	SRR1968688	2014	5		Europe	United Kingdom	Enteritidis	Enteritidis	1425	29522
SAL_CA0587AA	27978	SRR1968686	2014	7		Europe	United Kingdom	Enteritidis	Enteritidis	1425	29520
SAL_CA0593AA	27063	SRR1968683	2014	7		Europe	United Kingdom	Enteritidis	Enteritidis	3888	29515
SAL_CA0597AA	25227	SRR1968681	2014	7		Europe	United Kingdom	Enteritidis	Enteritidis	1425	29511
SAL_CA0607AA	31656	SRR1968675	2014	7	10	Europe	United Kingdom	Enteritidis	Enteritidis	3888	29502
SAL_CA0618AA	41989	SRR1968667	2014	8		Europe	United Kingdom	Enteritidis	Enteritidis	1425	29497
SAL_CA0634AA	36475	SRR1968659	2014	7	21	Europe	United Kingdom	Enteritidis	Enteritidis	3888	29482
SAL_CA0636AA	40276	SRR1968658	2014	8		Europe	United Kingdom	Enteritidis	Enteritidis	1425	29480
SAL_CA0644AA	38344	SRR1968654	2014	7	28	Europe	United Kingdom	Enteritidis	Enteritidis	3888	26229
SAL_CA0646AA	39516	SRR1968653	2014	8		Europe	United Kingdom	Enteritidis	Enteritidis	1425	29471
SAL_CA0650AA	25266	SRR1968651	2014	7	1	Europe	United Kingdom	Enteritidis	Enteritidis	3888	512
SAL_CA0656AA	42002	SRR1968648	2014	8		Europe	United Kingdom	Enteritidis	Enteritidis	1425	29464
SAL_CA0666AA	83726	SRR1968641	2015	1		Europe	United Kingdom	Enteritidis	Enteritidis	1425	29456
SAL_CA0668AA	46013	SRR1968640	2014	8		Europe	United Kingdom	Enteritidis	Enteritidis	1425	29454
SAL_CA0682AA	24052	SRR1968633	2014	6		Europe	United Kingdom	Enteritidis	Enteritidis	1425	29441
SAL_CA0685AA	JT1409	ERR018882						Enteritidis (Predicted)	Enteritidis	1425	29439

SAL_CA0686AA	23498	SRR1968631	2014	6		Europe	United Kingdom	Enteritidis	Enteritidis	1425	29438
SAL_CA0694AA	37813	SRR1968627	2014	7	31	Europe	United Kingdom	Enteritidis	Enteritidis	3888	29430
SAL_CA0697AA	38427	SRR1968626	2014	8		Europe	United Kingdom	Enteritidis	Enteritidis	1425	29427
SAL_CA0699AA	37008	SRR1968625	2014	8		Europe	United Kingdom	Enteritidis	Enteritidis	1425	29425
SAL_CA0704AA	65128	SRR1968622	2014	11		Europe	United Kingdom	Enteritidis	Enteritidis	1425	26283
SAL_CA0708AA	25343	SRR1968620	2014	7		Europe	United Kingdom	Enteritidis	Enteritidis	1425	29417
SAL_CA0716AA	34207	SRR1968616	2014	7		Europe	United Kingdom	Enteritidis	Enteritidis	1425	25842
SAL_CA0720AA	40274	SRR1968614	2014	8	11	Europe	United Kingdom	Enteritidis	Enteritidis	3888	29406
SAL_CA0735AA	45613	SRR1968606	2014	9		Europe	United Kingdom	Enteritidis	Enteritidis	1425	29393
SAL_CA0741AA	65434	SRR1968603	2014	9	30	Europe	United Kingdom	Enteritidis	Enteritidis	3888	7317
SAL_CA0743AA	63631	SRR1968602	2014	10		Europe	United Kingdom	Enteritidis	Enteritidis	1425	24879
SAL_CA0763AA	34188	SRR1968592	2014	7	25	Europe	United Kingdom	Enteritidis	Enteritidis	3888	29369
SAL_CA0768AA	27979	SRR1968590	2014	7		Europe	United Kingdom	Enteritidis	Enteritidis	1425	29365
SAL_CA0770AA	38422	SRR1968589	2014	7	25	Europe	United Kingdom	Enteritidis	Enteritidis	3888	29363
SAL_CA0776AA	69900	SRR1968586	2014	11		Europe	United Kingdom	Enteritidis	Enteritidis	3888	29358
SAL_CA0780AA	27014	SRR1968584	2014	7		Europe	United Kingdom	Enteritidis	Enteritidis	1425	29354
SAL_CA0795AA	39435	SRR1968577	2014	6		Europe	United Kingdom	Enteritidis	Enteritidis	1425	29340
SAL_CA0797AA	9178	SRR1968576	2014	5		Europe	United Kingdom	Enteritidis	Enteritidis	1425	29338
SAL_CA0817AA	68693	SRR1968566	2014	11		Europe	United Kingdom	Enteritidis	Enteritidis	1425	29320
SAL_CA0821AA	31972	SRR1968564	2014	7	25	Europe	United Kingdom	Enteritidis	Enteritidis	3888	2066
SAL_CA0835AA	61837	SRR1968557	2014	10		Europe	United Kingdom	Enteritidis	Enteritidis	1425	25076
SAL_CA0837AA	37825	SRR1968556	2014	7	28	Europe	United Kingdom	Enteritidis	Enteritidis	3888	29305
SAL_CA0844AA	36364	SRR1968553	2014	7	30	Europe	United Kingdom	Enteritidis	Enteritidis	3888	514
SAL_CA0848AA	38408	SRR1968551	2014	8		Europe	United Kingdom	Enteritidis	Enteritidis	1425	29301
SAL_CA0859AA	67565	SRR1968544	2014	11		Europe	United Kingdom	Enteritidis	Enteritidis	1425	25142
SAL_CA0860AA	99785	SRR1968543	2015	3		Europe	United Kingdom	Enteritidis	Enteritidis	3888	29293
SAL_CA0866AA	84439	SRR1968539	2015	2		Europe	United Kingdom	Enteritidis	Enteritidis	1425	29287
SAL_CA0885AA	37817	SRR1968529	2014	7	26	Europe	United Kingdom	Enteritidis	Enteritidis	3888	27851
SAL_CA0894AA	CFSAN002046	SRR749062				North America	Mexico	Enteritidis	Enteritidis	1425	29263
SAL_CA0896AA	CFSAN002045	SRR749061				North America	United States	Enteritidis	Enteritidis	1425	29261
SAL_CA0898AA	CFSAN002044	SRR749060				North America	United States	Enteritidis	Enteritidis	3801	29259
SAL_CA0903AA	40224	SRR1968520	2014	8	13	Europe	United Kingdom	Enteritidis	Enteritidis	3888	29255
SAL_CA0907AA	40235	SRR1968516	2014	8	12	Europe	United Kingdom	Enteritidis	Enteritidis	3888	29251
SAL_CA0914AA	23508	SRR1968511	2014	6	25	Europe	United Kingdom	Enteritidis	Enteritidis	1425	29244
SAL_CA1024AA	52974	SRR1968508	2014	10		Europe	United Kingdom	Enteritidis	Enteritidis	3888	29241
SAL_CA1030AA	73138	SRR1968505	2014	12		Europe	United Kingdom	Enteritidis	Enteritidis	1425	29237
SAL_CA1051AA	84472	SRR1968495	2015	2		Europe	United Kingdom	Enteritidis	Enteritidis	1425	29230
SAL_CA1058AA	45611	SRR1968492	2014	9		Europe	United Kingdom	Enteritidis	Enteritidis	1425	24690
SAL_CA1067AA	31918	SRR1968488	2014	7		Europe	United Kingdom	Enteritidis	Enteritidis	8003	29225
SAL_CA1071AA	46579	SRR1968486	2014	7	17	Europe	United Kingdom	Enteritidis	Enteritidis	3888	29224
SAL_CA1075AA	27962	SRR1968484	2014	7		Europe	United Kingdom	Enteritidis	Enteritidis	1425	29221
SAL_CA1093AA	31975	SRR1968475	2014	7	25	Europe	United Kingdom	Enteritidis	Enteritidis	3888	29212
SAL_CA1104AA	34192	SRR1968470	2014	7		Europe	United Kingdom	Enteritidis	Enteritidis	1425	29207
SAL_CA1111AA	6449	SRR1968467	2014	4		Europe	United Kingdom	Enteritidis	Enteritidis	1425	29204
SAL_CA1117AA	63735	SRR1968464	2014	10		Europe	United Kingdom	Enteritidis	Enteritidis	1425	24563
SAL_CA1119AA	13857	SRR1968463	2014	5		Europe	United Kingdom	Enteritidis	Enteritidis	1425	29201
SAL_CA1122AA	50358	SRR1968462	2014	9		Europe	United Kingdom	Enteritidis	Enteritidis	3888	29200
SAL_CA1133AA	89883	SRR1968452	2015	2		Europe	United Kingdom	Enteritidis	Enteritidis	1425	29190
SAL_CA1142AA	63645	SRR1968443	2014	10		Europe	United Kingdom	Enteritidis	Enteritidis	1425	29183
SAL_CA1145AA	24983	SRR1968440	2014	7		Europe	United Kingdom	Enteritidis	Enteritidis	3888	29180
SAL_CA1154AA	9282	SRR1968431	2014	5		Europe	United Kingdom	Enteritidis	Enteritidis	1425	29171
SAL_CA1156AA	38367	SRR1968429	2014	8		Europe	United Kingdom	Enteritidis	Enteritidis	1425	29169
SAL_CA1157AA	25268	SRR1968428	2014	7		Europe	United Kingdom	Enteritidis	Enteritidis	1425	29168
SAL_CA1162AA	34203	SRR1968423	2014	7	21	Europe	United Kingdom	Enteritidis	Enteritidis	3888	29164
SAL_CA1163AA	5914	SRR1968422	2014	4		Europe	United Kingdom	Enteritidis	Enteritidis	1425	29163
SAL_CA1168AA	13389	SRR1968417	2014	5	12	Europe	United Kingdom	Enteritidis	Enteritidis	3888	29158
SAL_CA1171AA	39437	SRR1968414	2014	6		Europe	United Kingdom	Enteritidis	Enteritidis	26142	29155
SAL_CA1172AA	31979	SRR1968413	2014	7		Europe	United Kingdom	Enteritidis	Enteritidis	1425	29154
SAL_CA1176AA	96957	SRR1968409	2015	3		Europe	United Kingdom	Enteritidis	Enteritidis	1425	29150

SAL_CA1179AA	39508	SRR1968406	2014	8	18	Europe	United Kingdom	Enteritidis	Enteritidis	28525	29147
SAL_CA1184AA	39479	SRR1968401	2014	8		Europe	United Kingdom	Enteritidis	Enteritidis	1425	29142
SAL_CA1186AA	40280	SRR1968399	2014	8	19	Europe	United Kingdom	Enteritidis	Enteritidis	1425	29141
SAL_CA1190AA	5373	SRR1968395	2014	4		Europe	United Kingdom	Enteritidis	Enteritidis	1425	6917
SAL_CA1197AA	99053	SRR1968388	2015	3		Europe	United Kingdom	Enteritidis	Enteritidis	1425	29131
SAL_CA1211AA	69284	SRR1968374	2014	11		Europe	United Kingdom	Enteritidis	Enteritidis	3888	3621
SAL_CA1215AA	23497	SRR1968370	2014	6		Europe	United Kingdom	Enteritidis	Enteritidis	1425	29117
SAL_CA1216AA	98245	SRR1968369	2015	3		Europe	United Kingdom	Enteritidis	Enteritidis	1425	29116
SAL_CA1218AA	27005	SRR1968367	2014	7		Europe	United Kingdom	Enteritidis	Enteritidis	1425	29114
SAL_CA1227AA	40326	SRR1968358	2014	8		Europe	United Kingdom	Enteritidis	Enteritidis	3888	29107
SAL_CA1229AA	23491	SRR1968356	2014	6		Europe	United Kingdom	Enteritidis	Enteritidis	1425	29104
SAL_CA1230AA	63628	SRR1968355	2014	10		Europe	United Kingdom	Enteritidis	Enteritidis	1425	29103
SAL_CA1231AA	25347	SRR1968354	2014	7		Europe	United Kingdom	Enteritidis	Enteritidis	1425	29102
SAL_CA1236AA	39440	SRR1968349	2014	7		Europe	United Kingdom	Enteritidis	Enteritidis	1425	29097
SAL_CA1237AA	9298	SRR1968348	2014	5		Europe	United Kingdom	Enteritidis	Enteritidis	1425	29096
SAL_CA1239AA	50879	SRR1968346	2014	9		Europe	United Kingdom	Enteritidis	Enteritidis	3888	29094
SAL_CA1240AA	45968	SRR1968345	2014	7		Europe	United Kingdom	Enteritidis	Enteritidis	1425	29022
SAL_CA1246AA	96241	SRR1968339	2015	3		Europe	United Kingdom	Enteritidis	Enteritidis	1425	29089
SAL_CA1253AA	46590	SRR1968332	2014	7	17	Europe	United Kingdom	Enteritidis	Enteritidis	3888	29082
SAL_CA1258AA	31965	SRR1968329	2014	7	25	Europe	United Kingdom	Enteritidis	Enteritidis	3888	2066
SAL_CA1262AA	40322	SRR1968327	2014	8		Europe	United Kingdom	Enteritidis	Enteritidis	3888	29078
SAL_CA1278AA	57111	SRR1968319	2014	8	1	Europe	United Kingdom	Enteritidis	Enteritidis	3888	29070
SAL_CA1291AA	50889	SRR1968313	2014	9		Europe	United Kingdom	Enteritidis	Enteritidis	1425	29064
SAL_CA1299AA	40273	SRR1968309	2014	8	12	Europe	United Kingdom	Enteritidis	Enteritidis	3888	29060
SAL_CA1302AA	12190	SRR1968307	2014	5		Europe	United Kingdom	Enteritidis	Enteritidis	1425	29058
SAL_CA1304AA	27973	SRR1968306	2014	7	17	Europe	United Kingdom	Enteritidis	Enteritidis	1425	29057
SAL_CA1311AA	9310	SRR1968303	2014	4	23	Europe	United Kingdom	Enteritidis	Enteritidis	3888	29054
SAL_CA1315AA	45614	SRR1968301	2014	9		Europe	United Kingdom	Enteritidis	Enteritidis	1425	29052
SAL_CA1318AA	25320	SRR1968299	2014	6	24	Europe	United Kingdom	Enteritidis	Enteritidis	3888	29050
SAL_CA1320AA	9268	SRR1968298	2014	4		Europe	United Kingdom	Enteritidis	Enteritidis	1425	29049
SAL_CA1323AA	13856	SRR1968297	2014	5		Europe	United Kingdom	Enteritidis	Enteritidis	1425	29048
SAL_CA1339AA	73195	SRR1968289	2014	12		Europe	United Kingdom	Enteritidis	Enteritidis	1425	29043
SAL_CA1353AA	72275	SRR1968282	2014	12		Europe	United Kingdom	Enteritidis	Enteritidis	1425	25004
SAL_CA1359AA	90511	SRR1968279	2015	2		Europe	United Kingdom	Enteritidis	Enteritidis	1425	29038
SAL_CA1364AA	55554	SRR1968277	2014	9		Europe	United Kingdom	Enteritidis	Enteritidis	1425	29036
SAL_CA1374AA	50355	SRR1968272	2014	9		Europe	United Kingdom	Enteritidis	Enteritidis	1425	29031
SAL_CA1382AA	40796	SRR1968268	2014	8		Europe	United Kingdom	Enteritidis	Enteritidis	27796	29027
SAL_CA1384AA	50364	SRR1968267	2014	9		Europe	United Kingdom	Enteritidis	Enteritidis	3897	29026
SAL_CA1386AA	53097	SRR1968266	2014	9		Europe	United Kingdom	Enteritidis	Enteritidis	1425	7550
SAL_CA1390AA	99080	SRR1968264	2015	3		Europe	United Kingdom	Enteritidis	Enteritidis	1425	29024
SAL_CA1394AA	37000	SRR1968262	2014	7		Europe	United Kingdom	Enteritidis	Enteritidis	1425	29022
SAL_CA1396AA	91291	SRR1968261	2015	2		Europe	United Kingdom	Enteritidis	Enteritidis	3888	29021
SAL_CA1406AA	91898	SRR1968256	2014	11		Europe	United Kingdom	Enteritidis	Enteritidis	1425	29018
SAL_CA1414AA	12187	SRR1968252	2014	5		Europe	United Kingdom	Enteritidis	Enteritidis	1425	29014
SAL_CA1418AA	65378	SRR1968250	2014	10		Europe	United Kingdom	Enteritidis	Enteritidis	26142	29012
SAL_CA1422AA	27060	SRR1968248	2014	7		Europe	United Kingdom	Enteritidis	Enteritidis	1425	29010
SAL_CA1429AA	75992	SRR1968244	2014	12		Europe	United Kingdom	Enteritidis	Enteritidis	1425	29007
SAL_CA1435AA	25342	SRR1968239	2014	7		Europe	United Kingdom	Enteritidis	Enteritidis	3888	29003
SAL_CA1440AA	83319	SRR1968235	2015	1		Europe	United Kingdom	Enteritidis	Enteritidis	1425	28999
SAL_CA1444AA	51292	SRR1968233	2014	9		Europe	United Kingdom	Enteritidis	Enteritidis	1425	28997
SAL_CA1454AA	80603	SRR1968228	2015	1		Europe	United Kingdom	Enteritidis	Enteritidis	1425	7117
SAL_CA1459AA	63622	SRR1968225	2014	9		Europe	United Kingdom	Enteritidis	Enteritidis	1425	28989
SAL_CA1461AA	56111	SRR1968224	2014	9		Europe	United Kingdom	Enteritidis	Enteritidis	1425	28988
SAL_CA1465AA	9177	SRR1968222	2014	5		Europe	United Kingdom	Enteritidis	Enteritidis	1425	28986
SAL_CA1478AA	31917	SRR1968213	2014	7	18	Europe	United Kingdom	Enteritidis	Enteritidis	3888	28978
SAL_CA1480AA	31651	SRR1968212	2014	7	15	Europe	United Kingdom	Enteritidis	Enteritidis	1425	28976
SAL_CA1486AA	55586	SRR1968209	2014	9		Europe	United Kingdom	Enteritidis	Enteritidis	1425	28970
SAL_CA1499AA	38412	SRR1968202	2014	8	6	Europe	United Kingdom	Enteritidis	Enteritidis	1425	28959
SAL_CA1519AA	68691	SRR1968192	2014	11		Europe	United Kingdom	Enteritidis	Enteritidis	1425	28940

SAL_CA1521AA	63665	SRR1968191	2014	10		Europe	United Kingdom	Enteritidis	Enteritidis	1425	28938
SAL_CA1535AA	53100	SRR1968184	2014	9		Europe	United Kingdom	Enteritidis	Enteritidis	1425	28925
SAL_CA1540AA	68686	SRR1968182	2014	11		Europe	United Kingdom	Enteritidis	Enteritidis	1425	25914
SAL_CA1543AA	91232	SRR1968180	2015	1		Europe	United Kingdom	Enteritidis	Enteritidis	3888	28918
SAL_CA1552AA	53101	SRR1968176	2014	9		Europe	United Kingdom	Enteritidis	Enteritidis	3888	7867
SAL_CA1558AA	60171	SRR1968173	2014	10		Europe	United Kingdom	Enteritidis	Enteritidis	1425	28904
SAL_CA1560AA	23076	SRR1968172	2014	6		Europe	United Kingdom	Enteritidis	Enteritidis	1425	28902
SAL_CA1562AA	25189	SRR1968171	2014	7		Europe	United Kingdom	Enteritidis	Enteritidis	1425	28900
SAL_CA1568AA	9176	SRR1968168	2014	5		Europe	United Kingdom	Enteritidis	Enteritidis	1425	28894
SAL_CA1575AA	83341	SRR1968164	2015	1		Europe	United Kingdom	Enteritidis	Enteritidis	1425	28888
SAL_CA1588AA	65355	SRR1968156	2014	10		Europe	United Kingdom	Enteritidis	Enteritidis	1425	28875
SAL_CA1594AA	63775	SRR1968152	2014	10		Europe	United Kingdom	Enteritidis	Enteritidis	1425	28869
SAL_CA1596AA	62988	SRR1968151	2014	9		Europe	United Kingdom	Enteritidis	Enteritidis	1425	28868
SAL_CA1602AA	63730	SRR1968147	2014	10		Europe	United Kingdom	Enteritidis	Enteritidis	1425	28863
SAL_CA1617AA	50835	SRR1968138	2014	9		Europe	United Kingdom	Enteritidis	Enteritidis	3888	28851
SAL_CA1621AA	91217	SRR1968136	2015	2		Europe	United Kingdom	Enteritidis	Enteritidis	1425	28848
SAL_CA1623AA	99757	SRR1968135	2015	3		Europe	United Kingdom	Enteritidis	Enteritidis	1425	28846
SAL_CA1625AA	56156	SRR1968134	2014	9		Europe	United Kingdom	Enteritidis	Enteritidis	1425	28844
SAL_CA1640AA	23065	SRR1968127	2014	6		Europe	United Kingdom	Enteritidis	Enteritidis	1425	28829
SAL_CA1644AA	9263	SRR1968125	2014	4	24	Europe	United Kingdom	Enteritidis	Enteritidis	1425	28825
SAL_CA1648AA	29393	SRR1968123	2014	7		Europe	United Kingdom	Enteritidis	Enteritidis	1425	28821
SAL_CA1654AA	37827	SRR1968120	2014	7	24	Europe	United Kingdom	Enteritidis	Enteritidis	3888	28815
SAL_CA1678AA	40271	SRR1968108	2014	8		Europe	United Kingdom	Enteritidis	Enteritidis	1425	28792
SAL_CA1680AA	89105	SRR1968107	2015	2		Europe	United Kingdom	Enteritidis	Enteritidis	1425	6917
SAL_CA1682AA	26666	SRR1968106	2014	7		Europe	United Kingdom	Enteritidis	Enteritidis	1425	28789
SAL_CA1709AA	45579	SRR1968089	2014	9	9	Europe	United Kingdom	Enteritidis	Enteritidis	1425	28762
SAL_CA1715AA	38326	SRR1968083	2014	8		Europe	United Kingdom	Enteritidis	Enteritidis	1425	28756
SAL_CA1717AA	61877	SRR1968081	2014	10		Europe	United Kingdom	Enteritidis	Enteritidis	1425	28754
SAL_CA1719AA	98249	SRR1968079	2015	3		Europe	United Kingdom	Enteritidis	Enteritidis	1425	24766
SAL_CA1720AA	72313	SRR1968078	2014	12		Europe	United Kingdom	Enteritidis	Enteritidis	1425	28752
SAL_CA1723AA	46016	SRR1968075	2014	8		Europe	United Kingdom	Enteritidis	Enteritidis	1425	28750
SAL_CA1727AA	14539	SRR1968073	2014	5		Europe	United Kingdom	Enteritidis	Enteritidis	26495	28748
SAL_CA1733AA	14536	SRR1968070	2014	5		Europe	United Kingdom	Enteritidis	Enteritidis	1425	28745
SAL_CA1735AA	65373	SRR1968069	2014	10	2	Europe	United Kingdom	Enteritidis	Enteritidis	3888	28744
SAL_CA1737AA	23449	SRR1968068	2014	5		Europe	United Kingdom	Enteritidis	Enteritidis	-1662	28743
SAL_CA1741AA	89897	SRR1968066	2015	2		Europe	United Kingdom	Enteritidis	Enteritidis	1425	28741
SAL_CA1743AA	41999	SRR1968065	2014	8		Europe	United Kingdom	Enteritidis	Enteritidis	1425	28740
SAL_CA1747AA	42171	SRR1968063	2014	8	26	Europe	United Kingdom	Enteritidis	Enteritidis	3888	28739
SAL_CA1753AA	90509	SRR1968060	2015	2		Europe	United Kingdom	Enteritidis	Enteritidis	1425	28737
SAL_CA1755AA	21789	SRR1968059	2014	6		Europe	United Kingdom	Enteritidis	Enteritidis	3888	28736
SAL_CA1763AA	37114	SRR1968055	2014	8		Europe	United Kingdom	Enteritidis	Enteritidis	1425	28733
SAL_CA1765AA	95022	SRR1968054	2015	3		Europe	United Kingdom	Enteritidis	Enteritidis	3897	28732
SAL_CA1771AA	38417	SRR1968051	2014	7	28	Europe	United Kingdom	Enteritidis	Enteritidis	3888	28729
SAL_CA1776AA	34170	SRR1968048	2014	7		Europe	United Kingdom	Enteritidis	Enteritidis	26493	28726
SAL_CA1781AA	95017	SRR1968045	2015	3	11	Europe	United Kingdom	Enteritidis	Enteritidis	1425	28723
SAL_CA1784AA	37810	SRR1968043	2014	7	29	Europe	United Kingdom	Enteritidis	Enteritidis	3888	28721
SAL_CA1794AA	41942	SRR1968038	2014	8		Europe	United Kingdom	Enteritidis	Enteritidis	1425	28716
SAL_CA1797AA	12130	SRR1968037	2014	5		Europe	United Kingdom	Enteritidis	Enteritidis	3888	28715
SAL_CA1801AA	38416	SRR1968035	2014	8		Europe	United Kingdom	Enteritidis	Enteritidis	1425	28713
SAL_CA1820AA	13387	SRR1968024	2014	5		Europe	United Kingdom	Enteritidis	Enteritidis	1425	28704
SAL_CA1838AA	12150	SRR1968015	2014	5		Europe	United Kingdom	Enteritidis	Enteritidis	1425	28696
SAL_CA1862AA	99058	SRR1968003	2015	3		Europe	United Kingdom	Enteritidis	Enteritidis	1425	28688
SAL_CA1864AA	42001	SRR1968002	2014	8		Europe	United Kingdom	Enteritidis	Enteritidis	1425	28687
SAL_CA1866AA	53106	SRR1968001	2014	9		Europe	United Kingdom	Enteritidis	Enteritidis	1425	28686
SAL_CA1871AA	37006	SRR1967999	2014	8	3	Europe	United Kingdom	Enteritidis	Enteritidis	3888	28684
SAL_CA1873AA	63580	SRR1967998	2014	9		Europe	United Kingdom	Enteritidis	Enteritidis	3888	28683
SAL_CA1874AA	91294	SRR1967997	2015	2		Europe	United Kingdom	Enteritidis	Enteritidis	1425	28682
SAL_CA1876AA	26652	SRR1967996	2014	7		Europe	United Kingdom	Enteritidis	Enteritidis	1425	28681
SAL_CA1882AA	36394	SRR1967993	2014	8	4	Europe	United Kingdom	Enteritidis	Enteritidis	3888	2066

SAL_CA1887AA	39447	SRR1967990	2014	7	18	Europe	United Kingdom	Enteritidis	Enteritidis	3888	28676
SAL_CA1889AA	32466	SRR1967989	2014	7	18	Europe	United Kingdom	Enteritidis	Enteritidis	3888	28675
SAL_CA1892AA	65123	SRR1967987	2014	11		Europe	United Kingdom	Enteritidis	Enteritidis	3888	25814
SAL_CA1895AA	53005	SRR1967985	2014	10		Europe	United Kingdom	Enteritidis	Enteritidis	1425	28673
SAL_CA1908AA	75965	SRR1967979	2014	12		Europe	United Kingdom	Enteritidis	Enteritidis	1425	28667
SAL_CA1913AA	36384	SRR1967976	2014	8		Europe	United Kingdom	Enteritidis	Enteritidis	1425	28664
SAL_CA1918AA	96230	SRR1967974	2015	3		Europe	United Kingdom	Enteritidis	Enteritidis	1425	28662
SAL_CA1919AA	65407	SRR1967973	2014	9	17	Europe	United Kingdom	Enteritidis	Enteritidis	1425	28661
SAL_CA1925AA	89901	SRR1967970	2015	2		Europe	United Kingdom	Enteritidis	Enteritidis	1425	27831
SAL_CA1946AA	96240	SRR1967960	2015	3		Europe	United Kingdom	Enteritidis	Enteritidis	1425	28651
SAL_CA1948AA	90476	SRR1967959	2015	2		Europe	United Kingdom	Enteritidis	Enteritidis	1425	28650
SAL_CA1951AA	37105	SRR1967958	2014	8		Europe	United Kingdom	Enteritidis	Enteritidis	1425	28649
SAL_CA1953AA	63720	SRR1967957	2014	10		Europe	United Kingdom	Enteritidis	Enteritidis	1425	28648
SAL_CA1955AA	40298	SRR1967956	2014	8		Europe	United Kingdom	Enteritidis	Enteritidis	1425	28647
SAL_CA1957AA	14898	SRR1967955	2014	5		Europe	United Kingdom	Enteritidis	Enteritidis	1425	28646
SAL_CA1965AA	25219	SRR1967951	2014	7		Europe	United Kingdom	Enteritidis	Enteritidis	1425	28643
SAL_CA1976AA	43426	SRR1967945	2014	9		Europe	United Kingdom	Enteritidis	Enteritidis	3888	28637
SAL_CA1984AA	63045	SRR1967941	2014	10		Europe	United Kingdom	Enteritidis	Enteritidis	26481	28633
SAL_CA1997AA	63685	SRR1967934	2014	10		Europe	United Kingdom	Enteritidis	Enteritidis	1425	28627
SAL_CA2004AA	80556	SRR1967931	2015	1		Europe	United Kingdom	Enteritidis	Enteritidis	1425	28624
SAL_CA2010AA	56153	SRR1967928	2014	9		Europe	United Kingdom	Enteritidis	Enteritidis	3888	25428
SAL_CA2018AA	90472	SRR1967924	2015	2		Europe	United Kingdom	Enteritidis	Enteritidis	1425	27622
SAL_CA2024AA	25003	SRR1967921	2014	7		Europe	United Kingdom	Enteritidis	Enteritidis	1425	28620
SAL_CA2037AA	23509	SRR1967914	2014	6		Europe	United Kingdom	Enteritidis	Enteritidis	1425	28613
SAL_CA2039AA	43430	SRR1967913	2014	9		Europe	United Kingdom	Enteritidis	Enteritidis	1425	28612
SAL_CA2045AA	31919	SRR1967910	2014	7		Europe	United Kingdom	Enteritidis	Enteritidis	1425	28609
SAL_CA2050AA	14126	SRR1967907	2014	5		Europe	United Kingdom	Enteritidis	Enteritidis	1425	28606
SAL_CA2052AA	37821	SRR1967906	2014	8		Europe	United Kingdom	Enteritidis	Enteritidis	1425	28605
SAL_CA2055AA	14883	SRR1967905	2014	5		Europe	United Kingdom	Enteritidis	Enteritidis	26483	28604
SAL_CA2059AA	38393	SRR1967903	2014	8		Europe	United Kingdom	Enteritidis	Enteritidis	1425	28602
SAL_CA2060AA	91962	SRR1967902	2015	2		Europe	United Kingdom	Enteritidis	Enteritidis	1425	28601
SAL_CA2066AA	39448	SRR1967899	2014	7		Europe	United Kingdom	Enteritidis	Enteritidis	1425	28598
SAL_CA2072AA	63625	SRR1967895	2014	9		Europe	United Kingdom	Enteritidis	Enteritidis	1425	28595
SAL_CA2077AA	50851	SRR1967892	2014	9		Europe	United Kingdom	Enteritidis	Enteritidis	1425	28591
SAL_CA2084AA	38332	SRR1967887	2014	7	25	Europe	United Kingdom	Enteritidis	Enteritidis	3888	2066
SAL_CA2093AA	63040	SRR1967878	2014	10		Europe	United Kingdom	Enteritidis	Enteritidis	8469	28576
SAL_CA2094AA	96964	SRR1967877	2015	3		Europe	United Kingdom	Enteritidis	Enteritidis	1425	28575
SAL_CA2109AA	25275	SRR1967862	2014	7		Europe	United Kingdom	Enteritidis	Enteritidis	1425	28563
SAL_CA2113AA	31968	SRR1967858	2014	7	26	Europe	United Kingdom	Enteritidis	Enteritidis	3888	28559
SAL_CA2121AA	5919	SRR1967850	2014	4		Europe	United Kingdom	Enteritidis	Enteritidis	1425	28551
SAL_CA2132AA	65046	SRR1967839	2014	11		Europe	United Kingdom	Enteritidis	Enteritidis	3888	28540
SAL_CA2133AA	38769	SRR1967838	2014	8		Europe	United Kingdom	Enteritidis	Enteritidis	1425	28539
SAL_CA2134AA	34169	SRR1967837	2014	7		Europe	United Kingdom	Enteritidis	Enteritidis	1425	28538
SAL_CA2138AA	89902	SRR1967833	2015	2		Europe	United Kingdom	Enteritidis	Enteritidis	1425	28535
SAL_CA2140AA	96229	SRR1967831	2015	3		Europe	United Kingdom	Enteritidis	Enteritidis	1425	28533
SAL_CA2141AA	68684	SRR1967830	2014	11		Europe	United Kingdom	Enteritidis	Enteritidis	1425	28532
SAL_CA2146AA	46015	SRR1967825	2014	8		Europe	United Kingdom	Enteritidis	Enteritidis	1425	28528
SAL_CA2150AA	9183	SRR1967821	2014	5		Europe	United Kingdom	Enteritidis	Enteritidis	1425	28525
SAL_CA2151AA	45608	SRR1967820	2014	9		Europe	United Kingdom	Enteritidis	Enteritidis	1425	28524
SAL_CA2157AA	67567	SRR1967814	2014	11		Europe	United Kingdom	Enteritidis	Enteritidis	1425	25914
SAL_CA2160AA	94100	SRR1967811	2015	3		Europe	United Kingdom	Enteritidis	Enteritidis	3888	28518
SAL_CA2162AA	57108	SRR1967809	2014	8		Europe	United Kingdom	Enteritidis	Enteritidis	1425	28516
SAL_CA2165AA	40230	SRR1967806	2014	8		Europe	United Kingdom	Enteritidis	Enteritidis	1425	28513
SAL_CA2167AA	40263	SRR1967804	2014	8		Europe	United Kingdom	Enteritidis	Enteritidis	1425	28512
SAL_CA2169AA	75957	SRR1967802	2014	12		Europe	United Kingdom	Enteritidis	Enteritidis	1425	28510
SAL_CA2170AA	50360	SRR1967801	2014	9	3	Europe	United Kingdom	Enteritidis	Enteritidis	3888	28509
SAL_CA2171AA	39444	SRR1967800	2014	7		Europe	United Kingdom	Enteritidis	Enteritidis	1425	28508
SAL_CA2175AA	21767	SRR1967796	2014	6	4	Europe	United Kingdom	Enteritidis	Enteritidis	3888	28504
SAL_CA2178AA	98287	SRR1967793	2015	3		Europe	United Kingdom	Enteritidis	Enteritidis	3888	28502

SAL_CA2184AA	31974	SRR1967787	2014	7	25	Europe	United Kingdom	Enteritidis	Enteritidis	3888	28497
SAL_CA2190AA	7410	SRR1967781	2014	4	29	Europe	United Kingdom	Enteritidis	Enteritidis	1425	28492
SAL_CA2191AA	24038	SRR1967780	2014	6	23	Europe	United Kingdom	Enteritidis	Enteritidis	3888	28491
SAL_CA2199AA	36531	SRR1967772	2014	7	29	Europe	United Kingdom	Enteritidis	Enteritidis	3888	28483
SAL_CA2200AA	36526	SRR1967771	2014	7	28	Europe	United Kingdom	Enteritidis	Enteritidis	3888	2066
SAL_CA2203AA	29387	SRR1967768	2014	7		Europe	United Kingdom	Enteritidis	Enteritidis	1425	28480
SAL_CA2205AA	38371	SRR1967766	2014	7	28	Europe	United Kingdom	Enteritidis	Enteritidis	3888	28478
SAL_CA2208AA	14899	SRR1967763	2014	5		Europe	United Kingdom	Enteritidis	Enteritidis	3888	28475
SAL_CA2209AA	57118	SRR1967762	2014	10		Europe	United Kingdom	Enteritidis	Enteritidis	3888	28474
SAL_CA2213AA	5934	SRR1967758	2014	4		Europe	United Kingdom	Enteritidis	Enteritidis	1425	28471
SAL_CA2214AA	9309	SRR1967757	2014	5		Europe	United Kingdom	Enteritidis	Enteritidis	3888	28470
SAL_CA2217AA	12192	SRR1967754	2014	5		Europe	United Kingdom	Enteritidis	Enteritidis	1425	28467
SAL_CA2221AA	43429	SRR1967750	2014	9		Europe	United Kingdom	Enteritidis	Enteritidis	1425	28464
SAL_CA2224AA	9181	SRR1967747	2014	5		Europe	United Kingdom	Enteritidis	Enteritidis	3888	28461
SAL_CA2231AA	63044	SRR1967740	2014	10		Europe	United Kingdom	Enteritidis	Enteritidis	1425	27354
SAL_CA2242AA	32464	SRR1967729	2014	7	17	Europe	United Kingdom	Enteritidis	Enteritidis	3888	28445
SAL_CA2246AA	53009	SRR1967725	2014	8		Europe	United Kingdom	Enteritidis (Predicted)	Enteritidis	1425	28441
SAL_CA2252AA	22253	SRR1967719	2014	6		Europe	United Kingdom	Enteritidis	Enteritidis	1425	28435
SAL_CA2255AA	38424	SRR1967716	2014	8		Europe	United Kingdom	Enteritidis	Enteritidis	3888	28432
SAL_CA2265AA	36523	SRR1967706	2014	8		Europe	United Kingdom	Enteritidis	Enteritidis	1425	28422
SAL_CA2273AA	56154	SRR1967698	2014	9		Europe	United Kingdom	Enteritidis	Enteritidis	3888	28415
SAL_CA2280AA	63678	SRR1967691	2014	10		Europe	United Kingdom	Enteritidis	Enteritidis	1425	24547
SAL_CA2284AA	5375	SRR1967687	2014	4		Europe	United Kingdom	Enteritidis	Enteritidis	1425	28406
SAL_CA2289AA	90477	SRR1967682	2015	2		Europe	United Kingdom	Enteritidis	Enteritidis	3888	28401
SAL_CA2290AA	63621	SRR1967681	2014	10		Europe	United Kingdom	Enteritidis	Enteritidis	26481	28400
SAL_CA2298AA	63779	SRR1967673	2014	10		Europe	United Kingdom	Enteritidis	Enteritidis	1425	28394
SAL_CA2313AA	43428	SRR1967658	2014	9		Europe	United Kingdom	Enteritidis	Enteritidis	26434	28383
SAL_CA2315AA	23443	SRR1967656	2014	4		Europe	United Kingdom	Enteritidis	Enteritidis	1425	28381
SAL_CA2319AA	25218	SRR1967652	2014	7		Europe	United Kingdom	Enteritidis	Enteritidis	3888	28377
SAL_CA2323AA	25233	SRR1967648	2014	7		Europe	United Kingdom	Enteritidis	Enteritidis	3888	28373
SAL_CA2324AA	13366	SRR1967647	2014	4		Europe	United Kingdom	Enteritidis	Enteritidis	1425	28372
SAL_CA2325AA	39509	SRR1967646	2014	8		Europe	United Kingdom	Enteritidis	Enteritidis	28500	28371
SAL_CA2338AA	53116	SRR1967633	2014	9		Europe	United Kingdom	Enteritidis	Enteritidis	1425	28361
SAL_CA2339AA	36363	SRR1967632	2014	8		Europe	United Kingdom	Enteritidis	Enteritidis	1425	28360
SAL_CA2342AA	51284	SRR1967629	2014	9		Europe	United Kingdom	Enteritidis	Enteritidis	1425	27094
SAL_CA2343AA	91267	SRR1967628	2015	2		Europe	United Kingdom	Enteritidis (Predicted)	Enteritidis	1425	28357
SAL_CA2346AA	36385	SRR1967625	2014	8	4	Europe	United Kingdom	Enteritidis	Enteritidis	3888	7317
SAL_CA2362AA	36464	SRR1967609	2014	7	25	Europe	United Kingdom	Enteritidis	Enteritidis	3888	2066
SAL_CA2364AA	56138	SRR1967607	2014	9		Europe	United Kingdom	Enteritidis	Enteritidis	3888	28341
SAL_CA2371AA	46589	SRR1967600	2014	7	17	Europe	United Kingdom	Enteritidis	Enteritidis	3888	28335
SAL_CA2372AA	13811	SRR1967599	2014	5		Europe	United Kingdom	Enteritidis	Enteritidis	1425	28334
SAL_CA2374AA	57130	SRR1967597	2014	9	30	Europe	United Kingdom	Enteritidis	Enteritidis	3888	28332
SAL_CA2378AA	38333	SRR1967593	2014	8		Europe	United Kingdom	Enteritidis (Predicted)	Enteritidis	3888	28328
SAL_CA2379AA	51268	SRR1967592	2014	9		Europe	United Kingdom	Enteritidis	Enteritidis	3888	7810
SAL_CA2381AA	14922	SRR1967590	2014	5		Europe	United Kingdom	Enteritidis	Enteritidis	3888	7105
SAL_CA2383AA	73222	SRR1967588	2014	12		Europe	United Kingdom	Enteritidis	Enteritidis	1425	26699
SAL_CA2390AA	84427	SRR1967581	2015	1		Europe	United Kingdom	Enteritidis	Enteritidis	1425	28321
SAL_CA2392AA	40321	SRR1967579	2014	8		Europe	United Kingdom	Enteritidis	Enteritidis	1425	28319
SAL_CA2395AA	38780	SRR1967576	2014	8		Europe	United Kingdom	Enteritidis	Enteritidis	1425	28316
SAL_CA2397AA	36503	SRR1967574	2014	7		Europe	United Kingdom	Enteritidis	Enteritidis	1425	28314
SAL_CA2399AA	38337	SRR1967572	2014	8		Europe	United Kingdom	Enteritidis	Enteritidis	1425	28312
SAL_CA2403AA	65452	SRR1967568	2014	10		Europe	United Kingdom	Enteritidis	Enteritidis	1425	28308
SAL_CA2409AA	86837	SRR1967562	2015	2		Europe	United Kingdom	Enteritidis	Enteritidis	1425	28302
SAL_CA2411AA	83682	SRR1967560	2014	10		Europe	United Kingdom	Typhi	Enteritidis	28454	26842
SAL_CA2412AA	98250	SRR1967559	2015	3		Europe	United Kingdom	Enteritidis	Enteritidis	1425	28300
SAL_CA2419AA	7394	SRR1967552	2014	4		Europe	United Kingdom	Enteritidis	Enteritidis	1425	28293
SAL_CA2421AA	13371	SRR1967550	2014	4		Europe	United Kingdom	Enteritidis	Enteritidis	3897	28291
SAL_CA2422AA	25269	SRR1967549	2014	7		Europe	United Kingdom	Enteritidis	Enteritidis	1425	28290
SAL_CA2423AA	31923	SRR1967548	2014	7	23	Europe	United Kingdom	Enteritidis	Enteritidis	3888	28289

SAL_CA2427AA	25171	SRR1967544	2014	7		Europe	United Kingdom	Enteritidis	Enteritidis	1425	28286
SAL_CA2428AA	69939	SRR1967543	2014	11		Europe	United Kingdom	Enteritidis	Enteritidis	3888	25602
SAL_CA2430AA	24036	SRR1967541	2014	6		Europe	United Kingdom	Enteritidis	Enteritidis	1425	28284
SAL_CA2431AA	24987	SRR1967540	2014	6	28	Europe	United Kingdom	Enteritidis	Enteritidis	3888	28283
SAL_CA2432AA	14119	SRR1967539	2014	5		Europe	United Kingdom	Enteritidis	Enteritidis	3888	28282
SAL_CA2438AA	32375	SRR1967533	2014	7	18	Europe	United Kingdom	Enteritidis	Enteritidis	3888	7153
SAL_CA2441AA	50838	SRR1967530	2014	9		Europe	United Kingdom	Enteritidis	Enteritidis	1425	28274
SAL_CA2445AA	9182	SRR1967526	2014	5	7	Europe	United Kingdom	Enteritidis	Enteritidis	1425	28270
SAL_CA2449AA	57122	SRR1967522	2014	10		Europe	United Kingdom	Enteritidis	Enteritidis	1425	28266
SAL_CA2455AA	37010	SRR1967516	2014	8		Europe	United Kingdom	Enteritidis	Enteritidis	1425	28260
SAL_CA2458AA	63590	SRR1967513	2014	9		Europe	United Kingdom	Enteritidis	Enteritidis	1425	28257
SAL_CA2461AA	38718	SRR1967510	2014	8	7	Europe	United Kingdom	Enteritidis	Enteritidis	3888	28254
SAL_CA2463AA	63706	SRR1967508	2014	10		Europe	United Kingdom	Enteritidis	Enteritidis	1425	7712
SAL_CA2465AA	25323	SRR1967506	2014	7		Europe	United Kingdom	Enteritidis	Enteritidis	3888	28251
SAL_CA2473AA	97866	SRR1967498	2015	3		Europe	United Kingdom	Enteritidis	Enteritidis	1425	27114
SAL_CA2474AA	38784	SRR1967497	2014	8		Europe	United Kingdom	Enteritidis	Enteritidis	1425	28243
SAL_CA2489AA	7425	SRR1967482	2014	4		Europe	United Kingdom	Enteritidis	Enteritidis	1425	28232
SAL_CA2492AA	37809	SRR1967479	2014	7	29	Europe	United Kingdom	Enteritidis	Enteritidis	3888	7612
SAL_CA2493AA	36476	SRR1967478	2014	7	21	Europe	United Kingdom	Enteritidis	Enteritidis	3888	7612
SAL_CA2498AA	27065	SRR1967473	2014	7		Europe	United Kingdom	Enteritidis	Enteritidis	1425	28225
SAL_CA2504AA	40323	SRR1967467	2014	8		Europe	United Kingdom	Enteritidis	Enteritidis	1425	28220
SAL_CA2505AA	38312	SRR1967466	2014	8		Europe	United Kingdom	Enteritidis	Enteritidis	3888	28218
SAL_CA2507AA	50842	SRR1967464	2014	9		Europe	United Kingdom	Enteritidis	Enteritidis	1425	28216
SAL_CA2509AA	32463	SRR1967462	2014	7	17	Europe	United Kingdom	Enteritidis	Enteritidis	3888	28214
SAL_CA2513AA	13396	SRR1967458	2014	5		Europe	United Kingdom	Enteritidis	Enteritidis	1425	28210
SAL_CA2515AA	38768	SRR1967456	2014	8		Europe	United Kingdom	Enteritidis	Enteritidis	1425	28208
SAL_CA2516AA	42003	SRR1967455	2014	8		Europe	United Kingdom	Enteritidis	Enteritidis	1425	28207
SAL_CA2521AA	69273	SRR1967450	2014	11		Europe	United Kingdom	Enteritidis (Predicted)	Enteritidis	1425	28202
SAL_CA2522AA	63725	SRR1967449	2014	10		Europe	United Kingdom	Enteritidis	Enteritidis	3888	28201
SAL_CA2538AA	98247	SRR1967433	2015	3		Europe	United Kingdom	Enteritidis	Enteritidis	1425	28191
SAL_CA2539AA	38343	SRR1967432	2014	8		Europe	United Kingdom	Enteritidis	Enteritidis	26493	28190
SAL_CA2546AA	22255	SRR1967425	2014	6		Europe	United Kingdom	Enteritidis	Enteritidis	1425	28185
SAL_CA2549AA	14099	SRR1967422	2014	5	21	Europe	United Kingdom	Enteritidis	Enteritidis	3888	28182
SAL_CA2555AA	65017	SRR1967416	2014	11		Europe	United Kingdom	Enteritidis	Enteritidis	1425	24707
SAL_CA2557AA	69917	SRR1967414	2014	11		Europe	United Kingdom	Enteritidis	Enteritidis	1425	7784
SAL_CA2561AA	31935	SRR1967410	2014	7		Europe	United Kingdom	Enteritidis	Enteritidis	1425	28172
SAL_CA2564AA	13357	SRR1967407	2014	4		Europe	United Kingdom	Enteritidis	Enteritidis	1425	28170
SAL_CA2567AA	36499	SRR1967404	2014	7		Europe	United Kingdom	Enteritidis	Enteritidis	3888	28167
SAL_CA2568AA	6432	SRR1967403	2014	4		Europe	United Kingdom	Enteritidis	Enteritidis	1425	28166
SAL_CA2570AA	3785	SRR1967401	2014	4		Europe	United Kingdom	Enteritidis	Enteritidis	1425	28164
SAL_CA2572AA	5353	SRR1967399	2014	4		Europe	United Kingdom	Enteritidis	Enteritidis	1425	28162
SAL_CA2575AA	53011	SRR1967396	2014	10		Europe	United Kingdom	Enteritidis	Enteritidis	3888	28159
SAL_CA2577AA	57103	SRR1967394	2014	7	31	Europe	United Kingdom	Enteritidis	Enteritidis	3888	28157
SAL_CA2580AA	52959	SRR1967391	2014	10		Europe	United Kingdom	Enteritidis	Enteritidis	1425	28154
SAL_CA2588AA	34184	SRR1967383	2014	7	22	Europe	United Kingdom	Enteritidis	Enteritidis	3888	28146
SAL_CA2591AA	63915	SRR1967380	2014	11		Europe	United Kingdom	Enteritidis	Enteritidis	1425	28143
SAL_CA2593AA	38317	SRR1967378	2014	7	28	Europe	United Kingdom	Enteritidis	Enteritidis	3888	28141
SAL_CA2596AA	12133	SRR1967375	2014	5		Europe	United Kingdom	Enteritidis	Enteritidis	1425	28138
SAL_CA2604AA	63781	SRR1967367	2014	10		Europe	United Kingdom	Enteritidis	Enteritidis	1425	25504
SAL_CA2615AA	91296	SRR1967356	2015	2		Europe	United Kingdom	Enteritidis	Enteritidis	1425	7210
SAL_CA2617AA	61881	SRR1967354	2014	10		Europe	United Kingdom	Enteritidis	Enteritidis	1425	28121
SAL_CA2619AA	25346	SRR1967352	2014	7		Europe	United Kingdom	Enteritidis	Enteritidis	3888	28119
SAL_CA2624AA	57156	SRR1967347	2014	10		Europe	United Kingdom	Enteritidis	Enteritidis	1425	28115
SAL_CA2631AA	50353	SRR1967340	2014	9		Europe	United Kingdom	Enteritidis	Enteritidis	1425	28109
SAL_CA2636AA	14895	SRR1967335	2014	5	21	Europe	United Kingdom	Enteritidis	Enteritidis	1425	28104
SAL_CA2637AA	23442	SRR1967334	2014	4		Europe	United Kingdom	Enteritidis	Enteritidis	1425	28103
SAL_CA2640AA	37803	SRR1967331	2014	8		Europe	United Kingdom	Enteritidis	Enteritidis	3888	28100
SAL_CA2642AA	65450	SRR1967329	2014	10		Europe	United Kingdom	Enteritidis	Enteritidis	1425	28098
SAL_CA2650AA	40324	SRR1967321	2014	8	18	Europe	United Kingdom	Enteritidis	Enteritidis	3888	28090

SAL_CA2652AA	40367	SRR1967319	2014	8		Europe	United Kingdom	Enteritidis	Enteritidis	1425	28088
SAL_CA2658AA	46575	SRR1967313	2014	7		Europe	United Kingdom	Enteritidis	Enteritidis	1425	28082
SAL_CA2661AA	38415	SRR1967310	2014	8	6	Europe	United Kingdom	Enteritidis	Enteritidis	1425	28079
SAL_CA2664AA	24986	SRR1967307	2014	6	28	Europe	United Kingdom	Enteritidis	Enteritidis	3888	28076
SAL_CA2668AA	25001	SRR1967303	2014	7		Europe	United Kingdom	Enteritidis	Enteritidis	1425	28072
SAL_CA2671AA	34172	SRR1967300	2014	7		Europe	United Kingdom	Enteritidis	Enteritidis	3888	3643
SAL_CA2673AA	36391	SRR1967298	2014	8		Europe	United Kingdom	Enteritidis	Enteritidis	1425	28068
SAL_CA2674AA	69881	SRR1967297	2014	11		Europe	United Kingdom	Enteritidis	Enteritidis	1425	28067
SAL_CA2685AA	34197	SRR1967286	2014	7	24	Europe	United Kingdom	Enteritidis	Enteritidis	3888	2066
SAL_CA2687AA	39515	SRR1967284	2014	8	11	Europe	United Kingdom	Enteritidis	Enteritidis	3888	28056
SAL_CA2688AA	24040	SRR1967283	2014	6	20	Europe	United Kingdom	Enteritidis	Enteritidis	3888	28055
SAL_CA2694AA	89106	SRR1967277	2015	2		Europe	United Kingdom	Enteritidis	Enteritidis	1425	25025
SAL_CA2696AA	68704	SRR1967275	2014	11		Europe	United Kingdom	Typhimurium	Enteritidis	1425	28049
SAL_CA2697AA	31920	SRR1967274	2014	7		Europe	United Kingdom	Enteritidis	Enteritidis	26483	28048
SAL_CA2701AA	39436	SRR1967270	2014	6		Europe	United Kingdom	Enteritidis	Enteritidis	1425	28045
SAL_CA2704AA	72302	SRR1967267	2014	12		Europe	United Kingdom	Enteritidis	Enteritidis	1425	28042
SAL_CA2708AA	38351	SRR1967263	2014	8		Europe	United Kingdom	Enteritidis	Enteritidis	3888	28038
SAL_CA2710AA	38411	SRR1967261	2014	7	30	Europe	United Kingdom	Enteritidis	Enteritidis	3888	28037
SAL_CA2716AA	97869	SRR1967255	2015	3		Europe	United Kingdom	Enteritidis	Enteritidis	1425	28031
SAL_CA2726AA	50853	SRR1967245	2014	9		Europe	United Kingdom	Enteritidis	Enteritidis	26142	28021
SAL_CA2728AA	63042	SRR1967243	2014	10		Europe	United Kingdom	Enteritidis	Enteritidis	3888	25253
SAL_CA2736AA	80551	SRR1967235	2015	1		Europe	United Kingdom	Enteritidis	Enteritidis	1425	28014
SAL_CA2739AA	57154	SRR1967232	2014	10		Europe	United Kingdom	Enteritidis	Enteritidis	1425	28011
SAL_CA2740AA	50847	SRR1967231	2014	9		Europe	United Kingdom	Enteritidis	Enteritidis	3888	28010
SAL_CA2743AA	21770	SRR1967228	2014	6	6	Europe	United Kingdom	Enteritidis	Enteritidis	3888	674
SAL_CA2749AA	25262	SRR1967222	2014	7		Europe	United Kingdom	Enteritidis	Enteritidis	1425	28004
SAL_CA2753AA	65367	SRR1967218	2014	10		Europe	United Kingdom	Enteritidis	Enteritidis	1425	27623
SAL_CA2755AA	32525	SRR1967216	2014	7	18	Europe	United Kingdom	Enteritidis	Enteritidis	3888	28000
SAL_CA2756AA	34206	SRR1967215	2014	7		Europe	United Kingdom	Enteritidis	Enteritidis	1425	27999
SAL_CA2767AA	83703	SRR1967204	2015	1		Europe	United Kingdom	Enteritidis	Enteritidis	1425	6917
SAL_CA2769AA	84428	SRR1967202	2015	1		Europe	United Kingdom	Enteritidis	Enteritidis	1425	27988
SAL_CA2772AA	65129	SRR1967199	2014	11		Europe	United Kingdom	Enteritidis	Enteritidis	1425	27985
SAL_CA2773AA	36492	SRR1967198	2014	7		Europe	United Kingdom	Enteritidis	Enteritidis	3888	27984
SAL_CA2774AA	96242	SRR1967197	2015	3		Europe	United Kingdom	Enteritidis	Enteritidis	1425	6917
SAL_CA2779AA	55549	SRR1967192	2014	9		Europe	United Kingdom	Enteritidis	Enteritidis	1425	27979
SAL_CA2784AA	34202	SRR1967187	2014	7	21	Europe	United Kingdom	Enteritidis	Enteritidis	3888	27975
SAL_CA2786AA	34196	SRR1967185	2014	7	25	Europe	United Kingdom	Enteritidis	Enteritidis	3888	27973
SAL_CA2788AA	65370	SRR1967183	2014	10		Europe	United Kingdom	Enteritidis	Enteritidis	1425	27971
SAL_CA2792AA	39450	SRR1967179	2014	7	23	Europe	United Kingdom	Enteritidis	Enteritidis	3888	27967
SAL_CA2793AA	63905	SRR1967178	2014	11		Europe	United Kingdom	Enteritidis	Enteritidis	1425	27966
SAL_CA2794AA	27034	SRR1967177	2014	7		Europe	United Kingdom	Enteritidis	Enteritidis	1425	27965
SAL_CA2796AA	94088	SRR1967175	2015	3		Europe	United Kingdom	Enteritidis	Enteritidis	1425	27964
SAL_CA2797AA	34185	SRR1967174	2014	7	22	Europe	United Kingdom	Enteritidis	Enteritidis	3888	7153
SAL_CA2799AA	65121	SRR1967172	2014	11		Europe	United Kingdom	Enteritidis	Enteritidis	1425	27961
SAL_CA2800AA	14901	SRR1967171	2014	5		Europe	United Kingdom	Enteritidis	Enteritidis	3888	27960
SAL_CA2801AA	37807	SRR1967170	2014	7	28	Europe	United Kingdom	Enteritidis	Enteritidis	3888	27959
SAL_CA2802AA	61859	SRR1967169	2014	10	10	Europe	United Kingdom	Enteritidis	Enteritidis	3888	27958
SAL_CA2807AA	40798	SRR1967164	2014	8		Europe	United Kingdom	Enteritidis	Enteritidis	1425	27954
SAL_CA2809AA	69948	SRR1967162	2014	11		Europe	United Kingdom	Enteritidis	Enteritidis	1425	27952
SAL_CA2811AA	23472	SRR1967160	2014	6	13	Europe	United Kingdom	Enteritidis	Enteritidis	3888	27950
SAL_CA2813AA	51261	SRR1967158	2014	9		Europe	United Kingdom	Enteritidis	Enteritidis	1425	27948
SAL_CA2817AA	65399	SRR1967154	2014	10		Europe	United Kingdom	Enteritidis (Predicted)	Enteritidis	1425	21622
SAL_CA2825AA	84412	SRR1967146	2015	1		Europe	United Kingdom	Enteritidis	Enteritidis	3888	27938
SAL_CA2829AA	27054	SRR1967142	2014	7		Europe	United Kingdom	Enteritidis	Enteritidis	1425	27934
SAL_CA2834AA	52967	SRR1967137	2014	10		Europe	United Kingdom	Enteritidis	Enteritidis	1425	27930
SAL_CA2838AA	68690	SRR1967133	2014	11	12	Europe	United Kingdom	Enteritidis	Enteritidis	1425	27927
SAL_CA2840AA	31611	SRR1967131	2014	7	14	Europe	United Kingdom	Enteritidis	Enteritidis	3888	27925
SAL_CA2848AA	63041	SRR1967123	2014	10		Europe	United Kingdom	Enteritidis	Enteritidis	1425	27918
SAL_CA2850AA	65390	SRR1967121	2014	9	12	Europe	United Kingdom	Enteritidis	Enteritidis	3888	24946

SAL_CA2852AA	45618	SRR1967119	2014	9		Europe	United Kingdom	Enteritidis	Enteritidis	3888	27916
SAL_CA2856AA	32473	SRR1967115	2014	7		Europe	United Kingdom	Enteritidis	Enteritidis	3888	27912
SAL_CA2860AA	45609	SRR1967111	2014	9		Europe	United Kingdom	Enteritidis	Enteritidis	1425	27908
SAL_CA2862AA	90524	SRR1967109	2015	2		Europe	United Kingdom	Enteritidis	Enteritidis	1425	25546
SAL_CA2863AA	63629	SRR1967108	2014	9		Europe	United Kingdom	Enteritidis	Enteritidis	1425	27906
SAL_CA2872AA	21776	SRR1967099	2014	6	3	Europe	United Kingdom	Enteritidis	Enteritidis	3888	27897
SAL_CA2877AA	9284	SRR1967094	2014	5		Europe	United Kingdom	Enteritidis	Enteritidis	1425	27892
SAL_CA2879AA	65382	SRR1967092	2014	10		Europe	United Kingdom	Enteritidis	Enteritidis	3906	27890
SAL_CA2880AA	85708	SRR1967091	2015	2		Europe	United Kingdom	Enteritidis	Enteritidis	1425	27237
SAL_CA2882AA	36524	SRR1967089	2014	7	22	Europe	United Kingdom	Enteritidis	Enteritidis	3888	27888
SAL_CA2885AA	13365	SRR1967086	2014	4		Europe	United Kingdom	Enteritidis	Enteritidis	1425	27885
SAL_CA2886AA	32472	SRR1967085	2014	7	18	Europe	United Kingdom	Enteritidis	Enteritidis	3888	2066
SAL_CA2891AA	34186	SRR1967080	2014	7	25	Europe	United Kingdom	Enteritidis	Enteritidis	3888	2066
SAL_CA2895AA	52972	SRR1967076	2014	9	26	Europe	United Kingdom	Enteritidis	Enteritidis	3888	2066
SAL_CA2900AA	36493	SRR1967071	2014	7		Europe	United Kingdom	Enteritidis	Enteritidis	1425	7771
SAL_CA2908AA	5873	SRR1967063	2014	4		Europe	United Kingdom	Enteritidis	Enteritidis	1425	27871
SAL_CA2910AA	7408	SRR1967061	2014	4		Europe	United Kingdom	Enteritidis	Enteritidis	1425	27869
SAL_CA2914AA	12185	SRR1967057	2014	5	13	Europe	United Kingdom	Enteritidis	Enteritidis	1425	27866
SAL_CA2915AA	22207	SRR1967056	2014	6		Europe	United Kingdom	Enteritidis	Enteritidis	1425	27865
SAL_CA2918AA	34198	SRR1967053	2014	7	24	Europe	United Kingdom	Enteritidis	Enteritidis	3888	27863
SAL_CA2919AA	69923	SRR1967052	2014	11		Europe	United Kingdom	Enteritidis	Enteritidis	1425	27862
SAL_CA2925AA	46582	SRR1967046	2014	7	16	Europe	United Kingdom	Enteritidis	Enteritidis	3888	27858
SAL_CA2930AA	6467	SRR1967041	2014	4		Europe	United Kingdom	Enteritidis	Enteritidis	3888	27854
SAL_CA2932AA	46591	SRR1967039	2014	7	18	Europe	United Kingdom	Enteritidis	Enteritidis	3888	27852
SAL_CA2933AA	37814	SRR1967038	2014	7	30	Europe	United Kingdom	Enteritidis	Enteritidis	3888	27851
SAL_CA2937AA	63652	SRR1967034	2014	10		Europe	United Kingdom	Enteritidis	Enteritidis	1425	25859
SAL_CA2938AA	36389	SRR1967033	2014	8		Europe	United Kingdom	Enteritidis	Enteritidis	1425	27848
SAL_CA2942AA	22251	SRR1967029	2014	6		Europe	United Kingdom	Enteritidis	Enteritidis	3900	25940
SAL_CA2943AA	38419	SRR1967028	2014	7	29	Europe	United Kingdom	Enteritidis	Enteritidis	3888	27844
SAL_CA2948AA	83587	SRR1967023	2015	1		Europe	United Kingdom	Enteritidis	Enteritidis	1425	27841
SAL_CA2952AA	63914	SRR1967019	2014	11	5	Europe	United Kingdom	Enteritidis	Enteritidis	1425	27838
SAL_CA2953AA	40800	SRR1967018	2014	8		Europe	United Kingdom	Enteritidis	Enteritidis	3888	27837
SAL_CA2959AA	32391	SRR1967012	2014	7		Europe	United Kingdom	Enteritidis	Enteritidis	1425	27831
SAL_CA2962AA	36381	SRR1967009	2014	8		Europe	United Kingdom	Enteritidis	Enteritidis	1425	27828
SAL_CA2965AA	73223	SRR1967006	2014	12	10	Europe	United Kingdom	Enteritidis	Enteritidis	28482	27825
SAL_CA2967AA	41993	SRR1967004	2014	8		Europe	United Kingdom	Enteritidis	Enteritidis	1425	27824
SAL_CA2969AA	36501	SRR1967002	2014	7	17	Europe	United Kingdom	Enteritidis	Enteritidis	3888	27822
SAL_CA2976AA	56157	SRR1966995	2014	9		Europe	United Kingdom	Enteritidis	Enteritidis	1425	27815
SAL_CA2986AA	37034	SRR1966985	2014	8		Europe	United Kingdom	Enteritidis	Enteritidis	3888	27805
SAL_CA2987AA	27067	SRR1966984	2014	7	7	Europe	United Kingdom	Enteritidis	Enteritidis	3888	674
SAL_CA2990AA	96973	SRR1966981	2015	3		Europe	United Kingdom	Enteritidis	Enteritidis	1425	7575
SAL_CA2993AA	36496	SRR1966978	2014	7		Europe	United Kingdom	Enteritidis	Enteritidis	3888	27800
SAL_CA2995AA	94269	SRR1966976	2015	3		Europe	United Kingdom	Enteritidis	Enteritidis	1425	27798
SAL_CA2997AA	45619	SRR1966974	2014	9		Europe	United Kingdom	Enteritidis	Enteritidis	26483	24903
SAL_CA2999AA	83701	SRR1966972	2015	1		Europe	United Kingdom	Enteritidis	Enteritidis	1425	27795
SAL_CA3000AA	38319	SRR1966971	2014	7	28	Europe	United Kingdom	Enteritidis	Enteritidis	3888	27794
SAL_CA3001AA	84458	SRR1966970	2015	2		Europe	United Kingdom	Enteritidis	Enteritidis	1425	27793
SAL_CA3002AA	38313	SRR1966969	2014	7	28	Europe	United Kingdom	Enteritidis	Enteritidis	3888	27792
SAL_CA3003AA	45621	SRR1966968	2014	9		Europe	United Kingdom	Enteritidis	Enteritidis	1425	27791
SAL_CA3004AA	65458	SRR1966967	2014	10		Europe	United Kingdom	Enteritidis	Enteritidis	3888	27790
SAL_CA3005AA	68974	SRR1966966	2014	11		Europe	United Kingdom	Enteritidis	Enteritidis	1425	27789
SAL_CA3007AA	90470	SRR1966964	2015	2		Europe	United Kingdom	Enteritidis	Enteritidis	1425	27787
SAL_CA3011AA	91216	SRR1966960	2015	2		Europe	United Kingdom	Enteritidis	Enteritidis	1425	651
SAL_CA3020AA	37758	SRR1966951	2014	8		Europe	United Kingdom	Enteritidis	Enteritidis	3888	27775
SAL_CA3025AA	36463	SRR1966946	2014	7		Europe	United Kingdom	Enteritidis	Enteritidis	1425	27771
SAL_CA3031AA	22227	SRR1966940	2014	6		Europe	United Kingdom	Enteritidis	Enteritidis	1425	27765
SAL_CA3033AA	65056	SRR1966938	2014	11		Europe	United Kingdom	Enteritidis	Enteritidis	1425	24563
SAL_CA3034AA	36525	SRR1966937	2014	7	24	Europe	United Kingdom	Enteritidis	Enteritidis	3888	27763
SAL_CA3036AA	14128	SRR1966935	2014	5	22	Europe	United Kingdom	Enteritidis	Enteritidis	3888	27761

SAL_CA3040AA	57106	SRR1966931	2014	8		Europe	United Kingdom	Enteritidis	Enteritidis	1425	27757
SAL_CA3043AA	34171	SRR1966928	2014	7	24	Europe	United Kingdom	Enteritidis	Enteritidis	3888	27755
SAL_CA3045AA	38414	SRR1966926	2014	8	6	Europe	United Kingdom	Enteritidis	Enteritidis	1425	27753
SAL_CA3048AA	37833	SRR1966923	2014	7	30	Europe	United Kingdom	Enteritidis	Enteritidis	3888	27750
SAL_CA3050AA	63039	SRR1966921	2014	10		Europe	United Kingdom	Enteritidis	Enteritidis	1425	27747
SAL_CA3052AA	14506	SRR1966919	2014	5		Europe	United Kingdom	Enteritidis	Enteritidis	3906	27745
SAL_CA3055AA	12129	SRR1966916	2014	5		Europe	United Kingdom	Enteritidis	Enteritidis	1425	27743
SAL_CA3060AA	73221	SRR1966911	2014	12	8	Europe	United Kingdom	Enteritidis	Enteritidis	3888	27738
SAL_CA3062AA	96978	SRR1966909	2015	3		Europe	United Kingdom	Enteritidis	Enteritidis	1425	27736
SAL_CA3068AA	80594	SRR1966903	2015	1		Europe	United Kingdom	Enteritidis	Enteritidis	1425	27731
SAL_CA3071AA	45616	SRR1966900	2014	9		Europe	United Kingdom	Enteritidis	Enteritidis	1425	27728
SAL_CA3072AA	21764	SRR1966899	2014	6		Europe	United Kingdom	Enteritidis	Enteritidis	1425	27727
SAL_CA3077AA	40279	SRR1966894	2014	8		Europe	United Kingdom	Enteritidis	Enteritidis	1425	27722
SAL_CA3078AA	41996	SRR1966893	2014	8		Europe	United Kingdom	Enteritidis	Enteritidis	3888	27721
SAL_CA3079AA	3743	SRR1966892	2014	4		Europe	United Kingdom	Enteritidis	Enteritidis	1425	27720
SAL_CA3087AA	63729	SRR1966884	2014	10		Europe	United Kingdom	Enteritidis	Enteritidis	1425	27712
SAL_CA3097AA	90514	SRR1966874	2015	2		Europe	United Kingdom	Enteritidis	Enteritidis	1425	27706
SAL_CA3105AA	94264	SRR1966866	2015	3		Europe	United Kingdom	Enteritidis	Enteritidis	1425	27700
SAL_CA3106AA	3753	SRR1966865	2014	4		Europe	United Kingdom	Enteritidis	Enteritidis	3888	27699
SAL_CA3108AA	50876	SRR1966863	2014	9		Europe	United Kingdom	Enteritidis	Enteritidis	3888	27697
SAL_CA3110AA	5878	SRR1966861	2014	4		Europe	United Kingdom	Enteritidis	Enteritidis	1425	20758
SAL_CA3113AA	21786	SRR1966858	2014	6	3	Europe	United Kingdom	Enteritidis	Enteritidis	3888	674
SAL_CA3114AA	89885	SRR1966857	2015	2		Europe	United Kingdom	Enteritidis	Enteritidis	1425	7683
SAL_CA3118AA	13804	SRR1966853	2014	5		Europe	United Kingdom	Enteritidis	Enteritidis	26481	27690
SAL_CA3120AA	34189	SRR1966851	2014	7	25	Europe	United Kingdom	Enteritidis	Enteritidis	3888	27688
SAL_CA3125AA	23496	SRR1966846	2014	6		Europe	United Kingdom	Enteritidis	Enteritidis	3888	27684
SAL_CA3141AA	32459	SRR1966830	2014	7	17	Europe	United Kingdom	Enteritidis	Enteritidis	3888	2066
SAL_CA3142AA	38362	SRR1966829	2014	8		Europe	United Kingdom	Enteritidis	Enteritidis	3888	27672
SAL_CA3146AA	40281	SRR1966825	2014	8		Europe	United Kingdom	Enteritidis	Enteritidis	26142	27669
SAL_CA3150AA	25325	SRR1966821	2014	7		Europe	United Kingdom	Enteritidis	Enteritidis	1425	27665
SAL_CA3153AA	63774	SRR1966818	2014	10		Europe	United Kingdom	Enteritidis	Enteritidis	3906	27662
SAL_CA3159AA	95046	SRR1966812	2015	3		Europe	United Kingdom	Enteritidis	Enteritidis	-1662	27657
SAL_CA3161AA	37117	SRR1966810	2014	8		Europe	United Kingdom	Enteritidis	Enteritidis	1425	27655
SAL_CA3165AA	63673	SRR1966806	2014	10		Europe	United Kingdom	Enteritidis	Enteritidis	1425	25482
SAL_CA3166AA	29388	SRR1966805	2014	7		Europe	United Kingdom	Enteritidis	Enteritidis	1425	27652
SAL_CA3167AA	23064	SRR1966804	2014	6		Europe	United Kingdom	Enteritidis	Enteritidis	1425	27651
SAL_CA3168AA	95018	SRR1966803	2015	3		Europe	United Kingdom	Enteritidis	Enteritidis	1425	27650
SAL_CA3174AA	12132	SRR1966797	2014	5		Europe	United Kingdom	Enteritidis	Enteritidis	1425	27645
SAL_CA3182AA	85736	SRR1966789	2015	2		Europe	United Kingdom	Enteritidis	Enteritidis	3888	27637
SAL_CA3190AA	90163	SRR1966781	2015	2		Europe	United Kingdom	Enteritidis	Enteritidis	1425	27630
SAL_CA3193AA	63579	SRR1966778	2014	10		Europe	United Kingdom	Enteritidis	Enteritidis	1425	27628
SAL_CA3196AA	96237	SRR1966775	2015	3		Europe	United Kingdom	Enteritidis	Enteritidis	1425	27625
SAL_CA3199AA	63703	SRR1966772	2014	10		Europe	United Kingdom	Enteritidis	Enteritidis	1425	27623
SAL_CA3200AA	84403	SRR1966771	2014	12		Europe	United Kingdom	Enteritidis	Enteritidis	1425	27622
SAL_CA3201AA	40283	SRR1966770	2014	8		Europe	United Kingdom	Enteritidis	Enteritidis	3888	27621
SAL_CA3206AA	36380	SRR1966765	2014	8		Europe	United Kingdom	Enteritidis	Enteritidis	1425	27616
SAL_CA3209AA	89094	SRR1966762	2015	2		Europe	United Kingdom	Enteritidis	Enteritidis	1425	27613
SAL_CA3216AA	38316	SRR1966755	2014	7	28	Europe	United Kingdom	Enteritidis	Enteritidis	3888	27608
SAL_CA3217AA	50354	SRR1966754	2014	9		Europe	United Kingdom	Enteritidis	Enteritidis	1425	27607
SAL_CA3221AA	50352	SRR1966750	2014	9		Europe	United Kingdom	Enteritidis	Enteritidis	1425	27603
SAL_CA3224AA	90510	SRR1966747	2015	2		Europe	United Kingdom	Enteritidis	Enteritidis	1425	27600
SAL_CA3226AA	37111	SRR1966745	2014	8	6	Europe	United Kingdom	Enteritidis	Enteritidis	3888	27599
SAL_CA3230AA	34173	SRR1966741	2014	7		Europe	United Kingdom	Enteritidis	Enteritidis	3888	27595
SAL_CA3233AA	65381	SRR1966738	2014	10		Europe	United Kingdom	Enteritidis	Enteritidis	1425	27592
SAL_CA3241AA	32469	SRR1966730	2014	7		Europe	United Kingdom	Enteritidis	Enteritidis	1425	27584
SAL_CA3247AA	34194	SRR1966724	2014	7	24	Europe	United Kingdom	Enteritidis	Enteritidis	3888	7153
SAL_CA3248AA	65019	SRR1966723	2014	11	4	Europe	United Kingdom	Enteritidis	Enteritidis	1425	27579
SAL_CA3253AA	63672	SRR1966718	2014	10		Europe	United Kingdom	Enteritidis	Enteritidis	3888	27574
SAL_CA3255AA	40217	SRR1966716	2014	8		Europe	United Kingdom	Enteritidis	Enteritidis	1425	27572

SAL_CA3256AA	38772	SRR1966715	2014	8	6	Europe	United Kingdom	Enteritidis	Enteritidis	3888	27571
SAL_CA3259AA	39507	SRR1966712	2014	8		Europe	United Kingdom	Enteritidis	Enteritidis	1425	27569
SAL_CA3269AA	63913	SRR1966702	2014	11		Europe	United Kingdom	Enteritidis	Enteritidis	1425	27560
SAL_CA3274AA	98257	SRR1966697	2015	3		Europe	United Kingdom	Enteritidis	Enteritidis	1425	2479
SAL_CA3276AA	75970	SRR1966695	2014	12		Europe	United Kingdom	Enteritidis	Enteritidis	1425	27555
SAL_CA3278AA	39438	SRR1966693	2014	6		Europe	United Kingdom	Enteritidis	Enteritidis	3888	27553
SAL_CA3279AA	39455	SRR1966692	2014	7	30	Europe	United Kingdom	Enteritidis	Enteritidis	3888	27552
SAL_CA3280AA	38346	SRR1966691	2014	8		Europe	United Kingdom	Enteritidis	Enteritidis	1425	27551
SAL_CA3281AA	55543	SRR1966690	2014	9		Europe	United Kingdom	Enteritidis	Enteritidis	1425	27550
SAL_CA3282AA	26635	SRR1966689	2014	7		Europe	United Kingdom	Enteritidis	Enteritidis	1425	27549
SAL_CA3285AA	63043	SRR1966686	2014	10		Europe	United Kingdom	Enteritidis	Enteritidis	3888	27547
SAL_CA3286AA	32470	SRR1966685	2014	7	17	Europe	United Kingdom	Enteritidis	Enteritidis	3888	27546
SAL_CA3287AA	9249	SRR1966684	2014	4		Europe	United Kingdom	Enteritidis	Enteritidis	1425	27545
SAL_CA3295AA	90496	SRR1966676	2015	2		Europe	United Kingdom	Enteritidis	Enteritidis	1425	27537
SAL_CA3299AA	87346	SRR1966672	2015	2		Europe	United Kingdom	Enteritidis	Enteritidis	1425	27533
SAL_CA3308AA	14508	SRR1966663	2014	5		Europe	United Kingdom	Enteritidis	Enteritidis	1425	27526
SAL_CA3312AA	40270	SRR1966659	2014	8		Europe	United Kingdom	Enteritidis	Enteritidis	1425	27522
SAL_CA3321AA	23080	SRR1966650	2014	6		Europe	United Kingdom	Enteritidis	Enteritidis	1425	27513
SAL_CA3322AA	41990	SRR1966649	2014	8		Europe	United Kingdom	Enteritidis	Enteritidis	3888	27512
SAL_CA3324AA	37816	SRR1966647	2014	8		Europe	United Kingdom	Enteritidis	Enteritidis	3888	24765
SAL_CA3325AA	27068	SRR1966646	2014	7		Europe	United Kingdom	Enteritidis	Enteritidis	1425	27510
SAL_CA3327AA	3740	SRR1966644	2014	4		Europe	United Kingdom	Enteritidis	Enteritidis	1425	27508
SAL_CA3328AA	94094	SRR1966643	2015	3		Europe	United Kingdom	Enteritidis	Enteritidis	1425	613
SAL_CA3337AA	68971	SRR1966633	2014	11		Europe	United Kingdom	Enteritidis	Enteritidis	1425	27500
SAL_CA3339AA	65049	SRR1966631	2014	11		Europe	United Kingdom	Enteritidis	Enteritidis	-1662	27498
SAL_CA3342AA	73134	SRR1966628	2014	12	1	Europe	United Kingdom	Enteritidis	Enteritidis	3888	27495
SAL_CA3343AA	41998	SRR1966627	2014	8		Europe	United Kingdom	Enteritidis	Enteritidis	1425	27494
SAL_CA3345AA	56112	SRR1966625	2014	9	12	Europe	United Kingdom	Enteritidis	Enteritidis	3888	27492
SAL_CA3347AA	67570	SRR1966623	2014	11		Europe	United Kingdom	Enteritidis	Enteritidis	1425	27381
SAL_CA3351AA	56151	SRR1966619	2014	9		Europe	United Kingdom	Enteritidis	Enteritidis	3888	27487
SAL_CA3359AA	83588	SRR1966609	2015	1	27	Europe	United Kingdom	Enteritidis	Enteritidis	1425	27480
SAL_CA3361AA	37004	SRR1966607	2014	8		Europe	United Kingdom	Enteritidis	Enteritidis	3906	27478
SAL_CA3363AA	36498	SRR1966605	2014	7	28	Europe	United Kingdom	Enteritidis	Enteritidis	3888	27476
SAL_CA3367AA	32465	SRR1966601	2014	7		Europe	United Kingdom	Enteritidis	Enteritidis	1425	27473
SAL_CA3372AA	42004	SRR1966596	2014	8		Europe	United Kingdom	Enteritidis	Enteritidis	1425	27469
SAL_CA3373AA	51262	SRR1966595	2014	9	17	Europe	United Kingdom	Enteritidis	Enteritidis	1425	27468
SAL_CA3376AA	93569	SRR1966592	2015	3		Europe	United Kingdom	Enteritidis	Enteritidis	28416	25524
SAL_CA3379AA	69283	SRR1966588	2014	11		Europe	United Kingdom	Enteritidis	Enteritidis	26465	27464
SAL_CA3380AA	87348	SRR1966587	2015	2		Europe	United Kingdom	Enteritidis	Enteritidis	26481	25998
SAL_CA3383AA	40293	SRR1966584	2014	8		Europe	United Kingdom	Enteritidis	Enteritidis	1425	27461
SAL_CA3384AA	5880	SRR1966583	2014	4		Europe	United Kingdom	Enteritidis	Enteritidis	1425	27460
SAL_CA3385AA	23473	SRR1966582	2014	6	13	Europe	United Kingdom	Enteritidis	Enteritidis	3888	27459
SAL_CA3388AA	31971	SRR1966579	2014	7		Europe	United Kingdom	Enteritidis	Enteritidis	1425	27457
SAL_CA3399AA	24056	SRR1966568	2014	6		Europe	United Kingdom	Enteritidis	Enteritidis	1425	27446
SAL_CA3408AA	63935	SRR1966559	2014	11		Europe	United Kingdom	Enteritidis	Enteritidis	1425	25143
SAL_CA3413AA	31922	SRR1966554	2014	7	21	Europe	United Kingdom	Enteritidis	Enteritidis	3888	7612
SAL_CA3414AA	3761	SRR1966553	2014	4		Europe	United Kingdom	Enteritidis	Enteritidis	3888	27433
SAL_CA3415AA	83354	SRR1966552	2015	1		Europe	United Kingdom	Enteritidis	Enteritidis	1425	27432
SAL_CA3421AA	63606	SRR1966546	2014	9		Europe	United Kingdom	Enteritidis	Enteritidis	1425	27427
SAL_CA3431AA	68972	SRR1966536	2014	11	11	Europe	United Kingdom	Enteritidis	Enteritidis	3888	7317
SAL_CA3433AA	39484	SRR1966534	2014	8		Europe	United Kingdom	Enteritidis	Enteritidis	3888	27417
SAL_CA3435AA	53090	SRR1966532	2014	9		Europe	United Kingdom	Enteritidis	Enteritidis	3888	27415
SAL_CA3442AA	38342	SRR1966525	2014	8		Europe	United Kingdom	Enteritidis	Enteritidis	1425	27410
SAL_CA3446AA	55581	SRR1966521	2014	9		Europe	United Kingdom	Enteritidis	Enteritidis	3888	7867
SAL_CA3454AA	37101	SRR1966512	2014	8		Europe	United Kingdom	Enteritidis	Enteritidis	1425	27404
SAL_CA3455AA	69882	SRR1966511	2014	11		Europe	United Kingdom	Enteritidis	Enteritidis	1425	27403
SAL_CA3456AA	14136	SRR1966510	2014	5		Europe	United Kingdom	Enteritidis	Enteritidis	3888	27402
SAL_CA3458AA	34200	SRR1966508	2014	7		Europe	United Kingdom	Enteritidis	Enteritidis	26465	27400
SAL_CA3459AA	37812	SRR1966507	2014	7	31	Europe	United Kingdom	Enteritidis	Enteritidis	3888	27399

SAL_CA3462AA	63934	SRR1966504	2014	11		Europe	United Kingdom	Enteritidis	Enteritidis	1425	27396
SAL_CA3464AA	36392	SRR1966502	2014	8	4	Europe	United Kingdom	Enteritidis	Enteritidis	3888	27394
SAL_CA3468AA	57162	SRR1966498	2014	10		Europe	United Kingdom	Enteritidis	Enteritidis	1425	27390
SAL_CA3469AA	69950	SRR1966497	2014	11		Europe	United Kingdom	Enteritidis	Enteritidis	1425	27389
SAL_CA3471AA	65376	SRR1966495	2014	10		Europe	United Kingdom	Enteritidis	Enteritidis	1425	6917
SAL_CA3475AA	37822	SRR1966491	2014	7	30	Europe	United Kingdom	Enteritidis	Enteritidis	3888	27384
SAL_CA3478AA	63912	SRR1966488	2014	11		Europe	United Kingdom	Enteritidis	Enteritidis	1425	27381
SAL_CA3479AA	83586	SRR1966487	2015	1		Europe	United Kingdom	Enteritidis	Enteritidis	1425	27380
SAL_CA3480AA	38318	SRR1966486	2014	7	28	Europe	United Kingdom	Enteritidis	Enteritidis	3888	27379
SAL_CA3484AA	21781	SRR1966482	2014	6	2	Europe	United Kingdom	Enteritidis	Enteritidis	3888	27375
SAL_CA3485AA	39451	SRR1966481	2014	8		Europe	United Kingdom	Enteritidis	Enteritidis	3906	27374
SAL_CA3488AA	68909	SRR1966478	2014	6	24	Europe	United Kingdom	Enteritidis	Enteritidis	3888	27371
SAL_CA3489AA	63906	SRR1966477	2014	11		Europe	United Kingdom	Enteritidis	Enteritidis	3888	27370
SAL_CA3494AA	63023	SRR1966472	2014	10		Europe	United Kingdom	Enteritidis	Enteritidis	1425	27365
SAL_CA3496AA	51275	SRR1966470	2014	9		Europe	United Kingdom	Enteritidis	Enteritidis	1425	27363
SAL_CA3497AA	73108	SRR1966469	2014	12		Europe	United Kingdom	Enteritidis	Enteritidis	1425	27362
SAL_CA3504AA	63620	SRR1966462	2014	9		Europe	United Kingdom	Enteritidis	Enteritidis	1425	27355
SAL_CA3505AA	61839	SRR1966461	2014	10		Europe	United Kingdom	Enteritidis	Enteritidis	1425	27354
SAL_CA3511AA	84446	SRR1966455	2015	2		Europe	United Kingdom	Enteritidis	Enteritidis	1425	2097
SAL_CA3513AA	51265	SRR1966453	2014	9		Europe	United Kingdom	Enteritidis	Enteritidis	3888	7810
SAL_CA3518AA	21778	SRR1966448	2014	6	12	Europe	United Kingdom	Enteritidis	Enteritidis	3888	674
SAL_CA3519AA	39449	SRR1966447	2014	7	21	Europe	United Kingdom	Enteritidis	Enteritidis	3888	27345
SAL_CA3524AA	57141	SRR1966442	2014	10		Europe	United Kingdom	Pensacola	Enteritidis	1425	27340
SAL_CA3536AA	51259	SRR1966430	2014	9		Europe	United Kingdom	Enteritidis	Enteritidis	1425	27329
SAL_CA3540AA	5921	SRR1966426	2014	4		Europe	United Kingdom	Enteritidis	Enteritidis	1425	27325
SAL_CA3544AA	12183	SRR1966422	2014	5		Europe	United Kingdom	Enteritidis	Enteritidis	1425	27321
SAL_CA3545AA	96954	SRR1966421	2015	3		Europe	United Kingdom	Enteritidis	Enteritidis	1425	27320
SAL_CA3546AA	63881	SRR1966420	2014	11		Europe	United Kingdom	Enteritidis	Enteritidis	3888	27319
SAL_CA3547AA	62984	SRR1966419	2014	9		Europe	United Kingdom	Enteritidis	Enteritidis	1425	27318
SAL_CA3550AA	91900	SRR1966417	2014	11		Europe	United Kingdom	Enteritidis	Enteritidis	1425	27315
SAL_CA3552AA	32393	SRR1966415	2014	7		Europe	United Kingdom	Enteritidis	Enteritidis	1425	27313
SAL_CA3561AA	51249	SRR1966406	2014	9		Europe	United Kingdom	Enteritidis	Enteritidis	1425	27304
SAL_CA3574AA	25264	SRR1966393	2014	7		Europe	United Kingdom	Enteritidis	Enteritidis	1425	27293
SAL_CA3576AA	27799	SRR1966391	2014	7		Europe	United Kingdom	Enteritidis	Enteritidis	1425	27291
SAL_CA3579AA	14125	SRR1966388	2014	5		Europe	United Kingdom	Enteritidis	Enteritidis	1425	27288
SAL_CA3580AA	31928	SRR1966387	2014	7	21	Europe	United Kingdom	Enteritidis	Enteritidis	3888	27287
SAL_CA3584AA	46585	SRR1966383	2014	7	18	Europe	United Kingdom	Enteritidis	Enteritidis	3888	27283
SAL_CA3586AA	23077	SRR1966381	2014	6		Europe	United Kingdom	Enteritidis	Enteritidis	1425	27281
SAL_CA3602AA	57105	SRR1966365	2014	8		Europe	United Kingdom	Enteritidis	Enteritidis	1425	27266
SAL_CA3604AA	27972	SRR1966363	2014	7		Europe	United Kingdom	Enteritidis	Enteritidis	1425	27264
SAL_CA3605AA	5354	SRR1966362	2014	4		Europe	United Kingdom	Enteritidis	Enteritidis	1425	27263
SAL_CA3606AA	63903	SRR1966361	2014	11		Europe	United Kingdom	Enteritidis	Enteritidis	1425	27262
SAL_CA3607AA	46574	SRR1966360	2014	7		Europe	United Kingdom	Enteritidis	Enteritidis	1425	27261
SAL_CA3611AA	41994	SRR1966356	2014	8		Europe	United Kingdom	Enteritidis	Enteritidis	1425	27257
SAL_CA3614AA	57138	SRR1966353	2014	10		Europe	United Kingdom	Enteritidis	Enteritidis	1425	27254
SAL_CA3615AA	38423	SRR1966352	2014	8		Europe	United Kingdom	Enteritidis	Enteritidis	3888	27253
SAL_CA3616AA	5355	SRR1966351	2014	4	2	Europe	United Kingdom	Enteritidis	Enteritidis	3888	7317
SAL_CA3618AA	36521	SRR1966349	2014	7	24	Europe	United Kingdom	Enteritidis	Enteritidis	3888	7612
SAL_CA3622AA	57113	SRR1966345	2014	8	5	Europe	United Kingdom	Enteritidis	Enteritidis	3888	27248
SAL_CA3623AA	39513	SRR1966344	2014	8		Europe	United Kingdom	Enteritidis	Enteritidis	1425	27247
SAL_CA3627AA	57163	SRR1966340	2014	10		Europe	United Kingdom	Enteritidis	Enteritidis	1425	27243
SAL_CA3628AA	34178	SRR1966339	2014	7	17	Europe	United Kingdom	Enteritidis	Enteritidis	3888	26229
SAL_CA3629AA	12186	SRR1966338	2014	5		Europe	United Kingdom	Enteritidis	Enteritidis	1425	27242
SAL_CA3630AA	7392	SRR1966337	2014	4		Europe	United Kingdom	Enteritidis	Enteritidis	1425	27241
SAL_CA3634AA	83723	SRR1966333	2015	1		Europe	United Kingdom	Enteritidis	Enteritidis	1425	27237
SAL_CA3635AA	40368	SRR1966332	2014	8		Europe	United Kingdom	Enteritidis	Enteritidis	1425	27236
SAL_CA3640AA	62995	SRR1966327	2014	9		Europe	United Kingdom	Enteritidis	Enteritidis	3888	27231
SAL_CA3642AA	57152	SRR1966325	2014	10		Europe	United Kingdom	Enteritidis	Enteritidis	1425	27229
SAL_CA3648AA	38353	SRR1966319	2014	8		Europe	United Kingdom	Enteritidis	Enteritidis	3888	27223

SAL_CA3649AA	21763	SRR1966318	2014	6		Europe	United Kingdom	Enteritidis	Enteritidis	3888	7105
SAL_CA3656AA	52956	SRR1966311	2014	9		Europe	United Kingdom	Enteritidis	Enteritidis	1425	27218
SAL_CA3658AA	50362	SRR1966309	2014	9		Europe	United Kingdom	Enteritidis	Enteritidis	3888	27216
SAL_CA3660AA	40794	SRR1966307	2014	8	12	Europe	United Kingdom	Enteritidis	Enteritidis	3888	27214
SAL_CA3667AA	50877	SRR1966300	2014	9		Europe	United Kingdom	Enteritidis	Enteritidis	1425	27209
SAL_CA3678AA	32477	SRR1966289	2014	7	17	Europe	United Kingdom	Enteritidis	Enteritidis	3888	27198
SAL_CA3679AA	42172	SRR1966288	2014	9		Europe	United Kingdom	Enteritidis	Enteritidis	3888	27197
SAL_CA3688AA	29381	SRR1966279	2014	7		Europe	United Kingdom	Enteritidis	Enteritidis	1425	27188
SAL_CA3690AA	37003	SRR1966277	2014	8		Europe	United Kingdom	Enteritidis	Enteritidis	28005	27186
SAL_CA3705AA	93571	SRR1966262	2015	3		Europe	United Kingdom	Enteritidis	Enteritidis	1425	27172
SAL_CA3707AA	25221	SRR1966260	2014	7		Europe	United Kingdom	Enteritidis	Enteritidis	1425	27170
SAL_CA3709AA	37108	SRR1966258	2014	8		Europe	United Kingdom	Enteritidis	Enteritidis	1425	27169
SAL_CA3715AA	34187	SRR1966252	2014	7	25	Europe	United Kingdom	Enteritidis	Enteritidis	3888	27163
SAL_CA3726AA	56152	SRR1966241	2014	9		Europe	United Kingdom	Enteritidis	Enteritidis	1425	27152
SAL_CA3727AA	31980	SRR1966240	2014	7	25	Europe	United Kingdom	Enteritidis	Enteritidis	3888	2066
SAL_CA3731AA	36532	SRR1966236	2014	8		Europe	United Kingdom	Enteritidis	Enteritidis	1425	27148
SAL_CA3734AA	65403	SRR1966233	2014	10		Europe	United Kingdom	Enteritidis	Enteritidis	3888	27145
SAL_CA3738AA	80605	SRR1966229	2015	1		Europe	United Kingdom	Enteritidis	Enteritidis	1425	27141
SAL_CA3742AA	46580	SRR1966225	2014	7	17	Europe	United Kingdom	Enteritidis	Enteritidis	3888	27137
SAL_CA3744AA	38308	SRR1966223	2014	8		Europe	United Kingdom	Enteritidis	Enteritidis	1425	27135
SAL_CA3748AA	56147	SRR1966219	2014	9		Europe	United Kingdom	Enteritidis	Enteritidis	1425	27131
SAL_CA3751AA	26633	SRR1966216	2014	7		Europe	United Kingdom	Enteritidis	Enteritidis	1425	27128
SAL_CA3752AA	50865	SRR1966215	2014	9		Europe	United Kingdom	Enteritidis	Enteritidis	1425	27127
SAL_CA3757AA	69947	SRR1966210	2014	11		Europe	United Kingdom	Enteritidis	Enteritidis	1425	27123
SAL_CA3759AA	85697	SRR1966208	2014	8		Europe	United Kingdom	Enteritidis	Enteritidis	1425	27121
SAL_CA3761AA	9464	SRR1966205	2014	4	29	Europe	United Kingdom	Enteritidis	Enteritidis	1425	27119
SAL_CA3766AA	83704	SRR1966200	2015	1		Europe	United Kingdom	Enteritidis	Enteritidis	1425	27114
SAL_CA3767AA	13860	SRR1966199	2014	5		Europe	United Kingdom	Enteritidis	Enteritidis	1425	27113
SAL_CA3771AA	9286	SRR1966195	2014	5	1	Europe	United Kingdom	Enteritidis	Enteritidis	3888	27109
SAL_CA3772AA	39453	SRR1966194	2014	7	29	Europe	United Kingdom	Enteritidis	Enteritidis	3888	27108
SAL_CA3773AA	63917	SRR1966193	2014	11		Europe	United Kingdom	Enteritidis	Enteritidis	1425	27107
SAL_CA3783AA	12117	SRR1966183	2014	5		Europe	United Kingdom	Enteritidis	Enteritidis	1425	27100
SAL_CA3787AA	63714	SRR1966179	2014	10		Europe	United Kingdom	Enteritidis	Enteritidis	1425	25107
SAL_CA3791AA	94096	SRR1966175	2015	3		Europe	United Kingdom	Enteritidis	Enteritidis	1425	27094
SAL_CA3794AA	27039	SRR1966171	2014	7		Europe	United Kingdom	Enteritidis	Enteritidis	3888	27091
SAL_CA3798AA	51278	SRR1966167	2014	9		Europe	United Kingdom	Enteritidis	Enteritidis	3888	27088
SAL_CA3801AA	27031	SRR1966164	2014	7		Europe	United Kingdom	Enteritidis	Enteritidis	1425	26210
SAL_CA3809AA	85735	SRR1966156	2015	2		Europe	United Kingdom	Enteritidis	Enteritidis	1425	27079
SAL_CA3814AA	31967	SRR1966151	2014	7	26	Europe	United Kingdom	Enteritidis	Enteritidis	3888	2066
SAL_CA3817AA	53105	SRR1966148	2014	9		Europe	United Kingdom	Enteritidis	Enteritidis	1425	25062
SAL_CA3831AA	52965	SRR1966134	2014	10		Europe	United Kingdom	Enteritidis	Enteritidis	1425	27062
SAL_CA3832AA	52963	SRR1966133	2014	10		Europe	United Kingdom	Enteritidis	Enteritidis	1425	27061
SAL_CA3836AA	37831	SRR1966129	2014	7	30	Europe	United Kingdom	Enteritidis	Enteritidis	3888	27057
SAL_CA3842AA	25188	SRR1966123	2014	7		Europe	United Kingdom	Enteritidis	Enteritidis	3888	27051
SAL_CA3845AA	36502	SRR1966120	2014	7		Europe	United Kingdom	Enteritidis	Enteritidis	1425	6886
SAL_CA3860AA	31973	SRR1966105	2014	7	22	Europe	United Kingdom	Enteritidis	Enteritidis	3888	27036
SAL_CA3862AA	25260	SRR1966102	2014	7	7	Europe	United Kingdom	Enteritidis	Enteritidis	3888	27034
SAL_CA3876AA	31650	SRR1966088	2014	7		Europe	United Kingdom	Enteritidis	Enteritidis	1425	27021
SAL_CA3877AA	32462	SRR1966087	2014	7	15	Europe	United Kingdom	Enteritidis	Enteritidis	3888	27020
SAL_CA3880AA	14875	SRR1966084	2014	5	20	Europe	United Kingdom	Enteritidis	Enteritidis	1425	27017
SAL_CA3881AA	38407	SRR1966083	2014	8		Europe	United Kingdom	Enteritidis	Enteritidis	26142	27016
SAL_CA3886AA	50867	SRR1966078	2014	9		Europe	United Kingdom	Enteritidis	Enteritidis	3888	27012
SAL_CA3887AA	36504	SRR1966077	2014	7	23	Europe	United Kingdom	Enteritidis	Enteritidis	3888	27011
SAL_CA3888AA	37835	SRR1966076	2014	7	28	Europe	United Kingdom	Enteritidis	Enteritidis	3888	27010
SAL_CA3890AA	65383	SRR1966074	2014	10		Europe	United Kingdom	Enteritidis	Enteritidis	1425	27008
SAL_CA3893AA	25224	SRR1966071	2014	7		Europe	United Kingdom	Enteritidis	Enteritidis	3888	27005
SAL_CA3895AA	45558	SRR1966069	2014	9		Europe	United Kingdom	Enteritidis	Enteritidis	1425	27003
SAL_CA3899AA	85696	SRR1966065	2014	8		Europe	United Kingdom	Enteritidis	Enteritidis	1425	27000
SAL_CA3900AA	60143	SRR1966064	2014	10		Europe	United Kingdom	Enteritidis	Enteritidis	1425	26999

SAL_CA3901AA	38778	SRR1966063	2014	8		Europe	United Kingdom	Enteritidis	Enteritidis	1425	26998
SAL_CA3912AA	96975	SRR1966052	2015	3		Europe	United Kingdom	Enteritidis	Enteritidis	3888	26987
SAL_CA3928AA	27004	SRR1966035	2014	7		Europe	United Kingdom	Enteritidis	Enteritidis	3888	25821
SAL_CA3930AA	56142	SRR1966033	2014	9		Europe	United Kingdom	Enteritidis	Enteritidis	1425	20758
SAL_CA3932AA	46014	SRR1966031	2014	8		Europe	United Kingdom	Enteritidis	Enteritidis	1425	26972
SAL_CA3934AA	26644	SRR1966029	2014	6	30	Europe	United Kingdom	Enteritidis	Enteritidis	3888	26971
SAL_CA3941AA	52953	SRR1966022	2014	9		Europe	United Kingdom	Enteritidis	Enteritidis	3888	26964
SAL_CA3942AA	94260	SRR1966021	2015	3		Europe	United Kingdom	Enteritidis	Enteritidis	1425	2163
SAL_CA3943AA	39486	SRR1966020	2014	8	4	Europe	United Kingdom	Enteritidis	Enteritidis	3888	26963
SAL_CA3947AA	40780	SRR1966016	2014	8		Europe	United Kingdom	Enteritidis	Enteritidis	3888	26960
SAL_CA3955AA	37112	SRR1966003	2014	8		Europe	United Kingdom	Enteritidis	Enteritidis	1425	26952
SAL_CA3956AA	23500	SRR1966002	2014	6	17	Europe	United Kingdom	Enteritidis	Enteritidis	3888	26951
SAL_CA3971AA	31976	SRR1965987	2014	7	23	Europe	United Kingdom	Enteritidis	Enteritidis	3888	26936
SAL_CA3973AA	27059	SRR1965985	2014	7		Europe	United Kingdom	Enteritidis	Enteritidis	1425	26934
SAL_CA3974AA	39512	SRR1965984	2014	8		Europe	United Kingdom	Enteritidis	Enteritidis	1425	26933
SAL_CA3976AA	9262	SRR1965982	2014	4		Europe	United Kingdom	Enteritidis	Enteritidis	1425	26931
SAL_CA3978AA	37837	SRR1965980	2014	8		Europe	United Kingdom	Enteritidis	Enteritidis	1425	26929
SAL_CA3983AA	24055	SRR1965975	2014	6		Europe	United Kingdom	Enteritidis	Enteritidis	1425	26924
SAL_CA3984AA	24041	SRR1965974	2014	6		Europe	United Kingdom	Enteritidis	Enteritidis	3888	26923
SAL_CA3985AA	14129	SRR1965973	2014	5		Europe	United Kingdom	Enteritidis	Enteritidis	3888	26922
SAL_CA3989AA	65436	SRR1965969	2014	10		Europe	United Kingdom	Enteritidis	Enteritidis	1425	26918
SAL_CA3995AA	83318	SRR1965963	2015	1	21	Europe	United Kingdom	Enteritidis	Enteritidis	1425	26913
SAL_CA3998AA	31649	SRR1965960	2014	7		Europe	United Kingdom	Enteritidis	Enteritidis	1425	26210
SAL_CA4000AA	34176	SRR1965958	2014	7	21	Europe	United Kingdom	Enteritidis	Enteritidis	3888	26909
SAL_CA4010AA	9245	SRR1965948	2014	4	23	Europe	United Kingdom	Enteritidis	Enteritidis	1425	26899
SAL_CA4020AA	96239	SRR1965938	2015	3	16	Europe	United Kingdom	Enteritidis	Enteritidis	1425	26890
SAL_CA4022AA	73213	SRR1965936	2014	12		Europe	United Kingdom	Enteritidis	Enteritidis	28459	26888
SAL_CA4026AA	39441	SRR1965932	2014	7		Europe	United Kingdom	Enteritidis	Enteritidis	1425	26884
SAL_CA4033AA	90473	SRR1965924	2015	2		Europe	United Kingdom	Enteritidis	Enteritidis	1425	26878
SAL_CA4040AA	63682	SRR1965917	2014	10		Europe	United Kingdom	Enteritidis	Enteritidis	3888	26872
SAL_CA4043AA	38339	SRR1965914	2014	8		Europe	United Kingdom	Enteritidis	Enteritidis	1425	26869
SAL_CA4045AA	68692	SRR1965912	2014	11	12	Europe	United Kingdom	Enteritidis	Enteritidis	27885	26868
SAL_CA4051AA	3907	SRR1965906	2014	4		Europe	United Kingdom	Enteritidis	Enteritidis	1425	26863
SAL_CA4053AA	51283	SRR1965904	2014	9		Europe	United Kingdom	Enteritidis	Enteritidis	1425	24766
SAL_CA4057AA	40792	SRR1965900	2014	8		Europe	United Kingdom	Enteritidis	Enteritidis	1425	26860
SAL_CA4061AA	31969	SRR1965895	2014	7	25	Europe	United Kingdom	Enteritidis	Enteritidis	3888	26858
SAL_CA4065AA	46576	SRR1965891	2014	7		Europe	United Kingdom	Enteritidis	Enteritidis	1425	26854
SAL_CA4069AA	12184	SRR1965887	2014	5		Europe	United Kingdom	Enteritidis	Enteritidis	1425	26851
SAL_CA4072AA	65369	SRR1965884	2014	10		Europe	United Kingdom	Enteritidis	Enteritidis	1425	26848
SAL_CA4073AA	83320	SRR1965883	2015	1		Europe	United Kingdom	Enteritidis	Enteritidis	1425	26847
SAL_CA4074AA	13372	SRR1965882	2014	4		Europe	United Kingdom	Enteritidis	Enteritidis	1425	26846
SAL_CA4077AA	91893	SRR1965879	2014	8	1	Europe	United Kingdom	Enteritidis	Enteritidis	3888	26844
SAL_CA4079AA	52976	SRR1965877	2014	10		Europe	United Kingdom	Enteritidis	Enteritidis	28454	26842
SAL_CA4080AA	63640	SRR1965876	2014	10		Europe	United Kingdom	Enteritidis	Enteritidis	1425	26841
SAL_CA4081AA	34158	SRR1965875	2014	7		Europe	United Kingdom	Enteritidis	Enteritidis	3888	26840
SAL_CA4084AA	63937	SRR1965872	2014	11		Europe	United Kingdom	Enteritidis	Enteritidis	1425	25223
SAL_CA4088AA	31952	SRR1965867	2014	7	24	Europe	United Kingdom	Enteritidis	Enteritidis	3888	512
SAL_CA4089AA	95044	SRR1965866	2015	3		Europe	United Kingdom	Enteritidis	Enteritidis	1425	26835
SAL_CA4095AA	38338	SRR1965860	2014	8		Europe	United Kingdom	Enteritidis	Enteritidis	1425	26830
SAL_CA4106AA	38350	SRR1965849	2014	8		Europe	United Kingdom	Enteritidis	Enteritidis	1425	26819
SAL_CA4107AA	63664	SRR1965848	2014	10		Europe	United Kingdom	Enteritidis	Enteritidis	1425	26818
SAL_CA4113AA	72310	SRR1965842	2014	11		Europe	United Kingdom	Enteritidis	Enteritidis	3888	26812
SAL_CA4124AA	39481	SRR1965831	2014	8		Europe	United Kingdom	Enteritidis	Enteritidis	1425	26802
SAL_CA4125AA	3745	SRR1965830	2014	4		Europe	United Kingdom	Enteritidis	Enteritidis	1425	7257
SAL_CA4130AA	45577	SRR1965824	2014	9		Europe	United Kingdom	Enteritidis	Enteritidis	1425	6917
SAL_CA4132AA	93530	SRR1965822	2015	3		Europe	United Kingdom	Enteritidis	Enteritidis	1425	26796
SAL_CA4141AA	61870	SRR1965813	2014	10		Europe	United Kingdom	Enteritidis	Enteritidis	1425	24829
SAL_CA4151AA	37808	SRR1965800	2014	7	31	Europe	United Kingdom	Enteritidis	Enteritidis	3888	7612
SAL_CA4152AA	69918	SRR1965799	2014	11		Europe	United Kingdom	Enteritidis	Enteritidis	1425	26780

SAL_CA4155AA	13429	SRR1965796	2014	5		Europe	United Kingdom	Enteritidis	Enteritidis	1425	26777
SAL_CA4157AA	14868	SRR1965794	2014	5		Europe	United Kingdom	Enteritidis	Enteritidis	3888	26776
SAL_CA4158AA	13358	SRR1965792	2014	4		Europe	United Kingdom	Enteritidis	Enteritidis	3888	26775
SAL_CA4160AA	25230	SRR1965790	2014	7		Europe	United Kingdom	Enteritidis	Enteritidis	1425	26773
SAL_CA4165AA	57112	SRR1965784	2014	8	1	Europe	United Kingdom	Enteritidis	Enteritidis	3888	26768
SAL_CA4168AA	53007	SRR1965781	2014	10	1	Europe	United Kingdom	Enteritidis	Enteritidis	1425	26765
SAL_CA4170AA	27036	SRR1965779	2014	7		Europe	United Kingdom	Enteritidis	Enteritidis	1425	26763
SAL_CA4172AA	57155	SRR1965776	2014	10		Europe	United Kingdom	Enteritidis	Enteritidis	3888	26761
SAL_CA4174AA	65379	SRR1965774	2014	9	29	Europe	United Kingdom	Enteritidis	Enteritidis	3888	26759
SAL_CA4175AA	25002	SRR1965773	2014	7		Europe	United Kingdom	Enteritidis	Enteritidis	1425	26758
SAL_CA4176AA	36522	SRR1965772	2014	7	23	Europe	United Kingdom	Enteritidis	Enteritidis	3888	26757
SAL_CA4181AA	24037	SRR1965767	2014	6		Europe	United Kingdom	Enteritidis	Enteritidis	1425	26752
SAL_CA4187AA	34140	SRR1965760	2014	7		Europe	United Kingdom	Enteritidis	Enteritidis	1425	26746
SAL_CA4189AA	63728	SRR1965758	2014	10		Europe	United Kingdom	Enteritidis	Enteritidis	3888	26744
SAL_CA4192AA	75938	SRR1965755	2014	12		Europe	United Kingdom	Enteritidis	Enteritidis	1425	26741
SAL_CA4194AA	46009	SRR1965753	2014	8	9	Europe	United Kingdom	Enteritidis	Enteritidis	3888	25112
SAL_CA4200AA	14507	SRR1965746	2014	5		Europe	United Kingdom	Enteritidis	Enteritidis	1425	26736
SAL_CA4203AA	40269	SRR1965742	2014	8	9	Europe	United Kingdom	Enteritidis	Enteritidis	3888	26733
SAL_CA4206AA	22230	SRR1965739	2014	6		Europe	United Kingdom	Enteritidis	Enteritidis	3888	26730
SAL_CA4207AA	25345	SRR1965738	2014	7		Europe	United Kingdom	Enteritidis	Enteritidis	3888	26729
SAL_CA4209AA	65449	SRR1965735	2014	8	1	Europe	United Kingdom	Enteritidis	Enteritidis	3888	26727
SAL_CA4213AA	37062	SRR1965730	2014	8	6	Europe	United Kingdom	Enteritidis	Enteritidis	3888	26723
SAL_CA4214AA	68636	SRR1965729	2014	10	13	Europe	United Kingdom	Enteritidis	Enteritidis	3888	7317
SAL_CA4217AA	63757	SRR1965726	2014	10		Europe	United Kingdom	Enteritidis	Enteritidis	3888	26720
SAL_CA4228AA	67566	SRR1965713	2014	11	11	Europe	United Kingdom	Enteritidis	Enteritidis	1425	26712
SAL_CA4232AA	98286	SRR1965709	2015	3		Europe	United Kingdom	Enteritidis	Enteritidis	1425	26709
SAL_CA4237AA	72325	SRR1965703	2014	11		Europe	United Kingdom	Enteritidis	Enteritidis	1425	7784
SAL_CA4240AA	37828	SRR1965700	2014	7	28	Europe	United Kingdom	Enteritidis	Enteritidis	3888	26702
SAL_CA4244AA	85726	SRR1965695	2015	2		Europe	United Kingdom	Enteritidis	Enteritidis	1425	26699
SAL_CA4247AA	38425	SRR1965692	2014	7	29	Europe	United Kingdom	Enteritidis	Enteritidis	3888	26696
SAL_CA4255AA	83342	SRR1965683	2015	1		Europe	United Kingdom	Enteritidis	Enteritidis	1425	26689
SAL_CA4257AA	65372	SRR1965681	2014	10		Europe	United Kingdom	Enteritidis	Enteritidis	1425	21622
SAL_CA4259AA	31929	SRR1965679	2014	7	21	Europe	United Kingdom	Enteritidis	Enteritidis	3888	26685
SAL_CA4266AA	63901	SRR1965671	2014	11		Europe	United Kingdom	Enteritidis	Enteritidis	26481	25998
SAL_CA4268AA	36506	SRR1965669	2014	7	24	Europe	United Kingdom	Enteritidis	Enteritidis	3888	26678
SAL_CA4279AA	93570	SRR1965657	2015	3		Europe	United Kingdom	Enteritidis	Enteritidis	1425	6917
SAL_CA4285AA	94083	SRR1965651	2015	3		Europe	United Kingdom	Enteritidis	Enteritidis	1425	26662
SAL_CA4299AA	99068	SRR1965636	2015	3	27	Europe	United Kingdom	Enteritidis	Enteritidis	1425	25745
SAL_CA4312AA	38421	SRR1965623	2014	7	29	Europe	United Kingdom	Enteritidis	Enteritidis	3888	26639
SAL_CA4315AA	42160	SRR1965620	2014	9		Europe	United Kingdom	Enteritidis	Enteritidis	1425	26636
SAL_CA4316AA	85728	SRR1965619	2015	2		Europe	United Kingdom	Enteritidis	Enteritidis	1425	26635
SAL_CA4317AA	36473	SRR1965618	2014	7	21	Europe	United Kingdom	Enteritidis	Enteritidis	3888	26634
SAL_CA4319AA	27061	SRR1965616	2014	7		Europe	United Kingdom	Enteritidis	Enteritidis	1425	26632
SAL_CA4320AA	38320	SRR1965615	2014	8		Europe	United Kingdom	Enteritidis	Enteritidis	1425	26631
SAL_CA4322AA	63598	SRR1965613	2014	10		Europe	United Kingdom	Enteritidis	Enteritidis	1425	24547
SAL_CA4326AA	9283	SRR1965609	2014	5		Europe	United Kingdom	Enteritidis	Enteritidis	1425	26627
SAL_CA4329AA	83343	SRR1965606	2015	1		Europe	United Kingdom	Enteritidis	Enteritidis	1425	6917
SAL_CA4332AA	45557	SRR1965603	2014	9		Europe	United Kingdom	Enteritidis	Enteritidis	1425	26622
SAL_CA4336AA	90471	SRR1965599	2015	2		Europe	United Kingdom	Enteritidis	Enteritidis	1425	26618
SAL_CA4339AA	38426	SRR1965596	2014	7	28	Europe	United Kingdom	Enteritidis	Enteritidis	3888	26616
SAL_CA4340AA	32519	SRR1965594	2014	7		Europe	United Kingdom	Enteritidis	Enteritidis	3888	26615
SAL_CA4343AA	38766	SRR1965590	2014	8	5	Europe	United Kingdom	Enteritidis	Enteritidis	3888	2066
SAL_CA4351AA	50351	SRR1965581	2014	9		Europe	United Kingdom	Enteritidis	Enteritidis	1425	26606
SAL_CA4352AA	21787	SRR1965580	2014	6		Europe	United Kingdom	Enteritidis	Enteritidis	1425	26605
SAL_CA4354AA	98254	SRR1965578	2015	3		Europe	United Kingdom	Enteritidis	Enteritidis	1425	2277
SAL_CA4355AA	93532	SRR1965577	2015	3		Europe	United Kingdom	Enteritidis	Enteritidis	3888	26603
SAL_CA4359AA	27974	SRR1965565	2014	7		Europe	United Kingdom	Enteritidis	Enteritidis	1425	26599
SAL_CA4360AA	29392	SRR1965564	2014	7		Europe	United Kingdom	Enteritidis	Enteritidis	3888	26598
SAL_CA4361AA	31977	SRR1965563	2014	4	22	Europe	United Kingdom	Enteritidis	Enteritidis	3888	514

SAL_CA4375AA	21777	SRR1965549	2014	6	12	Europe	United Kingdom	Enteritidis	Enteritidis	3888	26585
SAL_CA4377AA	75975	SRR1965547	2014	12		Europe	United Kingdom	Enteritidis	Enteritidis	1425	26583
SAL_CA4381AA	12159	SRR1965543	2014	5		Europe	United Kingdom	Enteritidis	Enteritidis	-1662	26580
SAL_CA4383AA	83287	SRR1965541	2015	1		Europe	United Kingdom	Enteritidis	Enteritidis	1425	26578
SAL_CA4386AA	75954	SRR1965538	2014	12		Europe	United Kingdom	Enteritidis	Enteritidis	1425	26575
SAL_CA4391AA	37819	SRR1965533	2014	7	29	Europe	United Kingdom	Enteritidis	Enteritidis	3888	2066
SAL_CA4394AA	37778	SRR1965530	2014	8	1	Europe	United Kingdom	Enteritidis	Enteritidis	3888	7612
SAL_CA4395AA	23495	SRR1965529	2014	6		Europe	United Kingdom	Enteritidis	Enteritidis	3888	26568
SAL_CA4397AA	23079	SRR1965527	2014	6		Europe	United Kingdom	Enteritidis	Enteritidis	1425	26566
SAL_CA4398AA	69951	SRR1965526	2014	11		Europe	United Kingdom	Enteritidis	Enteritidis	1425	26565
SAL_CA4399AA	61851	SRR1965525	2014	10		Europe	United Kingdom	Enteritidis	Enteritidis	1425	25842
SAL_CA4402AA	25321	SRR1965522	2014	6		Europe	United Kingdom	Enteritidis	Enteritidis	3888	26562
SAL_CA4404AA	68970	SRR1965520	2014	11		Europe	United Kingdom	Enteritidis	Enteritidis	1425	25432
SAL_CA4408AA	91295	SRR1965515	2015	2		Europe	United Kingdom	Enteritidis	Enteritidis	1425	26558
SAL_CA4409AA	25329	SRR1965514	2014	7		Europe	United Kingdom	Enteritidis	Enteritidis	3888	26557
SAL_CA4417AA	37832	SRR1965506	2014	7	31	Europe	United Kingdom	Enteritidis	Enteritidis	3888	26550
SAL_CA4418AA	32468	SRR1965505	2014	7		Europe	United Kingdom	Enteritidis	Enteritidis	1425	26549
SAL_CA4420AA	46010	SRR1965503	2014	8		Europe	United Kingdom	Enteritidis	Enteritidis	1425	26435
SAL_CA4427AA	38309	SRR1965496	2014	7	28	Europe	United Kingdom	Enteritidis	Enteritidis	3888	26542
SAL_CA4431AA	41987	SRR1965492	2014	8	27	Europe	United Kingdom	Enteritidis	Enteritidis	1425	26538
SAL_CA4433AA	37109	SRR1965488	2014	8		Europe	United Kingdom	Enteritidis	Enteritidis	1425	26536
SAL_CA4435AA	27001	SRR1965486	2014	6		Europe	United Kingdom	Enteritidis	Enteritidis	3888	26534
SAL_CA4437AA	43431	SRR1965484	2014	8	29	Europe	United Kingdom	Enteritidis	Enteritidis	3888	26532
SAL_CA4438AA	24053	SRR1965483	2014	6	19	Europe	United Kingdom	Enteritidis	Enteritidis	3888	26531
SAL_CA4440AA	14130	SRR1965481	2014	5		Europe	United Kingdom	Enteritidis	Enteritidis	1425	26529
SAL_CA4448AA	50890	SRR1965473	2014	9		Europe	United Kingdom	Hillingdon	Enteritidis	1425	7550
SAL_CA4450AA	60148	SRR1965471	2014	10		Europe	United Kingdom	Enteritidis	Enteritidis	1425	26521
SAL_CA4457AA	38311	SRR1965464	2014	7	29	Europe	United Kingdom	Enteritidis	Enteritidis	3888	26516
SAL_CA4458AA	85757	SRR1965463	2015	2		Europe	United Kingdom	Enteritidis	Enteritidis	1425	26515
SAL_CA4462AA	38418	SRR1965458	2014	7	30	Europe	United Kingdom	Enteritidis	Enteritidis	3888	26511
SAL_CA4463AA	68653	SRR1965457	2014	11		Europe	United Kingdom	Enteritidis	Enteritidis	1425	26283
SAL_CA4464AA	13803	SRR1965456	2014	4		Europe	United Kingdom	Enteritidis	Enteritidis	1425	26510
SAL_CA4466AA	50874	SRR1965454	2014	9		Europe	United Kingdom	Enteritidis	Enteritidis	1425	26508
SAL_CA4476AA	40327	SRR1965444	2014	8	18	Europe	United Kingdom	Enteritidis	Enteritidis	3888	26498
SAL_CA4481AA	27000	SRR1965439	2014	6		Europe	United Kingdom	Enteritidis	Enteritidis	1425	7007
SAL_CA4488AA	56120	SRR1965432	2014	9		Europe	United Kingdom	Enteritidis	Enteritidis	1425	26358
SAL_CA4496AA	37823	SRR1965424	2014	7	30	Europe	United Kingdom	Enteritidis	Enteritidis	3888	26481
SAL_CA4508AA	68651	SRR1965412	2014	11		Europe	United Kingdom	Enteritidis	Enteritidis	1425	26470
SAL_CA4509AA	65448	SRR1965411	2014	10	3	Europe	United Kingdom	Enteritidis	Enteritidis	3888	26469
SAL_CA4510AA	38420	SRR1965410	2014	7	29	Europe	United Kingdom	Enteritidis	Enteritidis	3888	26468
SAL_CA4512AA	39478	SRR1965408	2014	8		Europe	United Kingdom	Enteritidis	Enteritidis	28446	26466
SAL_CA4516AA	13808	SRR1965404	2014	5		Europe	United Kingdom	Enteritidis	Enteritidis	1425	26462
SAL_CA4517AA	63936	SRR1965403	2014	11		Europe	United Kingdom	Enteritidis	Enteritidis	1425	24842
SAL_CA4519AA	50854	SRR1965400	2014	9		Europe	United Kingdom	Enteritidis	Enteritidis	1425	26460
SAL_CA4520AA	96974	SRR1965399	2015	3		Europe	United Kingdom	Enteritidis	Enteritidis	1425	25914
SAL_CA4527AA	27802	SRR1965392	2014	7		Europe	United Kingdom	Enteritidis	Enteritidis	3888	26453
SAL_CA4534AA	57157	SRR1965385	2014	10		Europe	United Kingdom	Enteritidis	Enteritidis	1425	26446
SAL_CA4541AA	56145	SRR1965378	2014	9		Europe	United Kingdom	Enteritidis	Enteritidis	3888	7867
SAL_CA4542AA	94104	SRR1965377	2015	3		Europe	United Kingdom	Enteritidis	Enteritidis	1425	7921
SAL_CA4543AA	12153	SRR1965376	2014	5		Europe	United Kingdom	Enteritidis	Enteritidis	1425	26442
SAL_CA4544AA	32523	SRR1965375	2014	7	15	Europe	United Kingdom	Enteritidis	Enteritidis	3888	7153
SAL_CA4545AA	31939	SRR1965374	2014	7		Europe	United Kingdom	Enteritidis	Enteritidis	1425	25842
SAL_CA4547AA	65124	SRR1965372	2014	11		Europe	United Kingdom	Enteritidis	Enteritidis	1425	26440
SAL_CA4548AA	25267	SRR1965371	2014	7		Europe	United Kingdom	Enteritidis	Enteritidis	28443	26439
SAL_CA4550AA	38314	SRR1965369	2014	7	29	Europe	United Kingdom	Enteritidis	Enteritidis	3888	26438
SAL_CA4553AA	52949	SRR1965366	2014	10		Europe	United Kingdom	Enteritidis	Enteritidis	1425	26435
SAL_CA4559AA	45606	SRR1965360	2014	9	9	Europe	United Kingdom	Enteritidis	Enteritidis	1425	26429
SAL_CA4560AA	38361	SRR1965359	2014	7	28	Europe	United Kingdom	Enteritidis	Enteritidis	3888	2066
SAL_CA4566AA	50361	SRR1965353	2014	9	4	Europe	United Kingdom	Enteritidis	Enteritidis	3888	2066

SAL_CA4568AA	53127	SRR1965351	2014	9		Europe	United Kingdom	Enteritidis	Enteritidis	1425	26422
SAL_CA4570AA	93529	SRR1965349	2015	3		Europe	United Kingdom	Enteritidis	Enteritidis	28005	26420
SAL_CA4571AA	38352	SRR1965348	2014	8		Europe	United Kingdom	Enteritidis	Enteritidis	1425	26419
SAL_CA4574AA	14521	SRR1965345	2014	5		Europe	United Kingdom	Enteritidis	Enteritidis	1425	26417
SAL_CA4575AA	9186	SRR1965344	2014	5		Europe	United Kingdom	Enteritidis	Enteritidis	1425	3522
SAL_CA4580AA	37759	SRR1965339	2014	7	28	Europe	United Kingdom	Enteritidis	Enteritidis	3888	514
SAL_CA4582AA	13834	SRR1965336	2014	5		Europe	United Kingdom	Enteritidis	Enteritidis	26142	26411
SAL_CA4588AA	32527	SRR1965330	2014	7	23	Europe	United Kingdom	Enteritidis	Enteritidis	1425	26405
SAL_CA4591AA	52954	SRR1965327	2014	9	25	Europe	United Kingdom	Enteritidis	Enteritidis	3888	26402
SAL_CA4593AA	3749	SRR1965325	2014	4		Europe	United Kingdom	Enteritidis	Enteritidis	3888	26401
SAL_CA4598AA	99077	SRR1965320	2015	3		Europe	United Kingdom	Enteritidis	Enteritidis	1425	26397
SAL_CA4599AA	25183	SRR1965319	2014	7		Europe	United Kingdom	Enteritidis	Enteritidis	3888	26396
SAL_CA4601AA	84438	SRR1965317	2015	2		Europe	United Kingdom	Enteritidis	Enteritidis	1425	26394
SAL_CA4605AA	21785	SRR1965313	2014	6	3	Europe	United Kingdom	Enteritidis	Enteritidis	3888	26390
SAL_CA4615AA	55571	SRR1965303	2014	9		Europe	United Kingdom	Enteritidis	Enteritidis	1425	26381
SAL_CA4616AA	96956	SRR1965302	2015	3		Europe	United Kingdom	Enteritidis	Enteritidis	1425	26380
SAL_CA4628AA	39485	SRR1965290	2014	8		Europe	United Kingdom	Enteritidis	Enteritidis	1425	26368
SAL_CA4634AA	38771	SRR1965284	2014	8		Europe	United Kingdom	Enteritidis	Enteritidis	1425	26363
SAL_CA4636AA	85724	SRR1965282	2015	2		Europe	United Kingdom	Enteritidis	Enteritidis	1425	26361
SAL_CA4638AA	63599	SRR1965279	2014	10		Europe	United Kingdom	Enteritidis	Enteritidis	1425	26359
SAL_CA4639AA	75979	SRR1965278	2014	12		Europe	United Kingdom	Enteritidis	Enteritidis	1425	26358
SAL_CA4640AA	63577	SRR1965277	2014	10		Europe	United Kingdom	Enteritidis	Enteritidis	1425	26357
SAL_CA4642AA	53110	SRR1965275	2014	9	23	Europe	United Kingdom	Enteritidis	Enteritidis	3888	26355
SAL_CA4644AA	65365	SRR1965273	2014	10		Europe	United Kingdom	Enteritidis	Enteritidis	1425	24727
SAL_CA4648AA	63871	SRR1965269	2014	9		Europe	United Kingdom	Enteritidis	Enteritidis	1425	26350
SAL_CA4651AA	25341	SRR1965266	2014	6	25	Europe	United Kingdom	Enteritidis	Enteritidis	3888	26347
SAL_CA4657AA	46008	SRR1965260	2014	8		Europe	United Kingdom	Enteritidis	Enteritidis	1425	26342
SAL_CA4663AA	89998	SRR1965254	2015	2		Europe	United Kingdom	Enteritidis	Enteritidis	1425	23956
SAL_CA4664AA	45969	SRR1965253	2014	7		Europe	United Kingdom	Enteritidis	Enteritidis	1425	26337
SAL_CA4666AA	39510	SRR1965251	2014	8		Europe	United Kingdom	Enteritidis	Enteritidis	1425	26335
SAL_CA4669AA	93567	SRR1965248	2015	3		Europe	United Kingdom	Enteritidis	Enteritidis	1425	26333
SAL_CA4675AA	55567	SRR1965242	2014	9		Europe	United Kingdom	Enteritidis	Enteritidis	1425	26329
SAL_CA4680AA	53120	SRR1965237	2014	9		Europe	United Kingdom	Enteritidis	Enteritidis	1425	26325
SAL_CA4681AA	56130	SRR1965236	2014	9		Europe	United Kingdom	Enteritidis	Enteritidis	1425	26323
SAL_CA4683AA	50349	SRR1965234	2014	9		Europe	United Kingdom	Enteritidis	Enteritidis	3888	26322
SAL_CA4685AA	37763	SRR1965226	2014	8		Europe	United Kingdom	Enteritidis	Enteritidis	1425	26319
SAL_CA4688AA	65052	SRR1965222	2014	11		Europe	United Kingdom	Enteritidis	Enteritidis	26481	25998
SAL_CA4690AA	32395	SRR1965220	2014	7		Europe	United Kingdom	Enteritidis	Enteritidis	1425	26315
SAL_CA4691AA	76000	SRR1965219	2014	12		Europe	United Kingdom	Enteritidis	Enteritidis	1425	26314
SAL_CA4694AA	99786	SRR1965216	2015	3		Europe	United Kingdom	Enteritidis	Enteritidis	1425	26311
SAL_CA4697AA	50356	SRR1965213	2014	9	5	Europe	United Kingdom	Enteritidis	Enteritidis	3888	26308
SAL_CA4701AA	27800	SRR1965209	2014	7		Europe	United Kingdom	Enteritidis	Enteritidis	1425	26305
SAL_CA4703AA	31926	SRR1965207	2014	7		Europe	United Kingdom	Enteritidis	Enteritidis	3888	26303
SAL_CA4705AA	91259	SRR1965204	2015	2		Europe	United Kingdom	Enteritidis	Enteritidis	1425	26301
SAL_CA4713AA	50339	SRR1965196	2014	9		Europe	United Kingdom	Enteritidis	Enteritidis	1425	26295
SAL_CA4714AA	25186	SRR1965195	2014	7		Europe	United Kingdom	Enteritidis	Enteritidis	1425	26294
SAL_CA4719AA	3769	SRR1965190	2014	4		Europe	United Kingdom	Enteritidis	Enteritidis	3888	26289
SAL_CA4724AA	56155	SRR1965185	2014	9		Europe	United Kingdom	Enteritidis	Enteritidis	3888	26284
SAL_CA4725AA	72266	SRR1965184	2014	11		Europe	United Kingdom	Enteritidis	Enteritidis	1425	26283
SAL_CA4727AA	63907	SRR1965182	2014	11		Europe	United Kingdom	Enteritidis	Enteritidis	1425	26282
SAL_CA4733AA	98282	SRR1965175	2015	3		Europe	United Kingdom	Enteritidis	Enteritidis	1425	26278
SAL_CA4740AA	40799	SRR1965168	2014	8	15	Europe	United Kingdom	Enteritidis	Enteritidis	3888	26270
SAL_CA4741AA	65371	SRR1965167	2014	10		Europe	United Kingdom	Enteritidis	Enteritidis	1425	26271
SAL_CA4743AA	65368	SRR1965165	2014	10		Europe	United Kingdom	Enteritidis	Enteritidis	1425	26268
SAL_CA4753AA	65330	SRR1965155	2014	9		Europe	United Kingdom	Enteritidis	Enteritidis	1425	25842
SAL_CA4765AA	41997	SRR1965143	2014	8		Europe	United Kingdom	Enteritidis	Enteritidis	3888	26249
SAL_CA4766AA	38387	SRR1965142	2014	8		Europe	United Kingdom	Hillingdon	Enteritidis	1425	26248
SAL_CA4768AA	25217	SRR1965140	2014	7		Europe	United Kingdom	Enteritidis	Enteritidis	1425	26246
SAL_CA4770AA	34205	SRR1965138	2014	7		Europe	United Kingdom	Enteritidis	Enteritidis	1425	26244

SAL_CA4779AA	50837	SRR1965128	2014	9		Europe	United Kingdom	Enteritidis	Enteritidis	3888	26235
SAL_CA4780AA	60106	SRR1965127	2014	10		Europe	United Kingdom	Enteritidis	Enteritidis	1425	26234
SAL_CA4784AA	21762	SRR1965123	2014	6		Europe	United Kingdom	Enteritidis	Enteritidis	1425	26230
SAL_CA4785AA	32476	SRR1965122	2014	7	17	Europe	United Kingdom	Enteritidis	Enteritidis	3888	26229
SAL_CA4786AA	21768	SRR1965121	2014	6		Europe	United Kingdom	Enteritidis	Enteritidis	1425	26228
SAL_CA4789AA	40275	SRR1965117	2014	8		Europe	United Kingdom	Enteritidis	Enteritidis	3888	26225
SAL_CA4792AA	57102	SRR1965114	2014	8	11	Europe	United Kingdom	Enteritidis	Enteritidis	3888	26222
SAL_CA4797AA	6448	SRR1965109	2014	4		Europe	United Kingdom	Enteritidis	Enteritidis	1425	26218
SAL_CA4799AA	83355	SRR1965107	2015	1		Europe	United Kingdom	Enteritidis	Enteritidis	1425	26216
SAL_CA4800AA	63019	SRR1965106	2014	10		Europe	United Kingdom	Enteritidis	Enteritidis	3888	26215
SAL_CA4802AA	80562	SRR1965104	2015	1		Europe	United Kingdom	Enteritidis	Enteritidis	1425	7117
SAL_CA4805AA	63773	SRR1965101	2014	10		Europe	United Kingdom	Enteritidis	Enteritidis	3888	26212
SAL_CA4808AA	27069	SRR1965098	2014	7		Europe	United Kingdom	Enteritidis	Enteritidis	1425	26210
SAL_CA4812AA	40793	SRR1965094	2014	8	12	Europe	United Kingdom	Enteritidis	Enteritidis	3888	26206
SAL_CA4816AA	91218	SRR1965090	2015	2		Europe	United Kingdom	Enteritidis	Enteritidis	1425	26202
SAL_CA4820AA	38782	SRR1965086	2014	8		Europe	United Kingdom	Enteritidis	Enteritidis	1425	26198
SAL_CA4822AA	42000	SRR1965084	2014	8		Europe	United Kingdom	Enteritidis	Enteritidis	1425	26197
SAL_CA4828AA	37820	SRR1965078	2014	8		Europe	United Kingdom	Enteritidis	Enteritidis	1425	26191
SAL_CA4833AA	3788	SRR1965073	2014	4		Europe	United Kingdom	Enteritidis	Enteritidis	1425	26186
SAL_CA4837AA	34180	SRR1965068	2014	7	17	Europe	United Kingdom	Enteritidis	Enteritidis	3888	26182
SAL_CA4838AA	5357	SRR1965067	2014	4		Europe	United Kingdom	Enteritidis	Enteritidis	3888	26181
SAL_CA4849AA	36497	SRR1965055	2014	7		Europe	United Kingdom	Enteritidis	Enteritidis	3888	26172
SAL_CA4851AA	72321	SRR1965053	2014	12		Europe	United Kingdom	Enteritidis	Enteritidis	1425	25504
SAL_CA4860AA	34199	SRR1965044	2014	7	24	Europe	United Kingdom	Enteritidis	Enteritidis	3888	2066
SAL_CA4861AA	94267	SRR1965043	2015	3		Europe	United Kingdom	Enteritidis	Enteritidis	1425	26163
SAL_CA4862AA	38717	SRR1965042	2014	8		Europe	United Kingdom	Enteritidis	Enteritidis	3888	26162
SAL_CA4863AA	65459	SRR1965041	2014	10		Europe	United Kingdom	Enteritidis	Enteritidis	3888	26161
SAL_CA4889AA	78756	SRR1963533	2014	12		Europe	United Kingdom	Enteritidis	Enteritidis	1425	26140
SAL_CA4894AA	66868	SRR1963528	2014	10		Europe	United Kingdom	Enteritidis	Enteritidis	1425	26136
SAL_CA4898AA	69225	SRR1963524	2014	11		Europe	United Kingdom	Enteritidis	Enteritidis	3888	25681
SAL_CA4903AA	71405	SRR1963519	2012	11		Europe	United Kingdom	Enteritidis	Enteritidis	1425	26129
SAL_CA4910AA	78713	SRR1963512	2015	1		Europe	United Kingdom	Enteritidis	Enteritidis	1425	26124
SAL_CA4911AA	71577	SRR1963511	2014	12		Europe	United Kingdom	Enteritidis	Enteritidis	1425	7784
SAL_CA4912AA	69182	SRR1963510	2014	11		Europe	United Kingdom	Enteritidis	Enteritidis	1425	26123
SAL_CA4922AA	71410	SRR1963500	2012	11		Europe	United Kingdom	Enteritidis	Enteritidis	1425	26115
SAL_CA4927AA	71406	SRR1963495	2012	11		Europe	United Kingdom	Enteritidis	Enteritidis	1425	26110
SAL_CA4935AA	73654	SRR1963487	2014	12		Europe	United Kingdom	Enteritidis	Enteritidis	1425	24563
SAL_CA4936AA	66479	SRR1963486	2014	10		Europe	United Kingdom	Enteritidis	Enteritidis	1425	24563
SAL_CA4937AA	80364	SRR1963485	2015	1		Europe	United Kingdom	Enteritidis	Enteritidis	-1678	26102
SAL_CA4939AA	73553	SRR1963483	2014	9		Europe	United Kingdom	Enteritidis	Enteritidis	1425	26100
SAL_CA4942AA	70645	SRR1963480	2014	11		Europe	United Kingdom	Enteritidis	Enteritidis	1425	7784
SAL_CA4943AA	79105	SRR1963479	2015	1		Europe	United Kingdom	Enteritidis	Enteritidis	1425	26098
SAL_CA4945AA	80342	SRR1963477	2015	1		Europe	United Kingdom	Enteritidis	Enteritidis	1425	26097
SAL_CA4947AA	78670	SRR1963475	2014	12		Europe	United Kingdom	Enteritidis	Enteritidis	1425	25341
SAL_CA4952AA	71390	SRR1963470	2012	11		Europe	United Kingdom	Enteritidis	Enteritidis	1425	26091
SAL_CA4953AA	70646	SRR1963469	2014	11		Europe	United Kingdom	Enteritidis	Enteritidis	1425	26090
SAL_CA4954AA	80350	SRR1963468	2015	1		Europe	United Kingdom	Enteritidis	Enteritidis	1425	7210
SAL_CA4958AA	80348	SRR1963464	2015	1		Europe	United Kingdom	Enteritidis	Enteritidis	28119	26087
SAL_CA4966AA	69773	SRR1963456	2014	11		Europe	United Kingdom	Enteritidis	Enteritidis	1425	26079
SAL_CA4967AA	71386	SRR1963455	2012	11		Europe	United Kingdom	Enteritidis	Enteritidis	1425	26078
SAL_CA4968AA	73554	SRR1963454	2014	9	22	Europe	United Kingdom	Enteritidis	Enteritidis	1425	26077
SAL_CA4980AA	71398	SRR1963442	2012	11		Europe	United Kingdom	Enteritidis	Enteritidis	1425	26067
SAL_CA4981AA	78662	SRR1963441	2014	12		Europe	United Kingdom	Enteritidis	Enteritidis	1425	7043
SAL_CA4983AA	78697	SRR1963439	2014	12		Europe	United Kingdom	Enteritidis	Enteritidis	-1736	26064
SAL_CA4985AA	78759	SRR1963437	2014	12		Europe	United Kingdom	Enteritidis	Enteritidis	3888	404
SAL_CA4988AA	71403	SRR1963434	2012	11		Europe	United Kingdom	Enteritidis	Enteritidis	1425	26060
SAL_CA4990AA	79106	SRR1963432	2015	1		Europe	United Kingdom	Enteritidis	Enteritidis	1425	26058
SAL_CA4998AA	66885	SRR1963409	2014	10		Europe	United Kingdom	Enteritidis	Enteritidis	1425	16325
SAL_CA4999AA	66886	SRR1963408	2014	10		Europe	United Kingdom	Enteritidis	Enteritidis	1425	24879

SAL_CA5004AA	68899	SRR1963385	2014	10		Europe	United Kingdom	Enteritidis	Enteritidis	1425	24879
SAL_CA5007AA	71573	SRR1963382	2014	12		Europe	United Kingdom	Enteritidis	Enteritidis	1425	26047
SAL_CA5026AA	80295	SRR1963363	2014	12		Europe	United Kingdom	Enteritidis	Enteritidis	1425	25341
SAL_CA5027AA	79060	SRR1963361	2015	1		Europe	United Kingdom	Enteritidis	Enteritidis	1425	26030
SAL_CA5033AA	71402	SRR1963355	2012	11		Europe	United Kingdom	Enteritidis	Enteritidis	1425	26024
SAL_CA5039AA	79088	SRR1963349	2015	1		Europe	United Kingdom	Enteritidis	Enteritidis	1425	26018
SAL_CA5048AA	73580	SRR1963340	2014	9		Europe	United Kingdom	Enteritidis	Enteritidis	1425	26010
SAL_CA5049AA	74304	SRR1963339	2014	12		Europe	United Kingdom	Enteritidis	Enteritidis	1425	26009
SAL_CA5052AA	69216	SRR1963335	2014	11		Europe	United Kingdom	Enteritidis	Enteritidis	26465	26006
SAL_CA5061AA	69697	SRR1963326	2014	11		Europe	United Kingdom	Enteritidis	Enteritidis	1425	24879
SAL_CA5063AA	69749	SRR1963324	2014	11		Europe	United Kingdom	Enteritidis	Enteritidis	26481	25998
SAL_CA5065AA	71389	SRR1963322	2012	11		Europe	United Kingdom	Enteritidis	Enteritidis	1425	25997
SAL_CA5066AA	78968	SRR1963321	2015	1		Europe	United Kingdom	Enteritidis	Enteritidis	1425	25996
SAL_CA5067AA	79109	SRR1963320	2015	1		Europe	United Kingdom	Enteritidis	Enteritidis	1425	25808
SAL_CA5068AA	75675	SRR1963319	2014	12		Europe	United Kingdom	Enteritidis	Enteritidis	1425	25995
SAL_CA5073AA	71560	SRR1963314	2014	12		Europe	United Kingdom	Enteritidis	Enteritidis	1425	25990
SAL_CA5084AA	79112	SRR1963303	2015	1		Europe	United Kingdom	Enteritidis	Enteritidis	3888	25602
SAL_CA5090AA	78737	SRR1963297	2014	12		Europe	United Kingdom	Enteritidis	Enteritidis	1425	25976
SAL_CA5094AA	78706	SRR1963293	2014	12		Europe	United Kingdom	Enteritidis	Enteritidis	1425	25973
SAL_CA5101AA	73579	SRR1963286	2014	9		Europe	United Kingdom	Enteritidis	Enteritidis	1425	25968
SAL_CA5102AA	78755	SRR1963285	2014	12		Europe	United Kingdom	Enteritidis	Enteritidis	3888	404
SAL_CA5104AA	74310	SRR1963283	2014	12		Europe	United Kingdom	Enteritidis	Enteritidis	1425	25966
SAL_CA5107AA	68897	SRR1963280	2014	10		Europe	United Kingdom	Enteritidis	Enteritidis	3888	24864
SAL_CA5108AA	73606	SRR1963279	2014	12		Europe	United Kingdom	Enteritidis	Enteritidis	1425	25963
SAL_CA5115AA	69699	SRR1963272	2014	11	12	Europe	United Kingdom	Enteritidis	Enteritidis	3888	24946
SAL_CA5117AA	81174	SRR1963270	2015	1		Europe	United Kingdom	Enteritidis	Enteritidis	1425	25956
SAL_CA5118AA	70666	SRR1963269	2014	11	27	Europe	United Kingdom	Enteritidis	Enteritidis	1425	25955
SAL_CA5123AA	73575	SRR1963264	2014	9		Europe	United Kingdom	Enteritidis	Enteritidis	1425	25305
SAL_CA5132AA	73577	SRR1963255	2014	9		Europe	United Kingdom	Enteritidis	Enteritidis	1425	7710
SAL_CA5138AA	70663	SRR1963249	2014	11		Europe	United Kingdom	Enteritidis	Enteritidis	3900	25940
SAL_CA5139AA	66883	SRR1963248	2014	10		Europe	United Kingdom	Enteritidis	Enteritidis	1425	7043
SAL_CA5143AA	80323	SRR1963244	2015	1		Europe	United Kingdom	Enteritidis	Enteritidis	3888	3637
SAL_CA5145AA	43417	SRR1963242	2014	9		Europe	United Kingdom	Enteritidis	Enteritidis	1425	25936
SAL_CA5148AA	69212	SRR1963233	2014	11		Europe	United Kingdom	Enteritidis	Enteritidis	1425	25933
SAL_CA5149AA	79111	SRR1963232	2015	1		Europe	United Kingdom	Enteritidis	Enteritidis	1425	25932
SAL_CA5151AA	71595	SRR1963230	2014	12		Europe	United Kingdom	Enteritidis	Enteritidis	1425	25930
SAL_CA5169AA	69767	SRR1963145	2014	11		Europe	United Kingdom	Enteritidis	Enteritidis	1425	25918
SAL_CA5175AA	78615	SRR1963118	2014	12		Europe	United Kingdom	Enteritidis	Enteritidis	1425	25914
SAL_CA5176AA	71393	SRR1963117	2012	11		Europe	United Kingdom	Enteritidis	Enteritidis	1425	25913
SAL_CA5193AA	80322	SRR1963100	2015	1		Europe	United Kingdom	Enteritidis	Enteritidis	3888	25900
SAL_CA5196AA	71575	SRR1963097	2014	12		Europe	United Kingdom	Enteritidis	Enteritidis	1425	25897
SAL_CA5199AA	70622	SRR1963094	2014	8		Europe	United Kingdom	Enteritidis	Enteritidis	27796	25894
SAL_CA5203AA	69227	SRR1963090	2014	11		Europe	United Kingdom	Enteritidis	Enteritidis	1425	25859
SAL_CA5206AA	70700	SRR1963087	2014	12		Europe	United Kingdom	Enteritidis	Enteritidis	1425	25888
SAL_CA5207AA	70661	SRR1963086	2014	11		Europe	United Kingdom	Enteritidis	Enteritidis	3888	25887
SAL_CA5209AA	80345	SRR1963084	2015	1		Europe	United Kingdom	Enteritidis	Enteritidis	3906	25663
SAL_CA5212AA	69209	SRR1963081	2014	11		Europe	United Kingdom	Enteritidis	Enteritidis	1425	25884
SAL_CA5215AA	70696	SRR1963078	2014	12		Europe	United Kingdom	Enteritidis	Enteritidis	1425	25881
SAL_CA5219AA	71538	SRR1963074	2014	12		Europe	United Kingdom	Enteritidis	Enteritidis	1425	25878
SAL_CA5222AA	74309	SRR1963071	2014	11	27	Europe	United Kingdom	Enteritidis	Enteritidis	3888	7317
SAL_CA5226AA	74303	SRR1963067	2014	12		Europe	United Kingdom	Enteritidis	Enteritidis	1425	25873
SAL_CA5230AA	69701	SRR1962576	2014	11		Europe	United Kingdom	Enteritidis	Enteritidis	3888	25870
SAL_CA5233AA	70697	SRR1962558	2014	12		Europe	United Kingdom	Enteritidis	Enteritidis	1425	25868
SAL_CA5238AA	70693	SRR1962530	2014	12		Europe	United Kingdom	Enteritidis	Enteritidis	3888	25681
SAL_CA5243AA	69750	SRR1962403	2014	11		Europe	United Kingdom	Enteritidis	Enteritidis	1425	25859
SAL_CA5249AA	71594	SRR1962287	2014	12		Europe	United Kingdom	Enteritidis	Enteritidis	1425	25853
SAL_CA5250AA	78758	SRR1962286	2014	12		Europe	United Kingdom	Enteritidis	Enteritidis	1425	25852
SAL_CA5251AA	71585	SRR1962285	2014	12		Europe	United Kingdom	Enteritidis	Enteritidis	3888	25851
SAL_CA5256AA	66880	SRR1962067	2014	10		Europe	United Kingdom	Enteritidis	Enteritidis	1425	24879

SAL_CA5257AA	69195	SRR1962045	2014	11		Europe	United Kingdom	Enteritidis	Enteritidis	1425	25846
SAL_CA5260AA	69748	SRR1962042	2014	11		Europe	United Kingdom	Enteritidis	Enteritidis	1425	25843
SAL_CA5261AA	68895	SRR1962041	2014	10		Europe	United Kingdom	Enteritidis	Enteritidis	1425	25842
SAL_CA5264AA	73605	SRR1961943	2014	12		Europe	United Kingdom	Enteritidis	Enteritidis	1425	25840
SAL_CA5267AA	71399	SRR1961830	2012	11		Europe	United Kingdom	Enteritidis	Enteritidis	1425	25838
SAL_CA5272AA	78757	SRR1961770	2014	12		Europe	United Kingdom	Enteritidis	Enteritidis	3897	25834
SAL_CA5273AA	69700	SRR1961769	2014	11		Europe	United Kingdom	Enteritidis	Enteritidis	1425	25833
SAL_CA5275AA	71407	SRR1961727	2012	11		Europe	United Kingdom	Enteritidis	Enteritidis	1425	25831
SAL_CA5276AA	73627	SRR1961686	2014	12		Europe	United Kingdom	Enteritidis	Enteritidis	1425	25830
SAL_CA5284AA	66443	SRR1961506	2014	10		Europe	United Kingdom	Enteritidis	Enteritidis	1425	24879
SAL_CA5285AA	73535	SRR1961505	2014	9	19	Europe	United Kingdom	Enteritidis	Enteritidis	1425	50869
SAL_CA5287AA	66884	SRR1961503	2014	10		Europe	United Kingdom	Enteritidis	Enteritidis	3888	25821
SAL_CA5290AA	71597	SRR1961080	2014	12		Europe	United Kingdom	Enteritidis	Enteritidis	1425	25818
SAL_CA5293AA	73544	SRR1960997	2014	9		Europe	United Kingdom	Enteritidis	Enteritidis	1425	25816
SAL_CA5295AA	69741	SRR1960986	2014	11		Europe	United Kingdom	Enteritidis	Enteritidis	3888	25814
SAL_CA5298AA	66882	SRR1960983	2014	10		Europe	United Kingdom	Enteritidis	Enteritidis	1425	24563
SAL_CA5301AA	79090	SRR1960954	2015	1		Europe	United Kingdom	Enteritidis	Enteritidis	1425	25809
SAL_CA5302AA	80341	SRR1960928	2015	1	13	Europe	United Kingdom	Enteritidis	Enteritidis	1425	25808
SAL_CA5305AA	80304	SRR1960843	2015	1		Europe	United Kingdom	Enteritidis	Enteritidis	3888	25805
SAL_CA5315AA	80363	SRR1960665	2015	1	14	Europe	United Kingdom	Enteritidis	Enteritidis	1425	25798
SAL_CA5325AA	70659	SRR1960410	2014	11		Europe	United Kingdom	Enteritidis	Enteritidis	3888	25681
SAL_CA5328AA	73655	SRR1960384	2014	12		Europe	United Kingdom	Enteritidis	Enteritidis	1425	25788
SAL_CA5334AA	69702	SRR1960378	2014	11	19	Europe	United Kingdom	Enteritidis	Enteritidis	1425	25786
SAL_CA5338AA	78730	SRR1960374	2014	12		Europe	United Kingdom	Enteritidis	Enteritidis	1425	25782
SAL_CA5341AA	71391	SRR1960370	2012	11		Europe	United Kingdom	Enteritidis	Enteritidis	3888	25779
SAL_CA5358AA	73657	SRR1960353	2014	12		Europe	United Kingdom	Enteritidis	Enteritidis	1425	25763
SAL_CA5361AA	80367	SRR1960341	2015	1		Europe	United Kingdom	Enteritidis	Enteritidis	1425	25546
SAL_CA5363AA	69224	SRR1960326	2014	11		Europe	United Kingdom	Enteritidis	Enteritidis	1425	25761
SAL_CA5365AA	80339	SRR1960324	2015	1		Europe	United Kingdom	Enteritidis	Enteritidis	1425	25759
SAL_CA5370AA	73583	SRR1960319	2014	9		Europe	United Kingdom	Enteritidis	Enteritidis	3888	25755
SAL_CA5371AA	PNUSAS000259	SRR1960318				North America	United States	Enteritidis (Predicted)	Enteritidis	1425	25754
SAL_CA5372AA	PNUSAS000258	SRR1960317				North America	United States	Enteritidis (Predicted)	Enteritidis	7801	25753
SAL_CA5373AA	PNUSAS000257	SRR1960316				North America	United States	Enteritidis (Predicted)	Enteritidis	1425	16644
SAL_CA5377AA	71388	SRR1960312	2012	11		Europe	United Kingdom	Enteritidis	Enteritidis	1425	25749
SAL_CA5380AA	PNUSAS000255	SRR1960309				North America	United States	Enteritidis (Predicted)	Enteritidis	1425	25746
SAL_CA5381AA	PNUSAS000256	SRR1960308	2015	3		North America	United States	Enteritidis	Enteritidis	1425	25745
SAL_CA5382AA	PNUSAS000253	SRR1960307	2015	2		North America	United States	Enteritidis	Enteritidis	8003	25744
SAL_CA5385AA	PNUSAS000252	SRR1960304	2015	3		North America	United States	Enteritidis	Enteritidis	1425	25741
SAL_CA5386AA	PNUSAS000251	SRR1960303	2015	3		North America	United States	Enteritidis	Enteritidis	1425	12315
SAL_CA5387AA	PNUSAS000249	SRR1960302	2015	3		North America	United States	Enteritidis	Enteritidis	1425	25740
SAL_CA5388AA	PNUSAS000250	SRR1960301	2015	3		North America	United States	Enteritidis	Enteritidis	1425	25739
SAL_CA5389AA	PNUSAS000248	SRR1960300	2015	3		North America	United States	Enteritidis	Enteritidis	27901	25738
SAL_CA5390AA	PNUSAS000247	SRR1960299	2015	3		North America	United States	Enteritidis	Enteritidis	1425	25737
SAL_CA5391AA	PNUSAS000246	SRR1960298	2015	3		North America	United States	Enteritidis	Enteritidis	1425	25736
SAL_CA5392AA	69705	SRR1960297	2014	11		Europe	United Kingdom	Enteritidis	Enteritidis	1425	25735
SAL_CA5395AA	80344	SRR1960294	2015	1		Europe	United Kingdom	Enteritidis	Enteritidis	3906	25663
SAL_CA5398AA	PNUSAS000245	SRR1960291	2015	3		North America	United States	Enteritidis	Enteritidis	1425	25730
SAL_CA5400AA	PNUSAS000244	SRR1960289				North America	United States	Enteritidis (Predicted)	Enteritidis	1425	25728
SAL_CA5401AA	PNUSAS000243	SRR1960288				North America	United States	Enteritidis (Predicted)	Enteritidis	1425	25727
SAL_CA5402AA	PNUSAS000242	SRR1960287	2015	3		North America	United States	Enteritidis	Enteritidis	1425	25726
SAL_CA5403AA	PNUSAS000241	SRR1960286	2015	2		North America	United States	Enteritidis	Enteritidis	1425	25725
SAL_CA5409AA	PNUSAS000215	SRR1960280	2014	12		North America	United States	Enteritidis	Enteritidis	1425	23798
SAL_CA5414AA	78958	SRR1960275	2015	1		Europe	United Kingdom	Enteritidis	Enteritidis	1425	25717
SAL_CA5416AA	70694	SRR1960273	2014	12		Europe	United Kingdom	Enteritidis	Enteritidis	3888	25715
SAL_CA5420AA	PNUSAS000194	SRR1960269				North America	United States	Enteritidis (Predicted)	Enteritidis	1425	2668
SAL_CA5421AA	69218	SRR1960268	2014	11		Europe	United Kingdom	Enteritidis	Enteritidis	1425	25711
SAL_CA5422AA	PNUSAS000193	SRR1960267	2015	1		North America	United States	Enteritidis	Enteritidis	1425	25710
SAL_CA5423AA	PNUSAS000192	SRR1960266	2015	1		North America	United States	Enteritidis	Enteritidis	8003	25709
SAL_CA5424AA	PNUSAS000191	SRR1960265	2015	1		North America	United States	Enteritidis	Enteritidis	8003	18361

SAL_CA5426AA	PNUSAS000190	SRR1960263	2015	1		North America	United States	Enteritidis (Predicted)	Enteritidis	1425	25707
SAL_CA5433AA	79084	SRR1960256	2015	1		Europe	United Kingdom	Enteritidis	Enteritidis	1425	25700
SAL_CA5436AA	79068	SRR1960253	2015	1		Europe	United Kingdom	Enteritidis	Enteritidis	1425	25697
SAL_CA5440AA	71387	SRR1960249	2012	11		Europe	United Kingdom	Enteritidis	Enteritidis	1425	25693
SAL_CA5441AA	70653	SRR1960241	2014	11		Europe	United Kingdom	Enteritidis	Enteritidis	3888	25692
SAL_CA5445AA	69222	SRR1960237	2014	11		Europe	United Kingdom	Enteritidis	Enteritidis	3888	25688
SAL_CA5446AA	73639	SRR1960236	2014	12	10	Europe	United Kingdom	Enteritidis	Enteritidis	1425	25687
SAL_CA5448AA	78718	SRR1960234	2014	12		Europe	United Kingdom	Enteritidis	Enteritidis	1425	7043
SAL_CA5449AA	69198	SRR1960233	2014	11		Europe	United Kingdom	Enteritidis	Enteritidis	1425	25482
SAL_CA5450AA	75674	SRR1960232	2014	12		Europe	United Kingdom	Enteritidis	Enteritidis	1425	25685
SAL_CA5454AA	71584	SRR1960228	2014	12		Europe	United Kingdom	Enteritidis	Enteritidis	3888	25681
SAL_CA5455AA	78622	SRR1960227	2014	12		Europe	United Kingdom	Enteritidis	Enteritidis	1425	25680
SAL_CA5459AA	71395	SRR1960223	2012	11		Europe	United Kingdom	Enteritidis	Enteritidis	1425	25677
SAL_CA5460AA	75759	SRR1960222	2014	12		Europe	United Kingdom	Enteritidis	Enteritidis	3941	25676
SAL_CA5461AA	70647	SRR1960221	2014	11		Europe	United Kingdom	Enteritidis	Enteritidis	1425	25675
SAL_CA5471AA	69219	SRR1960211	2014	11		Europe	United Kingdom	Enteritidis	Enteritidis	1425	2067
SAL_CA5472AA	69211	SRR1960210	2014	11		Europe	United Kingdom	Enteritidis	Enteritidis	1425	25666
SAL_CA5475AA	79082	SRR1960207	2015	1		Europe	United Kingdom	Enteritidis	Enteritidis	3906	25663
SAL_CA5477AA	78664	SRR1960205	2014	12		Europe	United Kingdom	Enteritidis	Enteritidis	1425	25661
SAL_CA5490AA	71401	SRR1960192	2012	11		Europe	United Kingdom	Enteritidis	Enteritidis	1425	25649
SAL_CA5491AA	74270	SRR1960191	2014	11		Europe	United Kingdom	Enteritidis	Enteritidis	1425	25648
SAL_CA5501AA	73622	SRR1960181	2014	12		Europe	United Kingdom	Enteritidis	Enteritidis	1425	25638
SAL_CA5507AA	78695	SRR1960166	2014	12	24	Europe	United Kingdom	Enteritidis	Enteritidis	1425	25635
SAL_CA5517AA	71404	SRR1960145	2012	11		Europe	United Kingdom	Enteritidis	Enteritidis	1425	25629
SAL_CA5519AA	80324	SRR1960143	2015	1		Europe	United Kingdom	Enteritidis	Enteritidis	3888	3635
SAL_CA5526AA	80353	SRR1960136	2015	1		Europe	United Kingdom	Enteritidis	Enteritidis	1425	25388
SAL_CA5528AA	78668	SRR1960134	2014	12		Europe	United Kingdom	Enteritidis	Enteritidis	1425	25621
SAL_CA5544AA	69772	SRR1960095	2014	11		Europe	United Kingdom	Enteritidis	Enteritidis	1425	25607
SAL_CA5547AA	80351	SRR1960092	2015	1		Europe	United Kingdom	Enteritidis	Enteritidis	3888	25605
SAL_CA5548AA	78719	SRR1960091	2015	1		Europe	United Kingdom	Enteritidis	Enteritidis	1425	25604
SAL_CA5550AA	79107	SRR1960089	2015	1		Europe	United Kingdom	Enteritidis	Enteritidis	3888	25602
SAL_CA5551AA	73572	SRR1960088	2014	9		Europe	United Kingdom	Enteritidis	Enteritidis	1425	25601
SAL_CA5556AA	70668	SRR1960083	2014	11		Europe	United Kingdom	Enteritidis	Enteritidis	1425	25596
SAL_CA5557AA	78686	SRR1960082	2014	12		Europe	United Kingdom	Enteritidis	Enteritidis	1425	25595
SAL_CA5561AA	79069	SRR1960078	2015	1		Europe	United Kingdom	Enteritidis	Enteritidis	1425	25592
SAL_CA5566AA	71409	SRR1960073	2012	11	6	Europe	United Kingdom	Enteritidis	Enteritidis	1425	25587
SAL_CA5568AA	79087	SRR1960070	2015	1		Europe	United Kingdom	Enteritidis	Enteritidis	1425	25586
SAL_CA5573AA	78956	SRR1960057	2015	1		Europe	United Kingdom	Enteritidis	Enteritidis	1425	25582
SAL_CA5580AA	71408	SRR1960050	2012	11		Europe	United Kingdom	Enteritidis	Enteritidis	1425	25577
SAL_CA5581AA	79022	SRR1960049	2015	1		Europe	United Kingdom	Enteritidis	Enteritidis	1425	25576
SAL_CA5596AA	73623	SRR1960034	2014	12		Europe	United Kingdom	Enteritidis	Enteritidis	1425	25341
SAL_CA5598AA	69743	SRR1960032	2014	11	13	Europe	United Kingdom	Enteritidis	Enteritidis	3888	25562
SAL_CA5599AA	78699	SRR1960031	2014	12		Europe	United Kingdom	Enteritidis	Enteritidis	1425	25561
SAL_CA5606AA	68893	SRR1959724	2014	10		Europe	United Kingdom	Enteritidis	Enteritidis	1425	25555
SAL_CA5611AA	74301	SRR1959719	2014	12		Europe	United Kingdom	Enteritidis	Enteritidis	1425	25550
SAL_CA5615AA	71533	SRR1959512	2014	11		Europe	United Kingdom	Enteritidis	Enteritidis	1425	25546
SAL_CA5617AA	80352	SRR1959510	2015	1		Europe	United Kingdom	Enteritidis	Enteritidis	1425	25544
SAL_CA5624AA	75746	SRR1959503	2014	12		Europe	United Kingdom	Enteritidis	Enteritidis	1425	25537
SAL_CA5627AA	68898	SRR1959498	2014	10		Europe	United Kingdom	Enteritidis	Enteritidis	1425	25534
SAL_CA5636AA	80347	SRR1959487	2015	1		Europe	United Kingdom	Enteritidis	Enteritidis	1425	25525
SAL_CA5638AA	78717	SRR1959485	2015	1		Europe	United Kingdom	Enteritidis	Enteritidis	28416	25524
SAL_CA5639AA	73581	SRR1959484	2014	9		Europe	United Kingdom	Enteritidis	Enteritidis	3888	25523
SAL_CA5643AA	69742	SRR1959479	2014	11		Europe	United Kingdom	Enteritidis	Enteritidis	1425	25519
SAL_CA5654AA	68894	SRR1959468	2014	10	23	Europe	United Kingdom	Enteritidis	Enteritidis	1425	25510
SAL_CA5661AA	79086	SRR1959461	2015	1		Europe	United Kingdom	Enteritidis	Enteritidis	1425	7210
SAL_CA5663AA	69189	SRR1959459	2014	11		Europe	United Kingdom	Enteritidis	Enteritidis	1425	24707
SAL_CA5664AA	80340	SRR1959458	2015	1		Europe	United Kingdom	Enteritidis	Enteritidis	1425	25505
SAL_CA5665AA	69745	SRR1959457	2014	11		Europe	United Kingdom	Enteritidis	Enteritidis	1425	25504
SAL_CA5667AA	69769	SRR1959455	2014	11		Europe	United Kingdom	Enteritidis	Enteritidis	1425	25502

SAL_CA5678AA	71396	SRR1959443	2012	11	6	Europe	United Kingdom	Enteritidis	Enteritidis	1425	25491
SAL_CA5685AA	68875	SRR1959435	2014	10		Europe	United Kingdom	Enteritidis	Enteritidis	3888	25485
SAL_CA5686AA	71570	SRR1959434	2014	12		Europe	United Kingdom	Enteritidis	Enteritidis	1425	25484
SAL_CA5689AA	69139	SRR1959431	2014	11		Europe	United Kingdom	Enteritidis	Enteritidis	1425	25482
SAL_CA5692AA	71392	SRR1959428	2012	11		Europe	United Kingdom	Enteritidis	Enteritidis	1425	25479
SAL_CA5699AA	69744	SRR1959421	2014	11		Europe	United Kingdom	Enteritidis	Enteritidis	1425	25474
SAL_CA5705AA	80343	SRR1959415	2015	1		Europe	United Kingdom	Enteritidis	Enteritidis	1425	25468
SAL_CA5716AA	78703	SRR1959404	2014	12		Europe	United Kingdom	Enteritidis	Enteritidis	1425	25457
SAL_CA5721AA	68896	SRR1959399	2014	10		Europe	United Kingdom	Enteritidis	Enteritidis	1425	25454
SAL_CA5722AA	69192	SRR1959398	2014	11		Europe	United Kingdom	Enteritidis	Enteritidis	1425	25453
SAL_CA5724AA	71397	SRR1959396	2012	11		Europe	United Kingdom	Enteritidis	Enteritidis	1425	25451
SAL_CA5730AA	81176	SRR1959390	2015	1		Europe	United Kingdom	Enteritidis	Enteritidis	1425	25446
SAL_CA5731AA	80346	SRR1959389	2015	1		Europe	United Kingdom	Enteritidis	Enteritidis	1425	25445
SAL_CA5734AA	73611	SRR1959386	2014	12	9	Europe	United Kingdom	Enteritidis	Enteritidis	1425	25442
SAL_CA5742AA	66462	SRR1959366	2014	10		Europe	United Kingdom	Enteritidis	Enteritidis	1425	25437
SAL_CA5747AA	69747	SRR1959361	2014	11		Europe	United Kingdom	Enteritidis	Enteritidis	1425	25432
SAL_CA5751AA	73528	SRR1959321	2014	9		Europe	United Kingdom	Enteritidis	Enteritidis	3888	25428
SAL_CA5752AA	73573	SRR1959320	2014	9		Europe	United Kingdom	Enteritidis	Enteritidis	3963	21198
SAL_CA5757AA	74326	SRR1959315	2014	12		Europe	United Kingdom	Enteritidis	Enteritidis	1425	25424
SAL_CA5760AA	80361	SRR1959312	2015	1		Europe	United Kingdom	Enteritidis	Enteritidis	1425	7117
SAL_CA5774AA	79083	SRR1959298	2015	1		Europe	United Kingdom	Enteritidis	Enteritidis	1425	25411
SAL_CA5778AA	78742	SRR1959294	2014	12		Europe	United Kingdom	Enteritidis	Enteritidis	1425	25407
SAL_CA5782AA	69740	SRR1959290	2014	11		Europe	United Kingdom	Enteritidis	Enteritidis	1425	24936
SAL_CA5783AA	66881	SRR1959289	2014	10		Europe	United Kingdom	Enteritidis	Enteritidis	1425	24563
SAL_CA5784AA	73559	SRR1959288	2014	9		Europe	United Kingdom	Enteritidis	Enteritidis	1425	25403
SAL_CA5787AA	78727	SRR1959285	2014	12		Europe	United Kingdom	Enteritidis	Enteritidis	1425	25400
SAL_CA5791AA	73641	SRR1959281	2014	12		Europe	United Kingdom	Enteritidis	Enteritidis	1425	25396
SAL_CA5793AA	80349	SRR1959279	2015	1		Europe	United Kingdom	Enteritidis	Enteritidis	3888	25394
SAL_CA5797AA	71394	SRR1959275	2012	11		Europe	United Kingdom	Enteritidis	Enteritidis	1425	25391
SAL_CA5799AA	75668	SRR1959273	2014	12		Europe	United Kingdom	Enteritidis	Enteritidis	1425	25389
SAL_CA5800AA	73640	SRR1959272	2014	12		Europe	United Kingdom	Enteritidis	Enteritidis	1425	25388
SAL_CA5802AA	78660	SRR1959270	2014	12		Europe	United Kingdom	Enteritidis	Enteritidis	3888	25386
SAL_CA5803AA	73536	SRR1959269	2014	9		Europe	United Kingdom	Enteritidis	Enteritidis	1425	25385
SAL_CA5814AA	71400	SRR1959258	2012	11		Europe	United Kingdom	Enteritidis	Enteritidis	1425	25375
SAL_CA5818AA	68874	SRR1959254	2014	10		Europe	United Kingdom	Enteritidis	Enteritidis	1425	25372
SAL_CA5829AA	73582	SRR1959243	2014	9		Europe	United Kingdom	Enteritidis	Enteritidis	1425	24690
SAL_CA5843AA	66492	SRR1959229	2014	10		Europe	United Kingdom	Enteritidis	Enteritidis	1425	25348
SAL_CA5845AA	70667	SRR1959227	2014	11		Europe	United Kingdom	Enteritidis	Enteritidis	1425	25341
SAL_CA5846AA	70657	SRR1959226	2014	11	27	Europe	United Kingdom	Enteritidis	Enteritidis	1425	25347
SAL_CA5850AA	80307	SRR1959222	2015	1	13	Europe	United Kingdom	Enteritidis	Enteritidis	1425	25345
SAL_CA5854AA	71574	SRR1959217	2014	12		Europe	United Kingdom	Enteritidis	Enteritidis	1425	25341
SAL_CA5860AA	IEH-NGS-SAL-02398	SRR1958895	2014	4		North America	United States	Enteritidis (Predicted)	Enteritidis	1425	25336
SAL_CA5886AA	46117	SRR1958691	2014	8		Europe	United Kingdom	Enteritidis	Enteritidis	1425	25316
SAL_CA5888AA	50153	SRR1958689	2014	9		Europe	United Kingdom	Enteritidis	Enteritidis	1425	25062
SAL_CA5893AA	49752	SRR1958684	2014	9		Europe	United Kingdom	Enteritidis	Enteritidis	1425	25311
SAL_CA5898AA	66838	SRR1958679	2014	9		Europe	United Kingdom	Enteritidis	Enteritidis	28005	25306
SAL_CA5899AA	49748	SRR1958678	2014	9		Europe	United Kingdom	Enteritidis	Enteritidis	1425	25305
SAL_CA5901AA	60074	SRR1958676	2014	10	9	Europe	United Kingdom	Enteritidis	Enteritidis	3888	7317
SAL_CA5906AA	54048	SRR1958671	2014	9		Europe	United Kingdom	Enteritidis	Enteritidis	1425	25299
SAL_CA5909AA	62599	SRR1958668	2014	10		Europe	United Kingdom	Enteritidis	Enteritidis	3888	25296
SAL_CA5913AA	46122	SRR1958664	2014	8	29	Europe	United Kingdom	Enteritidis	Enteritidis	3961	25292
SAL_CA5926AA	48247	SRR1958651	2014	9		Europe	United Kingdom	Enteritidis	Enteritidis	1425	25282
SAL_CA5931AA	62549	SRR1958646	2014	9		Europe	United Kingdom	Enteritidis	Enteritidis	1425	25277
SAL_CA5944AA	66842	SRR1958633	2014	9		Europe	United Kingdom	Enteritidis	Enteritidis	1425	25267
SAL_CA5949AA	63547	SRR1958628	2014	10		Europe	United Kingdom	Enteritidis	Enteritidis	1425	25196
SAL_CA5960AA	62629	SRR1958617	2014	11		Europe	United Kingdom	Enteritidis	Enteritidis	3888	25253
SAL_CA5963AA	62556	SRR1958614	2014	8	29	Europe	United Kingdom	Enteritidis	Enteritidis	3888	25250
SAL_CA5971AA	50113	SRR1958606	2014	9		Europe	United Kingdom	Enteritidis	Enteritidis	1425	25242
SAL_CA5977AA	48246	SRR1958600	2014	9		Europe	United Kingdom	Enteritidis	Enteritidis	1425	25236

SAL_CA5980AA	50112	SRR1958597	2014	9		Europe	United Kingdom	Enteritidis	Enteritidis	1425	25233
SAL_CA5982AA	62600	SRR1958595	2014	10		Europe	United Kingdom	Enteritidis	Enteritidis	-9660	25231
SAL_CA5983AA	57643	SRR1958594	2014	10		Europe	United Kingdom	Enteritidis	Enteritidis	1425	25230
SAL_CA5992AA	60374	SRR1958585	2014	10		Europe	United Kingdom	Enteritidis	Enteritidis	1425	25223
SAL_CA5993AA	66480	SRR1958584	2014	10		Europe	United Kingdom	Enteritidis (Predicted)	Enteritidis	1425	25222
SAL_CA5997AA	48265	SRR1958580	2014	9		Europe	United Kingdom	Enteritidis	Enteritidis	3888	25220
SAL_CA5999AA	50150	SRR1958578	2014	9	24	Europe	United Kingdom	Enteritidis	Enteritidis	1425	25218
SAL_CA6003AA	62609	SRR1958574	2014	10		Europe	United Kingdom	Enteritidis	Enteritidis	1425	25215
SAL_CA6006AA	62616	SRR1958571	2014	10		Europe	United Kingdom	Enteritidis	Enteritidis	1425	25212
SAL_CA6012AA	63501	SRR1958565	2014	9		Europe	United Kingdom	Enteritidis	Enteritidis	3888	25206
SAL_CA6017AA	60355	SRR1958560	2014	9		Europe	United Kingdom	Enteritidis	Enteritidis	26493	25201
SAL_CA6018AA	54061	SRR1958559	2014	9		Europe	United Kingdom	Enteritidis	Enteritidis	1425	25200
SAL_CA6019AA	48226	SRR1958558	2014	9		Europe	United Kingdom	Enteritidis	Enteritidis	1425	25199
SAL_CA6022AA	62561	SRR1958555	2014	9		Europe	United Kingdom	Enteritidis	Enteritidis	1425	25196
SAL_CA6027AA	63524	SRR1958550	2014	10		Europe	United Kingdom	Enteritidis	Enteritidis	1425	25191
SAL_CA6030AA	60131	SRR1958547	2014	10		Europe	United Kingdom	Enteritidis	Enteritidis	1425	25188
SAL_CA6031AA	46156	SRR1958546	2014	8	19	Europe	United Kingdom	Enteritidis	Enteritidis	3888	25187
SAL_CA6038AA	54045	SRR1958539	2014	9		Europe	United Kingdom	Enteritidis	Enteritidis	1425	25180
SAL_CA6040AA	49740	SRR1958537	2014	9		Europe	United Kingdom	Enteritidis	Enteritidis	1425	25178
SAL_CA6041AA	57661	SRR1958536	2014	10		Europe	United Kingdom	Enteritidis	Enteritidis	1425	25177
SAL_CA6042AA	48258	SRR1958535	2014	9		Europe	United Kingdom	Enteritidis	Enteritidis	1425	25176
SAL_CA6049AA	49700	SRR1958528	2014	9		Europe	United Kingdom	Enteritidis	Enteritidis	1425	25171
SAL_CA6051AA	62513	SRR1958526	2014	10		Europe	United Kingdom	Enteritidis	Enteritidis	1425	25169
SAL_CA6060AA	49742	SRR1958517	2014	9		Europe	United Kingdom	Enteritidis	Enteritidis	3963	25161
SAL_CA6061AA	64967	SRR1958516	2014	11		Europe	United Kingdom	Enteritidis (Predicted)	Enteritidis	1425	24834
SAL_CA6066AA	54021	SRR1958511	2014	9		Europe	United Kingdom	Enteritidis	Enteritidis	1425	25156
SAL_CA6067AA	60068	SRR1958510	2014	10		Europe	United Kingdom	Enteritidis	Enteritidis	1425	24842
SAL_CA6073AA	60382	SRR1958504	2014	10	7	Europe	United Kingdom	Enteritidis	Enteritidis	3888	7317
SAL_CA6083AA	50152	SRR1958494	2014	9		Europe	United Kingdom	Enteritidis	Enteritidis	1425	25143
SAL_CA6084AA	62550	SRR1958493	2014	9		Europe	United Kingdom	Enteritidis	Enteritidis	1425	25142
SAL_CA6087AA	49745	SRR1958490	2014	9		Europe	United Kingdom	Enteritidis	Enteritidis	1425	25139
SAL_CA6088AA	54052	SRR1958489	2014	9		Europe	United Kingdom	Enteritidis	Enteritidis	1425	25138
SAL_CA6091AA	46148	SRR1958486	2014	9		Europe	United Kingdom	Enteritidis	Enteritidis	1425	25135
SAL_CA6098AA	63557	SRR1958479	2014	10		Europe	United Kingdom	Enteritidis	Enteritidis	3888	25129
SAL_CA6117AA	62554	SRR1958460	2014	8	24	Europe	United Kingdom	Enteritidis	Enteritidis	3888	25112
SAL_CA6123AA	63516	SRR1958454	2014	10		Europe	United Kingdom	Enteritidis	Enteritidis	1425	25107
SAL_CA6129AA	60364	SRR1958448	2014	10		Europe	United Kingdom	Enteritidis	Enteritidis	1425	24842
SAL_CA6142AA	50149	SRR1958435	2014	9		Europe	United Kingdom	Enteritidis	Enteritidis	1425	25091
SAL_CA6146AA	63510	SRR1958431	2014	10		Europe	United Kingdom	Enteritidis	Enteritidis	1425	25087
SAL_CA6147AA	60375	SRR1958430	2014	10		Europe	United Kingdom	Enteritidis	Enteritidis	3888	3621
SAL_CA6148AA	50111	SRR1958429	2014	9		Europe	United Kingdom	Enteritidis	Enteritidis	1425	25086
SAL_CA6149AA	62558	SRR1958428	2014	9		Europe	United Kingdom	Enteritidis	Enteritidis	3906	25085
SAL_CA6150AA	46123	SRR1958427	2014	8		Europe	United Kingdom	Enteritidis	Enteritidis	1425	25084
SAL_CA6151AA	50147	SRR1958426	2014	9		Europe	United Kingdom	Enteritidis	Enteritidis	1425	25083
SAL_CA6153AA	63542	SRR1958424	2014	10		Europe	United Kingdom	Enteritidis	Enteritidis	3888	25081
SAL_CA6155AA	54039	SRR1958422	2014	9		Europe	United Kingdom	Enteritidis	Enteritidis	1425	25079
SAL_CA6156AA	50161	SRR1958421	2014	9		Europe	United Kingdom	Enteritidis	Enteritidis	3888	25078
SAL_CA6158AA	60127	SRR1958419	2014	10		Europe	United Kingdom	Enteritidis	Enteritidis	1425	25076
SAL_CA6163AA	53985	SRR1958414	2014	9		Europe	United Kingdom	Enteritidis	Enteritidis	1425	25072
SAL_CA6164AA	62553	SRR1958413	2014	9		Europe	United Kingdom	Enteritidis	Enteritidis	1425	25071
SAL_CA6168AA	46150	SRR1958409	2014	9		Europe	United Kingdom	Enteritidis	Enteritidis	1425	25067
SAL_CA6174AA	63536	SRR1958403	2014	10		Europe	United Kingdom	Enteritidis	Enteritidis	1425	25062
SAL_CA6179AA	60065	SRR1958398	2014	10		Europe	United Kingdom	Enteritidis	Enteritidis	1425	25057
SAL_CA6180AA	50160	SRR1958397	2014	9		Europe	United Kingdom	Enteritidis	Enteritidis	1425	25056
SAL_CA6187AA	59992	SRR1958390	2014	9		Europe	United Kingdom	Enteritidis	Enteritidis	1425	25049
SAL_CA6192AA	54030	SRR1958385	2014	9		Europe	United Kingdom	Enteritidis	Enteritidis	1425	25044
SAL_CA6195AA	49703	SRR1958382	2014	9		Europe	United Kingdom	Enteritidis	Enteritidis	1425	25041
SAL_CA6196AA	63513	SRR1958381	2014	9		Europe	United Kingdom	Enteritidis	Enteritidis	1425	25004
SAL_CA6198AA	46617	SRR1958379	2014	7		Europe	United Kingdom	Enteritidis	Enteritidis	1425	25039

SAL_CA6204AA	60086	SRR1958373	2014	10		Europe	United Kingdom	Enteritidis	Enteritidis	1425	25034
SAL_CA6208AA	60004	SRR1958369	2014	10		Europe	United Kingdom	Enteritidis	Enteritidis	3906	25030
SAL_CA6209AA	62539	SRR1958368	2014	10		Europe	United Kingdom	Enteritidis	Enteritidis	1425	25029
SAL_CA6210AA	49696	SRR1958367	2014	9		Europe	United Kingdom	Enteritidis	Enteritidis	1425	25028
SAL_CA6213AA	46151	SRR1958364	2014	9		Europe	United Kingdom	Enteritidis	Enteritidis	1425	25025
SAL_CA6216AA	60071	SRR1958361	2014	10		Europe	United Kingdom	Enteritidis	Enteritidis	1425	25023
SAL_CA6230AA	58531	SRR1958347	2014	10		Europe	United Kingdom	Enteritidis	Enteritidis	1425	25011
SAL_CA6239AA	63538	SRR1958338	2014	10		Europe	United Kingdom	Enteritidis	Enteritidis	1425	25004
SAL_CA6245AA	46146	SRR1958332	2014	9		Europe	United Kingdom	Enteritidis	Enteritidis	1425	24999
SAL_CA6248AA	46614	SRR1958329	2014	7		Europe	United Kingdom	Enteritidis	Enteritidis	3888	24996
SAL_CA6252AA	54060	SRR1958325	2014	9	25	Europe	United Kingdom	Enteritidis	Enteritidis	28412	24992
SAL_CA6253AA	49741	SRR1958324	2014	9		Europe	United Kingdom	Enteritidis	Enteritidis	3888	24991
SAL_CA6256AA	57626	SRR1958321	2014	8		Europe	United Kingdom	Enteritidis	Enteritidis	1425	24988
SAL_CA6263AA	60363	SRR1958314	2014	10		Europe	United Kingdom	Enteritidis	Enteritidis	1425	24982
SAL_CA6267AA	57627	SRR1958310	2014	10		Europe	United Kingdom	Enteritidis	Enteritidis	1425	24978
SAL_CA6268AA	60381	SRR1958309	2014	10		Europe	United Kingdom	Enteritidis	Enteritidis	1425	24977
SAL_CA6270AA	48282	SRR1958307	2014	9		Europe	United Kingdom	Enteritidis	Enteritidis	1425	6917
SAL_CA6272AA	49746	SRR1958305	2014	9		Europe	United Kingdom	Enteritidis	Enteritidis	1425	24974
SAL_CA6282AA	53986	SRR1958295	2014	9		Europe	United Kingdom	Enteritidis	Enteritidis	1425	24965
SAL_CA6284AA	48240	SRR1958293	2014	9		Europe	United Kingdom	Enteritidis	Enteritidis	1425	24963
SAL_CA6287AA	48250	SRR1958290	2014	9		Europe	United Kingdom	Enteritidis	Enteritidis	1425	24690
SAL_CA6293AA	48224	SRR1958284	2014	9		Europe	United Kingdom	Enteritidis	Enteritidis	1425	24956
SAL_CA6295AA	60138	SRR1958282	2014	10		Europe	United Kingdom	Enteritidis	Enteritidis	1425	24954
SAL_CA6297AA	66845	SRR1958280	2014	10	13	Europe	United Kingdom	Enteritidis	Enteritidis	3888	7317
SAL_CA6302AA	62559	SRR1958275	2014	9		Europe	United Kingdom	Enteritidis	Enteritidis	1425	2222
SAL_CA6305AA	62610	SRR1958272	2014	10	30	Europe	United Kingdom	Enteritidis	Enteritidis	3888	24946
SAL_CA6307AA	46613	SRR1958270	2014	7		Europe	United Kingdom	Enteritidis	Enteritidis	3888	24944
SAL_CA6308AA	48235	SRR1958269	2014	9		Europe	United Kingdom	Enteritidis	Enteritidis	1425	24943
SAL_CA6309AA	48248	SRR1958268	2014	9		Europe	United Kingdom	Enteritidis	Enteritidis	3888	24942
SAL_CA6313AA	46119	SRR1958264	2014	8		Europe	United Kingdom	Enteritidis	Enteritidis	3888	24938
SAL_CA6315AA	60378	SRR1958262	2014	10		Europe	United Kingdom	Enteritidis	Enteritidis	1425	24936
SAL_CA6323AA	50107	SRR1958254	2014	9		Europe	United Kingdom	Enteritidis	Enteritidis	1425	24928
SAL_CA6324AA	66469	SRR1958253	2014	9		Europe	United Kingdom	Enteritidis	Enteritidis	3888	24927
SAL_CA6325AA	63509	SRR1958252	2014	10		Europe	United Kingdom	Enteritidis	Enteritidis	1425	24766
SAL_CA6326AA	66477	SRR1958251	2014	10		Europe	United Kingdom	Enteritidis	Enteritidis	1425	24926
SAL_CA6335AA	48254	SRR1958242	2014	9		Europe	United Kingdom	Enteritidis	Enteritidis	1425	24917
SAL_CA6341AA	62580	SRR1958236	2014	9		Europe	United Kingdom	Enteritidis	Enteritidis	1425	24913
SAL_CA6342AA	46149	SRR1958235	2014	9		Europe	United Kingdom	Enteritidis	Enteritidis	1425	24912
SAL_CA6347AA	53991	SRR1958230	2014	9		Europe	United Kingdom	Enteritidis	Enteritidis	1425	24907
SAL_CA6351AA	66862	SRR1958226	2014	10		Europe	United Kingdom	Enteritidis	Enteritidis	26483	24903
SAL_CA6358AA	57641	SRR1958219	2014	9	26	Europe	United Kingdom	Enteritidis	Enteritidis	3888	7317
SAL_CA6369AA	50158	SRR1958208	2014	9	20	Europe	United Kingdom	Enteritidis	Enteritidis	3888	2066
SAL_CA6373AA	62583	SRR1958204	2014	9		Europe	United Kingdom	Enteritidis	Enteritidis	1425	24884
SAL_CA6379AA	62625	SRR1958198	2014	11		Europe	United Kingdom	Enteritidis	Enteritidis	1425	24879
SAL_CA6381AA	60379	SRR1958196	2014	10		Europe	United Kingdom	Enteritidis	Enteritidis	3906	24877
SAL_CA6387AA	57650	SRR1958190	2014	10		Europe	United Kingdom	Enteritidis	Enteritidis	1425	24871
SAL_CA6390AA	63532	SRR1958187	2014	10		Europe	United Kingdom	Enteritidis	Enteritidis	1425	24868
SAL_CA6396AA	63568	SRR1958180	2014	9		Europe	United Kingdom	Enteritidis	Enteritidis	3888	24864
SAL_CA6406AA	46128	SRR1958164	2014	8	29	Europe	United Kingdom	Enteritidis	Enteritidis	1425	24854
SAL_CA6408AA	63570	SRR1958162	2014	9		Europe	United Kingdom	Enteritidis	Enteritidis	1425	24852
SAL_CA6414AA	46627	SRR1958156	2014	7		Europe	United Kingdom	Enteritidis	Enteritidis	1425	24848
SAL_CA6418AA	48243	SRR1958152	2014	9	15	Europe	United Kingdom	Enteritidis	Enteritidis	26493	24844
SAL_CA6420AA	63528	SRR1958150	2014	10		Europe	United Kingdom	Enteritidis	Enteritidis	1425	24842
SAL_CA6426AA	46630	SRR1958144	2014	7		Europe	United Kingdom	Enteritidis	Enteritidis	1425	24836
SAL_CA6428AA	64957	SRR1958142	2014	11		Europe	United Kingdom	Enteritidis	Enteritidis	1425	24834
SAL_CA6433AA	63523	SRR1958137	2014	10		Europe	United Kingdom	Enteritidis	Enteritidis	1425	24829
SAL_CA6434AA	63518	SRR1958136	2014	10		Europe	United Kingdom	Enteritidis	Enteritidis	1425	24766
SAL_CA6435AA	49738	SRR1958135	2014	9		Europe	United Kingdom	Enteritidis	Enteritidis	1425	24828
SAL_CA6436AA	62511	SRR1958134	2014	10		Europe	United Kingdom	Enteritidis	Enteritidis	1425	24827

SAL_CA6439AA	48206	SRR1958131	2014	9	4	Europe	United Kingdom	Enteritidis	Enteritidis	3888	24824
SAL_CA6440AA	50155	SRR1958130	2014	9		Europe	United Kingdom	Enteritidis	Enteritidis	1425	24823
SAL_CA6441AA	50157	SRR1958129	2014	9		Europe	United Kingdom	Enteritidis	Enteritidis	3888	24822
SAL_CA6449AA	66859	SRR1958121	2014	10	24	Europe	United Kingdom	Enteritidis	Enteritidis	1425	24816
SAL_CA6451AA	50154	SRR1958119	2014	9		Europe	United Kingdom	Enteritidis	Enteritidis	1425	24814
SAL_CA6452AA	58514	SRR1958118	2014	10		Europe	United Kingdom	Enteritidis	Enteritidis	1425	24813
SAL_CA6453AA	46612	SRR1958117	2014	7		Europe	United Kingdom	Enteritidis	Enteritidis	1425	24812
SAL_CA6455AA	49750	SRR1958115	2014	9		Europe	United Kingdom	Enteritidis	Enteritidis	1425	24810
SAL_CA6457AA	50110	SRR1958113	2014	9		Europe	United Kingdom	Enteritidis	Enteritidis	1425	24808
SAL_CA6462AA	62548	SRR1958108	2014	9		Europe	United Kingdom	Enteritidis	Enteritidis	3888	24804
SAL_CA6464AA	50128	SRR1958106	2014	9		Europe	United Kingdom	Enteritidis	Enteritidis	1425	24802
SAL_CA6467AA	63519	SRR1958103	2014	10		Europe	United Kingdom	Enteritidis	Enteritidis	1425	18562
SAL_CA6468AA	64951	SRR1958102	2014	11		Europe	United Kingdom	Enteritidis	Enteritidis	1425	24799
SAL_CA6473AA	63541	SRR1958097	2014	10		Europe	United Kingdom	Enteritidis	Enteritidis	1425	24794
SAL_CA6475AA	60133	SRR1958095	2014	10		Europe	United Kingdom	Enteritidis	Enteritidis	1425	24792
SAL_CA6478AA	48214	SRR1958092	2014	9		Europe	United Kingdom	Enteritidis	Enteritidis	1425	24789
SAL_CA6481AA	49743	SRR1958089	2014	9		Europe	United Kingdom	Enteritidis	Enteritidis	3963	24786
SAL_CA6482AA	58464	SRR1958088	2014	9		Europe	United Kingdom	Enteritidis	Enteritidis	1425	24785
SAL_CA6493AA	63530	SRR1958077	2014	9		Europe	United Kingdom	Enteritidis	Enteritidis	1425	24775
SAL_CA6494AA	62495	SRR1958076	2014	9		Europe	United Kingdom	Enteritidis	Enteritidis	1425	24774
SAL_CA6496AA	46124	SRR1958074	2014	8		Europe	United Kingdom	Enteritidis	Enteritidis	1425	24772
SAL_CA6502AA	66488	SRR1958068	2014	10		Europe	United Kingdom	Enteritidis	Enteritidis	1425	24766
SAL_CA6503AA	62552	SRR1958067	2014	9		Europe	United Kingdom	Enteritidis	Enteritidis	3888	24765
SAL_CA6505AA	57648	SRR1958065	2014	10		Europe	United Kingdom	Enteritidis	Enteritidis	3888	404
SAL_CA6506AA	60134	SRR1958064	2014	10		Europe	United Kingdom	Enteritidis	Enteritidis	1425	24707
SAL_CA6510AA	60129	SRR1958060	2014	10		Europe	United Kingdom	Enteritidis	Enteritidis	1425	16120
SAL_CA6517AA	57623	SRR1958052	2014	8	8	Europe	United Kingdom	Enteritidis	Enteritidis	3888	2066
SAL_CA6518AA	48208	SRR1958051	2014	9		Europe	United Kingdom	Enteritidis	Enteritidis	1425	24755
SAL_CA6523AA	63533	SRR1958046	2014	9		Europe	United Kingdom	Enteritidis	Enteritidis	1425	24750
SAL_CA6526AA	66839	SRR1958043	2014	9		Europe	United Kingdom	Enteritidis	Enteritidis	3888	24747
SAL_CA6529AA	48229	SRR1958040	2014	9		Europe	United Kingdom	Enteritidis	Enteritidis	1425	24744
SAL_CA6534AA	66473	SRR1958035	2014	10		Europe	United Kingdom	Enteritidis	Enteritidis	7811	24739
SAL_CA6536AA	50109	SRR1958033	2014	9	16	Europe	United Kingdom	Enteritidis	Enteritidis	3888	24738
SAL_CA6541AA	46085	SRR1958028	2014	8	19	Europe	United Kingdom	Enteritidis	Enteritidis	3888	24732
SAL_CA6542AA	49739	SRR1958027	2014	9		Europe	United Kingdom	Enteritidis	Enteritidis	1425	24731
SAL_CA6546AA	62591	SRR1958023	2014	10		Europe	United Kingdom	Enteritidis	Enteritidis	1425	24727
SAL_CA6547AA	60377	SRR1958022	2014	10		Europe	United Kingdom	Enteritidis	Enteritidis	1425	24726
SAL_CA6550AA	50159	SRR1958018	2014	9		Europe	United Kingdom	Enteritidis	Enteritidis	1425	24723
SAL_CA6551AA	54056	SRR1958017	2014	9		Europe	United Kingdom	Enteritidis	Enteritidis	1425	24722
SAL_CA6557AA	58510	SRR1958011	2014	10	2	Europe	United Kingdom	Enteritidis	Enteritidis	3888	7317
SAL_CA6568AA	63527	SRR1958000	2014	10		Europe	United Kingdom	Enteritidis	Enteritidis	1425	24707
SAL_CA6575AA	63521	SRR1957993	2014	9		Europe	United Kingdom	Enteritidis	Enteritidis	1425	24700
SAL_CA6580AA	46152	SRR1957988	2014	9		Europe	United Kingdom	Enteritidis	Enteritidis	1425	24695
SAL_CA6582AA	46118	SRR1957986	2014	8	20	Europe	United Kingdom	Enteritidis	Enteritidis	3888	24693
SAL_CA6585AA	58530	SRR1957983	2014	10		Europe	United Kingdom	Enteritidis	Enteritidis	1425	24690
SAL_CA6587AA	48253	SRR1957981	2014	9		Europe	United Kingdom	Enteritidis	Enteritidis	1425	24688
SAL_CA6589AA	60062	SRR1957979	2014	10		Europe	United Kingdom	Enteritidis	Enteritidis	3888	24686
SAL_CA6590AA	54027	SRR1957978	2014	9		Europe	United Kingdom	Enteritidis	Enteritidis	1425	24685
SAL_CA6592AA	54043	SRR1957976	2014	9		Europe	United Kingdom	Enteritidis	Enteritidis	1425	24683
SAL_CA6593AA	49704	SRR1957975	2014	9		Europe	United Kingdom	Enteritidis	Enteritidis	1425	24682
SAL_CA6594AA	62585	SRR1957974	2014	10		Europe	United Kingdom	Enteritidis	Enteritidis	3906	24681
SAL_CA6599AA	49697	SRR1957969	2014	9		Europe	United Kingdom	Enteritidis	Enteritidis	1425	24676
SAL_CA6601AA	60136	SRR1957967	2014	10		Europe	United Kingdom	Enteritidis	Enteritidis	1425	24674
SAL_CA6610AA	59995	SRR1957958	2014	10		Europe	United Kingdom	Enteritidis	Enteritidis	1425	24666
SAL_CA6617AA	60140	SRR1957951	2014	10		Europe	United Kingdom	Enteritidis	Enteritidis	1425	24660
SAL_CA6618AA	58477	SRR1957950	2014	9		Europe	United Kingdom	Enteritidis	Enteritidis	1425	24659
SAL_CA6619AA	60376	SRR1957949	2014	10		Europe	United Kingdom	Enteritidis	Enteritidis	1425	24563
SAL_CA6626AA	63549	SRR1957942	2014	10		Europe	United Kingdom	Enteritidis	Enteritidis	1425	24652
SAL_CA6629AA	57672	SRR1957939	2014	8		Europe	United Kingdom	Enteritidis	Enteritidis	1425	24649

SAL_CA6635AA	62551	SRR1957933	2014	9	3	Europe	United Kingdom	Enteritidis	Enteritidis	1425	24643
SAL_CA6647AA	48255	SRR1957921	2014	9		Europe	United Kingdom	Enteritidis	Enteritidis	3888	24631
SAL_CA6650AA	49749	SRR1957918	2014	9		Europe	United Kingdom	Enteritidis	Enteritidis	1425	24629
SAL_CA6654AA	54020	SRR1957914	2014	9		Europe	United Kingdom	Enteritidis	Enteritidis	1425	24625
SAL_CA6657AA	60125	SRR1957911	2014	10		Europe	United Kingdom	Enteritidis	Enteritidis	1425	24622
SAL_CA6661AA	46631	SRR1957907	2014	7		Europe	United Kingdom	Enteritidis	Enteritidis	1425	24618
SAL_CA6664AA	62617	SRR1957904	2014	10		Europe	United Kingdom	Enteritidis	Enteritidis	1425	24616
SAL_CA6670AA	54007	SRR1957898	2014	9		Europe	United Kingdom	Enteritidis	Enteritidis	1425	24611
SAL_CA6675AA	50129	SRR1957893	2014	9		Europe	United Kingdom	Enteritidis	Enteritidis	1425	24605
SAL_CA6680AA	48262	SRR1957888	2014	9	5	Europe	United Kingdom	Enteritidis	Enteritidis	3888	24601
SAL_CA6689AA	66471	SRR1957879	2014	10		Europe	United Kingdom	Enteritidis	Enteritidis	1425	6917
SAL_CA6691AA	62581	SRR1957877	2014	8	27	Europe	United Kingdom	Enteritidis	Enteritidis	3888	24591
SAL_CA6693AA	57629	SRR1957875	2014	10		Europe	United Kingdom	Enteritidis	Enteritidis	3888	24589
SAL_CA6695AA	46126	SRR1957873	2014	8		Europe	United Kingdom	Enteritidis	Enteritidis	1425	24587
SAL_CA6696AA	62594	SRR1957872	2014	10		Europe	United Kingdom	Enteritidis	Enteritidis	1425	24586
SAL_CA6703AA	54054	SRR1957865	2014	9		Europe	United Kingdom	Enteritidis	Enteritidis	1425	24580
SAL_CA6704AA	62555	SRR1957864	2014	9		Europe	United Kingdom	Enteritidis	Enteritidis	1425	24579
SAL_CA6705AA	46132	SRR1957863	2014	8	29	Europe	United Kingdom	Enteritidis	Enteritidis	26449	24578
SAL_CA6715AA	49744	SRR1957853	2014	9	23	Europe	United Kingdom	Enteritidis	Enteritidis	1425	24569
SAL_CA6716AA	62574	SRR1957852	2014	9	1	Europe	United Kingdom	Enteritidis	Enteritidis	3888	24568
SAL_CA6721AA	62619	SRR1957847	2014	11		Europe	United Kingdom	Enteritidis	Enteritidis	1425	24563
SAL_CA6726AA	62582	SRR1957842	2014	9	2	Europe	United Kingdom	Enteritidis	Enteritidis	3888	24558
SAL_CA6738AA	66449	SRR1957830	2014	10		Europe	United Kingdom	Enteritidis	Enteritidis	1425	24547
SAL_CA6742AA	60373	SRR1957826	2014	10		Europe	United Kingdom	Enteritidis	Enteritidis	1425	24544
SAL_CA6746AA	62557	SRR1957822	2014	9		Europe	United Kingdom	Enteritidis	Enteritidis	27926	24540
SAL_CA6748AA	49751	SRR1957820	2014	9	15	Europe	United Kingdom	Enteritidis	Enteritidis	3888	24538
SAL_CA6754AA	45262	SRR1957814	2014	9	1	Europe	United Kingdom	Enteritidis	Enteritidis	3888	24533
SAL_CA6756AA	44743	SRR1957812	2014	9		Europe	United Kingdom	Enteritidis	Enteritidis	1425	24531
SAL_CA6757AA	44725	SRR1957811	2014	9		Europe	United Kingdom	Enteritidis	Enteritidis	1425	24530
SAL_CA6759AA	44692	SRR1957809	2014	9	8	Europe	United Kingdom	Enteritidis	Enteritidis	1425	15673
SAL_CA6760AA	44744	SRR1957808	2014	9		Europe	United Kingdom	Enteritidis	Enteritidis	1425	24528
SAL_CA6766AA	44739	SRR1957802	2014	9		Europe	United Kingdom	Enteritidis	Enteritidis	1425	24522
SAL_CA6773AA	44757	SRR1957795	2014	9		Europe	United Kingdom	Enteritidis	Enteritidis	1425	24515
SAL_CA6774AA	44756	SRR1957794	2014	9		Europe	United Kingdom	Enteritidis	Enteritidis	3888	24514
SAL_CA6776AA	44689	SRR1957792	2014	9		Europe	United Kingdom	Enteritidis	Enteritidis	1425	24512
SAL_CA6778AA	44753	SRR1957790	2014	9		Europe	United Kingdom	Enteritidis	Enteritidis	1425	24510
SAL_CA6780AA	44723	SRR1957788	2014	9		Europe	United Kingdom	Enteritidis	Enteritidis	3888	24508
SAL_CA6782AA	44730	SRR1957786	2014	9		Europe	United Kingdom	Enteritidis	Enteritidis	1425	24506
SAL_CA6786AA	44726	SRR1957782	2014	9		Europe	United Kingdom	Enteritidis	Enteritidis	1425	24502
SAL_CA6787AA	44737	SRR1957781	2014	9		Europe	United Kingdom	Enteritidis	Enteritidis	1425	24501
SAL_CA6788AA	44752	SRR1957780	2014	9		Europe	United Kingdom	Enteritidis	Enteritidis	3888	24500
SAL_CA6791AA	44741	SRR1957777	2014	9		Europe	United Kingdom	Enteritidis	Enteritidis	1425	24497
SAL_CA6794AA	44683	SRR1957774	2014	8	29	Europe	United Kingdom	Enteritidis	Enteritidis	3888	24493
SAL_CA6797AA	44750	SRR1957771	2014	9		Europe	United Kingdom	Enteritidis	Enteritidis	1425	24492
SAL_CA6798AA	46604	SRR1957770	2014	6		Europe	United Kingdom	Enteritidis	Enteritidis	1425	24491
SAL_CA6801AA	44751	SRR1957767	2014	9		Europe	United Kingdom	Enteritidis	Enteritidis	1425	24488
SAL_CA6802AA	46147	SRR1957766	2014	9		Europe	United Kingdom	Enteritidis	Enteritidis	1425	24487
SAL_CA6805AA	44738	SRR1957763	2014	9		Europe	United Kingdom	Enteritidis	Enteritidis	3888	24485
SAL_CA6806AA	44746	SRR1957762	2014	9		Europe	United Kingdom	Enteritidis	Enteritidis	3888	24484
SAL_CA6807AA	44734	SRR1957761	2014	9		Europe	United Kingdom	Enteritidis	Enteritidis	1425	24483
SAL_CA6810AA	44754	SRR1957758	2014	9		Europe	United Kingdom	Enteritidis	Enteritidis	3888	24480
SAL_CA6811AA	46607	SRR1957757	2014	6		Europe	United Kingdom	Enteritidis	Enteritidis	3888	24479
SAL_CA6813AA	44736	SRR1957755	2014	9		Europe	United Kingdom	Enteritidis	Enteritidis	1425	24477
SAL_CA6814AA	44709	SRR1957754	2014	9		Europe	United Kingdom	Enteritidis	Enteritidis	3888	7867
SAL_CA6818AA	44682	SRR1957750	2014	9		Europe	United Kingdom	Enteritidis	Enteritidis	1425	24473
SAL_CA6819AA	44740	SRR1957749	2014	9		Europe	United Kingdom	Enteritidis	Enteritidis	1425	24472
SAL_CA6820AA	44758	SRR1957748	2014	9		Europe	United Kingdom	Enteritidis	Enteritidis	1425	24471
SAL_CA6821AA	44745	SRR1957747	2014	9		Europe	United Kingdom	Enteritidis	Enteritidis	1425	24470
SAL_CA6822AA	46606	SRR1957746	2014	6		Europe	United Kingdom	Enteritidis	Enteritidis	1425	24469

SAL_CA6827AA	44717	SRR1957741	2014	9	Europe	United Kingdom	Enteritidis	Enteritidis	1425	24465
SAL_CA6829AA	44684	SRR1957739	2014	9	Europe	United Kingdom	Enteritidis	Enteritidis	1425	24463
SAL_CA6831AA	44735	SRR1957737	2014	9	Europe	United Kingdom	Enteritidis	Enteritidis	1425	24461
SAL_CA6837AA	44691	SRR1957731	2014	9	Europe	United Kingdom	Enteritidis	Enteritidis	1425	24456
SAL_CA6838AA	44685	SRR1957730	2014	9	Europe	United Kingdom	Enteritidis	Enteritidis	1425	24455
SAL_CA6841AA	44748	SRR1957727	2014	9	Europe	United Kingdom	Enteritidis	Enteritidis	1425	24452
SAL_CA6849AA	46605	SRR1957719	2014	6	Europe	United Kingdom	Enteritidis	Enteritidis	1425	24444
SAL_CA6859AA	CFSAN030503	SRR1955481	2012		North America	United States	Enteritidis	Enteritidis	1425	24435
SAL_CA6860AA	CFSAN030497	SRR1955480	2012		North America	United States	Enteritidis	Enteritidis	1425	24434
SAL_CA6864AA	CFSAN030266	SRR1955235	2014	4	North America	United States	Enteritidis (Predicted)	Enteritidis	1425	24430
SAL_CA6879AA	CFSAN030504	SRR1952942	2012		North America	United States	Enteritidis	Enteritidis	26162	24416
SAL_CA6880AA	CFSAN030496	SRR1952941	2012		North America	United States	Enteritidis	Enteritidis	1425	24415
SAL_CA6881AA	WAPHL_SAL-A00893	SRR1952939	2015	1	North America	United States	Enteritidis	Enteritidis	1425	24414
SAL_CA6882AA	85727	SRR1952936	2015	2	Europe	United Kingdom	Enteritidis	Enteritidis	1425	24413
SAL_CA6884AA	78666	SRR1952934	2014	12	Europe	United Kingdom	Enteritidis	Enteritidis	1425	24411
SAL_CA6885AA	95238	SRR1952933	2015	3	Europe	United Kingdom	Enteritidis (Predicted)	Enteritidis	1425	24410
SAL_CA6886AA	86879	SRR1952932	2015	2	Europe	United Kingdom	Enteritidis	Enteritidis	1425	24409
SAL_CA6887AA	69274	SRR1952931	2014	11	Europe	United Kingdom	Enteritidis	Enteritidis	1425	24378
SAL_CA6888AA	83323	SRR1952930	2015	1	Europe	United Kingdom	Enteritidis	Enteritidis	1425	24408
SAL_CA6889AA	83344	SRR1952929	2015	1	Europe	United Kingdom	Enteritidis	Enteritidis	1425	24405
SAL_CA6890AA	96238	SRR1952928	2015	3	Europe	United Kingdom	Enteritidis	Enteritidis	1425	24407
SAL_CA6891AA	96227	SRR1952927	2015	3	Europe	United Kingdom	Enteritidis	Enteritidis	1425	24406
SAL_CA6892AA	82547	SRR1952926	2015	1	Europe	United Kingdom	Enteritidis	Enteritidis	1425	24405
SAL_CA6893AA	90474	SRR1952925	2015	2	Europe	United Kingdom	Enteritidis	Enteritidis	27915	24404
SAL_CA6894AA	62590	SRR1952924	2014	10	Europe	United Kingdom	Enteritidis	Enteritidis	1425	24403
SAL_CA6895AA	90475	SRR1952923	2015	2	Europe	United Kingdom	Enteritidis	Enteritidis	27915	24402
SAL_CA6896AA	87355	SRR1952922	2015	2	Europe	United Kingdom	Enteritidis	Enteritidis	1425	24401
SAL_CA6897AA	94101	SRR1952921	2015	3	Europe	United Kingdom	Enteritidis	Enteritidis	1425	24400
SAL_CA6898AA	67533	SRR1952920	2014	11	Europe	United Kingdom	Enteritidis	Enteritidis	1425	24399
SAL_CA6899AA	91219	SRR1952919	2015	2	Europe	United Kingdom	Enteritidis	Enteritidis	1425	24378
SAL_CA6900AA	63904	SRR1952918	2014	11	Europe	United Kingdom	Enteritidis	Enteritidis	1425	24378
SAL_CA6901AA	98281	SRR1952917	2015	3	Europe	United Kingdom	Enteritidis	Enteritidis	1425	24378
SAL_CA6902AA	62540	SRR1952916	2014	10	Europe	United Kingdom	Enteritidis	Enteritidis	1425	24398
SAL_CA6903AA	60083	SRR1952915	2014	10	Europe	United Kingdom	Enteritidis	Enteritidis	1425	24397
SAL_CA6904AA	63734	SRR1952914	2014	10	Europe	United Kingdom	Enteritidis	Enteritidis	1425	24396
SAL_CA6905AA	52961	SRR1952913	2014	10	Europe	United Kingdom	Enteritidis	Enteritidis	1425	24395
SAL_CA6906AA	60380	SRR1952912	2014	10	Europe	United Kingdom	Enteritidis	Enteritidis	1425	24394
SAL_CA6907AA	69746	SRR1952911	2014	11	Europe	United Kingdom	Enteritidis	Enteritidis	1425	24393
SAL_CA6908AA	94071	SRR1952910	2015	3	Europe	United Kingdom	Enteritidis (Predicted)	Enteritidis	1425	24392
SAL_CA6909AA	67563	SRR1952909	2014	11	Europe	United Kingdom	Enteritidis	Enteritidis	1425	24391
SAL_CA6910AA	83702	SRR1952908	2015	1	Europe	United Kingdom	Enteritidis	Enteritidis	1425	24390
SAL_CA6911AA	50148	SRR1952907	2014	9	Europe	United Kingdom	Enteritidis	Enteritidis	1425	24389
SAL_CA6912AA	70698	SRR1952906	2014	12	Europe	United Kingdom	Enteritidis	Enteritidis	1425	24378
SAL_CA6913AA	49747	SRR1952905	2014	9	Europe	United Kingdom	Enteritidis	Enteritidis	1425	24388
SAL_CA6914AA	78701	SRR1952903	2014	12	Europe	United Kingdom	Enteritidis	Enteritidis	1425	24387
SAL_CA6915AA	68973	SRR1952902	2014	11	Europe	United Kingdom	Enteritidis	Enteritidis	1425	24386
SAL_CA6916AA	73136	SRR1952901	2014	12	Europe	United Kingdom	Enteritidis	Enteritidis	1425	24378
SAL_CA6917AA	63535	SRR1952900	2014	10	Europe	United Kingdom	Enteritidis	Enteritidis	1425	24378
SAL_CA6918AA	78629	SRR1952899	2014	12	Europe	United Kingdom	Enteritidis	Enteritidis	1425	24378
SAL_CA6919AA	75956	SRR1952898	2014	12	Europe	United Kingdom	Enteritidis	Enteritidis	1425	24385
SAL_CA6920AA	75953	SRR1952897	2014	12	Europe	United Kingdom	Enteritidis	Enteritidis	1425	24384
SAL_CA6921AA	85756	SRR1952896	2015	2	Europe	United Kingdom	Enteritidis	Enteritidis	1425	24383
SAL_CA6922AA	69213	SRR1952895	2014	11	Europe	United Kingdom	Enteritidis	Enteritidis	1425	24382
SAL_CA6923AA	98284	SRR1952894	2015	3	Europe	United Kingdom	Enteritidis	Enteritidis	1425	24381
SAL_CA6924AA	69753	SRR1952892	2014	11	Europe	United Kingdom	Enteritidis	Enteritidis	1425	24378
SAL_CA6925AA	96204	SRR1952891	2015	3	Europe	United Kingdom	Enteritidis	Enteritidis	1425	24380
SAL_CA6926AA	55562	SRR1952890	2014	9	Europe	United Kingdom	Enteritidis	Enteritidis	1425	24378
SAL_CA6927AA	69752	SRR1952889	2014	11	Europe	United Kingdom	Enteritidis	Enteritidis	1425	24378
SAL_CA6928AA	89104	SRR1952888	2015	2	Europe	United Kingdom	Enteritidis	Enteritidis	1425	24379

SAL_CA6929AA	63883	SRR1952887	2014	11	Europe	United Kingdom	Enteritidis	Enteritidis	1425	24378
SAL_CA6930AA	65024	SRR1952886	2014	11	Europe	United Kingdom	Enteritidis	Enteritidis	1425	24378
SAL_CA6931AA	69221	SRR1952885	2014	11	Europe	United Kingdom	Enteritidis	Enteritidis	1425	24377
SAL_CA6932AA	69911	SRR1952884	2014	11	Europe	United Kingdom	Enteritidis	Enteritidis	1425	24376
SAL_CA6933AA	46154	SRR1952822	2014	9	Europe	United Kingdom	Enteritidis	Enteritidis	1425	24375
SAL_CA6934AA	95024	SRR1952821	2015	3	Europe	United Kingdom	Enteritidis	Enteritidis	-1662	24374
SAL_CA6935AA	94262	SRR1952820	2015	3	Europe	United Kingdom	Enteritidis	Enteritidis	1425	24373
SAL_CA7008AA	IEH-NGS-SAL-02337	SRR1950907	2014	4	North America	United States	Enteritidis (Predicted)	Enteritidis	27904	24310
SAL_CA7012AA	IEH-NGS-SAL-02346	SRR1950902	2014	4	North America	United States	Enteritidis (Predicted)	Enteritidis	1425	24308
SAL_CA7017AA	CFSAN030208	SRR1950897	2014	4	North America	United States	Enteritidis (Predicted)	Enteritidis	1425	24303
SAL_CA7020AA	CFSAN030238	SRR1950884	2014	4	North America	United States	Enteritidis (Predicted)	Enteritidis	1425	24300
SAL_CA7038AA	FDA00000230	SRR1947031	2002	3	North America	Canada	Enteritidis	Enteritidis	1425	24282
SAL_CA7046AA	CFSAN030165	SRR1947023	2014	4	North America	United States	Enteritidis (Predicted)	Enteritidis	1425	24275
SAL_CA7055AA	NY-N11265	SRR1947012	2006		North America	United States	Enteritidis	Enteritidis	1425	24268
SAL_CA7073AA	CFSAN030189	SRR1946992	2014	4	North America	United States	Enteritidis (Predicted)	Enteritidis	1425	24251
SAL_CA7081AA	CFSAN030194	SRR1946982	2014	4	North America	United States	Enteritidis (Predicted)	Enteritidis	1425	24243
SAL_CA7084AA	CFSAN030188	SRR1946978	2014	4	North America	United States	Enteritidis (Predicted)	Enteritidis	1425	24240
SAL_CA7143AA	CFSAN030176	SRR1946906	2014	4	North America	United States	Enteritidis (Predicted)	Enteritidis	1425	24187
SAL_CA7153AA	IEH-NGS-SAL-02307	SRR1946886	2014	4	North America	United States	Enteritidis (Predicted)	Enteritidis	1425	24176
SAL_CA7156AA	NY-N11268	SRR1946883	2006		North America	United States	Enteritidis	Enteritidis	1425	24174
SAL_CA7162AA	CFSAN030200	SRR1946871	2014	4	North America	United States	Enteritidis (Predicted)	Enteritidis	1425	24168
SAL_CA7177AA	PNUSAS000218	SRR1931737			North America	United States	Enteritidis (Predicted)	Enteritidis	1425	24156
SAL_CA7178AA	PNUSAS000217	SRR1931736			North America	United States	Enteritidis (Predicted)	Enteritidis	1425	651
SAL_CA7180AA	PNUSAS000196	SRR1931734	2015	2	North America	United States	Enteritidis	Enteritidis	1425	24154
SAL_CA7181AA	PNUSAS000195	SRR1931732	2015	2	North America	United States	Enteritidis	Enteritidis	1425	24153
SAL_CA7195AA	WAPHL_SAL-A00717	SRR1923641	2006		North America	United States	Enteritidis	Enteritidis	1425	24140
SAL_CA7206AA	FDA00002688	SRR1920228	2008	2	North America	Mexico	Enteritidis	Enteritidis	1425	24129
SAL_CA7219AA	FDA00002665	SRR1920213	2008	1	North America	Mexico	Enteritidis	Enteritidis	1425	24116
SAL_CA7246AA	IEH-NGS-SAL-02239	SRR1918976	2014	4	North America	United States	Enteritidis (Predicted)	Enteritidis	1425	24090
SAL_CA7248AA	IEH-NGS-SAL-02238	SRR1918974	2014	4	North America	United States	Enteritidis (Predicted)	Enteritidis	1425	24088
SAL_CA7252AA	IEH-NGS-SAL-02251	SRR1918967	2014	4	North America	United States	Enteritidis (Predicted)	Enteritidis	1425	24084
SAL_CA7282AA	FDA00001051	SRR1917494	2006	9	Asia	China	Enteritidis	Enteritidis	1425	24058
SAL_CA7306AA	FDA00001096	SRR1917445	2007	4	North America	United States	Enteritidis	Enteritidis	8384	24036
SAL_CA7326AA	IEH-NGS-SAL-02215	SRR1916385	2014	4	North America	United States	Enteritidis (Predicted)	Enteritidis	1425	24017
SAL_CA7329AA	WAPHL_SAL-A00712	SRR1916371	2006		North America	United States	Enteritidis	Enteritidis	1425	8780
SAL_CA7351AA	IEH-NGS-SAL-02219	SRR1916108	2014	4	North America	United States	Enteritidis (Predicted)	Enteritidis	1425	23995
SAL_CA7374AA	IEH-NGS-SAL-02216	SRR1916085	2014	4	North America	United States	Enteritidis (Predicted)	Enteritidis	1425	23973
SAL_CA7393AA	PNUSAS000172	SRR1915427	2015	1	North America	United States	Enteritidis	Enteritidis	1425	23956
SAL_CA7424AA	FL-NRM299	SRR1910756	2006		North America	United States	Enteritidis	Enteritidis	1425	23931
SAL_CA7425AA	FL-NRM296	SRR1910755	2006		North America	United States	Enteritidis	Enteritidis	1425	23930
SAL_CA7435AA	CFSAN029739	SRR1910309	2014	4	North America	United States	Enteritidis (Predicted)	Enteritidis	1425	23920
SAL_CA7437AA	CFSAN029732	SRR1910307	2014	4	North America	United States	Enteritidis (Predicted)	Enteritidis	1425	23918
SAL_CA7439AA	IEH-NGS-SAL-02189	SRR1910305	2014	4	North America	United States	Enteritidis (Predicted)	Enteritidis	1425	23916
SAL_CA7440AA	IEH-NGS-SAL-02187	SRR1910304	2014	4	North America	United States	Enteritidis (Predicted)	Enteritidis	1425	23915
SAL_CA7444AA	IEH-NGS-SAL-02181	SRR1910300	2014	4	North America	United States	Enteritidis (Predicted)	Enteritidis	1425	23911
SAL_CA7450AA	CFSAN029730	SRR1910291	2014	4	North America	United States	Enteritidis (Predicted)	Enteritidis	1425	23905
SAL_CA7458AA	CFSAN029708	SRR1909002	2014	4	North America	United States	Enteritidis (Predicted)	Enteritidis	1425	23898
SAL_CA7465AA	CFSAN029692	SRR1908958	2014	4	North America	United States	Enteritidis (Predicted)	Enteritidis	1425	23891
SAL_CA7472AA	CFSAN029701	SRR1908947	2014	4	North America	United States	Enteritidis (Predicted)	Enteritidis	1425	23884
SAL_CA7488AA	CFSAN029693	SRR1861865	2014	4	North America	United States	Enteritidis (Predicted)	Enteritidis	1425	23868
SAL_CA7490AA	CFSAN029687	SRR1861847	2014	4	North America	United States	Enteritidis (Predicted)	Enteritidis	1425	23866
SAL_CA7491AA	CFSAN029685	SRR1861846	2014	4	North America	United States	Enteritidis (Predicted)	Enteritidis	1425	23865
SAL_CA7497AA	CFSAN029697	SRR1861661	2014	4	North America	United States	Enteritidis (Predicted)	Enteritidis	1425	23860
SAL_CA7514AA	AUCVM-3370	SRR1849400	2008	11	North America	United States	Enteritidis	Enteritidis	1425	23844
SAL_CA7552AA	15MN00034	SRR1828986	2015	1	North America	United States	Enteritidis	Enteritidis	8003	23812
SAL_CA7553AA	15MN00031	SRR1828985	2015	1	North America	United States	Enteritidis	Enteritidis	1425	23811
SAL_CA7555AA	15MN00037	SRR1828980	2015	1	North America	United States	Enteritidis	Enteritidis	8003	23809
SAL_CA7556AA	15MN00032	SRR1828979	2015	1	North America	United States	Enteritidis	Enteritidis	1425	23808
SAL_CA7557AA	15MN00030	SRR1828978	2015	1	North America	United States	Enteritidis	Enteritidis	1425	23807

SAL_CA7558AA	15MN00029	SRR1828977	2015	1	North America	United States	Enteritidis	Enteritidis	1425	23806
SAL_CA7560AA	15MN00052	SRR1828973	2015	1	North America	United States	Enteritidis	Enteritidis	1425	15921
SAL_CA7561AA	15MN00042	SRR1828972	2015	1	North America	United States	Enteritidis	Enteritidis	1425	23804
SAL_CA7562AA	15MN00051	SRR1828971	2015	1	North America	United States	Enteritidis	Enteritidis	1425	23803
SAL_CA7563AA	15MN00033	SRR1828970	2015	1	North America	United States	Enteritidis	Enteritidis	1425	23802
SAL_CA7564AA	15MN00035	SRR1828969	2015	1	North America	United States	Enteritidis	Enteritidis	1425	23801
SAL_CA7565AA	15MN00036	SRR1828968	2015	1	North America	United States	Enteritidis	Enteritidis	1425	22169
SAL_CA7566AA	15MN00043	SRR1828967	2015	1	North America	United States	Enteritidis	Enteritidis	1425	23800
SAL_CA7567AA	PNUSAS000171	SRR1824479	2014	12	North America	United States	Enteritidis	Enteritidis	1425	23799
SAL_CA7568AA	PNUSAS000170	SRR1824478	2014	12	North America	United States	Enteritidis	Enteritidis	1425	12241
SAL_CA7569AA	PNUSAS000167	SRR1824476	2014	12	North America	United States	Enteritidis	Enteritidis	1425	23798
SAL_CA7570AA	PNUSAS000168	SRR1824475	2014	12	North America	United States	Enteritidis	Enteritidis	1425	23797
SAL_CA7571AA	PNUSAS000169	SRR1824473	2014	12	North America	United States	Enteritidis	Enteritidis	1425	23796
SAL_CA7591AA	FDA00003185	SRR1823704	2009	9	Asia	Indonesia	Enteritidis	Enteritidis	1425	23780
SAL_CA7601AA	FL-NRM274	SRR1823687	2006		North America	United States	Enteritidis	Enteritidis	1425	23771
SAL_CA7620AA	PNUSAS000149	SRR1822478	2015	1	North America	United States	Enteritidis	Enteritidis	1425	23757
SAL_CA7635AA	WAPHL_SAL-A00700	SRR1822299	2006		North America	United States	Enteritidis	Enteritidis	1425	23747
SAL_CA7646AA	15MN00060	SRR1822285	2015	2	North America	United States	Enteritidis	Enteritidis	1425	23738
SAL_CA7650AA	15MN00063	SRR1822281	2015	2	North America	United States	Enteritidis	Enteritidis	1425	23736
SAL_CA7651AA	15MN00062	SRR1822280	2015	2	North America	United States	Enteritidis	Enteritidis	1425	23735
SAL_CA7652AA	15MN00059	SRR1822279	2015	2	North America	United States	Enteritidis	Enteritidis	1425	23734
SAL_CA7653AA	15MN00058	SRR1822278	2015	2	North America	United States	Enteritidis	Enteritidis	1425	17527
SAL_CA7656AA	15MN00061	SRR1822275	2015	2	North America	United States	Enteritidis	Enteritidis	1425	23731
SAL_CA7743AA	CFSAN028645	SRR1813457	2014	3	North America	United States	Enteritidis (Predicted)	Enteritidis	1425	23658
SAL_CA7756AA	15MN00057	SRR1813444	2015	2	North America	United States	Enteritidis	Enteritidis	1425	23647
SAL_CA7766AA	15MN00056	SRR1813434	2015	2	North America	United States	Enteritidis	Enteritidis	1425	23637
SAL_CA7770AA	CFSAN028641	SRR1813430	2014	3	North America	United States	Enteritidis (Predicted)	Enteritidis	1425	23633
SAL_CA7776AA	CFSAN028636	SRR1813424	2014	3	North America	United States	Enteritidis (Predicted)	Enteritidis	1425	23629
SAL_CA7804AA	CFSAN027388	SRR1812866	2012		North America	United States	Enteritidis	Enteritidis	1425	23602
SAL_CA7806AA	CFSAN027394	SRR1812863	2012		North America	United States	Enteritidis	Enteritidis	1425	14326
SAL_CA7819AA	CFSAN027389	SRR1812849	2012		North America	United States	Enteritidis	Enteritidis	1425	23589
SAL_CA7837AA	CFSAN027378	SRR1812826	2012		North America	United States	Enteritidis	Enteritidis	1425	23574
SAL_CA7857AA	CFSAN027377	SRR1812801	2012		North America	United States	Enteritidis	Enteritidis	1425	23556
SAL_CA7870AA	2015K-0047	SRR1812342			North America	United States	Enteritidis (Predicted)	Enteritidis	1425	23543
SAL_CA7878AA	15MN00054	SRR1811675	2015	2	North America	United States	Enteritidis	Enteritidis	1425	23535
SAL_CA7879AA	15MN00050	SRR1811628	2015	2	North America	United States	Enteritidis	Enteritidis	1425	23534
SAL_CA7880AA	NY_swgs1001	SRR974686	2013	8	North America	United States	Enteritidis (Predicted)	Enteritidis	1425	23533
SAL_CA7909AA	CFSAN028609	SRR1810569	2014	3	North America	United States	Enteritidis (Predicted)	Enteritidis	28208	23509
SAL_CA7916AA	CFSAN028615	SRR1810557	2014	3	North America	United States	Enteritidis (Predicted)	Enteritidis	27901	23503
SAL_CA7971AA	CFSAN028592	SRR1805655	2014	3	North America	United States	Enteritidis (Predicted)	Enteritidis	1425	23461
SAL_CA8006AA	15MN00023	SRR1805601	2015	1	North America	United States	Enteritidis	Enteritidis	1425	23427
SAL_CA8028AA	WAPHL_SAL-A00684	SRR1805577	2006		North America	United States	Enteritidis	Enteritidis	1425	23405
SAL_CA8075AA	IEH-NGS-SAL-01942	SRR1803038	2014	3	North America	United States	Enteritidis (Predicted)	Enteritidis	1425	23368
SAL_CA8082AA	FDA00001583	SRR1802986	2001	4	North America	United States	Enteritidis	Enteritidis	1425	16461
SAL_CA8084AA	CFSAN028568	SRR1802984	2014	3	North America	United States	Enteritidis (Predicted)	Enteritidis	1425	23361
SAL_CA8089AA	FDA00001582	SRR1802935	2001	4	North America	United States	Enteritidis	Enteritidis	1425	23357
SAL_CA8095AA	FDA00000054	SRR1802918	2001	4	North America	Canada	Enteritidis	Enteritidis	1425	23353
SAL_CA8115AA	PNUSAS000162	SRR1802280			North America	United States	Enteritidis (Predicted)	Enteritidis	1425	23337
SAL_CA8130AA	FL-NRM244	SRR1801551	2006		North America	United States	Enteritidis	Enteritidis	1425	23323
SAL_CA8141AA	IEH-NGS-SAL-01922	SRR1799716	2014	3	North America	United States	Enteritidis (Predicted)	Enteritidis	1425	23312
SAL_CA8142AA	IEH-NGS-SAL-01941	SRR1799714	2014	3	North America	United States	Enteritidis (Predicted)	Enteritidis	1425	10271
SAL_CA8148AA	CFSAN028061	SRR1799708	2014	3	North America	United States	Enteritidis (Predicted)	Enteritidis	1425	23306
SAL_CA8149AA	CFSAN028053	SRR1799707	2014	3	North America	United States	Enteritidis (Predicted)	Enteritidis	1425	23305
SAL_CA8156AA	CFSAN028056	SRR1799700	2014	3	North America	United States	Enteritidis (Predicted)	Enteritidis	1425	23298
SAL_CA8159AA	CFSAN028027	SRR1796135	2014	1	North America	United States	Enteritidis (Predicted)	Enteritidis	1425	23295
SAL_CA8168AA	IEH-NGS-SAL-01907	SRR1796098	2014	1	North America	United States	Enteritidis (Predicted)	Enteritidis	1425	23286
SAL_CA8172AA	CFSAN028028	SRR1796066	2014	1	North America	United States	Enteritidis (Predicted)	Enteritidis	1425	23282
SAL_CA8234AA	FL-NRM220	SRR1793258	2006		North America	United States	Enteritidis	Enteritidis	1425	23222
SAL_CA8258AA	FDA00001501	SRR1792725	2010	12	Asia	Taiwan	Enteritidis	Enteritidis	28195	23199

SAL_CA8291AA	FL-NRM203	SRR1791289	2005		North America	United States	Enteritidis	Enteritidis	1425	23170
SAL_CA8301AA	FL-NRM202	SRR1791272	2005		North America	United States	Enteritidis	Enteritidis	1425	23162
SAL_CA8310AA	PNUSAS000148	SRR1784332	2014	12	North America	United States	Enteritidis	Enteritidis	1425	23157
SAL_CA8311AA	PNUSAS000147	SRR1784330			North America	United States	Enteritidis (Predicted)	Enteritidis	1425	23156
SAL_CA8312AA	PNUSAS000146	SRR1784329			North America	United States	Enteritidis (Predicted)	Enteritidis	1425	23155
SAL_CA8313AA	PNUSAS000145	SRR1784328			North America	United States	Enteritidis (Predicted)	Enteritidis	28194	23154
SAL_CA8350AA	NY-33944	SRR1783182	2003		North America	United States	Enteritidis	Enteritidis	1425	23123
SAL_CA8462AA	FDA00000897	SRR1777689	2005	10	Asia	China	Enteritidis	Enteritidis	1425	23028
SAL_CA8483AA	FL-NRM169	SRR1777668	2005		North America	United States	Enteritidis	Enteritidis	1425	23009
SAL_CA8494AA	CFSAN028523	SRR1774102	2012		North America	United States	Enteritidis	Enteritidis	1425	22999
SAL_CA8521AA	CFSAN028525	SRR1774059	2012		North America	United States	Enteritidis	Enteritidis	1425	22977
SAL_CA8527AA	CFSAN028530	SRR1774053	2012		North America	United States	Enteritidis	Enteritidis	1425	22973
SAL_CA8529AA	15MN00025	SRR1774023	2015	1	North America	United States	Enteritidis	Enteritidis	1425	9531
SAL_CA8532AA	WAPHL_SAL-A00674	SRR1774020	2014	11	North America	United States	Enteritidis	Enteritidis	1425	14335
SAL_CA8534AA	15MN00022	SRR1774018	2015	1	North America	United States	Enteritidis	Enteritidis	26832	22968
SAL_CA8536AA	WAPHL_SAL-A00677	SRR1774016	2014	11	North America	United States	Enteritidis	Enteritidis	1425	22966
SAL_CA8537AA	WAPHL_SAL-A00670	SRR1774015	2014	10	North America	United States	Enteritidis	Enteritidis	1425	22292
SAL_CA8538AA	WAPHL_SAL-A00669	SRR1774014	2014	10	North America	United States	Enteritidis	Enteritidis	1425	22965
SAL_CA8540AA	15MN00028	SRR1773839	2015	1	North America	United States	Enteritidis	Enteritidis	1425	22964
SAL_CA8552AA	15MN00027	SRR1773636	2015	1	North America	United States	Enteritidis	Enteritidis	1425	22955
SAL_CA8557AA	WAPHL_SAL-A00676	SRR1773596	2014	11	North America	United States	Enteritidis	Enteritidis	1425	22950
SAL_CA8558AA	15MN00026	SRR1773594	2015	1	North America	United States	Enteritidis	Enteritidis	1425	22949
SAL_CA8559AA	15MN00024	SRR1773593	2015	1	North America	United States	Enteritidis	Enteritidis	1425	22948
SAL_CA8660AA	15MN00021	SRR1765792	2015	1	North America	United States	Enteritidis	Enteritidis	1425	22863
SAL_CA8661AA	15MN00020	SRR1765791	2015	1	North America	United States	Enteritidis	Enteritidis	1425	22862
SAL_CA8662AA	15MN00004	SRR1765788	2015	1	North America	United States	Enteritidis	Enteritidis	1425	22861
SAL_CA8663AA	15MN00003	SRR1765787	2015	1	North America	United States	Enteritidis	Enteritidis	26832	22860
SAL_CA8664AA	15MN00002	SRR1765786	2015	1	North America	United States	Enteritidis	Enteritidis	1425	22859
SAL_CA8684AA	CFSAN026628	SRR1763865	2007		Europe	Belgium	Enteritidis	Enteritidis	1425	22841
SAL_CA8686AA	CFSAN026633	SRR1763862	2013		Europe	Belgium	Enteritidis	Enteritidis	1425	22839
SAL_CA8687AA	CFSAN026627	SRR1763861	2002		Europe	Belgium	Enteritidis	Enteritidis	1425	22838
SAL_CA8690AA	CFSAN026639	SRR1763856	2011		Europe	Belgium	Enteritidis	Enteritidis	1425	22835
SAL_CA8705AA	CFSAN026640	SRR1763836	2011		Europe	Belgium	Enteritidis	Enteritidis	1425	22820
SAL_CA8709AA	CFSAN026634	SRR1763830	2014		Europe	Belgium	Enteritidis	Enteritidis	1425	22817
SAL_CA8712AA	CFSAN026636	SRR1763827	2013		Europe	Belgium	Enteritidis	Enteritidis	-1731	22814
SAL_CA8716AA	CFSAN026641	SRR1763822	2011		Europe	Belgium	Enteritidis	Enteritidis	3888	22810
SAL_CA8717AA	CFSAN026631	SRR1763821	2010		Europe	Belgium	Enteritidis	Enteritidis	1425	22809
SAL_CA8718AA	CFSAN026630	SRR1763820	2009		Europe	Belgium	Enteritidis	Enteritidis	1425	22808
SAL_CA8719AA	CFSAN026629	SRR1763819	2008		Europe	Belgium	Enteritidis	Enteritidis	1425	22807
SAL_CA8728AA	CFSAN026632	SRR1763807	2012		Europe	Belgium	Enteritidis	Enteritidis	1425	22800
SAL_CA8730AA	CFSAN026642	SRR1763803	2011		Europe	Belgium	Enteritidis	Enteritidis	3888	22798
SAL_CA8731AA	CFSAN026638	SRR1763802	2011		Europe	Belgium	Enteritidis	Enteritidis	3888	22797
SAL_CA8732AA	CFSAN026637	SRR1763801	2010		Europe	Belgium	Enteritidis	Enteritidis	1425	22796
SAL_CA8754AA	MDH-2014-00967	SRR1576829	2014	9	North America	United States	Enteritidis	Enteritidis	1425	18563
SAL_CA8760AA	AUCVM-2480	SRR1752896	1990	6	North America	United States	Enteritidis	Enteritidis	1425	22770
SAL_CA8761AA	AUCVM-775	SRR1752895	2001	9	North America	United States	Enteritidis	Enteritidis	1425	22771
SAL_CA8762AA	WAPHL_SAL-A00673	SRR1752894	2014	10	North America	United States	Enteritidis	Enteritidis	1425	22715
SAL_CA8766AA	MDH-2014-01089	SRR1752890	2014	12	North America	United States	Enteritidis	Enteritidis	1425	22767
SAL_CA8769AA	AZ-TG74584	SRR1752887	2005		North America	United States	Enteritidis	Enteritidis	1425	22764
SAL_CA8773AA	AZ-TG74476	SRR1752882	2005		North America	United States	Enteritidis	Enteritidis	1425	22761
SAL_CA8783AA	WAPHL_SAL-A00678	SRR1752871	2014	12	North America	United States	Enteritidis	Enteritidis	8003	22752
SAL_CA8787AA	AUCVM-1614	SRR1752867	1990	2	North America	United States	Enteritidis	Enteritidis	1425	22748
SAL_CA8794AA	AUCVM-279	SRR1752853	1990	8	North America	United States	Enteritidis	Enteritidis	1425	22741
SAL_CA8801AA	WAPHL_SAL-A00671	SRR1752835	2014	10	North America	United States	Enteritidis	Enteritidis	1425	22734
SAL_CA8805AA	AUCVM-420	SRR1752828	2006	8	North America	United States	Enteritidis	Enteritidis	7801	22730
SAL_CA8810AA	AUCVM-2640	SRR1752814	2004	2	North America	United States	Enteritidis	Enteritidis	1425	22725
SAL_CA8812AA	WAPHL_SAL-A00668	SRR1752812	2014	10	North America	United States	Enteritidis	Enteritidis	1425	22724
SAL_CA8815AA	WAPHL_SAL-A00681	SRR1752809	2014	12	North America	United States	Enteritidis	Enteritidis	1425	22721
SAL_CA8821AA	WAPHL_SAL-A00682	SRR1752785	2014	12	North America	United States	Enteritidis	Enteritidis	1425	22716

SAL_CA8822AA	WAPHL_SAL-A00679	SRR1752784	2014	11	North America	United States	Enteritidis	Enteritidis	1425	22715
SAL_CA8823AA	AUCVM-1660	SRR1752783	1994	1	North America	United States	Enteritidis	Enteritidis	7801	22714
SAL_CA8828AA	WAPHL_SAL-A00675	SRR1752778	2014	10	North America	United States	Enteritidis	Enteritidis	1425	22710
SAL_CA8829AA	WAPHL_SAL-A00683	SRR1752739	2014	12	North America	United States	Enteritidis	Enteritidis	1425	22709
SAL_CA8830AA	12D14456	SRR1752737	2012	9	North America	United States	Enteritidis	Enteritidis	1425	22708
SAL_CA8837AA	15MN00001	SRR1752724	2014	12	North America	United States	Enteritidis	Enteritidis	1425	22702
SAL_CA8838AA	WAPHL_SAL-A00680	SRR1752723	2014	11	North America	United States	Enteritidis	Enteritidis	1425	22701
SAL_CA8855AA	FDA00001350	SRR1745625	2009	7	Asia	China	Enteritidis	Enteritidis	1425	22689
SAL_CA8887AA	CFSAN024814	SRR1745591	2012		North America	United States	Enteritidis	Enteritidis	1425	18646
SAL_CA8900AA	CFSAN024813	SRR1745577	2012		North America	United States	Enteritidis	Enteritidis	26162	22651
SAL_CA8999AA	FDA00000603	SRR1743998	2004	2	South America	Ecuador	Enteritidis	Enteritidis	1425	22578
SAL_CA9014AA	PNUSAS000125	SRR1738017	2014	10	North America	United States	Enteritidis	Enteritidis	1425	22564
SAL_CA9089AA	FDA00003514	SRR1732585	2011	10	North America	United States	Enteritidis	Enteritidis	1425	12822
SAL_CA9205AA	PNUSAS000123	SRR1724679	2014	11	North America	United States	Enteritidis	Enteritidis	1425	22404
SAL_CA9209AA	PNUSAS000118	SRR1724675	2014	10	North America	United States	Enteritidis	Enteritidis	1425	22401
SAL_CA9210AA	PNUSAS000117	SRR1724674	2014	10	North America	United States	Enteritidis	Enteritidis	1425	22400
SAL_CA9211AA	PNUSAS000116	SRR1724673	2014	10	North America	United States	Enteritidis	Enteritidis	1425	22399
SAL_CA9213AA	2014K-1007	SRR1724671	2014	11	North America	United States	Enteritidis	Enteritidis	1425	21858
SAL_CA9214AA	2014K-0996	SRR1724670			North America	United States	Enteritidis (Predicted)	Enteritidis	1425	21858
SAL_CA9244AA	CFSAN025693	SRR1720754	2012		North America	United States	Enteritidis	Enteritidis	1425	22379
SAL_CA9262AA	NY-swgs1556	SRR1720477	2014	11	North America	United States	Enteritidis	Enteritidis	1425	22363
SAL_CA9279AA	NY-swgs1557	SRR1720459	2014	11	North America	United States	Enteritidis	Enteritidis	1425	22346
SAL_CA9300AA	MDH-2014-01078	SRR1713649	2014	12	North America	United States	Enteritidis	Enteritidis	1425	22325
SAL_CA9304AA	MDH-2014-01080	SRR1713645	2014	12	North America	United States	Enteritidis	Enteritidis	3888	22322
SAL_CA9309AA	NY-30663	SRR1713640	2003		North America	United States	Enteritidis	Enteritidis	1425	22319
SAL_CA9311AA	MDH-2014-01079	SRR1713637	2014	12	North America	United States	Enteritidis	Enteritidis	1425	21950
SAL_CA9340AA	WAPHL_SAL-A00666	SRR1709664	2014	10	North America	United States	Enteritidis	Enteritidis	1425	22294
SAL_CA9341AA	WAPHL_SAL-A00672	SRR1709633	2014	10	North America	United States	Enteritidis	Enteritidis	1425	22293
SAL_CA9342AA	WAPHL_SAL-A00667	SRR1709600	2014	10	North America	United States	Enteritidis	Enteritidis	1425	22292
SAL_CA9385AA	PNUSAS000073	SRR1703349	2014	10	North America	United States	Enteritidis	Enteritidis	1425	22259
SAL_CA9386AA	PNUSAS000072	SRR1703348			North America	United States	Enteritidis (Predicted)	Enteritidis	1425	22258
SAL_CA9387AA	PNUSAS000071	SRR1703347	2014	9	North America	United States	Enteritidis	Enteritidis	1425	21729
SAL_CA9388AA	PNUSAS000070	SRR1703346			North America	United States	Enteritidis (Predicted)	Enteritidis	1425	22257
SAL_CA9389AA	PNUSAS000069	SRR1703345			North America	United States	Enteritidis (Predicted)	Enteritidis	1425	22256
SAL_CA9393AA	2014K-0977	SRR1703341			North America	United States	Enteritidis (Predicted)	Enteritidis	1425	22248
SAL_CA9398AA	2014K-0964	SRR1703336	2014	11	North America	United States	Enteritidis	Enteritidis	1425	21858
SAL_CA9402AA	2014K-0949	SRR1703332	2014	10	North America	United States	Enteritidis	Enteritidis	1425	22248
SAL_CA9407AA	2014K-0502-2	SRR1703258			North America	United States	Enteritidis (Predicted)	Enteritidis	27863	22245
SAL_CA9408AA	2014K-0501-2	SRR1703257			North America	United States	Enteritidis (Predicted)	Enteritidis	27863	19990
SAL_CA9409AA	2014K-0499-2	SRR1703256			North America	United States	Enteritidis (Predicted)	Enteritidis	27863	19990
SAL_CA9436AA	NY-30658	SRR1698204	2003		North America	United States	Enteritidis	Enteritidis	28163	22228
SAL_CA9440AA	FDA00004012	SRR1698195	2012	4	Asia	China	Enteritidis	Enteritidis	1425	22224
SAL_CA9457AA	PNUSAS000102	SRR1696968	2014	11	North America	United States	Enteritidis	Enteritidis	1425	22208
SAL_CA9522AA	MDH-2014-01067	SRR1696485	2014	11	North America	United States	Enteritidis	Enteritidis	1425	22169
SAL_CA9541AA	NY-swgs1550	SRR1695773	2014	10	North America	United States	Enteritidis	Enteritidis	7801	22154
SAL_CA9543AA	CFSAN025708	SRR1695770	2012		North America	United States	Enteritidis	Enteritidis	1425	22152
SAL_CA9549AA	CFSAN025699	SRR1695764	2012		North America	United States	Enteritidis	Enteritidis	1425	22149
SAL_CA9551AA	CFSAN024831	SRR1695762	2012		North America	United States	Enteritidis	Enteritidis	1425	22140
SAL_CA9552AA	CFSAN024827	SRR1695761	2012		North America	United States	Enteritidis	Enteritidis	1425	22147
SAL_CA9556AA	CFSAN025717	SRR1695757	2012		North America	United States	Enteritidis	Enteritidis	1425	22143
SAL_CA9557AA	CFSAN025689	SRR1695756	2012		North America	United States	Enteritidis	Enteritidis	1425	22142
SAL_CA9558AA	CFSAN024840	SRR1695755	2012		North America	United States	Enteritidis	Enteritidis	1425	22141
SAL_CA9559AA	CFSAN024832	SRR1695754	2012		North America	United States	Enteritidis	Enteritidis	1425	22140
SAL_CA9561AA	NY-swgs1549	SRR1695752	2014	11	North America	United States	Enteritidis	Enteritidis	8003	22138
SAL_CA9565AA	CFSAN025700	SRR1695748	2012		North America	United States	Enteritidis	Enteritidis	1425	22134
SAL_CA9572AA	NY-swgs1552	SRR1695741	2014	11	North America	United States	Enteritidis	Enteritidis	1425	2668
SAL_CA9581AA	NY-swgs1554	SRR1695628	2014	11	North America	United States	Enteritidis	Enteritidis	1425	22120
SAL_CA9582AA	NY-swgs1547	SRR1695627	2014	11	North America	United States	Enteritidis	Enteritidis	1425	21858
SAL_CA9583AA	NY-swgs1544	SRR1695626	2014	10	North America	United States	Enteritidis	Enteritidis	1425	22119

SAL_CA9588AA	NY-swgs1553	SRR1695616	2014	11	North America	United States	Enteritidis	Enteritidis	1425	22114
SAL_CA9590AA	NY-swgs1548	SRR1695613	2014	11	North America	United States	Enteritidis	Enteritidis	1425	22112
SAL_CA9591AA	NY-swgs1546	SRR1695612	2014	11	North America	United States	Enteritidis	Enteritidis	1425	22111
SAL_CA9592AA	NY-swgs1545	SRR1695611	2014	11	North America	United States	Enteritidis	Enteritidis	1425	22110
SAL_CA9593AA	NY-swgs1543	SRR1695610	2014	11	North America	United States	Enteritidis	Enteritidis	1425	22109
SAL_CA9594AA	NY-swgs1542	SRR1695609	2014	11	North America	United States	Enteritidis	Enteritidis	1425	21858
SAL_CA9601AA	NY-swgs1555	SRR1695600	2014	11	North America	United States	Enteritidis	Enteritidis	1425	21858
SAL_CA9602AA	NY-swgs1551	SRR1695599	2014	11	North America	United States	Enteritidis	Enteritidis	1425	21858
SAL_CA9648AA	PNUSAS000109	SRR1693630	2014	10	North America	United States	Enteritidis	Enteritidis	1425	22065
SAL_CA9650AA	PNUSAS000107	SRR1693628			North America	United States	Enteritidis (Predicted)	Enteritidis	1425	22063
SAL_CA9651AA	PNUSAS000106	SRR1693627			North America	United States	Enteritidis (Predicted)	Enteritidis	1425	22062
SAL_CA9664AA	WAPHL_SAL-A00657	SRR1693399	2014	9	North America	United States	Enteritidis	Enteritidis	1425	22051
SAL_CA9666AA	WAPHL_SAL-A00655	SRR1693394	2014	9	North America	United States	Enteritidis	Enteritidis	1425	22050
SAL_CA9675AA	WAPHL_SAL-A00656	SRR1693302	2014	9	North America	United States	Enteritidis	Enteritidis	1425	22041
SAL_CA9686AA	WAPHL_SAL-A00658	SRR1693282	2014	9	North America	United States	Enteritidis	Enteritidis	1425	22031
SAL_CA9690AA	WAPHL_SAL-A00654	SRR1693275	2014	9	North America	United States	Enteritidis	Enteritidis	1425	22027
SAL_CA9728AA	FDA00000397	SRR1687183	2003	3	Europe	Netherlands	Enteritidis	Enteritidis	1425	21991
SAL_CA9730AA	2014K-0958	SRR1686427	2014	11	North America	United States	Enteritidis	Enteritidis	1425	21858
SAL_CA9734AA	2014K-0950	SRR1686423	2014	10	North America	United States	Enteritidis	Enteritidis	1425	21858
SAL_CA9735AA	2014K-0948	SRR1686422	2014	10	North America	United States	Enteritidis	Enteritidis	1425	21858
SAL_CA9758AA	FDA00002645	SRR1685377	2007	12	Asia	South Korea	Enteritidis	Enteritidis	1425	21971
SAL_CA9784AA	MDH-2014-01062	SRR1664788	2014	11	North America	United States	Enteritidis	Enteritidis	1425	21950
SAL_CA9801AA	PNUSAS000105	SRR1664340			North America	United States	Enteritidis (Predicted)	Enteritidis	1425	21935
SAL_CA9802AA	PNUSAS000104	SRR1664339			North America	United States	Enteritidis (Predicted)	Enteritidis	1425	14319
SAL_CA9803AA	PNUSAS000103	SRR1664338			North America	United States	Enteritidis (Predicted)	Enteritidis	1425	21934
SAL_CA9804AA	PNUSAS000093	SRR1664337			North America	United States	Enteritidis (Predicted)	Enteritidis	1425	21933
SAL_CA9805AA	PNUSAS000092	SRR1664336			North America	United States	Enteritidis (Predicted)	Enteritidis	1425	21932
SAL_CA9806AA	PNUSAS000091	SRR1664335			North America	United States	Enteritidis (Predicted)	Enteritidis	1425	21931
SAL_CA9807AA	PNUSAS000090	SRR1664334			North America	United States	Enteritidis (Predicted)	Enteritidis	1425	21930
SAL_CA9808AA	PNUSAS000089	SRR1664333			North America	United States	Enteritidis (Predicted)	Enteritidis	1425	21929
SAL_CA9809AA	PNUSAS000088	SRR1664332			North America	United States	Enteritidis (Predicted)	Enteritidis	1425	21928
SAL_CA9810AA	PNUSAS000087	SRR1664331			North America	United States	Enteritidis (Predicted)	Enteritidis	1425	21927
SAL_CA9811AA	PNUSAS000086	SRR1664330			North America	United States	Enteritidis (Predicted)	Enteritidis	1425	21926
SAL_CA9812AA	PNUSAS000085	SRR1664329			North America	United States	Enteritidis (Predicted)	Enteritidis	1425	21925
SAL_CA9813AA	PNUSAS000084	SRR1664328	2014	8	North America	United States	Enteritidis	Enteritidis	1425	21924
SAL_CA9814AA	PNUSAS000083	SRR1664327	2014	8	North America	United States	Enteritidis	Enteritidis	1425	21923
SAL_CA9815AA	PNUSAS000082	SRR1664326	2014	8	North America	United States	Enteritidis	Enteritidis	1425	21922
SAL_CA9816AA	PNUSAS000081	SRR1664325	2014	8	North America	United States	Enteritidis	Enteritidis	1425	21921
SAL_CA9817AA	PNUSAS000080	SRR1664324	2014	10	North America	United States	Enteritidis	Enteritidis	1425	14332
SAL_CA9818AA	PNUSAS000079	SRR1664323	2014	8	North America	United States	Enteritidis	Enteritidis	27904	21920
SAL_CA9820AA	MDH-2014-01053	SRR1664320	2014	11	North America	United States	Enteritidis	Enteritidis	1425	21919
SAL_CA9826AA	NY-swgs1539	SRR1664314	2014	10	North America	United States	Enteritidis	Enteritidis	1425	21916
SAL_CA9830AA	NY-swgs1534	SRR1664310	2014	10	North America	United States	Enteritidis	Enteritidis	1425	21912
SAL_CA9835AA	MDH-2014-01052	SRR1664305	2014	11	North America	United States	Enteritidis	Enteritidis	1425	21909
SAL_CA9862AA	NY-swgs1540	SRR1664278	2014	11	North America	United States	Enteritidis	Enteritidis	1425	21892
SAL_CA9881AA	NY-swgs1538	SRR1664256	2014	10	North America	United States	Enteritidis	Enteritidis	1425	21875
SAL_CA9886AA	MDH-2014-01029	SRR1664227	2014	11	North America	United States	Enteritidis	Enteritidis	1425	21871
SAL_CA9892AA	MDH-2014-01050	SRR1664213	2014	11	North America	United States	Enteritidis	Enteritidis	3888	21865
SAL_CA9896AA	NY-swgs1535	SRR1664208	2014	10	North America	United States	Enteritidis	Enteritidis	1425	21862
SAL_CA9898AA	MDH-2014-01054	SRR1664206	2014	11	North America	United States	Enteritidis	Enteritidis	3888	21860
SAL_CA9899AA	MDH-2014-01051	SRR1664205	2014	11	North America	United States	Enteritidis	Enteritidis	26775	21859
SAL_CA9900AA	NY-swgs1537	SRR1664204	2014	10	North America	United States	Enteritidis	Enteritidis	1425	21858
SAL_CA9907AA	NY-swgs1536	SRR1664196	2014	10	North America	United States	Enteritidis	Enteritidis	1425	21853
SAL_CA9923AA	VA-WGS-00364	SRR1660418	2012	7	North America	United States	Enteritidis	Enteritidis	1425	21839
SAL_CA9932AA	VA-WGS-00400	SRR1660409	2014	10	North America	United States	Enteritidis	Enteritidis	1425	21830
SAL_DA0044AA	MDH-2014-01022	SRR1653757	2014	10	North America	United States	Enteritidis	Enteritidis	1425	21738
SAL_DA0045AA	MDH-2014-01018	SRR1653756	2014	11	North America	United States	Enteritidis	Enteritidis	3888	21735
SAL_DA0046AA	MDH-2014-01017	SRR1653755	2014	11	North America	United States	Enteritidis	Enteritidis	1425	21737
SAL_DA0047AA	MDH-2014-01016	SRR1653754	2014	10	North America	United States	Enteritidis	Enteritidis	1425	21736

SAL_DA0048AA	MDH-2014-01021	SRR1653753	2014	11		North America	United States	Enteritidis	Enteritidis	3888	21735
SAL_DA0050AA	MDH-2014-01015	SRR1653721	2014	10		North America	United States	Enteritidis	Enteritidis	1425	21733
SAL_DA0052AA	NY-swgs1533	SRR1653464	2014	10		North America	United States	Enteritidis	Enteritidis	1425	21731
SAL_DA0053AA	NY-swgs1532	SRR1653463	2014	10		North America	United States	Enteritidis	Enteritidis	1425	21730
SAL_DA0054AA	NY-swgs1531	SRR1653462	2014	10		North America	United States	Enteritidis	Enteritidis	1425	21729
SAL_DA0055AA	NY-swgs1530	SRR1653461	2014	10		North America	United States	Enteritidis	Enteritidis	1425	21728
SAL_DA0079AA	FDA00003630	SRR1653429	2010	10		Asia	Iran	Anatum	Enteritidis	1425	21710
SAL_DA0138AA	FDA00000738	SRR1653366	2004	11		Asia	China	Enteritidis	Enteritidis	1425	21655
SAL_DA0171AA	PNUSAS000063	SRR1649580	2014	10		North America	United States	Enteritidis	Enteritidis	1425	21624
SAL_DA0172AA	PNUSAS000062	SRR1649579	2014	10		North America	United States	Enteritidis	Enteritidis	1425	21623
SAL_DA0173AA	PNUSAS000061	SRR1649578	2014	10		North America	United States	Enteritidis	Enteritidis	1425	21622
SAL_DA0174AA	PNUSAS000060	SRR1649577	2014	10		North America	United States	Enteritidis	Enteritidis	1425	14339
SAL_DA0175AA	PNUSAS000059	SRR1649576	2014	9		North America	United States	Enteritidis	Enteritidis	1425	21621
SAL_DA0234AA	WAPHL_SAL-A00652	SRR1648171	2014	9		North America	United States	Enteritidis	Enteritidis	1425	19848
SAL_DA0243AA	WAPHL_SAL-A00653	SRR1648162	2014	9		North America	United States	Enteritidis	Enteritidis	1425	21586
SAL_DA0267AA	AZ-TG74328	SRR1646946	2005			North America	United States	Enteritidis	Enteritidis	1425	21563
SAL_DA0299AA	AZ-TG74236	SRR1646583	2005			North America	United States	Enteritidis	Enteritidis	1425	21533
SAL_DA0353AA	FDA00003512	SRR1646529	2010	6		Asia	Vietnam	Enteritidis	Enteritidis	1425	21490
SAL_DA0370AA	H130100242	SRR1646424	2012	12		Europe	United Kingdom	Enteritidis	Enteritidis	1425	21476
SAL_DA0375AA	H130100241	SRR1646407	2012	12		Europe	United Kingdom	Enteritidis	Enteritidis	1425	21472
SAL_DA0376AA	H122260456	SRR1646406	2012	5		Europe	United Kingdom	Enteritidis	Enteritidis	1425	21471
SAL_DA0378AA	H121660236	SRR1646404	2012	4		Europe	United Kingdom	Enteritidis	Enteritidis	1425	21469
SAL_DA0380AA	H121800465	SRR1646402	2012	4		Europe	United Kingdom	Enteritidis	Enteritidis	3888	21467
SAL_DA0388AA	H122740560	SRR1646394	2012	7		Europe	United Kingdom	Enteritidis	Enteritidis	1425	21461
SAL_DA0389AA	H123740526	SRR1646393	2012	9		Europe	United Kingdom	Enteritidis	Enteritidis	1425	21460
SAL_DA0390AA	H123060331	SRR1646392	2012	7		Europe	United Kingdom	Enteritidis	Enteritidis	1425	21459
SAL_DA0396AA	H122660489	SRR1646385	2012	6		Europe	United Kingdom	Enteritidis	Enteritidis	3900	21178
SAL_DA0407AA	H121220662	SRR1646374	2012	3		Europe	United Kingdom	Enteritidis	Enteritidis	1425	21445
SAL_DA0408AA	H124080667	SRR1646373	2012	10		Europe	United Kingdom	Enteritidis	Enteritidis	1425	21444
SAL_DA0419AA	H124040548	SRR1646362	2012	10		Europe	United Kingdom	Enteritidis	Enteritidis	3888	21433
SAL_DA0421AA	H124220635	SRR1646360	2012	10		Europe	United Kingdom	Enteritidis	Enteritidis	3888	21431
SAL_DA0430AA	H123360564	SRR1646351	2012	8		Europe	United Kingdom	Enteritidis	Enteritidis	3888	21066
SAL_DA0436AA	H124380523	SRR1646289	2012	10				Enteritidis	Enteritidis	3982	21417
SAL_DA0444AA	H121380297	SRR1646281	2012	3		Europe	United Kingdom	Enteritidis	Enteritidis	3888	21410
SAL_DA0445AA	H122940335	SRR1646280	2012	7		Europe	United Kingdom	Enteritidis	Enteritidis	1425	21409
SAL_DA0447AA	H123980391	SRR1646278	2012	9	17	Europe	United Kingdom	Enteritidis	Enteritidis	1425	21407
SAL_DA0454AA	H123420629	SRR1646271	2012	8		Europe	United Kingdom	Enteritidis	Enteritidis	3888	21401
SAL_DA0459AA	H124740804	SRR1646265	2012	11		Europe	United Kingdom	Enteritidis	Enteritidis	1425	20758
SAL_DA0467AA	H124260481	SRR1646256	2012	10		Europe	United Kingdom	Enteritidis	Enteritidis	3888	21390
SAL_DA0471AA	H124420608	SRR1646252	2012	10		Europe	United Kingdom	Enteritidis	Enteritidis	1425	21386
SAL_DA0472AA	H123340556	SRR1646251	2012	8		Europe	United Kingdom	Enteritidis	Enteritidis	1425	21385
SAL_DA0474AA	H122080470	SRR1646249	2012	5		Europe	United Kingdom	Enteritidis	Enteritidis	1425	21384
SAL_DA0480AA	H120540611	SRR1646243	2012	2	1	Europe	United Kingdom	Enteritidis	Enteritidis	1425	21378
SAL_DA0483AA	H120580508	SRR1646240	2012	2		Europe	United Kingdom	Enteritidis	Enteritidis	1425	21375
SAL_DA0484AA	H124380528	SRR1646239	2012	10		Europe	United Kingdom	Enteritidis	Enteritidis	1425	21374
SAL_DA0487AA	H123560713	SRR1646236	2012	8		Europe	United Kingdom	Enteritidis	Enteritidis	1425	21371
SAL_DA0498AA	H122540674	SRR1646225	2012	6		Europe	United Kingdom	Enteritidis	Enteritidis	3888	21360
SAL_DA0499AA	H122880621	SRR1646224	2012	7		Europe	United Kingdom	Enteritidis	Enteritidis	1425	21359
SAL_DA0500AA	H124180493	SRR1646223	2012	10		Europe	United Kingdom	Enteritidis	Enteritidis	1425	21358
SAL_DA0502AA	H124780465	SRR1646206	2012	11		Europe	United Kingdom	Enteritidis	Enteritidis	1425	21356
SAL_DA0508AA	H123120722	SRR1646200	2012	7		Europe	United Kingdom	Enteritidis	Enteritidis	3888	21351
SAL_DA0511AA	H122580623	SRR1646197	2012	6		Europe	United Kingdom	Enteritidis	Enteritidis	1425	21349
SAL_DA0516AA	H124000530	SRR1646192	2012	10		Europe	United Kingdom	Enteritidis	Enteritidis	3969	21344
SAL_DA0521AA	H123800458	SRR1646175	2012	9		Europe	United Kingdom	Enteritidis	Enteritidis	3888	12624
SAL_DA0522AA	H122460487	SRR1646174	2012	6		Europe	United Kingdom	Enteritidis	Enteritidis	1425	21339
SAL_DA0526AA	H121660235	SRR1646170	2012	4				Enteritidis	Enteritidis	1425	21335
SAL_DA0527AA	H122940328	SRR1646169	2012	7		Europe	United Kingdom	Enteritidis	Enteritidis	3888	20809
SAL_DA0529AA	H120440464	SRR1646167	2012	1		Europe	United Kingdom	Enteritidis	Enteritidis	1425	21333
SAL_DA0532AA	H123380358	SRR1646164	2012	8				Enteritidis	Enteritidis	1425	21330

SAL_DA0541AA	H124400512	SRR1646155	2012	10		Europe	United Kingdom	Enteritidis	Enteritidis	1425	21321
SAL_DA0558AA	H124020505	SRR1646138	2012	10		Europe	United Kingdom	Enteritidis	Enteritidis	1425	21306
SAL_DA0563AA	H122420304	SRR1646133	2012	6	7	Europe	United Kingdom	Enteritidis	Enteritidis	1425	21303
SAL_DA0570AA	H124360215	SRR1646126	2012	10		Europe	United Kingdom	Enteritidis	Enteritidis	1425	21296
SAL_DA0573AA	H123540624	SRR1646123	2012	8		Europe	United Kingdom	Enteritidis	Enteritidis	26142	21293
SAL_DA0580AA	H123780494	SRR1646116	2012	9		Europe	United Kingdom	Enteritidis	Enteritidis	1425	21286
SAL_DA0585AA	H123440410	SRR1646111	2012	8		Europe	United Kingdom	Enteritidis	Enteritidis	1425	21282
SAL_DA0597AA	H123640722	SRR1646099	2012	9				Enteritidis	Enteritidis	3941	21271
SAL_DA0609AA	H123800462	SRR1646086	2012	9		Europe	United Kingdom	Enteritidis	Enteritidis	1425	21260
SAL_DA0610AA	H121220667	SRR1646085	2012	3		Europe	United Kingdom	Enteritidis	Enteritidis	1425	21259
SAL_DA0611AA	H124300159	SRR1646084	2012	10		Europe	United Kingdom	Enteritidis	Enteritidis	26142	21258
SAL_DA0612AA	H130220269	SRR1646083	2013	1		Europe	United Kingdom	Enteritidis	Enteritidis	1425	21257
SAL_DA0616AA	H123020532	SRR1646079	2012	7		Europe	United Kingdom	Enteritidis	Enteritidis	3888	20675
SAL_DA0620AA	H123300317	SRR1646075	2012	8		Europe	United Kingdom	Enteritidis	Enteritidis	3906	21250
SAL_DA0621AA	H123120717	SRR1646074	2012	7		Europe	United Kingdom	Enteritidis	Enteritidis	1425	21249
SAL_DA0627AA	H123340537	SRR1646068	2012	8		Europe	United Kingdom	Enteritidis	Enteritidis	1425	21243
SAL_DA0631AA	H123120716	SRR1646064	2012	7		Europe	United Kingdom	Enteritidis	Enteritidis	3888	21239
SAL_DA0634AA	H122540665	SRR1646061	2012	6		Europe	United Kingdom	Enteritidis	Enteritidis	1425	21158
SAL_DA0635AA	H123140502	SRR1646060	2012	8		Europe	United Kingdom	Enteritidis	Enteritidis	3888	21236
SAL_DA0641AA	H124920540	SRR1646054	2012	12		Europe	United Kingdom	Enteritidis	Enteritidis	1425	21230
SAL_DA0662AA	H123220510	SRR1646011	2012	8		Europe	United Kingdom	Enteritidis	Enteritidis	1425	21209
SAL_DA0664AA	H122980207	SRR1646009	2012	7		Europe	United Kingdom	Enteritidis	Enteritidis	3888	21207
SAL_DA0667AA	H123120707	SRR1646006	2012	7		Europe	United Kingdom	Enteritidis	Enteritidis	1425	21204
SAL_DA0673AA	H123800455	SRR1646000	2012	9		Europe	United Kingdom	Enteritidis	Enteritidis	3963	21198
SAL_DA0680AA	H122880601	SRR1645993	2012	7		Europe	United Kingdom	Enteritidis	Enteritidis	3888	21192
SAL_DA0683AA	H123760523	SRR1645990	2012	9		Europe	United Kingdom	Enteritidis	Enteritidis	1425	21190
SAL_DA0696AA	H122580622	SRR1645977	2012	6		Europe	United Kingdom	Enteritidis	Enteritidis	3900	21178
SAL_DA0697AA	H123980394	SRR1645976	2012	9		Europe	United Kingdom	Enteritidis	Enteritidis	1425	21177
SAL_DA0698AA	H124740793	SRR1645975	2012	11		Europe	United Kingdom	Enteritidis	Enteritidis	1425	21176
SAL_DA0701AA	H123940584	SRR1645972	2012	9		Europe	United Kingdom	Enteritidis	Enteritidis	1425	21173
SAL_DA0711AA	H124420605	SRR1645962	2012	10		Europe	United Kingdom	Enteritidis	Enteritidis	3888	21163
SAL_DA0712AA	H123760538	SRR1645961	2012	9		Europe	United Kingdom	Enteritidis	Enteritidis	3961	21162
SAL_DA0716AA	H122460435	SRR1645957	2012	6		Europe	United Kingdom	Enteritidis	Enteritidis	1425	21158
SAL_DA0725AA	H121540989	SRR1645948	2012	4		Europe	United Kingdom	Enteritidis	Enteritidis	1425	21149
SAL_DA0726AA	H124860435	SRR1645947	2012	11		Europe	United Kingdom	Enteritidis	Enteritidis	-1733	21148
SAL_DA0730AA	H124760740	SRR1645943	2012	11		Europe	United Kingdom	Enteritidis	Enteritidis	3888	21145
SAL_DA0735AA	H122260447	SRR1645938	2012	5		Europe	United Kingdom	Enteritidis	Enteritidis	1425	21140
SAL_DA0741AA	H124200164	SRR1645932	2012	10		Europe	United Kingdom	Enteritidis	Enteritidis	1425	21134
SAL_DA0742AA	H123740506	SRR1645931	2012	9		Europe	United Kingdom	Enteritidis	Enteritidis	1425	21133
SAL_DA0745AA	H124340362	SRR1645928	2012	10		Europe	United Kingdom	Enteritidis	Enteritidis	1425	21131
SAL_DA0747AA	H122440435	SRR1645926	2012	6		Europe	United Kingdom	Enteritidis	Enteritidis	3888	21129
SAL_DA0748AA	H120860350	SRR1645925	2012	2		Europe	United Kingdom	Enteritidis	Enteritidis	1425	21128
SAL_DA0750AA	H123060362	SRR1645923	2012	7		Europe	United Kingdom	Enteritidis	Enteritidis	1425	21127
SAL_DA0752AA	H123520427	SRR1645921	2012	8		Europe	United Kingdom	Enteritidis	Enteritidis	1425	21126
SAL_DA0759AA	H124240337	SRR1645914	2012	10		Europe	United Kingdom	Enteritidis	Enteritidis	1425	21118
SAL_DA0766AA	H123600580	SRR1645907	2012	9		Europe	United Kingdom	Enteritidis	Enteritidis	1425	21112
SAL_DA0775AA	H122280366	SRR1645898	2012	5	29	Europe	United Kingdom	Enteritidis	Enteritidis	1425	21103
SAL_DA0776AA	H122540696	SRR1645897	2012	6				Enteritidis	Enteritidis	1425	21102
SAL_DA0777AA	H123340536	SRR1645896	2012	8		Europe	United Kingdom	Enteritidis	Enteritidis	1425	21101
SAL_DA0778AA	H123360592	SRR1645895	2012	8		Europe	United Kingdom	Enteritidis	Enteritidis	3888	21100
SAL_DA0796AA	H122540669	SRR1645877	2012	6	16	Europe	United Kingdom	Enteritidis	Enteritidis	1425	21083
SAL_DA0799AA	H123380359	SRR1645874	2012	8				Enteritidis	Enteritidis	1425	21080
SAL_DA0814AA	H123400445	SRR1645851	2012	8		Europe	United Kingdom	Enteritidis	Enteritidis	3888	21066
SAL_DA0821AA	H123320606	SRR1645844	2012	8		Europe	United Kingdom	Enteritidis	Enteritidis	3888	21059
SAL_DA0827AA	H122720544	SRR1645838	2012	7		Europe	United Kingdom	Enteritidis	Enteritidis	3888	21054
SAL_DA0838AA	H122880692	SRR1645827	2012	7		Europe	United Kingdom	Enteritidis	Enteritidis	1425	21044
SAL_DA0841AA	H123300320	SRR1645824	2012	8		Europe	United Kingdom	Enteritidis	Enteritidis	1425	21041
SAL_DA0842AA	H125140343	SRR1645823	2012	12		Europe	United Kingdom	Enteritidis	Enteritidis	1425	21040
SAL_DA0844AA	H123420612	SRR1645821	2012	8		Europe	United Kingdom	Enteritidis	Enteritidis	1425	20186

SAL_DA0845AA	H121080598	SRR1645820	2012	3		Europe	United Kingdom	Enteritidis	Enteritidis	1425	21038
SAL_DA0851AA	H122100471	SRR1645814	2012	5		Europe	United Kingdom	Enteritidis	Enteritidis	3888	21032
SAL_DA0858AA	H120440462	SRR1645807	2012	1		Europe	United Kingdom	Enteritidis	Enteritidis	1425	21025
SAL_DA0864AA	H123560705	SRR1645801	2012	8		Europe	United Kingdom	Enteritidis	Enteritidis	3888	21020
SAL_DA0872AA	H123420621	SRR1645793	2012	8		Europe	United Kingdom	Enteritidis	Enteritidis	1425	21013
SAL_DA0876AA	H122720531	SRR1645789	2012	7		Europe	United Kingdom	Enteritidis	Enteritidis	3906	21009
SAL_DA0883AA	H123580521	SRR1645782	2012	8	22	Europe	United Kingdom	Enteritidis	Enteritidis	1425	21002
SAL_DA0887AA	H123900517	SRR1645778	2012	9		Europe	United Kingdom	Enteritidis	Enteritidis	3949	20998
SAL_DA0900AA	H123640725	SRR1645765	2012	9		Europe	United Kingdom	Enteritidis	Enteritidis	1425	20988
SAL_DA0905AA	H121940508	SRR1645760	2012	5		Europe	United Kingdom	Enteritidis	Enteritidis	1425	20983
SAL_DA0908AA	H120880463	SRR1645757	2012	2		Europe	United Kingdom	Enteritidis	Enteritidis	3888	20980
SAL_DA0909AA	H122240473	SRR1645756	2012	5		Europe	United Kingdom	Enteritidis	Enteritidis	1425	20979
SAL_DA0914AA	H122680469	SRR1645751	2012	6		Europe	United Kingdom	Enteritidis	Enteritidis	1425	20974
SAL_DA0916AA	H123480491	SRR1645749	2012	8		Europe	United Kingdom	Enteritidis	Enteritidis	1425	20972
SAL_DA0923AA	H121760528	SRR1645742	2012	4				Enteritidis	Enteritidis	1425	20966
SAL_DA0927AA	H123280504	SRR1645738	2012	8		Europe	United Kingdom	Enteritidis	Enteritidis	3888	20963
SAL_DA0937AA	H125060662	SRR1645728	2012	12		Europe	United Kingdom	Enteritidis	Enteritidis	1425	20954
SAL_DA0938AA	H123560767	SRR1645727	2012	8		Europe	United Kingdom	Enteritidis	Enteritidis	1425	20953
SAL_DA0952AA	H124020450	SRR1645649	2012	10		Europe	United Kingdom	Enteritidis	Enteritidis	3888	20944
SAL_DA0953AA	H122740577	SRR1645648	2012	7		Europe	United Kingdom	Enteritidis	Enteritidis	1425	20943
SAL_DA0958AA	H120720534	SRR1645643	2012	2		Europe	United Kingdom	Enteritidis	Enteritidis	1425	20938
SAL_DA0960AA	H122820788	SRR1645641	2012	7		Europe	United Kingdom	Enteritidis	Enteritidis	1425	20936
SAL_DA0977AA	H124400517	SRR1645602	2012	10		Europe	United Kingdom	Enteritidis	Enteritidis	3888	20921
SAL_DA0979AA	H123420628	SRR1645600	2012	8		Europe	United Kingdom	Enteritidis	Enteritidis	1425	20919
SAL_DA0990AA	H121940486	SRR1645582	2012	5		Europe	United Kingdom	Enteritidis	Enteritidis	1425	20909
SAL_DA0991AA	H123600545	SRR1645581	2012	9		Europe	United Kingdom	Enteritidis	Enteritidis	3888	20169
SAL_DA0993AA	H122820782	SRR1645579	2012	7		Europe	United Kingdom	Enteritidis	Enteritidis	3906	20907
SAL_DA0998AA	H121980557	SRR1645574	2012	5		Europe	United Kingdom	Enteritidis	Enteritidis	1425	20902
SAL_DA1016AA	H123440412	SRR1645556	2012	8		Europe	United Kingdom	Enteritidis	Enteritidis	3888	20884
SAL_DA1022AA	H122780286	SRR1645550	2012	7		Europe	United Kingdom	Enteritidis	Enteritidis	1425	20878
SAL_DA1024AA	H124640511	SRR1645548	2012	11		Europe	United Kingdom	Enteritidis	Enteritidis	3888	20876
SAL_DA1026AA	H123640721	SRR1645546	2012	9		Europe	United Kingdom	Enteritidis	Enteritidis	3888	20874
SAL_DA1029AA	H123160664	SRR1645543	2012	8		Europe	United Kingdom	Enteritidis	Enteritidis	3888	20871
SAL_DA1040AA	H124840245	SRR1645532	2012	11		Europe	United Kingdom	Enteritidis	Enteritidis	3888	20861
SAL_DA1051AA	H124020441	SRR1645464	2012	10		Europe	United Kingdom	Enteritidis	Enteritidis	1425	20851
SAL_DA1063AA	H124340310	SRR1645452	2012	10		Europe	United Kingdom	Enteritidis	Enteritidis	3888	20839
SAL_DA1065AA	H122920419	SRR1645450	2012	7		Europe	United Kingdom	Enteritidis	Enteritidis	3888	20838
SAL_DA1066AA	H120240588	SRR1645449	2012	1		Europe	United Kingdom	Enteritidis	Enteritidis	1425	20837
SAL_DA1070AA	H123740519	SRR1645445	2012	9	3	Europe	United Kingdom	Enteritidis	Enteritidis	1425	20833
SAL_DA1075AA	H123540642	SRR1645440	2012	8		Europe	United Kingdom	Enteritidis	Enteritidis	3888	20828
SAL_DA1080AA	H122360417	SRR1645435	2012	6		Europe	United Kingdom	Enteritidis	Enteritidis	3897	20823
SAL_DA1083AA	H120860352	SRR1645432	2012	2		Europe	United Kingdom	Enteritidis	Enteritidis	1425	20820
SAL_DA1094AA	H123060335	SRR1645396	2012	7		Europe	United Kingdom	Enteritidis	Enteritidis	3888	20809
SAL_DA1095AA	H121760530	SRR1645395	2012	4		Europe	United Kingdom	Enteritidis	Enteritidis	1425	20808
SAL_DA1117AA	H122860674	SRR1645373	2012	7		Europe	United Kingdom	Enteritidis	Enteritidis	3914	20788
SAL_DA1125AA	H124900685	SRR1645365	2012	12		Europe	United Kingdom	Enteritidis	Enteritidis	1425	20780
SAL_DA1139AA	H123160634	SRR1645351	2012	8		Europe	United Kingdom	Enteritidis	Enteritidis	3888	20766
SAL_DA1147AA	H122740562	SRR1645343	2012	7		Europe	United Kingdom	Enteritidis	Enteritidis	3888	20169
SAL_DA1148AA	H122400345	SRR1645338	2012	6				Enteritidis	Enteritidis	1425	20758
SAL_DA1150AA	H123400449	SRR1645335	2012	8		Europe	United Kingdom	Enteritidis	Enteritidis	3888	20756
SAL_DA1156AA	H123340544	SRR1645326	2012	8		Europe	United Kingdom	Enteritidis	Enteritidis	1425	20750
SAL_DA1160AA	H124120610	SRR1645295	2012	10		Europe	United Kingdom	Enteritidis	Enteritidis	1425	20746
SAL_DA1176AA	H123860321	SRR1645271	2012	9		Europe	United Kingdom	Enteritidis	Enteritidis	1425	20731
SAL_DA1181AA	H123740522	SRR1645266	2012	9		Europe	United Kingdom	Enteritidis	Enteritidis	3888	20726
SAL_DA1183AA	H122660488	SRR1645264	2012	6		Europe	United Kingdom	Enteritidis	Enteritidis	1425	20724
SAL_DA1186AA	H122040338	SRR1645257	2012	5		Europe	United Kingdom	Enteritidis	Enteritidis	1425	20721
SAL_DA1189AA	H124760731	SRR1645253	2012	11		Europe	United Kingdom	Enteritidis	Enteritidis	1425	20718
SAL_DA1190AA	H122380504	SRR1645252	2012	6		Europe	United Kingdom	Enteritidis	Enteritidis	1425	20717
SAL_DA1192AA	H120980502	SRR1645250	2012	3		Europe	United Kingdom	Enteritidis	Enteritidis	1425	20715

SAL_DA1193AA	H121420346	SRR1645249	2012	4		Europe	United Kingdom	Enteritidis	Enteritidis	1425	20714
SAL_DA1200AA	H124000535	SRR1645242	2012	10		Europe	United Kingdom	Enteritidis	Enteritidis	1425	20708
SAL_DA1201AA	H123640728	SRR1645241	2012	9		Europe	United Kingdom	Enteritidis	Enteritidis	1425	20707
SAL_DA1202AA	H123540620	SRR1645240	2012	8		Europe	United Kingdom	Enteritidis	Enteritidis	3941	20706
SAL_DA1209AA	H121860585	SRR1645233	2012	5		Europe	United Kingdom	Enteritidis	Enteritidis	1425	20700
SAL_DA1210AA	H121960504	SRR1645232	2012	5		Europe	United Kingdom	Enteritidis	Enteritidis	1425	20699
SAL_DA1219AA	H122660493	SRR1645223	2012	6		Europe	United Kingdom	Enteritidis	Enteritidis	3888	20190
SAL_DA1235AA	H123060355	SRR1645207	2012	7		Europe	United Kingdom	Enteritidis	Enteritidis	3888	20675
SAL_DA1241AA	H122420299	SRR1645200	2012	6		Europe	United Kingdom	Enteritidis	Enteritidis	1425	20669
SAL_DA1244AA	H122620655	SRR1645197	2012	6		Europe	United Kingdom	Enteritidis	Enteritidis	3888	20666
SAL_DA1245AA	H123220515	SRR1645196	2012	8		Europe	United Kingdom	Enteritidis	Enteritidis	1425	20665
SAL_DA1256AA	H125140342	SRR1645185	2012	12		Europe	United Kingdom	Enteritidis	Enteritidis	1425	20654
SAL_DA1263AA	H123540621	SRR1645178	2012	8		Europe	United Kingdom	Enteritidis	Enteritidis	1425	20649
SAL_DA1278AA	H124040553	SRR1645163	2012	10		Europe	United Kingdom	Enteritidis	Enteritidis	1425	20634
SAL_DA1281AA	H124140614	SRR1645160	2012	10		Europe	United Kingdom	Enteritidis	Enteritidis	3888	20631
SAL_DA1295AA	H124080630	SRR1645145	2012	10		Europe	United Kingdom	Enteritidis	Enteritidis	1425	20619
SAL_DA1300AA	H124220625	SRR1645140	2012	10		Europe	United Kingdom	Enteritidis	Enteritidis	1425	20614
SAL_DA1306AA	NY-swgs1525	SRR1645058	2014	10		North America	United States	Enteritidis	Enteritidis	1425	20608
SAL_DA1309AA	NY-swgs1523	SRR1645055	2014	10		North America	United States	Enteritidis	Enteritidis	1425	20605
SAL_DA1310AA	NY-swgs1522	SRR1645054	2014	9		North America	United States	Enteritidis	Enteritidis	1425	20604
SAL_DA1312AA	NY-swgs1526	SRR1644560	2014	10		North America	United States	Enteritidis	Enteritidis	1425	2690
SAL_DA1315AA	NY-swgs1527	SRR1644554	2014	10		North America	United States	Enteritidis	Enteritidis	1425	11696
SAL_DA1318AA	NY-swgs1524	SRR1644550	2014	10		North America	United States	Enteritidis	Enteritidis	1425	20598
SAL_DA1442AA	MDH-2014-00966	SRR1576830	2014	9		North America	United States	Enteritidis	Enteritidis	8003	12492
SAL_DA1541AA	FDA00003515	SRR1638891	2011	10		North America	United States	Enteritidis	Enteritidis	1425	20396
SAL_DA1542AA	MDH-2014-01007	SRR1638888	2014	10		North America	United States	Enteritidis	Enteritidis	1425	20395
SAL_DA1544AA	FDA00003516	SRR1638862	2011	10		North America	United States	Enteritidis	Enteritidis	1425	20394
SAL_DA1552AA	MDH-2014-01008	SRR1638449	2014	10		North America	United States	Enteritidis	Enteritidis	1425	20387
SAL_DA1615AA	WAPHL_SAL-A00659	SRR1636990	2014	7		North America	United States	Enteritidis	Enteritidis	1425	19175
SAL_DA1703AA	NY-swgs1521	SRR1636569	2014	10		North America	United States	Enteritidis	Enteritidis	8183	20269
SAL_DA1704AA	NY-swgs1519	SRR1636568	2014	10		North America	United States	Enteritidis	Enteritidis	1425	20268
SAL_DA1709AA	NY-swgs1518	SRR1636563	2014	10		North America	United States	Enteritidis	Enteritidis	26832	20259
SAL_DA1716AA	NY-swgs1520	SRR1636556	2014	10		North America	United States	Enteritidis	Enteritidis	26832	20259
SAL_DA1723AA	NY-swgs1515	SRR1636549	2014	9		North America	United States	Enteritidis	Enteritidis	1425	20254
SAL_DA1724AA	NY-swgs1514	SRR1636548	2014	9		North America	United States	Enteritidis	Enteritidis	1425	20254
SAL_DA1726AA	NY-swgs1517	SRR1636546	2014	9		North America	United States	Enteritidis	Enteritidis	1425	18599
SAL_DA1747AA	NY-swgs1516	SRR1636522	2014	9		North America	United States	Enteritidis	Enteritidis	1425	18599
SAL_DA1759AA	H120960556	SRR1635128	2012	3		Europe	United Kingdom	Enteritidis	Enteritidis	3888	20225
SAL_DA1769AA	H121760556	SRR1635118	2012	4		Europe	United Kingdom	Enteritidis	Enteritidis	1425	20215
SAL_DA1771AA	H121220661	SRR1635116	2012	3		Europe	United Kingdom	Enteritidis	Enteritidis	1425	20213
SAL_DA1772AA	H122800341	SRR1635115	2012	7		Europe	United Kingdom	Enteritidis	Enteritidis	1425	20212
SAL_DA1773AA	H123940649	SRR1635114	2012	9	21	Europe	United Kingdom	Enteritidis	Enteritidis	1425	20211
SAL_DA1774AA	H121840555	SRR1635113	2012	5		Europe	United Kingdom	Enteritidis (Predicted)	Enteritidis	1425	20210
SAL_DA1784AA	H121560545	SRR1635103	2012	4		Europe	United Kingdom	Enteritidis	Enteritidis	1425	20200
SAL_DA1786AA	H124040561	SRR1635101	2012	10		Europe	United Kingdom	Enteritidis (Predicted)	Enteritidis	3888	12619
SAL_DA1790AA	H124680351	SRR1635097	2012	11	7	Europe	United Kingdom	Enteritidis	Enteritidis	1425	20195
SAL_DA1795AA	H122820787	SRR1635092	2012	7		Europe	United Kingdom	Enteritidis	Enteritidis	3888	20190
SAL_DA1799AA	H122600589	SRR1635087	2012	6		Europe	United Kingdom	Enteritidis	Enteritidis	1425	20186
SAL_DA1811AA	H124260464	SRR1635072	2012	10		Europe	United Kingdom	Enteritidis	Enteritidis	3888	20174
SAL_DA1815AA	H123660400	SRR1635066	2012	9		Europe	United Kingdom	Enteritidis	Enteritidis	1425	20170
SAL_DA1816AA	H123560722	SRR1635065	2012	8		Europe	United Kingdom	Enteritidis	Enteritidis	3888	20169
SAL_DA1818AA	H124220640	SRR1635060	2012	10		Europe	United Kingdom	Enteritidis	Enteritidis	3888	20167
SAL_DA1828AA	2014K-0491	SRR1634588	2014	4		North America	United States	Enteritidis	Enteritidis	1425	19984
SAL_DA1851AA	FDA00000127	SRR1633876	2001	9		Asia	China	Enteritidis	Enteritidis	1425	20143
SAL_DA1858AA	MDH-2014-00987	SRR1633867	2014	10		North America	United States	Enteritidis	Enteritidis	1425	20138
SAL_DA1859AA	NY-swgs1513	SRR1633866	2014	9		North America	United States	Enteritidis	Enteritidis	1425	651
SAL_DA1870AA	MDH-2014-00986	SRR1633854	2014	10		North America	United States	Enteritidis	Enteritidis	1425	20130
SAL_DA1874AA	MDH-2014-00981	SRR1633846	2014	10		North America	United States	Enteritidis	Enteritidis	1425	18563
SAL_DA1878AA	MDH-2014-00983	SRR1633553	2014	10		North America	United States	Enteritidis	Enteritidis	1425	18563

SAL_DA1879AA	NY-swgs1504	SRR1633549	2014	9	North America	United States	Enteritidis	Enteritidis	1425	20123
SAL_DA1883AA	MDH-2014-00998	SRR1633540	2014	10	North America	United States	Enteritidis	Enteritidis	1425	18563
SAL_DA1884AA	MDH-2014-00996	SRR1633539	2014	10	North America	United States	Enteritidis	Enteritidis	1425	20119
SAL_DA1885AA	NY-swgs1507	SRR1633536	2014	9	North America	United States	Enteritidis	Enteritidis	1425	20118
SAL_DA1886AA	FCC0185	SRR1633535	2010	10	North America	United States	Enteritidis	Enteritidis	1425	20117
SAL_DA1887AA	MDH-2014-01002	SRR1633534	2014	10	North America	United States	Enteritidis	Enteritidis	1425	18563
SAL_DA1889AA	MDH-2014-00902	SRR1633529	2014	7	North America	United States	Enteritidis	Enteritidis	1425	20115
SAL_DA1892AA	MDH-2014-00984	SRR1633526	2014	10	North America	United States	Enteritidis	Enteritidis	1425	18563
SAL_DA1895AA	MDH-2014-01000	SRR1633522	2014	10	North America	United States	Enteritidis	Enteritidis	1425	20111
SAL_DA1896AA	MDH-2014-00999	SRR1633521	2014	10	North America	United States	Enteritidis	Enteritidis	1425	18563
SAL_DA1898AA	NY-swgs1509	SRR1633518	2014	9	North America	United States	Enteritidis	Enteritidis	1425	20109
SAL_DA1899AA	NY-swgs1508	SRR1633517	2014	9	North America	United States	Enteritidis	Enteritidis	8003	12492
SAL_DA1900AA	MDH-2014-00903	SRR1633516	2014	8	North America	United States	Enteritidis	Enteritidis	1425	20108
SAL_DA1903AA	FCC0183	SRR1633512	2010	10	North America	United States	Enteritidis	Enteritidis	1425	20105
SAL_DA1904AA	MDH-2014-00997	SRR1633511	2014	10	North America	United States	Enteritidis	Enteritidis	1425	18563
SAL_DA1905AA	NY-swgs1506	SRR1633506	2014	9	North America	United States	Enteritidis	Enteritidis	1425	20104
SAL_DA1907AA	MDH-2014-00982	SRR1633504	2014	10	North America	United States	Enteritidis	Enteritidis	1425	18563
SAL_DA1908AA	MDH-2014-00979	SRR1633503	2014	10	North America	United States	Enteritidis	Enteritidis	1425	18563
SAL_DA1909AA	MDH-2014-00985	SRR1633501	2014	10	North America	United States	Enteritidis	Enteritidis	1425	18563
SAL_DA1913AA	MDH-2014-00988	SRR1633497	2014	10	North America	United States	Enteritidis	Enteritidis	1425	18563
SAL_DA1915AA	MDH-2014-00978	SRR1633495	2014	10	North America	United States	Enteritidis	Enteritidis	1425	20099
SAL_DA1916AA	NY-swgs1505	SRR1633494	2014	9	North America	United States	Enteritidis	Enteritidis	8003	12492
SAL_DA1919AA	FCC0187	SRR1633491	2010	10	North America	United States	Enteritidis	Enteritidis	1425	20097
SAL_DA1920AA	FCC0053	SRR1633490	2010	6	Asia	China	Enteritidis	Enteritidis	1425	20096
SAL_DA1922AA	MDH-2014-00980	SRR1633488	2014	10	North America	United States	Enteritidis	Enteritidis	1425	18563
SAL_DA1938AA	AZ-TG74480	SRR1631184	2005		North America	United States	Enteritidis	Enteritidis	26912	20079
SAL_DA1939AA	AZ-TG74516	SRR1631183	2005		North America	United States	Johannesburg	Enteritidis	1425	20078
SAL_DA1940AA	AZ-TG74448	SRR1631182	2005		North America	United States	Enteritidis	Enteritidis	1425	20077
SAL_DA2040AA	2014K-0528	SRR1616823	2014	6	North America	United States	Enteritidis	Enteritidis	1425	19991
SAL_DA2041AA	2014K-0527	SRR1616822	2014	6	North America	United States	Enteritidis	Enteritidis	1425	19991
SAL_DA2042AA	2014K-0526	SRR1616821	2014	6	North America	United States	Enteritidis	Enteritidis	1425	19991
SAL_DA2043AA	2014K-0525	SRR1616820			North America	United States	Enteritidis	Enteritidis	1425	19991
SAL_DA2044AA	2014K-0502	SRR1616819			North America	United States	Enteritidis	Enteritidis	27863	19990
SAL_DA2045AA	2014K-0501	SRR1616818			North America	United States	Enteritidis	Enteritidis	27863	19989
SAL_DA2046AA	2014K-0500	SRR1616817	2014	5	North America	United States	Enteritidis	Enteritidis	27863	19988
SAL_DA2047AA	2014K-0499	SRR1616816	2014	3	North America	United States	Enteritidis	Enteritidis	27863	19987
SAL_DA2048AA	2014K-0490	SRR1616815	2014	3	North America	United States	Enteritidis	Enteritidis	1425	19984
SAL_DA2049AA	2014K-0487	SRR1616814	2014	5	North America	United States	Enteritidis	Enteritidis	1425	19986
SAL_DA2050AA	2014K-0486	SRR1616813	2014	4	North America	United States	Enteritidis	Enteritidis	1425	19985
SAL_DA2051AA	2014K-0485	SRR1616812	2014	4	North America	United States	Enteritidis	Enteritidis	1425	19984
SAL_DA2071AA	2014K-0184	SRR1616792	2014	1	North America	United States	Enteritidis	Enteritidis	1425	19969
SAL_DA2072AA	2014K-0183	SRR1616791	2014	1	North America	United States	Enteritidis	Enteritidis	1425	19968
SAL_DA2073AA	2014K-0182	SRR1616790			North America	United States	Enteritidis (Predicted)	Enteritidis	1425	19960
SAL_DA2074AA	2014K-0181	SRR1616789			North America	United States	Enteritidis (Predicted)	Enteritidis	1425	19967
SAL_DA2075AA	2014K-0180	SRR1616788			North America	United States	Enteritidis (Predicted)	Enteritidis	1425	19960
SAL_DA2076AA	2014K-0179	SRR1616787			North America	United States	Enteritidis (Predicted)	Enteritidis	1425	14319
SAL_DA2077AA	2014K-0178	SRR1616786			North America	United States	Enteritidis (Predicted)	Enteritidis	1425	19966
SAL_DA2078AA	2014K-0177	SRR1616785			North America	United States	Enteritidis (Predicted)	Enteritidis	1425	19965
SAL_DA2079AA	2014K-0176	SRR1616784			North America	United States	Enteritidis (Predicted)	Enteritidis	1425	19964
SAL_DA2080AA	2014K-0175	SRR1616783			North America	United States	Enteritidis (Predicted)	Enteritidis	1425	14319
SAL_DA2081AA	2014K-0174	SRR1616782	2014	1	North America	United States	Enteritidis	Enteritidis	1425	19963
SAL_DA2082AA	2014K-0173	SRR1616781	2014	1	North America	United States	Enteritidis	Enteritidis	1425	19962
SAL_DA2083AA	2014K-0172	SRR1616780	2014	1	North America	United States	Enteritidis	Enteritidis	1425	14319
SAL_DA2084AA	2014K-0171	SRR2586135	2013	12	North America	United States	Enteritidis	Enteritidis	1425	14319
SAL_DA2085AA	2014K-0170	SRR1616778	2013	12	North America	United States	Enteritidis	Enteritidis	1425	14319
SAL_DA2086AA	2014K-0169	SRR1616777			North America	United States	Enteritidis (Predicted)	Enteritidis	1425	19960
SAL_DA2087AA	2014K-0168	SRR1616776			North America	United States	Enteritidis (Predicted)	Enteritidis	1425	19961
SAL_DA2088AA	2014K-0167	SRR1616775			North America	United States	Enteritidis (Predicted)	Enteritidis	1425	19960
SAL_DA2188AA	WAPHL_SAL-A00632	SRR1615109	2014	8	North America	United States	Enteritidis	Enteritidis	1425	19868

SAL_DA2193AA	WAPHL_SAL-A00640	SRR1615104	2014	9	North America	United States	Enteritidis	Enteritidis	1425	19863
SAL_DA2194AA	WAPHL_SAL-A00633	SRR1615103	2014	8	North America	United States	Enteritidis	Enteritidis	1425	17155
SAL_DA2197AA	WAPHL_SAL-A00638	SRR1615100	2014	9	North America	United States	Enteritidis	Enteritidis	1425	19860
SAL_DA2207AA	WAPHL_SAL-A00630	SRR1615089	2014	8	North America	United States	Enteritidis	Enteritidis	1425	19852
SAL_DA2209AA	WAPHL_SAL-A00637	SRR1615087	2014	9	North America	United States	Enteritidis	Enteritidis	1425	19851
SAL_DA2210AA	WAPHL_SAL-A00636	SRR1615086	2014	9	North America	United States	Enteritidis	Enteritidis	1425	19850
SAL_DA2212AA	WAPHL_SAL-A00639	SRR1615084	2014	9	North America	United States	Enteritidis	Enteritidis	1425	19848
SAL_DA2213AA	WAPHL_SAL-A00631	SRR3945751	2014	8	North America	United States	Enteritidis	Enteritidis	1425	19847
SAL_DA2221AA	WAPHL_SAL-A00634	SRR1615075	2014	8	North America	United States	Enteritidis	Enteritidis	1425	19839
SAL_DA2224AA	WAPHL_SAL-A00635	SRR1615000	2014	9	North America	United States	Enteritidis	Enteritidis	1425	18379
SAL_DA2225AA	NY-swgs1460	SRR1614999	2014	8	North America	United States	Enteritidis	Enteritidis	1425	19038
SAL_DA2317AA	NY-swgs1499	SRR1612367	2014	9	North America	United States	Enteritidis	Enteritidis	1425	18599
SAL_DA2319AA	NY-swgs1501	SRR1612365	2014	9	North America	United States	Enteritidis	Enteritidis	1425	19759
SAL_DA2320AA	NY-swgs1500	SRR1612364	2014	9	North America	United States	Enteritidis	Enteritidis	8003	19758
SAL_DA2339AA	MDH-2014-00976	SRR1612334	2014	9	North America	United States	Enteritidis	Enteritidis	1425	19741
SAL_DA2340AA	NY-swgs1495	SRR1612333	2014	8	North America	United States	Enteritidis	Enteritidis	1425	2668
SAL_DA2345AA	MDH-2014-00977	SRR1612326	2014	9	North America	United States	Enteritidis	Enteritidis	1425	19737
SAL_DA2350AA	NY-swgs1498	SRR1612316	2014	9	North America	United States	Enteritidis	Enteritidis	1425	19732
SAL_DA2357AA	NY-swgs1497	SRR1612304	2014	9	North America	United States	Enteritidis	Enteritidis	1425	19726
SAL_DA2361AA	NY-swgs1496	SRR1612292	2014	9	North America	United States	Enteritidis	Enteritidis	1425	19722
SAL_DA2381AA	AZ-TG74752	SRR1609683	2005		North America	United States	Enteritidis	Enteritidis	1425	19706
SAL_DA2394AA	FDAARGOS_70	SRR1608974	2013	7	North America	United States	Group D	Enteritidis	1425	19694
SAL_DA2441AA	MDH-2014-00975	SRR1598969	2014	9	North America	United States	Enteritidis	Enteritidis	1425	18563
SAL_DA2443AA	MDH-2014-00973	SRR1598968	2014	9	North America	United States	Enteritidis	Enteritidis	1425	19657
SAL_DA2447AA	MDH-2014-00972	SRR1598964	2014	9	North America	United States	Enteritidis	Enteritidis	1425	18563
SAL_DA2453AA	MDH-2014-00974	SRR1598957	2014	9	North America	United States	Enteritidis	Enteritidis	1425	19649
SAL_DA2480AA	NY-swgs1478	SRR1596415	2014	9	North America	United States	Enteritidis	Enteritidis	1425	18599
SAL_DA2490AA	NY-swgs1485	SRR1596404	2014	9	North America	United States	Enteritidis	Enteritidis	1425	19620
SAL_DA2496AA	NY-swgs1481	SRR1596397	2014	9	North America	United States	Enteritidis	Enteritidis	1425	19182
SAL_DA2497AA	NY-swgs1477	SRR1596396	2014	8	North America	United States	Enteritidis	Enteritidis	1425	19616
SAL_DA2503AA	NY-swgs1484	SRR1596390	2014	9	North America	United States	Enteritidis	Enteritidis	1425	19612
SAL_DA2504AA	NY-swgs1479	SRR1596389	2014	8	North America	United States	Enteritidis	Enteritidis	1425	19611
SAL_DA2506AA	FDN0159	SRR1596387	2013	5	North America	United States	Enteritidis (Predicted)	Enteritidis	1425	19609
SAL_DA2509AA	NY-swgs1482	SRR1596384	2014	9	North America	United States	Enteritidis	Enteritidis	1425	19607
SAL_DA2510AA	NY-swgs1480	SRR1596383	2014	9	North America	United States	Enteritidis	Enteritidis	1425	19606
SAL_DA2518AA	NY-swgs1483	SRR1596374	2014	9	North America	United States	Enteritidis	Enteritidis	1425	19600
SAL_DA2553AA	FDA00003855	SRR1593413	2011	3	Asia	Bangladesh	Enteritidis	Enteritidis	1425	19559
SAL_DA2561AA	MDH-2014-00971	SRR1593405	2014	9	North America	United States	Enteritidis	Enteritidis	1425	19569
SAL_DA2562AA	MDH-2014-00970	SRR1593404	2014	9	North America	United States	Enteritidis	Enteritidis	1425	19568
SAL_DA2574AA	FDA00003854	SRR1593392	2011	3	Asia	Bangladesh	Enteritidis	Enteritidis	1425	19559
SAL_DA2628AA	NY-swgs1472	SRR1588691	2014	9	North America	United States	Enteritidis	Enteritidis	1425	19517
SAL_DA2630AA	NY-swgs1473	SRR1588689	2014	9	North America	United States	Enteritidis	Enteritidis	8003	12492
SAL_DA2635AA	NY-swgs1476	SRR1588684	2014	8	North America	United States	Enteritidis	Enteritidis	1425	19512
SAL_DA2636AA	NY-swgs1474	SRR1588683	2014	9	North America	United States	Enteritidis	Enteritidis	1425	18599
SAL_DA2637AA	NY-swgs1468	SRR1588682	2014	8	North America	United States	Enteritidis	Enteritidis	1425	17266
SAL_DA2642AA	NY-swgs1475	SRR1588677	2014	9	North America	United States	Enteritidis	Enteritidis	1425	19507
SAL_DA2649AA	NY-swgs1471	SRR1588670	2014	9	North America	United States	Enteritidis	Enteritidis	1425	19182
SAL_DA2650AA	NY-swgs1470	SRR1588669	2014	8	North America	United States	Enteritidis	Enteritidis	1425	18599
SAL_DA2651AA	NY-swgs1469	SRR1588668	2014	8	North America	United States	Enteritidis	Enteritidis	1425	17266
SAL_DA2652AA	NY-swgs1467	SRR1588667	2014	8	North America	United States	Enteritidis	Enteritidis	1425	18599
SAL_DA2659AA	FDA00004351	SRR1586612	2014	4	Europe	Lithuania	Enteritidis (Predicted)	Enteritidis	1425	19497
SAL_DA2691AA	MDH-2014-00969	SRR1586580	2014	9	North America	United States	Enteritidis	Enteritidis	8003	19480
SAL_DA2720AA	MDH-2014-00968	SRR1586550	2014	9	North America	United States	Enteritidis	Enteritidis	1425	18290
SAL_DA2854AA	NY-swgs1452	SRR1582196	2014	8	North America	United States	Enteritidis	Enteritidis	27796	19374
SAL_DA2855AA	NY-swgs1397	SRR1582195	2014	7	North America	United States	Enteritidis	Enteritidis	1425	19373
SAL_DA2857AA	NY-swgs1462	SRR1582193	2014	8	North America	United States	Enteritidis	Enteritidis	1425	18599
SAL_DA2860AA	NY-swgs1456	SRR1582189	2014	8	North America	United States	Enteritidis	Enteritidis	1425	19369
SAL_DA2863AA	NY-swgs1398	SRR1582186	2014	7	North America	United States	Enteritidis	Enteritidis	1425	19367
SAL_DA2864AA	NY-swgs1392	SRR1582185	2014	7	North America	United States	Enteritidis	Enteritidis	1425	19366

SAL_DA2868AA	NY-swgs1453	SRR1582181	2014	8	North America	United States	Enteritidis	Enteritidis	1425	18599
SAL_DA2870AA	NY-swgs1454	SRR1582179	2014	8	North America	United States	Enteritidis	Enteritidis	1425	19361
SAL_DA2871AA	NY-swgs1393	SRR1582178	2014	7	North America	United States	Enteritidis	Enteritidis	1425	19360
SAL_DA2874AA	NY-swgs1394	SRR1582175	2014	7	North America	United States	Enteritidis	Enteritidis	1425	19358
SAL_DA2876AA	NY-swgs1390	SRR1582172	2014	7	North America	United States	Enteritidis	Enteritidis	1425	19356
SAL_DA2877AA	NY-swgs1389	SRR1582171	2014	7	North America	United States	Enteritidis	Enteritidis	1425	19018
SAL_DA2881AA	NY-swgs1455	SRR1582151	2014	8	North America	United States	Enteritidis	Enteritidis	1425	18599
SAL_DA2882AA	NY-swgs1395	SRR1582150	2014	7	North America	United States	Enteritidis	Enteritidis	1425	19353
SAL_DA2887AA	NY-swgs1463	SRR1582144	2014	8	North America	United States	Enteritidis	Enteritidis	1425	18380
SAL_DA2903AA	NY-swgs1461	SRR1581508	2014	8	North America	United States	Enteritidis	Enteritidis	1425	19339
SAL_DA2904AA	NY-swgs1396	SRR1581507	2014	7	North America	United States	Enteritidis	Enteritidis	1425	19338
SAL_DA2913AA	NY-swgs1391	SRR1581498	2014	7	North America	United States	Enteritidis	Enteritidis	1425	7686
SAL_DA2986AA	WAPHL_SAL-A00620	SRR1576611	2014	7	North America	United States	Enteritidis	Enteritidis	1425	19284
SAL_DA2988AA	WAPHL_SAL-A00616	SRR1576609	2014	5	North America	United States	Enteritidis	Enteritidis	1425	19281
SAL_DA3016AA	MDH-2014-00414	SRR1576563	2003	3	North America	United States	Enteritidis	Enteritidis	26543	19263
SAL_DA3017AA	MDH-2014-00413	SRR1576562	2003	3	North America	United States	Enteritidis	Enteritidis	26543	19262
SAL_DA3035AA	WAPHL_SAL-A00624	SRR1576244	2014	7	North America	United States	Enteritidis	Enteritidis	1425	19248
SAL_DA3067AA	WAPHL_SAL-A00615	SRR1576209	2014	5	North America	United States	Enteritidis	Enteritidis	1425	19187
SAL_DA3121AA	WAPHL_SAL-A00614	SRR1576101	2014	5	North America	United States	Enteritidis	Enteritidis	1425	19187
SAL_DA3126AA	WAPHL_SAL-A00613	SRR1576095	2014	5	North America	United States	Enteritidis	Enteritidis	1425	19187
SAL_DA3133AA	WAPHL_SAL-A00622	SRR1576023	2014	7	North America	United States	Enteritidis	Enteritidis	1425	19182
SAL_DA3135AA	WAPHL_SAL-A00628	SRR1576021	2014	7	North America	United States	Enteritidis	Enteritidis	1425	19180
SAL_DA3140AA	WAPHL_SAL-A00626	SRR1576016	2014	7	North America	United States	Enteritidis	Enteritidis	1425	19175
SAL_DA3141AA	WAPHL_SAL-A00625	SRR1576015	2014	7	North America	United States	Enteritidis	Enteritidis	1425	19174
SAL_DA3144AA	WAPHL_SAL-A00621	SRR1576011	2014	7	North America	United States	Enteritidis	Enteritidis	1425	19171
SAL_DA3145AA	WAPHL_SAL-A00618	SRR1576010	2014	7	North America	United States	Enteritidis	Enteritidis	1425	19170
SAL_DA3150AA	CFSAN023296	SRR1576005	2001	5	North America	United States	Enteritidis	Enteritidis	1425	14275
SAL_DA3151AA	CFSAN023294	SRR1576004	2001	5	North America	United States	Enteritidis	Enteritidis	1425	14275
SAL_DA3160AA	CFSAN023290	SRR1575991	2001	5	North America	United States	Enteritidis	Enteritidis	1425	19159
SAL_DA3161AA	WAPHL_SAL-A00623	SRR1575989	2014	7	North America	United States	Enteritidis	Enteritidis	1425	19158
SAL_DA3180AA	FDA00003513	SRR1575959	2011	10	North America	United States	Enteritidis	Enteritidis	1425	19143
SAL_DA3184AA	CFSAN023300	SRR1575955	2001	7	North America	United States	Enteritidis	Enteritidis	1425	14261
SAL_DA3196AA	WAPHL_SAL-A00627	SRR1575942	2014	8	North America	United States	Enteritidis	Enteritidis	1425	19134
SAL_DA3197AA	CFSAN023677	SRR1575941	2006	4	North America	United States	Enteritidis (Predicted)	Enteritidis	26543	19133
SAL_DA3200AA	WAPHL_SAL-A00619	SRR1575911	2014	7	North America	United States	Enteritidis	Enteritidis	1425	15185
SAL_DA3205AA	CFSAN023311	SRR1575906	2003	11	North America	United States	Enteritidis	Enteritidis	1425	19128
SAL_DA3208AA	MDH-2014-00944	SRR1574317	2014	8	North America	United States	Enteritidis	Enteritidis	1425	19125
SAL_DA3222AA	MDH-2014-00496	SRR1574303	2003	5	North America	United States	Enteritidis	Enteritidis	27433	19113
SAL_DA3227AA	NY-swgs1449	SRR1574298	2014	8	North America	United States	Enteritidis	Enteritidis	7801	19108
SAL_DA3228AA	NY-swgs1448	SRR1574297	2014	8	North America	United States	Enteritidis	Enteritidis	1425	19107
SAL_DA3235AA	MDH-2014-00965	SRR1574287	2014	8	North America	United States	Enteritidis	Enteritidis	1425	19101
SAL_DA3236AA	MDH-2014-00945	SRR1574286	2014	8	North America	United States	Enteritidis	Enteritidis	1425	19100
SAL_DA3237AA	NY-swgs1445	SRR1574285	2014	8	North America	United States	Enteritidis	Enteritidis	1425	19099
SAL_DA3246AA	NY-swgs1447	SRR1574276	2014	8	North America	United States	Enteritidis	Enteritidis	1425	17155
SAL_DA3247AA	NY-swgs1446	SRR1574275	2014	8	North America	United States	Enteritidis	Enteritidis	1425	16241
SAL_DA3248AA	NY-swgs1450	SRR1574267	2014	8	North America	United States	Enteritidis	Enteritidis	1425	19038
SAL_DA3255AA	MDH-2014-00943	SRR1574258	2014	8	North America	United States	Enteritidis	Enteritidis	1425	18563
SAL_DA3262AA	MDH-2014-00946	SRR1574250	2014	8	North America	United States	Enteritidis	Enteritidis	1425	19081
SAL_DA3263AA	NY-swgs1451	SRR1574249	2014	8	North America	United States	Enteritidis	Enteritidis	1425	19043
SAL_DA3264AA	NY-swgs1444	SRR1574248	2014	8	North America	United States	Enteritidis	Enteritidis	1425	18599
SAL_DA3289AA	WAPHL_SAL-A00540	SRR1573562	2004		North America	United States	Enteritidis	Enteritidis	1425	19061
SAL_DA3291AA	NY-swgs1431	SRR1573189	2014	8	North America	United States	Enteritidis	Enteritidis	1425	19059
SAL_DA3292AA	NY-swgs1428	SRR1573188	2014	8	North America	United States	Enteritidis	Enteritidis	8003	12492
SAL_DA3295AA	NY-swgs1429	SRR1573185	2014	8	North America	United States	Enteritidis	Enteritidis	1425	19057
SAL_DA3296AA	NY-swgs1426	SRR1573184	2014	8	North America	United States	Enteritidis	Enteritidis	1425	19056
SAL_DA3300AA	NY-swgs1417	SRR1573180	2014	8	North America	United States	Enteritidis	Enteritidis	1425	19022
SAL_DA3301AA	NY-swgs1433	SRR1573179	2014	8	North America	United States	Enteritidis	Enteritidis	1425	19053
SAL_DA3302AA	NY-swgs1421	SRR1573178	2014	8	North America	United States	Enteritidis	Enteritidis	1425	19052
SAL_DA3303AA	NY-swgs1420	SRR1573177	2014	8	North America	United States	Enteritidis	Enteritidis	1425	19051

SAL_DA3305AA	CFSAN023306	SRR1573175	2003	11	North America	United States	Enteritidis	Enteritidis	1425	19049
SAL_DA3309AA	CFSAN023287	SRR1573171	2000	9	North America	United States	Enteritidis	Enteritidis	1425	14269
SAL_DA3310AA	NY-swgs1427	SRR1573137	2014	8	North America	United States	Enteritidis	Enteritidis	1425	19045
SAL_DA3312AA	CFSAN023301	SRR1573135	2001	7	North America	United States	Enteritidis	Enteritidis	1425	14261
SAL_DA3313AA	CFSAN023299	SRR1573134	2001	6	North America	United States	Enteritidis	Enteritidis	1425	19044
SAL_DA3314AA	NY-swgs1424	SRR1573132	2014	8	North America	United States	Enteritidis	Enteritidis	1425	19037
SAL_DA3315AA	CFSAN023616	SRR1573131	2007	2	North America	United States	Enteritidis (Predicted)	Enteritidis	1425	13508
SAL_DA3317AA	NY-swgs1419	SRR1573129	2014	8	North America	United States	Enteritidis	Enteritidis	1425	19043
SAL_DA3318AA	NY-swgs1418	SRR1573128	2014	8	North America	United States	Enteritidis	Enteritidis	1425	19042
SAL_DA3321AA	CFSAN023317	SRR1573125	2011	10	North America	United States	Enteritidis	Enteritidis	1425	19040
SAL_DA3322AA	NY-swgs1432	SRR1573124	2014	8	North America	United States	Enteritidis	Enteritidis	1425	19039
SAL_DA3323AA	NY-swgs1430	SRR1573123	2014	8	North America	United States	Enteritidis	Enteritidis	1425	19038
SAL_DA3324AA	NY-swgs1425	SRR1573122	2014	8	North America	United States	Enteritidis	Enteritidis	1425	19037
SAL_DA3327AA	CFSAN023314	SRR1573119	2003	11	North America	United States	Enteritidis	Enteritidis	1425	19036
SAL_DA3337AA	NY-swgs1423	SRR1573097	2014	8	North America	United States	Enteritidis	Enteritidis	1425	19029
SAL_DA3340AA	CFSAN023304	SRR1573094	2000	8	North America	United States	Enteritidis	Enteritidis	1425	19027
SAL_DA3341AA	NY-swgs1422	SRR1573093	2014	8	North America	United States	Enteritidis	Enteritidis	1425	19026
SAL_DA3345AA	NY-swgs1387	SRR1571600	2014	7	North America	United States	Enteritidis	Enteritidis	8003	19024
SAL_DA3346AA	NY-swgs1381	SRR1571599	2014	7	North America	United States	Enteritidis	Enteritidis	1425	19023
SAL_DA3347AA	NY-swgs1386	SRR1571598	2014	7	North America	United States	Enteritidis	Enteritidis	1425	19022
SAL_DA3348AA	NY-swgs1380	SRR1571597	2014	7	North America	United States	Enteritidis	Enteritidis	1425	19021
SAL_DA3349AA	NY-swgs1388	SRR1571594	2014	7	North America	United States	Enteritidis	Enteritidis	1425	19020
SAL_DA3350AA	NY-swgs1385	SRR1571590	2014	7	North America	United States	Enteritidis	Enteritidis	1425	19019
SAL_DA3351AA	NY-swgs1384	SRR1571586	2014	7	North America	United States	Enteritidis	Enteritidis	1425	19018
SAL_DA3352AA	NY-swgs1383	SRR1571582	2014	7	North America	United States	Enteritidis	Enteritidis	1425	19017
SAL_DA3353AA	NY-swgs1382	SRR1571579	2014	7	North America	United States	Enteritidis	Enteritidis	1425	19017
SAL_DA3425AA	MDH-2014-00940	SRR1569753	2014	8	North America	United States	Enteritidis	Enteritidis	1425	18950
SAL_DA3430AA	CFSAN023319	SRR1569748	2011	9	North America	United States	Enteritidis	Enteritidis	28313	18946
SAL_DA3442AA	CFSAN023285	SRR1569730	2000	9	North America	United States	Enteritidis	Enteritidis	1425	14269
SAL_DA3448AA	MDH-2014-00941	SRR1569723	2014	8	North America	United States	Enteritidis	Enteritidis	1425	18563
SAL_DA3452AA	CFSAN023310	SRR1569719	2003	11	North America	United States	Enteritidis	Enteritidis	1425	14267
SAL_DA3453AA	CFSAN023308	SRR1569718	2003	11	North America	United States	Enteritidis	Enteritidis	1425	14267
SAL_DA3458AA	CFSAN023291	SRR1569712	2001	5	North America	United States	Enteritidis	Enteritidis	1425	18926
SAL_DA3459AA	CFSAN023289	SRR1569711	2000	10	North America	United States	Enteritidis	Enteritidis	1425	18925
SAL_DA3477AA	MDH-2014-00942	SRR1569690	2014	8	North America	United States	Enteritidis	Enteritidis	1425	18912
SAL_DA3479AA	CFSAN023284	SRR1569688	2000	9	North America	United States	Enteritidis	Enteritidis	1425	14269
SAL_DA3484AA	CFSAN023292	SRR1569683	2001	5	North America	United States	Enteritidis	Enteritidis	1425	14275
SAL_DA3487AA	CFSAN023295	SRR1569679	2001	5	North America	United States	Enteritidis	Enteritidis	1425	14275
SAL_DA3495AA	CFSAN023288	SRR1569670	2000	10	North America	United States	Enteritidis	Enteritidis	1425	14269
SAL_DA3502AA	CFSAN023307	SRR1569663	2003	11	North America	United States	Enteritidis	Enteritidis	1425	14267
SAL_DA3503AA	CFSAN023297	SRR1569662	2001	5	North America	United States	Enteritidis	Enteritidis	1425	14275
SAL_DA3504AA	CFSAN023283	SRR1569661	2000	9	North America	United States	Enteritidis	Enteritidis	1425	18898
SAL_DA3509AA	CFSAN023303	SRR1569655	2001	7	North America	United States	Enteritidis	Enteritidis	1425	18895
SAL_DA3510AA	CFSAN023286	SRR1569654	2000	9	North America	United States	Enteritidis	Enteritidis	1425	14269
SAL_DA3513AA	CFSAN023293	SRR1569650	2001	5	North America	United States	Enteritidis	Enteritidis	1425	18893
SAL_DA3518AA	CFSAN023298	SRR1569645	2001	4	North America	United States	Enteritidis	Enteritidis	1425	18889
SAL_DA3527AA	CFSAN023309	SRR1569635	2003	11	North America	United States	Enteritidis	Enteritidis	1425	18883
SAL_DA3533AA	CFSAN023318	SRR1569623	2011	9	North America	United States	Enteritidis	Enteritidis	1425	18878
SAL_DA3534AA	MDH-2014-00939	SRR1569622	2014	8	North America	United States	Enteritidis	Enteritidis	1425	18877
SAL_DA3537AA	CFSAN023313	SRR1569619	2003	8	North America	United States	Enteritidis	Enteritidis	1425	18875
SAL_DA3543AA	CFSAN023302	SRR1569607	2001	7	North America	United States	Enteritidis	Enteritidis	1425	18869
SAL_DA3546AA	CFSAN023305	SRR1569604	2000	11	North America	United States	Enteritidis	Enteritidis	1425	14304
SAL_DA3551AA	CFSAN023315	SRR1569599	2011	9	North America	United States	Enteritidis	Enteritidis	1425	18864
SAL_DA3559AA	CFSAN023312	SRR1569590	2003	12	North America	United States	Enteritidis	Enteritidis	1425	14267
SAL_DA3562AA	CFSAN023316	SRR1569587	2011	10	North America	United States	Enteritidis	Enteritidis	1425	14281
SAL_DA3566AA	CFSAN023236	SRR1567879	2006	11	North America	United States	Enteritidis (Predicted)	Enteritidis	1425	18852
SAL_DA3567AA	CFSAN023235	SRR1567878	2006	11	North America	United States	Enteritidis (Predicted)	Enteritidis	1425	18851
SAL_DA3569AA	CFSAN023237	SRR1567875	2006	11	North America	United States	Enteritidis (Predicted)	Enteritidis	1425	18849
SAL_DA3574AA	CFSAN023239	SRR1567847	2006	11	North America	United States	Enteritidis (Predicted)	Enteritidis	1425	18845

SAL_DA3779AA	NY-swgs1416	SRR1566240	2014	8	North America	United States	Enteritidis	Enteritidis	1425	18705
SAL_DA3780AA	NY-swgs1415	SRR1566238	2014	8	North America	United States	Enteritidis	Enteritidis	1425	18704
SAL_DA3781AA	NY-swgs1414	SRR1566237	2014	8	North America	United States	Enteritidis	Enteritidis	8003	18703
SAL_DA3782AA	NY-swgs1413	SRR1566234	2014	8	North America	United States	Enteritidis	Enteritidis	1425	18702
SAL_DA3783AA	NY-swgs1412	SRR1566233	2014	8	North America	United States	Enteritidis	Enteritidis	1425	18694
SAL_DA3791AA	CFSAN023258	SRR1566223	2006	11	North America	United States	Enteritidis (Predicted)	Enteritidis	26543	18687
SAL_DA3792AA	CFSAN023257	SRR1566222	2006	11	North America	United States	Enteritidis (Predicted)	Enteritidis	26543	18686
SAL_DA3793AA	CFSAN023256	SRR1566221	2006	11	North America	United States	Enteritidis (Predicted)	Enteritidis	26543	18685
SAL_DA3794AA	CFSAN023255	SRR1566220	2006	11	North America	United States	Enteritidis (Predicted)	Enteritidis	26543	18684
SAL_DA3795AA	CFSAN023254	SRR1566219	2006	11	North America	United States	Enteritidis (Predicted)	Enteritidis	26543	18683
SAL_DA3796AA	CFSAN023253	SRR1566218	2006	11	North America	United States	Enteritidis (Predicted)	Enteritidis	1425	18682
SAL_DA3797AA	CFSAN023252	SRR1566217	2006	11	North America	United States	Enteritidis (Predicted)	Enteritidis	1425	18681
SAL_DA3798AA	CFSAN023251	SRR1566216	2006	11	North America	United States	Enteritidis (Predicted)	Enteritidis	1425	18680
SAL_DA3799AA	CFSAN023250	SRR1566215	2006	11	North America	United States	Enteritidis (Predicted)	Enteritidis	28296	18679
SAL_DA3800AA	CFSAN023249	SRR1566214	2006	11	North America	United States	Enteritidis (Predicted)	Enteritidis	26543	18678
SAL_DA3801AA	CFSAN023248	SRR1566213	2006	11	North America	United States	Enteritidis (Predicted)	Enteritidis	1425	18677
SAL_DA3802AA	CFSAN023247	SRR1566212	2006	11	North America	United States	Enteritidis (Predicted)	Enteritidis	1425	18676
SAL_DA3803AA	CFSAN023246	SRR1566211	2006	11	North America	United States	Enteritidis (Predicted)	Enteritidis	1425	18675
SAL_DA3804AA	CFSAN023245	SRR1566210	2006	11	North America	United States	Enteritidis (Predicted)	Enteritidis	28295	18674
SAL_DA3805AA	CFSAN023244	SRR1566209	2006	11	North America	United States	Enteritidis (Predicted)	Enteritidis	28295	18673
SAL_DA3806AA	CFSAN023243	SRR1566208	2006	11	North America	United States	Enteritidis (Predicted)	Enteritidis	26543	18672
SAL_DA3807AA	CFSAN023242	SRR1566207	2006	11	North America	United States	Enteritidis (Predicted)	Enteritidis	26543	18671
SAL_DA3808AA	CFSAN023241	SRR1566206	2006	11	North America	United States	Enteritidis (Predicted)	Enteritidis	1425	18670
SAL_DA3814AA	CFSAN023238	SRR1564497	2006	11	North America	United States	Enteritidis (Predicted)	Enteritidis	1425	18667
SAL_DA3820AA	MDH-2014-00937	SRR1562664	2014	8	North America	United States	Enteritidis	Enteritidis	27436	18662
SAL_DA3822AA	FDA00004276	SRR1562662	2014	1	Asia	Bangladesh	Enteritidis	Enteritidis	1425	18660
SAL_DA3827AA	MDH-2014-00934	SRR1562657	2014	8	North America	United States	Enteritidis	Enteritidis	1425	18655
SAL_DA3833AA	MDH-2014-00938	SRR1562651	2014	8	North America	United States	Enteritidis	Enteritidis	1425	18563
SAL_DA3834AA	NY-swgs1407	SRR1562650	2014	8	North America	United States	Enteritidis	Enteritidis	1425	18651
SAL_DA3835AA	NY-swgs1404	SRR1562649	2014	7	North America	United States	Enteritidis	Enteritidis	3888	18650
SAL_DA3840AA	NY-swgs1411	SRR1562644	2014	8	North America	United States	Enteritidis	Enteritidis	1425	18646
SAL_DA3841AA	NY-swgs1410	SRR1562643	2014	8	North America	United States	Enteritidis	Enteritidis	1425	18645
SAL_DA3842AA	NY-swgs1401	SRR1562642	2014	7	North America	United States	Enteritidis	Enteritidis	1425	18644
SAL_DA3847AA	NY-swgs1402	SRR1562636	2014	7	North America	United States	Enteritidis	Enteritidis	1425	18640
SAL_DA3848AA	NY-swgs1399	SRR1562635	2014	8	North America	United States	Enteritidis	Enteritidis	1425	18639
SAL_DA3858AA	NY-swgs1409	SRR1562625	2014	8	North America	United States	Enteritidis	Enteritidis	1425	18630
SAL_DA3866AA	MDH-2014-00935	SRR1562612	2014	8	North America	United States	Enteritidis	Enteritidis	1425	17514
SAL_DA3867AA	NY-swgs1406	SRR1562611	2014	8	North America	United States	Enteritidis	Enteritidis	8003	18623
SAL_DA3872AA	NY-swgs1400	SRR1562604	2014	7	North America	United States	Enteritidis	Enteritidis	1425	18618
SAL_DA3879AA	NY-swgs1403	SRR1562597	2014	8	North America	United States	Enteritidis	Enteritidis	1425	18611
SAL_DA3893AA	MDH-2014-00936	SRR1562583	2014	8	North America	United States	Enteritidis	Enteritidis	1425	18600
SAL_DA3894AA	NY-swgs1408	SRR1562582	2014	8	North America	United States	Enteritidis	Enteritidis	1425	18599
SAL_DA3899AA	NY-swgs1405	SRR1562568	2014	7	North America	United States	Enteritidis	Enteritidis	1425	18594
SAL_DA3906AA	MDH-2014-00930	SRR1561716	2014	8	North America	United States	Enteritidis	Enteritidis	1425	18563
SAL_DA3933AA	MDH-2014-00933	SRR1561689	2014	8	North America	United States	Enteritidis	Enteritidis	1425	18563
SAL_DA3934AA	MDH-2014-00932	SRR1561688	2014	8	North America	United States	Enteritidis	Enteritidis	1425	18561
SAL_DA3935AA	MDH-2014-00931	SRR1561687	2014	8	North America	United States	Enteritidis	Enteritidis	1425	18562
SAL_DA3936AA	MDH-2014-00929	SRR1561686	2014	8	North America	United States	Enteritidis	Enteritidis	1425	18561
SAL_DA4007AA	MDH-2014-00910	SRR1557008	2014	8	North America	United States	Enteritidis	Enteritidis	1425	18498
SAL_DA4012AA	MDH-2014-00908	SRR1557001	2014	8	North America	United States	Enteritidis	Enteritidis	1425	18493
SAL_DA4024AA	MDH-2014-00912	SRR1556985	2014	8	North America	United States	Enteritidis	Enteritidis	1425	18369
SAL_DA4035AA	MDH-2014-00909	SRR1556970	2014	8	North America	United States	Enteritidis	Enteritidis	1425	18478
SAL_DA4047AA	MDH-2014-00911	SRR1556957	2014	8	North America	United States	Enteritidis	Enteritidis	1425	18469
SAL_DA4090AA	WAPHL_SAL-A00505	SRR1556074	2004		North America	United States	Enteritidis	Enteritidis	1425	18432
SAL_DA4142AA	NY-swgs1373	SRR1553898	2014	7	North America	United States	Enteritidis	Enteritidis	1425	18389
SAL_DA4145AA	FCC0179	SRR1553895	2010	9	North America	United States	Enteritidis	Enteritidis	1425	18387
SAL_DA4147AA	NY-swgs1308	SRR1544416	2014	5	North America	United States	Enteritidis	Enteritidis	1425	18385
SAL_DA4151AA	FCC0181	SRR1553883	2010	9	North America	United States	Enteritidis	Enteritidis	1425	41056
SAL_DA4153AA	NY-swgs1379	SRR1553878	2014	7	North America	United States	Enteritidis	Enteritidis	1425	18380

SAL_DA4154AA	NY-swgs1378	SRR1553877	2014	7	North America	United States	Enteritidis	Enteritidis	1425	18379
SAL_DA4156AA	FCC0175	SRR1553873	2010	9	North America	United States	Enteritidis	Enteritidis	1425	18377
SAL_DA4160AA	NY-swgs1377	SRR1553865	2014	7	North America	United States	Enteritidis	Enteritidis	1425	18373
SAL_DA4161AA	NY-swgs1371	SRR1553864	2014	7	North America	United States	Enteritidis	Enteritidis	1425	18372
SAL_DA4164AA	MDH-2014-00907	SRR1553860	2014	8	North America	United States	Enteritidis	Enteritidis	1425	18369
SAL_DA4169AA	NY-swgs1370	SRR1553848	2014	7	North America	United States	Enteritidis	Enteritidis	1425	18364
SAL_DA4172AA	NY-swgs1375	SRR1553845	2014	7	North America	United States	Enteritidis	Enteritidis	8003	18361
SAL_DA4181AA	NY-swgs1372	SRR1553833	2014	7	North America	United States	Enteritidis	Enteritidis	1425	18353
SAL_DA4194AA	NY-swgs1376	SRR1553814	2014	7	North America	United States	Enteritidis	Enteritidis	1425	18341
SAL_DA4201AA	FCC0182	SRR1553805	2010	10	North America	United States	Enteritidis	Enteritidis	1425	18334
SAL_DA4208AA	NY-swgs1369	SRR1553794	2014	7	North America	United States	Enteritidis	Enteritidis	1425	18327
SAL_DA4228AA	MDH-2014-00905	SRR1553763	2014	8	North America	United States	Enteritidis	Enteritidis	1425	12315
SAL_DA4230AA	MDH-2014-00904	SRR1553761	2014	8	North America	United States	Enteritidis	Enteritidis	1425	15673
SAL_DA4232AA	NY-swgs1374	SRR1553759	2014	7	North America	United States	Enteritidis	Enteritidis	1425	18308
SAL_DA4238AA	FCC0177	SRR1553751	2010	9	North America	United States	Enteritidis	Enteritidis	1425	18302
SAL_DA4244AA	FCC0174	SRR1553743	2010	9	North America	United States	Enteritidis	Enteritidis	1425	18297
SAL_DA4245AA	FCC0173	SRR1553742	2010	9	North America	United States	Enteritidis	Enteritidis	1425	18296
SAL_DA4248AA	NY-swgs1302	SRR1553735	2014	5	North America	United States	Enteritidis	Enteritidis	1425	18293
SAL_DA4251AA	MDH-2014-00906	SRR1553731	2014	8	North America	United States	Enteritidis	Enteritidis	1425	18290
SAL_DA4254AA	NY-swgs1309	SRR1544146	2014	5	North America	United States	Enteritidis	Enteritidis	1425	18287
SAL_DA4352AA	MDH-2014-00901	SRR1544396	2014	7	North America	United States	Enteritidis	Enteritidis	7801	18211
SAL_DA4399AA	swgs1199	SRR1544333	2014	1	North America	United States	Enteritidis	Enteritidis	1425	18176
SAL_DA4440AA	swgs1202	SRR1220758	2014	1	North America	United States	Enteritidis	Enteritidis	1425	18141
SAL_DA4716AA	P184	ERR025165					Enteritidis (Predicted)	Enteritidis	1425	17948
SAL_DA4717AA	I21	ERR025166					Enteritidis (Predicted)	Enteritidis	1425	17947
SAL_DA4723AA	I99	ERR025172					Enteritidis (Predicted)	Enteritidis	1425	17941
SAL_DA4725AA	P43	ERR025174					Enteritidis (Predicted)	Enteritidis	1425	17940
SAL_DA4728AA	LIV43439	ERR024947					Enteritidis (Predicted)	Enteritidis	-1714	17937
SAL_DA4730AA	U952	ERR024949					Enteritidis (Predicted)	Enteritidis	1424	17936
SAL_DA4843AA	M281758.7	ERR024419					Enteritidis (Predicted)	Enteritidis	1424	17834
SAL_DA4969AA	C4810	ERR023629					Enteritidis (Predicted)	Enteritidis	1424	17731
SAL_DA5026AA	MDH-2014-00898	SRR1544254	2014	7	North America	United States	Enteritidis	Enteritidis	1425	17689
SAL_DA5061AA	MDH-2014-00900	SRR1544215	2014	7	North America	United States	Enteritidis	Enteritidis	1425	17661
SAL_DA5080AA	MDH-2014-00897	SRR1544186	2014	7	North America	United States	Enteritidis	Enteritidis	1425	17646
SAL_DA5105AA	IEH-NGS-SAL-00898	SRR1544153	2007	1	North America	United States		Enteritidis	1425	17625
SAL_DA5157AA	MDH-2014-00896	SRR1539601	2014	7	North America	United States	Enteritidis	Enteritidis	1425	17577
SAL_DA5223AA	MDH-2014-00895	SRR1536172	2014	7	North America	United States	Enteritidis	Enteritidis	1425	17527
SAL_DA5224AA	MDH-2014-00885	SRR1536171	2014	7	North America	United States	Enteritidis	Enteritidis	1425	17526
SAL_DA5227AA	MDH-2014-00892	SRR1536168	2014	7	North America	United States	Enteritidis	Enteritidis	1425	17523
SAL_DA5228AA	MDH-2014-00889	SRR1536167	2014	7	North America	United States	Enteritidis	Enteritidis	1425	17522
SAL_DA5229AA	MDH-2014-00887	SRR1536166	2014	6	North America	United States	Enteritidis	Enteritidis	1425	17507
SAL_DA5234AA	MDH-2014-00893	SRR1536161	2014	7	North America	United States	Enteritidis	Enteritidis	1425	17517
SAL_DA5235AA	MDH-2014-00884	SRR1536160	2014	7	North America	United States	Enteritidis	Enteritidis	1425	17516
SAL_DA5238AA	MDH-2014-00883	SRR1536156	2014	7	North America	United States	Enteritidis	Enteritidis	1425	17515
SAL_DA5239AA	MDH-2014-00882	SRR1536155	2014	7	North America	United States	Enteritidis	Enteritidis	1425	17453
SAL_DA5240AA	MDH-2014-00894	SRR1536154	2014	7	North America	United States	Enteritidis	Enteritidis	1425	17514
SAL_DA5241AA	MDH-2014-00888	SRR1536153	2014	7	North America	United States	Enteritidis	Enteritidis	1425	17513
SAL_DA5246AA	MDH-2014-00890	SRR1536148	2014	7	North America	United States	Enteritidis	Enteritidis	27432	17508
SAL_DA5247AA	MDH-2014-00886	SRR1536147	2014	7	North America	United States	Enteritidis	Enteritidis	1425	17507
SAL_DA5251AA	MDH-2014-00891	SRR1536142	2014	7	North America	United States	Enteritidis	Enteritidis	1425	17503
SAL_DA5289AA	NY-swgs1368	SRR1534907	2014	7	North America	United States	Enteritidis	Enteritidis	1425	17457
SAL_DA5290AA	NY-swgs1365	SRR1534905	2014	6	North America	United States	Enteritidis	Enteritidis	1425	17456
SAL_DA5293AA	MDH-2014-00881	SRR1534902	2014	7	North America	United States	Enteritidis	Enteritidis	1425	17453
SAL_DA5294AA	NY-swgs1366	SRR1534900	2014	7	North America	United States	Enteritidis	Enteritidis	8003	12492
SAL_DA5295AA	MDH-2014-00855	SRR1534899	2014	7	North America	United States	Enteritidis	Enteritidis	1425	17452
SAL_DA5296AA	NY-swgs1367	SRR1534898	2014	7	North America	United States	Enteritidis	Enteritidis	1425	17451
SAL_DA5305AA	MDH-2014-00856	SRR1534887	2014	7	North America	United States	Enteritidis	Enteritidis	1425	17443
SAL_DA5308AA	MDH-2014-00879	SRR1534884	2014	7	North America	United States	Enteritidis	Enteritidis	1425	17440
SAL_DA5309AA	MDH-2014-00857	SRR1534883	2014	7	North America	United States	Enteritidis	Enteritidis	1425	17439

SAL_DA5316AA	MDH-2014-00852	SRR1534875	2014	7	North America	United States	Enteritidis	Enteritidis	27901	17435
SAL_DA5318AA	MDH-2014-00853	SRR1534868	2014	7	North America	United States	Enteritidis	Enteritidis	27901	17433
SAL_DA5323AA	MDH-2014-00850	SRR1534855	2014	7	North America	United States	Enteritidis	Enteritidis	1425	17428
SAL_DA5325AA	MDH-2014-00851	SRR1534851	2014	7	North America	United States	Enteritidis	Enteritidis	1425	4151
SAL_DA5327AA	MDH-2014-00880	SRR1534849	2014	7	North America	United States	Enteritidis	Enteritidis	1425	17425
SAL_DA5328AA	MDH-2014-00854	SRR1534847	2014	7	North America	United States	Enteritidis	Enteritidis	1425	17424
SAL_DA5338AA	MDH-2014-00878	SRR1534818	2014	7	North America	United States	Enteritidis	Enteritidis	1425	16878
SAL_DA5443AA	NY-swgs1364	SRR1525276	2014	6	North America	United States	Enteritidis	Enteritidis	1425	10271
SAL_DA5445AA	NY-swgs1359	SRR1525274	2014	6	North America	United States	Enteritidis	Enteritidis	1425	17325
SAL_DA5446AA	NY-swgs1358	SRR1525273	2014	6	North America	United States	Enteritidis	Enteritidis	1425	17324
SAL_DA5448AA	NY-swgs1360	SRR1525271	2014	6	North America	United States	Enteritidis	Enteritidis	8003	12492
SAL_DA5451AA	NY-swgs1361	SRR1525268	2014	6	North America	United States	Enteritidis	Enteritidis	1425	17321
SAL_DA5453AA	NY-swgs1362	SRR1525266	2014	6	North America	United States	Enteritidis	Enteritidis	1425	17319
SAL_DA5455AA	NY-swgs1363	SRR1525264	2014	6	North America	United States	Enteritidis	Enteritidis	1425	17317
SAL_DA5468AA	NY-swgs1349	SRR1521602	2014	6	North America	United States	Enteritidis	Enteritidis	3888	17304
SAL_DA5469AA	NY-swgs1351	SRR1521601	2014	6	North America	United States	Enteritidis	Enteritidis	1425	17303
SAL_DA5475AA	NY-swgs1355	SRR1521595	2014	6	North America	United States	Enteritidis	Enteritidis	8003	17299
SAL_DA5482AA	NY-swgs1352	SRR1521588	2014	6	North America	United States	Enteritidis	Enteritidis	1425	17293
SAL_DA5485AA	NY-swgs1353	SRR1521585	2014	6	North America	United States	Enteritidis	Enteritidis	1425	17290
SAL_DA5502AA	NY-swgs1357	SRR1521568	2014	6	North America	United States	Enteritidis	Enteritidis	1425	2067
SAL_DA5503AA	NY-swgs1346	SRR1521567	2014	6	North America	United States	Enteritidis	Enteritidis	1425	17277
SAL_DA5504AA	NY-swgs1347	SRR1521566	2014	6	North America	United States	Enteritidis	Enteritidis	1425	17155
SAL_DA5506AA	NY-swgs1348	SRR1521564	2014	6	North America	United States	Enteritidis	Enteritidis	1425	17275
SAL_DA5517AA	NY-swgs1354	SRR1521523	2014	6	North America	United States	Enteritidis	Enteritidis	1425	17266
SAL_DA5519AA	NY-swgs1356	SRR1521521	2014	6	North America	United States	Enteritidis	Enteritidis	1425	17264
SAL_DA5526AA	NY-swgs1350	SRR1521514	2014	6	North America	United States	Enteritidis	Enteritidis	1425	17257
SAL_DA5634AA	NY-swgs1345	SRR1514767	2014	6	North America	United States	Enteritidis	Enteritidis	1425	17166
SAL_DA5640AA	MDH-2014-00843	SRR1514760	2014	6	North America	United States	Enteritidis	Enteritidis	1425	17160
SAL_DA5643AA	MDH-2014-00849	SRR1514757	2014	7	North America	United States	Enteritidis	Enteritidis	1425	12265
SAL_DA5646AA	MDH-2014-00848	SRR1514753	2014	7	North America	United States	Enteritidis	Enteritidis	1425	17155
SAL_DA5658AA	MDH-2014-00847	SRR1514739	2014	7	North America	United States	Enteritidis	Enteritidis	1425	17148
SAL_DA5661AA	MDH-2014-00846	SRR1514736	2014	7	North America	United States	Enteritidis	Enteritidis	1425	17145
SAL_DA5663AA	NY-swgs1264	SRR1514734	2009	5	North America	United States	Enteritidis	Enteritidis	1425	17143
SAL_DA5667AA	NY-swgs1341	SRR1514730	2014	6	North America	United States	Enteritidis	Enteritidis	1425	17140
SAL_DA5670AA	NY-swgs1343	SRR1514727	2014	6	North America	United States	Enteritidis	Enteritidis	1425	17138
SAL_DA5678AA	NY-swgs1344	SRR1514718	2014	6	North America	United States	Enteritidis	Enteritidis	1425	17131
SAL_DA5681AA	FLDOH-50	SRR1514715	2014	4	North America	United States	Enteritidis	Enteritidis	1425	17128
SAL_DA5682AA	MDH-2014-00844	SRR1514714	2014	6	North America	United States	Enteritidis	Enteritidis	1425	17120
SAL_DA5686AA	FLDOH-49	SRR1514709	2014	4	North America	United States	Enteritidis	Enteritidis	1425	17124
SAL_DA5687AA	NY-swgs1342	SRR1514708	2014	6	North America	United States	Enteritidis	Enteritidis	1425	17123
SAL_DA5692AA	MDH-2014-00845	SRR1514703	2014	6	North America	United States	Enteritidis	Enteritidis	1425	17120
SAL_DA5715AA	NY-swgs1333	SRR1511583	2014	6	North America	United States	Enteritidis	Enteritidis	8003	17102
SAL_DA5718AA	NY-swgs1332	SRR1511580	2014	5	North America	United States	Enteritidis	Enteritidis	1425	17099
SAL_DA5722AA	NY-swgs1337	SRR1511576	2014	6	North America	United States	Enteritidis	Enteritidis	1425	17095
SAL_DA5754AA	MDH-2014-00836	SRR1511544	2014	6	North America	United States	Enteritidis	Enteritidis	1425	14388
SAL_DA5755AA	NY-swgs1340	SRR1511543	2014	6	North America	United States	Enteritidis	Enteritidis	1425	17068
SAL_DA5760AA	MDH-2014-00365	SRR1511538	2002	12	North America	United States	Enteritidis	Enteritidis	1425	17063
SAL_DA5780AA	NY-swgs1339	SRR1511518	2014	6	North America	United States	Enteritidis	Enteritidis	8003	12492
SAL_DA5786AA	NY-swgs1334	SRR1511512	2014	6	North America	United States	Enteritidis	Enteritidis	1425	16811
SAL_DA5794AA	NY-swgs1338	SRR1511503	2014	6	North America	United States	Enteritidis	Enteritidis	1425	15038
SAL_DA5803AA	NY-swgs1336	SRR1511494	2014	6	North America	United States	Enteritidis	Enteritidis	1425	17029
SAL_DA5804AA	NY-swgs1335	SRR1511493	2014	6	North America	United States	Enteritidis	Enteritidis	1425	17028
SAL_DA5805AA	CFSAN018635	SRR1511492	2007	1	North America	United States	Enteritidis	Enteritidis	1425	17027
SAL_DA5889AA	FDA00001496	SRR1503325	2010	12	Asia	China	Enteritidis	Enteritidis	1425	16960
SAL_DA5916AA	CFSAN018529	SRR1501708	2007	1	North America	United States	Enteritidis	Enteritidis	1425	13840
SAL_DA5920AA	CFSAN018539	SRR1501704	2007	1	North America	United States	Enteritidis	Enteritidis	1425	13840
SAL_DA5982AA	MDH-2014-00835	SRR1501640	2014	6	North America	United States	Enteritidis	Enteritidis	1425	16878
SAL_DA6020AA	MDH-2014-00834	SRR1501484	2014	6	North America	United States	Enteritidis	Enteritidis	1425	16845
SAL_DA6032AA	CFSAN018518	SRR1501472	2007	1	North America	United States	Enteritidis	Enteritidis	8469	16833

SAL_DA6051AA	NY-swgs1331	SRR1482402	2014	5	North America	United States	Enteritidis	Enteritidis	1425	14321
SAL_DA6052AA	NY-swgs1330	SRR1482401	2014	5	North America	United States	Enteritidis	Enteritidis	1425	16815
SAL_DA6053AA	NY-swgs1324	SRR1482400	2014	5	North America	United States	Enteritidis	Enteritidis	1425	16814
SAL_DA6054AA	NY-swgs1322	SRR1482399	2014	5	North America	United States	Enteritidis	Enteritidis	1425	16813
SAL_DA6055AA	NY-swgs1321	SRR1482398	2014	6	North America	United States	Enteritidis	Enteritidis	1425	16812
SAL_DA6058AA	NY-swgs1329	SRR1482392	2014	6	North America	United States	Enteritidis	Enteritidis	1425	16807
SAL_DA6059AA	NY-swgs1323	SRR1482391	2014	5	North America	United States	Enteritidis	Enteritidis	1425	16811
SAL_DA6060AA	NY-swgs1318	SRR1482389	2014	5	North America	United States	Enteritidis	Enteritidis	1425	16810
SAL_DA6061AA	NY-swgs1325	SRR1482386	2014	5	North America	United States	Enteritidis	Enteritidis	1425	16809
SAL_DA6063AA	NY-swgs1327	SRR1482384	2014	5	North America	United States	Enteritidis	Enteritidis	1425	16808
SAL_DA6064AA	NY-swgs1326	SRR1482383	2014	5	North America	United States	Enteritidis	Enteritidis	1425	14321
SAL_DA6065AA	NY-swgs1319	SRR1482382	2014	5	North America	United States	Enteritidis	Enteritidis	1425	651
SAL_DA6066AA	NY-swgs1328	SRR1482378	2014	5	North America	United States	Enteritidis	Enteritidis	1425	16807
SAL_DA6067AA	NY-swgs1320	SRR1482377	2014	5	North America	United States	Enteritidis	Enteritidis	1425	16807
SAL_DA6073AA	FNE0130	SRR1481932	2013	3	Asia	China	Enteritidis	Enteritidis	1425	16802
SAL_DA6094AA	NY-swgs1317	SRR1481687	2014	5	North America	United States	Enteritidis	Enteritidis	1425	16783
SAL_DA6097AA	FLDOH-40	SRR1481682	2014	3	North America	United States	Enteritidis	Enteritidis	1425	16780
SAL_DA6101AA	FLDOH-48	SRR1481676	2014	3	North America	United States	Enteritidis	Enteritidis	8003	16776
SAL_DA6106AA	FLDOH-41	SRR1481671	2014	3	North America	United States	Enteritidis	Enteritidis	1425	16772
SAL_DA6107AA	FLDOH-33	SRR1481670	2014	2	North America	United States	Enteritidis	Enteritidis	1425	16771
SAL_DA6108AA	FLDOH-39	SRR1481669	2014	2	North America	United States	Enteritidis	Enteritidis	1425	16770
SAL_DA6115AA	FLDOH-42	SRR1481661	2014	3	North America	United States	Enteritidis	Enteritidis	3888	16763
SAL_DA6154AA	FSE0166	SRR1449930	2011	7	North America	United States	Enteritidis	Enteritidis	1425	16728
SAL_DA6155AA	FSE0162	SRR1449929	2011	7	North America	United States	Enteritidis	Enteritidis	1425	16727
SAL_DA6156AA	FSE0168	SRR1449927	2011	7	North America	United States	Enteritidis	Enteritidis	1425	16726
SAL_DA6157AA	FSE0160	SRR1449926	2011	7	North America	United States	Enteritidis	Enteritidis	1425	16725
SAL_DA6158AA	FSE0158	SRR1449925	2011	5	Africa	Ethiopia	Ramatgan	Enteritidis	27697	16724
SAL_DA6159AA	FSE0167	SRR1449924	2011	7	North America	United States	Enteritidis	Enteritidis	1425	16723
SAL_DA6161AA	FSE0169	SRR1449922	2011	7	North America	United States	Enteritidis	Enteritidis	1425	16721
SAL_DA6162AA	FSE0165	SRR1449921	2011	7	North America	Costa Rica	Sandiego	Enteritidis	1425	16720
SAL_DA6163AA	FSE0161	SRR1449920	2011	7	North America	United States	Enteritidis	Enteritidis	1425	16719
SAL_DA6164AA	FSE0159	SRR1449919	2011	7	North America	United States	Enteritidis	Enteritidis	1425	16718
SAL_DA6167AA	FLDOH-27	SRR1448937	2014	1	North America	United States	Enteritidis	Enteritidis	1425	16715
SAL_DA6170AA	MDH-2014-00832	SRR1448934	2014	6	North America	United States	Enteritidis	Enteritidis	1425	16712
SAL_DA6183AA	MDH-2014-00833	SRR1448887	2014	6	North America	United States	Enteritidis	Enteritidis	1425	16702
SAL_DA6216AA	FSE0147	SRR1427724	2011	4	North America	United States	Enteritidis	Enteritidis	1425	16670
SAL_DA6221AA	FSE0152	SRR1427719	2011	5	North America	United States	Enteritidis	Enteritidis	1425	16666
SAL_DA6230AA	FSE0145	SRR1427710	2011	4	North America	United States	Enteritidis	Enteritidis	1425	16657
SAL_DA6232AA	FSE0138	SRR6071648	2011	3	North America	United States	Enteritidis	Enteritidis	1425	16655
SAL_DA6233AA	FSE0150	SRR1427706	2011	5	North America	United States	Enteritidis	Enteritidis	1425	16654
SAL_DA6238AA	NY-swgs1305	SRR1427107	2014	5	North America	United States	Enteritidis	Enteritidis	8003	12492
SAL_DA6239AA	NY-swgs1298	SRR1427106	2014	5	North America	United States	Enteritidis	Enteritidis	1425	12315
SAL_DA6243AA	NY-swgs1313	SRR1427102	2014	5	North America	United States	Enteritidis	Enteritidis	1425	16644
SAL_DA6244AA	NY-swgs1310	SRR1427101	2014	5	North America	United States	Enteritidis	Enteritidis	1425	16646
SAL_DA6245AA	NY-swgs1307	SRR1427100	2014	5	North America	United States	Enteritidis	Enteritidis	1425	16645
SAL_DA6246AA	NY-swgs1297	SRR1427099	2014	5	North America	United States	Enteritidis	Enteritidis	1425	16644
SAL_DA6249AA	NY-swgs1304	SRR1427096	2014	5	North America	United States	Enteritidis	Enteritidis	1425	16641
SAL_DA6250AA	NY-swgs1301	SRR1427095	2014		North America	United States	Enteritidis	Enteritidis	1425	16611
SAL_DA6251AA	NY-swgs1300	SRR1427094	2014	5	North America	United States	Enteritidis	Enteritidis	1425	16640
SAL_DA6252AA	NY-swgs1296	SRR1427093	2014	5	North America	United States	Enteritidis	Enteritidis	1425	16639
SAL_DA6255AA	NY-swgs1314	SRR1427090	2014	4	North America	United States	Enteritidis	Enteritidis	1425	16636
SAL_DA6256AA	NY-swgs1311	SRR1427089	2014	5	North America	United States	Enteritidis	Enteritidis	1425	16635
SAL_DA6257AA	NY-swgs1303	SRR1427088	2014	5	North America	United States	Enteritidis	Enteritidis	1425	16634
SAL_DA6261AA	NY-swgs1312	SRR1427084	2014	5	North America	United States	Enteritidis	Enteritidis	1425	16630
SAL_DA6262AA	NY-swgs1306	SRR1427083	2014	5	North America	United States	Enteritidis	Enteritidis	1425	2067
SAL_DA6263AA	NY-swgs1299	SRR1427082	2014	5	North America	United States	Enteritidis	Enteritidis	7801	16629
SAL_DA6283AA	NY-swgs1295	SRR1425273	2014	5	North America	United States	Enteritidis	Enteritidis	1425	16611
SAL_DA6291AA	MDH-2014-00831	SRR1425265	2014	6	North America	United States	Enteritidis	Enteritidis	1425	16605
SAL_DA6292AA	NY-swgs1293	SRR1425264	2014	4	North America	United States	Enteritidis	Enteritidis	1425	16604

SAL_DA6296AA	MDH-2014-00823	SRR1425243	2014	5	North America	United States	Enteritidis	Enteritidis	1425	16602
SAL_DA6297AA	NY-swgs1294	SRR1425242	2014	5	North America	United States	Enteritidis	Enteritidis	1425	16601
SAL_DA6301AA	MDH-2014-00825	SRR1422845	2014	4	North America	United States	Enteritidis	Enteritidis	1425	16597
SAL_DA6307AA	MDH-2014-00828	SRR1422670	2014	6	North America	United States	Enteritidis	Enteritidis	1425	16591
SAL_DA6310AA	MDH-2014-00826	SRR1422665	2014	5	North America	United States	Enteritidis	Enteritidis	1425	16588
SAL_DA6311AA	MDH-2014-00824	SRR1422663	2014	5	North America	United States	Enteritidis	Enteritidis	1425	14335
SAL_DA6313AA	MDH-2014-00830	SRR1422661	2014	5	North America	United States	Enteritidis	Enteritidis	8003	16586
SAL_DA6314AA	MDH-2014-00827	SRR1422660	2014	6	North America	United States	Enteritidis	Enteritidis	1425	16585
SAL_DA6333AA	MDH-2014-00829	SRR1422539	2014	5	North America	United States	Enteritidis	Enteritidis	1425	16566
SAL_DA6343AA	FLDOH-15	SRR1411168	2013	12	North America	United States		Enteritidis	1425	16558
SAL_DA6353AA	FMA0192	SRR1411152	2011	10	Asia	Turkey	Enteritidis	Enteritidis	1425	16548
SAL_DA6362AA	FMA0198	SRR1411138	2011	11	North America	United States	Enteritidis	Enteritidis	1425	16540
SAL_DA6387AA	FMA0193	SRR1411106	2011	10	Asia	Turkey	Enteritidis	Enteritidis	1425	11004
SAL_DA6391AA	MDH-2014-00817	SRR1395323	2014	5	North America	United States	Enteritidis	Enteritidis	1425	16512
SAL_DA6398AA	MDH-2014-00816	SRR1395308	2014	5	North America	United States	Enteritidis	Enteritidis	1425	16507
SAL_DA6440AA	2012K-0501	SRR1379787	2012		North America	United States	Enteritidis	Enteritidis	1425	16475
SAL_DA6441AA	2012K-0500	SRR1379786	2012		North America	United States	Enteritidis	Enteritidis	1425	16474
SAL_DA6442AA	2012K-0499	SRR1379785	2012		North America	United States	Enteritidis	Enteritidis	1425	16473
SAL_DA6443AA	2011K-0104	SRR1379784	2011		North America	United States	Enteritidis	Enteritidis	1425	16472
SAL_DA6444AA	2011K-0079	SRR1379783	2011		North America	United States	Enteritidis	Enteritidis	1425	16471
SAL_DA6445AA	2011K-0019	SRR1379782	2011		North America	United States	Enteritidis	Enteritidis	1425	16470
SAL_DA6446AA	2010K-2617	SRR1379781	2011		North America	United States	Enteritidis	Enteritidis	1425	16469
SAL_DA6447AA	2009K-1562	SRR1379780	2009		North America	United States	Enteritidis	Enteritidis	1425	16468
SAL_DA6448AA	2009K-1559	SRR1379779	2009		North America	United States	Enteritidis	Enteritidis	1425	16468
SAL_DA6449AA	2009K-1553	SRR1379778	2009		North America	United States	Enteritidis	Enteritidis	1425	16468
SAL_DA6450AA	2009K-1545	SRR1379777	2009		North America	United States	Enteritidis	Enteritidis	1425	6403
SAL_DA6451AA	K2083	SRR1379776	2005		North America	United States	Enteritidis	Enteritidis	1425	14261
SAL_DA6452AA	K2082	SRR1379775	2005		North America	United States	Enteritidis	Enteritidis	1425	16467
SAL_DA6453AA	K3310	SRR1379774	2006		North America	United States	Enteritidis	Enteritidis	1425	16466
SAL_DA6454AA	K3308	SRR1379773	2006		North America	United States	Enteritidis	Enteritidis	1425	15184
SAL_DA6455AA	K2331	SRR1379772	2005		North America	United States	Enteritidis	Enteritidis	1425	16465
SAL_DA6456AA	K2330	SRR1379771	2005		North America	United States	Enteritidis	Enteritidis	1425	16464
SAL_DA6457AA	H9558	SRR1379770	2003		North America	United States	Enteritidis	Enteritidis	27569	16463
SAL_DA6458AA	H9556	SRR1379769	2003		North America	United States	Enteritidis	Enteritidis	27569	16462
SAL_DA6459AA	J0905	SRR1379768	2001		North America	United States	Enteritidis	Enteritidis	1425	16461
SAL_DA6460AA	J0900	SRR1379767	2001		North America	United States	Enteritidis	Enteritidis	1425	16461
SAL_DA6462AA	2012K-0597	SRR1379765	2012		North America	United States	Enteritidis	Enteritidis	1425	16459
SAL_DA6463AA	2012K-0619	SRR1379764	2012		North America	United States	Enteritidis	Enteritidis	1425	16458
SAL_DA6464AA	2012K-0644	SRR1379763	2012		North America	United States	Enteritidis	Enteritidis	1425	13381
SAL_DA6465AA	2012K-0628	SRR1379762	2012		North America	United States	Enteritidis	Enteritidis	1425	13381
SAL_DA6466AA	2012K-0627	SRR1379761	2012		North America	United States	Enteritidis	Enteritidis	1425	16457
SAL_DA6469AA	2011K-1846	SRR1379758	2011		North America	United States	Enteritidis	Enteritidis	1425	16455
SAL_DA6470AA	2011K-1845	SRR1379757	2011		North America	United States	Enteritidis	Enteritidis	1425	16454
SAL_DA6471AA	2011K-1668	SRR1379756	2011		North America	United States	Enteritidis	Enteritidis	1425	11004
SAL_DA6472AA	2011K-1667	SRR1379755	2011	10	North America	United States	Enteritidis	Enteritidis	1425	11004
SAL_DA6473AA	2010K-0675	SRR1379754	2010		North America	United States	Enteritidis	Enteritidis	1425	16453
SAL_DA6474AA	2010K-0678	SRR1379753	2010		North America	United States	Enteritidis	Enteritidis	1425	16452
SAL_DA6475AA	2010K-0677	SRR1379752	2010		North America	United States	Enteritidis	Enteritidis	1425	16450
SAL_DA6476AA	2010K-0673	SRR1379751	2010		North America	United States	Enteritidis	Enteritidis	1425	16450
SAL_DA6477AA	2010K-0672	SRR1379750	2010		North America	United States	Enteritidis	Enteritidis	1425	16450
SAL_DA6478AA	2010K-0669	SRR1379749	2010		North America	United States	Enteritidis	Enteritidis	1425	16451
SAL_DA6479AA	2010K-0668	SRR1379748	2010		North America	United States	Enteritidis	Enteritidis	1425	16450
SAL_DA6480AA	2010K-0667	SRR1379747	2010		North America	United States	Enteritidis	Enteritidis	1425	16450
SAL_DA6481AA	2010K-0666	SRR1379746	2010		North America	United States	Enteritidis	Enteritidis	1425	16450
SAL_DA6482AA	2010K-1947	SRR1379745	2010		North America	United States	Enteritidis	Enteritidis	1425	16449
SAL_DA6483AA	2010K-1946	SRR1379744	2010		North America	United States	Enteritidis	Enteritidis	1425	16448
SAL_DA6484AA	2010K-0362	SRR1379743	2009		Africa	Mauritius	Enteritidis	Enteritidis	3888	16447
SAL_DA6485AA	2010K-0358	SRR1379742	2009		Africa	Mauritius	Enteritidis	Enteritidis	3888	16446
SAL_DA6486AA	2010K-0351	SRR1379741	2009		Africa	Mauritius	Enteritidis	Enteritidis	3888	16445

SAL_DA6487AA	2010K-0348	SRR1379740	2009		Africa	Mauritius	Enteritidis	Enteritidis	3888	16444
SAL_DA6488AA	2010K-0338	SRR1379739	2009		Africa	Mauritius	Enteritidis	Enteritidis	3888	16443
SAL_DA6489AA	2012K-0283	SRR1379738	2012		North America	United States	Enteritidis	Enteritidis	1425	16442
SAL_DA6490AA	2012K-0285	SRR1379737	2012		North America	United States	Enteritidis	Enteritidis	1425	16441
SAL_DA6491AA	2012K-0284	SRR1379736	2012		North America	United States	Enteritidis	Enteritidis	1425	16440
SAL_DA6592AA	CFSAN018300	SRR1346329	2014	5	North America	United States	Enteritidis	Enteritidis	1425	16358
SAL_DA6593AA	CFSAN018298	SRR1346328	2014	5	North America	United States	Enteritidis	Enteritidis	1425	16357
SAL_DA6594AA	NY-swgs1291	SRR1346327	2014	5	North America	United States	Enteritidis	Enteritidis	1425	16356
SAL_DA6596AA	CFSAN018305	SRR1346325	2014	5	North America	United States	Enteritidis	Enteritidis	1425	16354
SAL_DA6597AA	CFSAN018302	SRR1346324	2014	5	North America	United States	Enteritidis	Enteritidis	1425	16353
SAL_DA6598AA	NY-swgs1287	SRR1346323	2014	5	North America	United States	Enteritidis	Enteritidis	1425	16352
SAL_DA6604AA	IEH-NGS-SAL-00593	SRR1346317	2014	5	North America	United States	Enteritidis	Enteritidis	1425	16346
SAL_DA6610AA	NY-swgs1284	SRR1346311	2014	5	North America	United States	Enteritidis	Enteritidis	1425	16341
SAL_DA6613AA	CFSAN018312	SRR1346308	2014	5	North America	United States	Enteritidis	Enteritidis	1425	16339
SAL_DA6617AA	CFSAN018306	SRR1346304	2014	5	North America	United States	Enteritidis	Enteritidis	1425	16335
SAL_DA6618AA	CFSAN018301	SRR1346303	2014	5	North America	United States	Enteritidis	Enteritidis	1425	16334
SAL_DA6620AA	CFSAN018299	SRR1346301	2014	5	North America	United States	Enteritidis	Enteritidis	1425	16332
SAL_DA6621AA	CFSAN018293	SRR1346300	2014	5	North America	United States	Enteritidis	Enteritidis	3900	16331
SAL_DA6624AA	CFSAN018309	SRR1346297	2014	5	North America	United States	Enteritidis	Enteritidis	1425	16328
SAL_DA6625AA	CFSAN018303	SRR1346296	2014	5	North America	United States	Enteritidis	Enteritidis	1425	16327
SAL_DA6627AA	NY-swgs1288	SRR1346293	2014	5	North America	United States	Enteritidis	Enteritidis	1425	16325
SAL_DA6628AA	NY-swgs1285	SRR1346292	2014	5	North America	United States	Enteritidis	Enteritidis	1425	16324
SAL_DA6634AA	IEH-NGS-SAL-00596	SRR1346285	2014	5	North America	United States	Enteritidis	Enteritidis	1425	16320
SAL_DA6643AA	CFSAN018304	SRR1346276	2014	5	North America	United States	Enteritidis	Enteritidis	1425	16311
SAL_DA6644AA	NY-swgs1290	SRR1346275	2014	5	North America	United States	Enteritidis	Enteritidis	1425	14321
SAL_DA6650AA	NY-swgs1292	SRR1346268	2014	5	North America	United States	Enteritidis	Enteritidis	1425	16305
SAL_DA6651AA	FMA0175	SRR1346267	2011	9	Asia	China	Enteritidis	Enteritidis	1425	16303
SAL_DA6659AA	NY-swgs1286	SRR1346259	2014	5	North America	United States	Enteritidis	Enteritidis	1425	16296
SAL_DA6662AA	IEH-NGS-SAL-00599	SRR1346256	2014	5	North America	United States	Enteritidis	Enteritidis	1425	10599
SAL_DA6666AA	CFSAN018297	SRR1346251	2014	5	North America	United States	Enteritidis	Enteritidis	8384	16292
SAL_DA6669AA	CFSAN018308	SRR1346248	2014	5	North America	United States	Enteritidis	Enteritidis	1425	16289
SAL_DA6672AA	CFSAN018296	SRR1346245	2014	5	North America	United States	Enteritidis	Enteritidis	1425	16286
SAL_DA6673AA	CFSAN018295	SRR1346244	2014	5	North America	United States	Enteritidis	Enteritidis	1425	16285
SAL_DA6674AA	CFSAN018294	SRR1346243	2014	5	North America	United States	Enteritidis	Enteritidis	1425	16284
SAL_DA6675AA	NY-swgs1289	SRR1346242	2014	5	North America	United States	Enteritidis	Enteritidis	1425	16283
SAL_DA6692AA	MDH-2014-00815	SRR1334667	2014	5	North America	United States	Enteritidis	Enteritidis	1425	14388
SAL_DA6711AA	MDH-2014-00813	SRR1334489	2014	5	North America	United States	Enteritidis	Enteritidis	1425	16254
SAL_DA6715AA	MDH-2014-00814	SRR1334485	2014	5	North America	United States	Enteritidis	Enteritidis	1425	14335
SAL_DA6716AA	NY-swgs1279	SRR1334484	2014	4	North America	United States	Enteritidis	Enteritidis	1425	15921
SAL_DA6735AA	NY-swgs1283	SRR1334227	2014	5	North America	United States	Enteritidis	Enteritidis	1425	16241
SAL_DA6740AA	NY-swgs1281	SRR1334179	2014	4	North America	United States	Enteritidis	Enteritidis	26543	16237
SAL_DA6741AA	NY-swgs1280	SRR1334178	2014	4	North America	United States	Enteritidis	Enteritidis	1425	16236
SAL_DA6765AA	NY-swgs1282	SRR1333885	2014	4	North America	United States	Enteritidis	Enteritidis	1425	16216
SAL_DA6792AA	Enteritidis	SRR1321142	2012		Europe	United Kingdom	Enteritidis	Enteritidis	7811	16193
SAL_DA6796AA	FDA00004360	SRR1302870	2014	3	North America	United States	Enteritidis (Predicted)	Enteritidis	1425	16190
SAL_DA6801AA	MDH-2014-00810	SRR1302865	2014	5	North America	United States	Enteritidis	Enteritidis	1425	16186
SAL_DA6805AA	MDH-2014-00811	SRR1302861	2014	5	North America	United States	Enteritidis	Enteritidis	1425	14335
SAL_DA6819AA	MDH-2014-00809	SRR1302838	2014	5	North America	United States	Enteritidis	Enteritidis	1425	16172
SAL_DA6820AA	MDH-2014-00808	SRR1302837	2014	5	North America	United States	Enteritidis	Enteritidis	1425	16171
SAL_DA6821AA	FDA00004362	SRR1302836	2014	3	North America	United States	Enteritidis (Predicted)	Enteritidis	1425	16170
SAL_DA6824AA	MDH-2014-00812	SRR1302833	2014	5	North America	United States	Enteritidis	Enteritidis	1425	16167
SAL_DA6826AA	FDA00004363	SRR1302828	2014	3	North America	United States	Enteritidis (Predicted)	Enteritidis	1425	16165
SAL_DA6876AA	NY-swgs1276	SRR1300735	2014	4	North America	United States	Enteritidis	Enteritidis	1425	16120
SAL_DA6880AA	MDH-2014-00804	SRR1300731	2014	5	North America	United States	Enteritidis	Enteritidis	1425	16081
SAL_DA6887AA	NY-swgs1266	SRR1300723	2014	4	North America	United States	Enteritidis	Enteritidis	1425	16110
SAL_DA6898AA	NY-swgs1268	SRR1300712	2014	4	North America	United States	Enteritidis	Enteritidis	1425	16099
SAL_DA6903AA	MDH-2014-00803	SRR1300707	2014	5	North America	United States	Enteritidis	Enteritidis	1425	15038
SAL_DA6910AA	NY-swgs1278	SRR1300700	2014		North America	United States	Enteritidis	Enteritidis	1425	16088
SAL_DA6916AA	MDH-2014-00806	SRR1300694	2014	5	North America	United States	Enteritidis	Enteritidis	1425	16082

SAL_DA6917AA	MDH-2014-00802	SRR1300693	2014	5	North America	United States	Enteritidis	Enteritidis	1425	16081
SAL_DA6937AA	MDH-2014-00805	SRR1300671	2014	5	North America	United States	Enteritidis	Enteritidis	1425	15673
SAL_DA6940AA	NY-swgs1272	SRR1300668	2014	4	North America	United States	Enteritidis	Enteritidis	1425	16061
SAL_DA6954AA	NY-swgs1277	SRR1300650	2014	4	North America	United States	Enteritidis	Enteritidis	1425	16051
SAL_DA6958AA	FL-NRM042	SRR1300645	2004		North America	United States	Enteritidis	Enteritidis	1425	16047
SAL_DA6960AA	NY-swgs1274	SRR1300643	2014	4	North America	United States	Enteritidis	Enteritidis	1425	16045
SAL_DA6975AA	NY-swgs1269	SRR1300627	2014	4	North America	United States	Enteritidis	Enteritidis	1425	16032
SAL_DA6991AA	NY-swgs1271	SRR1300601	2014	4	North America	United States	Enteritidis	Enteritidis	1425	16017
SAL_DA6997AA	NY-swgs1273	SRR1300594	2014	4	North America	United States	Enteritidis	Enteritidis	1425	16012
SAL_DA6998AA	MDH-2014-00800	SRR1300593	2014	5	North America	United States	Enteritidis	Enteritidis	1425	14388
SAL_DA6999AA	FMA0169	SRR1300592	2011	8	Asia	Syria	Enteritidis	Enteritidis	27438	16011
SAL_DA7008AA	NY-swgs1267	SRR1300583	2014	4	North America	United States	Enteritidis	Enteritidis	1425	16004
SAL_DA7015AA	NY-swgs1270	SRR1300566	2014	4	North America	United States	Enteritidis	Enteritidis	1425	14339
SAL_DA7021AA	NY-swgs1275	SRR1300560	2014	4	North America	United States	Enteritidis	Enteritidis	1425	15992
SAL_DA7030AA	VA-WGS-00247	SRR1299434	2010	4	North America	United States	Enteritidis	Enteritidis	1425	15983
SAL_DA7041AA	WAPHL-SAL-A00337	SRR1299423	2002		North America	United States	Enteritidis	Enteritidis	1425	14768
SAL_DA7044AA	NY-swgs1261	SRR1299420	2014	4	North America	United States	Enteritidis	Enteritidis	1425	15972
SAL_DA7048AA	VA-WGS-00246	SRR1299399	2010	4	North America	United States	Enteritidis	Enteritidis	1425	15968
SAL_DA7054AA	NY-swgs1259	SRR1299392	2014	4	North America	United States	Enteritidis	Enteritidis	1425	15963
SAL_DA7078AA	NY-swgs1262	SRR1299360	2014	4	North America	United States	Enteritidis	Enteritidis	1425	15944
SAL_DA7079AA	NY-swgs1258	SRR1299359	2014	4	North America	United States	Enteritidis	Enteritidis	1425	15943
SAL_DA7109AA	NY-swgs1257	SRR1299295	2014	4	North America	United States	Enteritidis	Enteritidis	1425	15921
SAL_DA7132AA	MDH-2014-00009	SRR1299270	2003	8	North America	United States	Enteritidis	Enteritidis	7801	15900
SAL_DA7140AA	NY-swgs1263	SRR1299215	2014	4	North America	United States	Enteritidis	Enteritidis	1425	15892
SAL_DA7147AA	FDA00000036	SRR1292330			North America	United States	Enteritidis (Predicted)	Enteritidis	1425	15886
SAL_DA7169AA	FDA00000033	SRR1292281			North America	United States	Enteritidis (Predicted)	Enteritidis	1425	15865
SAL_DA7175AA	FDA00000037	SRR1292275			North America	United States	Enteritidis (Predicted)	Enteritidis	1425	15859
SAL_DA7186AA	FDA00000035	SRR1292264			North America	United States	Enteritidis (Predicted)	Enteritidis	1425	15848
SAL_DA7187AA	FDA00000034	SRR1292263			North America	United States	Enteritidis (Predicted)	Enteritidis	1425	15847
SAL_DA7246AA	MDH-2014-00801	SRR1284731	2014	4	North America	United States	Enteritidis	Enteritidis	1425	15795
SAL_DA7249AA	MDH-2014-00799	SRR1284716	2014	4	North America	United States	Enteritidis	Enteritidis	1425	15792
SAL_DA7330AA	NY-swgs1252	SRR1272888	2014	3	North America	United States	Enteritidis	Enteritidis	1425	15741
SAL_DA7342AA	NY-swgs1250	SRR1272866	2014	4	North America	United States	Enteritidis	Enteritidis	7801	15729
SAL_DA7344AA	NY-swgs1254	SRR1272864	2014	4	North America	United States	Enteritidis	Enteritidis	1425	3809
SAL_DA7352AA	WAPHL-SAL-A00376	SRR1272853	2003		North America	United States	Enteritidis	Enteritidis	1425	15720
SAL_DA7368AA	NY-swgs1255	SRR1272790	2014	4	North America	United States	Enteritidis	Enteritidis	1425	15704
SAL_DA7384AA	MDH-2014-00797	SRR1272538	2014	4	North America	United States	Enteritidis	Enteritidis	1425	15690
SAL_DA7395AA	MDH-2014-00795	SRR1272518	2014	4	North America	United States	Enteritidis	Enteritidis	1425	14319
SAL_DA7396AA	NY-swgs1251	SRR1272516	2014	4	North America	United States	Enteritidis	Enteritidis	27443	15680
SAL_DA7397AA	NY_BAC0900005400-1	SRR1272515	2009	11	North America	United States	Enteritidis	Enteritidis	1425	2514
SAL_DA7398AA	NY-swgs1256	SRR1272514	2014	4	North America	United States	Enteritidis	Enteritidis	1425	15679
SAL_DA7405AA	MDH-2014-00796	SRR1272502	2014	4	North America	United States	Enteritidis	Enteritidis	1425	15673
SAL_DA7413AA	VA_WGS-00196	SRR1272492	2011	10	North America	United States	Enteritidis	Enteritidis	1425	15666
SAL_DA7420AA	NY-swgs1253	SRR1272476	2014	4	North America	United States	Enteritidis	Enteritidis	1425	15660
SAL_DA7423AA	D3063	ERR037597					Enteritidis (Predicted)	Enteritidis	1424	15658
SAL_DA7424AA	A3582	ERR037599					Enteritidis	Enteritidis	1425	15657
SAL_DA7425AA	D3314	ERR037600					Enteritidis	Enteritidis	1424	15656
SAL_DA7426AA	D1374	ERR037601					Enteritidis	Enteritidis	1424	15655
SAL_DA7427AA	A9395	ERR037602					Enteritidis (Predicted)	Enteritidis	1424	15654
SAL_DA7428AA	D10599	ERR037603					Enteritidis (Predicted)	Enteritidis	1424	15653
SAL_DA7429AA	D7795	ERR037604						Enteritidis	-2006	15652
SAL_DA7430AA	D3098	ERR037605					Enteritidis (Predicted)	Enteritidis	1424	15651
SAL_DA7431AA	D3493	ERR037606					Enteritidis (Predicted)	Enteritidis	1424	15650
SAL_DA7433AA	A2331	ERR037608					Enteritidis (Predicted)	Enteritidis	1424	15648
SAL_DA7434AA	A850	ERR037609					Enteritidis	Enteritidis	1424	15647
SAL_DA7446AA	Q71AS-sc-2011-05-05T14:42	ERR037596					Typhimurium	Enteritidis	1424	15638
SAL_DA7447AA	A38441-sc-2011-03-02-1044	ERR037559					Enteritidis (Predicted)	Enteritidis	-1931	15637
SAL_DA7448AA	D36956-sc-2011-03-07-1045	ERR037560					Enteritidis (Predicted)	Enteritidis	1424	15636
SAL_DA7449AA	D25809-sc-2011-03-07-1045	ERR037561					Enteritidis (Predicted)	Enteritidis	1425	15635

SAL_DA7451AA	D23145-sc-2011-03-31T14:0	ERR037563					Enteritidis	Enteritidis	1424	15633
SAL_DA7452AA	D40290-sc-2011-03-31T14:0	ERR037564					Enteritidis	Enteritidis	1424	15632
SAL_DA7454AA	D35807-sc-2011-03-31T14:0	ERR037566					Enteritidis	Enteritidis	7874	15630
SAL_DA7456AA	A39243-sc-2011-03-07-1044	ERR037568					Enteritidis (Predicted)	Enteritidis	1425	15629
SAL_DA7457AA	A39959-sc-2011-03-07-1045	ERR037569					Enteritidis (Predicted)	Enteritidis	1425	15628
SAL_DA7458AA	D36263-sc-2011-03-07-1045	ERR037570					Enteritidis (Predicted)	Enteritidis	1424	15627
SAL_DA7466AA	D23974-sc-2011-03-07-1045	ERR037579					Enteritidis (Predicted)	Enteritidis	3745	15622
SAL_DA7467AA	D24443-sc-2011-03-07-1046	ERR037580					Enteritidis (Predicted)	Enteritidis	1424	15621
SAL_DA7502AA	MDH-2014-00794	SRR1269368	2014	4	North America	United States	Enteritidis	Enteritidis	1425	15595
SAL_DA7513AA	MDH-2014-00793	SRR1269352	2014	4	North America	United States	Enteritidis	Enteritidis	1425	15584
SAL_DA7802AA	MDH-2014-00791	SRR1264970	2014	4	North America	United States	Enteritidis	Enteritidis	1425	15388
SAL_DA7821AA	MDH-2014-00792	SRR1264941	2014	4	North America	United States	Enteritidis	Enteritidis	1425	15370
SAL_DA7834AA	NY-swgs1249	SRR1263931	2014	3	North America	United States	Enteritidis	Enteritidis	1425	15357
SAL_DA7835AA	NY-swgs1248	SRR1263930	2014	3	North America	United States	Enteritidis	Enteritidis	1425	15071
SAL_DA7859AA	MDH-2014-00790	SRR1263425	2014	4	North America	United States	Enteritidis	Enteritidis	1425	15336
SAL_DA7866AA	MDH-2014-00789	SRR1263418	2014	4	North America	United States	Enteritidis	Enteritidis	1425	15331
SAL_DA7968AA	NY-swgs1234	SRR1258655	2014	3	North America	United States	Enteritidis	Enteritidis	1425	15230
SAL_DA7972AA	VA_WGS-00190	SRR1258650	2009	7	North America	United States	Enteritidis	Enteritidis	1425	15226
SAL_DA7978AA	NY-swgs1229	SRR1258631	2014	2	North America	United States	Enteritidis	Enteritidis	1425	15220
SAL_DA7981AA	MDH-2014-00786	SRR1258628	2014	4	North America	United States	Enteritidis	Enteritidis	1425	15217
SAL_DA7991AA	MDH-2014-00788	SRR1258613	2014	4	North America	United States	Enteritidis	Enteritidis	1425	15207
SAL_DA7992AA	NY-swgs1230	SRR1258612	2014	2	North America	United States	Enteritidis	Enteritidis	1425	15206
SAL_DA8002AA	MDH-2014-00787	SRR1258600	2014	4	North America	United States	Enteritidis	Enteritidis	1425	15197
SAL_DA8008AA	MDH-2014-00785	SRR1258592	2014	4	North America	United States	Enteritidis	Enteritidis	1425	2067
SAL_DA8010AA	NY-swgs1235	SRR1258588	2014	3	North America	United States	Enteritidis	Enteritidis	1425	15191
SAL_DA8014AA	MDH-2014-00784	SRR1258584	2014	4	North America	United States	Enteritidis	Enteritidis	1425	15187
SAL_DA8016AA	NY-swgs1233	SRR1258582	2014	1	North America	United States	Enteritidis	Enteritidis	1425	15185
SAL_DA8017AA	MDH-2014-00128	SRR1258581	2006	2	North America	United States	Enteritidis	Enteritidis	1425	15184
SAL_DA8025AA	FSW0122	SRR1258572	2013	5	Asia	Pakistan	Enteritidis	Enteritidis	1425	15176
SAL_DA8047AA	MDH-2014-00782	SRR1257318	2014	4	North America	United States	Enteritidis	Enteritidis	1425	15156
SAL_DA8060AA	MDH-2014-00783	SRR1257305	2014	4	North America	United States	Enteritidis	Enteritidis	1425	15146
SAL_DA8071AA	FSW0107	SRR1257292	2013	2	Asia	Indonesia	Enteritidis (Predicted)	Enteritidis	1425	15136
SAL_DA8132AA	MDH-2014-00143	SRR1248810	2006	6	North America	United States	Enteritidis	Enteritidis	1425	15078
SAL_DA8136AA	FAR0085	SRR1248786	2011	8	Asia	Pakistan	Enteritidis	Enteritidis	3888	15074
SAL_DA8140AA	MDH-2014-00779	SRR1238797	2014	3			Enteritidis	Enteritidis	1425	2067
SAL_DA8141AA	NY-swgs1247	SRR1238796	2014	3	North America	United States	Enteritidis	Enteritidis	1425	15071
SAL_DA8151AA	NY-swgs1246	SRR1238786	2014	3			Enteritidis	Enteritidis	1425	15064
SAL_DA8152AA	NY-swgs1244	SRR1238785	2014	3	North America	United States	Enteritidis	Enteritidis	1425	15038
SAL_DA8160AA	MDH-2014-00780	SRR1238776	2014	3			Enteritidis	Enteritidis	1425	15057
SAL_DA8170AA	NY-swgs1243	SRR1238766	2014	3	North America	United States	Enteritidis	Enteritidis	1425	15052
SAL_DA8181AA	MDH-2014-00781	SRR1238753	2014	4			Enteritidis	Enteritidis	1425	15043
SAL_DA8187AA	NY-swgs1245	SRR1238742	2014	3	North America	United States	Enteritidis	Enteritidis	1425	15038
SAL_DA8351AA	Q23F1	ERR028261	2002		Africa	Malawi	Enteritidis (Predicted)	Enteritidis	1425	14852
SAL_DA8358AA	Q255F2_U	ERR028268	2002		Africa	Malawi	Enteritidis (Predicted)	Enteritidis	1424	14879
SAL_DA8388AA	Q23F2_BM	ERR028251	2002		Africa	Malawi	Enteritidis (Predicted)	Enteritidis	1425	14852
SAL_DA8390AA	Q23A	ERR028253	2002		Africa	Malawi	Enteritidis (Predicted)	Enteritidis	1425	14852
SAL_DA8404AA	NY-swgs1236	SRR1233951	2014	3			Enteritidis	Enteritidis	1425	14838
SAL_DA8426AA	MDH-2014-00778	SRR1233929	2014	3	North America	United States	Enteritidis	Enteritidis	1425	14816
SAL_DA8450AA	swgs1200	SRR1220816	2014	1	North America	United States	Enteritidis	Enteritidis	1425	14326
SAL_DA8451AA	swgs1211	SRR1220812	2014	1	North America	United States	Enteritidis	Enteritidis	1425	14798
SAL_DA8456AA	WAPHL-SAL-A00328	SRR1220800	2002		North America	United States	Enteritidis	Enteritidis	3801	14794
SAL_DA8461AA	NY-swgs1240	SRR1220788	2014	3	North America	United States	Enteritidis	Enteritidis	1425	14789
SAL_DA8466AA	NY-swgs1239	SRR1220773	2014	3	North America	United States	Enteritidis	Enteritidis	1425	14787
SAL_DA8467AA	swgs1201	SRR1220771	2014	1	North America	United States	Enteritidis	Enteritidis	1425	14786
SAL_DA8470AA	NY-swgs1242	SRR1220760	2014	3	North America	United States	Enteritidis	Enteritidis	1425	14783
SAL_DA8472AA	NY-swgs1241	SRR1220754	2014	3	North America	United States	Enteritidis	Enteritidis	1425	14781
SAL_DA8475AA	NY-swgs1238	SRR1220747	2014	3	North America	United States	Enteritidis	Enteritidis	1425	14778
SAL_DA8477AA	WAPHL-SAL-A00324	SRR1220744	2002		North America	United States	Enteritidis	Enteritidis	1425	14776
SAL_DA8482AA	WAPHL-SAL-A00335	SRR1220732	2002		North America	United States	Enteritidis	Enteritidis	1425	14771

SAL_DA8485AA	WAPHL-SAL-A00336	SRR1220722	2002		North America	United States	Enteritidis	Enteritidis	1425	14768
SAL_DA8489AA	WAPHL-SAL-A00323	SRR1220713	2002		North America	United States	Enteritidis	Enteritidis	1425	14765
SAL_DA8545AA	FSE0057	SRR1212344	2011	7	North America	United States	Enteritidis	Enteritidis	1425	14716
SAL_DA8571AA	FSE0083	SRR1212298	2010	6	North America	United States	Enteritidis (Predicted)	Enteritidis	1425	14692
SAL_DA8602AA	VA_WGS-00029	SRR1207551	2005	10	North America	United States	Enteritidis	Enteritidis	1425	14663
SAL_DA8609AA	VA_WGS-00032	SRR1207515	2006	3	North America	United States	Enteritidis	Enteritidis	1425	14656
SAL_DA8620AA	VA_WGS-00033	SRR1207483	2006	5	North America	United States	Enteritidis	Enteritidis	1425	14637
SAL_DA8627AA	VA_WGS-00030	SRR1207468	2005	11	North America	United States	Enteritidis	Enteritidis	1425	14640
SAL_DA8630AA	VA_WGS-00028	SRR1207461	2005	10	North America	United States	Enteritidis	Enteritidis	1425	14637
SAL_DA8631AA	VA_WGS-00027	SRR1207460	2005	9	North America	United States	Enteritidis	Enteritidis	1425	14636
SAL_DA8638AA	VA_WGS-00031	SRR1207440	2005	12	North America	United States	Enteritidis	Enteritidis	1425	14629
SAL_DA8640AA	CFSAN004342	SRR1206205	2013	4	North America	United States	Enteritidis	Enteritidis	1425	14627
SAL_DA8676AA	swgs1228	SRR1203004	2014	2	North America	United States	Enteritidis	Enteritidis	1425	10271
SAL_DA8677AA	swgs1227	SRR1203003	2014	2	North America	United States	Enteritidis	Enteritidis	1425	14594
SAL_DA8696AA	AZ_TG71151	SRR1201356	2013	7	North America	United States	Enteritidis	Enteritidis	27732	14579
SAL_DA8758AA	swgs1223	SRR1198974	2014	2	North America	United States	Enteritidis	Enteritidis	8003	12492
SAL_DA8795AA	swgs1226	SRR1198920	2014	2	North America	United States	Enteritidis	Enteritidis	8003	12492
SAL_DA8796AA	swgs1224	SRR1198919	2014	2	North America	United States	Enteritidis	Enteritidis	8003	14494
SAL_DA8819AA	swgs1225	SRR1198886	2014	2	North America	United States	Enteritidis	Enteritidis	1425	14477
SAL_DA8852AA	AZ_TG71143	SRR1195697	2013	7	North America	United States	Enteritidis	Enteritidis	1425	14445
SAL_DA8900AA	swgs1074	SRR1187617	2013	12	North America	United States	Enteritidis	Enteritidis	1425	14360
SAL_DA8901AA	swgs1213	SRR1187615	2014	1	North America	United States	Enteritidis	Enteritidis	1425	14402
SAL_DA8902AA	swgs1085	SRR1187614	2011	2	North America	United States	Enteritidis	Enteritidis	1425	14401
SAL_DA8903AA	swgs1047	SRR1187612	2013	11	North America	United States	Enteritidis	Enteritidis	1425	2668
SAL_DA8904AA	swgs1057	SRR1187611	2013	11	North America	United States	Enteritidis	Enteritidis	1425	14322
SAL_DA8905AA	swgs1034	SRR1187610	2013	10	North America	United States	Enteritidis	Enteritidis	1425	14400
SAL_DA8906AA	swgs1023	SRR1187609	2013	10	North America	United States	Enteritidis	Enteritidis	3888	14399
SAL_DA8907AA	swgs1046	SRR1187608	2013	10	North America	United States	Enteritidis	Enteritidis	1425	14388
SAL_DA8908AA	swgs1089	SRR1187607	2013	12	North America	United States	Enteritidis	Enteritidis	1425	14398
SAL_DA8909AA	swgs1081	SRR1187606	2013	12	North America	United States	Enteritidis	Enteritidis	1425	14397
SAL_DA8910AA	swgs1031	SRR1187605	2013	10	North America	United States	Enteritidis	Enteritidis	1425	14396
SAL_DA8911AA	swgs1198	SRR1187604	2014	1	North America	United States	Enteritidis	Enteritidis	1425	14395
SAL_DA8912AA	swgs1084	SRR1187603	2008	10	North America	United States	Enteritidis	Enteritidis	1425	14394
SAL_DA8913AA	swgs1028	SRR1187602	2013	10	North America	United States	Enteritidis	Enteritidis	1425	14393
SAL_DA8914AA	swgs1059	SRR1187601	2013	11	North America	United States	Enteritidis	Enteritidis	1425	5730
SAL_DA8915AA	swgs1040	SRR1187600	2013	11	North America	United States	Enteritidis	Enteritidis	1425	2067
SAL_DA8916AA	swgs1026	SRR1187599	2013	9	North America	United States	Enteritidis	Enteritidis	1425	14392
SAL_DA8917AA	swgs1214	SRR1187597	2014	2	North America	United States	Enteritidis	Enteritidis	1425	14391
SAL_DA8918AA	swgs1194	SRR1187596	2013	12	North America	United States	Enteritidis	Enteritidis	1425	14390
SAL_DA8919AA	swgs1049	SRR1187595	2013	11	North America	United States	Enteritidis	Enteritidis	1425	14389
SAL_DA8920AA	swgs1025	SRR1187594	2013	10	North America	United States	Enteritidis	Enteritidis	1425	14388
SAL_DA8921AA	swgs1054	SRR1187593	2013	11	North America	United States	Enteritidis	Enteritidis	1425	14319
SAL_DA8922AA	swgs1017	SRR1187592	2013	10	North America	United States	Enteritidis	Enteritidis	1425	14322
SAL_DA8923AA	swgs1053	SRR1187591	2013	11	North America	United States	Enteritidis	Enteritidis	27443	14387
SAL_DA8924AA	swgs1037	SRR1187590	2013	10	North America	United States	Enteritidis	Enteritidis	1425	14386
SAL_DA8925AA	swgs1070	SRR1187588	2013	12	North America	United States	Enteritidis	Enteritidis	1425	14385
SAL_DA8926AA	swgs1051	SRR1187586	2013	11	North America	United States	Enteritidis	Enteritidis	1425	14323
SAL_DA8927AA	swgs1032	SRR1187585	2013	10	North America	United States	Enteritidis	Enteritidis	1425	14384
SAL_DA8928AA	swgs1094	SRR1187583	2013	12	North America	United States	Enteritidis	Enteritidis	1425	14383
SAL_DA8929AA	swgs1038	SRR1187582	2013	10	North America	United States	Enteritidis	Enteritidis	1425	14382
SAL_DA8930AA	swgs1068	SRR1187581	2013	12	North America	United States	Enteritidis	Enteritidis	1425	14360
SAL_DA8931AA	swgs1197	SRR1187580	2014	1	North America	United States	Enteritidis	Enteritidis	1425	14381
SAL_DA8932AA	swgs1067	SRR1187579	2013	12	North America	United States	Enteritidis	Enteritidis	1425	2067
SAL_DA8933AA	swgs1035	SRR1187578	2013	10	North America	United States	Enteritidis	Enteritidis	1425	4170
SAL_DA8934AA	swgs1024	SRR1187577	2013	9	North America	United States	Enteritidis	Enteritidis	1425	14380
SAL_DA8935AA	swgs1222	SRR1187575	2014	2	North America	United States	Enteritidis	Enteritidis	1425	14379
SAL_DA8936AA	swgs1045	SRR1187574	2013	11	North America	United States	Enteritidis	Enteritidis	1425	14376
SAL_DA8937AA	swgs1039	SRR1187573	2013	11	North America	United States	Enteritidis	Enteritidis	1425	14378
SAL_DA8938AA	swgs1030	SRR1187572	2013	10	North America	United States	Enteritidis	Enteritidis	8384	14377

SAL_DA8939AA	swgs1036	SRR1187571	2013	10	North America	United States	Enteritidis	Enteritidis	1425	14376
SAL_DA8940AA	swgs1022	SRR1187570	2013	9	North America	United States	Enteritidis	Enteritidis	1425	14375
SAL_DA8941AA	swgs1195	SRR1187569	2013	12	North America	United States	Enteritidis	Enteritidis	1425	14374
SAL_DA8942AA	swgs1066	SRR1187568	2013	11	North America	United States	Enteritidis	Enteritidis	1425	14373
SAL_DA8943AA	swgs1019	SRR1187567	2013	2	North America	United States	Enteritidis	Enteritidis	1425	14372
SAL_DA8944AA	swgs1208	SRR1187459	2014	1	North America	United States	Enteritidis	Enteritidis	1425	14371
SAL_DA8945AA	swgs1080	SRR1187458	2013	12	North America	United States	Enteritidis	Enteritidis	1425	14370
SAL_DA8946AA	swgs1056	SRR1187457	2013	11	North America	United States	Enteritidis	Enteritidis	1425	14369
SAL_DA8947AA	swgs1203	SRR1187456	2014	1	North America	United States	Enteritidis	Enteritidis	26756	14368
SAL_DA8948AA	swgs1210	SRR1187455	2014	1	North America	United States	Enteritidis	Enteritidis	1425	14367
SAL_DA8949AA	swgs1082	SRR1187454	2013	12	North America	United States	Enteritidis	Enteritidis	1425	14366
SAL_DA8950AA	swgs1042	SRR1187453	2013	11	North America	United States	Enteritidis	Enteritidis	1425	14365
SAL_DA8951AA	swgs1095	SRR1187452	2013	12	North America	United States	Enteritidis	Enteritidis	1425	14364
SAL_DA8952AA	swgs1072	SRR1187451	2013	12	North America	United States	Enteritidis	Enteritidis	1425	14326
SAL_DA8953AA	swgs1063	SRR1187450	2013	11	North America	United States	Enteritidis	Enteritidis	1425	14363
SAL_DA8954AA	swgs1071	SRR1187449	2013	12	North America	United States	Enteritidis	Enteritidis	1425	14362
SAL_DA8955AA	swgs1021	SRR1187448	2013	9	North America	United States	Enteritidis	Enteritidis	1425	14345
SAL_DA8956AA	swgs1204	SRR1187447	2014	1	North America	United States	Enteritidis	Enteritidis	1425	14361
SAL_DA8957AA	swgs1073	SRR1187446	2013	12	North America	United States	Enteritidis	Enteritidis	1425	14360
SAL_DA8958AA	swgs1064	SRR1187444	2013	11	North America	United States	Enteritidis	Enteritidis	1425	14359
SAL_DA8959AA	swgs1044	SRR1187443	2013	11	North America	United States	Enteritidis	Enteritidis	1425	14358
SAL_DA8960AA	swgs1033	SRR1187441	2013	10	North America	United States	Enteritidis	Enteritidis	1425	14357
SAL_DA8961AA	swgs1206	SRR1187439	2014	1	North America	United States	Enteritidis	Enteritidis	1425	14356
SAL_DA8962AA	swgs1069	SRR1187438	2013	12	North America	United States	Enteritidis	Enteritidis	1425	14355
SAL_DA8963AA	swgs1215	SRR1187437	2014	1	North America	United States	Enteritidis	Enteritidis	1425	14354
SAL_DA8964AA	swgs1060	SRR1187436	2013	11	North America	United States	Enteritidis	Enteritidis	1425	14353
SAL_DA8965AA	swgs1050	SRR1187435	2013	11	North America	United States	Enteritidis	Enteritidis	1425	14352
SAL_DA8966AA	swgs1207	SRR1187434	2014	1	North America	United States	Enteritidis	Enteritidis	1425	14351
SAL_DA8967AA	swgs1077	SRR1187433	2013	12	North America	United States	Enteritidis	Enteritidis	1425	14350
SAL_DA8968AA	swgs1041	SRR1187432	2013	11	North America	United States	Enteritidis	Enteritidis	1425	2668
SAL_DA8969AA	swgs1083	SRR1187431	2006	11	North America	United States	Enteritidis	Enteritidis	1425	14349
SAL_DA8970AA	swgs1076	SRR1187430	2013	12	North America	United States	Enteritidis	Enteritidis	1425	14348
SAL_DA8971AA	swgs1065	SRR1187429	2013	11	North America	United States	Enteritidis	Enteritidis	1425	14347
SAL_DA8972AA	swgs1020	SRR1187428	2013	2	North America	United States	Enteritidis	Enteritidis	1425	14346
SAL_DA8973AA	swgs1088	SRR1187426	2013	12	North America	United States	Enteritidis	Enteritidis	1425	14345
SAL_DA8974AA	swgs1209	SRR1187424	2014	1	North America	United States	Enteritidis	Enteritidis	1425	14344
SAL_DA8975AA	swgs1062	SRR1187422	2013	11	North America	United States	Enteritidis	Enteritidis	1425	14343
SAL_DA8976AA	swgs1052	SRR1187421	2013	11	North America	United States	Enteritidis	Enteritidis	1425	14342
SAL_DA8977AA	swgs1091	SRR1187419	2013	12	North America	United States	Enteritidis	Enteritidis	1425	14341
SAL_DA8978AA	swgs1086	SRR1187418	2013	12	North America	United States	Enteritidis	Enteritidis	1425	14340
SAL_DA8979AA	swgs1079	SRR1187417	2013	12	North America	United States	Enteritidis	Enteritidis	8003	12492
SAL_DA8980AA	swgs1218	SRR1187416	2014	2	North America	United States	Enteritidis	Enteritidis	1425	14339
SAL_DA8981AA	swgs1078	SRR1187415	2013	12	North America	United States	Enteritidis	Enteritidis	1425	14338
SAL_DA8982AA	swgs1048	SRR1187414	2013	11	North America	United States	Enteritidis	Enteritidis	1425	14322
SAL_DA8983AA	swgs1029	SRR1187411	2013	10	North America	United States	Enteritidis	Enteritidis	1425	14337
SAL_DA8984AA	swgs1092	SRR1187410	2013	12	North America	United States	Enteritidis	Enteritidis	1425	14336
SAL_DA8985AA	swgs1058	SRR1187409	2013	11	North America	United States	Enteritidis	Enteritidis	1425	14335
SAL_DA8986AA	swgs1043	SRR1187408	2013	11	North America	United States	Enteritidis	Enteritidis	1425	14323
SAL_DA8987AA	swgs1217	SRR1187407	2014	2	North America	United States	Enteritidis	Enteritidis	8003	14334
SAL_DA8988AA	swgs1212	SRR1187406	2014	1	North America	United States	Enteritidis	Enteritidis	1425	14333
SAL_DA8989AA	swgs1205	SRR1187405	2014	1	North America	United States	Enteritidis	Enteritidis	1425	14332
SAL_DA8990AA	swgs1018	SRR1187404	2013	10	North America	United States	Enteritidis	Enteritidis	8003	14331
SAL_DA8991AA	swgs1220	SRR1187397	2014	2	North America	United States	Enteritidis	Enteritidis	1425	14330
SAL_DA8992AA	swgs1196	SRR1187396	2013	12	North America	United States	Enteritidis	Enteritidis	1425	14329
SAL_DA8993AA	swgs1027	SRR1187395	2013	10	North America	United States	Enteritidis	Enteritidis	1425	14328
SAL_DA8994AA	swgs1216	SRR1187394	2014	2	North America	United States	Enteritidis	Enteritidis	1425	14327
SAL_DA8995AA	swgs1093	SRR1187392	2013	12	North America	United States	Enteritidis	Enteritidis	1425	14326
SAL_DA8996AA	swgs1221	SRR1187391	2014	2	North America	United States	Enteritidis	Enteritidis	1425	14325
SAL_DA8997AA	swgs1090	SRR1187390	2013	12	North America	United States	Enteritidis	Enteritidis	1425	14324

SAL_DA8998AA	swgs1087	SRR1187389	2013	12	North America	United States	Enteritidis	Enteritidis	1425	14323
SAL_DA8999AA	swgs1061	SRR1187388	2013	11	North America	United States	Enteritidis	Enteritidis	1425	14322
SAL_DA9000AA	swgs1219	SRR1187387	2014	2	North America	United States	Enteritidis	Enteritidis	1425	14321
SAL_DA9001AA	swgs1075	SRR1187386	2013	12	North America	United States	Enteritidis	Enteritidis	1425	14320
SAL_DA9002AA	swgs1055	SRR1187385	2013	11	North America	United States	Enteritidis	Enteritidis	1425	14319
SAL_DA9003AA	MDH-2014-00246	SRR1188546	2012	7	North America	United States	Enteritidis	Enteritidis	1425	14318
SAL_DA9004AA	MDH-2014-00227	SRR1188545	2001	6	North America	United States	Enteritidis	Enteritidis	1425	14317
SAL_DA9005AA	MDH-2014-00253	SRR1188542	2003	12	North America	United States	Enteritidis	Enteritidis	1425	14316
SAL_DA9008AA	MDH-2014-00208	SRR1188539	2000	9	North America	United States	Enteritidis	Enteritidis	1425	14314
SAL_DA9009AA	MDH-2014-00248	SRR1188538	2013	6	North America	United States	Enteritidis	Enteritidis	1425	14313
SAL_DA9010AA	MDH-2014-00217	SRR1188537	2001	4	North America	United States	Enteritidis	Enteritidis	1425	14312
SAL_DA9013AA	MDH-2014-00229	SRR1188534	2001	7	North America	United States	Enteritidis	Enteritidis	1425	14309
SAL_DA9014AA	MDH-2014-00223	SRR1188533	2001	5	North America	United States	Enteritidis	Enteritidis	1425	14306
SAL_DA9015AA	MDH-2014-00233	SRR1188526	2001	12	North America	United States	Enteritidis	Enteritidis	1425	14308
SAL_DA9016AA	MDH-2014-00212	SRR1188525	2000	9	North America	United States	Enteritidis	Enteritidis	1425	14307
SAL_DA9017AA	MDH-2014-00254	SRR1188519	2003	11	North America	United States	Enteritidis	Enteritidis	1425	14267
SAL_DA9018AA	MDH-2014-00228	SRR1188518	2001	7	North America	United States	Enteritidis	Enteritidis	1425	14275
SAL_DA9019AA	MDH-2014-00234	SRR1188510	2003	11	North America	United States	Enteritidis	Enteritidis	1425	14267
SAL_DA9020AA	MDH-2014-00222	SRR1188509	2001	5	North America	United States	Enteritidis	Enteritidis	1425	14306
SAL_DA9021AA	MDH-2014-00255	SRR1188508	2014	1	North America	United States	Enteritidis	Enteritidis	1425	14259
SAL_DA9022AA	MDH-2014-00209	SRR1188507	2000	9	North America	United States	Enteritidis	Enteritidis	1425	14269
SAL_DA9023AA	MDH-2014-00204	SRR1188500	2000	7	North America	United States	Enteritidis	Enteritidis	1425	14305
SAL_DA9024AA	MDH-2014-00226	SRR1188494	2001	6	North America	United States	Enteritidis	Enteritidis	1425	14304
SAL_DA9026AA	MDH-2014-00221	SRR1188485	2001	5	North America	United States	Enteritidis	Enteritidis	1425	14302
SAL_DA9027AA	MDH-2014-00218	SRR1188484	2001	5	North America	United States	Enteritidis	Enteritidis	1425	14301
SAL_DA9028AA	MDH-2014-00242	SRR1188483	2011	9	North America	United States	Enteritidis	Enteritidis	1425	14300
SAL_DA9029AA	MDH-2014-00219	SRR1188482	2001	5	North America	United States	Enteritidis	Enteritidis	1425	14275
SAL_DA9031AA	MDH-2014-00245	SRR1188480	2012	6	North America	United States	Enteritidis	Enteritidis	1425	14298
SAL_DA9032AA	MDH-2014-00241	SRR1188478	2011	9	North America	United States	Enteritidis	Enteritidis	1425	14297
SAL_DA9033AA	MDH-2014-00207	SRR1188477	2000	8	North America	United States	Enteritidis	Enteritidis	1425	14296
SAL_DA9034AA	MDH-2014-00206	SRR1188476	2000	8	North America	United States	Enteritidis	Enteritidis	1425	14295
SAL_DA9036AA	MDH-2014-00213	SRR1188474	2000	9	North America	United States	Enteritidis	Enteritidis	1425	14293
SAL_DA9038AA	MDH-2014-00235	SRR1188472	2005	9	North America	United States	Enteritidis	Enteritidis	1425	14291
SAL_DA9039AA	MDH-2014-00210	SRR1188471	2000	9	North America	United States	Enteritidis	Enteritidis	1425	14290
SAL_DA9040AA	MDH-2014-00203	SRR1188470	2000	7	North America	United States	Enteritidis	Enteritidis	1425	14289
SAL_DA9042AA	MDH-2014-00236	SRR1188468	2011	5	North America	United States	Enteritidis	Enteritidis	1425	14287
SAL_DA9043AA	MDH-2014-00230	SRR1188467	2001	7	North America	United States	Enteritidis	Enteritidis	1425	14286
SAL_DA9044AA	MDH-2014-00249	SRR1188466	2014	1	North America	United States	Enteritidis	Enteritidis	1425	14285
SAL_DA9045AA	MDH-2014-00202	SRR1188465	2000	6	North America	United States	Enteritidis	Enteritidis	1425	14284
SAL_DA9046AA	MDH-2014-00247	SRR1188464	2012	7	North America	United States	Enteritidis	Enteritidis	1425	14283
SAL_DA9047AA	MDH-2014-00237	SRR1188463	2011	6	North America	United States	Enteritidis	Enteritidis	1425	14282
SAL_DA9048AA	MDH-2014-00244	SRR1188462	2011	10	North America	United States	Enteritidis	Enteritidis	1425	14281
SAL_DA9049AA	MDH-2014-00220	SRR1188461	2001	5	North America	United States	Enteritidis	Enteritidis	1425	14275
SAL_DA9051AA	MDH-2014-00214	SRR1188458	2001	3	North America	United States	Enteritidis	Enteritidis	26543	14279
SAL_DA9053AA	MDH-2014-00250	SRR1188456	2014	1	North America	United States	Enteritidis	Enteritidis	1425	14277
SAL_DA9054AA	MDH-2014-00240	SRR1188455	2011	8	North America	United States	Enteritidis	Enteritidis	1425	14276
SAL_DA9055AA	MDH-2014-00225	SRR1188454	2001	6	North America	United States	Enteritidis	Enteritidis	1425	14275
SAL_DA9056AA	MDH-2014-00215	SRR1188453	2001	4	North America	United States	Enteritidis	Enteritidis	1425	14274
SAL_DA9057AA	MDH-2014-00205	SRR1188452	2000	8	North America	United States	Enteritidis	Enteritidis	1425	14273
SAL_DA9062AA	MDH-2014-00211	SRR1188445	2000	9	North America	United States	Enteritidis	Enteritidis	1425	14269
SAL_DA9064AA	MDH-2014-00252	SRR1188443	2003	11	North America	United States	Enteritidis	Enteritidis	1425	14267
SAL_DA9065AA	MDH-2014-00216	SRR1188442	2001	4	North America	United States	Enteritidis	Enteritidis	1425	14266
SAL_DA9066AA	MDH-2014-00238	SRR1188441	2011	8	North America	United States	Enteritidis	Enteritidis	1425	14265
SAL_DA9067AA	MDH-2014-00231	SRR1188440	2001	6	North America	United States	Enteritidis	Enteritidis	1425	14264
SAL_DA9068AA	MDH-2014-00232	SRR1188438	2001	10	North America	United States	Enteritidis	Enteritidis	1425	14263
SAL_DA9070AA	MDH-2014-00251	SRR1188436	2001	7	North America	United States	Enteritidis	Enteritidis	1425	14261
SAL_DA9071AA	MDH-2014-00243	SRR1188435	2011	10	North America	United States	Enteritidis	Enteritidis	1425	14260
SAL_DA9072AA	MDH-2014-00256	SRR1188434	2014	2	North America	United States	Enteritidis	Enteritidis	1425	14259
SAL_DA9073AA	MDH-2014-00239	SRR1188433	2011	8	North America	United States	Enteritidis	Enteritidis	1425	14258

SAL_DA9074AA	MDH-2014-00224	SRR1188432	2001	6	North America	United States	Enteritidis	Enteritidis	1425	14257
SAL_DA9077AA	AZ_TG71071	SRR1186369	2013	7	North America	United States	Enteritidis	Enteritidis	27721	14254
SAL_DA9086AA	AZ_TG71063	SRR1186340	2013	7	North America	United States	Enteritidis	Enteritidis	1425	14247
SAL_DA9093AA	AZ_TG71051	SRR1186320	2013	7	North America	United States	Enteritidis	Enteritidis	1425	14241
SAL_DA9097AA	MDH-2014-00197	SRR1186219	2011	6	North America	United States	Enteritidis	Enteritidis	1425	14238
SAL_DA9135AA	NY_IDR1200027330	SRR1185800	2012	8	North America	United States	Enteritidis	Enteritidis	1425	14207
SAL_DA9149AA	EC20110222	GCF_000624275	2005		North America	Canada	Enteritidis	Enteritidis	1425	58327
SAL_DA9150AA	SA19992322	SRR1183745	1998		North America	Canada	Enteritidis	Enteritidis	1425	14193
SAL_DA9152AA	EC20120008	SRR1183999	2006		North America	Canada	Enteritidis	Enteritidis	1425	14191
SAL_DA9153AA	EC20120007	SRR1183998	2011		North America	Canada	Enteritidis	Enteritidis	1425	58208
SAL_DA9154AA	EC20120005	GCF_000623195	2011		North America	Canada	Enteritidis	Enteritidis	1425	14190
SAL_DA9155AA	EC20120003	GCF_000624415	2005		North America	Canada	Enteritidis	Enteritidis	1425	58207
SAL_DA9156AA	EC20120002	SRR1183995	2010		North America	Canada	Enteritidis	Enteritidis	1425	14188
SAL_DA9157AA	EC20100134	SRR1183994	2009		North America	Canada	Enteritidis (Predicted)	Enteritidis	1425	14187
SAL_DA9158AA	EC20100130	GCF_000831045	2009		North America	Canada	Enteritidis (Predicted)	Enteritidis	1425	14186
SAL_DA9159AA	EC20090884	SRR1183992	2009		North America	Canada	Enteritidis	Enteritidis	1425	14185
SAL_DA9160AA	EC20090531	SRR1183991	2009		North America	Canada	Enteritidis	Enteritidis	1425	14184
SAL_DA9161AA	EC20090332	SRR1183758	2008		North America	Canada	Enteritidis	Enteritidis	1425	14183
SAL_DA9162AA	EC20090135	SRR1183989	2008		North America	Canada	Enteritidis	Enteritidis	1425	14182
SAL_DA9163AA	EC20090193	SRR1183988	2008		North America	Canada	Enteritidis	Enteritidis	1425	14181
SAL_DA9164AA	EC20100103	SRR1183987	2009		North America	Canada	Enteritidis	Enteritidis	1425	14180
SAL_DA9167AA	EC20111175	SRR1183752	2011		North America	Canada	Enteritidis	Enteritidis	1425	14177
SAL_DA9168AA	EC20110221	GCF_000626235	2005		North America	Canada	Enteritidis	Enteritidis	1425	14176
SAL_DA9170AA	EC20090641	SRR1183981	2008		North America	Canada	Enteritidis	Enteritidis	1425	14174
SAL_DA9172AA	EC20110353	SRR1183979	2010		North America	Canada	Enteritidis	Enteritidis	-2008	14172
SAL_DA9173AA	EC20111095	SRR1183744	2011		North America	Canada	Enteritidis	Enteritidis	1425	14136
SAL_DA9175AA	EC20110360	GCF_000623295	2004		North America	Canada	Enteritidis	Enteritidis	1425	14170
SAL_DA9176AA	EC20110223	SRR1183975	2005		North America	Canada	Enteritidis	Enteritidis	1425	14169
SAL_DA9177AA	EC20110359	SRR1183974	2004		North America	Canada	Enteritidis	Enteritidis	1425	14168
SAL_DA9178AA	EC20110358	SRR1183973	2009		North America	Canada	Enteritidis	Enteritidis	27446	14167
SAL_DA9179AA	EC20110357	GCF_000623355	2003		North America	Canada	Enteritidis	Enteritidis	27707	14166
SAL_DA9181AA	EC20110354	SRR1183970	2010		North America	Canada	Enteritidis	Enteritidis	1425	14164
SAL_DA9182AA	EC20120969	GCF_000625215	2012		North America	Canada	Enteritidis	Enteritidis	1425	58265
SAL_DA9183AA	EC20120929	SRR1183915	2012		North America	Canada	Enteritidis	Enteritidis	1425	14162
SAL_DA9184AA	EC20120927	GCF_000625155	2012		North America	Canada	Enteritidis	Enteritidis	1425	58267
SAL_DA9185AA	EC20121689	GCF_000624795	2012		North America	Canada	Enteritidis	Enteritidis	1425	58274
SAL_DA9186AA	EC20120925	GCF_000625135	2012		North America	Canada	Enteritidis	Enteritidis	1425	58277
SAL_DA9187AA	EC20120918	GCF_000625115	2012		North America	Canada	Enteritidis	Enteritidis	1425	58217
SAL_DA9188AA	EC20120917	GCF_000625095	2012		North America	Canada	Enteritidis	Enteritidis	1425	58218
SAL_DA9189AA	EC20120774	GCF_000625035	2012		North America	Canada	Enteritidis	Enteritidis	1425	58269
SAL_DA9190AA	EC20120773	GCF_000625015	2012		North America	Canada	Enteritidis	Enteritidis	1425	58220
SAL_DA9191AA	EC20120738	GCF_000624975	2012		North America	Canada	Enteritidis	Enteritidis	1425	58272
SAL_DA9192AA	EC20120734	GCF_000624955	2012		North America	Canada	Enteritidis	Enteritidis	1425	58222
SAL_DA9193AA	EC20120687	GCF_000625575	2011		North America	Canada	Enteritidis	Enteritidis	1425	58213
SAL_DA9194AA	EC20120686	GCF_000625555	2011		North America	Canada	Enteritidis	Enteritidis	1425	58264
SAL_DA9195AA	EC20120597	GCF_000625515	2011		North America	Canada	Enteritidis	Enteritidis	1425	58278
SAL_DA9196AA	EC20120590	GCF_000625495	2011		North America	Canada	Enteritidis	Enteritidis	26751	58201
SAL_DA9197AA	EC20120581	GCF_000625475	2011				Enteritidis	Enteritidis	1425	58261
SAL_DA9198AA	EC20120580	GCF_000625455	2011				Enteritidis	Enteritidis	1425	58271
SAL_DA9199AA	EC20120356	GCF_000624535	2011		North America	Canada	Enteritidis	Enteritidis	27703	58240
SAL_DA9200AA	EC20120051	GCF_000625835	2011		North America	Canada	Enteritidis	Enteritidis	27712	58310
SAL_DA9201AA	EC20100325	SRR1183897	2010		North America	Canada	Enteritidis	Enteritidis	27700	14144
SAL_DA9202AA	EC20100089	GCF_000625435	2009		North America	Canada	Enteritidis	Enteritidis	1425	58230
SAL_DA9203AA	EC20100088	GCF_000625415	2009		North America	Canada	Enteritidis	Enteritidis	1425	58231
SAL_DA9204AA	EC20121180	SRR1183894			North America	Canada	Enteritidis	Enteritidis	1425	14141
SAL_DA9205AA	EC20121179	GCF_000623115			North America	Canada	Enteritidis	Enteritidis	-1697	14140
SAL_DA9206AA	EC20121178	SRR1183892			North America	Canada	Enteritidis	Enteritidis	1425	14139
SAL_DA9207AA	EC20121177	GCA_000624175			North America	Canada	Enteritidis	Enteritidis	1425	14138
SAL_DA9209AA	EC20121175	SRR1183889			North America	Canada	Enteritidis	Enteritidis	1425	14136

SAL_DA9210AA	EC20130348	GCF_000626075	2010	North America	Canada	Enteritidis	Enteritidis	27446	58256
SAL_DA9211AA	EC20130347	GCF_000626055	2010	North America	Canada	Enteritidis	Enteritidis	1425	58259
SAL_DA9212AA	EC20130346	SRR1183886	2010	North America	Canada	Enteritidis	Enteritidis	1425	58252
SAL_DA9213AA	EC20130345	GCF_000626015	2008	North America	Canada	Enteritidis	Enteritidis	1425	58253
SAL_DA9214AA	EC20090195	SRR1183884	2008	North America	Canada	Enteritidis	Enteritidis	1425	58318
SAL_DA9215AA	EC20100100	GCF_000968775	2009	North America	Canada	Enteritidis (Predicted)	Enteritidis	1425	58306
SAL_DA9216AA	EC20090530	GCF_000625395	2009	North America	Canada	Enteritidis	Enteritidis	1425	58232
SAL_DA9217AA	EC20100131	GCF_000625715	2009	North America	Canada	Enteritidis	Enteritidis	1425	58316
SAL_DA9218AA	EC20121969	SRR1183880	2012	North America	Canada	Enteritidis	Enteritidis	1425	58215
SAL_DA9219AA	EC20121970	GCF_000625335	2012	North America	Canada	Enteritidis	Enteritidis	1425	58212
SAL_DA9220AA	EC20121976	SRR1183878	2012	North America	Canada	Enteritidis	Enteritidis	1425	58211
SAL_DA9221AA	EC20121986	GCF_000625795	2012	North America	Canada	Enteritidis	Enteritidis	1425	58210
SAL_DA9222AA	EC20121989	SRR1183876	2012	North America	Canada	Enteritidis	Enteritidis	1425	58209
SAL_DA9223AA	EC20121990	GCF_000625875	2012	North America	Canada	Enteritidis	Enteritidis	1425	58229
SAL_DA9224AA	EC20122022	GCF_000625895	2012	North America	Canada	Enteritidis	Enteritidis	1425	58321
SAL_DA9225AA	EC20122026	GCF_000625915	2012	North America	Canada	Enteritidis	Enteritidis	1425	58228
SAL_DA9226AA	EC20122031	SRR1183872	2012	North America	Canada	Enteritidis	Enteritidis	1425	58227
SAL_DA9227AA	EC20122033	GCF_000625955	2012	North America	Canada	Enteritidis	Enteritidis	1425	58255
SAL_DA9228AA	EC20122045	SRR1183870	2012	North America	Canada	Enteritidis	Enteritidis	1425	58254
SAL_DA9229AA	EC20111561	GCF_000624355	2010	North America	Canada	Enteritidis	Enteritidis	1425	58206
SAL_DA9230AA	EC20121812	SRR1183868	2011	North America	Canada	Enteritidis	Enteritidis	26641	58263
SAL_DA9231AA	EC20120916	SRR1183867	2012	North America	Canada	Enteritidis	Enteritidis	-1733	14114
SAL_DA9232AA	EC20120994	GCA_000624675	2012	North America	Canada	Enteritidis	Enteritidis	1425	58182
SAL_DA9233AA	EC20120497	GCF_000624595	2010	North America	Canada	Enteritidis	Enteritidis	1425	58237
SAL_DA9234AA	EC20111554	GCF_000624335	2010	North America	Canada	Enteritidis	Enteritidis	1425	58285
SAL_DA9235AA	EC20120555	GCF_000623255	2010	North America	Canada	Enteritidis	Enteritidis	1425	58308
SAL_DA9236AA	EC20121671	GCF_000624755	2012	North America	Canada	Enteritidis	Enteritidis	1425	58279
SAL_DA9237AA	EC20120765	GCF_000624995	2011	North America	Canada	Enteritidis	Enteritidis	1425	58221
SAL_DA9238AA	EC20121746	GCF_000624855	2011	North America	Canada	Enteritidis	Enteritidis	1425	58287
SAL_DA9240AA	EC20121672	GCF_000624775	2012	North America	Canada	Enteritidis	Enteritidis	1425	58276
SAL_DA9241AA	EC20121748	GCF_000624875	2011	North America	Canada	Enteritidis	Enteritidis	1425	58225
SAL_DA9242AA	EC20120677	SRR1183856	2011	North America	Canada	Enteritidis	Enteritidis	1425	58313
SAL_DA9243AA	EC20120968	GCF_000625195	2012	North America	Canada	Enteritidis	Enteritidis	1425	58325
SAL_DA9244AA	EC20121542	SRR1183854	2012	North America	Canada	Enteritidis	Enteritidis	1425	58291
SAL_DA9245AA	EC20120496	GCF_000624575	2010	North America	Canada	Enteritidis	Enteritidis	1425	58238
SAL_DA9246AA	EC20121765	SRR1183852	2011	North America	Canada	Enteritidis	Enteritidis	27446	58319
SAL_DA9247AA	EC20121744	GCF_000624835	2011	North America	Canada	Enteritidis	Enteritidis	1425	58273
SAL_DA9248AA	EC20120697	GCA_000625595	2011			Enteritidis	Enteritidis	1425	58183
SAL_DA9249AA	EC20120469	GCF_000624555	2010	North America	Canada	Enteritidis	Enteritidis	1425	58239
SAL_DA9250AA	EC20121753	GCF_000624935	2011	North America	Canada	Enteritidis	Enteritidis	1425	58223
SAL_DA9251AA	EC20120722	GCF_000625615	2012	North America	Canada	Enteritidis	Enteritidis	1425	58214
SAL_DA9252AA	EC20111510	GCF_000626555	2010	North America	Canada	Enteritidis	Enteritidis	1425	58311
SAL_DA9253AA	EC20120219	GCF_000624475	2010	North America	Canada	Enteritidis	Enteritidis	1425	58283
SAL_DA9254AA	EC20120229	GCF_000624495	2010	North America	Canada	Enteritidis	Enteritidis	1425	58282
SAL_DA9255AA	EC20120240	GCF_000624515	2010	North America	Canada	Enteritidis	Enteritidis	1425	58241
SAL_DA9256AA	EC20120498	GCF_000624615	2010	North America	Canada	Enteritidis	Enteritidis	1425	58236
SAL_DA9257AA	EC20120505	GCF_000624635	2010	North America	Canada	Enteritidis	Enteritidis	1425	58235
SAL_DA9258AA	EC20120548	GCF_000623235	2010	North America	Canada	Enteritidis	Enteritidis	1425	58301
SAL_DA9259AA	EC20120970	GCF_000625235	2012	North America	Canada	Enteritidis	Enteritidis	1425	58275
SAL_DA9260AA	EC20121750	GCF_000624895	2011	North America	Canada	Enteritidis	Enteritidis	1425	58270
SAL_DA9261AA	EC20121751	GCF_000624915	2011	North America	Canada	Enteritidis	Enteritidis	1425	58224
SAL_DA9262AA	EC20111514	GCF_000624295	2010	North America	Canada	Enteritidis	Enteritidis	1425	58288
SAL_DA9263AA	EC20111515	GCF_000624315	2010	North America	Canada	Enteritidis	Enteritidis	1425	58326
SAL_DA9264AA	EC20120528	GCF_000624655	2010	North America	Canada	Enteritidis	Enteritidis	1425	58281
SAL_DA9265AA	EC20120544	GCF_000623215	2010	North America	Canada	Enteritidis	Enteritidis	1425	58302
SAL_DA9266AA	EC20111576	GCF_000624375	2010	North America	Canada	Enteritidis	Enteritidis	1425	58284
SAL_DA9267AA	EC20120685	SRR1183831	2011	North America	Canada	Enteritidis	Enteritidis	1425	58324
SAL_DA9268AA	EC20120775	GCF_000625055	2012	North America	Canada	Enteritidis	Enteritidis	1425	58268
SAL_DA9269AA	EC20120776	SRR1183829	2012	North America	Canada	Enteritidis	Enteritidis	1425	58219

SAL_DA9270AA	EC20120963	GCF_000625175	2012	North America	Canada	Enteritidis	Enteritidis	1425	58266
SAL_DA9271AA	EC20121004	GCF_000624695	2012	North America	Canada	Enteritidis	Enteritidis	1425	58286
SAL_DA9272AA	EC20120200	SRR1183826	2010	North America	Canada	Enteritidis	Enteritidis	26645	14074
SAL_DA9273AA	EC20120213	GCF_000624455	2010	North America	Canada	Enteritidis	Enteritidis	1425	58303
SAL_DA9274AA	EC20121826	GCF_000625295	2011	North America	Canada	Enteritidis	Enteritidis	1425	58216
SAL_DA9275AA	EC20121825	GCF_000625275	2011	North America	Canada	Enteritidis	Enteritidis	1425	58262
SAL_DA9276AA	EC20121541	SRR1183822	2012	North America	Canada	Enteritidis	Enteritidis	1425	58280
SAL_DA9277AA	SA20094079	GCF_000624015	2009	North America	Canada	Enteritidis	Enteritidis	1425	14069
SAL_DA9278AA	SA20091739	GCF_000623835	2009	North America	Canada	Enteritidis	Enteritidis	1425	58242
SAL_DA9279AA	SA20093538	GCF_000623895	2009	North America	Canada	Enteritidis	Enteritidis	1425	58307
SAL_DA9280AA	SA20090877	SRR1183818	2009	North America	Canada	Enteritidis	Enteritidis	49194	58243
SAL_DA9281AA	SA20093543	GCF_000623915	2009	North America	Canada	Enteritidis	Enteritidis	1425	58315
SAL_DA9282AA	SA20093950	SRR1183816	2009	North America	Canada	Enteritidis	Enteritidis	1425	58297
SAL_DA9283AA	SA20094642	GCF_000624135	2009	North America	Canada	Enteritidis	Enteritidis	1425	58203
SAL_DA9284AA	SA20090419	GCA_000625355	2008	North America	Canada	Enteritidis	Enteritidis	1425	58234
SAL_DA9285AA	SA20094383	GCF_000624075	2009	North America	Canada	Enteritidis	Enteritidis	26744	58298
SAL_DA9286AA	SA20094389	GCF_000624095	2009	North America	Canada	Enteritidis	Enteritidis	1425	58292
SAL_DA9287AA	SA20093421	GCF_000623855	2009	North America	Canada	Enteritidis	Enteritidis	1425	58299
SAL_DA9288AA	SA20090435	GCF_000625375	2008	North America	Canada	Enteritidis	Enteritidis	-11716	58233
SAL_DA9289AA	SA20093430	GCF_000623875	2009	North America	Canada	Enteritidis	Enteritidis	1425	58300
SAL_DA9290AA	SA20093788	GCF_000623955	2009	North America	Canada	Enteritidis	Enteritidis	1425	58295
SAL_DA9291AA	SA20093977	GCF_000623995	2009	North America	Canada	Enteritidis	Enteritidis	27686	58294
SAL_DA9292AA	SA20093784	GCF_000623935	2009	North America	Canada	Enteritidis	Enteritidis	27689	58296
SAL_DA9293AA	SA20092320	GCF_000624195	2009			Enteritidis	Enteritidis	49195	58290
SAL_DA9294AA	SA20095309	GCF_000624235	2009	North America	Canada	Enteritidis	Enteritidis	1425	58205
SAL_DA9295AA	SA20083456	GCF_000623495	2008	North America	Canada	Enteritidis	Enteritidis	1425	58250
SAL_DA9297AA	SA20094350	GCF_000624035	2009	North America	Canada	Enteritidis	Enteritidis	1425	58293
SAL_DA9298AA	SA20094803	GCF_000624215	2009	North America	Canada	Enteritidis	Enteritidis	1425	58204
SAL_DA9299AA	SA19982831	GCF_000623415	1998	North America	Canada	Enteritidis	Enteritidis	1425	58257
SAL_DA9301AA	SA19930684	GCF_000623555	1993	North America	Canada	Enteritidis	Enteritidis	1425	58248
SAL_DA9302AA	SA19994216	GCA_000623475	1999	North America	Canada	Enteritidis	Enteritidis	1425	14045
SAL_DA9303AA	SA19983126	GCF_000623435	1998	North America	Canada	Enteritidis	Enteritidis	1425	58260
SAL_DA9304AA	SA19961622	GCF_000623635	1996	North America	Canada	Enteritidis	Enteritidis	1425	58245
SAL_DA9305AA	SA19971331	SRR1183793	1997	North America	Canada	Enteritidis	Enteritidis	1425	58244
SAL_DA9306AA	SA20123395	GCF_000625995	2012	North America	Canada	Enteritidis	Enteritidis	1425	58258
SAL_DA9307AA	SA19943269	SRR1183791	1994	North America	Canada	Enteritidis	Enteritidis	1425	58246
SAL_DA9308AA	SA19942384	GCF_000623575	1994	North America	Canada	Enteritidis	Enteritidis	1425	58247
SAL_DA9309AA	SA20095440	GCF_000624255	2009	North America	Canada	Enteritidis	Enteritidis	1425	58181
SAL_DA9310AA	SA20094352	SRR1183788	2009	North America	Canada	Enteritidis	Enteritidis	1425	14038
SAL_DA9311AA	SA20083636	GCF_000623515	2008	North America	Canada	Enteritidis	Enteritidis	27675	58251
SAL_DA9312AA	SA20085285	SRR1183786	2008	North America	Canada	Enteritidis	Enteritidis	27675	58184
SAL_DA9313AA	SA20082034	GCA_000625855	2008	North America	Canada	Enteritidis	Enteritidis	27675	14036
SAL_DA9314AA	SA19960848	SRR1183784	1996	North America	Canada	Enteritidis	Enteritidis	1425	14035
SAL_DA9315AA	SA19981857	SRR1183783	1998	North America	Canada	Enteritidis	Enteritidis	1425	14034
SAL_DA9316AA	SA19981522	SRR1183782	1998	North America	Canada	Enteritidis	Enteritidis	1425	14033
SAL_DA9317AA	SA20094521	GCF_000624115	2009	North America	Canada	Enteritidis	Enteritidis	49194	58202
SAL_DA9318AA	SA20121703	GCF_000624815	2012	North America	Canada	Enteritidis	Enteritidis	1425	58289
SAL_DA9319AA	SA20100349	GCA_000968795	2010	North America	Canada	Enteritidis (Predicted)	Enteritidis	1425	58187
SAL_DA9320AA	SA20100239	GCF_000625735	2010	North America	Canada	Enteritidis	Enteritidis	1425	58314
SAL_DA9321AA	SA20084644	GCF_000626475	2008	North America	Canada	Enteritidis	Enteritidis	1425	58305
SAL_DA9324AA	SA20084384	SRR1183774	2008	North America	Canada	Enteritidis	Enteritidis	1425	58249
SAL_DA9325AA	SA20094682	GCF_000625695	2009	North America	Canada	Enteritidis	Enteritidis	1425	58317
SAL_DA9326AA	SA20093266	SRR1183772	2009	North America	Canada	Enteritidis	Enteritidis	1425	14023
SAL_DA9327AA	SA19970769	SRR1183771	1997	North America	Canada	Enteritidis	Enteritidis	1425	14022
SAL_DA9328AA	SA19970510	GCF_000623655	1997	North America	Canada	Enteritidis	Enteritidis	1425	14021
SAL_DA9329AA	SA19980677	SRR1183769	1998	North America	Canada	Enteritidis	Enteritidis	1425	14020
SAL_DA9365AA	WAPHL-SAL-A00228	SRR1181547	2002	North America	United States	Enteritidis	Enteritidis	1425	13988
SAL_DA9373AA	WAPHL-SAL-A00221	SRR1181526	2002	North America	United States	Enteritidis	Enteritidis	1425	13981
SAL_DA9374AA	WAPHL-SAL-A00216	SRR1181525	2002	North America	United States	Enteritidis	Enteritidis	1425	13981

SAL_DA9377AA	WAPHL-SAL-A00237	SRR1181520	2002		North America	United States	Enteritidis	Enteritidis	1425	13978
SAL_DA9434AA	WAPHL_SAL-A00196	SRR1177580	2004		North America	United States	Enteritidis	Enteritidis	1425	13840
SAL_DA9444AA	WAPHL_SAL-A00198	SRR1177566	2004		North America	United States	Enteritidis	Enteritidis	1425	13840
SAL_DA9445AA	WAPHL_SAL-A00194	SRR1177565	2010		North America	United States	Enteritidis	Enteritidis	1425	13919
SAL_DA9467AA	WAPHL_SAL-A00173	SRR1177367	2012	1	North America	United States	Enteritidis	Enteritidis	1425	13899
SAL_DA9491AA	WAPHL_SAL-A00195	SRR1177301	2004		North America	United States	Enteritidis	Enteritidis	1425	13840
SAL_DA9532AA	WAPHL_SAL-A00199	SRR1177234	2004		North America	United States	Enteritidis	Enteritidis	1425	13840
SAL_DA9540AA	NY_IDR1000024945X1	SRR1177214	2010	7	North America	United States	Typhimurium var. O5 - (Copenhagen)	Enteritidis	1425	13832
SAL_DA9558AA	WAPHL_SAL-A00200	SRR1177180	2004		North America	United States	Enteritidis	Enteritidis	1425	13814
SAL_DA9623AA	FNW19G90	SRR1175785	2010	7	North America	United States	Enteritidis	Enteritidis	1425	13766
SAL_DA9625AA	FNW19G89	SRR1175782	2010	7	North America	United States	Enteritidis	Enteritidis	1425	13765
SAL_DA9651AA	MDH-2013-00173	SRR1170901	2012	8	North America	United States	Enteritidis	Enteritidis	1425	13741
SAL_DA9734AA	MDH-2013-00075	SRR1164605	2009	4	North America	United States	Enteritidis	Enteritidis	1425	13664
SAL_DA9773AA	MDH-2013-00050	SRR1164562	2008	9	North America	United States	Enteritidis	Enteritidis	1425	13628
SAL_DA9819AA	WAPHL_SAL-A00189	SRR1158158	2003		North America	United States	Enteritidis	Enteritidis	1425	13584
SAL_DA9828AA	WAPHL_SAL-A00187	SRR1158135	2006		North America	United States	Enteritidis	Enteritidis	1425	13575
SAL_DA9859AA	WAPHL_SAL-A00184	SRR1158049	2005		North America	United States	Enteritidis	Enteritidis	1425	13545
SAL_DA9860AA	NY_BAC0900005834	SRR1158048	2009	12	North America	United States	I 9,12:-:-	Enteritidis	1425	13544
SAL_DA9881AA	WAPHL_SAL-A00193	SRR1157978	2008		North America	United States	Enteritidis	Enteritidis	1425	13527
SAL_DA9885AA	WAPHL_SAL-A00188	SRR1157967	2006		North America	United States	Enteritidis	Enteritidis	1425	13523
SAL_DA9891AA	WAPHL_SAL-A00191	SRR1157942	1986		North America	United States	Enteritidis	Enteritidis	1425	13517
SAL_DA9893AA	NY_BAC0900001112	SRR1157940	2009	3	North America	United States	Enteritidis	Enteritidis	1425	5905
SAL_DA9901AA	MDH-2013-00106	SRR1157899	2010	1	North America	United States	Enteritidis	Enteritidis	1425	13509
SAL_DA9902AA	WAPHL_SAL-A00185	SRR1157898	2004		North America	United States	Enteritidis	Enteritidis	1425	13508
SAL_DA9910AA	WAPHL_SAL-A00192	SRR1157879	2003		North America	United States	Enteritidis	Enteritidis	1425	13502
SAL_DA9915AA	FSF0075	SRR1157867	2011	7	Asia	China	Enteritidis	Enteritidis	1425	13499
SAL_DA9920AA	MDH-2013-00077	SRR1157840	2009	4	North America	United States	Enteritidis	Enteritidis	1425	13495
SAL_DA9933AA	MDH-2013-00090	SRR1157605	2009	8	North America	United States	Enteritidis	Enteritidis	1425	13484
SAL_DA9970AA	WAPHL_SAL-A00174	SRR1141178	2012	1	North America	United States	Enteritidis	Enteritidis	1425	13454
SAL_DA9973AA	WAPHL_SAL-A00144	SRR1141171	2012	4	North America	United States	Enteritidis	Enteritidis	1425	13451
SAL_DA9978AA	WAPHL_SAL-A00147	SRR1141152	2012	4	North America	United States	Enteritidis	Enteritidis	1425	13447
SAL_DA9980AA	WAPHL_SAL-A00171	SRR1141135	2012	1	North America	United States	Enteritidis	Enteritidis	1425	13300
SAL_EA0013AA	FNE0001	SRR1121759	2012	5			Enteritidis (Predicted)	Enteritidis	1425	13417
SAL_EA0041AA	NY_IDR1100031092-9T1	SRR1107484	2011	10	North America	United States	Enteritidis	Enteritidis	1425	13401
SAL_EA0048AA	FNE0025	SRR1107477	2011	7	Asia	Hong Kong	Enteritidis	Enteritidis	1425	13397
SAL_EA0065AA	NY_IDR1200019824-2L	SRR1107460	2012	6	North America	United States	Enteritidis	Enteritidis	1425	13381
SAL_EA0084AA	WAPHL_SAL-A00160	SRR1106266	2011	11	North America	United States	Enteritidis	Enteritidis	1425	13363
SAL_EA0091AA	WAPHL_SAL-A00131	SRR1106259	2012	3	North America	United States	Enteritidis	Enteritidis	1425	13358
SAL_EA0093AA	WAPHL_SAL-A00138	SRR1106257	2012	4	North America	United States	Enteritidis	Enteritidis	1425	13356
SAL_EA0101AA	WAPHL_SAL-A00169	SRR1106249	2011	12	North America	United States	Enteritidis	Enteritidis	1425	13349
SAL_EA0105AA	WAPHL_SAL-A00148	SRR1106245	2012	4	North America	United States	Enteritidis	Enteritidis	1425	13345
SAL_EA0112AA	WAPHL_SAL-A00159	SRR1106237	2011	11	North America	United States	Enteritidis	Enteritidis	1425	13338
SAL_EA0115AA	NY_BAC0800006747	SRR1106234	2008	11	North America	United States	Enteritidis	Enteritidis	1425	13335
SAL_EA0116AA	WAPHL_SAL-A00123	SRR1106233	2012	3	North America	United States	Enteritidis	Enteritidis	1425	13334
SAL_EA0127AA	NY_IDR1000021807	SRR1106222	2010	6	North America	United States	Enteritidis	Enteritidis	1425	13324
SAL_EA0142AA	WAPHL_SAL-A00094	SRR1106207	2008	10	North America	United States	Enteritidis	Enteritidis	1425	13310
SAL_EA0152AA	WAPHL_SAL-A00112	SRR1106179	2012	11			Enteritidis	Enteritidis	1425	13300
SAL_EA0154AA	WAPHL_SAL-A00152	SRR1106177	2011	11	North America	United States	Enteritidis	Enteritidis	1425	13298
SAL_EA0157AA	WAPHL_SAL-A00156	SRR1106174	2011	11	North America	United States	Enteritidis	Enteritidis	1425	13295
SAL_EA0158AA	WAPHL_SAL-A00113	SRR1106173	2012	11			Enteritidis	Enteritidis	1425	13294
SAL_EA0159AA	WAPHL_SAL-A00161	SRR1106172	2011	11	North America	United States	Enteritidis	Enteritidis	1425	13293
SAL_EA0160AA	WAPHL_SAL-A00136	SRR1106171	2012	3	North America	United States	Enteritidis	Enteritidis	1425	13292
SAL_EA0162AA	WAPHL_SAL-A00163	SRR1106168	2011	12	North America	United States	Enteritidis	Enteritidis	1425	13290
SAL_EA0164AA	WAPHL_SAL-A00164	SRR1106166	2011	12	North America	United States	Enteritidis	Enteritidis	1425	13288
SAL_EA0268AA	FSW0074	SRR1068360	2010	11	Asia	Indonesia	Enteritidis	Enteritidis	1425	13196
SAL_EA0324AA	NY_02022200	SRR1067669	2004	8	North America	United States	Enteritidis	Enteritidis	1425	12432
SAL_EA0328AA	NY_02035028	SRR1067665	2009	11	North America	United States	Enteritidis	Enteritidis	1425	13143
SAL_EA0338AA	NY_02037914	SRR1067655	2011	2	North America	United States	Enteritidis	Enteritidis	1425	13135
SAL_EA0349AA	NY_02034766	SRR1067644	2009	9	North America	United States	Enteritidis	Enteritidis	1425	13126

SAL_EA0353AA	MDH-2013-00168	SRR1067640	2012	6	North America	United States	Enteritidis	Enteritidis	1425	13122
SAL_EA0367AA	NY_02037913	SRR1067626	2011	2	North America	United States	Enteritidis	Enteritidis	1425	13109
SAL_EA0392AA	FCC0062	SRR1055756	2010	5	North America	United States	Enteritidis	Enteritidis	1425	13087
SAL_EA0401AA	FCC0063	SRR1055747	2010	5	North America	United States	Enteritidis	Enteritidis	1425	13078
SAL_EA0448AA	NY_02034452	SRR1048627	2009	7	North America	United States	Enteritidis	Enteritidis	1425	12432
SAL_EA0449AA	MDH-2013-00179	SRR1048623	2012	9	North America	United States	Enteritidis	Enteritidis	1425	13034
SAL_EA0457AA	NY_02034528	SRR1048327	2009	7	North America	United States	Enteritidis	Enteritidis	1425	13027
SAL_EA0470AA	MDH-2013-00188	SRR1048308	2013	2	North America	United States	Enteritidis	Enteritidis	1425	13017
SAL_EA0488AA	FCC0051	SRR1048275	2010	6	North America	United States	Enteritidis	Enteritidis	1425	13002
SAL_EA0494AA	FCC0050	SRR1048253	2010	6	North America	United States	Enteritidis	Enteritidis	27809	12996
SAL_EA0496AA	FSF0037	SRR1048249	2010	6	Asia	India	Enteritidis	Enteritidis	1425	12994
SAL_EA0541AA	NY_02034201	SRR1043653	2009	6	North America	United States	Enteritidis	Enteritidis	1425	12956
SAL_EA0562AA	FCC0033	SRR1041529	2010	8	North America	United States	Enteritidis	Enteritidis	1425	12942
SAL_EA0577AA	NY_02033982	SRR1041497	2009	4	North America	United States	Enteritidis	Enteritidis	1425	12903
SAL_EA0590AA	NY_02033961	SRR1041473	2009	4	North America	United States	Enteritidis	Enteritidis	1425	12917
SAL_EA0593AA	FCC0035	SRR1041466	2010	8	Asia	Philippines	Essen	Enteritidis	27796	12914
SAL_EA0605AA	NY_02033707	SRR1036455	2009	2	North America	United States	Enteritidis	Enteritidis	1425	12903
SAL_EA0606AA	NY_02033580	SRR1036454	2009	1	North America	United States	Enteritidis	Enteritidis	1425	12902
SAL_EA0607AA	NY_02033389	SRR1036453	2008	11	North America	United States	Enteritidis	Enteritidis	1425	12901
SAL_EA0608AA	NY_02033071	SRR1036452	2008	9	North America	United States	Enteritidis	Enteritidis	1425	12900
SAL_EA0609AA	NY_02033861	SRR1036451	2009	3	North America	United States	Enteritidis	Enteritidis	1425	12899
SAL_EA0610AA	NY_02033339	SRR1036450	2008	11	North America	United States	Enteritidis	Enteritidis	1425	12432
SAL_EA0613AA	NY_02033826	SRR1036446	2009	3	North America	United States	Enteritidis	Enteritidis	1425	12897
SAL_EA0614AA	NY_02033388	SRR1036445	2008	11	North America	United States	Enteritidis	Enteritidis	1425	12432
SAL_EA0624AA	NY_02033111	SRR1036433	2008	9	North America	United States	Enteritidis	Enteritidis	1425	12890
SAL_EA0703AA	FAR0001	SRR1033533	2010	11	North America	United States	Enteritidis	Enteritidis	1425	12822
SAL_EA0746AA	FSW0023	SRR1033488	2011	9	Asia	China	Enteritidis (Predicted)	Enteritidis	1425	12788
SAL_EA0755AA	swgs1009	SRR1033476	2013	9	North America	United States	Enteritidis	Enteritidis	1425	12491
SAL_EA0761AA	NY_02022468	SRR1033470	2004	9	North America	United States	Enteritidis	Enteritidis	1425	12777
SAL_EA0797AA	NY_02032083	SRR1030366	2008	4	North America	United States	Enteritidis	Enteritidis	1425	12432
SAL_EA0800AA	NY_02031589	SRR1030363	2008	2	North America	United States	Enteritidis	Enteritidis	1425	12432
SAL_EA0802AA	NY_02031374	SRR1030361	2008	1	North America	United States	Enteritidis	Enteritidis	1425	12748
SAL_EA0812AA	NY_02032082	SRR1030350	2008	5	North America	United States	Enteritidis	Enteritidis	1425	12738
SAL_EA0878AA	SAEN19-sc-2013-02-27T10:2	ERR311285					Enteritidis	Enteritidis	1424	41379
SAL_EA0916AA	SAEN437-sc-2013-02-27T10:	ERR311305					Enteritidis	Enteritidis	1425	12676
SAL_EA0932AA	SAEN84-sc-2013-02-27T10:2	ERR311289					Enteritidis	Enteritidis	1424	41076
SAL_EA0942AA	ASERH121644-sc-2013-02-2	ERR304840					Enteritidis	Enteritidis	3906	12667
SAL_EA0948AA	ASERH120656-sc-2013-02-2	ERR304834					Enteritidis (Predicted)	Enteritidis	3888	12619
SAL_EA0949AA	ASERH121498-sc-2013-02-2	ERR304833					Enteritidis	Enteritidis	3888	12619
SAL_EA0962AA	ASERH116267-sc-2013-02-2	ERR304820					Enteritidis	Enteritidis	1425	12654
SAL_EA0974AA	ASERH111712-sc-2013-02-2	ERR304808					Enteritidis (Predicted)	Enteritidis	1425	12645
SAL_EA0975AA	ASERH117224-sc-2013-02-2	ERR304807					Enteritidis (Predicted)	Enteritidis	1425	12645
SAL_EA0976AA	ASERH110579-sc-2013-02-2	ERR304806					Enteritidis	Enteritidis	1425	12644
SAL_EA0979AA	ASERH112876-sc-2013-02-2	ERR304803					Enteritidis	Enteritidis	1425	12642
SAL_EA0984AA	ASERH114386-sc-2013-02-2	ERR304871					Enteritidis (Predicted)	Enteritidis	1425	12638
SAL_EA0988AA	ASERH120153-sc-2013-02-2	ERR304867					Enteritidis (Predicted)	Enteritidis	1425	12636
SAL_EA0992AA	ASERH085062-sc-2013-02-2	ERR304863					Enteritidis (Predicted)	Enteritidis	1425	12633
SAL_EA0993AA	ASERH083817-sc-2013-02-2	ERR304862					Enteritidis (Predicted)	Enteritidis	1425	12632
SAL_EA1004AA	ASERH122384-sc-2013-02-2	ERR304851					Enteritidis	Enteritidis	3888	12624
SAL_EA1006AA	ASERH121685-sc-2013-02-2	ERR304849					Enteritidis	Enteritidis	1425	12622
SAL_EA1010AA	ASERH120613-sc-2013-02-2	ERR304845					Enteritidis	Enteritidis	3888	12619
SAL_EA1014AA	ASERH122848-sc-2013-02-2	ERR304841					Typhimurium	Enteritidis	3906	12615
SAL_EA1147AA	1025084	ERR279135					Typhi	Enteritidis	1425	12559
SAL_EA1285AA	swgs1013	SRR1029938	2013	10	North America	United States	Enteritidis	Enteritidis	1425	12496
SAL_EA1286AA	swgs1006	SRR1029937	2013	9	North America	United States	Enteritidis	Enteritidis	1425	12495
SAL_EA1287AA	swgs1015	SRR1029936	2013	10	North America	United States	Enteritidis	Enteritidis	1425	12494
SAL_EA1288AA	swgs1012	SRR1029935	2013	10	North America	United States	Enteritidis	Enteritidis	1425	12493
SAL_EA1289AA	swgs1008	SRR1029934	2013	9	North America	United States	Enteritidis	Enteritidis	8003	12492
SAL_EA1290AA	swgs1004	SRR1029933	2013	9	North America	United States	Enteritidis	Enteritidis	1425	12491

SAL_EA1291AA	swgs1005	SRR1029932	2013	9	North America	United States	Enteritidis	Enteritidis	1425	12490
SAL_EA1292AA	swgs1016	SRR1029931	2013	10	North America	United States	Enteritidis	Enteritidis	1425	12489
SAL_EA1293AA	swgs1014	SRR1029930	2013	10	North America	United States	Enteritidis	Enteritidis	1425	12488
SAL_EA1294AA	swgs1010	SRR1029929	2013	10	North America	United States	Enteritidis	Enteritidis	1425	12487
SAL_EA1295AA	swgs1007	SRR1029928	2013	9	North America	United States	Enteritidis	Enteritidis	1425	12486
SAL_EA1296AA	swgs1011	SRR1029927	2013	10	North America	United States	Enteritidis	Enteritidis	1425	12485
SAL_EA1297AA	swgs1003	SRR1029926	2013	9	North America	United States	Enteritidis	Enteritidis	1425	12484
SAL_EA1303AA	MDH-2013-00190	SRR1029577	2013	1	North America	United States	Enteritidis	Enteritidis	1425	12478
SAL_EA1313AA	AZ_TG68316	SRR1029567	2010		North America	United States	Enteritidis	Enteritidis	1425	12469
SAL_EA1327AA	NY_02028764	SRR1027064	2007	1	North America	United States	Enteritidis	Enteritidis	1425	12455
SAL_EA1328AA	NY_02027072	SRR1027063	2006	7	North America	United States	Enteritidis	Enteritidis	1425	12454
SAL_EA1330AA	NY_02026187	SRR1027061	2006	3	North America	United States	Enteritidis	Enteritidis	1425	12452
SAL_EA1348AA	AZ_TG68280	SRR1023856	2010		North America	United States	Enteritidis	Enteritidis	1425	12435
SAL_EA1354AA	NY_02022758	SRR1023850	2004	11	North America	United States	Enteritidis	Enteritidis	1425	12434
SAL_EA1355AA	NY_02022529	SRR1023849	2004	10	North America	United States	Enteritidis	Enteritidis	1425	12433
SAL_EA1356AA	NY_02021643	SRR1023848	2004	4	North America	United States	Enteritidis	Enteritidis	1425	12432
SAL_EA1404AA	AZ_TG68108	SRR1021813	2008		North America	United States	Enteritidis	Enteritidis	1425	12390
SAL_EA1439AA	FSIS1500767	SRR2239752	2014		North America	United States	Enteritidis	Enteritidis	1425	12370
SAL_EA1445AA	PNUSAS000806	SRR2229853	2015	8	North America	United States	Enteritidis	Enteritidis	1425	12364
SAL_EA1446AA	PNUSAS000804	SRR2229852	2015	8	North America	United States	Enteritidis	Enteritidis	1425	12257
SAL_EA1447AA	PNUSAS000803	SRR2229851	2015	7	North America	United States	Enteritidis	Enteritidis	1425	12363
SAL_EA1448AA	PNUSAS000802	SRR2229850	2015	7	North America	United States	Enteritidis	Enteritidis	1425	11501
SAL_EA1449AA	PNUSAS000801	SRR2229663	2015	7	North America	United States	Enteritidis	Enteritidis	1425	12362
SAL_EA1450AA	PNUSAS000800	SRR2229662			North America	United States	Enteritidis (Predicted)	Enteritidis	1425	12361
SAL_EA1489AA	PNUSAS000703	SRR2194504	2015	7	North America	United States	Enteritidis	Enteritidis	1425	2690
SAL_EA1490AA	PNUSAS000702	SRR2194503	2015	7	North America	United States	Enteritidis	Enteritidis	1425	12324
SAL_EA1491AA	PNUSAS000701	SRR2194502	2015	7	North America	United States	Enteritidis	Enteritidis	1425	12323
SAL_EA1492AA	PNUSAS000699	SRR2194501	2015	7	North America	United States	Enteritidis	Enteritidis	1425	12322
SAL_EA1493AA	PNUSAS000667	SRR2194500	2015	6	North America	United States	Enteritidis	Enteritidis	1425	8925
SAL_EA1494AA	PNUSAS000658	SRR2194499	2015	6	North America	United States	Enteritidis	Enteritidis	27885	12321
SAL_EA1495AA	PNUSAS000617	SRR2194498	2015	6	North America	United States	Enteritidis	Enteritidis	1425	12320
SAL_EA1496AA	PNUSAS000637	SRR2194497	2015	5	North America	United States	Enteritidis	Enteritidis	1425	12319
SAL_EA1497AA	PNUSAS000635	SRR2194496	2015	5	North America	United States	Enteritidis	Enteritidis	1425	12253
SAL_EA1498AA	PNUSAS000612	SRR2194495	2015	5	North America	United States	Enteritidis	Enteritidis	1425	12253
SAL_EA1501AA	PNUSAS000700	SRR2194265	2015	7	North America	United States	Enteritidis	Enteritidis	27892	5650
SAL_EA1503AA	PNUSAS000697	SRR2194108			North America	United States	Enteritidis (Predicted)	Enteritidis	1425	12316
SAL_EA1504AA	PNUSAS000696	SRR2194103			North America	United States	Enteritidis (Predicted)	Enteritidis	1425	651
SAL_EA1505AA	PNUSAS000695	SRR2194102	2015	6	North America	United States	Enteritidis	Enteritidis	1425	12315
SAL_EA1506AA	PNUSAS000694	SRR2194101	2015	7	North America	United States	Enteritidis	Enteritidis	1425	12252
SAL_EA1507AA	PNUSAS000693	SRR2194089			North America	United States	Enteritidis (Predicted)	Enteritidis	1425	12314
SAL_EA1508AA	PNUSAS000692	SRR2194083	2015	7	North America	United States	Enteritidis	Enteritidis	1425	12313
SAL_EA1509AA	PNUSAS000691	SRR2194074	2015	6	North America	United States	Enteritidis	Enteritidis	1425	12312
SAL_EA1510AA	PNUSAS000690	SRR2194069	2015	6	North America	United States	Enteritidis	Enteritidis	1425	12311
SAL_EA1511AA	PNUSAS000689	SRR2194064	2015	6	North America	United States	Enteritidis	Enteritidis	1425	8925
SAL_EA1512AA	PNUSAS000688	SRR2194036	2015	6	North America	United States	Enteritidis	Enteritidis	1425	12223
SAL_EA1513AA	PNUSAS000687	SRR2194029	2015	6	North America	United States	Enteritidis	Enteritidis	1425	12310
SAL_EA1514AA	PNUSAS000686	SRR2194024	2015	6	North America	United States	Enteritidis	Enteritidis	1425	12309
SAL_EA1515AA	PNUSAS000582	SRR2194023	2015	5	North America	United States	Enteritidis	Enteritidis	1425	12308
SAL_EA1516AA	PNUSAS000581	SRR2194022	2015	4	North America	United States	Enteritidis	Enteritidis	1425	12307
SAL_EA1543AA	PNUSAS000755	SRR2193907	2015	8	North America	United States	Enteritidis	Enteritidis	1425	12280
SAL_EA1544AA	PNUSAS000754	SRR2193903	2015	8	North America	United States	Enteritidis	Enteritidis	1425	12279
SAL_EA1548AA	PNUSAS000753	SRR2193806	2015	7	North America	United States	Enteritidis	Enteritidis	1425	12275
SAL_EA1560AA	PNUSAS000659	SRR2192248			North America	United States	Enteritidis (Predicted)	Enteritidis	1425	12266
SAL_EA1561AA	PNUSAS000614	SRR2192247			North America	United States	Enteritidis (Predicted)	Enteritidis	1425	12265
SAL_EA1564AA	PNUSAS000639	SRR2192244	2015	5	North America	United States	Enteritidis	Enteritidis	1425	12263
SAL_EA1565AA	PNUSAS000463	SRR2192243	2015	4	North America	United States	Enteritidis	Enteritidis	1425	12262
SAL_EA1566AA	PNUSAS000664	SRR2192242	2015	6	North America	United States	Enteritidis	Enteritidis	1425	12261
SAL_EA1567AA	PNUSAS000646	SRR2192241	2015	6	North America	United States	Enteritidis	Enteritidis	1425	2690
SAL_EA1568AA	PNUSAS000518	SRR2192240			North America	United States	Enteritidis (Predicted)	Enteritidis	1425	12260

SAL_EA1570AA	PNUSAS000651	SRR2192235	2015	6	North America	United States	Enteritidis	Enteritidis	1425	10152
SAL_EA1571AA	PNUSAS000663	SRR2192234	2015	6	North America	United States	Enteritidis	Enteritidis	1425	12258
SAL_EA1573AA	PNUSAS000660	SRR2192199	2015	6	North America	United States	Enteritidis	Enteritidis	1425	12257
SAL_EA1574AA	PNUSAS000665	SRR2192198	2015	6	North America	United States	Enteritidis	Enteritidis	1425	12256
SAL_EA1575AA	PNUSAS000662	SRR2192197	2015	6	North America	United States	Enteritidis	Enteritidis	1425	10709
SAL_EA1576AA	PNUSAS000706	SRR2192196	2015	7	North America	United States	Enteritidis	Enteritidis	1425	12255
SAL_EA1577AA	PNUSAS000642	SRR2192195	2015	6	North America	United States	Enteritidis	Enteritidis	1425	12254
SAL_EA1578AA	PNUSAS000520	SRR2192193	2015	6	North America	United States	Enteritidis	Enteritidis	1425	12253
SAL_EA1579AA	PNUSAS000661	SRR2192192	2015	6	North America	United States	Enteritidis	Enteritidis	1425	12252
SAL_EA1580AA	PNUSAS000521	SRR2192191	2015	6	North America	United States	Enteritidis	Enteritidis	1425	12251
SAL_EA1581AA	PNUSAS000618	SRR2192190	2015	6	North America	United States	Enteritidis	Enteritidis	1425	12250
SAL_EA1582AA	PNUSAS000517	SRR2192189	2015	5	North America	United States	Enteritidis	Enteritidis	1425	12249
SAL_EA1585AA	PNUSAS000650	SRR2192186	2015	6	North America	United States	Enteritidis	Enteritidis	1425	12247
SAL_EA1586AA	PNUSAS000525	SRR2192184	2015	6	North America	United States	Enteritidis	Enteritidis	1425	12246
SAL_EA1587AA	PNUSAS000668	SRR2192183	2015	5	North America	United States	Enteritidis	Enteritidis	1425	12245
SAL_EA1588AA	PNUSAS000522	SRR2192182	2015	6	North America	United States	Enteritidis	Enteritidis	1425	12244
SAL_EA1589AA	PNUSAS000666	SRR2192181	2015	6	North America	United States	Enteritidis	Enteritidis	1425	8925
SAL_EA1590AA	PNUSAS000652	SRR2192180	2015	6	North America	United States	Enteritidis	Enteritidis	1425	12243
SAL_EA1591AA	PNUSAS000648	SRR2192179	2015	6	North America	United States	Enteritidis	Enteritidis	1425	12242
SAL_EA1592AA	PNUSAS000705	SRR2192178	2015	7	North America	United States	Enteritidis	Enteritidis	1425	12241
SAL_EA1593AA	PNUSAS000523	SRR2192177	2015	6	North America	United States	Enteritidis	Enteritidis	1425	12240
SAL_EA1594AA	PNUSAS000654	SRR2192176	2015	6	North America	United States	Enteritidis	Enteritidis	1425	12239
SAL_EA1595AA	PNUSAS000526	SRR2192175	2015	6	North America	United States	Enteritidis	Enteritidis	1425	12238
SAL_EA1596AA	PNUSAS000636	SRR2192174	2015	5	North America	United States	Enteritidis	Enteritidis	1425	10183
SAL_EA1597AA	PNUSAS000524	SRR2192173	2015	6	North America	United States	Enteritidis	Enteritidis	1425	12237
SAL_EA1598AA	PNUSAS000640	SRR2192172	2015	5	North America	United States	Enteritidis	Enteritidis	1425	2067
SAL_EA1599AA	PNUSAS000644	SRR2192171	2015	6	North America	United States	Enteritidis	Enteritidis	1425	2067
SAL_EA1600AA	PNUSAS000645	SRR2192170	2015	5	North America	United States	Enteritidis	Enteritidis	1425	10183
SAL_EA1601AA	PNUSAS000704	SRR2192169	2015	7	North America	United States	Enteritidis	Enteritidis	1425	12236
SAL_EA1602AA	PNUSAS000481	SRR2192168	2015	5	North America	United States	Enteritidis	Enteritidis	1425	12235
SAL_EA1605AA	PNUSAS000669	SRR2192146	2015	6	North America	United States	Enteritidis	Enteritidis	1425	12232
SAL_EA1606AA	PNUSAS000649	SRR2192145	2015	6	North America	United States	Enteritidis	Enteritidis	1425	12231
SAL_EA1607AA	PNUSAS000482	SRR2192144	2015	5	North America	United States	Enteritidis	Enteritidis	1425	12230
SAL_EA1608AA	PNUSAS000655	SRR2192143	2015	6	North America	United States	Enteritidis	Enteritidis	1425	12229
SAL_EA1609AA	PNUSAS000656	SRR2192142	2015	6	North America	United States	Enteritidis	Enteritidis	1425	12228
SAL_EA1610AA	PNUSAS000513	SRR2192141	2015	5	North America	United States	Enteritidis	Enteritidis	1425	12227
SAL_EA1611AA	PNUSAS000638	SRR2192140	2015	5	North America	United States	Enteritidis	Enteritidis	1425	10152
SAL_EA1613AA	PNUSAS000615	SRR2192138	2015	5	North America	United States	Enteritidis	Enteritidis	1425	12226
SAL_EA1614AA	PNUSAS000515	SRR2192137	2015	5	North America	United States	Enteritidis	Enteritidis	1425	12225
SAL_EA1616AA	PNUSAS000653	SRR2192135	2015	6	North America	United States	Enteritidis	Enteritidis	1425	12223
SAL_EA1617AA	PNUSAS000476	SRR2192134	2015	5	North America	United States	Enteritidis	Enteritidis	1425	12222
SAL_EA1618AA	PNUSAS000641	SRR2192133	2015	6	North America	United States	Enteritidis	Enteritidis	1425	12221
SAL_EA1620AA	PNUSAS000657	SRR2192131	2015	6	North America	United States	Enteritidis	Enteritidis	1425	12219
SAL_EA1621AA	PNUSAS000516	SRR2192130	2015	5	North America	United States	Enteritidis (Predicted)	Enteritidis	1425	12218
SAL_EA1622AA	PNUSAS000479	SRR2192129	2015	5	North America	United States	Enteritidis	Enteritidis	1425	12217
SAL_EA1623AA	PNUSAS000477	SRR2192128	2015	5	North America	United States	Enteritidis	Enteritidis	27901	12216
SAL_EA1624AA	PNUSAS000472	SRR2192127	2015	3	North America	United States	Enteritidis	Enteritidis	1425	12215
SAL_EA1625AA	PNUSAS000475	SRR2192126	2015	5	North America	United States	Enteritidis	Enteritidis	1425	12214
SAL_EA1626AA	PNUSAS000647	SRR2192125	2015	6	North America	United States	Enteritidis	Enteritidis	1425	12213
SAL_EA1627AA	PNUSAS000473	SRR2192124	2015	5	North America	United States	Enteritidis	Enteritidis	8003	12205
SAL_EA1628AA	PNUSAS000478	SRR2192123			North America	United States	Enteritidis (Predicted)	Enteritidis	8003	12205
SAL_EA1629AA	PNUSAS000471	SRR2192122			North America	United States	Enteritidis (Predicted)	Enteritidis	1425	12212
SAL_EA1630AA	PNUSAS000470	SRR2192121			North America	United States	Enteritidis (Predicted)	Enteritidis	1425	12211
SAL_EA1632AA	PNUSAS000474	SRR2192119	2015	5	North America	United States	Enteritidis	Enteritidis	1425	12209
SAL_EA1633AA	PNUSAS000460	SRR2192118			North America	United States	Enteritidis (Predicted)	Enteritidis	1425	12208
SAL_EA1634AA	PNUSAS000480	SRR2192117			North America	United States	Enteritidis (Predicted)	Enteritidis	1425	12207
SAL_EA1635AA	PNUSAS000514	SRR2192116	2015	5	North America	United States	Enteritidis	Enteritidis	1425	12206
SAL_EA1636AA	PNUSAS000583	SRR2192115			North America	United States	Enteritidis (Predicted)	Enteritidis	8003	12205
SAL_EA1637AA	PNUSAS000469	SRR2192114	2015	5	North America	United States	Enteritidis	Enteritidis	1425	12204

SAL_EA1639AA	PNUSAS000464	SRR2192112	2015	5	North America	United States	Enteritidis	Enteritidis	1425	2067
SAL_EA1640AA	PNUSAS000519	SRR2192111			North America	United States	Enteritidis (Predicted)	Enteritidis	27904	12203
SAL_EA1641AA	PNUSAS000461	SRR2192110			North America	United States	Enteritidis (Predicted)	Enteritidis	1425	12202
SAL_EA1642AA	PNUSAS000462	SRR2192109			North America	United States	Enteritidis (Predicted)	Enteritidis	1425	10183
SAL_EA1643AA	PNUSAS000584	SRR2192104	2015	5	North America	United States	Enteritidis	Enteritidis	1425	12201
SAL_EA1659AA	FSIS1500748	SRR2188124	2015		North America	United States	Enteritidis	Enteritidis	1425	12191
SAL_EA1680AA	PNUSAS000490	SRR2243424	2015	4	North America	United States	Enteritidis	Enteritidis	1425	12174
SAL_EA1694AA	PNUSAS000830	SRR2321304	2015	8	North America	United States	Enteritidis	Enteritidis	1425	8600
SAL_EA1696AA	PNUSAS000824	SRR2321302	2015	8	North America	United States	Enteritidis	Enteritidis	1425	8600
SAL_EA1701AA	PNUSAS000823	SRR2321290	2015	8	North America	United States	Enteritidis	Enteritidis	1425	8600
SAL_EA1706AA	PNUSAS000827	SRR2338099	2015	8	North America	United States	Enteritidis	Enteritidis	1425	8600
SAL_EA1707AA	PNUSAS000825	SRR2338098	2015	8	North America	United States	Enteritidis	Enteritidis	1425	12157
SAL_EA1715AA	PNUSAS000828	SRR2321293	2015	8	North America	United States	Enteritidis	Enteritidis	1425	8600
SAL_EA1717AA	PNUSAS000826	SRR2320954	2015	8	North America	United States	Enteritidis	Enteritidis	1425	12148
SAL_EA1729AA	ASM_50	SRR2352234	2015		North America	United States	Enteritidis (Predicted)	Enteritidis	1425	12141
SAL_EA1730AA	ASM_49	SRR2352233	2015		North America	United States	Enteritidis (Predicted)	Enteritidis	1425	12140
SAL_EA1731AA	ASM_48	SRR2352232	2015		North America	United States	Enteritidis (Predicted)	Enteritidis	1425	12139
SAL_EA1732AA	ASM_47	SRR2352231	2015		North America	United States	Enteritidis (Predicted)	Enteritidis	1425	12138
SAL_EA1733AA	ASM_46	SRR2352230	2015		North America	United States	Enteritidis (Predicted)	Enteritidis	1425	12137
SAL_EA1734AA	ASM_45	SRR2352229	2015		North America	United States	Enteritidis (Predicted)	Enteritidis	1425	12136
SAL_EA1735AA	ASM_44	SRR2352228	2015		North America	United States	Enteritidis (Predicted)	Enteritidis	1425	12135
SAL_EA1736AA	ASM_43	SRR2352227	2015		North America	United States	Enteritidis (Predicted)	Enteritidis	1425	12134
SAL_EA1737AA	ASM_42	SRR2352226	2015		North America	United States	Enteritidis (Predicted)	Enteritidis	1425	12133
SAL_EA1738AA	ASM_41	SRR2352225	2015		North America	United States	Enteritidis (Predicted)	Enteritidis	1425	12132
SAL_EA1739AA	ASM_40	SRR2352224	2015		North America	United States	Enteritidis (Predicted)	Enteritidis	1425	12131
SAL_EA1740AA	ASM_39	SRR2352223	2015		North America	United States	Enteritidis (Predicted)	Enteritidis	1425	12130
SAL_EA1741AA	ASM_38	SRR2352222	2015		North America	United States	Enteritidis (Predicted)	Enteritidis	1425	12130
SAL_EA1742AA	ASM_37	SRR2352221	2015		North America	United States	Enteritidis (Predicted)	Enteritidis	1425	12130
SAL_EA1743AA	ASM_36	SRR2352220	2015		North America	United States	Enteritidis (Predicted)	Enteritidis	1425	12129
SAL_EA1744AA	ASM_35	SRR2352219	2015		North America	United States	Enteritidis (Predicted)	Enteritidis	1425	12129
SAL_EA1745AA	ASM_34	SRR2352218	2015		North America	United States	Enteritidis (Predicted)	Enteritidis	1425	12128
SAL_EA1746AA	ASM_33	SRR2352217	2015		North America	United States	Enteritidis (Predicted)	Enteritidis	1425	12128
SAL_EA1747AA	ASM_32	SRR2352216	2015		North America	United States	Enteritidis (Predicted)	Enteritidis	8116	12126
SAL_EA1748AA	ASM_31	SRR2352215	2015		North America	United States	Enteritidis (Predicted)	Enteritidis	1425	12127
SAL_EA1749AA	ASM_30	SRR2352214	2015		North America	United States	Enteritidis (Predicted)	Enteritidis	8116	12126
SAL_EA1750AA	ASM_29	SRR2352213	2015		North America	United States	Enteritidis (Predicted)	Enteritidis	8116	12125
SAL_EA1751AA	ASM_28	SRR2352212	2015		North America	United States	Enteritidis (Predicted)	Enteritidis	1425	12124
SAL_EA1752AA	ASM_27	SRR2352211	2015		North America	United States	Enteritidis (Predicted)	Enteritidis	1425	12124
SAL_EA1753AA	ASM_26	SRR2352210	2015		North America	United States	Enteritidis (Predicted)	Enteritidis	1425	12123
SAL_EA1754AA	ASM_25	SRR2352209	2015		North America	United States	Enteritidis (Predicted)	Enteritidis	1425	12122
SAL_EA1755AA	ASM_24	SRR2352208	2015		North America	United States	Enteritidis (Predicted)	Enteritidis	1425	12121
SAL_EA1756AA	ASM_23	SRR2352207	2015		North America	United States	Enteritidis (Predicted)	Enteritidis	1425	12120
SAL_EA1757AA	ASM_22	SRR2352206	2015		North America	United States	Enteritidis (Predicted)	Enteritidis	1425	12119
SAL_EA1758AA	ASM_21	SRR2352205	2015		North America	United States	Enteritidis (Predicted)	Enteritidis	1425	12118
SAL_EA1760AA	ASM_19	SRR2352203	2015		North America	United States	Enteritidis (Predicted)	Enteritidis	1425	12117
SAL_EA1761AA	ASM_18	SRR2352202	2015		North America	United States	Enteritidis (Predicted)	Enteritidis	1425	12116
SAL_EA1762AA	ASM_17	SRR2352201	2015		North America	United States	Enteritidis (Predicted)	Enteritidis	8116	12115
SAL_EA1763AA	ASM_16	SRR2352200	2015		North America	United States	Enteritidis (Predicted)	Enteritidis	1425	12114
SAL_EA1764AA	ASM_15	SRR2352199	2015		North America	United States	Enteritidis (Predicted)	Enteritidis	1425	12113
SAL_EA1765AA	ASM_14	SRR2352198	2015		North America	United States	Enteritidis (Predicted)	Enteritidis	1425	12112
SAL_EA1766AA	ASM_13	SRR2352197	2015		North America	United States	Enteritidis (Predicted)	Enteritidis	8116	12111
SAL_EA1767AA	ASM_12	SRR2352196	2015		North America	United States	Enteritidis (Predicted)	Enteritidis	1425	12110
SAL_EA1768AA	ASM_11	SRR2352195	2015		North America	United States	Enteritidis (Predicted)	Enteritidis	1425	12109
SAL_EA1769AA	ASM_10	SRR2352194	2015		North America	United States	Enteritidis (Predicted)	Enteritidis	-1706	12108
SAL_EA1770AA	ASM_09	SRR2352193	2015		North America	United States	Enteritidis (Predicted)	Enteritidis	1425	12107
SAL_EA1771AA	ASM_08	SRR2352192	2015		North America	United States	Enteritidis (Predicted)	Enteritidis	1425	12106
SAL_EA1772AA	ASM_07	SRR2352191	2015		North America	United States	Enteritidis (Predicted)	Enteritidis	8116	12105
SAL_EA1773AA	ASM_06	SRR2352190	2015		North America	United States	Enteritidis (Predicted)	Enteritidis	1425	12104
SAL_EA1774AA	ASM_05	SRR2352189	2015		North America	United States	Enteritidis (Predicted)	Enteritidis	1425	12103

SAL_EA1775AA	ASM_04	SRR2352188	2015		North America	United States	Enteritidis (Predicted)	Enteritidis	1425	12102
SAL_EA1776AA	ASM_03	SRR2352187	2015		North America	United States	Enteritidis (Predicted)	Enteritidis	1425	12101
SAL_EA1777AA	ASM_02	SRR2352186	2015		North America	United States	Enteritidis (Predicted)	Enteritidis	1425	12100
SAL_EA1778AA	ASM_01	SRR2352185	2015		North America	United States	Enteritidis (Predicted)	Enteritidis	1425	12099
SAL_EA1793AA	FSIS1503901	SRR2353818	2015		North America	United States	Enteritidis	Enteritidis	1425	12092
SAL_EA1804AA	2015K-0901	SRR2401921			North America	United States	Enteritidis (Predicted)	Enteritidis	1425	8925
SAL_EA1813AA	PNUSAS000487	SRR2401912	2015	5	North America	United States	Enteritidis	Enteritidis	1425	12080
SAL_EA1815AA	PNUSAS000295	SRR2401910			North America	United States	Enteritidis (Predicted)	Enteritidis	1425	12078
SAL_EA1816AA	FSIS1504079	SRR2396740	2015		North America	United States	Enteritidis	Enteritidis	1425	12077
SAL_EA1818AA	FSIS1504004	SRR2396658	2015		North America	United States	Enteritidis	Enteritidis	1425	12075
SAL_EA1819AA	FSIS1504006	SRR2396594	2015		North America	United States	Enteritidis	Enteritidis	1425	10709
SAL_EA1820AA	FSIS1504078	SRR2396593	2015		North America	United States	Enteritidis	Enteritidis	1425	12074
SAL_EA1829AA	FSIS1504080	SRR2396521	2015		North America	United States	Enteritidis	Enteritidis	1425	12068
SAL_EA1837AA	FSIS1503965	SRR2395755	2015		North America	United States	Enteritidis	Enteritidis	1425	12060
SAL_EA1881AA	CVM N55398	SRR2407798	2014		North America	United States	Enteritidis	Enteritidis	1425	11919
SAL_EA1898AA	CVM N55381	SRR2407781	2014		North America	United States	Enteritidis	Enteritidis	1425	12015
SAL_EA1921AA	CVM N54742	SRR2407758	2014		North America	United States	Enteritidis	Enteritidis	1425	11993
SAL_EA1927AA	CVM N54736	SRR2407752	2014		North America	United States	Enteritidis	Enteritidis	1425	11989
SAL_EA1947AA	CVM N54716	SRR2407732	2014		North America	United States	Enteritidis	Enteritidis	1425	11972
SAL_EA1950AA	CVM N54713	SRR2407729	2014		North America	United States	Enteritidis	Enteritidis	1425	11970
SAL_EA1951AA	CVM N54712	SRR2407728	2014		North America	United States	Enteritidis	Enteritidis	1425	11969
SAL_EA1957AA	CVM N54706	SRR2407722	2014		North America	United States	Enteritidis	Enteritidis	1425	11963
SAL_EA1975AA	CVM N54278	SRR2407704	2014		North America	United States	Enteritidis	Enteritidis	1425	11947
SAL_EA1976AA	CVM N54277	SRR2407703	2014		North America	United States	Enteritidis	Enteritidis	1425	11946
SAL_EA1981AA	CVM N54272	SRR2407698	2014		North America	United States	Enteritidis	Enteritidis	1425	11941
SAL_EA2004AA	CVM N53843	SRR2407675	2014		North America	United States	Enteritidis	Enteritidis	1425	11919
SAL_EA2027AA	CVM N53077	SRR2407652	2014		North America	United States	Enteritidis	Enteritidis	1425	11896
SAL_EA2029AA	CVM N53075	SRR2407650	2014		North America	United States	Enteritidis	Enteritidis	8003	11894
SAL_EA2050AA	CVM N53053	SRR2407629	2014		North America	United States	Enteritidis	Enteritidis	1425	11873
SAL_EA2051AA	CVM N53052	SRR2407628	2014		North America	United States	Enteritidis	Enteritidis	1425	11872
SAL_EA2052AA	CVM N53051	SRR2407627	2014		North America	United States	I 4,5,12: I : -	Enteritidis	1425	11871
SAL_EA2055AA	CVM N53048	SRR2407624	2014		North America	United States	Enteritidis	Enteritidis	1425	11869
SAL_EA2062AA	CVM N53041	SRR2407617	2014		North America	United States	Enteritidis	Enteritidis	27904	11863
SAL_EA2080AA	CVM N53022	SRR2407599	2014		North America	United States	Schwarzengrund	Enteritidis	8003	11846
SAL_EA2096AA	CVM N52062	SRR2407583	2014		North America	United States	Enteritidis	Enteritidis	1425	11830
SAL_EA2097AA	CVM N52061	SRR2407582	2014		North America	United States	Enteritidis	Enteritidis	1425	11829
SAL_EA2107AA	CVM N52051	SRR2407572	2014		North America	United States	Enteritidis	Enteritidis	1425	11820
SAL_EA2140AA	CVM N52018	SRR2407539	2014		North America	United States	Enteritidis	Enteritidis	1425	11788
SAL_EA2141AA	CVM N52017	SRR2407538	2014		North America	United States	Enteritidis	Enteritidis	1425	11787
SAL_EA2142AA	CVM N52016	SRR2407537	2014		North America	United States	Enteritidis	Enteritidis	1425	11786
SAL_EA2240AA	FSIS1504440	SRR2483569	2015		North America	United States	Enteritidis	Enteritidis	1425	11707
SAL_EA2245AA	FSIS1504441	SRR2483564	2015		North America	United States	Enteritidis	Enteritidis	26166	11703
SAL_EA2247AA	FSIS1504405	SRR2482983	2015		North America	United States	Enteritidis	Enteritidis	8003	11701
SAL_EA2248AA	FSIS1504285	SRR2482982	2015		North America	United States	Enteritidis	Enteritidis	1425	11699
SAL_EA2249AA	FSIS1504284	SRR2482981	2015		North America	United States	Enteritidis	Enteritidis	1425	11699
SAL_EA2250AA	FSIS1504219	SRR2482980	2015		North America	United States	Enteritidis	Enteritidis	1425	11700
SAL_EA2251AA	FSIS1504283	SRR2482979	2015		North America	United States	Enteritidis	Enteritidis	1425	11699
SAL_EA2252AA	FSIS1504281	SRR2482977	2015		North America	United States	Enteritidis	Enteritidis	1425	11699
SAL_EA2253AA	FSIS1504403	SRR2482976	2015		North America	United States	Enteritidis	Enteritidis	8003	11698
SAL_EA2281AA	FSIS1504501	SRR2537090	2015		North America	United States	Enteritidis	Enteritidis	1425	11696
SAL_EA2282AA	FSIS1504091	SRR2537089	2015		North America	United States	Enteritidis	Enteritidis	1425	11695
SAL_EA2283AA	FSIS1504099	SRR2537088	2015		North America	United States	Enteritidis	Enteritidis	1425	11695
SAL_EA2291AA	PNUSAS000805	SRR2533165			North America	United States	Enteritidis (Predicted)	Enteritidis	1425	11692
SAL_EA2293AA	PNUSAS000836	SRR2533163			North America	United States	Enteritidis (Predicted)	Enteritidis	1425	11255
SAL_EA2294AA	PNUSAS000835	SRR2533162	2015	8	North America	United States	Enteritidis	Enteritidis	1425	11691
SAL_EA2295AA	PNUSAS000834	SRR2533161	2015	7	North America	United States	Enteritidis	Enteritidis	1425	11690
SAL_EA2296AA	PNUSAS000833	SRR2533160			North America	United States	Enteritidis (Predicted)	Enteritidis	1425	11689
SAL_EA2310AA	FSIS1504536	SRR2532683	2015		North America	United States	Enteritidis	Enteritidis	1425	11680
SAL_EA2311AA	FSIS1504537	SRR2532682	2015		North America	United States	Enteritidis	Enteritidis	1425	11679

SAL_EA2349AA	VA-WGS-00602	SRR2542489	2008		North America	United States	Enteritidis	Enteritidis	1425	11650
SAL_EA2423AA	FDA00000460	SRR2534124	2003	7	North America	Mexico	Enteritidis	Enteritidis	1425	11587
SAL_EA2539AA	E2015012816	SRR2533570	2015	9	North America	United States	Enteritidis	Enteritidis	1425	11502
SAL_EA2540AA	E2015012699	SRR2533569	2015	9	North America	United States	Enteritidis	Enteritidis	1425	11501
SAL_EA2541AA	E2015012695	SRR2533568	2015	9	North America	United States	Enteritidis	Enteritidis	1425	11500
SAL_EA2566AA	CVM-N15804	SRR2568392	2007		North America	United States	Enteritidis	Enteritidis	1425	11476
SAL_EA2573AA	CVM-N15781	SRR2599656	2007		North America	United States	Enteritidis	Enteritidis	26678	11470
SAL_EA2583AA	E2015011742	SRR2533513	2015	8	North America	United States	Enteritidis	Enteritidis	1425	11460
SAL_EA2584AA	E2015011741	SRR2533512	2015	8	North America	United States	Enteritidis	Enteritidis	1425	11460
SAL_EA2585AA	E2015011740	SRR2533511	2015	8	North America	United States	Enteritidis	Enteritidis	1425	11460
SAL_EA2586AA	E2015011853	SRR2533509	2015	8	North America	United States	Enteritidis	Enteritidis	1425	11456
SAL_EA2587AA	E2015011730	SRR2533508	2015	8	North America	United States	Enteritidis	Enteritidis	1425	11459
SAL_EA2588AA	E2015011528	SRR2533506	2015	8	North America	United States	Enteritidis	Enteritidis	1425	11458
SAL_EA2589AA	E2015011381	SRR2533505	2015	8	North America	United States	Enteritidis	Enteritidis	1425	11457
SAL_EA2590AA	E2015011355	SRR2533504	2015	8	North America	United States	Enteritidis	Enteritidis	1425	11456
SAL_EA2591AA	E2015012226	SRR2533503	2015	8	North America	United States	Enteritidis	Enteritidis	1425	11455
SAL_EA2592AA	E2015012170	SRR2533501	2015	8	North America	United States	Enteritidis	Enteritidis	1425	11454
SAL_EA2593AA	E2015012162	SRR2533500	2015	8	North America	United States	Enteritidis	Enteritidis	1425	11453
SAL_EA2594AA	E2015012057	SRR2533499	2015	8	North America	United States	Enteritidis	Enteritidis	1425	11452
SAL_EA2595AA	E2015012021	SRR2533498	2015	8	North America	United States	Enteritidis	Enteritidis	1425	11451
SAL_EA2596AA	E2015011911	SRR2533497	2015	8	North America	United States	Enteritidis	Enteritidis	1425	11450
SAL_EA2598AA	E2015012606	SRR2533494	2015	9	North America	United States	Enteritidis	Enteritidis	1425	11448
SAL_EA2599AA	E2015012441	SRR2533493	2015	8	North America	United States	Enteritidis	Enteritidis	1425	11447
SAL_EA2632AA	WAPHL_SAL-A00845	SRR2533321	2006		North America	United States	Enteritidis	Enteritidis	1425	11422
SAL_EA2653AA	WAPHL_SAL-A00882	SRR2532787	2006		North America	United States	Enteritidis	Enteritidis	1425	11405
SAL_EA2683AA	VA-WGS-00584	SRR2533380	2008		North America	United States	Enteritidis	Enteritidis	1425	11162
SAL_EA2687AA	VA-WGS-00585	SRR2532717	2008		North America	United States	Enteritidis	Enteritidis	1425	11380
SAL_EA2741AA	CVM-N15254	SRR2532635	2007		North America	United States	Enteritidis	Enteritidis	1425	11335
SAL_EA2762AA	CVM-N15755	SRR2533455	2007		North America	United States	Enteritidis	Enteritidis	1425	11316
SAL_EA2777AA	PNUSAS001020	SRR2601072			North America	United States	Enteritidis (Predicted)	Enteritidis	1425	180
SAL_EA2778AA	PNUSAS001019	SRR2601070			North America	United States	Enteritidis (Predicted)	Enteritidis	1425	11303
SAL_EA2779AA	PNUSAS001021	SRR2601069			North America	United States	Enteritidis (Predicted)	Enteritidis	1425	11302
SAL_EA2822AA	PNUSAS000976	SRR2586132	2015	8	North America	United States	Enteritidis	Enteritidis	1425	11263
SAL_EA2824AA	PNUSAS000975	SRR2586121	2015	8	North America	United States	Enteritidis	Enteritidis	1425	11261
SAL_EA2826AA	PNUSAS000974	SRR2586076	2015	8	North America	United States	Enteritidis	Enteritidis	1425	11259
SAL_EA2829AA	PNUSAS000973	SRR2586029			North America	United States	Enteritidis (Predicted)	Enteritidis	1425	11255
SAL_EA2831AA	PNUSAS000972	SRR2586028	2015	8	North America	United States	Enteritidis	Enteritidis	1425	11255
SAL_EA2835AA	FDA00000365	SRR2586123	2003	1	Asia	China	Enteritidis	Enteritidis	1425	11253
SAL_EA2894AA	FDA00004254	SRR2585504	2013	12	Asia	China	Enteritidis	Enteritidis	1425	11205
SAL_EA2898AA	PNUSAS000962	SRR2585485	2015	9	North America	United States	Enteritidis	Enteritidis	1425	5380
SAL_EA2900AA	PNUSAS000960	SRR2585484	2015	8	North America	United States	Enteritidis (Predicted)	Enteritidis	1425	5380
SAL_EA2925AA	FDA00004166	SRR2585443	2013	5	North America	United States	Enteritidis (Predicted)	Enteritidis	1425	11179
SAL_EA2926AA	FDA00004167	SRR2585442	2013	5	North America	United States	Enteritidis (Predicted)	Enteritidis	1425	11179
SAL_EA2927AA	FDA00004168	SRR2585441	2013	5	North America	United States	Enteritidis (Predicted)	Enteritidis	1425	11179
SAL_EA2950AA	VA-WGS-00587	SRR2648191	2008		North America	United States	Enteritidis	Enteritidis	1425	11162
SAL_EA2960AA	CVM-N16404	SRR2584392	2008		North America	United States	Enteritidis	Enteritidis	1425	11154
SAL_EA2968AA	E2015013122	SRR2584383	2015	9	North America	United States	Enteritidis	Enteritidis	26543	11146
SAL_EA2969AA	E2015013115	SRR2584382	2015	9	North America	United States	Enteritidis	Enteritidis	1425	11145
SAL_EA2970AA	E2015013177	SRR2584381	2015	9	North America	United States	Enteritidis	Enteritidis	27901	11144
SAL_EA2981AA	CVM-N16429	SRR2583957	2008		North America	United States	Enteritidis	Enteritidis	1425	11135
SAL_EA2983AA	CVM-N16428	SRR2583955	2008		North America	United States	Enteritidis	Enteritidis	1425	11133
SAL_EA3050AA	WAPHL_SAL-A00941	SRR2648211	2015	9	North America	United States	Enteritidis	Enteritidis	1425	11077
SAL_EA3051AA	WAPHL_SAL-A00940	SRR2648209	2015	9	North America	United States	Enteritidis	Enteritidis	1425	11076
SAL_EA3052AA	WAPHL_SAL-A00939	SRR2648208	2015	9	North America	United States	Enteritidis	Enteritidis	1425	11075
SAL_EA3053AA	WAPHL_SAL-A00938	SRR2648207	2015	9	North America	United States	Enteritidis	Enteritidis	1425	11074
SAL_EA3054AA	WAPHL_SAL-A00937	SRR2648206	2015	8	North America	United States	Enteritidis	Enteritidis	1425	11073
SAL_EA3067AA	E2015014375	SRR2724349	2015	10	North America	United States	Enteritidis	Enteritidis	1425	11061
SAL_EA3068AA	E2015014301	SRR2724348	2015	10	North America	United States	Enteritidis	Enteritidis	1425	11060
SAL_EA3069AA	E2015014267	SRR2724329	2015	10	North America	United States	Enteritidis	Enteritidis	27904	11059

SAL_EA3070AA	E2015014162	SRR2724318	2015	9	North America	United States	Enteritidis	Enteritidis	1425	11058
SAL_EA3071AA	E2015014140	SRR2724298	2015	9	North America	United States	Enteritidis	Enteritidis	26543	11057
SAL_EA3072AA	E2015013963	SRR2724297	2015	9	North America	United States	Enteritidis	Enteritidis	1425	11056
SAL_EA3129AA	MDA113268	SRR2671588	2011	10	North America	United States	Enteritidis	Enteritidis	1425	11004
SAL_EA3130AA	MDA113267	SRR2671587	2011	10	North America	United States	Enteritidis	Enteritidis	1425	11005
SAL_EA3131AA	MDA113269	SRR2671568	2011	10	North America	United States	Enteritidis	Enteritidis	1425	11004
SAL_EA3159AA	CVM-N15822	SRR2670711	2007		North America	United States	Enteritidis	Enteritidis	1425	10986
SAL_EA3162AA	CVM-N15828	SRR2670708	2007		North America	United States	Enteritidis	Enteritidis	1425	10983
SAL_EA3165AA	CVM-N15819	SRR2670705	2007		North America	United States	Enteritidis	Enteritidis	1425	10980
SAL_EA3177AA	CVM-N16413	SRR2670691	2008		North America	United States	Enteritidis	Enteritidis	1425	10972
SAL_EA3195AA	CVM-N16445	SRR2669159	2008		North America	United States	Enteritidis	Enteritidis	1425	10956
SAL_EA3196AA	CVM-N16444	SRR2669158	2008		North America	United States	Enteritidis	Enteritidis	1425	10957
SAL_EA3298AA	2015K-1102	SRR2748728	2015	7	North America	United States	Enteritidis	Enteritidis	1425	10874
SAL_EA3299AA	2015K-1101	SRR2748727	2015	8	North America	United States	Enteritidis	Enteritidis	1425	10873
SAL_EA3300AA	2015K-1100	SRR2748726	2015	7	North America	United States	Enteritidis	Enteritidis	1425	10872
SAL_EA3309AA	PNUSAS001071	SRR2748709			North America	United States	Enteritidis (Predicted)	Enteritidis	1425	10865
SAL_EA3347AA	BS_13000-sc-2322490	ERR1082915					Enteritidis (Predicted)	Enteritidis	1425	10852
SAL_EA3357AA	BS_08037-sc-2322480	ERR1082905					Enteritidis (Predicted)	Enteritidis	1425	10845
SAL_EA3358AA	BS_08849-sc-2322479	ERR1082904					Enteritidis (Predicted)	Enteritidis	1425	10845
SAL_EA3361AA	BS_22197-sc-2322476	ERR1082901					Enteritidis (Predicted)	Enteritidis	1425	41057
SAL_EA3375AA	BS_22784-sc-2322462	ERR1082887					Enteritidis (Predicted)	Enteritidis	1425	10831
SAL_EA3377AA	BS_22400-sc-2322460	ERR1082885					Enteritidis (Predicted)	Enteritidis	1425	10831
SAL_EA3404AA	BN_7275-sc-2322433	ERR1082858					Enteritidis (Predicted)	Enteritidis	1425	6101
SAL_EA3409AA	BS_21887-sc-2322428	ERR1082853					Enteritidis (Predicted)	Enteritidis	1425	10805
SAL_EA3413AA	BS_19536-sc-2322424	ERR1082849					Enteritidis (Predicted)	Enteritidis	1425	10802
SAL_EA3414AA	BS_10338-sc-2322423	ERR1082848					Enteritidis (Predicted)	Enteritidis	1425	6101
SAL_EA3421AA	ATCC_13076__ve_control-sc	ERR1082841					Enteritidis (Predicted)	Enteritidis	1425	41062
SAL_EA3422AA	ATCC_13076_control-sc-232	ERR1082840					Enteritidis (Predicted)	Enteritidis	1425	41062
SAL_EA3423AA	BS_10167-sc-2322394	ERR1082839					Enteritidis (Predicted)	Enteritidis	1425	10794
SAL_EA3427AA	BS_7944-sc-2322390	ERR1082835					Enteritidis (Predicted)	Enteritidis	1425	10790
SAL_EA3430AA	BS_6913-sc-2322387	ERR1082832					Enteritidis (Predicted)	Enteritidis	1425	6101
SAL_EA3433AA	BS_7276-sc-2322384	ERR1082829					Enteritidis (Predicted)	Enteritidis	1425	6101
SAL_EA3434AA	BS_12051-sc-2322383	ERR1082828					Enteritidis (Predicted)	Enteritidis	1425	6101
SAL_EA3446AA	BS_15208-sc-2322369	ERR1082816					Enteritidis (Predicted)	Enteritidis	1425	10778
SAL_EA3447AA	BS_14449-sc-2322368	ERR1082815					Enteritidis (Predicted)	Enteritidis	1425	6102
SAL_EA3449AA	BS_13139-sc-2322366	ERR1082813					Enteritidis (Predicted)	Enteritidis	1425	10775
SAL_EA3450AA	BS_14756-sc-2322365	ERR1082812					Enteritidis (Predicted)	Enteritidis	1425	6102
SAL_EA3451AA	BS_07198-sc-2322363	ERR1082811					Enteritidis (Predicted)	Enteritidis	1425	6101
SAL_EA3452AA	BS_07768-sc-2322362	ERR1082810					Enteritidis (Predicted)	Enteritidis	1425	10790
SAL_EA3453AA	BS_09728-sc-2322361	ERR1082809					Enteritidis (Predicted)	Enteritidis	1425	10772
SAL_EA3454AA	BS_07045-sc-2322360	ERR1082808					Enteritidis (Predicted)	Enteritidis	1425	41081
SAL_EA3455AA	BS_07998-sc-2322359	ERR1082807					Enteritidis (Predicted)	Enteritidis	1425	6101
SAL_EA3456AA	BS_8257-sc-2322358	ERR1082806					Enteritidis (Predicted)	Enteritidis	1425	6101
SAL_EA3458AA	BS_07810-sc-2322356	ERR1082804					Enteritidis (Predicted)	Enteritidis	1425	6101
SAL_EA3460AA	BS_08179-sc-2322354	ERR1082802					Enteritidis (Predicted)	Enteritidis	1425	41415
SAL_EA3461AA	BS_06278-sc-2322353	ERR1082801					Enteritidis (Predicted)	Enteritidis	1425	10767
SAL_EA3463AA	BS_06376-sc-2322351	ERR1082799					Enteritidis (Predicted)	Enteritidis	1425	6101
SAL_EA3465AA	BS_06406-sc-2322349	ERR1082797					Enteritidis (Predicted)	Enteritidis	1425	6101
SAL_EA3467AA	BS_07052-sc-2322347	ERR1082795					Enteritidis (Predicted)	Enteritidis	1425	6101
SAL_EA3468AA	BS_08071-sc-2322346	ERR1082794					Enteritidis (Predicted)	Enteritidis	1425	10845
SAL_EA3471AA	BS_06418-sc-2322343	ERR1082791					Enteritidis (Predicted)	Enteritidis	1425	6101
SAL_EA3472AA	BS_6436-sc-2322342	ERR1082790					Enteritidis (Predicted)	Enteritidis	1425	6101
SAL_EA3474AA	BS_06252-sc-2322339	ERR1082788					Enteritidis (Predicted)	Enteritidis	1425	6101
SAL_EA3477AA	BS_06670-sc-2322336	ERR1082785					Enteritidis (Predicted)	Enteritidis	1425	6101
SAL_EA3478AA	BS_06254-sc-2322335	ERR1082784					Enteritidis (Predicted)	Enteritidis	1425	6101
SAL_EA3481AA	BS_06616-sc-2322332	ERR1082781					Enteritidis (Predicted)	Enteritidis	1425	10752
SAL_EA3483AA	BS_06444-sc-2322330	ERR1082779					Enteritidis (Predicted)	Enteritidis	1425	6101
SAL_EA3484AA	BS_03828-sc-2322329	ERR1082778					Enteritidis (Predicted)	Enteritidis	1425	10749
SAL_EA3516AA	E2015014785	SRR2759018	2015	10	North America	United States	Enteritidis (Predicted)	Enteritidis	1425	10717

SAL_EA3517AA	E2015014738	SRR2759017	2015	10	North America	United States	Enteritidis (Predicted)	Enteritidis	1425	10716
SAL_EA3526AA	FSIS1504786	SRR2778072	2015		North America	United States	Enteritidis	Enteritidis	1425	10709
SAL_EA3528AA	FSIS1504787	SRR2778060	2015		North America	United States	Enteritidis	Enteritidis	1425	10709
SAL_EA3550AA	CVM N51310	SRR2567203	2013		North America	United States	Enteritidis	Enteritidis	1425	10692
SAL_EA3551AA	CVM N51309	SRR2567202	2013		North America	United States	Enteritidis	Enteritidis	1425	10691
SAL_EA3623AA	CVM N50448	SRR2567130	2013		North America	United States	Enteritidis	Enteritidis	1425	10623
SAL_EA3624AA	CVM N50447	SRR2567129	2013		North America	United States	Enteritidis	Enteritidis	1425	10622
SAL_EA3640AA	CVM N50431	SRR2567113	2013		North America	United States	Enteritidis	Enteritidis	1425	10606
SAL_EA3645AA	CVM N50426	SRR2567108	2013		North America	United States	Enteritidis	Enteritidis	1425	10601
SAL_EA3647AA	CVM N50424	SRR2567106	2013		North America	United States	Enteritidis	Enteritidis	1425	10599
SAL_EA3662AA	CVM N48702	SRR2567091	2013		North America	United States	Enteritidis	Enteritidis	1425	10584
SAL_EA3683AA	CVM N48681	SRR2567070	2013		North America	United States	Enteritidis	Enteritidis	1425	10565
SAL_EA3694AA	CVM N47727	SRR2567059	2013		North America	United States	Enteritidis	Enteritidis	1425	10554
SAL_EA3733AA	CVM N46839	SRR2567020	2013		North America	United States	Enteritidis	Enteritidis	1425	10517
SAL_EA3734AA	CVM N46838	SRR2567019	2013		North America	United States	Enteritidis	Enteritidis	1425	10516
SAL_EA3750AA	CVM N46822	SRR2567003	2013		North America	United States	Enteritidis	Enteritidis	1425	10500
SAL_EA3755AA	CVM N46817	SRR2566998	2013		North America	United States	Enteritidis	Enteritidis	1425	10495
SAL_EA3797AA	CVM N45409	SRR2566956	2013		North America	United States	Enteritidis	Enteritidis	1425	10454
SAL_EA3799AA	CVM N45407	SRR2566954	2013		North America	United States	Enteritidis	Enteritidis	1425	10452
SAL_EA3832AA	CVM N44704	SRR2566923	2013		North America	United States	Enteritidis	Enteritidis	1425	10420
SAL_EA3850AA	CVM N43833	SRR2566907	2013		North America	United States	Enteritidis	Enteritidis	1425	10403
SAL_EA3870AA	CVM N43476	SRR2566887	2013		North America	United States	Enteritidis	Enteritidis	1425	10383
SAL_EA3876AA	CVM N43470	SRR2566881	2013		North America	United States	Enteritidis	Enteritidis	1425	10378
SAL_EA3878AA	CVM N43468	SRR2566879	2013		North America	United States	Enteritidis	Enteritidis	1425	10376
SAL_EA3882AA	CVM N43464	SRR2566875	2013		North America	United States	Enteritidis	Enteritidis	1425	10372
SAL_EA3888AA	CVM N43458	SRR2566869	2013		North America	United States	Enteritidis	Enteritidis	1425	10366
SAL_EA3889AA	CVM N43457	SRR2566868	2013		North America	United States	Enteritidis	Enteritidis	1425	10365
SAL_EA3895AA	CVM N43451	SRR2566862	2013		North America	United States	Enteritidis	Enteritidis	1425	10360
SAL_EA3897AA	CVM N43449	SRR2566860	2013		North America	United States	Enteritidis	Enteritidis	1425	10358
SAL_EA4010AA	NY59080747	SRR2566761	2015	8	North America	United States	Enteritidis	Enteritidis	1425	10276
SAL_EA4011AA	NY59080698	SRR2566760	2015	8	North America	United States	Enteritidis	Enteritidis	1425	10274
SAL_EA4012AA	NY58982376	SRR2566758	2015	8	North America	United States	Enteritidis	Enteritidis	1425	10275
SAL_EA4013AA	NY58961956	SRR2566757	2015	8	North America	United States	Enteritidis	Enteritidis	1425	10273
SAL_EA4014AA	NY58961905	SRR2566756	2015	8	North America	United States	Enteritidis	Enteritidis	1425	10272
SAL_EA4015AA	NY59238186	SRR2566753	2015	9	North America	United States	Enteritidis	Enteritidis	1425	10271
SAL_EA4016AA	NY59238138	SRR2566751	2015	9	North America	United States	Enteritidis	Enteritidis	26483	10270
SAL_EA4017AA	NY59200108	SRR2566750	2015	9	North America	United States	Enteritidis	Enteritidis	1425	10269
SAL_EA4018AA	NY59200087	SRR2566749	2015	9	North America	United States	Enteritidis	Enteritidis	1425	10268
SAL_EA4019AA	NY59136601	SRR2566747	2015	9	North America	United States	Enteritidis	Enteritidis	1425	9138
SAL_EA4020AA	NY59080685	SRR2566734	2015	8	North America	United States	Enteritidis	Enteritidis	1425	10267
SAL_EA4021AA	NY59080632	SRR2566733	2015	8	North America	United States	Enteritidis	Enteritidis	1425	10266
SAL_EA4022AA	NY58964103	SRR2566731	2015	8	North America	United States	Enteritidis	Enteritidis	1425	10265
SAL_EA4023AA	NY59238226	SRR2566730	2015	9	North America	United States	Enteritidis	Enteritidis	1425	10264
SAL_EA4024AA	NY58961892	SRR2566728	2015	8	North America	United States	Enteritidis	Enteritidis	1425	10263
SAL_EA4025AA	NY58277913	SRR2566727	2015	7	North America	United States	Enteritidis	Enteritidis	1425	10156
SAL_EA4042AA	E2015013832	SRR2559367	2015	9	North America	United States	Enteritidis	Enteritidis	1425	10247
SAL_EA4043AA	E2015013909	SRR2559366	2015	9	North America	United States	Enteritidis	Enteritidis	1425	10246
SAL_EA4044AA	E2015013787	SRR2559365	2015	9	North America	United States	Enteritidis	Enteritidis	1425	10245
SAL_EA4045AA	E2015013546	SRR2559364	2015	9	North America	United States	Enteritidis	Enteritidis	1425	10244
SAL_EA4046AA	NY58851607	SRR2559321					Enteritidis (Predicted)	Enteritidis	1425	10243
SAL_EA4047AA	NY58851516	SRR2559320					Enteritidis (Predicted)	Enteritidis	1425	10242
SAL_EA4048AA	NY58851438	SRR2559319					Enteritidis (Predicted)	Enteritidis	1425	10241
SAL_EA4049AA	NY58851695	SRR2559318					Enteritidis (Predicted)	Enteritidis	1425	10240
SAL_EA4092AA	NY58895207	SRR2451965	2015	8	North America	United States	Enteritidis	Enteritidis	1425	10211
SAL_EA4093AA	NY58895186	SRR2451964	2015	8	North America	United States	Enteritidis	Enteritidis	1425	10210
SAL_EA4094AA	NY58851851	SRR2451963	2015	6	North America	United States	Enteritidis	Enteritidis	1425	10209
SAL_EA4095AA	NY58851760	SRR2422839	2015	8	North America	United States	Enteritidis	Enteritidis	1425	10208
SAL_EA4096AA	NY58851593	SRR2422799	2015	8	North America	United States	Enteritidis	Enteritidis	1425	10207
SAL_EA4097AA	NY58800391	SRR2422756	2015	8	North America	United States	Enteritidis	Enteritidis	1425	8389

SAL_EA4099AA	0000-0053-5947	SRR2341508	2015		North America	United States	Enteritidis	Enteritidis	1425	10205
SAL_EA4103AA	0000-0051-8442	SRR2341408	2015		North America	United States	Enteritidis	Enteritidis	1425	10201
SAL_EA4104AA	NY58703876	SRR2318712	2015	8	North America	United States	Enteritidis	Enteritidis	1425	10200
SAL_EA4105AA	NY58703799	SRR2318711	2015	8	North America	United States	Enteritidis	Enteritidis	1425	10199
SAL_EA4106AA	NY58703781	SRR2318710	2015	8	North America	United States	Enteritidis	Enteritidis	1425	10198
SAL_EA4107AA	NY58703765	SRR2318709	2015	8	North America	United States	Enteritidis	Enteritidis	27779	10197
SAL_EA4108AA	NY58642507	SRR2318708	2015	8	North America	United States	Enteritidis	Enteritidis	1425	10196
SAL_EA4109AA	NY58592785	SRR2318707	2015	8	North America	United States	Enteritidis	Enteritidis	1425	10195
SAL_EA4110AA	NY58592614	SRR2318705	2015	8	North America	United States	Enteritidis	Enteritidis	1425	10194
SAL_EA4111AA	NY58364782	SRR2296852	2015	7	North America	United States	Enteritidis	Enteritidis	1425	10193
SAL_EA4112AA	NY58364767	SRR2296851	2015	8	North America	United States	Enteritidis	Enteritidis	1425	10192
SAL_EA4113AA	NY58364714	SRR2296850	2015	8	North America	United States	Enteritidis	Enteritidis	1425	10191
SAL_EA4114AA	NY58364624	SRR2296849	2015	7	North America	United States	Enteritidis	Enteritidis	1425	10190
SAL_EA4115AA	NY58364429	SRR2296848	2015	8	North America	United States	Enteritidis	Enteritidis	1425	10189
SAL_EA4116AA	NY58277261	SRR2296847	2015	7	North America	United States	Enteritidis	Enteritidis	1425	10188
SAL_EA4117AA	NY58277242	SRR2296846	2015	7	North America	United States	Enteritidis	Enteritidis	1425	10187
SAL_EA4118AA	NY58277201	SRR2296845	2015	7	North America	United States	Enteritidis	Enteritidis	1425	10186
SAL_EA4119AA	NY58474216	SRR2296843	2015	8	North America	United States	Enteritidis	Enteritidis	1425	10185
SAL_EA4120AA	NY58474062	SRR2296842	2015	8	North America	United States	Enteritidis	Enteritidis	1425	10184
SAL_EA4121AA	NY58473931	SRR2296841	2015	7	North America	United States	Enteritidis	Enteritidis	1425	10183
SAL_EA4122AA	NY58473252	SRR2296840	2015	8	North America	United States	Enteritidis	Enteritidis	1425	10182
SAL_EA4123AA	NY58276992	SRR2296839	2015	8	North America	United States	Enteritidis	Enteritidis	1425	10181
SAL_EA4124AA	NY58276931	SRR2296838	2015	8	North America	United States	Enteritidis	Enteritidis	1425	10180
SAL_EA4127AA	FSIS1501043	SRR2239957	2015		North America	United States	Enteritidis	Enteritidis	1425	10177
SAL_EA4152AA	NY58277053	SRR2218462	2015	8	North America	United States	Enteritidis	Enteritidis	1425	10159
SAL_EA4153AA	NY58276911	SRR2218461	2015	7	North America	United States	Enteritidis	Enteritidis	1425	10158
SAL_EA4154AA	NY58212073	SRR2187695	2015	7	North America	United States	Enteritidis	Enteritidis	1425	10157
SAL_EA4155AA	NY58211943	SRR2187694	2015	7	North America	United States	Enteritidis	Enteritidis	1425	10156
SAL_EA4156AA	NY58048947	SRR2187693	2015	7	North America	United States	Enteritidis	Enteritidis	1425	10155
SAL_EA4157AA	NY58048932	SRR2187692	2015	7	North America	United States	Enteritidis	Enteritidis	1425	10154
SAL_EA4158AA	NY58048917	SRR2187691	2015	7	North America	United States	Enteritidis	Enteritidis	1425	10153
SAL_EA4159AA	NY58046494	SRR2187690	2015	7	North America	United States	Enteritidis	Enteritidis	1425	10152
SAL_EA4160AA	NY58046468	SRR2187689	2015	7	North America	United States	Enteritidis	Enteritidis	1425	10152
SAL_EA4161AA	NY58046405	SRR2187688	2015	7	North America	United States	Enteritidis	Enteritidis	1425	10151
SAL_EA4162AA	NY58227041	SRR2187687	2015	7	North America	United States	Enteritidis	Enteritidis	1425	10150
SAL_EA4163AA	NY58223648	SRR2187686	2015	7	North America	United States	Enteritidis	Enteritidis	1425	10149
SAL_EA4164AA	NY58223281	SRR2187685	2015	7	North America	United States	Enteritidis	Enteritidis	1425	10148
SAL_EA4165AA	NY58221007	SRR2187684	2015	7	North America	United States	Enteritidis	Enteritidis	1425	10147
SAL_EA4166AA	NY57819061	SRR2187683	2015	7	North America	United States	Enteritidis	Enteritidis	1425	10146
SAL_EA4167AA	NY57819050	SRR2187682	2015	7	North America	United States	Enteritidis	Enteritidis	1425	10145
SAL_EA4185AA	CFSAN024735	SRR2176225	2012		South America	Chile	Enteritidis	Enteritidis	1425	10128
SAL_EA4186AA	CFSAN024734	SRR2176224	2012		South America	Chile	Enteritidis	Enteritidis	1425	10120
SAL_EA4187AA	CFSAN024733	SRR2176223	2012		South America	Chile	Enteritidis	Enteritidis	1425	10127
SAL_EA4188AA	CFSAN024732	SRR2176222	2012		South America	Chile	Enteritidis	Enteritidis	27775	10126
SAL_EA4189AA	CFSAN024731	SRR2176221	2011		South America	Chile	Enteritidis	Enteritidis	1425	10125
SAL_EA4191AA	CFSAN024729	SRR2176219	2010		South America	Chile	Enteritidis	Enteritidis	1425	10123
SAL_EA4192AA	CFSAN024755	SRR2176218	2012		South America	Chile	Enteritidis	Enteritidis	1425	10120
SAL_EA4193AA	CFSAN024754	SRR2176217	2012		South America	Chile	Enteritidis	Enteritidis	1425	10120
SAL_EA4194AA	CFSAN024753	SRR2176216	2012		South America	Chile	Enteritidis	Enteritidis	1425	10122
SAL_EA4195AA	CFSAN024752	SRR2176215	2012		South America	Chile	Enteritidis	Enteritidis	1425	10121
SAL_EA4196AA	CFSAN024751	SRR2176214	2012		South America	Chile	Enteritidis	Enteritidis	1425	10120
SAL_EA4197AA	CFSAN024750	SRR2176213	2012		South America	Chile	Enteritidis	Enteritidis	1425	10120
SAL_EA4198AA	CFSAN024749	SRR2176211	2012		South America	Chile	Enteritidis	Enteritidis	1425	10120
SAL_EA4200AA	CFSAN024747	SRR2176209	2012		South America	Chile	Enteritidis	Enteritidis	1425	10118
SAL_EA4201AA	CFSAN024746	SRR2176208	2011		South America	Chile	Enteritidis	Enteritidis	1425	10117
SAL_EA4203AA	CFSAN024745	SRR2176204	2012		South America	Chile	Enteritidis	Enteritidis	1425	10116
SAL_EA4204AA	CFSAN024744	SRR2176203	2012		South America	Chile	Enteritidis	Enteritidis	1425	10115
SAL_EA4205AA	CFSAN024743	SRR2176202	2012		South America	Chile	Enteritidis	Enteritidis	1425	10115
SAL_EA4206AA	CFSAN024742	SRR2176201	2012		South America	Chile	Enteritidis	Enteritidis	27775	10114

SAL_EA4207AA	CFSAN024741	SRR2176200	2012		South America	Chile	Enteritidis	Enteritidis	1425	10113
SAL_EA4208AA	CFSAN024740	SRR2176199	2012		South America	Chile	Enteritidis	Enteritidis	1425	10112
SAL_EA4209AA	CFSAN024739	SRR2176198	2012		South America	Chile	Enteritidis	Enteritidis	1425	10111
SAL_EA4210AA	CFSAN024738	SRR2176197	2012		South America	Chile	Enteritidis	Enteritidis	1425	10110
SAL_EA4211AA	CFSAN024737	SRR2176196	2012		South America	Chile	Enteritidis	Enteritidis	1425	10109
SAL_EA4212AA	CFSAN024736	SRR2176195	2012		South America	Chile	Enteritidis	Enteritidis	1425	10108
SAL_EA4213AA	CFSAN024727	SRR2176194	2009		South America	Chile	Enteritidis	Enteritidis	1425	1035
SAL_EA4214AA	CFSAN024726	SRR2176193	2009		South America	Chile	Enteritidis	Enteritidis	1425	10107
SAL_EA4261AA	E2015015187	SRR2831448	2015	10	North America	United States	Enteritidis	Enteritidis	26775	10068
SAL_EA4262AA	E2015014948	SRR2831447	2015	10	North America	United States	Enteritidis	Enteritidis	27901	10067
SAL_EA4263AA	E2015014944	SRR2831445	2015	10	North America	United States	Enteritidis	Enteritidis	1425	10066
SAL_EA4264AA	E2015014909	SRR2831444	2015	10	North America	United States	Enteritidis	Enteritidis	1425	10065
SAL_EA4265AA	E2015014906	SRR2831443	2015	10	North America	United States	Enteritidis	Enteritidis	27885	10064
SAL_EA4266AA	E2015014863	SRR2831442	2015	10	North America	United States	Enteritidis	Enteritidis	1425	10063
SAL_EA4267AA	E2015014853	SRR2831441	2015	10	North America	United States	Enteritidis	Enteritidis	1425	10062
SAL_EA4786AA	E2015015239	SRR2906170	2015	10	North America	United States	Enteritidis	Enteritidis	1425	3333
SAL_EA4790AA	E2015015342	SRR2906166	2015	10	North America	United States	Enteritidis	Enteritidis	1425	9593
SAL_EA4791AA	E2015015299	SRR2906165	2015	10	North America	United States	Enteritidis	Enteritidis	1425	9592
SAL_EA4792AA	E2015015259	SRR2906164	2015	10	North America	United States	Enteritidis	Enteritidis	1425	9591
SAL_EA4793AA	E2015015240	SRR2906163	2015	10	North America	United States	Enteritidis	Enteritidis	1425	9590
SAL_EA4825AA	FDA00009622	SRR2911026	2015	10	North America	United States	Enteritidis (Predicted)	Enteritidis	1425	9575
SAL_EA4829AA	FDA00001997	SRR2911006	2004	1	North America	United States	Enteritidis	Enteritidis	1425	9571
SAL_EA4830AA	FDA00001996	SRR2911005	2004	1	North America	United States	Enteritidis	Enteritidis	1425	9570
SAL_EA4832AA	FDA00001994	SRR2911003	2004	1	North America	United States	Enteritidis	Enteritidis	1425	9568
SAL_EA4868AA	CFSAN034774	SRR2910631	2014	12	North America	United States	Enteritidis (Predicted)	Enteritidis	27472	9532
SAL_EA4869AA	CFSAN034773	SRR2910630	2014	12	North America	United States	Enteritidis (Predicted)	Enteritidis	1425	9531
SAL_EA4870AA	CFSAN034772	SRR2910629	2014	12	North America	United States	Enteritidis (Predicted)	Enteritidis	1425	9530
SAL_EA4871AA	IEH-NGS-SAL-03233	SRR2910628	2014	12	North America	United States	Enteritidis (Predicted)	Enteritidis	1425	9529
SAL_EA4872AA	IEH-NGS-SAL-03232	SRR2910627	2014	12	North America	United States	Enteritidis (Predicted)	Enteritidis	1425	9528
SAL_EA4873AA	CFSAN034769	SRR2910626	2014	12	North America	United States	Enteritidis (Predicted)	Enteritidis	1425	9527
SAL_EA4874AA	CFSAN034768	SRR2910625	2014	12	North America	United States	Enteritidis (Predicted)	Enteritidis	1425	9526
SAL_EA4875AA	CFSAN034767	SRR2910624	2014	12	North America	United States	Enteritidis (Predicted)	Enteritidis	1425	9525
SAL_EA4876AA	CFSAN034766	SRR2910623	2014	12	North America	United States	Enteritidis (Predicted)	Enteritidis	1425	9524
SAL_EA4877AA	CFSAN034765	SRR2910622	2014	12	North America	United States	Enteritidis (Predicted)	Enteritidis	1425	9523
SAL_EA4910AA	WAPHL_SAL-A00935	SRR2906736	2015	8	North America	United States	Enteritidis	Enteritidis	1425	9491
SAL_EA4911AA	WAPHL_SAL-A00934	SRR2906735	2015	7	North America	United States	Enteritidis	Enteritidis	1425	9490
SAL_EA4912AA	WAPHL_SAL-A00931	SRR2906734	2015	6	North America	United States	Enteritidis	Enteritidis	1425	6712
SAL_EA4913AA	WAPHL_SAL-A00932	SRR2906733	2015	7	North America	United States	Enteritidis	Enteritidis	1425	6712
SAL_EA4920AA	VA-WGS-00532	SRR2912653	2008		North America	United States	Enteritidis	Enteritidis	1425	9483
SAL_EA4925AA	VA-WGS-00527	SRR2912645	2008		North America	United States	Enteritidis	Enteritidis	1425	9479
SAL_EA4956AA	VA-WGS-00516	SRR2911786	2008		North America	United States	Enteritidis	Enteritidis	1425	9454
SAL_EA4958AA	VA-WGS-00514	SRR2911784	2008		North America	United States	Enteritidis	Enteritidis	1425	9452
SAL_EA5013AA	E2015015718	SRR2914510	2015	10	North America	United States	Enteritidis	Enteritidis	1425	9400
SAL_EA5014AA	E2015015776	SRR2914508	2015	10	North America	United States	Enteritidis	Enteritidis	1425	9399
SAL_EA5015AA	E2015015391	SRR2914507	2015	10	North America	United States	Enteritidis	Enteritidis	1425	9398
SAL_EA5022AA	PNUSAS001070	SRR2914287	2015	8	North America	United States	Enteritidis	Enteritidis	1425	9391
SAL_EA5084AA	2015K-1106	SRR2913540			North America	United States	Enteritidis (Predicted)	Enteritidis	1425	3333
SAL_EA5085AA	2015K-1105	SRR2913539			North America	United States	Enteritidis (Predicted)	Enteritidis	1425	3333
SAL_EA5098AA	2015K-1108	SRR2913362			North America	United States	Enteritidis (Predicted)	Enteritidis	1425	9332
SAL_EA5099AA	2015K-1107	SRR2913361			North America	United States	Enteritidis (Predicted)	Enteritidis	1425	9331
SAL_EA5100AA	2015K-1104	SRR2913360			North America	United States	Enteritidis (Predicted)	Enteritidis	1425	9330
SAL_EA5105AA	PNUSAS001154	SRR2913304			North America	United States	Enteritidis (Predicted)	Enteritidis	1425	9326
SAL_EA5106AA	PNUSAS001153	SRR2913303			North America	United States	Enteritidis (Predicted)	Enteritidis	1425	9325
SAL_EA5107AA	PNUSAS001152	SRR2913302			North America	United States	Enteritidis (Predicted)	Enteritidis	1425	9324
SAL_EA5128AA	PNUSAS001156	SRR2917148			North America	United States	Enteritidis (Predicted)	Enteritidis	1425	9304
SAL_EA5129AA	PNUSAS001155	SRR2917141			North America	United States	Enteritidis (Predicted)	Enteritidis	1425	9303
SAL_EA5135AA	PNUSAS000965	SRR2917135			North America	United States	Enteritidis (Predicted)	Enteritidis	1425	5380
SAL_EA5136AA	PNUSAS000963	SRR2917134			North America	United States	Enteritidis (Predicted)	Enteritidis	1425	5380
SAL_EA5137AA	PNUSAS000964	SRR2917133			North America	United States	Enteritidis	Enteritidis	1425	5380

SAL_EA5158AA	FDA00009434	SRR2906730	2015	9			Enteritidis (Predicted)	Enteritidis	1425	9279
SAL_EA5165AA	VA-WGS-00509	SRR2918190	2008		North America	United States	Enteritidis	Enteritidis	1425	9274
SAL_EA5231AA	FSIS1502817	SRR2925647	2015		North America	United States	Enteritidis	Enteritidis	27901	9217
SAL_EA5237AA	FSIS1502815	SRR2925640	2015		North America	United States	Enteritidis	Enteritidis	1425	9213
SAL_EA5249AA	FSIS1503362	SRR2925323	2015		North America	United States	Enteritidis	Enteritidis	1425	9202
SAL_EA5310AA	PNUSAS001218	SRR2939521	2015	9	North America	United States	Enteritidis	Enteritidis	1425	5380
SAL_EA5323AA	E2015016458	SRR2954254	2015	11	North America	United States	Enteritidis	Enteritidis	1425	9138
SAL_EA5324AA	E2015016457	SRR2954253	2015	11	North America	United States	Enteritidis	Enteritidis	1425	9138
SAL_EA5337AA	FSIS1503461	SRR2954358	2015		North America	United States	Enteritidis	Enteritidis	1425	9127
SAL_EA5553AA	MOD1-Lipp-402	SRR2966834	2006	8	North America	United States	Enteritidis	Enteritidis	28719	8987
SAL_EA5620AA	2015K-0769	SRR2969503	2015	6	North America	United States	Enteritidis	Enteritidis	1425	8925
SAL_EA5690AA	E2015012194	SRR2968600	2015	8	North America	United States	Enteritidis	Enteritidis	1425	8869
SAL_EA5810AA	VA-WGS-00491	SRR2982343	2008		North America	United States	Enteritidis	Enteritidis	1425	8780
SAL_EA5971AA	E2015017264	SRR2976759	2015	11	North America	United States	Enteritidis	Enteritidis	1425	8634
SAL_EA5972AA	E2015017252	SRR2976758	2015	11	North America	United States	Enteritidis	Enteritidis	1425	8633
SAL_EA5989AA	PNUSAS000977	SRR2976019	2015	8	North America	United States	Enteritidis	Enteritidis	1425	8621
SAL_EA5990AA	PNUSAS000910	SRR2976018	2015	8	North America	United States	Enteritidis	Enteritidis	1425	8620
SAL_EA5991AA	PNUSAS000909	SRR2976017	2015	8	North America	United States	Enteritidis	Enteritidis	1425	8619
SAL_EA6012AA	PNUSAS001029	SRR2975977			North America	United States	Enteritidis (Predicted)	Enteritidis	1425	8600
SAL_EA6124AA	2015K-1103	SRR2973812			North America	United States	Enteritidis (Predicted)	Enteritidis	1425	3333
SAL_EA6161AA	2015K-0730	SRR2972338	2015	6	North America	United States	Enteritidis	Enteritidis	1425	8477
SAL_EA6178AA	ADRDL-15-6315	SRR2971407	2015		North America	United States	Enteritidis	Enteritidis	7801	8462
SAL_EA6196AA	FSIS1505003	SRR2970940	2015		North America	United States	Enteritidis	Enteritidis	1425	8447
SAL_EA6199AA	FSIS1505002	SRR2970917	2015		North America	United States	Enteritidis	Enteritidis	1425	8444
SAL_EA6200AA	FSIS1505001	SRR2970898	2015		North America	United States	Enteritidis	Enteritidis	1425	8444
SAL_EA6201AA	E2015016768	SRR2968059	2015	11	North America	United States	Enteritidis	Enteritidis	1425	8443
SAL_EA6202AA	NY59977125	SRR2937465	2015	9	North America	United States	Enteritidis	Enteritidis	1425	8442
SAL_EA6203AA	NY59371642	SRR2937464	2015	9	North America	United States	Enteritidis	Enteritidis	1425	8441
SAL_EA6204AA	NY59918542	SRR2913283	2015	10	North America	United States	Enteritidis	Enteritidis	1425	8440
SAL_EA6205AA	NY59876803	SRR2913281	2015	9	North America	United States	Enteritidis	Enteritidis	1425	8439
SAL_EA6206AA	NY59550191	SRR2913280	2015	9	North America	United States	Enteritidis	Enteritidis	1425	8438
SAL_EA6208AA	NY59478967	SRR2913277	2015	9	North America	United States	Enteritidis	Enteritidis	7811	8436
SAL_EA6209AA	NY59371436	SRR2913276	2015	9	North America	United States	Enteritidis	Enteritidis	1425	8435
SAL_EA6210AA	NY59297775	SRR2913274	2015	9	North America	United States	Enteritidis	Enteritidis	1425	8434
SAL_EA6216AA	FSIS1504878	SRR2920065	2015		North America	United States	Enteritidis	Enteritidis	1425	8428
SAL_EA6235AA	NY59977200	SRR2891612	2015	9	North America	United States	Enteritidis	Enteritidis	3888	8412
SAL_EA6236AA	NY59977027	SRR2891611	2015	10	North America	United States	Enteritidis	Enteritidis	1425	8411
SAL_EA6237AA	NY59926854	SRR2891610	2015	10	North America	United States	Enteritidis	Enteritidis	1425	8410
SAL_EA6238AA	NY59926810	SRR2891609	2015	10	North America	United States	Enteritidis	Enteritidis	1425	8409
SAL_EA6239AA	NY59926192	SRR2891608	2015	10	North America	United States	Enteritidis	Enteritidis	1425	8408
SAL_EA6240AA	NY59918937	SRR2891607	2015	10	North America	United States	Enteritidis	Enteritidis	1425	8391
SAL_EA6241AA	NY59918888	SRR2891606	2015	9	North America	United States	Enteritidis	Enteritidis	1425	8407
SAL_EA6242AA	NY59918848	SRR2891605	2015	10	North America	United States	Enteritidis	Enteritidis	1425	8406
SAL_EA6243AA	NY59918471	SRR2891604	2015	10	North America	United States	Enteritidis	Enteritidis	1425	8405
SAL_EA6244AA	NY59876834	SRR2891006	2015	9	North America	United States	Enteritidis	Enteritidis	1425	8404
SAL_EA6245AA	NY59876758	SRR2891005	2015	9	North America	United States	Enteritidis	Enteritidis	1425	8403
SAL_EA6246AA	NY59876559	SRR2891004	2015	9	North America	United States	Enteritidis	Enteritidis	1425	8402
SAL_EA6247AA	NY59371629	SRR2891003	2015	9	North America	United States	Enteritidis	Enteritidis	1425	8401
SAL_EA6248AA	NY59371508	SRR2891002	2015	9	North America	United States	Enteritidis	Enteritidis	1425	8400
SAL_EA6249AA	NY59297740	SRR2891001	2015	9	North America	United States	Enteritidis	Enteritidis	1425	8399
SAL_EA6250AA	NY59297727	SRR2891000	2015	9	North America	United States	Enteritidis	Enteritidis	1425	8398
SAL_EA6251AA	NY59297711	SRR2890999	2015	9	North America	United States	Enteritidis	Enteritidis	1425	8397
SAL_EA6252AA	NY59297691	SRR2890998	2015	9	North America	United States	Enteritidis	Enteritidis	1425	8396
SAL_EA6253AA	E2015012669	SRR2585379	2015	9	North America	United States	Enteritidis	Enteritidis	1425	8395
SAL_EA6254AA	NY59371706	SRR2847924	2015	9	North America	United States	Enteritidis	Enteritidis	1425	2054
SAL_EA6255AA	NY59297540	SRR2847923	2015	9	North America	United States	Enteritidis	Enteritidis	1425	8394
SAL_EA6256AA	NY59136577	SRR2847922	2015	8	North America	United States	Enteritidis	Enteritidis	1425	8393
SAL_EA6257AA	NY59136564	SRR2847921	2015	8	North America	United States	Enteritidis	Enteritidis	1425	2054
SAL_EA6258AA	NY59080619	SRR2847920	2015	8	North America	United States	Enteritidis	Enteritidis	1425	8392

SAL_EA6259AA	NY59680303	SRR2843234	2015	9		North America	United States	Enteritidis	Enteritidis	1425	8391
SAL_EA6260AA	NY59679385	SRR2843233	2015	9		North America	United States	Enteritidis	Enteritidis	1425	8390
SAL_EA6261AA	NY59675661	SRR2843232	2015	9		North America	United States	Enteritidis	Enteritidis	1425	5380
SAL_EA6262AA	NY59601741	SRR2843231				North America	United States	Enteritidis	Enteritidis	1425	8389
SAL_EA6263AA	NY59601725	SRR2843230	2015	8		North America	United States	Enteritidis	Enteritidis	1425	8388
SAL_EA6324AA	2015K-1359	SRR3001542				North America	United States	Enteritidis (Predicted)	Enteritidis	1425	8331
SAL_EA6325AA	2015K-1360	SRR3001541				North America	United States	Enteritidis (Predicted)	Enteritidis	1425	5380
SAL_EA6326AA	2015K-1358	SRR3001540				North America	United States	Enteritidis (Predicted)	Enteritidis	1425	5380
SAL_EA6457AA	FDA00000821	SRR3027080	2005	5		Asia	China	Enteritidis	Enteritidis	1425	8257
SAL_EA6550AA	E2015017909	SRR3037416	2015	12		North America	United States	Enteritidis	Enteritidis	1425	1018
SAL_EA6551AA	E2015017609	SRR3037415	2015	12		North America	United States	Enteritidis	Enteritidis	1425	8176
SAL_EA6552AA	AZ-TG79608	SRR3037414	2015			North America	United States	Enteritidis (Predicted)	Enteritidis	1425	4174
SAL_EA6553AA	AZ-TG79604	SRR3037413	2015			North America	United States	Enteritidis (Predicted)	Enteritidis	1425	4174
SAL_EA6554AA	AZ-TG79600	SRR3037412	2015			North America	United States	Enteritidis (Predicted)	Enteritidis	1425	8175
SAL_EA6564AA	ADRDL-126604-11-1-4	SRR3037402	2011	11		North America	United States	Dublin	Enteritidis	1425	8172
SAL_EA6619AA	2015K-1409	SRR3030381				North America	United States	Enteritidis (Predicted)	Enteritidis	1425	8127
SAL_EA6733AA	FSIS1505177	SRR3016236	2015			North America	United States	Enteritidis	Enteritidis	1425	8046
SAL_EA6734AA	FSIS1505176	SRR3016232	2015			North America	United States	Enteritidis	Enteritidis	1425	8045
SAL_EA6735AA	FSIS1505179	SRR3016114	2015			North America	United States	Enteritidis	Enteritidis	1425	8044
SAL_EA6736AA	FSIS1505178	SRR3016113	2015			North America	United States	Enteritidis	Enteritidis	1425	8044
SAL_EA6760AA	E2015017577	SRR2993269	2015	12		North America	United States	Enteritidis	Enteritidis	1425	8023
SAL_EA6761AA	E2015017571	SRR2993268	2015	11		North America	United States	Enteritidis	Enteritidis	1425	8022
SAL_EA6762AA	E2015017439	SRR2993267	2015	11		North America	United States	Enteritidis	Enteritidis	1425	8021
SAL_EA6839AA	PNUSAS001336	SRR3048029	2015	11		North America	United States	Enteritidis	Enteritidis	1425	3680
SAL_EA6853AA	FDA00004115	SRR3046418	2012	12		North America	Mexico	Rubislaw	Enteritidis	28412	7952
SAL_EA6873AA	CVM-N23800	SRR3045972	2010			North America	United States	Enteritidis	Enteritidis	33238	7933
SAL_EA6881AA	53057	SRR3049970				Europe	United Kingdom	Enteritidis	Enteritidis	1425	7927
SAL_EA6884AA	82518	SRR3049967				Europe	United Kingdom	Enteritidis	Enteritidis	3888	7925
SAL_EA6888AA	95248	SRR3049963				Europe	United Kingdom	Enteritidis	Enteritidis	1425	7921
SAL_EA6890AA	53056	SRR3049960				Europe	United Kingdom	Enteritidis	Enteritidis	3888	7919
SAL_EA6901AA	97252	SRR3049948				Europe	United Kingdom	Enteritidis	Enteritidis	1425	7575
SAL_EA6909AA	1862	SRR3049940				Europe	United Kingdom	Enteritidis	Enteritidis	1425	7903
SAL_EA6910AA	21115	SRR3049939	2014	6	4	Europe	United Kingdom	Enteritidis	Enteritidis	3888	7902
SAL_EA6911AA	21815	SRR3049937	2014	6	9	Europe	United Kingdom	Enteritidis	Enteritidis	3888	7901
SAL_EA6916AA	18553	SRR3049932				Europe	United Kingdom	Enteritidis	Enteritidis	1425	7896
SAL_EA6917AA	15240	SRR3049931				Europe	United Kingdom	Enteritidis	Enteritidis	1425	7895
SAL_EA6922AA	43965	SRR3049925				Europe	United Kingdom	Enteritidis	Enteritidis	1425	7890
SAL_EA6927AA	29965	SRR3049920				Europe	United Kingdom	Enteritidis	Enteritidis	1425	7885
SAL_EA6931AA	53020	SRR3049915				Europe	United Kingdom	Enteritidis	Enteritidis	3906	7881
SAL_EA6933AA	21869	SRR3049911				Europe	United Kingdom	Enteritidis	Enteritidis	1425	7879
SAL_EA6935AA	58566	SRR3049909				Europe	United Kingdom	Enteritidis	Enteritidis	3888	7877
SAL_EA6938AA	56990	SRR3049906				Europe	United Kingdom	Enteritidis	Enteritidis	1425	7875
SAL_EA6939AA	29236	SRR3049905				Europe	United Kingdom	Enteritidis	Enteritidis	1425	7874
SAL_EA6943AA	21113	SRR3049901	2014	6	4	Europe	United Kingdom	Enteritidis	Enteritidis	3888	7870
SAL_EA6946AA	82008	SRR3049897				Europe	United Kingdom	Enteritidis	Enteritidis	3888	3636
SAL_EA6948AA	103200	SRR3049894				Europe	United Kingdom	Enteritidis	Enteritidis	3888	7867
SAL_EA6950AA	82520	SRR3049891				Europe	United Kingdom	Enteritidis	Enteritidis	1425	7865
SAL_EA6958AA	18526	SRR3049881				Europe	United Kingdom	Enteritidis	Enteritidis	1425	7858
SAL_EA6960AA	29961	SRR3049879				Europe	United Kingdom	Enteritidis	Enteritidis	1425	7856
SAL_EA6972AA	6485	SRR3049864				Europe	United Kingdom	Weltevreden	Enteritidis	3906	7844
SAL_EA6980AA	37110	SRR3049854				Europe	United Kingdom	Enteritidis	Enteritidis	1425	7836
SAL_EA6981AA	14134	SRR3049853				Europe	United Kingdom	Enteritidis	Enteritidis	3897	7835
SAL_EA6983AA	21120	SRR3049851	2014	6	2	Europe	United Kingdom	Enteritidis	Enteritidis	3888	7833
SAL_EA6985AA	46131	SRR3049849				Europe	United Kingdom	Enteritidis	Enteritidis	1425	7831
SAL_EA6989AA	21114	SRR3049844	2014	6	4	Europe	United Kingdom	Enteritidis	Enteritidis	3888	7827
SAL_EA6990AA	29277	SRR3049843				Europe	United Kingdom	Enteritidis	Enteritidis	3888	7826
SAL_EA6991AA	6499	SRR3049842				Europe	United Kingdom	Enteritidis	Enteritidis	1425	7825
SAL_EA6992AA	29276	SRR3049841				Europe	United Kingdom	Enteritidis	Enteritidis	1425	7824
SAL_EA6995AA	21089	SRR3049838				Europe	United Kingdom	Enteritidis	Enteritidis	1425	7821

SAL_EA6997AA	29307	SRR3049835				Europe	United Kingdom	Enteritidis	Enteritidis	1425	3522
SAL_EA7007AA	56987	SRR3049824				Europe	United Kingdom	Enteritidis	Enteritidis	3888	7810
SAL_EA7010AA	53043	SRR3049821				Europe	United Kingdom	Enteritidis	Enteritidis	3888	7808
SAL_EA7011AA	82523	SRR3049820				Europe	United Kingdom	Enteritidis	Enteritidis	1425	7807
SAL_EA7013AA	95234	SRR3049818				Europe	United Kingdom	Enteritidis	Enteritidis	1425	7576
SAL_EA7015AA	60147	SRR3049816				Europe	United Kingdom	Enteritidis	Enteritidis	1425	7804
SAL_EA7020AA	43937	SRR3049811	2014	8	21	Europe	United Kingdom	Enteritidis	Enteritidis	3888	7799
SAL_EA7026AA	29310	SRR3049804				Europe	United Kingdom	Enteritidis	Enteritidis	1425	7793
SAL_EA7030AA	29279	SRR3049800				Europe	United Kingdom	Enteritidis	Enteritidis	1425	7789
SAL_EA7031AA	53074	SRR3049799				Europe	United Kingdom	Enteritidis	Enteritidis	1425	304
SAL_EA7034AA	1514	SRR3049796	2014	3	25	Europe	United Kingdom	Enteritidis	Enteritidis	3888	7786
SAL_EA7036AA	82514	SRR3049793				Europe	United Kingdom	Enteritidis	Enteritidis	1425	7784
SAL_EA7041AA	25004	SRR3049783	2014	7	3	Europe	United Kingdom	Enteritidis	Enteritidis	3888	7779
SAL_EA7044AA	7427	SRR3049779				Europe	United Kingdom	Enteritidis	Enteritidis	3888	7776
SAL_EA7045AA	21065	SRR3049778	2014	6	3	Europe	United Kingdom	Enteritidis	Enteritidis	33231	7775
SAL_EA7047AA	29308	SRR3049775	2014	7	15	Europe	United Kingdom	Enteritidis	Enteritidis	3888	7773
SAL_EA7049AA	29977	SRR3049773				Europe	United Kingdom	Enteritidis	Enteritidis	1425	7771
SAL_EA7056AA	99783	SRR3049766				Europe	United Kingdom	Enteritidis	Enteritidis	1425	7764
SAL_EA7057AA	99750	SRR3049765				Europe	United Kingdom	Enteritidis	Enteritidis	28005	7763
SAL_EA7058AA	97239	SRR3049763				Europe	United Kingdom	Enteritidis	Enteritidis	3888	7762
SAL_EA7063AA	21847	SRR3049752				Europe	United Kingdom	Enteritidis	Enteritidis	1425	7757
SAL_EA7071AA	18556	SRR3049743	2014	6	6	Europe	United Kingdom	Enteritidis	Enteritidis	3888	7750
SAL_EA7072AA	18546	SRR3049742				Europe	United Kingdom	Enteritidis	Enteritidis	1425	7749
SAL_EA7076AA	82013	SRR3049736				Europe	United Kingdom	Enteritidis	Enteritidis	1425	7117
SAL_EA7082AA	21811	SRR3049724				Europe	United Kingdom	Enteritidis	Enteritidis	3888	7740
SAL_EA7083AA	21844	SRR3049723	2014	6	10	Europe	United Kingdom	Enteritidis	Enteritidis	3888	7739
SAL_EA7084AA	43960	SRR3049722	2014	8	18	Europe	United Kingdom	Enteritidis	Enteritidis	3888	7738
SAL_EA7086AA	29972	SRR3049720	2014	7	21	Europe	United Kingdom	Enteritidis	Enteritidis	3888	7736
SAL_EA7091AA	18550	SRR3049715				Europe	United Kingdom	Enteritidis	Enteritidis	1425	7732
SAL_EA7092AA	58597	SRR3049713				Europe	United Kingdom	Enteritidis	Enteritidis	1425	7731
SAL_EA7096AA	53050	SRR3049708				Europe	United Kingdom	Enteritidis	Enteritidis	3888	7727
SAL_EA7100AA	7429	SRR3049703				Europe	United Kingdom	Enteritidis	Enteritidis	1425	7723
SAL_EA7101AA	7428	SRR3049702				Europe	United Kingdom	Enteritidis	Enteritidis	1425	7722
SAL_EA7103AA	21848	SRR3049700				Europe	United Kingdom	Enteritidis	Enteritidis	3888	7720
SAL_EA7105AA	18627	SRR3049698				Europe	United Kingdom	Enteritidis	Enteritidis	1425	7718
SAL_EA7106AA	15256	SRR3049697				Europe	United Kingdom	Enteritidis	Enteritidis	1425	7717
SAL_EA7108AA	5859	SRR3049694				Europe	United Kingdom	Enteritidis	Enteritidis	1425	7716
SAL_EA7111AA	58593	SRR3049691				Europe	United Kingdom	Enteritidis	Enteritidis	1425	7713
SAL_EA7112AA	58599	SRR3049690				Europe	United Kingdom	Enteritidis	Enteritidis	1425	7712
SAL_EA7115AA	58596	SRR3049687				Europe	United Kingdom	Enteritidis	Enteritidis	1425	7710
SAL_EA7131AA	21121	SRR3049665				Europe	United Kingdom	Enteritidis	Enteritidis	26142	7694
SAL_EA7135AA	21843	SRR3049661	2014	6	1	Europe	United Kingdom	Enteritidis	Enteritidis	3888	674
SAL_EA7141AA	1513	SRR3049653				Europe	United Kingdom	Enteritidis	Enteritidis	1425	7686
SAL_EA7142AA	58553	SRR3049652				Europe	United Kingdom	Enteritidis	Enteritidis	3888	7685
SAL_EA7144AA	97870	SRR3049650				Europe	United Kingdom	Enteritidis	Enteritidis	1425	7683
SAL_EA7146AA	45271	SRR3049645				Europe	United Kingdom	Enteritidis	Enteritidis	1425	7681
SAL_EA7150AA	18533	SRR3049640				Europe	United Kingdom	Enteritidis	Enteritidis	1425	7677
SAL_EA7155AA	43926	SRR3049635				Europe	United Kingdom	Enteritidis	Enteritidis	1425	7672
SAL_EA7160AA	29242	SRR3049630				Europe	United Kingdom	Enteritidis	Enteritidis	1425	7667
SAL_EA7165AA	1864	SRR3049624				Europe	United Kingdom	Enteritidis	Enteritidis	1425	7662
SAL_EA7167AA	34190	SRR3049621	2014	7	24	Europe	United Kingdom	Enteritidis	Enteritidis	3888	7660
SAL_EA7170AA	29240	SRR3049617				Europe	United Kingdom	Enteritidis	Enteritidis	3888	7657
SAL_EA7171AA	82011	SRR3049615				Europe	United Kingdom	Enteritidis	Enteritidis	1425	7656
SAL_EA7173AA	29966	SRR3049612				Europe	United Kingdom	Enteritidis	Enteritidis	1425	7654
SAL_EA7174AA	18661	SRR3049611				Europe	United Kingdom	Enteritidis	Enteritidis	1425	7653
SAL_EA7177AA	2305	SRR3049608				Europe	United Kingdom	Enteritidis	Enteritidis	1425	7650
SAL_EA7185AA	21854	SRR3049600				Europe	United Kingdom	Enteritidis	Enteritidis	1425	7642
SAL_EA7186AA	18513	SRR3049599				Europe	United Kingdom	Enteritidis	Enteritidis	1425	7641
SAL_EA7187AA	6498	SRR3049597				Europe	United Kingdom	Enteritidis	Enteritidis	1425	7640

SAL_EA7189AA	21840	SRR3049594				Europe	United Kingdom	Enteritidis	Enteritidis	1425	7638
SAL_EA7197AA	21087	SRR3049585				Europe	United Kingdom	Enteritidis	Enteritidis	3888	7630
SAL_EA7199AA	29286	SRR3049583				Europe	United Kingdom	Enteritidis	Enteritidis	1425	7628
SAL_EA7213AA	48128	SRR3049568				Europe	United Kingdom	Enteritidis	Enteritidis	1425	7615
SAL_EA7215AA	5853	SRR3049566				Europe	United Kingdom	Enteritidis	Enteritidis	1425	7613
SAL_EA7216AA	29971	SRR3049565	2014	7	21	Europe	United Kingdom	Enteritidis	Enteritidis	3888	7612
SAL_EA7217AA	20902	SRR3049564				Europe	United Kingdom	Enteritidis	Enteritidis	1425	7611
SAL_EA7219AA	21084	SRR3049562				Europe	United Kingdom	Enteritidis	Enteritidis	1425	7609
SAL_EA7222AA	1519	SRR3049559				Europe	United Kingdom	Enteritidis	Enteritidis	3888	7606
SAL_EA7231AA	39517	SRR3049549	2014	8	12	Europe	United Kingdom	Enteritidis	Enteritidis	3888	7599
SAL_EA7236AA	29287	SRR3049544				Europe	United Kingdom	Enteritidis	Enteritidis	3888	7594
SAL_EA7237AA	20927	SRR3049543	2014	6	8	Europe	United Kingdom	Enteritidis	Enteritidis	3888	7593
SAL_EA7238AA	48165	SRR3049542				Europe	United Kingdom	Enteritidis	Enteritidis	3906	7592
SAL_EA7247AA	18638	SRR3049533				Europe	United Kingdom	Enteritidis	Enteritidis	1425	7584
SAL_EA7252AA	18667	SRR3049528				Europe	United Kingdom	Enteritidis	Enteritidis	1425	7580
SAL_EA7254AA	82553	SRR3049526				Europe	United Kingdom	Enteritidis	Enteritidis	1425	7578
SAL_EA7256AA	97243	SRR3049524				Europe	United Kingdom	Enteritidis	Enteritidis	1425	7576
SAL_EA7257AA	97253	SRR3049523				Europe	United Kingdom	Enteritidis	Enteritidis	1425	7575
SAL_EA7258AA	82546	SRR3049522				Europe	United Kingdom	Enteritidis	Enteritidis	1425	7574
SAL_EA7266AA	21812	SRR3049514				Europe	United Kingdom	Enteritidis	Enteritidis	3888	7566
SAL_EA7267AA	43958	SRR3049513	2014	8	18	Europe	United Kingdom	Enteritidis	Enteritidis	3888	7565
SAL_EA7268AA	40325	SRR3049512				Europe	United Kingdom	Enteritidis	Enteritidis	1425	7564
SAL_EA7272AA	18528	SRR3049508				Europe	United Kingdom	Enteritidis	Enteritidis	1425	7560
SAL_EA7277AA	20926	SRR3049503	2014	6	7	Europe	United Kingdom	Enteritidis	Enteritidis	3888	7555
SAL_EA7278AA	20929	SRR3049502	2014	6	3	Europe	United Kingdom	Enteritidis	Enteritidis	3888	7554
SAL_EA7283AA	58595	SRR3049495				Europe	United Kingdom	Enteritidis	Enteritidis	1425	7550
SAL_EA7284AA	43925	SRR3049494	2014	8	13	Europe	United Kingdom	Enteritidis	Enteritidis	3888	7549
SAL_EA7297AA	2308	SRR3049481				Europe	United Kingdom	Enteritidis	Enteritidis	1425	7537
SAL_EA7299AA	56993	SRR3049479				Europe	United Kingdom	Enteritidis	Enteritidis	1425	7535
SAL_EA7300AA	45218	SRR3049478				Europe	United Kingdom	Enteritidis	Enteritidis	1425	7534
SAL_EA7302AA	43938	SRR3049476				Europe	United Kingdom	Enteritidis	Enteritidis	3888	7532
SAL_EA7305AA	29306	SRR3049473				Europe	United Kingdom	Enteritidis	Enteritidis	1425	7529
SAL_EA7315AA	1517	SRR3049463				Europe	United Kingdom	Enteritidis	Enteritidis	1425	7520
SAL_EA7316AA	95223	SRR3049462				Europe	United Kingdom	Enteritidis	Enteritidis	28005	7519
SAL_EA7318AA	56994	SRR3049460				Europe	United Kingdom	Enteritidis	Enteritidis	1425	7517
SAL_EA7325AA	58582	SRR3049453				Europe	United Kingdom	Enteritidis	Enteritidis	1425	7510
SAL_EA7331AA	29964	SRR3049446				Europe	United Kingdom	Enteritidis	Enteritidis	1425	7504
SAL_EA7332AA	15209	SRR3049445				Europe	United Kingdom	Enteritidis	Enteritidis	1425	7503
SAL_EA7333AA	21867	SRR3049444				Europe	United Kingdom	Enteritidis	Enteritidis	1425	7502
SAL_EA7334AA	20925	SRR3049443	2014	6	6	Europe	United Kingdom	Enteritidis	Enteritidis	3888	674
SAL_EA7340AA	58592	SRR3049437				Europe	United Kingdom	Enteritidis	Enteritidis	1425	7496
SAL_EA7341AA	77332	SRR3049436				Europe	United Kingdom	Enteritidis	Enteritidis	1425	7495
SAL_EA7348AA	82548	SRR3049429				Europe	United Kingdom	Enteritidis	Enteritidis	1425	7489
SAL_EA7353AA	7436	SRR3049423				Europe	United Kingdom	Enteritidis	Enteritidis	1425	7486
SAL_EA7354AA	5852	SRR3049422				Europe	United Kingdom	Enteritidis	Enteritidis	1425	7485
SAL_EA7364AA	29311	SRR3049412				Europe	United Kingdom	Enteritidis	Enteritidis	1425	7475
SAL_EA7367AA	29281	SRR3049409	2014	7	11	Europe	United Kingdom	Enteritidis	Enteritidis	3888	7473
SAL_EA7368AA	53066	SRR3049408				Europe	United Kingdom	Enteritidis	Enteritidis	3888	7472
SAL_EA7378AA	21096	SRR3049397				Europe	United Kingdom	Enteritidis	Enteritidis	1425	7462
SAL_EA7380AA	15243	SRR3049395				Europe	United Kingdom	Enteritidis	Enteritidis	1425	7460
SAL_EA7384AA	18579	SRR3049391				Europe	United Kingdom	Enteritidis	Enteritidis	1425	7457
SAL_EA7391AA	69751	SRR3049383				Europe	United Kingdom	Enteritidis	Enteritidis	3888	7452
SAL_EA7396AA	15230	SRR3049378				Europe	United Kingdom	Enteritidis	Enteritidis	26142	7447
SAL_EA7400AA	3780	SRR3049374				Europe	United Kingdom	Enteritidis	Enteritidis	1425	7443
SAL_EA7402AA	3777	SRR3049372				Europe	United Kingdom	Enteritidis	Enteritidis	1425	7441
SAL_EA7405AA	99738	SRR3049369				Europe	United Kingdom	Enteritidis	Enteritidis	1425	7438
SAL_EA7407AA	18531	SRR3049367	2014	6	4	Europe	United Kingdom	Enteritidis	Enteritidis	1425	7436
SAL_EA7408AA	18554	SRR3049366	2014	6	6	Europe	United Kingdom	Enteritidis	Enteritidis	3888	7435
SAL_EA7409AA	45269	SRR3049365	2014	8	27	Europe	United Kingdom	Enteritidis	Enteritidis	3888	7434

SAL_EA7411AA	20924	SRR3049363				Europe	United Kingdom	Enteritidis	Enteritidis	1425	7432
SAL_EA7415AA	29282	SRR3049359				Europe	United Kingdom	Enteritidis	Enteritidis	1425	7428
SAL_EA7416AA	77344	SRR3049358				Europe	United Kingdom	Enteritidis	Enteritidis	1425	6087
SAL_EA7417AA	58594	SRR3049357				Europe	United Kingdom	Enteritidis	Enteritidis	1425	7427
SAL_EA7418AA	15207	SRR3049356				Europe	United Kingdom	Enteritidis	Enteritidis	26495	7426
SAL_EA7420AA	53076	SRR3049354	2014	9	30	Europe	United Kingdom	Enteritidis	Enteritidis	1425	7424
SAL_EA7422AA	53068	SRR3049352				Europe	United Kingdom	Enteritidis	Enteritidis	1425	7422
SAL_EA7423AA	20932	SRR3049351				Europe	United Kingdom	Enteritidis	Enteritidis	1425	7421
SAL_EA7431AA	40364	SRR3049342				Europe	United Kingdom	Enteritidis	Enteritidis	3888	7415
SAL_EA7432AA	6497	SRR3049341				Europe	United Kingdom	Enteritidis	Enteritidis	33220	7414
SAL_EA7441AA	56961	SRR3049331				Europe	United Kingdom	Enteritidis	Enteritidis	1425	7406
SAL_EA7447AA	97270	SRR3049325				Europe	United Kingdom	Enteritidis	Enteritidis	1425	2479
SAL_EA7448AA	37102	SRR3049323				Europe	United Kingdom	Enteritidis	Enteritidis	1425	7400
SAL_EA7449AA	46155	SRR3049322	2014	8	27	Europe	United Kingdom	Enteritidis	Enteritidis	3888	7399
SAL_EA7451AA	46130	SRR3049320				Europe	United Kingdom	Enteritidis	Enteritidis	1425	7397
SAL_EA7455AA	18637	SRR3049316				Europe	United Kingdom	Enteritidis	Enteritidis	3888	7393
SAL_EA7460AA	48187	SRR3049311				Europe	United Kingdom	Enteritidis	Enteritidis	1425	7388
SAL_EA7463AA	48156	SRR3049308				Europe	United Kingdom	Enteritidis	Enteritidis	1425	7385
SAL_EA7464AA	18590	SRR3049307				Europe	United Kingdom	Enteritidis	Enteritidis	1425	7384
SAL_EA7465AA	29272	SRR3049306				Europe	United Kingdom	Enteritidis	Enteritidis	1425	7383
SAL_EA7467AA	21116	SRR3049304	2014	6	4	Europe	United Kingdom	Enteritidis	Enteritidis	3888	7381
SAL_EA7481AA	18524	SRR3049288				Europe	United Kingdom	Enteritidis	Enteritidis	3888	7367
SAL_EA7490AA	1512	SRR3049279				Europe	United Kingdom	Enteritidis	Enteritidis	1425	7358
SAL_EA7496AA	98285	SRR3049273				Europe	United Kingdom	Enteritidis	Enteritidis	1425	7352
SAL_EA7497AA	97248	SRR3049272				Europe	United Kingdom	Enteritidis	Enteritidis	1425	7351
SAL_EA7498AA	21126	SRR3049271				Europe	United Kingdom	Enteritidis	Enteritidis	1425	7350
SAL_EA7504AA	18555	SRR3049265	2014	6	6	Europe	United Kingdom	Enteritidis	Enteritidis	3888	7344
SAL_EA7507AA	53061	SRR3049262				Europe	United Kingdom	Enteritidis	Enteritidis	3888	7341
SAL_EA7513AA	37077	SRR3049256				Europe	United Kingdom	Enteritidis	Enteritidis	1425	7335
SAL_EA7519AA	95061	SRR3049250				Europe	United Kingdom	Enteritidis	Enteritidis	1425	7329
SAL_EA7524AA	29960	SRR3049245	2014	7	23	Europe	United Kingdom	Enteritidis	Enteritidis	1425	7324
SAL_EA7528AA	43929	SRR3049241				Europe	United Kingdom	Enteritidis	Enteritidis	1425	7320
SAL_EA7529AA	18577	SRR3049240	2014	6	6	Europe	United Kingdom	Enteritidis	Enteritidis	1425	7319
SAL_EA7531AA	15210	SRR3049238				Europe	United Kingdom	Enteritidis	Enteritidis	3888	7317
SAL_EA7534AA	100720	SRR3049235				Europe	United Kingdom	Enteritidis	Enteritidis	1425	7314
SAL_EA7535AA	9196	SRR3049234				Europe	United Kingdom	Enteritidis	Enteritidis	1425	7313
SAL_EA7540AA	21868	SRR3049229	2014	6	11	Europe	United Kingdom	Enteritidis	Enteritidis	3888	7309
SAL_EA7546AA	97251	SRR3049223				Europe	United Kingdom	Enteritidis	Enteritidis	1425	7303
SAL_EA7548AA	6500	SRR3049221				Europe	United Kingdom	Enteritidis	Enteritidis	1425	7301
SAL_EA7550AA	29963	SRR3049219				Europe	United Kingdom	Enteritidis	Enteritidis	1425	7299
SAL_EA7552AA	20913	SRR3049217				Europe	United Kingdom	Enteritidis	Enteritidis	1425	7297
SAL_EA7556AA	15239	SRR3049213				Europe	United Kingdom	Enteritidis	Enteritidis	1425	7293
SAL_EA7557AA	82010	SRR3049212				Europe	United Kingdom	Enteritidis	Enteritidis	3888	7292
SAL_EA7565AA	18532	SRR3049204				Europe	United Kingdom	Enteritidis	Enteritidis	1425	7284
SAL_EA7570AA	25185	SRR3049199				Europe	United Kingdom	Enteritidis	Enteritidis	1425	7279
SAL_EA7573AA	18523	SRR3049196				Europe	United Kingdom	Enteritidis	Enteritidis	3888	7276
SAL_EA7577AA	15244	SRR3049192				Europe	United Kingdom	Enteritidis	Enteritidis	1425	7272
SAL_EA7578AA	21846	SRR3049191	2014	6	9	Europe	United Kingdom	Enteritidis	Enteritidis	3888	7271
SAL_EA7579AA	48158	SRR3049190				Europe	United Kingdom	Enteritidis	Enteritidis	1425	7270
SAL_EA7582AA	43936	SRR3049187	2014	8	21	Europe	United Kingdom	Enteritidis	Enteritidis	3888	7267
SAL_EA7586AA	3784	SRR3049183				Europe	United Kingdom	Enteritidis	Enteritidis	1425	7263
SAL_EA7587AA	53041	SRR3049182				Europe	United Kingdom	Enteritidis	Enteritidis	1425	7262
SAL_EA7592AA	2307	SRR3049177				Europe	United Kingdom	Enteritidis	Enteritidis	1425	7257
SAL_EA7595AA	82521	SRR3049174				Europe	United Kingdom	Enteritidis	Enteritidis	1425	7254
SAL_EA7599AA	48161	SRR3049170	2014	8	24	Europe	United Kingdom	Enteritidis	Enteritidis	3888	7250
SAL_EA7600AA	46097	SRR3049169				Europe	United Kingdom	Enteritidis	Enteritidis	1425	7249
SAL_EA7608AA	21841	SRR3049161				Europe	United Kingdom	Enteritidis	Enteritidis	3888	7241
SAL_EA7611AA	21813	SRR3049158				Europe	United Kingdom	Enteritidis	Enteritidis	3888	7238
SAL_EA7612AA	43968	SRR3049157				Europe	United Kingdom	Enteritidis	Enteritidis	1425	7237

SAL_EA7614AA	29275	SRR3049155				Europe	United Kingdom	Enteritidis	Enteritidis	1425	7235
SAL_EA7615AA	43928	SRR3049154				Europe	United Kingdom	Enteritidis	Enteritidis	3888	7234
SAL_EA7617AA	1516	SRR3049152				Europe	United Kingdom	Enteritidis	Enteritidis	1425	7232
SAL_EA7621AA	95249	SRR3049148				Europe	United Kingdom	Enteritidis	Enteritidis	1425	7228
SAL_EA7626AA	20934	SRR3049143				Europe	United Kingdom	Enteritidis (Predicted)	Enteritidis	1425	7223
SAL_EA7627AA	60362	SRR3049142				Europe	United Kingdom	Enteritidis	Enteritidis	3888	7222
SAL_EA7633AA	29976	SRR3049136				Europe	United Kingdom	Enteritidis	Enteritidis	1425	7216
SAL_EA7635AA	43930	SRR3049134	2014	8	14	Europe	United Kingdom	Enteritidis	Enteritidis	3888	7214
SAL_EA7639AA	82006	SRR3049130				Europe	United Kingdom	Enteritidis	Enteritidis	1425	7210
SAL_EA7641AA	2310	SRR3049128				Europe	United Kingdom	Enteritidis	Enteritidis	1425	7208
SAL_EA7643AA	40362	SRR3049126				Europe	United Kingdom	Enteritidis	Enteritidis	1425	7206
SAL_EA7644AA	43884	SRR3049125	2014	8	13	Europe	United Kingdom	Enteritidis	Enteritidis	3888	7205
SAL_EA7646AA	21818	SRR3049123				Europe	United Kingdom	Enteritidis	Enteritidis	1425	7203
SAL_EA7648AA	45234	SRR3049121				Europe	United Kingdom	Enteritidis	Enteritidis	3888	7201
SAL_EA7653AA	6482	SRR3049116				Europe	United Kingdom	Enteritidis	Enteritidis	1425	7196
SAL_EA7665AA	20909	SRR3049104	2014	4	6	Europe	United Kingdom	Enteritidis	Enteritidis	3888	674
SAL_EA7669AA	82516	SRR3049100				Europe	United Kingdom	Enteritidis	Enteritidis	1425	7183
SAL_EA7671AA	95227	SRR3049098				Europe	United Kingdom	Enteritidis	Enteritidis	1425	7181
SAL_EA7675AA	21117	SRR3049094	2014	6	4	Europe	United Kingdom	Enteritidis	Enteritidis	3888	7177
SAL_EA7677AA	21119	SRR3049092	2014	6	4	Europe	United Kingdom	Enteritidis	Enteritidis	3888	7175
SAL_EA7678AA	43933	SRR3049091				Europe	United Kingdom	Enteritidis	Enteritidis	1425	7174
SAL_EA7682AA	21102	SRR3049087				Europe	United Kingdom	Enteritidis	Enteritidis	1425	7170
SAL_EA7684AA	19662	SRR3049085				Europe	United Kingdom	Enteritidis	Enteritidis	1425	7168
SAL_EA7689AA	20928	SRR3049080				Europe	United Kingdom	Enteritidis	Enteritidis	1425	7163
SAL_EA7693AA	48152	SRR3049076				Europe	United Kingdom	Enteritidis	Enteritidis	3888	7159
SAL_EA7694AA	29284	SRR3049075				Europe	United Kingdom	Enteritidis	Enteritidis	1425	7158
SAL_EA7696AA	18588	SRR3049073				Europe	United Kingdom	Enteritidis	Enteritidis	3888	7156
SAL_EA7699AA	29280	SRR3049070	2014	7	11	Europe	United Kingdom	Enteritidis	Enteritidis	3888	7153
SAL_EA7701AA	56991	SRR3049068	2014	9	30	Europe	United Kingdom	Enteritidis	Enteritidis	3888	7151
SAL_EA7702AA	53036	SRR3049067	2014	9	23	Europe	United Kingdom	Enteritidis	Enteritidis	3888	674
SAL_EA7703AA	82519	SRR3049066				Europe	United Kingdom	Enteritidis	Enteritidis	1425	7150
SAL_EA7705AA	95229	SRR3049064				Europe	United Kingdom	Enteritidis	Enteritidis	1425	7149
SAL_EA7706AA	18529	SRR3049063				Europe	United Kingdom	Enteritidis	Enteritidis	3888	7148
SAL_EA7708AA	20903	SRR3049061	2014	6	5	Europe	United Kingdom	Enteritidis	Enteritidis	1425	7146
SAL_EA7709AA	21849	SRR3049060				Europe	United Kingdom	Enteritidis	Enteritidis	1425	7145
SAL_EA7710AA	29285	SRR3049059				Europe	United Kingdom	Enteritidis	Enteritidis	28005	7144
SAL_EA7711AA	15211	SRR3049058				Europe	United Kingdom	Enteritidis	Enteritidis	33215	7143
SAL_EA7713AA	21124	SRR3049056				Europe	United Kingdom	Enteritidis	Enteritidis	1425	7141
SAL_EA7723AA	82012	SRR3049046				Europe	United Kingdom	Enteritidis	Enteritidis	1425	7131
SAL_EA7737AA	21118	SRR3049031				Europe	United Kingdom	Enteritidis	Enteritidis	1425	7117
SAL_EA7746AA	45232	SRR3049020				Europe	United Kingdom	Enteritidis	Enteritidis	1425	7108
SAL_EA7747AA	21820	SRR3049019				Europe	United Kingdom	Enteritidis	Enteritidis	3888	7107
SAL_EA7749AA	21086	SRR3049017				Europe	United Kingdom	Enteritidis	Enteritidis	3888	7105
SAL_EA7752AA	58576	SRR3049014	2014	9	28	Europe	United Kingdom	Enteritidis	Enteritidis	3888	7102
SAL_EA7753AA	29289	SRR3049013				Europe	United Kingdom	Enteritidis	Enteritidis	1425	7101
SAL_EA7757AA	53039	SRR3049009				Europe	United Kingdom	Enteritidis	Enteritidis	1425	7097
SAL_EA7763AA	21132	SRR3048999	2014	5	31	Europe	United Kingdom	Enteritidis	Enteritidis	-1672	7091
SAL_EA7764AA	29962	SRR3048998				Europe	United Kingdom	Enteritidis	Enteritidis	1425	7090
SAL_EA7772AA	18659	SRR3048990				Europe	United Kingdom	Enteritidis	Enteritidis	1425	7083
SAL_EA7779AA	60145	SRR3048983				Europe	United Kingdom	Enteritidis	Enteritidis	1425	7077
SAL_EA7787AA	21091	SRR3048975	2014	6	3	Europe	United Kingdom	Enteritidis	Enteritidis	1425	7069
SAL_EA7788AA	43932	SRR3048974				Europe	United Kingdom	Enteritidis	Enteritidis	1425	7068
SAL_EA7797AA	19667	SRR3048965				Europe	United Kingdom	Enteritidis	Enteritidis	1425	7060
SAL_EA7808AA	46125	SRR3048951	2014	8	23	Europe	United Kingdom	Enteritidis	Enteritidis	3888	7049
SAL_EA7809AA	48160	SRR3048950				Europe	United Kingdom	Enteritidis	Enteritidis	1425	7048
SAL_EA7810AA	45249	SRR3048949				Europe	United Kingdom	Enteritidis	Enteritidis	1425	7047
SAL_EA7811AA	43939	SRR3048947				Europe	United Kingdom	Enteritidis	Enteritidis	3888	7046
SAL_EA7814AA	77331	SRR3048944				Europe	United Kingdom	Enteritidis	Enteritidis	1425	7043
SAL_EA7822AA	20930	SRR3048935				Europe	United Kingdom	Enteritidis	Enteritidis	1425	7035

SAL_EA7824AA	29970	SRR3048933	2014	7	21	Europe	United Kingdom	Enteritidis	Enteritidis	3888	7033
SAL_EA7836AA	95232	SRR3048920				Europe	United Kingdom	Enteritidis	Enteritidis	1425	7021
SAL_EA7842AA	18589	SRR3048912				Europe	United Kingdom	Enteritidis	Enteritidis	1425	7015
SAL_EA7850AA	21088	SRR3048902				Europe	United Kingdom	Enteritidis	Enteritidis	1425	7008
SAL_EA7851AA	53013	SRR3048901				Europe	United Kingdom	Enteritidis	Enteritidis	1425	7007
SAL_EA7867AA	21122	SRR3048883				Europe	United Kingdom	Enteritidis	Enteritidis	33209	6991
SAL_EA7873AA	1863	SRR3048877				Europe	United Kingdom	Enteritidis	Enteritidis	1425	6986
SAL_EA7874AA	56992	SRR3048876				Europe	United Kingdom	Enteritidis	Enteritidis	3888	6985
SAL_EA7887AA	18525	SRR3048861				Europe	United Kingdom	Enteritidis	Enteritidis	3888	6973
SAL_EA7890AA	18527	SRR3048858				Europe	United Kingdom	Enteritidis	Enteritidis	26142	6970
SAL_EA7895AA	20933	SRR3048853				Europe	United Kingdom	Enteritidis	Enteritidis	3888	2007
SAL_EA7896AA	58602	SRR3048852				Europe	United Kingdom	Enteritidis	Enteritidis	1425	6965
SAL_EA7898AA	82509	SRR3048850				Europe	United Kingdom	Enteritidis	Enteritidis	1425	6963
SAL_EA7902AA	25172	SRR3048846				Europe	United Kingdom	Enteritidis	Enteritidis	1425	6959
SAL_EA7904AA	43909	SRR3048844	2014	8	21	Europe	United Kingdom	Enteritidis	Enteritidis	3888	6957
SAL_EA7908AA	21085	SRR3048840				Europe	United Kingdom	Enteritidis	Enteritidis	1425	6917
SAL_EA7910AA	53035	SRR3048838				Europe	United Kingdom	Enteritidis	Enteritidis	1425	6952
SAL_EA7912AA	56960	SRR3048836				Europe	United Kingdom	Enteritidis	Enteritidis	3888	6951
SAL_EA7917AA	97250	SRR3048831				Europe	United Kingdom	Enteritidis	Enteritidis	1425	6946
SAL_EA7919AA	21851	SRR3048829				Europe	United Kingdom	Enteritidis	Enteritidis	1425	6944
SAL_EA7921AA	48157	SRR3048827				Europe	United Kingdom	Enteritidis	Enteritidis	3888	6942
SAL_EA7931AA	95264	SRR3048817				Europe	United Kingdom	Enteritidis	Enteritidis	1425	6932
SAL_EA7936AA	48162	SRR3048812				Europe	United Kingdom	Enteritidis	Enteritidis	1425	6927
SAL_EA7937AA	48167	SRR3048811				Europe	United Kingdom	Enteritidis	Enteritidis	1425	6926
SAL_EA7939AA	21068	SRR3048808				Europe	United Kingdom	Enteritidis	Enteritidis	1425	6924
SAL_EA7940AA	43935	SRR3048807				Europe	United Kingdom	Enteritidis	Enteritidis	1425	6923
SAL_EA7941AA	20923	SRR3048806				Europe	United Kingdom	Enteritidis	Enteritidis	3888	6922
SAL_EA7945AA	29958	SRR3048802				Europe	United Kingdom	Enteritidis	Enteritidis	1425	6918
SAL_EA7946AA	20931	SRR3048801				Europe	United Kingdom	Enteritidis	Enteritidis	1425	6917
SAL_EA7948AA	18660	SRR3048799				Europe	United Kingdom	Enteritidis	Enteritidis	26142	6915
SAL_EA7957AA	2283	SRR3048790				Europe	United Kingdom	Enteritidis	Enteritidis	3888	6906
SAL_EA7958AA	91975	SRR3048789				Europe	United Kingdom	Enteritidis	Enteritidis	1425	6905
SAL_EA7959AA	91996	SRR3048788				Europe	United Kingdom	Enteritidis	Enteritidis	1425	6904
SAL_EA7962AA	21810	SRR3048785	2014	6	6	Europe	United Kingdom	Enteritidis	Enteritidis	3888	6902
SAL_EA7963AA	43959	SRR3048784				Europe	United Kingdom	Enteritidis	Enteritidis	1425	6901
SAL_EA7964AA	29939	SRR3048783				Europe	United Kingdom	Enteritidis	Enteritidis	1425	6900
SAL_EA7967AA	20938	SRR3048780				Europe	United Kingdom	Enteritidis	Enteritidis	1425	6897
SAL_EA7975AA	48146	SRR3048770				Europe	United Kingdom	Enteritidis	Enteritidis	1425	6889
SAL_EA7978AA	29278	SRR3048766				Europe	United Kingdom	Enteritidis	Enteritidis	1425	6886
SAL_EA7984AA	56989	SRR3048760				Europe	United Kingdom	Enteritidis	Enteritidis	1425	6880
SAL_EA7999AA	100723	SRR3048725				Europe	United Kingdom	Enteritidis	Enteritidis	1425	6865
SAL_EA8002AA	19668	SRR3048722				Europe	United Kingdom	Enteritidis	Enteritidis	1425	6863
SAL_EA8003AA	29973	SRR3048721				Europe	United Kingdom	Enteritidis	Enteritidis	3888	6862
SAL_EA8005AA	56988	SRR3048719				Europe	United Kingdom	Enteritidis	Enteritidis	1425	6860
SAL_EA8006AA	18572	SRR3048718				Europe	United Kingdom	Enteritidis	Enteritidis	1425	6859
SAL_EA8008AA	56935	SRR3048716	2014	8	1	Europe	United Kingdom	Enteritidis	Enteritidis	3888	2066
SAL_EA8011AA	2309	SRR3048713				Europe	United Kingdom	Enteritidis	Enteritidis	1425	6855
SAL_EA8019AA	15238	SRR3048703				Europe	United Kingdom	Enteritidis	Enteritidis	1425	6847
SAL_EA8029AA	29968	SRR3048691				Europe	United Kingdom	Enteritidis	Enteritidis	1425	6837
SAL_EA8030AA	6481	SRR3048690				Europe	United Kingdom	Enteritidis	Enteritidis	1425	6836
SAL_EA8040AA	15236	SRR3048680				Europe	United Kingdom	Enteritidis	Enteritidis	1425	6826
SAL_EA8043AA	58600	SRR3048656				Europe	United Kingdom	Enteritidis	Enteritidis	3888	6823
SAL_EA8047AA	82007	SRR3048603				Europe	United Kingdom	Enteritidis	Enteritidis	1425	6819
SAL_EA8049AA	18557	SRR3048580	2014	6	6	Europe	United Kingdom	Enteritidis	Enteritidis	3888	6818
SAL_EA8050AA	21845	SRR3048579	2014	6	10	Europe	United Kingdom	Enteritidis	Enteritidis	3888	6817
SAL_EA8057AA	29299	SRR3048572				Europe	United Kingdom	Enteritidis	Enteritidis	1425	6810
SAL_EA8061AA	1861	SRR3048568				Europe	United Kingdom	Enteritidis	Enteritidis	1425	6806
SAL_EA8068AA	21831	SRR3048560				Europe	United Kingdom	Enteritidis	Enteritidis	1425	6799
SAL_EA8076AA	18607	SRR3048552				Europe	United Kingdom	Enteritidis	Enteritidis	1425	6791

SAL_EA8077AA	29274	SRR3048551				Europe	United Kingdom	Enteritidis	Enteritidis	1425	6790
SAL_EA8091AA	29969	SRR3048535				Europe	United Kingdom	Enteritidis	Enteritidis	1425	6776
SAL_EA8092AA	1515	SRR3048534				Europe	United Kingdom	Enteritidis	Enteritidis	1425	6775
SAL_EA8096AA	15242	SRR3048529				Europe	United Kingdom	Enteritidis	Enteritidis	3888	6771
SAL_EA8103AA	95034	SRR3048521				Europe	United Kingdom	Enteritidis	Enteritidis	1425	6764
SAL_EA8108AA	43961	SRR3048515				Europe	United Kingdom	Enteritidis	Enteritidis	3888	6759
SAL_EA8110AA	163178_S15	SRR3047223				Europe	United Kingdom	Enteritidis	Enteritidis	1425	3680
SAL_EA8112AA	201405760	traces-0JiMSsi	2014	7	23	Europe	France	Enteritidis	Enteritidis	3888	512
SAL_EA8113AA	201405756	traces-0qWOdHp	2014	7	21	Europe	France	Enteritidis	Enteritidis	3888	554
SAL_EA8159AA	WAPHL_SAL-A00933	SRR3053228	2015	7		North America	United States	Enteritidis	Enteritidis	1425	6712
SAL_EA8160AA	WAPHL_SAL-A00930	SRR3053227	2015	6		North America	United States	Enteritidis	Enteritidis	1425	6711
SAL_EA8161AA	WAPHL_SAL-A01150	SRR3052034	2015	11		North America	United States	Enteritidis	Enteritidis	1425	6710
SAL_EA8162AA	WAPHL_SAL-A01149	SRR3052033	2015	11		North America	United States	Enteritidis	Enteritidis	1425	6709
SAL_EA8163AA	WAPHL_SAL-A01148	SRR3052032	2015	11		North America	United States	Enteritidis	Enteritidis	1425	6709
SAL_EA8164AA	WAPHL_SAL-A01147	SRR3052031	2015	11		North America	United States	Enteritidis	Enteritidis	26775	6708
SAL_EA8173AA	CVM-N23779	SRR3052022	2010			North America	United States	Albany	Enteritidis	1425	6699
SAL_EA8203AA	WAPHL-SAL-A00985	SRR3082383	2009			North America	United States	Enteritidis	Enteritidis	1425	6670
SAL_EA8245AA	CFSAN031777	SRR3081649	2013	5		Africa	Ethiopia	Typhimurium	Enteritidis	7942	6642
SAL_EA8416AA	201405122	traces-0wRGnlX	2014	7	19	Europe	France	Enteritidis	Enteritidis	3888	553
SAL_EA8417AA	201405861	traces-0hvfoaj	2014	7	18	Europe	France	Enteritidis	Enteritidis	3888	556
SAL_EA8419AA	201405757	traces-0foIqty	2014	7	2	Europe	France	Enteritidis	Enteritidis	3888	555
SAL_EA8492AA	VA-WGS-00657	SRR3085566	2014			North America	United States	Enteritidis (Predicted)	Enteritidis	33238	6446
SAL_EA8506AA	WAPHL_SAL-A01146	SRR3086931	2015	11		North America	United States	Enteritidis	Enteritidis	1425	6441
SAL_EA8507AA	WAPHL_SAL-A01145	SRR3086930	2015	10		North America	United States	Enteritidis	Enteritidis	1425	6440
SAL_EA8508AA	WAPHL_SAL-A01144	SRR3086929	2015	10		North America	United States	Enteritidis	Enteritidis	26775	6439
SAL_EA8509AA	WAPHL_SAL-A01143	SRR3086928	2015	10		North America	United States	Enteritidis	Enteritidis	1425	6438
SAL_EA8510AA	WAPHL_SAL-A01142	SRR3086927	2015	9		North America	United States	Enteritidis	Enteritidis	1425	6437
SAL_EA8511AA	WAPHL_SAL-A01141	SRR3086922	2015	9		North America	United States	Enteritidis	Enteritidis	1425	6436
SAL_EA8521AA	CFSAN031671	SRR3088941	2005	9		Africa	Ethiopia	Enteritidis (Predicted)	Enteritidis	3801	5947
SAL_EA8522AA	CFSAN031686	SRR3088940	2005	9		Africa	Ethiopia	Enteritidis (Predicted)	Enteritidis	3801	5947
SAL_EA8524AA	CFSAN031681	SRR3088938	2005	9		Africa	Ethiopia	Enteritidis (Predicted)	Enteritidis	3801	5947
SAL_EA8526AA	CFSAN031668	SRR3088933	2005	9		Africa	Ethiopia	Enteritidis (Predicted)	Enteritidis	3801	5947
SAL_EA8527AA	CFSAN031688	SRR3088932	2005	9		Africa	Ethiopia	Enteritidis (Predicted)	Enteritidis	3801	6430
SAL_EA8528AA	CFSAN031683	SRR3088931	2005	9		Africa	Ethiopia	Enteritidis (Predicted)	Enteritidis	3801	5947
SAL_EA8529AA	CFSAN031670	SRR3088930	2005	9		Africa	Ethiopia	Enteritidis (Predicted)	Enteritidis	3801	6429
SAL_EA8532AA	CFSAN031680	SRR3088927	2005	9		Africa	Ethiopia	Enteritidis (Predicted)	Enteritidis	3801	5947
SAL_EA8533AA	CFSAN031667	SRR3088926	2005	9		Africa	Ethiopia	Enteritidis (Predicted)	Enteritidis	3801	5947
SAL_EA8534AA	CFSAN031687	SRR3088925	2005	9		Africa	Ethiopia	Enteritidis (Predicted)	Enteritidis	3801	6427
SAL_EA8536AA	CFSAN031682	SRR3088923	2005	9		Africa	Ethiopia	Enteritidis (Predicted)	Enteritidis	3801	5947
SAL_EA8537AA	CFSAN031684	SRR3088922	2005	9		Africa	Ethiopia	Enteritidis (Predicted)	Enteritidis	3801	6426
SAL_EA8539AA	CFSAN031669	SRR3088920	2005	9		Africa	Ethiopia	Enteritidis (Predicted)	Enteritidis	3801	6424
SAL_EA8540AA	CFSAN031679	SRR3088919	2005	9		Africa	Ethiopia	Enteritidis (Predicted)	Enteritidis	3801	5947
SAL_EA8565AA	WAPHL-SAL-A00979	SRR3089999	2009			North America	United States	Enteritidis	Enteritidis	1425	6406
SAL_EA8568AA	WAPHL-SAL-A00976	SRR3089996	2009			North America	United States	Enteritidis	Enteritidis	1425	6403
SAL_EA8573AA	NY-N19971	SRR3091415	2009			North America	United States	Enteritidis	Enteritidis	1425	6398
SAL_EA8575AA	NY-N19969	SRR3091413	2009			North America	United States	Enteritidis	Enteritidis	1425	6396
SAL_EA8609AA	NY-N19890	SRR3091347	2008			North America	United States	Enteritidis	Enteritidis	1425	6362
SAL_EA8642AA	FX_89_1	traces-0kPIPiA	1989			Europe	France	Paratyphi C	Enteritidis	1425	6338
SAL_EA8655AA	NY-N19878	SRR3091340	2008			North America	United States	Enteritidis	Enteritidis	1425	6325
SAL_EA8667AA	NY-N19876	SRR3091328	2008			North America	United States	Enteritidis	Enteritidis	1425	6313
SAL_EA8725AA	NY-N19993	SRR3096104	2009			North America	United States	Enteritidis	Enteritidis	1425	6269
SAL_EA8763AA	CDPHFDLB-F14M01163-1	SRR3096070	2014	7		North America	United States	Enteritidis (Predicted)	Enteritidis	1425	6235
SAL_EA8804AA	CDPHFDLB-F14M01163-2	SRR3096061	2014	7		North America	United States	Enteritidis (Predicted)	Enteritidis	1425	6200
SAL_EA8838AA	CVM-N23799	SRR3099648	2010			North America	United States	Enteritidis	Enteritidis	1425	6171
SAL_EA8847AA	CVM-N23756	SRR3099620	2010			North America	United States	Enteritidis	Enteritidis	1425	6162
SAL_EA8867AA	WAPHL-SAL-A01025	SRR3101888	2009			North America	United States	Enteritidis	Enteritidis	1425	6142
SAL_EA8869AA	PNUSAS001388	SRR3103842				North America	United States	Enteritidis (Predicted)	Enteritidis	1425	6140
SAL_EA8870AA	PNUSAS001387	SRR3103840	2015	12		North America	United States	Enteritidis	Enteritidis	1425	6139
SAL_EA8871AA	PNUSAS001386	SRR3103839	2015	12		North America	United States	Enteritidis	Enteritidis	1425	6138

SAL_EA8872AA	PNUSAS001385	SRR3103838	2015	12		North America	United States	Enteritidis	Enteritidis	1425	6138
SAL_EA8873AA	PNUSAS001383	SRR3103837	2015	12		North America	United States	Enteritidis	Enteritidis	27901	6137
SAL_EA8881AA	PNUSAS001374	SRR3103829	2015	9		North America	United States	Enteritidis	Enteritidis	1425	6129
SAL_EA8883AA	PNUSAS001372	SRR3103827	2015	10		North America	United States	Enteritidis (Predicted)	Enteritidis	1425	6127
SAL_EA8893AA	LSS-6	traces-0KwBNyr	2015			Europe	United Kingdom	Enteritidis (Predicted)	Enteritidis	1425	6117
SAL_EA8896AA	LSS-9	traces-0OkNPGs	2015			Europe	United Kingdom	Enteritidis (Predicted)	Enteritidis	1425	6114
SAL_EA8942AA	BS_14759-sc-2322364	ERR1216910						Enteritidis (Predicted)	Enteritidis	1425	6102
SAL_EA8943AA	BS_06626-sc-2322341	ERR1216909						Enteritidis (Predicted)	Enteritidis	1425	6101
SAL_EA8962AA	E2015017856	SRR3110501	2015	12		North America	United States	Enteritidis	Enteritidis	1425	6089
SAL_EA8963AA	E2015018531	SRR3110500	2015	12		North America	United States	Enteritidis	Enteritidis	1425	6088
SAL_EA8964AA	E2016000176	SRR3110499	2015	12		North America	United States	Enteritidis	Enteritidis	1425	6087
SAL_EA8965AA	E2016000204	SRR3110498	2015	12		North America	United States	Enteritidis	Enteritidis	1425	6086
SAL_EA8966AA	E2016000215	SRR3110497	2016	1		North America	United States	Enteritidis	Enteritidis	1425	6085
SAL_EA8994AA	WAPHL-SAL-A01040	SRR3110468	2009			North America	United States	Enteritidis	Enteritidis	1425	6057
SAL_EA9002AA	CDPHFDLB-F14M01191d	SRR3110378	2014	7		North America	United States	Enteritidis	Enteritidis	1425	6050
SAL_EA9003AA	CDPHFDLB-F14M01191c	SRR3110377	2014	7		North America	United States	Enteritidis	Enteritidis	1425	6049
SAL_EA9025AA	FDA00009786	SRR3114034	2000	3		Asia	Republic of the Union of Myanmar	Enteritidis (Predicted)	Enteritidis	1425	6029
SAL_EA9100AA	CFSAN031666	SRR3116008	2005	9		Africa	Ethiopia	Enteritidis (Predicted)	Enteritidis	3801	5959
SAL_EA9102AA	CFSAN031663	SRR3116006	2005	9		Africa	Ethiopia	Enteritidis (Predicted)	Enteritidis	3801	5957
SAL_EA9105AA	CFSAN031665	SRR3116003	2005	9		Africa	Ethiopia	Enteritidis (Predicted)	Enteritidis	3801	5954
SAL_EA9109AA	CFSAN031662	SRR3115998	2005	9		Africa	Ethiopia	Enteritidis (Predicted)	Enteritidis	3801	5950
SAL_EA9112AA	CFSAN031664	SRR3115995	2005	9		Africa	Ethiopia	Enteritidis (Predicted)	Enteritidis	3801	5947
SAL_EA9121AA	CFSAN031736	SRR3115985	2005	9		Africa	Ethiopia	Enteritidis (Predicted)	Enteritidis	33152	5940
SAL_EA9159AA	WAPHL-SAL-A01088	SRR3116156	2009			North America	United States	Enteritidis	Enteritidis	1425	5905
SAL_EA9258AA	E2016000801	SRR3131112	2016	1		North America	United States	Enteritidis	Enteritidis	1425	5820
SAL_EA9259AA	E2016000740	SRR3131111	2016	1		North America	United States	Enteritidis	Enteritidis	1425	5819
SAL_EA9314AA	38368	SRR3120716	2014	7	31	Europe	United Kingdom	Enteritidis	Enteritidis	3888	5791
SAL_EA9317AA	36461	SRR3120711				Europe	United Kingdom	Enteritidis	Enteritidis	3888	5789
SAL_EA9321AA	43927	SRR3120707	2014	8	17	Europe	United Kingdom	Enteritidis	Enteritidis	3888	5788
SAL_EA9323AA	57093	SRR3120669				Europe	United Kingdom	Enteritidis	Enteritidis	3900	5787
SAL_EA9325AA	36462	SRR3120667				Europe	United Kingdom	Enteritidis	Enteritidis	1425	5786
SAL_EA9355AA	FDA00000266	SRR3137523	2002	6		Asia	Thailand	Enteritidis	Enteritidis	1425	5772
SAL_EA9378AA	PNUSAS001392	SRR3137222	2015	10		North America	United States	Enteritidis	Enteritidis	1425	5752
SAL_EA9400AA	2016K-0030	SRR3137166				North America	United States	Enteritidis (Predicted)	Enteritidis	1425	5730
SAL_EA9420AA	PNUSAS001391	SRR3137132				North America	United States	Enteritidis (Predicted)	Enteritidis	1425	5714
SAL_EA9421AA	PNUSAS001390	SRR3137129				North America	United States	Enteritidis (Predicted)	Enteritidis	1425	5713
SAL_EA9424AA	PNUSAS001393	SRR3137124				North America	United States	Enteritidis (Predicted)	Enteritidis	1425	5710
SAL_EA9433AA	PNUSAS001478	SRR3137085				North America	United States	Enteritidis (Predicted)	Enteritidis	1425	5701
SAL_EA9434AA	PNUSAS001477	SRR3137084	2015	12		North America	United States	Enteritidis	Enteritidis	1425	5700
SAL_EA9436AA	PNUSAS001475	SRR3137081	2015	12		North America	United States	Enteritidis	Enteritidis	1425	5698
SAL_EA9437AA	PNUSAS001474	SRR3137080	2015	11		North America	United States	Enteritidis	Enteritidis	1425	5697
SAL_EA9438AA	PNUSAS001473	SRR3137079	2015	10		North America	United States	Enteritidis	Enteritidis	1425	2067
SAL_EA9473AA	E2016001421	SRR3136814	2016	1		North America	United States	Enteritidis (Predicted)	Enteritidis	1425	1056
SAL_EA9474AA	E2016001120	SRR3136813	2016	1		North America	United States	Enteritidis	Enteritidis	1425	613
SAL_EA9475AA	E2016001099	SRR3136812	2016	1		North America	United States	Enteritidis	Enteritidis	1425	5664
SAL_EA9476AA	E2016001071	SRR3136811	2016	1		North America	United States	Enteritidis	Enteritidis	1425	5663
SAL_EA9477AA	E2016001029	SRR3136810	2016	1		North America	United States	Enteritidis	Enteritidis	1425	5662
SAL_EA9491AA	E2015011581	SRR3143631	2015	8		North America	United States	Enteritidis	Enteritidis	27892	5650
SAL_EA9528AA	CVM-N25383	SRR3146666	2010			North America	United States	Enteritidis	Enteritidis	1425	5615
SAL_EA9535AA	CVM-N25372	SRR3146659	2010			North America	United States	Enteritidis	Enteritidis	1425	5608
SAL_EA9536AA	CVM-N23854	SRR3146658	2010			North America	United States	Enteritidis	Enteritidis	1425	5607
SAL_EA9537AA	CVM-N23852	SRR3146657	2010			North America	United States	Enteritidis	Enteritidis	1425	5606
SAL_EA9591AA	PNUSAS001462	SRR3147768	2015	12		North America	United States	Enteritidis	Enteritidis	1425	5566
SAL_EA9600AA	PNUSAS001458	SRR3147759	2015	9		North America	United States	Enteritidis	Enteritidis	1425	5557
SAL_EA9705AA	CMCC50041	GCF_001484025				Europe	Denmark	Enteritidis	Enteritidis	1425	5537
SAL_EA9714AA	FORC_007	GCF_001305235	2014	4		Asia	South Korea	Enteritidis (Predicted)	Enteritidis	1425	5528
SAL_EA9744AA	OLF-SE3-98983-4	SRR1533455	2000			North America	Canada	Enteritidis	Enteritidis	27446	5498
SAL_EA9745AA	OLF-SE2-98984-6	GCF_000750415	2000			North America	Canada	Enteritidis	Enteritidis	27446	5497
SAL_EA9751AA	OLF-SE6	SRR1533592	2010			North America	Canada	Enteritidis	Enteritidis	1425	5491

SAL_EA9752AA	OLF-SE1	SRR1533150	2010			North America	Canada	Enteritidis	Enteritidis	1425	5490
SAL_EA9753AA	OLF-SE5	GCF_000750455	2008			North America	Canada	Enteritidis	Enteritidis	1425	5489
SAL_EA9754AA	OLF-SE4	SRR1533456	2009			North America	Canada	Enteritidis	Enteritidis	1425	5488
SAL_EA9755AA	OLF-SE11	SRR1533835	2010			North America	Canada	Enteritidis	Enteritidis	1425	5487
SAL_EA9756AA	OLF-SE10	GCF_000750335	2010			North America	Canada	Enteritidis	Enteritidis	1425	5486
SAL_EA9757AA	OLF-SE9	SRR1533833	2009			North America	Canada	Enteritidis	Enteritidis	49191	5485
SAL_EA9758AA	OLF-SE8	GCF_000750255	2010			North America	Canada	Enteritidis	Enteritidis	1425	5484
SAL_EA9759AA	OLF-SE7	SRR1533624	2010			North America	Canada	Enteritidis	Enteritidis	1425	5483
SAL_EA9765AA	Durban	GCF_000612325	2013	7		Africa	South Africa	Enteritidis	Enteritidis	3888	5477
SAL_EA9783AA	P125109 / NCTC 13349	GCF_000009505						Enteritidis	Enteritidis	1425	5459
SAL_EA9786AA	E2016001566	SRR3151856	2016	1		North America	United States	Enteritidis	Enteritidis	1425	5456
SAL_EA9787AA	E2016001533	SRR3151855	2016	1		North America	United States	Enteritidis	Enteritidis	1425	5455
SAL_EA9788AA	E2016001504	SRR3151854	2016	1		North America	United States	Enteritidis	Enteritidis	1425	5454
SAL_EA9789AA	E2016001477	SRR3151853	2016	1		North America	United States	Enteritidis	Enteritidis	1425	5453
SAL_EA9790AA	E2016001474	SRR3151852	2016	1		North America	United States	Enteritidis	Enteritidis	27796	5452
SAL_EA9792AA	CVM-N23714	SRR3151802	2010			North America	United States	Typhimurium var. O5 - (Copenhagen)	Enteritidis	1425	5450
SAL_EA9900AA	FDA00000848	SRR3170570	2005	6		Asia	China	Enteritidis	Enteritidis	1425	5389
SAL_EA9908AA	2015K-1300	SRR3157769	2015	9		North America	United States	Enteritidis	Enteritidis	1425	5380
SAL_EA9909AA	2015K-1299	SRR3157768				North America	United States	Enteritidis (Predicted)	Enteritidis	1425	5381
SAL_EA9910AA	2015K-1298	SRR3157767	2015	9		North America	United States	Enteritidis	Enteritidis	1425	5380
SAL_EA9911AA	2015K-1297	SRR3157766	2015	9		North America	United States	Enteritidis	Enteritidis	1425	5380
SAL_EA9974AA	6282_96-sc-2416994	ERR1251781						Enteritidis (Predicted)	Enteritidis	1425	41131
SAL_FA0045AA	6148_1993-sc-2416919	ERR1251710						Enteritidis (Predicted)	Enteritidis	1425	41147
SAL_FA0049AA	3293_1993-sc-2416915	ERR1251706						Enteritidis (Predicted)	Enteritidis	1425	5259
SAL_FA0053AA	5351_1995-sc-2416911	ERR1251702						Enteritidis (Predicted)	Enteritidis	1425	41146
SAL_FA0461AA	Enteritidis	SRR3175437	2011	1		North America	United States	Enteritidis	Enteritidis	1425	4904
SAL_FA0463AA	S/20141173	traces-0ayzGfB	2014	8	18	Europe	United Kingdom	Enteritidis	Enteritidis	3888	4902
SAL_FA0472AA	S/20141063	traces-0mLmkJS	2014	7	25	Europe	United Kingdom	Enteritidis	Enteritidis	3888	4894
SAL_FA0473AA	S/20151576	traces-0KoIdrg	2015	11	2	Europe	United Kingdom	Enteritidis	Enteritidis	3888	4893
SAL_FA0475AA	S/20151675	traces-0WBnHwO	2015	11	21	Europe	United Kingdom	Enteritidis	Enteritidis	3888	4892
SAL_FA0476AA	S/20151717	traces-0ctTrOn	2015	12	4	Europe	United Kingdom	Enteritidis	Enteritidis	3888	4891
SAL_FA0477AA	S/20151730	traces-0kyZTGI	2015	12	8	Europe	United Kingdom	Enteritidis	Enteritidis	3888	4890
SAL_FA0479AA	S/20151764	traces-0FRkjLH	2015	12	12	Europe	United Kingdom	Enteritidis	Enteritidis	3888	4889
SAL_FA0480AA	S/20151765	traces-0xQjxHw	2015	12	18	Europe	United Kingdom	Enteritidis	Enteritidis	3888	4888
SAL_FA0481AA	S/20151766	traces-0mwJlEg	2015	12	18	Europe	United Kingdom	Enteritidis	Enteritidis	3888	4887
SAL_FA0482AA	S/20160045	traces-0iyxVeZ	2016	1	8	Europe	United Kingdom	Enteritidis	Enteritidis	3888	4886
SAL_FA0483AA	S/20160073	traces-0lyfCNS	2016	1	8	Europe	United Kingdom	Enteritidis	Enteritidis	3888	4885
SAL_FA0484AA	S/20160107	traces-0TYvnWT	2016	1	19	Europe	United Kingdom	Enteritidis	Enteritidis	3888	4884
SAL_FA0485AA	S/20151457	traces-0KzGWnR	2015	10	12	Europe	United Kingdom	Enteritidis	Enteritidis	1425	4883
SAL_FA0486AA	S/20151513	traces-0yaeAvE	2015	10	23	Europe	United Kingdom	Enteritidis	Enteritidis	1425	4882
SAL_FA0535AA	FDA00009808	SRR3173719	2016			Asia	Bangladesh	Enteritidis (Predicted)	Enteritidis	1425	4836
SAL_FA0606AA	PNUSAS001520	SRR3171696				North America	United States	Enteritidis (Predicted)	Enteritidis	1425	4774
SAL_FA0623AA	E2016001974	SRR3173548	2016	2		North America	United States	Enteritidis	Enteritidis	1425	4757
SAL_FA0624AA	E2016001927	SRR3173547	2016	2		North America	United States	Enteritidis	Enteritidis	1425	4756
SAL_FA0625AA	E2016001746	SRR3173546	2016	1		North America	United States	Enteritidis	Enteritidis	1425	3254
SAL_FA0626AA	E2016001727	SRR3173545	2016	1		North America	United States	Enteritidis	Enteritidis	34197	4755
SAL_FA0627AA	E2016001531	SRR3173544	2016	1		North America	United States	Enteritidis	Enteritidis	1425	3254
SAL_FA0707AA	WAPHL-SAL-A01133	SRR3182552	2009			North America	United States	Enteritidis	Enteritidis	1425	4701
SAL_FA0711AA	WAPHL-SAL-A01129	SRR3182548	2009			North America	United States	Enteritidis	Enteritidis	1425	4697
SAL_FA0726AA	HIY0101	SRR3182470	2010	7		North America	United States	Enteritidis	Enteritidis	1425	4684
SAL_FA0791AA	AK1131900079	SRR3182265	2011	11		North America	United States	Enteritidis	Enteritidis	1425	4627
SAL_FA0792AA	AZ-TG79532	SRR3182236	2015			North America	United States	Enteritidis (Predicted)	Enteritidis	1425	4625
SAL_FA0793AA	AZ-TG79528	SRR3182235	2015			North America	United States	Enteritidis (Predicted)	Enteritidis	1425	4626
SAL_FA0794AA	AZ-TG79524	SRR3182234	2015			North America	United States	Enteritidis (Predicted)	Enteritidis	1425	4625
SAL_FA0802AA	FDA00009902	SRR3182180	2016	2		North America	United States	Enteritidis (Predicted)	Enteritidis	1425	4618
SAL_FA0803AA	FDA00009901	SRR3182179	2016	2		North America	United States	Enteritidis (Predicted)	Enteritidis	1425	4617
SAL_FA0830AA	2016K-0074	SRR3184400	2015	11		North America	United States	Enteritidis	Enteritidis	1425	3680
SAL_FA0831AA	2016K-0075	SRR3184399	2015	10		North America	United States	Enteritidis	Enteritidis	1425	3680
SAL_FA0832AA	2016K-0076	SRR3184398	2015	11		North America	United States	Enteritidis	Enteritidis	1425	4600

SAL_FA0839AA	FSIS1605663	SRR3184347	2016			North America	United States	Enteritidis	Enteritidis	1425	4594
SAL_FA0843AA	2016K-0077	SRR3184322				North America	United States	Enteritidis (Predicted)	Enteritidis	1425	4590
SAL_FA0847AA	2016K-0095	SRR3184317	2015	10		North America	United States	Enteritidis	Enteritidis	1425	4586
SAL_FA0848AA	2016K-0073	SRR3184316	2015	10		North America	United States	Enteritidis	Enteritidis	1425	3680
SAL_FA0851AA	FSIS1605665	SRR3184278	2016			North America	United States	Enteritidis	Enteritidis	1425	4583
SAL_FA0861AA	CVM-N26081	SRR3184189	2010			North America	United States	Enteritidis	Enteritidis	1425	4573
SAL_FA0862AA	CVM-N26080	SRR3184188	2010			North America	United States	Typhimurium var. O5 - (Copenhagen)	Enteritidis	1425	4572
SAL_FA0863AA	CVM-N26077	SRR3184187	2010			North America	United States	Enteritidis	Enteritidis	1425	4571
SAL_FA0884AA	E2016002268	SRR3184147	2016	2		North America	United States	Enteritidis	Enteritidis	1425	3254
SAL_FA0885AA	E2016002209	SRR3184144	2016	2		North America	United States	Enteritidis	Enteritidis	1425	4551
SAL_FA1093AA	PNUSAS001519	SRR3185195	2015	11		North America	United States	Enteritidis	Enteritidis	1425	3680
SAL_FA1094AA	PNUSAS001518	SRR3185194	2015	10		North America	United States	Enteritidis	Enteritidis	1425	3680
SAL_FA1106AA	PNUSAS001577	SRR3185114	2016	1		North America	United States	Enteritidis (Predicted)	Enteritidis	1425	4342
SAL_FA1144AA	E2016002349	SRR3188272	2016	2		North America	United States	Enteritidis	Enteritidis	1425	4307
SAL_FA1145AA	E2016002341	SRR3188269	2016	2		North America	United States	Enteritidis	Enteritidis	1425	4306
SAL_FA1159AA	S/20151247	traces-0jeACSe	2015	9	4	Europe	United Kingdom	Enteritidis	Enteritidis	3888	4292
SAL_FA1160AA	S/20151254	traces-0MHnrau	2015	9	7	Europe	United Kingdom	Enteritidis	Enteritidis	3888	3621
SAL_FA1162AA	S/20151384	traces-0VxhlKR	2015	9	30	Europe	United Kingdom	Enteritidis	Enteritidis	3888	4290
SAL_FA1163AA	S/20151412	traces-0OQHYTl	2015	10	7	Europe	United Kingdom	Enteritidis	Enteritidis	1425	1990
SAL_FA1164AA	S/20151453	traces-0kCuBXb	2015	10	13	Europe	United Kingdom	Enteritidis	Enteritidis	1425	1990
SAL_FA1165AA	S/20151518	traces-0rviUXH	2015	10	26	Europe	United Kingdom	Enteritidis	Enteritidis	3888	2037
SAL_FA1166AA	S/20151709	traces-0OrwHjf	2015	12	3	Europe	United Kingdom	Enteritidis	Enteritidis	3888	3623
SAL_FA1167AA	S/20151729	traces-0orNCFv	2015	12	9	Europe	United Kingdom	Enteritidis	Enteritidis	3888	2037
SAL_FA1168AA	S/20160097	traces-0KXoxkq	2016	1	16	Europe	United Kingdom	Enteritidis	Enteritidis	3888	2002
SAL_FA1172AA	ADRDL-105825-10-4	SRR3191589	2010	11		North America	United States	Enteritidis	Enteritidis	1425	4286
SAL_FA1269AA	CVM-N18564	SRR3194572	2008			North America	United States	Enteritidis	Enteritidis	1425	4196
SAL_FA1270AA	CVM-N18563	SRR3194571	2008			North America	United States	Enteritidis	Enteritidis	1425	4195
SAL_FA1277AA	CVM-N18556	SRR3194564	2008			North America	United States	Enteritidis	Enteritidis	1425	4188
SAL_FA1279AA	AZ-TG79648	SRR3194562	2015			North America	United States	Enteritidis (Predicted)	Enteritidis	26775	4186
SAL_FA1280AA	AZ-TG79644	SRR3194560	2015			North America	United States	Enteritidis (Predicted)	Enteritidis	26775	4185
SAL_FA1287AA	AZ-TG79640	SRR3198738	2015			North America	United States	Enteritidis (Predicted)	Enteritidis	1425	4178
SAL_FA1288AA	AZ-TG79636	SRR3198737	2015			North America	United States	Enteritidis (Predicted)	Enteritidis	1425	4177
SAL_FA1289AA	AZ-TG79632	SRR3198736	2015			North America	United States	Enteritidis (Predicted)	Enteritidis	1425	4176
SAL_FA1294AA	AZ-TG79596	SRR3198728	2015			North America	United States	Enteritidis (Predicted)	Enteritidis	1425	4174
SAL_FA1298AA	AZ-TG79548	SRR3198710	2015			North America	United States	Enteritidis (Predicted)	Enteritidis	1425	4169
SAL_FA1299AA	AZ-TG79544	SRR3198702	2015			North America	United States	Enteritidis (Predicted)	Enteritidis	1425	4169
SAL_FA1300AA	AZ-TG79540	SRR3198700	2015			North America	United States	Enteritidis (Predicted)	Enteritidis	1425	4169
SAL_FA1301AA	AZ-TG79536	SRR3198699	2015			North America	United States	Enteritidis (Predicted)	Enteritidis	1425	4169
SAL_FA1302AA	AZ-TG92656	SRR3198652	2016	1		North America	United States	Enteritidis	Enteritidis	1425	4170
SAL_FA1303AA	AZ-TG92652	SRR3198651	2015	5		North America	United States	Enteritidis	Enteritidis	1425	4169
SAL_FA1304AA	AZ-TG92648	SRR3198650	2015	4		North America	United States	Enteritidis	Enteritidis	1425	4168
SAL_FA1305AA	I2016002520	SRR3198637	2016	2		North America	United States	Enteritidis	Enteritidis	1425	4167
SAL_FA1306AA	E2016002593	SRR3217343	2016	2		North America	United States	Enteritidis	Enteritidis	1425	4166
SAL_FA1307AA	E2016002591	SRR3198635	2016	2		North America	United States	Enteritidis	Enteritidis	1425	166
SAL_FA1308AA	E2016002583	SRR3198634	2016	2		North America	United States	Enteritidis	Enteritidis	1425	4165
SAL_FA1309AA	E2016002547	SRR3198633	2016	2		North America	United States	Enteritidis	Enteritidis	34197	4164
SAL_FA1322AA	FSIS1605765	SRR3199700	2016			North America	United States	Enteritidis	Enteritidis	1425	4151
SAL_FA1376AA	H15042077201	traces-0LZsIJa				Europe	United Kingdom	Enteritidis	Enteritidis	3888	4110
SAL_FA1377AA	H15372061101	traces-0yqFzyW				Europe	United Kingdom	Enteritidis	Enteritidis	3888	2002
SAL_FA1378AA	H15376029605	traces-0vatOYX				Europe	United Kingdom	Enteritidis	Enteritidis	3888	2045
SAL_FA1379AA	1091200407	traces-0WLUzPw				Europe	Netherlands	Enteritidis	Enteritidis	3941	4109
SAL_FA1380AA	1091201969	traces-0xgSgqt				Europe	Netherlands	Enteritidis	Enteritidis	3888	4108
SAL_FA1381AA	1091301454	traces-0Kosqda				Europe	Netherlands	Enteritidis	Enteritidis	3941	4107
SAL_FA1382AA	1091302865	traces-0AxCfPK				Europe	Netherlands	Enteritidis	Enteritidis	1425	4106
SAL_FA1383AA	1091401736	traces-0IgLgxn				Europe	Netherlands	Enteritidis	Enteritidis	3941	4105
SAL_FA1384AA	1091500827	traces-0fIffBu				Europe	Netherlands	Enteritidis	Enteritidis	3888	3621
SAL_FA1385AA	1091501188	traces-0raYDbZ				Europe	Netherlands	Enteritidis	Enteritidis	3888	3621
SAL_FA1386AA	1091500894	traces-0ELbjJn				Europe	Netherlands	Enteritidis	Enteritidis	3888	3621
SAL_FA1387AA	1091501193	traces-0xPcudp				Europe	Netherlands	Enteritidis	Enteritidis	3888	3630

SAL_FA1388AA	1091501248	traces-0rpldUH			Europe	Netherlands	Enteritidis	Enteritidis	3888	3630
SAL_FA1389AA	1091501424	traces-0SbvFxt			Europe	Netherlands	Enteritidis	Enteritidis	3888	4104
SAL_FA1390AA	1091501466	traces-0niZWzC			Europe	Netherlands	Enteritidis	Enteritidis	3888	3628
SAL_FA1391AA	1091501831	traces-0jCTslZ			Europe	Netherlands	Enteritidis	Enteritidis	3888	4103
SAL_FA1392AA	1091501844	traces-0jIpShs			Europe	Netherlands	Enteritidis	Enteritidis	3888	3621
SAL_FA1393AA	1091501849	traces-0WXuuTY			Europe	Netherlands	Enteritidis	Enteritidis	3888	2002
SAL_FA1394AA	1091501884	traces-0oVxuEz			Europe	Netherlands	Enteritidis	Enteritidis	3888	4102
SAL_FA1395AA	1091501906	traces-0wXjWkC			Europe	Netherlands	Enteritidis	Enteritidis	3888	3621
SAL_FA1396AA	1091502012	traces-0jPtslz			Europe	Netherlands	Enteritidis	Enteritidis	3888	4101
SAL_FA1397AA	1091502075	traces-0cxExGv			Europe	Netherlands	Enteritidis	Enteritidis	3888	4101
SAL_FA1398AA	1091502499	traces-0wyGyZf			Europe	Netherlands	Enteritidis	Enteritidis	3888	4100
SAL_FA1399AA	114213	traces-0vLQyfX			Europe	Finland	Enteritidis	Enteritidis	3888	2002
SAL_FA1411AA	FSIS1605964	SRR3204218	2016		North America	United States	Enteritidis	Enteritidis	1425	4088
SAL_FA1412AA	FSIS1605965	SRR3204217	2016		North America	United States	Enteritidis	Enteritidis	1425	4089
SAL_FA1413AA	FSIS1605963	SRR3204216	2016		North America	United States	Enteritidis	Enteritidis	1425	4088
SAL_FA1414AA	FSIS1605961	SRR3204215	2016		North America	United States	Enteritidis	Enteritidis	1425	4084
SAL_FA1415AA	FSIS1605960	SRR3204214	2016		North America	United States	Enteritidis	Enteritidis	1425	4084
SAL_FA1416AA	FSIS1605966	SRR3204212	2016		North America	United States	Enteritidis	Enteritidis	1425	4087
SAL_FA1417AA	FSIS1605962	SRR3204211	2016		North America	United States	Enteritidis	Enteritidis	1425	4084
SAL_FA1418AA	FSIS1605956	SRR3204210	2016		North America	United States	Enteritidis	Enteritidis	1425	4086
SAL_FA1419AA	FSIS1605954	SRR3204209	2016		North America	United States	Enteritidis	Enteritidis	1425	915
SAL_FA1420AA	FSIS1605953	SRR3204208	2016		North America	United States	Enteritidis	Enteritidis	1425	915
SAL_FA1421AA	FSIS1605952	SRR3204207	2016		North America	United States	Enteritidis	Enteritidis	1425	4085
SAL_FA1422AA	FSIS1605958	SRR3204206	2016		North America	United States	Enteritidis	Enteritidis	1425	4084
SAL_FA1423AA	FSIS1605959	SRR3204026	2016		North America	United States	Enteritidis	Enteritidis	1425	4084
SAL_FA1424AA	FSIS1605955	SRR3204025	2016		North America	United States	Enteritidis	Enteritidis	1425	915
SAL_FA1425AA	FSIS1605957	SRR3204023	2016		North America	United States	Enteritidis	Enteritidis	1425	4083
SAL_FA1426AA	FDA00004015	SRR3205955	2012	4	North America	United States	Enteritidis	Enteritidis	26543	4082
SAL_FA1427AA	FDA00004016	SRR3205954	2012	5	Asia	Pakistan	Enteritidis	Enteritidis	1425	4081
SAL_FA1428AA	FDA00004017	SRR3205953	2012	5	Asia	Pakistan	Enteritidis	Enteritidis	1425	4080
SAL_FA1469AA	PNUSAS001644	SRR3205703	2016	2	North America	United States	Enteritidis	Enteritidis	1425	1201
SAL_FA1470AA	PNUSAS001643	SRR3205702	2016	2	North America	United States	Enteritidis	Enteritidis	1425	1201
SAL_FA1471AA	PNUSAS001642	SRR3205701			North America	United States	Enteritidis (Predicted)	Enteritidis	1425	1201
SAL_FA1480AA	PNUSAS001517	SRR3205692	2015	12	North America	United States	Enteritidis	Enteritidis	1425	4034
SAL_FA1482AA	Enteritidis	SRR3205690	2011	1	North America	United States	Enteritidis	Enteritidis	1425	4032
SAL_FA1489AA	Enteritidis	SRR3205683	2010	7	North America	United States	Enteritidis (Predicted)	Enteritidis	1425	4025
SAL_FA1496AA	PNUSAS001516	SRR3205676			North America	United States	Enteritidis (Predicted)	Enteritidis	1425	4019
SAL_FA1497AA	PNUSAS001515	SRR3205675			North America	United States	Enteritidis (Predicted)	Enteritidis	1425	4018
SAL_FA1498AA	PNUSAS001514	SRR3205674			North America	United States	Enteritidis (Predicted)	Enteritidis	1425	4017
SAL_FA1499AA	NY62259358	SRR3206638	2016	2	North America	United States	Enteritidis	Enteritidis	1425	4016
SAL_FA1500AA	NY62259321	SRR3206637	2016	2	North America	United States	Enteritidis	Enteritidis	1425	4015
SAL_FA1501AA	NY62259097	SRR3206636	2016	1	North America	United States	Enteritidis	Enteritidis	1425	4014
SAL_FA1506AA	NY-N20133	SRR3210664	2009		North America	United States	Enteritidis	Enteritidis	1425	4011
SAL_FA1515AA	NY-N20124	SRR3210550	2009		North America	United States	Enteritidis	Enteritidis	1425	4004
SAL_FA1518AA	NY-N20121	SRR3210652	2009		North America	United States	Enteritidis	Enteritidis	1425	4001
SAL_FA1520AA	NY-N20119	SRR3210650	2009		North America	United States	Enteritidis	Enteritidis	1425	3999
SAL_FA1537AA	WAPHL_SAL-A00846	SRR3210452	2006		North America	United States	Enteritidis	Enteritidis	1425	3994
SAL_FA1609AA	E2016002697	SRR3214118	2016	2	North America	United States	Enteritidis	Enteritidis	1425	3929
SAL_FA1610AA	E2016002658	SRR3214106	2016	2	North America	United States	Enteritidis	Enteritidis	1425	3254
SAL_FA1633AA	FSIS1605968	SRR3209764	2015		North America	United States	Enteritidis	Enteritidis	1425	3911
SAL_FA1678AA	PNUSAS001721	SRR3216568			North America	United States	Enteritidis (Predicted)	Enteritidis	1425	3871
SAL_FA1679AA	PNUSAS001720	SRR3216567			North America	United States	Enteritidis (Predicted)	Enteritidis	1425	3870
SAL_FA1689AA	2016K-0114	SRR3216127	2015	12	North America	United States		Enteritidis	1425	3680
SAL_FA1737AA	HIY0325	SRR3214193	2011	1	North America	United States	Enteritidis	Enteritidis	1425	3820
SAL_FA1741AA	HIY0313	SRR3214156	2011	1	North America	United States	Enteritidis	Enteritidis	1425	3816
SAL_FA1746AA	HIY0332	SRR3215265	2011	1	North America	United States	Enteritidis	Enteritidis	1425	3813
SAL_FA1750AA	CFSAN047179	SRR3215232	2016	2	North America	United States	Enteritidis	Enteritidis	1425	3811
SAL_FA1751AA	CFSAN047178	SRR3215231	2016	2	North America	United States	Enteritidis	Enteritidis	1425	3810
SAL_FA1752AA	CFSAN047176	SRR3215229	2016	2	North America	United States	Enteritidis	Enteritidis	1425	3809

SAL_FA1753AA	CFSAN047177	SRR3215230	2016	2		North America	United States	Enteritidis	Enteritidis	1425	3808
SAL_FA1754AA	CFSAN047175	SRR3215228	2016	2		North America	United States	Enteritidis	Enteritidis	1425	3807
SAL_FA1865AA	H14252044805	traces-0wYwXTP				Europe	United Kingdom	Enteritidis	Enteritidis	3888	3718
SAL_FA1893AA	FSIS1605818	SRR3225830	2016			North America	United States	Enteritidis	Enteritidis	1425	3690
SAL_FA1904AA	2016K-0113	SRR3231366	2015	10		North America	United States	Serotype Pending	Enteritidis	1425	3680
SAL_FA1952AA	H14306020905	traces-0XQLSgo				Europe	United Kingdom	Enteritidis	Enteritidis	3888	3643
SAL_FA1953AA	H14312043801	traces-0JLoago				Europe	United Kingdom	Enteritidis	Enteritidis	3888	3643
SAL_FA1954AA	H14316038101	traces-0mrszXs				Europe	United Kingdom	Enteritidis	Enteritidis	3888	3643
SAL_FA1955AA	H14352045501	traces-0engMQs				Europe	United Kingdom	Enteritidis	Enteritidis	3888	3621
SAL_FA1956AA	H14354090001	traces-0nQglAU				Europe	United Kingdom	Enteritidis	Enteritidis	3888	3642
SAL_FA1957AA	H14422064201	traces-0ouGCEz				Europe	United Kingdom	Enteritidis	Enteritidis	3888	3621
SAL_FA1958AA	H14444076001	traces-0IyFcHD						Enteritidis	Enteritidis	3888	3641
SAL_FA1959AA	H14386066501	traces-0zHptIl						Enteritidis	Enteritidis	3888	3640
SAL_FA1960AA	H14422065001	traces-0RHhKNf						Enteritidis	Enteritidis	3888	3639
SAL_FA1961AA	H14470042801	traces-0icwGHr				Europe	United Kingdom	Enteritidis	Enteritidis	3888	3621
SAL_FA1962AA	H14494056401	traces-0ulLPhU				Europe	United Kingdom	Enteritidis	Enteritidis	3888	3630
SAL_FA1963AA	H15032069901	traces-0IhemcD				Europe	United Kingdom	Enteritidis	Enteritidis	3888	3638
SAL_FA1964AA	H15032070001	traces-0ydCprd				Europe	United Kingdom	Enteritidis	Enteritidis	3888	3637
SAL_FA1965AA	H15032070101	traces-0tlKLxI						Enteritidis	Enteritidis	3888	3635
SAL_FA1966AA	H15042077601	traces-0edoGZB				Europe	United Kingdom	Enteritidis	Enteritidis	3888	3636
SAL_FA1967AA	H15044067501	traces-0CXaLzc						Enteritidis	Enteritidis	3888	3635
SAL_FA1968AA	H15192051601	traces-0eGyllR				Europe	United Kingdom	Enteritidis	Enteritidis	3888	3634
SAL_FA1969AA	H15224048305	traces-0RhRwHc				Europe	United Kingdom	Enteritidis	Enteritidis	3888	3633
SAL_FA1970AA	H15308062501	traces-0jaEFvw				Europe	United Kingdom	Enteritidis	Enteritidis	3888	3628
SAL_FA1971AA	H15312063501	traces-0iBerUR				Europe	United Kingdom	Enteritidis	Enteritidis	3888	3632
SAL_FA1972AA	H15322048701	traces-0sVADYb				Europe	United Kingdom	Enteritidis	Enteritidis	3888	3631
SAL_FA1973AA	H15328043601	traces-0xaRlfT				Europe	United Kingdom	Enteritidis	Enteritidis	3888	3630
SAL_FA1974AA	H15328043701	traces-0QBgkFW						Enteritidis	Enteritidis	3888	3629
SAL_FA1975AA	H15320043005	traces-0lzWBlO				Europe	United Kingdom	Enteritidis	Enteritidis	3888	3628
SAL_FA1976AA	H15356015101	traces-0LCdwLj				Europe	United Kingdom	Enteritidis	Enteritidis	3888	2002
SAL_FA1977AA	H15370048401	traces-0mVriFT				Europe	United Kingdom	Enteritidis	Enteritidis	3888	2002
SAL_FA1978AA	H15374051701	traces-0WcmDVt				Europe	United Kingdom	Enteritidis	Enteritidis	3888	3627
SAL_FA1979AA	H15374051801	traces-0jRwWtw				Europe	United Kingdom	Enteritidis	Enteritidis	3888	2002
SAL_FA1980AA	H15388050601	traces-0CaNDVn				Europe	United Kingdom	Enteritidis	Enteritidis	3888	2002
SAL_FA1981AA	H15392042801	traces-0eCtmym				Europe	United Kingdom	Enteritidis	Enteritidis	3888	2002
SAL_FA1982AA	H15412042801	traces-0JuHwNf				Europe	United Kingdom	Enteritidis	Enteritidis	3888	2037
SAL_FA1983AA	H15418062005	traces-0xWCqYJ				Europe	United Kingdom	Enteritidis	Enteritidis	3888	2179
SAL_FA1984AA	H15422079405	traces-0IlIyDU				Europe	United Kingdom	Enteritidis	Enteritidis	3888	2002
SAL_FA1985AA	H15470034701	traces-0SkXQVp				Europe	United Kingdom	Enteritidis	Enteritidis	3888	3626
SAL_FA1986AA	H15472054001	traces-0WFNDzJ				Europe	United Kingdom	Enteritidis	Enteritidis	3888	2002
SAL_FA1987AA	H15484056501	traces-0XEsWLg						Enteritidis	Enteritidis	3888	3625
SAL_FA1988AA	H15488060801	traces-0qhlAVh				Europe	United Kingdom	Enteritidis	Enteritidis	3888	2037
SAL_FA1989AA	H15496055001	traces-0cnPqrm				Europe	United Kingdom	Enteritidis	Enteritidis	3888	3621
SAL_FA1990AA	H15508030801	traces-0MDEJnR				Europe	United Kingdom	Enteritidis	Enteritidis	3888	3621
SAL_FA1991AA	H15510049001	traces-0FoiTNF				Europe	United Kingdom	Enteritidis	Enteritidis	3888	3624
SAL_FA1992AA	H15512050601	traces-0kwvbjO				Europe	United Kingdom	Enteritidis	Enteritidis	3888	3623
SAL_FA1993AA	H15518052901	traces-0zUVTBl				Europe	United Kingdom	Enteritidis	Enteritidis	3888	3621
SAL_FA1994AA	H15534109101	traces-0pXhbCy				Europe	United Kingdom	Enteritidis	Enteritidis	3888	3622
SAL_FA1995AA	H16022067101	traces-0eHzSBo				Europe	United Kingdom	Enteritidis	Enteritidis	3888	3621
SAL_FA1996AA	H16022067901	traces-0YPYcKH				Europe	United Kingdom	Enteritidis	Enteritidis	3888	3621
SAL_FA1997AA	H16022068001	traces-0rUQvKK				Europe	United Kingdom	Enteritidis	Enteritidis	3888	2002
SAL_FA1998AA	H16022068101	traces-0nmuYpa				Europe	United Kingdom	Enteritidis	Enteritidis	3888	3621
SAL_FA1999AA	H16022068201	traces-0kpIDPe				Europe	United Kingdom	Enteritidis	Enteritidis	3888	2002
SAL_FA2107AA	1091204377	traces-0wpLPmN	2012	11	15	Europe	Netherlands	Enteritidis	Enteritidis	1425	3524
SAL_FA2108AA	1091300815	traces-0WpJdBj	2013	4	11	Europe	Netherlands	Enteritidis	Enteritidis	1425	3523
SAL_FA2109AA	1091300816	traces-0gRShgM	2013	4	11	Europe	Netherlands	Enteritidis	Enteritidis	1425	3522
SAL_FA2110AA	1091302548	traces-0niaqui	2013	11	14	Europe	Netherlands	Enteritidis	Enteritidis	3888	3521
SAL_FA2111AA	1091302549	traces-0phPOtJ	2013	11	14	Europe	Netherlands	Enteritidis	Enteritidis	3888	3520
SAL_FA2112AA	1091302550	traces-0zcQHWQ	2013	11	14	Europe	Netherlands	Enteritidis	Enteritidis	3888	3519

SAL_FA2113AA	1091302777	traces-0SIKcAU	2013	12	12	Europe	Netherlands	Enteritidis	Enteritidis	3888	3518
SAL_FA2114AA	1091302778	traces-0ghzaUi	2013	12	12	Europe	Netherlands	Enteritidis	Enteritidis	3888	3517
SAL_FA2115AA	1091502338	traces-0JuIuFd	2015	11	30	Europe	Netherlands	Enteritidis	Enteritidis	3888	3516
SAL_FA2184AA	PNUSAS001719	SRR3240353				North America	United States	Enteritidis (Predicted)	Enteritidis	1425	3461
SAL_FA2185AA	PNUSAS001718	SRR3240352				North America	United States	Enteritidis (Predicted)	Enteritidis	1425	1201
SAL_FA2186AA	PHUSAS001717	SRR3240351				North America	United States	Enteritidis (Predicted)	Enteritidis	1425	3460
SAL_FA2187AA	PNUSAS001716	SRR3240350				North America	United States	Enteritidis (Predicted)	Enteritidis	1425	3459
SAL_FA2188AA	PNUSAS001715	SRR3240349				North America	United States	Enteritidis (Predicted)	Enteritidis	1425	1201
SAL_FA2204AA	FSIS1605967	SRR3241322	2015			North America	United States	Enteritidis	Enteritidis	1425	3445
SAL_FA2239AA	CVM-N19747	SRR3242211	2008			North America	United States	Enteritidis	Enteritidis	1425	3417
SAL_FA2254AA	I2016002162	SRR3242191	2016	2		North America	United States	Enteritidis	Enteritidis	1425	3404
SAL_FA2307AA	FDA00003975	SRR3271929	2012	1		North America	United States	Enteritidis	Enteritidis	1425	3360
SAL_FA2308AA	FDA00003976	SRR3271928	2012	1		North America	United States	Enteritidis	Enteritidis	1425	3359
SAL_FA2309AA	FDA00003977	SRR3271927	2012	1		North America	United States	Enteritidis	Enteritidis	1425	3358
SAL_FA2332AA	CVM-N23570	SRR3271882	2009			North America	United States	I 9,12:Nonmotile	Enteritidis	1425	3337
SAL_FA2335AA	I2016003617	SRR3271880	2016	3		North America	United States	Enteritidis	Enteritidis	1425	3334
SAL_FA2336AA	E2016003746	SRR3271879	2016	3		North America	United States	Enteritidis	Enteritidis	1425	3333
SAL_FA2337AA	E2016003743	SRR3271878	2016	3		North America	United States	Enteritidis	Enteritidis	1425	3332
SAL_FA2338AA	E2016003725	SRR3271877	2016	3		North America	United States	Enteritidis	Enteritidis	1425	3331
SAL_FA2339AA	E2016003724	SRR3271876	2016	3		North America	United States	Enteritidis	Enteritidis	1425	3330
SAL_FA2426AA	PNUSAS001736	SRR3277290				North America	United States	Enteritidis (Predicted)	Enteritidis	1425	3254
SAL_FA2451AA	S/20151604	traces-0nnRFex	2015	11	4	Europe	United Kingdom	Enteritidis	Enteritidis	3888	3230
SAL_FA2452AA	S/20160151	traces-0lVsVXF	2016	2	3	Europe	United Kingdom	Enteritidis	Enteritidis	3888	3229
SAL_FA2453AA	S/20160163	traces-0DYCXIJ	2016	2	10	Europe	United Kingdom	Enteritidis	Enteritidis	3888	3228
SAL_FA2454AA	S/20160169	traces-0shDhps	2016	2	17	Europe	United Kingdom	Enteritidis	Enteritidis	3888	3227
SAL_FA2467AA	CFSAN047858	SRR3286887	2016	3		North America	United States	Enteritidis (Predicted)	Enteritidis	1425	3214
SAL_FA2468AA	CFSAN047857	SRR3286885	2016	3		North America	United States	Enteritidis (Predicted)	Enteritidis	1425	3213
SAL_FA2534AA	PNUSAS001765	SRR3289848				North America	United States	Enteritidis (Predicted)	Enteritidis	26775	3141
SAL_FA2539AA	PNUSAS001761	SRR3289843	2016	2		North America	United States	Enteritidis	Enteritidis	1425	3136
SAL_FA2543AA	PNUSAS001759	SRR3289839	2016	1		North America	United States	Enteritidis	Enteritidis	1425	3132
SAL_FA2562AA	CVM-N19825	SRR3289793	2008			North America	United States	Enteritidis	Enteritidis	1425	3173
SAL_FA2566AA	CVM-N19818	SRR3289778	2008			North America	United States	Enteritidis	Enteritidis	1425	3169
SAL_FA2570AA	CVM-N19813	SRR3289768	2008			North America	United States	Enteritidis	Enteritidis	1425	3168
SAL_FA2584AA	PNUSAS001784	SRR3290704	2016	2		North America	United States	Enteritidis	Enteritidis	1425	3106
SAL_FA2637AA	CVM-N26930	SRR3312093	2010			North America	United States	Enteritidis	Enteritidis	1425	3066
SAL_FA2648AA	CVM-N26918	SRR3312088	2010			North America	United States	Enteritidis	Enteritidis	1425	3057
SAL_FA2650AA	CVM-N26664	SRR3294526	2010			North America	United States	Enteritidis	Enteritidis	1425	3055
SAL_FA3015AA	CVM N58014	SRR3295598	2015			North America	United States	Enteritidis	Enteritidis	1425	2708
SAL_FA3018AA	CVM N58010	SRR3295595	2015			North America	United States	Enteritidis	Enteritidis	1425	2705
SAL_FA3036AA	CVM N57992	SRR3295577	2015			North America	United States	Enteritidis	Enteritidis	1425	2690
SAL_FA3043AA	CVM N57985	SRR3295570	2015			North America	United States	Enteritidis	Enteritidis	27901	2680
SAL_FA3047AA	CVM N57981	SRR3295566	2015			North America	United States	Enteritidis	Enteritidis	27901	2680
SAL_FA3048AA	CVM N57980	SRR3295565	2015			North America	United States	Enteritidis	Enteritidis	27901	2679
SAL_FA3059AA	CVM N56570	SRR3295554	2015			North America	United States	Enteritidis	Enteritidis	1425	2668
SAL_FA3080AA	CVM N56251	SRR3295533	2015			North America	United States	Enteritidis	Enteritidis	1425	2649
SAL_FA3098AA	CVM N55862	SRR3295515	2015			North America	United States	Enteritidis	Enteritidis	1425	2632
SAL_FA3099AA	CVM N55861	SRR3295514	2015			North America	United States	Enteritidis	Enteritidis	1425	2631
SAL_FA3102AA	CVM N55856	SRR3295511	2015			North America	United States	Enteritidis	Enteritidis	1425	2628
SAL_FA3105AA	CVM N55853	SRR3295508	2015			North America	United States	Enteritidis	Enteritidis	1425	2625
SAL_FA3107AA	CVM N55851	SRR3295506	2015			North America	United States	Enteritidis	Enteritidis	1425	2623
SAL_FA3292AA	E2016004270	SRR3306270	2016	3		North America	United States	Enteritidis	Enteritidis	1425	650
SAL_FA3294AA	E2016003967	SRR3306268	2016	3		North America	United States	Enteritidis	Enteritidis	1425	2520
SAL_FA3300AA	CVM-N23556	SRR3306213	2009			North America	United States	Enteritidis	Enteritidis	1425	2514
SAL_FA3303AA	CVM-N23568	SRR3306209	2009			North America	United States	I 9,12:Nonmotile	Enteritidis	1425	2511
SAL_FA3347AA	161082	SRR3312605	2015	9		Europe	United Kingdom	Enteritidis	Enteritidis	1425	1268
SAL_FA3348AA	161084	SRR3312604	2015	9		Europe	United Kingdom	Enteritidis	Enteritidis	1425	2459
SAL_FA3349AA	153196	SRR3312597	2015	8		Europe	United Kingdom	Enteritidis	Enteritidis	1425	2480
SAL_FA3350AA	159004	SRR3312591	2015	9		Europe	United Kingdom	Enteritidis	Enteritidis	1425	1268
SAL_FA3351AA	161080	SRR3312589	2015	9		Europe	United Kingdom	Enteritidis	Enteritidis	1425	2313

SAL_FA3352AA	163138	SRR3312579	2015	9	Europe	United Kingdom	Enteritidis	Enteritidis	1425	2467
SAL_FA3353AA	109930	SRR3312578	2015	5	Europe	United Kingdom	Enteritidis	Enteritidis	1425	2479
SAL_FA3354AA	168359	SRR3312577	2015	9	Europe	United Kingdom	Enteritidis	Enteritidis	1425	2478
SAL_FA3355AA	118939	SRR3312576	2015	5	Europe	United Kingdom	Enteritidis	Enteritidis	1425	2387
SAL_FA3356AA	107120	SRR3312559	2015	4	Europe	United Kingdom	Enteritidis	Enteritidis	1425	2254
SAL_FA3357AA	107108	SRR3312551	2015	4	Europe	United Kingdom	Enteritidis	Enteritidis	1425	2477
SAL_FA3358AA	166331	SRR3312550	2015	9	Europe	United Kingdom	Enteritidis	Enteritidis	1425	1268
SAL_FA3359AA	113051	SRR3312549	2015	5	Europe	United Kingdom	Enteritidis	Enteritidis	1425	1274
SAL_FA3360AA	164712	SRR3312548	2015	9	Europe	United Kingdom	Enteritidis	Enteritidis	1425	1268
SAL_FA3361AA	157364	SRR3312547	2015	9	Europe	United Kingdom	Enteritidis	Enteritidis	1425	2163
SAL_FA3362AA	146108	SRR3312546	2015	8	Europe	United Kingdom	Enteritidis	Enteritidis	1425	2313
SAL_FA3363AA	161864	SRR3312545	2015	9	Europe	United Kingdom	Enteritidis	Enteritidis	1425	2476
SAL_FA3364AA	109246	SRR3312539	2015	5	Europe	United Kingdom	Enteritidis	Enteritidis	1425	1274
SAL_FA3365AA	159649	SRR3312533	2015	9	Europe	United Kingdom	Enteritidis	Enteritidis	1425	2475
SAL_FA3366AA	161077	SRR3312531	2015	9	Europe	United Kingdom	Enteritidis	Enteritidis	1425	2474
SAL_FA3367AA	146095	SRR3312530	2015	8	Europe	United Kingdom	Enteritidis	Enteritidis	1425	2473
SAL_FA3368AA	164967	SRR3312529	2015	9	Europe	United Kingdom	Enteritidis	Enteritidis	1425	2472
SAL_FA3369AA	122194	SRR3312528	2015	6	Europe	United Kingdom	Enteritidis	Enteritidis	1425	2471
SAL_FA3370AA	157283	SRR3312525	2015	9	Europe	United Kingdom	Enteritidis	Enteritidis	1425	2470
SAL_FA3371AA	161871	SRR3312520	2015	9	Europe	United Kingdom	Enteritidis	Enteritidis	1425	1268
SAL_FA3372AA	116053	SRR3312514	2015	5	Europe	United Kingdom	Enteritidis	Enteritidis	1425	2469
SAL_FA3373AA	107752	SRR3312513	2015	4	Europe	United Kingdom	Enteritidis	Enteritidis	1425	2468
SAL_FA3374AA	168362	SRR3312512	2015	9	Europe	United Kingdom	Enteritidis	Enteritidis	1425	2467
SAL_FA3375AA	146110	SRR3312492	2015	8	Europe	United Kingdom	Enteritidis	Enteritidis	1425	2163
SAL_FA3376AA	161872	SRR3312356	2015	9	Europe	United Kingdom	Enteritidis	Enteritidis	1425	2466
SAL_FA3377AA	107320	SRR3312348	2015	4	Europe	United Kingdom	Enteritidis	Enteritidis	1425	2465
SAL_FA3378AA	152400	SRR3312345	2015	8	Europe	United Kingdom	Enteritidis	Enteritidis	1425	2464
SAL_FA3379AA	159682	SRR3312344	2015	9	Europe	United Kingdom	Enteritidis	Enteritidis	1425	2463
SAL_FA3380AA	128188	SRR3312343	2015	6	Europe	United Kingdom	Enteritidis	Enteritidis	1425	2462
SAL_FA3381AA	137127	SRR3312342	2015	7	Europe	United Kingdom	Enteritidis	Enteritidis	1425	1275
SAL_FA3382AA	161878	SRR3312341	2015	9	Europe	United Kingdom	Enteritidis	Enteritidis	1425	2461
SAL_FA3383AA	137141	SRR3312340	2015	7	Europe	United Kingdom	Enteritidis	Enteritidis	1425	1275
SAL_FA3384AA	153195	SRR3312339	2015	8	Europe	United Kingdom	Enteritidis	Enteritidis	1425	2460
SAL_FA3385AA	163124	SRR3312337	2015	9	Europe	United Kingdom	Enteritidis	Enteritidis	1425	2459
SAL_FA3386AA	159003	SRR3312335	2015	9	Europe	United Kingdom	Enteritidis	Enteritidis	1425	2507
SAL_FA3387AA	161079	SRR3312324	2015	9	Europe	United Kingdom	Enteritidis	Enteritidis	1425	1268
SAL_FA3388AA	137142	SRR3312312	2015	7	Europe	United Kingdom	Enteritidis	Enteritidis	1425	3166
SAL_FA3389AA	143546	SRR3312300	2015	8	Europe	United Kingdom	Enteritidis	Enteritidis	1425	2506
SAL_FA3390AA	153191	SRR3312277	2015	8	Europe	United Kingdom	Enteritidis	Enteritidis	1425	3165
SAL_FA3391AA	130502	SRR3312276	2015	7	Europe	United Kingdom	Enteritidis	Enteritidis	1425	3164
SAL_FA3392AA	152486	SRR3312187	2015	8	Europe	United Kingdom	Enteritidis	Enteritidis	1425	2505
SAL_FA3393AA	159667	SRR3312174	2015	9	Europe	United Kingdom	Enteritidis	Enteritidis	1425	2504
SAL_FA3394AA	106554	SRR3312173	2015	4	Europe	United Kingdom	Enteritidis	Enteritidis	1425	2254
SAL_FA3395AA	152395	SRR3312161	2015	8	Europe	United Kingdom	Enteritidis	Enteritidis	1425	2503
SAL_FA3396AA	117272	SRR3312073	2015	5	Europe	United Kingdom	Enteritidis	Enteritidis	1425	2254
SAL_FA3397AA	130504	SRR3312072	2015	7	Europe	United Kingdom	Enteritidis	Enteritidis	1425	2458
SAL_FA3404AA	131447	SRR3286970	2015	7	Europe	United Kingdom	Enteritidis	Enteritidis	26449	2452
SAL_FA3406AA	130304	SRR3286968	2015	6	Europe	United Kingdom	Enteritidis	Enteritidis	1425	2450
SAL_FA3410AA	131511	SRR3286964	2015	7	Europe	United Kingdom	Enteritidis	Enteritidis	1425	2446
SAL_FA3412AA	118957	SRR3286962	2015	6	Europe	United Kingdom	Enteritidis	Enteritidis	1425	1995
SAL_FA3417AA	128232	SRR3286957	2015	7	Europe	United Kingdom	Enteritidis	Enteritidis	3888	2440
SAL_FA3420AA	129371	SRR3286954	2015	6	Europe	United Kingdom	Enteritidis	Enteritidis	1425	2437
SAL_FA3421AA	131458	SRR3286952	2015	7	Europe	United Kingdom	Enteritidis	Enteritidis	26483	2119
SAL_FA3422AA	129437	SRR3286951	2015	6	Europe	United Kingdom	Enteritidis	Enteritidis	1425	2436
SAL_FA3426AA	128227	SRR3286947	2015	7	Europe	United Kingdom	Enteritidis	Enteritidis	3888	2432
SAL_FA3429AA	129439	SRR3286944	2015	6	Europe	United Kingdom	Enteritidis	Enteritidis	26483	2430
SAL_FA3430AA	129404	SRR3286943	2015	6	Europe	United Kingdom	Enteritidis	Enteritidis	1425	2429
SAL_FA3431AA	129410	SRR3286942	2015	6	Europe	United Kingdom	Enteritidis	Enteritidis	1425	2428
SAL_FA3432AA	126846	SRR3286941	2015	6	Europe	United Kingdom	Enteritidis	Enteritidis	35040	2427

SAL_FA3434AA	128186	SRR3286939	2015	6		Europe	United Kingdom	Enteritidis	Enteritidis	3888	1632
SAL_FA3435AA	127545	SRR3286938	2015	6		Europe	United Kingdom	Enteritidis	Enteritidis	1425	2425
SAL_FA3436AA	128203	SRR3286937	2015	6		Europe	United Kingdom	Enteritidis	Enteritidis	1425	2424
SAL_FA3438AA	128229	SRR3286935	2015	7		Europe	United Kingdom	Enteritidis	Enteritidis	1425	717
SAL_FA3439AA	126180	SRR3286934	2015	6		Europe	United Kingdom	Enteritidis	Enteritidis	26493	2374
SAL_FA3440AA	128198	SRR3286933	2015	6		Europe	United Kingdom	Enteritidis	Enteritidis	1425	2422
SAL_FA3442AA	126087	SRR3286930	2015	6		Europe	United Kingdom	Enteritidis	Enteritidis	1425	2421
SAL_FA3444AA	129506	SRR3286928	2015	7		Europe	United Kingdom	Enteritidis	Enteritidis	1425	2419
SAL_FA3448AA	128180	SRR3286924	2015	6		Europe	United Kingdom	Enteritidis	Enteritidis	1425	2416
SAL_FA3449AA	126066	SRR3286923	2015	6		Europe	United Kingdom	Enteritidis	Enteritidis	27796	2415
SAL_FA3452AA	128172	SRR3286919	2015	6		Europe	United Kingdom	Enteritidis	Enteritidis	3888	2412
SAL_FA3455AA	126090	SRR3286916	2015	6		Europe	United Kingdom	Enteritidis	Enteritidis	28574	2409
SAL_FA3460AA	120627	SRR3286909	2015	6		Europe	United Kingdom	Enteritidis	Enteritidis	1425	2406
SAL_FA3461AA	126177	SRR3286908	2015	6		Europe	United Kingdom	Enteritidis	Enteritidis	1425	2254
SAL_FA3462AA	122202	SRR3286907	2015	6		Europe	United Kingdom	Enteritidis	Enteritidis	1425	2404
SAL_FA3463AA	120616	SRR3286906	2015	6		Europe	United Kingdom	Enteritidis	Enteritidis	1425	2405
SAL_FA3467AA	120625	SRR3286902	2015	6		Europe	United Kingdom	Enteritidis	Enteritidis	3888	2402
SAL_FA3469AA	109261	SRR3286900	2015	5		Europe	United Kingdom	Enteritidis	Enteritidis	1425	2400
SAL_FA3479AA	119502	SRR3286886	2015	6	2	Europe	United Kingdom	Enteritidis	Enteritidis	1425	2392
SAL_FA3482AA	123914	SRR3286880	2015	6		Europe	United Kingdom	Enteritidis	Enteritidis	1425	2389
SAL_FA3483AA	119500	SRR3286879	2015	6		Europe	United Kingdom	Enteritidis	Enteritidis	1425	2388
SAL_FA3484AA	122192	SRR3286877	2015	6		Europe	United Kingdom	Enteritidis	Enteritidis	1425	2387
SAL_FA3487AA	120575	SRR3286870	2015	4	30	Europe	United Kingdom	Enteritidis	Enteritidis	35347	2385
SAL_FA3491AA	122699	SRR3286863	2015	6	15	Europe	United Kingdom	Enteritidis	Enteritidis	1425	2381
SAL_FA3493AA	123883	SRR3286861	2015	6		Europe	United Kingdom	Enteritidis	Enteritidis	1425	2379
SAL_FA3494AA	117250	SRR3286860	2015	5		Europe	United Kingdom	Enteritidis	Enteritidis	1425	2378
SAL_FA3495AA	123913	SRR3286859	2015	6		Europe	United Kingdom	Enteritidis	Enteritidis	26493	2377
SAL_FA3498AA	123863	SRR3286856	2015	6		Europe	United Kingdom	Enteritidis	Enteritidis	3888	2375
SAL_FA3499AA	123899	SRR3286855	2015	6		Europe	United Kingdom	Enteritidis	Enteritidis	26493	2374
SAL_FA3501AA	118882	SRR3286853	2015	5		Europe	United Kingdom	Enteritidis	Enteritidis	1425	2163
SAL_FA3502AA	122649	SRR3286852	2015	6		Europe	United Kingdom	Enteritidis	Enteritidis	1425	2372
SAL_FA3504AA	123862	SRR3286850	2015	6		Europe	United Kingdom	Enteritidis	Enteritidis	1425	2371
SAL_FA3505AA	121453	SRR3286849	2015	6		Europe	United Kingdom	Enteritidis	Enteritidis	3888	2370
SAL_FA3509AA	122682	SRR3286836	2015	6	12	Europe	United Kingdom	Enteritidis	Enteritidis	1425	2367
SAL_FA3516AA	121448	SRR3286812	2015	6		Europe	United Kingdom	Enteritidis	Enteritidis	1425	2361
SAL_FA3517AA	109254	SRR3286811	2015	5		Europe	United Kingdom	Enteritidis	Enteritidis	3888	2351
SAL_FA3518AA	115280	SRR3286809	2015	5		Europe	United Kingdom	Enteritidis	Enteritidis	1425	2360
SAL_FA3520AA	114071	SRR3286778	2015	5		Europe	United Kingdom	Enteritidis	Enteritidis	1425	2358
SAL_FA3522AA	117283	SRR3286730	2015	5		Europe	United Kingdom	Enteritidis	Enteritidis	1425	2357
SAL_FA3527AA	117301	SRR3286718	2015	5		Europe	United Kingdom	Enteritidis	Enteritidis	1425	2352
SAL_FA3528AA	114085	SRR3286717	2015	5		Europe	United Kingdom	Enteritidis	Enteritidis	3888	2351
SAL_FA3534AA	113499	SRR3286711	2015	5		Europe	United Kingdom	Enteritidis	Enteritidis	1425	2345
SAL_FA3535AA	116566	SRR3286710	2015	5		Europe	United Kingdom	Enteritidis	Enteritidis	1425	2344
SAL_FA3537AA	115274	SRR3286708	2015	5		Europe	United Kingdom	Enteritidis	Enteritidis	1425	2343
SAL_FA3543AA	113497	SRR3286702	2015	5		Europe	United Kingdom	Enteritidis	Enteritidis	1425	2337
SAL_FA3544AA	115281	SRR3286701	2015	5		Europe	United Kingdom	Enteritidis	Enteritidis	1425	2336
SAL_FA3545AA	114067	SRR3286700	2015	5		Europe	United Kingdom	Enteritidis	Enteritidis	1425	2299
SAL_FA3547AA	115273	SRR3286698	2015	5		Europe	United Kingdom	Enteritidis	Enteritidis	1425	2334
SAL_FA3548AA	114068	SRR3286697	2015	5		Europe	United Kingdom	Enteritidis	Enteritidis	1425	2299
SAL_FA3551AA	113001	SRR3286694	2015	5		Europe	United Kingdom	Enteritidis	Enteritidis	1425	2331
SAL_FA3552AA	106528	SRR3286693	2015	4		Europe	United Kingdom	Enteritidis	Enteritidis	3906	2330
SAL_FA3553AA	112986	SRR3286692	2015	5		Europe	United Kingdom	Enteritidis	Enteritidis	1425	2329
SAL_FA3554AA	109876	SRR3286691	2015	5		Europe	United Kingdom	Enteritidis	Enteritidis	1425	2328
SAL_FA3555AA	103758	SRR3286690	2015	4		Europe	United Kingdom	Enteritidis	Enteritidis	1425	2163
SAL_FA3558AA	115283	SRR3286687	2015	5		Europe	United Kingdom	Enteritidis	Enteritidis	1425	2325
SAL_FA3560AA	114038	SRR3286685	2015	5		Europe	United Kingdom	Enteritidis	Enteritidis	1425	2323
SAL_FA3561AA	106562	SRR3286684	2015	4		Europe	United Kingdom	Enteritidis	Enteritidis	7811	2322
SAL_FA3564AA	109873	SRR3286681	2015	5		Europe	United Kingdom	Enteritidis	Enteritidis	1425	2319
SAL_FA3565AA	105998	SRR3286680	2015	4		Europe	United Kingdom	Enteritidis	Enteritidis	1425	2318

SAL_FA3568AA	112980	SRR3286677	2015	5		Europe	United Kingdom	Enteritidis	Enteritidis	1425	2315
SAL_FA3570AA	103763	SRR3286675	2015	4		Europe	United Kingdom	Enteritidis	Enteritidis	1425	2313
SAL_FA3571AA	113078	SRR3286674	2015	5		Europe	United Kingdom	Enteritidis	Enteritidis	1425	2312
SAL_FA3575AA	106032	SRR3286670	2015	4		Europe	United Kingdom	Enteritidis	Enteritidis	1425	503
SAL_FA3576AA	109919	SRR3286669	2015	5		Europe	United Kingdom	Enteritidis	Enteritidis	1425	2309
SAL_FA3577AA	110978	SRR3286668	2015	5		Europe	United Kingdom	Enteritidis	Enteritidis	3900	2308
SAL_FA3579AA	101448	SRR3286666	2015	4		Europe	United Kingdom	Enteritidis	Enteritidis	1425	2306
SAL_FA3580AA	106010	SRR3286665	2015	4		Europe	United Kingdom	Enteritidis	Enteritidis	1425	2157
SAL_FA3583AA	101460	SRR3286661	2015	4		Europe	United Kingdom	Enteritidis	Enteritidis	1425	2303
SAL_FA3585AA	103655	SRR3286659	2015	4		Europe	United Kingdom	Enteritidis	Enteritidis	3888	2301
SAL_FA3587AA	103731	SRR3286657	2015	4		Europe	United Kingdom	Enteritidis	Enteritidis	1425	2163
SAL_FA3588AA	103618	SRR3286656	2015	4		Europe	United Kingdom	Enteritidis	Enteritidis	1425	2300
SAL_FA3589AA	103724	SRR3286655	2015	4		Europe	United Kingdom	Enteritidis	Enteritidis	1425	2299
SAL_FA3590AA	113040	SRR3286649	2015	5		Europe	United Kingdom	Enteritidis	Enteritidis	1425	2298
SAL_FA3592AA	109918	SRR3286647	2015	5		Europe	United Kingdom	Enteritidis	Enteritidis	1425	2296
SAL_FA3594AA	103642	SRR3286645	2015	4		Europe	United Kingdom	Enteritidis	Enteritidis	1425	2294
SAL_FA3596AA	103607	SRR3286643	2015	4		Europe	United Kingdom	Enteritidis	Enteritidis	1425	2292
SAL_FA3599AA	101470	SRR3286639	2015	4		Europe	United Kingdom	Enteritidis	Enteritidis	1425	2291
SAL_FA3600AA	113022	SRR3286638	2015	5		Europe	United Kingdom	Enteritidis	Enteritidis	1425	2290
SAL_FA3602AA	106576	SRR3286636	2015	4		Europe	United Kingdom	Enteritidis	Enteritidis	1425	1274
SAL_FA3603AA	101434	SRR3286635	2015	4		Europe	United Kingdom	Enteritidis	Enteritidis	1425	2288
SAL_FA3606AA	107233	SRR3286632	2015	4		Europe	United Kingdom	Enteritidis	Enteritidis	1425	2254
SAL_FA3608AA	101458	SRR3286630	2015	4		Europe	United Kingdom	Enteritidis	Enteritidis	1425	2285
SAL_FA3609AA	113033	SRR3286629	2015	5		Europe	United Kingdom	Enteritidis	Enteritidis	1425	2284
SAL_FA3610AA	101462	SRR3286628	2015	4		Europe	United Kingdom	Enteritidis	Enteritidis	1425	2283
SAL_FA3612AA	106517	SRR3286626	2015	4		Europe	United Kingdom	Enteritidis	Enteritidis	1425	2281
SAL_FA3613AA	104256	SRR3286624	2015	4		Europe	United Kingdom	Enteritidis	Enteritidis	1425	2280
SAL_FA3615AA	113032	SRR3286622	2015	5		Europe	United Kingdom	Enteritidis	Enteritidis	1425	2278
SAL_FA3616AA	106510	SRR3286621	2015	4		Europe	United Kingdom	Enteritidis	Enteritidis	1425	2277
SAL_FA3618AA	107747	SRR3286619	2015	4		Europe	United Kingdom	Enteritidis	Enteritidis	1425	2274
SAL_FA3621AA	106521	SRR3286615	2015	4		Europe	United Kingdom	Enteritidis	Enteritidis	1425	2272
SAL_FA3622AA	106523	SRR3286614	2015	4		Europe	United Kingdom	Enteritidis	Enteritidis	1425	2271
SAL_FA3623AA	101443	SRR3286613	2015	4		Europe	United Kingdom	Enteritidis	Enteritidis	1425	2270
SAL_FA3625AA	106564	SRR3286611	2015	4		Europe	United Kingdom	Enteritidis	Enteritidis	1425	2269
SAL_FA3626AA	101465	SRR3286610	2015	4		Europe	United Kingdom	Enteritidis	Enteritidis	1425	2268
SAL_FA3627AA	107119	SRR3286609	2015	4		Europe	United Kingdom	Enteritidis	Enteritidis	1425	2254
SAL_FA3630AA	101313	SRR3286606	2015	4		Europe	United Kingdom	Enteritidis	Enteritidis	1425	2266
SAL_FA3631AA	103747	SRR3286605	2015	4		Europe	United Kingdom	Enteritidis	Enteritidis	1425	2265
SAL_FA3633AA	106553	SRR3286603	2015	4		Europe	United Kingdom	Enteritidis	Enteritidis	1425	2263
SAL_FA3634AA	104253	SRR3286602	2015	4	20	Europe	United Kingdom	Enteritidis	Enteritidis	1425	2262
SAL_FA3635AA	107750	SRR3286601	2015	4		Europe	United Kingdom	Enteritidis	Enteritidis	1425	2261
SAL_FA3640AA	101331	SRR3286596	2015	4		Europe	United Kingdom	Enteritidis	Enteritidis	1425	2257
SAL_FA3643AA	107296	SRR3286593	2015	4		Europe	United Kingdom	Enteritidis	Enteritidis	1425	2254
SAL_FA3646AA	101468	SRR3286590	2015	4		Europe	United Kingdom	Enteritidis	Enteritidis	1425	2251
SAL_FA3650AA	100742	SRR3286586	2015	4		Europe	United Kingdom	Enteritidis	Enteritidis	1425	2247
SAL_FA3651AA	100495	SRR3286585	2015	4		Europe	United Kingdom	Enteritidis	Enteritidis	1425	2246
SAL_FA3652AA	105328	SRR3286584	2015	4		Europe	United Kingdom	Enteritidis	Enteritidis	1425	2245
SAL_FA3653AA	103641	SRR3286583	2015	4		Europe	United Kingdom	Enteritidis	Enteritidis	1425	2244
SAL_FA3655AA	103767	SRR3286581	2015	4		Europe	United Kingdom	Enteritidis	Enteritidis	1425	2242
SAL_FA3660AA	103645	SRR3286576	2015	4		Europe	United Kingdom	Enteritidis	Enteritidis	1425	2237
SAL_FA3661AA	100738	SRR3286575	2015	4		Europe	United Kingdom	Enteritidis	Enteritidis	28005	2236
SAL_FA3662AA	100733	SRR3286574	2015	4		Europe	United Kingdom	Enteritidis	Enteritidis	1425	2235
SAL_FA3663AA	106518	SRR3286573	2015	4		Europe	United Kingdom	Enteritidis	Enteritidis	1425	2234
SAL_FA3664AA	103629	SRR3286572	2015	4		Europe	United Kingdom	Enteritidis	Enteritidis	3897	2233
SAL_FA3665AA	107131	SRR3286571	2015	4		Europe	United Kingdom	Enteritidis	Enteritidis	1425	2232
SAL_FA3667AA	100736	SRR3286569	2015	4		Europe	United Kingdom	Enteritidis	Enteritidis	1425	2230
SAL_FA3668AA	107096	SRR3286568	2015	4		Europe	United Kingdom	Enteritidis	Enteritidis	1425	2229
SAL_FA3672AA	129359	SRR3285500	2015	6		Europe	United Kingdom	Enteritidis	Enteritidis	1425	1808
SAL_FA3674AA	178855	SRR3285498	2015	10		Europe	United Kingdom	Enteritidis	Enteritidis	1425	2224

SAL_FA3675AA	166184	SRR3285497	2015	9		Europe	United Kingdom	Enteritidis	Enteritidis	1425	2223
SAL_FA3676AA	48168	SRR3285496	2014	9		Europe	United Kingdom	Enteritidis	Enteritidis	1425	2222
SAL_FA3677AA	178691	SRR3285495	2015	10		Europe	United Kingdom	Enteritidis	Enteritidis	1425	2221
SAL_FA3678AA	137140	SRR3285494	2015	7		Europe	United Kingdom	Enteritidis	Enteritidis	1425	2220
SAL_FA3679AA	169597	SRR3285493	2015	9		Europe	United Kingdom	Enteritidis	Enteritidis	1425	2219
SAL_FA3680AA	143958	SRR3285492	2015	8		Europe	United Kingdom	Enteritidis	Enteritidis	3888	2218
SAL_FA3684AA	192871	SRR3285488	2015	11		Europe	United Kingdom	Enteritidis	Enteritidis	1425	2216
SAL_FA3686AA	170325	SRR3285486	2015	9		Europe	United Kingdom	Enteritidis	Enteritidis	1425	2214
SAL_FA3687AA	109245	SRR3285485	2015	5		Europe	United Kingdom	Enteritidis	Enteritidis	1425	1274
SAL_FA3688AA	189248	SRR3285484	2015	11		Europe	United Kingdom	Enteritidis	Enteritidis	1425	2213
SAL_FA3691AA	126065	SRR3285481	2015	6		Europe	United Kingdom	Enteritidis	Enteritidis	1425	2210
SAL_FA3692AA	171186	SRR3285480	2015	10		Europe	United Kingdom	Enteritidis	Enteritidis	1425	2163
SAL_FA3693AA	146123	SRR3285479	2015	8		Europe	United Kingdom	Enteritidis	Enteritidis	3888	2209
SAL_FA3697AA	176346	SRR3285475	2015	10		Europe	United Kingdom	Enteritidis	Enteritidis	1425	1408
SAL_FA3698AA	142688	SRR3285474	2015	8		Europe	United Kingdom	Enteritidis	Enteritidis	1425	2205
SAL_FA3700AA	164973	SRR3285472	2015	9		Europe	United Kingdom	Enteritidis	Enteritidis	1425	1990
SAL_FA3702AA	170334	SRR3285470	2015	9	17	Europe	United Kingdom	Enteritidis	Enteritidis	1425	2203
SAL_FA3708AA	121459	SRR3285464	2015	6		Europe	United Kingdom	Enteritidis	Enteritidis	1425	2198
SAL_FA3709AA	170408	SRR3285463	2015	9		Europe	United Kingdom	Enteritidis	Enteritidis	3888	2197
SAL_FA3710AA	180813	SRR3285462	2015	9	10	Europe	United Kingdom	Enteritidis	Enteritidis	1425	2196
SAL_FA3713AA	157325	SRR3285459	2015	9		Europe	United Kingdom	Enteritidis	Enteritidis	1425	2194
SAL_FA3715AA	135156	SRR3285457	2015	7		Europe	United Kingdom	Enteritidis	Enteritidis	3888	2193
SAL_FA3719AA	182010	SRR3285453	2015	10		Europe	United Kingdom	Enteritidis	Enteritidis	1425	2189
SAL_FA3723AA	142218	SRR3285449	2015	8		Europe	United Kingdom	Enteritidis	Enteritidis	1425	2185
SAL_FA3724AA	172293	SRR3285448	2015	10		Europe	United Kingdom	Enteritidis	Enteritidis	3888	2184
SAL_FA3725AA	171322	SRR3285447	2015	10		Europe	United Kingdom	Enteritidis	Enteritidis	1425	2183
SAL_FA3728AA	180569	SRR3285444	2015	10		Europe	United Kingdom	Enteritidis	Enteritidis	3888	2180
SAL_FA3729AA	174748	SRR3285443	2015	10		Europe	United Kingdom	Enteritidis	Enteritidis	3888	2179
SAL_FA3731AA	198247	SRR3285441	2015	12		Europe	United Kingdom	Enteritidis	Enteritidis	1425	2177
SAL_FA3736AA	121429	SRR3285436	2015	6		Europe	United Kingdom	Enteritidis	Enteritidis	1425	2172
SAL_FA3739AA	122279	SRR3285433	2015	6		Europe	United Kingdom	Enteritidis	Enteritidis	1425	2169
SAL_FA3741AA	161875	SRR3285431	2015	9		Europe	United Kingdom	Enteritidis	Enteritidis	1425	2167
SAL_FA3747AA	103640	SRR3285424	2015	4		Europe	United Kingdom	Enteritidis	Enteritidis	1425	2163
SAL_FA3748AA	106547	SRR3285423	2015	4		Europe	United Kingdom	Enteritidis	Enteritidis	1425	2162
SAL_FA3754AA	104249	SRR3285417	2015	4		Europe	United Kingdom	Enteritidis	Enteritidis	1425	2157
SAL_FA3755AA	146116	SRR3285416	2015	8	12	Europe	United Kingdom	Enteritidis	Enteritidis	1425	2156
SAL_FA3756AA	200657	SRR3285415	2015	12		Europe	United Kingdom	Enteritidis	Enteritidis	1425	2155
SAL_FA3760AA	181056	SRR3285411	2015	10		Europe	United Kingdom	Enteritidis	Enteritidis	1425	2152
SAL_FA3761AA	135063	SRR3285410	2015	7		Europe	United Kingdom	Enteritidis	Enteritidis	3888	2151
SAL_FA3762AA	193285	SRR3285409	2015	12		Europe	United Kingdom	Enteritidis	Enteritidis	1425	2097
SAL_FA3764AA	152452	SRR3285407	2015	8		Europe	United Kingdom	Enteritidis	Enteritidis	26493	2149
SAL_FA3767AA	107125	SRR3285404	2015	4		Europe	United Kingdom	Enteritidis	Enteritidis	26493	2147
SAL_FA3768AA	142494	SRR3285403	2015	7		Europe	United Kingdom	Enteritidis	Enteritidis	1425	2146
SAL_FA3776AA	133213	SRR3285395	2015	7		Europe	United Kingdom	Enteritidis	Enteritidis	1425	2139
SAL_FA3777AA	166228	SRR3285394	2015	9		Europe	United Kingdom	Enteritidis	Enteritidis	26493	2018
SAL_FA3778AA	170466	SRR3285393	2015	10		Europe	United Kingdom	Enteritidis	Enteritidis	1425	2138
SAL_FA3780AA	187543	SRR3285391	2015	11		Europe	United Kingdom	Enteritidis	Enteritidis	3888	2007
SAL_FA3781AA	158997	SRR3285390	2015	9		Europe	United Kingdom	Enteritidis	Enteritidis	3888	2136
SAL_FA3782AA	130369	SRR3285389	2015	6		Europe	United Kingdom	Enteritidis	Enteritidis	26493	2135
SAL_FA3783AA	179841	SRR3285386	2015	10		Europe	United Kingdom	Enteritidis	Enteritidis	34197	2134
SAL_FA3784AA	185449	SRR3285385	2015	11		Europe	United Kingdom	Enteritidis	Enteritidis	1425	1990
SAL_FA3787AA	179798	SRR3285382	2015	10		Europe	United Kingdom	Enteritidis	Enteritidis	1425	1990
SAL_FA3788AA	140241	SRR3285381	2015	7		Europe	United Kingdom	Enteritidis	Enteritidis	1425	2131
SAL_FA3792AA	152434	SRR3285377	2015	8		Europe	United Kingdom	Enteritidis	Enteritidis	3888	2127
SAL_FA3796AA	118884	SRR3285373	2015	5		Europe	United Kingdom	Enteritidis	Enteritidis	1425	2123
SAL_FA3797AA	118870	SRR3285372	2015	5		Europe	United Kingdom	Enteritidis	Enteritidis	1425	2122
SAL_FA3798AA	149324	SRR3285371	2015	8		Europe	United Kingdom	Enteritidis	Enteritidis	1425	2121
SAL_FA3799AA	123846	SRR3285370	2015	6		Europe	United Kingdom	Enteritidis	Enteritidis	26493	2120
SAL_FA3800AA	163160	SRR3285369	2015	9		Europe	United Kingdom	Enteritidis	Enteritidis	26483	2119

SAL_FA3804AA	175615	SRR3285365	2015	10		Europe	United Kingdom	Enteritidis	Enteritidis	3888	2116
SAL_FA3806AA	142224	SRR3285363	2015	8		Europe	United Kingdom	Enteritidis	Enteritidis	1425	2115
SAL_FA3807AA	143571	SRR3285362	2015	8		Europe	United Kingdom	Enteritidis	Enteritidis	1425	2069
SAL_FA3808AA	171340	SRR3285361	2015	9		Europe	United Kingdom	Enteritidis	Enteritidis	1425	2114
SAL_FA3813AA	178704	SRR3285355	2015	10		Europe	United Kingdom	Enteritidis	Enteritidis	1425	2109
SAL_FA3815AA	159662	SRR3285353	2015	9		Europe	United Kingdom	Enteritidis	Enteritidis	3888	2002
SAL_FA3816AA	135064	SRR3285352	2015	7		Europe	United Kingdom	Enteritidis	Enteritidis	1425	2107
SAL_FA3818AA	169663	SRR3285350	2015	9		Europe	United Kingdom	Enteritidis	Enteritidis	8185	2105
SAL_FA3823AA	186568	SRR3285345	2015	11		Europe	United Kingdom	Enteritidis	Enteritidis	1425	2100
SAL_FA3824AA	174648	SRR3285344	2015	9		Europe	United Kingdom	Enteritidis	Enteritidis	1425	2099
SAL_FA3825AA	189183	SRR3285343	2015	11		Europe	United Kingdom	Enteritidis	Enteritidis	3888	2098
SAL_FA3826AA	120597	SRR3285342	2015	6		Europe	United Kingdom	Enteritidis	Enteritidis	1425	2097
SAL_FA3828AA	112994	SRR3285339	2015	5		Europe	United Kingdom	Enteritidis	Enteritidis	1425	2095
SAL_FA3830AA	126091	SRR3285284	2015	6		Europe	United Kingdom	Enteritidis	Enteritidis	1425	2093
SAL_FA3831AA	133214	SRR3285283	2015	7	14	Europe	United Kingdom	Enteritidis	Enteritidis	1425	2092
SAL_FA3834AA	184423	SRR3285280	2015	11		Europe	United Kingdom	Enteritidis	Enteritidis	1425	1990
SAL_FA3836AA	136785	SRR3285278	2015	7		Europe	United Kingdom	Enteritidis	Enteritidis	1425	2088
SAL_FA3837AA	131518	SRR3285254	2015	7		Europe	United Kingdom	Enteritidis	Enteritidis	1425	2087
SAL_FA3839AA	106567	SRR3285245	2015	4		Europe	United Kingdom	Enteritidis	Enteritidis	1425	2085
SAL_FA3840AA	206728	SRR3285244	2016	1	12	Europe	United Kingdom	Enteritidis	Enteritidis	1425	2084
SAL_FA3844AA	183662	SRR3285109	2015	10	27	Europe	United Kingdom	Enteritidis	Enteritidis	1425	2080
SAL_FA3849AA	190586	SRR3285054	2015	11		Europe	United Kingdom	Enteritidis	Enteritidis	1425	2077
SAL_FA3852AA	159715	SRR3284869	2015	9		Europe	United Kingdom	Enteritidis	Enteritidis	1425	1948
SAL_FA3853AA	105339	SRR3284868	2015	4		Europe	United Kingdom	Enteritidis	Enteritidis	1425	2074
SAL_FA3855AA	103255	SRR3284861	2015	4		Europe	United Kingdom	Enteritidis	Enteritidis	1425	2072
SAL_FA3856AA	169668	SRR3284856	2015	9		Europe	United Kingdom	Enteritidis	Enteritidis	1425	2071
SAL_FA3858AA	143570	SRR3284852	2015	8		Europe	United Kingdom	Enteritidis	Enteritidis	1425	2069
SAL_FA3860AA	182159	SRR3284850	2015	10		Europe	United Kingdom	Enteritidis	Enteritidis	1425	2067
SAL_FA3861AA	149212	SRR3284849	2015	8		Europe	United Kingdom	Enteritidis	Enteritidis	3888	2066
SAL_FA3862AA	176277	SRR3284848	2015	10		Europe	United Kingdom	Enteritidis	Enteritidis	1425	2065
SAL_FA3864AA	158933	SRR3284846	2015	9		Europe	United Kingdom	Enteritidis	Enteritidis	1425	2063
SAL_FA3866AA	181051	SRR3284844	2015	10		Europe	United Kingdom	Enteritidis	Enteritidis	1425	2062
SAL_FA3867AA	172472	SRR3284843	2015	10		Europe	United Kingdom	Enteritidis	Enteritidis	1425	1957
SAL_FA3870AA	166231	SRR3284840	2015	9		Europe	United Kingdom	Enteritidis	Enteritidis	1425	2059
SAL_FA3872AA	149231	SRR3284838	2015	8		Europe	United Kingdom	Enteritidis	Enteritidis	1425	2057
SAL_FA3873AA	107309	SRR3284837	2015	4		Europe	United Kingdom	Enteritidis	Enteritidis	26493	2056
SAL_FA3874AA	109938	SRR3284836	2015	5		Europe	United Kingdom	Enteritidis	Enteritidis	1425	2055
SAL_FA3875AA	144002	SRR3284835	2015	8		Europe	United Kingdom	Enteritidis	Enteritidis	1425	2054
SAL_FA3877AA	175539	SRR3284833	2015	9		Europe	United Kingdom	Enteritidis	Enteritidis	1425	613
SAL_FA3883AA	188175	SRR3284827	2015	11		Europe	United Kingdom	Enteritidis	Enteritidis	1425	2016
SAL_FA3884AA	122697	SRR3284826	2015	6		Europe	United Kingdom	Enteritidis	Enteritidis	26493	2047
SAL_FA3886AA	160809	SRR3284824	2015	9		Europe	United Kingdom	Enteritidis	Enteritidis	3888	2045
SAL_FA3888AA	169671	SRR3284822	2015	9		Europe	United Kingdom	Enteritidis	Enteritidis	1425	2043
SAL_FA3890AA	204008	SRR3284820	2016	1		Europe	United Kingdom	Enteritidis	Enteritidis	1425	2041
SAL_FA3893AA	121447	SRR3284817	2015	6		Europe	United Kingdom	Enteritidis	Enteritidis	1425	2039
SAL_FA3895AA	191967	SRR3284815	2015	11		Europe	United Kingdom	Enteritidis	Enteritidis	3888	2037
SAL_FA3902AA	176347	SRR3284808	2015	10		Europe	United Kingdom	Enteritidis	Enteritidis	1425	2030
SAL_FA3904AA	174783	SRR3284805	2015	10		Europe	United Kingdom	Enteritidis	Enteritidis	1425	2028
SAL_FA3907AA	161866	SRR3284782	2015	9		Europe	United Kingdom	Enteritidis	Enteritidis	3888	2023
SAL_FA3911AA	158994	SRR3284775	2015	9		Europe	United Kingdom	Enteritidis	Enteritidis	3888	2023
SAL_FA3912AA	190575	SRR3284774	2015	11		Europe	United Kingdom	Enteritidis	Enteritidis	1425	2022
SAL_FA3913AA	197074	SRR3284773	2015	12	4	Europe	United Kingdom	Enteritidis	Enteritidis	1425	2021
SAL_FA3914AA	100737	SRR3284772	2015	4		Europe	United Kingdom	Enteritidis	Enteritidis	3888	2020
SAL_FA3915AA	128174	SRR3284771	2015	6		Europe	United Kingdom	Enteritidis	Enteritidis	1425	2019
SAL_FA3916AA	191951	SRR3284770	2015	11		Europe	United Kingdom	Enteritidis	Enteritidis	1425	2016
SAL_FA3917AA	157319	SRR3284769	2015	8		Europe	United Kingdom	Enteritidis	Enteritidis	26493	2018
SAL_FA3919AA	189189	SRR3284766	2015	11		Europe	United Kingdom	Enteritidis	Enteritidis	1425	2016
SAL_FA3923AA	140604	SRR3284762	2015	7		Europe	United Kingdom	Enteritidis	Enteritidis	1425	1275
SAL_FA3925AA	131459	SRR3284759	2015	7		Europe	United Kingdom	Enteritidis	Enteritidis	26483	2011

SAL_FA3929AA	182868	SRR3284755	2015	11		Europe	United Kingdom	Enteritidis	Enteritidis	3888	2007
SAL_FA3934AA	154870	SRR3284750	2015	9		Europe	United Kingdom	Enteritidis	Enteritidis	3888	2002
SAL_FA3938AA	109931	SRR3284746	2015	5		Europe	United Kingdom	Enteritidis	Enteritidis	1425	1274
SAL_FA3939AA	178676	SRR3284745	2015	10		Europe	United Kingdom	Enteritidis	Enteritidis	1425	1998
SAL_FA3942AA	190564	SRR3284742	2015	11		Europe	United Kingdom	Enteritidis	Enteritidis	1425	1995
SAL_FA3944AA	107094	SRR3284740	2015	4		Europe	United Kingdom	Enteritidis	Enteritidis	1425	1993
SAL_FA3946AA	142529	SRR3284738	2015	8		Europe	United Kingdom	Enteritidis	Enteritidis	26493	1991
SAL_FA3947AA	163142	SRR3284737	2015	9		Europe	United Kingdom	Enteritidis	Enteritidis	1425	1990
SAL_FA3948AA	134013	SRR3284736	2015	7	15	Europe	United Kingdom	Enteritidis	Enteritidis	35071	1989
SAL_FA3949AA	131461	SRR3284735	2015	7		Europe	United Kingdom	Enteritidis	Enteritidis	1425	1988
SAL_FA3952AA	170467	SRR3284732	2015	10		Europe	United Kingdom	Enteritidis	Enteritidis	1425	1268
SAL_FA3955AA	109267	SRR3284729	2015	5		Europe	United Kingdom	Enteritidis	Enteritidis	1425	1983
SAL_FA3960AA	164956	SRR3284724	2015	9		Europe	United Kingdom	Enteritidis	Enteritidis	1425	1979
SAL_FA3963AA	166245	SRR3284721	2015	9	23	Europe	United Kingdom	Enteritidis	Enteritidis	35377	1976
SAL_FA3964AA	164874	SRR3284720	2015	9		Europe	United Kingdom	Enteritidis	Enteritidis	1425	1975
SAL_FA3966AA	182816	SRR3284718	2015	11		Europe	United Kingdom	Enteritidis	Enteritidis	1425	1973
SAL_FA3971AA	128230	SRR3284713	2015	7		Europe	United Kingdom	Enteritidis	Enteritidis	1425	1969
SAL_FA3972AA	164816	SRR3284712	2015	9		Europe	United Kingdom	Enteritidis	Enteritidis	1425	1968
SAL_FA3974AA	158998	SRR3284710	2015	9		Europe	United Kingdom	Enteritidis	Enteritidis	1425	1966
SAL_FA3976AA	180566	SRR3284708	2015	10		Europe	United Kingdom	Enteritidis	Enteritidis	1425	1964
SAL_FA3977AA	196423	SRR3284707	2015	12		Europe	United Kingdom	Enteritidis	Enteritidis	1425	1963
SAL_FA3978AA	179657	SRR3284694	2015	10		Europe	United Kingdom	Enteritidis	Enteritidis	26493	1962
SAL_FA3980AA	132044	SRR3284692	2015	7		Europe	United Kingdom	Enteritidis	Enteritidis	1425	1960
SAL_FA3982AA	106516	SRR3284690	2015	4		Europe	United Kingdom	Enteritidis	Enteritidis	1425	1958
SAL_FA3983AA	185901	SRR3284689	2015	11	3	Europe	United Kingdom	Enteritidis	Enteritidis	1425	1957
SAL_FA3984AA	143653	SRR3284688	2015	8		Europe	United Kingdom	Enteritidis	Enteritidis	1425	1956
SAL_FA3985AA	199911	SRR3284687	2015	12		Europe	United Kingdom	Enteritidis	Enteritidis	1425	1955
SAL_FA3987AA	174784	SRR3284685	2015	10		Europe	United Kingdom	Enteritidis	Enteritidis	3888	1953
SAL_FA3988AA	184480	SRR3284684	2015	11		Europe	United Kingdom	Enteritidis	Enteritidis	1425	1952
SAL_FA3989AA	146181	SRR3284683	2015	8		Europe	United Kingdom	Enteritidis	Enteritidis	1425	1951
SAL_FA3992AA	107130	SRR3284680	2015	4		Europe	United Kingdom	Enteritidis	Enteritidis	1425	1948
SAL_FA3993AA	152404	SRR3284679	2015	8		Europe	United Kingdom	Enteritidis	Enteritidis	1425	1947
SAL_FA4003AA	E2016003857	SRR3316428	2016	3		North America	United States	Enteritidis	Enteritidis	1425	1938
SAL_FA4004AA	E2016003759	SRR3316427	2016	3		North America	United States	Enteritidis	Enteritidis	1425	1938
SAL_FA4063AA	207512	SRR3323060	2016	1		Europe	United Kingdom	Enteritidis	Enteritidis	1425	1887
SAL_FA4108AA	21807	SRR3323014	2014	6	8	Europe	United Kingdom	Enteritidis	Enteritidis	3888	674
SAL_FA4116AA	176329	SRR3323006	2015	10		Europe	United Kingdom	Enteritidis	Enteritidis	1425	1842
SAL_FA4152AA	126064	SRR3322938	2015	6		Europe	United Kingdom	Enteritidis	Enteritidis	1425	1808
SAL_FA4170AA	132048	SRR3322733	2015	7		Europe	United Kingdom	Enteritidis	Enteritidis	3888	1791
SAL_FA4249AA	170402	SRR3322652	2015	9		Europe	United Kingdom	Enteritidis	Enteritidis	3888	1717
SAL_FA4280AA	197002	SRR3322619	2015	12		Europe	United Kingdom	Enteritidis	Enteritidis	1425	295
SAL_FA4296AA	185896	SRR3322538	2015	11		Europe	United Kingdom	Enteritidis	Enteritidis	1425	1676
SAL_FA4301AA	128156	SRR3322421	2015	6		Europe	United Kingdom	Enteritidis	Enteritidis	27796	1671
SAL_FA4303AA	160820	SRR3322419	2015	9		Europe	United Kingdom	Enteritidis	Enteritidis	1425	1669
SAL_FA4327AA	137149	SRR3322393	2015	7		Europe	United Kingdom	Enteritidis	Enteritidis	1425	1648
SAL_FA4338AA	164957	SRR3322381	2015	9		Europe	United Kingdom	Enteritidis	Enteritidis	1425	1637
SAL_FA4344AA	127426	SRR3322375	2015	6		Europe	United Kingdom	Enteritidis	Enteritidis	3888	1632
SAL_FA4428AA	109887	SRR3322199	2015	5		Europe	United Kingdom	Enteritidis	Enteritidis	1425	1554
SAL_FA4437AA	189242	SRR3322162	2015	11		Europe	United Kingdom	Enteritidis	Enteritidis	1425	1546
SAL_FA4438AA	166319	SRR3322161	2015	9		Europe	United Kingdom	Enteritidis	Enteritidis	3888	1545
SAL_FA4489AA	182004	SRR3322107	2015	10		Europe	United Kingdom	Enteritidis	Enteritidis	1425	1498
SAL_FA4524AA	176343	SRR3322070	2015	10		Europe	United Kingdom	Enteritidis	Enteritidis	-1926	1466
SAL_FA4542AA	140585	SRR3322032	2015	7		Europe	United Kingdom	Enteritidis	Enteritidis	1425	1449
SAL_FA4558AA	134020	SRR3322014	2015	7		Europe	United Kingdom	Enteritidis	Enteritidis	1425	1434
SAL_FA4571AA	126088	SRR3321904	2015	6		Europe	United Kingdom	Enteritidis	Enteritidis	1425	1422
SAL_FA4585AA	176352	SRR3321890	2015	10		Europe	United Kingdom	Enteritidis	Enteritidis	1425	1408
SAL_FA4598AA	118881	SRR3321876	2015	5		Europe	United Kingdom	Enteritidis	Enteritidis	1425	1396
SAL_FA4638AA	133215	SRR3321836	2015	7		Europe	United Kingdom	Enteritidis	Enteritidis	1425	1358
SAL_FA4648AA	140221	SRR3321814	2015	7		Europe	United Kingdom	Enteritidis	Enteritidis	1425	1348

SAL_FA4728AA	154871	SRR3319072	2015	9	Europe	United Kingdom	Enteritidis	Enteritidis	1425	1280
SAL_FA4729AA	154875	SRR3319071	2015	9	Europe	United Kingdom	Enteritidis	Enteritidis	1425	1279
SAL_FA4731AA	113500	SRR3319069	2015	5	Europe	United Kingdom	Enteritidis	Enteritidis	1425	1277
SAL_FA4732AA	149234	SRR3319068	2015	8	Europe	United Kingdom	Enteritidis	Enteritidis	1425	1276
SAL_FA4733AA	137144	SRR3319067	2015	7	Europe	United Kingdom	Enteritidis	Enteritidis	1425	1275
SAL_FA4734AA	109929	SRR3319066	2015	5	Europe	United Kingdom	Enteritidis	Enteritidis	1425	1274
SAL_FA4735AA	174697	SRR3319065	2015	10	Europe	United Kingdom	Enteritidis	Enteritidis	1425	1265
SAL_FA4738AA	155656	SRR3319062	2015	9	Europe	United Kingdom	Enteritidis	Enteritidis	1425	1271
SAL_FA4739AA	155653	SRR3319061	2015	9	Europe	United Kingdom	Enteritidis	Enteritidis	1425	1270
SAL_FA4740AA	155650	SRR3319060	2015	9	Europe	United Kingdom	Enteritidis	Enteritidis	1425	1269
SAL_FA4741AA	155659	SRR3319059	2015	9	Europe	United Kingdom	Enteritidis	Enteritidis	1425	1268
SAL_FA4742AA	164954	SRR3319058	2015	9	Europe	United Kingdom	Enteritidis	Enteritidis	1425	1267
SAL_FA4743AA	135161	SRR3319057	2015	7	Europe	United Kingdom	Enteritidis	Enteritidis	1425	1266
SAL_FA4744AA	155705	SRR3319056	2015	8	Europe	United Kingdom	Enteritidis	Enteritidis	1425	1265
SAL_FA4745AA	155649	SRR3319055	2015	9	Europe	United Kingdom	Enteritidis	Enteritidis	1425	1264
SAL_FA4808AA	CVM-N27379	SRR3330244	2010		North America	United States	Enteritidis	Enteritidis	1425	1205
SAL_FA4812AA	I2016004507-1	SRR3330240	2016	3	North America	United States	Enteritidis	Enteritidis	1425	1201
SAL_FA4813AA	I2016004374-4	SRR3330239	2016	3	North America	United States	Enteritidis	Enteritidis	1425	1200
SAL_FA4814AA	I2016004355	SRR3372231	2016	3	North America	United States	Enteritidis	Enteritidis	1425	1199
SAL_FA4815AA	E2016004634	SRR3330237	2016	3	North America	United States	Enteritidis	Enteritidis	1425	1198
SAL_FA4816AA	E2016004542	SRR3330236	2016	3	North America	United States	Enteritidis	Enteritidis	1425	240
SAL_FA4817AA	E2016004291	SRR3330232	2016	3	North America	United States	Enteritidis	Enteritidis	1425	1197
SAL_FA4819AA	CVM-N19817	SRR3330228	2008		North America	United States	Enteritidis	Enteritidis	1425	1195
SAL_FA4840AA	CVM-N27385	SRR3330248	2010		North America	United States	Enteritidis	Enteritidis	1425	1174
SAL_FA4841AA	CVM-N27382	SRR3330247	2010		North America	United States	Enteritidis	Enteritidis	1425	1173
SAL_FA4842AA	CVM-N27381	SRR3330246	2010		North America	United States	Enteritidis	Enteritidis	1425	1172
SAL_FA4843AA	E2016004541	SRR3330235	2016	3	North America	United States	Enteritidis	Enteritidis	1425	1171
SAL_FA4844AA	E2016004518	SRR3330234	2016	3	North America	United States	Enteritidis	Enteritidis	1425	1170
SAL_FA4845AA	E2016004517	SRR3330233	2016	3	North America	United States	Enteritidis	Enteritidis	1425	1170
SAL_FA4847AA	CVM-N19819	SRR3330229	2008		North America	United States	Enteritidis	Enteritidis	1425	1168
SAL_FA4982AA	MS150181	traces-0CZhrSa	2015		Europe	Ireland	Enteritidis	Enteritidis	1425	1066
SAL_FA4992AA	CFSAN035132	SRR3394620	2012		South America	Chile	Enteritidis	Enteritidis	1425	1057
SAL_FA4993AA	PNUSAS001812	SRR3393569	2016	3	North America	United States	Enteritidis	Enteritidis	1425	1056
SAL_FA4994AA	CFSAN035130	SRR3393568	2012		South America	Chile	Enteritidis	Enteritidis	1425	1055
SAL_FA4995AA	CFSAN035129	SRR3393567	2012		South America	Chile	Enteritidis	Enteritidis	35328	1054
SAL_FA5003AA	PNUSAS001826	SRR3392876			North America	United States	Enteritidis (Predicted)	Enteritidis	1425	1026
SAL_FA5004AA	CFSAN035124	SRR3394941	2011		South America	Chile	Enteritidis	Enteritidis	1425	1047
SAL_FA5005AA	CFSAN035123	SRR3394940	2012		South America	Chile	Enteritidis	Enteritidis	27775	767
SAL_FA5006AA	CFSAN035122	SRR3394939	2011		South America	Chile	Enteritidis	Enteritidis	1425	1046
SAL_FA5007AA	CFSAN035121	SRR3394938	2010		South America	Chile	Enteritidis	Enteritidis	1425	1045
SAL_FA5008AA	CFSAN035120	SRR3394937	2010		South America	Chile	Enteritidis	Enteritidis	1425	1044
SAL_FA5009AA	CFSAN035119	SRR3394936	2010		South America	Chile	Enteritidis	Enteritidis	1425	1043
SAL_FA5010AA	CFSAN035118	SRR3394935	2010		South America	Chile	Enteritidis	Enteritidis	1425	1042
SAL_FA5011AA	CFSAN035117	SRR3394934	2010		South America	Chile	Enteritidis	Enteritidis	27775	1041
SAL_FA5012AA	CFSAN035116	SRR3394933	2008		South America	Chile	Enteritidis	Enteritidis	1425	1040
SAL_FA5013AA	CFSAN035115	SRR3394932	2008		South America	Chile	Enteritidis	Enteritidis	1425	1039
SAL_FA5014AA	CFSAN035114	SRR3394931	2008		South America	Chile	Enteritidis	Enteritidis	1425	1038
SAL_FA5015AA	CFSAN035113	SRR3392831	2008		South America	Chile	Enteritidis	Enteritidis	1425	1037
SAL_FA5016AA	CFSAN035112	SRR3392830	2009		South America	Chile	Enteritidis	Enteritidis	1425	1036
SAL_FA5017AA	CFSAN035111	SRR3392829	2009		South America	Chile	Enteritidis	Enteritidis	1425	1035
SAL_FA5018AA	CFSAN035110	SRR3392828	2009		South America	Chile	Enteritidis	Enteritidis	1425	1035
SAL_FA5019AA	CFSAN035109	SRR3392826	2009		South America	Chile	Enteritidis	Enteritidis	1425	1034
SAL_FA5020AA	CFSAN035108	SRR3392825	2009		South America	Chile	Enteritidis	Enteritidis	1425	1033
SAL_FA5021AA	CFSAN035107	SRR3392824	2009		South America	Chile	Enteritidis	Enteritidis	1425	1032
SAL_FA5024AA	PNUSAS001770	SRR3392815	2016	3	North America	United States	Enteritidis	Enteritidis	1425	1029
SAL_FA5027AA	PNUSAS001827	SRR3392812	2015	11	North America	United States	Enteritidis	Enteritidis	1425	1026
SAL_FA5037AA	PNUSAS001769	SRR3392802			North America	United States	Enteritidis (Predicted)	Enteritidis	1425	1018
SAL_FA5038AA	PNUSAS001768	SRR3392801			North America	United States	Enteritidis (Predicted)	Enteritidis	1425	1017
SAL_FA5044AA	2016K-0210	SRR3392791			North America	United States	Enteritidis (Predicted)	Enteritidis	1425	1011

SAL_FA5113AA	CFSAN048656	SRR3391920	2016	4		North America	United States	Enteritidis (Predicted)	Enteritidis	1425	956
SAL_FA5114AA	CFSAN048655	SRR3391919	2016	4		North America	United States	Enteritidis (Predicted)	Enteritidis	1425	955
SAL_FA5147AA	FSIS1606217	SRR3383972	2016			North America	United States	Enteritidis	Enteritidis	1425	915
SAL_FA5153AA	FSIS1606216	SRR3383893	2016			North America	United States	Enteritidis	Enteritidis	1425	915
SAL_FA5154AA	FSIS1606214	SRR3383892	2016			North America	United States	Enteritidis	Enteritidis	1425	915
SAL_FA5160AA	FDA00003979	SRR3383906	2012	1		Asia	India	I 8:-:1,5	Enteritidis	7942	922
SAL_FA5162AA	I2016004602	SRR3465450	2016	3		North America	United States	Enteritidis	Enteritidis	1425	920
SAL_FA5163AA	I2016004601	SRR3457736	2016	3		North America	United States	Enteritidis	Enteritidis	1425	919
SAL_FA5164AA	I2016003741	SRR3383901	2016	3		North America	United States	Enteritidis	Enteritidis	1425	918
SAL_FA5167AA	FSIS1606215	SRR3383819	2016			North America	United States	Enteritidis	Enteritidis	1425	915
SAL_FA5177AA	FDA00003903	SRR3379326	2011	7		North America	United States	Enteritidis	Enteritidis	1425	906
SAL_FA5178AA	FDA00003906	SRR3379325	2012	5		North America	United States	Enteritidis	Enteritidis	1425	905
SAL_FA5179AA	FDA00003907	SRR3379324	2012	5		North America	United States	Enteritidis	Enteritidis	1425	905
SAL_FA5180AA	FDA00003908	SRR3379323	2012	5		North America	United States	Enteritidis	Enteritidis	1425	904
SAL_FA5181AA	FDA00003909	SRR3379322	2012	5		North America	United States	Enteritidis	Enteritidis	1425	903
SAL_FA5220AA	CFSAN047695	SRR3372347	2015	11		North America	United States	Enteritidis (Predicted)	Enteritidis	1425	867
SAL_FA5254AA	CFSAN048431	SRR3372313	2016	3		North America	United States	Enteritidis	Enteritidis	1425	841
SAL_FA5318AA	I2016004512-1	SRR3372232	2016	3		North America	United States	Enteritidis	Enteritidis	1425	789
SAL_FA5319AA	E2016005001	SRR3372230	2016	3		North America	United States	Enteritidis	Enteritidis	1425	788
SAL_FA5320AA	E2016004853	SRR3372229	2016	3		North America	United States	Enteritidis	Enteritidis	1425	787
SAL_FA5321AA	E2016004845	SRR3372228	2016	3		North America	United States	Enteritidis	Enteritidis	34197	786
SAL_FA5322AA	E2016004805	SRR3372227	2016	3		North America	United States	Enteritidis	Enteritidis	1425	785
SAL_FA5323AA	E2016004789	SRR3372226	2016	3		North America	United States	Enteritidis	Enteritidis	1425	784
SAL_FA5324AA	E2016004753	SRR3372225	2016	3		North America	United States	Enteritidis	Enteritidis	1425	783
SAL_FA5325AA	E2016004642	SRR3372224	2016	3		North America	United States	Enteritidis	Enteritidis	1425	617
SAL_FA5328AA	CFSAN035149	SRR3394980	2012			South America	Chile	Enteritidis	Enteritidis	1425	780
SAL_FA5329AA	CFSAN035148	SRR3394967	2012			South America	Chile	Enteritidis	Enteritidis	1425	780
SAL_FA5330AA	CFSAN035147	SRR3394966	2012			South America	Chile	Enteritidis	Enteritidis	1425	780
SAL_FA5338AA	CFSAN035157	SRR3394958	2013			South America	Chile	Enteritidis	Enteritidis	1425	772
SAL_FA5342AA	CFSAN035153	SRR3394954	2013			South America	Chile	Enteritidis	Enteritidis	1425	768
SAL_FA5343AA	CFSAN035152	SRR3394953	2013			South America	Chile	Enteritidis	Enteritidis	1425	768
SAL_FA5344AA	CFSAN035151	SRR3394952	2013			South America	Chile	Enteritidis	Enteritidis	27775	767
SAL_FA5345AA	CFSAN035150	SRR3394951	2012			South America	Chile	Enteritidis	Enteritidis	27775	766
SAL_FA5348AA	CFSAN035134	SRR3394948	2012			South America	Chile	Enteritidis	Enteritidis	1425	763
SAL_FA5349AA	CFSAN035133	SRR3394947	2012			South America	Chile	Enteritidis	Enteritidis	1425	762
SAL_FA5350AA	CFSAN035131	SRR3394946	2012			South America	Chile	Enteritidis	Enteritidis	1425	761
SAL_FA5351AA	CFSAN035128	SRR3394945	2012			South America	Chile	Enteritidis	Enteritidis	1425	760
SAL_FA5352AA	CFSAN035127	SRR3394944	2012			South America	Chile	Enteritidis	Enteritidis	1425	759
SAL_FA5353AA	CFSAN035126	SRR3394943	2011			South America	Chile	Enteritidis	Enteritidis	1425	758
SAL_FA5354AA	CFSAN035125	SRR3394942	2011			South America	Chile	Enteritidis	Enteritidis	1425	757
SAL_FA5367AA	028V9	traces-0ZYCBAz				Africa	Malawi	Enteritidis (Predicted)	Enteritidis	1425	747
SAL_FA5405AA	2016K-0215	SRR3405708				North America	United States	Enteritidis (Predicted)	Enteritidis	1425	718
SAL_FA5406AA	2016K-0216	SRR3405707	2016	2		North America	United States	Enteritidis (Predicted)	Enteritidis	1425	717
SAL_FA5432AA	FSIS1606187	SRR3402072	2016			North America	United States	Enteritidis	Enteritidis	1425	693
SAL_FA5451AA	21809	SRR3401403	2014	6	8	Europe	United Kingdom	Enteritidis	Enteritidis	3888	674
SAL_FA5473AA	I2016005133	SRR3400300	2016	4		North America	United States	Enteritidis	Enteritidis	1425	656
SAL_FA5474AA	I2016005110	SRR3400299	2016	4		North America	United States	Enteritidis	Enteritidis	1425	655
SAL_FA5475AA	I2016005047	SRR3400298	2016	4		North America	United States	Enteritidis	Enteritidis	1425	654
SAL_FA5476AA	I2016005045	SRR3400297	2016	4		North America	United States	Enteritidis	Enteritidis	1425	653
SAL_FA5477AA	I2016005030	SRR3400296	2016	4		North America	United States	Enteritidis	Enteritidis	1425	652
SAL_FA5478AA	E2016005293	SRR3400295	2016	4		North America	United States	Enteritidis	Enteritidis	1425	651
SAL_FA5479AA	E2016005274	SRR3400294	2016	4		North America	United States	Enteritidis	Enteritidis	1425	650
SAL_FA5480AA	E2016005190	SRR3400293	2016	4		North America	United States	Enteritidis	Enteritidis	1425	649
SAL_FA5481AA	E2016005076	SRR3400292	2016	4		North America	United States	Enteritidis	Enteritidis	1425	648
SAL_FA5482AA	E2016005028	SRR3400291	2016	3		North America	United States	Enteritidis	Enteritidis	1425	647
SAL_FA5498AA	E2016005025	SRR3400015	2016	4		North America	United States	Enteritidis	Enteritidis	1425	240
SAL_FA5508AA	CVM-N19847	SRR3399993	2008			North America	United States	Enteritidis	Enteritidis	1425	622
SAL_FA5511AA	PNUSAS001887	SRR3399512	2016	3		North America	United States	Enteritidis	Enteritidis	26775	619
SAL_FA5513AA	PNUSAS001903	SRR3405952	2016	3		North America	United States	Enteritidis	Enteritidis	1425	617

SAL_FA5514AA	PNUSAS001902	SRR3405951	2016	3		North America	United States	Enteritidis	Enteritidis	1425	616
SAL_FA5515AA	PNUSAS001901	SRR3405950	2016	3		North America	United States	Enteritidis	Enteritidis	1425	615
SAL_FA5516AA	PNUSAS001900	SRR3405949				North America	United States	Enteritidis (Predicted)	Enteritidis	1425	614
SAL_FA5517AA	PNUSAS001899	SRR3405948	2016	3		North America	United States	Enteritidis	Enteritidis	1425	613
SAL_FA5538AA	PNUSAS001883	SRR3405867	2016	3		North America	United States	Enteritidis	Enteritidis	1425	595
SAL_FA5539AA	PNUSAS001947	SRR3405866	2016	3		North America	United States	Enteritidis	Enteritidis	1425	594
SAL_FA5543AA	PNUSAS001951	SRR3405858				North America	United States	Enteritidis (Predicted)	Enteritidis	1425	180
SAL_FA5547AA	PNUSAS001882	SRR3405766	2016	3		North America	United States	Enteritidis	Enteritidis	1425	589
SAL_FA5548AA	PNUSAS001881	SRR3405765	2016	3		North America	United States	Enteritidis	Enteritidis	1425	588
SAL_FA5551AA	PNUSAS001878	SRR3405762	2016	3		North America	United States	Enteritidis	Enteritidis	1425	585
SAL_FA5552AA	PNUSAS001877	SRR3405761	2016	3		North America	United States	Enteritidis	Enteritidis	1425	584
SAL_FA5557AA	PNUSAS001875	SRR3405754	2016	2		North America	United States	Enteritidis	Enteritidis	1425	580
SAL_FA5589AA	6388	SRR3410216	2014			Europe	Germany	Enteritidis	Enteritidis	3888	552
SAL_FA5590AA	6175	SRR3410215	2014			Europe	Germany	Enteritidis	Enteritidis	3888	551
SAL_FA5591AA	6012	SRR3410214	2014			Europe	Germany	Enteritidis	Enteritidis	3888	551
SAL_FA5592AA	5946	SRR3410213	2014			Europe	Germany	Enteritidis	Enteritidis	3888	550
SAL_FA5593AA	5795	SRR3410212	2014			Europe	Germany	Enteritidis	Enteritidis	3888	549
SAL_FA5594AA	5227	SRR3410211	2014			Europe	Germany	Enteritidis	Enteritidis	3888	548
SAL_FA5595AA	5226	SRR3410210	2014			Europe	Germany	Enteritidis	Enteritidis	3888	547
SAL_FA5596AA	5225	SRR3410209	2014			Europe	Germany	Enteritidis	Enteritidis	3888	546
SAL_FA5597AA	5224	SRR3410208	2014			Europe	Germany	Enteritidis	Enteritidis	3888	545
SAL_FA5619AA	54059	SRR3417508	2014			Europe	Germany	Enteritidis	Enteritidis	3888	526
SAL_FA5620AA	54053	SRR3417507	2014			Europe	Germany	Enteritidis	Enteritidis	3888	525
SAL_FA5621AA	54047	SRR3417506	2014			Europe	Germany	Enteritidis	Enteritidis	3888	524
SAL_FA5622AA	54038	SRR3417505	2014			Europe	Germany	Enteritidis	Enteritidis	3888	523
SAL_FA5623AA	54014	SRR3417504	2014			Europe	Germany	Enteritidis	Enteritidis	3888	522
SAL_FA5624AA	54008	SRR3417503	2014			Europe	Germany	Enteritidis	Enteritidis	3888	521
SAL_FA5625AA	53996	SRR3417502	2014			Europe	Germany	Enteritidis	Enteritidis	3888	520
SAL_FA5626AA	53973	SRR3417501	2014			Europe	Germany	Enteritidis	Enteritidis	3888	519
SAL_FA5627AA	50104	SRR3417500	2014			Europe	Austria	Enteritidis	Enteritidis	3888	518
SAL_FA5628AA	49693	SRR3417499	2014			Europe	Austria	Enteritidis	Enteritidis	3888	517
SAL_FA5629AA	49692	SRR3417498	2014			Europe	Austria	Enteritidis	Enteritidis	3888	516
SAL_FA5630AA	49691	SRR3417497	2014			Europe	Austria	Enteritidis	Enteritidis	3888	515
SAL_FA5631AA	45605	SRR3417496	2014			Europe	Luxembourg	Enteritidis	Enteritidis	3888	514
SAL_FA5633AA	37046	SRR3417494	2014			Europe	Austria	Enteritidis	Enteritidis	3888	512
SAL_FA5634AA	37045	SRR3417493	2014			Europe	Austria	Enteritidis	Enteritidis	3888	511
SAL_FA5660AA	MS150184	traces-0ELniWa	2015			Europe	Ireland	Enteritidis	Enteritidis	1425	503
SAL_FA5661AA	MS150182	traces-0xeNbNg	2015			Europe	Ireland	Enteritidis	Enteritidis	1425	503
SAL_FA5662AA	MS150185	traces-0zuhqHu	2015			Europe	Ireland	Enteritidis	Enteritidis	1425	502
SAL_FA5664AA	MS150094	traces-0hdCvCl	2015			Europe	Ireland	Enteritidis	Enteritidis	26465	500
SAL_FA5666AA	MS150056	traces-0XpIpow	2015			Europe	Ireland	Enteritidis	Enteritidis	1425	498
SAL_FA5670AA	15066-2014	traces-0DiqLlV				Europe	Slovenia	Enteritidis	Enteritidis	3941	494
SAL_FA5671AA	15070-2014	traces-0PthVOe				Europe	Slovenia	Enteritidis	Enteritidis	3941	493
SAL_FA5724AA	E2016005372	SRR3437476	2016	4		North America	United States	Enteritidis	Enteritidis	1425	447
SAL_FA5725AA	E2016005325	SRR3437475	2016	4		North America	United States	Enteritidis	Enteritidis	1425	446
SAL_FA5738AA	MS150004	traces-0cOlCST	2015			Europe	Ireland	Enteritidis	Enteritidis	3888	404
SAL_FA5755AA	NSW059	traces-0fCRfwj	2016			Oceania	Australia	Enteritidis	Enteritidis	1425	418
SAL_FA5756AA	NSW061	traces-0nmudVP	2016	4	27	Oceania	Australia	Enteritidis	Enteritidis	1425	417
SAL_FA5758AA	MS150020	traces-0MPlWcY	2015			Europe	Ireland	Enteritidis	Enteritidis	1425	415
SAL_FA5760AA	MS150016	traces-0UuqSLb	2015			Europe	Ireland	Enteritidis	Enteritidis	1425	413
SAL_FA5761AA	MS150050	traces-0VypkXB	2015			Europe	Ireland	Enteritidis	Enteritidis	1425	412
SAL_FA5763AA	MS150021	traces-0DnzsfD	2015			Europe	Ireland	Enteritidis	Enteritidis	3888	410
SAL_FA5767AA	MS150186	traces-0pEmkiB	2015			Europe	Ireland	Enteritidis	Enteritidis	1425	303
SAL_FA5771AA	MS150071	traces-0aWEscj	2015			Europe	Ireland	Enteritidis	Enteritidis	3888	404
SAL_FA5773AA	117968	ERR1370587						Enteritidis (Predicted)	Enteritidis	1425	402
SAL_FA5774AA	117967	ERR1370586						Enteritidis (Predicted)	Enteritidis	1425	402
SAL_FA5775AA	117961	ERR1370585						Enteritidis (Predicted)	Enteritidis	1425	402
SAL_FA5776AA	117960	ERR1370584						Enteritidis (Predicted)	Enteritidis	1425	402
SAL_FA5777AA	117927	ERR1370583						Enteritidis (Predicted)	Enteritidis	1425	402

SAL_FA5782AA	MS150109	traces-0lMdNxM	2015			Europe	Ireland	Enteritidis	Enteritidis	1425	397
SAL_FA5808AA	NSW070	traces-0KVJwHZ	2016	4	27	Oceania	Australia	Enteritidis	Enteritidis	28412	374
SAL_FA5817AA	E2016005458	SRR3457764	2016	4		North America	United States	Enteritidis	Enteritidis	1425	366
SAL_FA5818AA	E2016005476	SRR3457765	2016	4		North America	United States	Enteritidis	Enteritidis	1425	240
SAL_FA5819AA	AK1131900080	SRR3457709	2011	11		North America	United States	Enteritidis	Enteritidis	1425	365
SAL_FA5823AA	FDA00002756	SRR3457704	2008	6		South America	Peru	Enteritidis	Enteritidis	1425	361
SAL_FA5883AA	MS150236	traces-0GLQRll	2015			Europe	Ireland	Enteritidis	Enteritidis	1425	304
SAL_FA5884AA	MS150187	traces-0rOMpGZ	2015			Europe	Ireland	Enteritidis	Enteritidis	1425	303
SAL_FA5889AA	MS150278	traces-0JpiPUf	2015			Europe	Ireland	Enteritidis	Enteritidis	1425	298
SAL_FA5891AA	MS150266	traces-0ZaCLPv	2015			Europe	Ireland	Enteritidis	Enteritidis	1425	296
SAL_FA5892AA	MS150280	traces-0NPcjPm	2015			Europe	Ireland	Enteritidis	Enteritidis	1425	295
SAL_FA5893AA	MS150279	traces-0hTdmyE	2015			Europe	Ireland	Enteritidis	Enteritidis	1425	294
SAL_FA5897AA	MS150291	traces-0QxDFSH	2015			Europe	Ireland	Enteritidis	Enteritidis	36954	292
SAL_FA5957AA	E2016005692	SRR3465485	2016	4		North America	United States	Enteritidis	Enteritidis	1425	241
SAL_FA5958AA	E2016005675	SRR3465470	2016	4		North America	United States	Enteritidis	Enteritidis	1425	240
SAL_FA5959AA	E2016005628	SRR3465460	2016	4		North America	United States	Enteritidis	Enteritidis	1425	239
SAL_FA6023AA	PNUSAS002039	SRR3475681	2016	4		North America	United States	Enteritidis	Enteritidis	1425	186
SAL_FA6026AA	PNUSAS002027	SRR3475678				North America	United States	Enteritidis (Predicted)	Enteritidis	27863	184
SAL_FA6028AA	PNUSAS002025	SRR3475676	2016	3		North America	United States	Enteritidis	Enteritidis	1425	180
SAL_FA6029AA	PNUSAS002024	SRR3475675				North America	United States	Enteritidis (Predicted)	Enteritidis	1425	182
SAL_FA6031AA	PNUSAS002022	SRR3475673				North America	United States	Enteritidis (Predicted)	Enteritidis	1425	180
SAL_FA6032AA	PNUSAS002021	SRR3475671				North America	United States	Enteritidis (Predicted)	Enteritidis	1425	179
SAL_FA6034AA	PNUSAS002020	SRR3475669				North America	United States	Enteritidis (Predicted)	Enteritidis	37133	177
SAL_FA6040AA	PNUSAS002095	SRR3475663				North America	United States	Enteritidis (Predicted)	Enteritidis	1425	171
SAL_FA6042AA	PNUSAS001895	SRR3475661				North America	United States	Enteritidis (Predicted)	Enteritidis	1425	169
SAL_FA6044AA	PNUSAS001893	SRR3475659				North America	United States	Enteritidis (Predicted)	Enteritidis	1425	167
SAL_FA6045AA	PNUSAS001892	SRR3475658				North America	United States	Enteritidis (Predicted)	Enteritidis	1425	166
SAL_FA6046AA	PNUSAS001891	SRR3475657				North America	United States	Enteritidis (Predicted)	Enteritidis	1425	165
SAL_FA6048AA	PNUSAS001747	SRR3475655				North America	United States	Enteritidis (Predicted)	Enteritidis	1425	163
SAL_FA6051AA	PNUSAS001742	SRR3475652				North America	United States	Enteritidis (Predicted)	Enteritidis	1425	160
SAL_FA6060AA	PNUSAS001858	SRR3475642	2016	2		North America	United States	Enteritidis	Enteritidis	1425	151
SAL_FA6061AA	PNUSAS001857	SRR3475639	2016	1		North America	United States	Enteritidis	Enteritidis	1425	150
SAL_FA6100AA	PNUSAS001851	SRR3473926				North America	United States	Enteritidis	Enteritidis	1425	112
SAL_FA6126AA	PNUSAS002053	SRR3473898	2016	3		North America	United States	Enteritidis	Enteritidis	1425	87
SAL_FA6128AA	PNUSAS002051	SRR3473896	2016	3		North America	United States	Enteritidis	Enteritidis	1425	85
SAL_FA6130AA	PNUSAS001998	SRR3473894				North America	United States	Enteritidis (Predicted)	Enteritidis	1425	83
SAL_FA6131AA	PNUSAS001997	SRR3473893				North America	United States	Enteritidis (Predicted)	Enteritidis	27901	82
SAL_FA6187AA	FSIS1606338	SRR3476370	2016			North America	United States	Enteritidis	Enteritidis	1425	27
SAL_FA6202AA	FSIS1503700	SRR3475939	2015			North America	United States	Enteritidis	Enteritidis	1425	12
SAL_FA6227AA	CFSAN032104	SRR3479657	2014	1		North America	United States	Enteritidis	Enteritidis	1425	18698
SAL_FA6284AA	S/20160357	traces-0uCJiea	2016	4	6	Europe	United Kingdom	Enteritidis	Enteritidis	3888	39554
SAL_FA6285AA	S/20160335	traces-0oZNxMV	2016	4	6	Europe	United Kingdom	Enteritidis	Enteritidis	3888	39556
SAL_FA6286AA	S/20160309	traces-0JytDoE	2016	3	31	Europe	United Kingdom	Enteritidis	Enteritidis	3888	39556
SAL_FA6287AA	S/20160344	traces-0VuxFqF	2016	4	7	Europe	United Kingdom	Enteritidis	Enteritidis	3888	39555
SAL_FA6294AA	2016K-0174	SRR3489712				North America	United States	Enteritidis (Predicted)	Enteritidis	1425	3680
SAL_FA6363AA	CFSAN049417	SRR3490045	2016	4		North America	United States	Enteritidis	Enteritidis	1425	39607
SAL_FA6364AA	CFSAN049416	SRR3490044	2016	4		North America	United States	Enteritidis	Enteritidis	1425	39606
SAL_FA6365AA	CFSAN049415	SRR3490043	2016	4		North America	United States	Enteritidis	Enteritidis	1425	39605
SAL_FA6379AA	I2016005854	SRR3490027	2016	4		North America	United States	Enteritidis	Enteritidis	1425	39582
SAL_FA6380AA	I2016005745	SRR3490026	2016	4		North America	United States	Enteritidis	Enteritidis	1425	39581
SAL_FA6381AA	I2016005701-2	SRR3490025	2016	4		North America	United States	Enteritidis	Enteritidis	3888	39580
SAL_FA6382AA	E2016006266	SRR3509910	2016	4		North America	United States	Enteritidis	Enteritidis	1425	39579
SAL_FA6383AA	E2016005959	SRR3490023	2016	4		North America	United States	Enteritidis	Enteritidis	1425	39578
SAL_FA6384AA	E2016005950	SRR3490022	2016	4		North America	United States	Enteritidis	Enteritidis	34197	39577
SAL_FA6385AA	E2016005931	SRR3490021	2016	4		North America	United States	Enteritidis	Enteritidis	1425	39576
SAL_FA6386AA	E2016005845	SRR3490020	2016	4		North America	United States	Enteritidis	Enteritidis	1425	39575
SAL_FA6387AA	E2016005766	SRR3490019	2016	4		North America	United States	Enteritidis	Enteritidis	1425	39574
SAL_FA6388AA	E2016005761	SRR3490018	2016	4		North America	United States	Enteritidis	Enteritidis	1425	39573
SAL_FA6389AA	I2016005972	SRR3490017	2016	4		North America	United States	Enteritidis	Enteritidis	3888	39588

SAL_FA6390AA	E2016006522	SRR3490016	2016	4		North America	United States	Enteritidis	Enteritidis	1425	39587
SAL_FA6391AA	E2016006515	SRR3490015	2016	4		North America	United States	Enteritidis	Enteritidis	1425	39586
SAL_FA6392AA	E2016006274	SRR3490013	2016	4		North America	United States	Enteritidis	Enteritidis	1425	39572
SAL_FA6393AA	E2016006269	SRR3490012	2016	4		North America	United States	Enteritidis	Enteritidis	1425	447
SAL_FA6415AA	PNUSAS001773	SRR3495149				North America	United States	Enteritidis (Predicted)	Enteritidis	1425	39641
SAL_FA6421AA	PNUSAS002115	SRR3495143	2016	4		North America	United States	Enteritidis	Enteritidis	1425	39635
SAL_FA6422AA	PNUSAS002116	SRR3495142	2016	4		North America	United States	Enteritidis	Enteritidis	1425	39630
SAL_FA6424AA	PNUSAS002118	SRR3495139	2016	3		North America	United States	Enteritidis	Enteritidis	37741	39633
SAL_FA6425AA	PNUSAS002119	SRR3495137	2016	4		North America	United States	Enteritidis (Predicted)	Enteritidis	1425	39629
SAL_FA6446AA	NSW071	traces-0abgYmm	2016	5	11	Oceania	Australia	Enteritidis	Enteritidis	1425	25840
SAL_FA6447AA	NSW063	traces-0cGhVTQ	2016	4	27	Oceania	Australia	Enteritidis	Enteritidis	1425	39701
SAL_FA6448AA	NSW064	traces-0ZyjWEu	2016	4	27	Oceania	Australia	Enteritidis	Enteritidis	1425	25840
SAL_FA6450AA	NSW062	traces-0XvcwFG	2016	4	27	Oceania	Australia	Enteritidis	Enteritidis	1425	39676
SAL_FA6451AA	NSW072	traces-0ojgIcj	2016	5	11	Oceania	Australia	Enteritidis	Enteritidis	1425	39675
SAL_FA6452AA	NSW069	traces-0jcbehO	2016	4	27	Oceania	Australia	Enteritidis	Enteritidis	1425	39674
SAL_FA6455AA	NSW073	traces-0zGVDfj	2016	5	11	Oceania	Australia	Enteritidis	Enteritidis	1425	39699
SAL_FA6456AA	NSW074	traces-0RNdEGM	2016	5	11	Oceania	Australia	Enteritidis	Enteritidis	1425	39698
SAL_FA6457AA	NSW075	traces-0nSYPIe	2016	5	11	Oceania	Australia	Enteritidis	Enteritidis	1425	39697
SAL_FA6459AA	NSW077	traces-0TuBmrg	2016	5	11	Oceania	Australia	Enteritidis	Enteritidis	1425	39674
SAL_FA6517AA	NSW056	traces-0idrIxI	2015			Oceania	Australia	Enteritidis	Enteritidis	1425	39775
SAL_FA6518AA	NSW060	traces-0IDQQLv	2016	4	27	Oceania	Australia	Enteritidis	Enteritidis	1425	39774
SAL_FA6520AA	NSW078	traces-0pfxCdF	2016	5	12	Oceania	Australia	Enteritidis	Enteritidis	1425	39772
SAL_FA6521AA	NSW083	traces-0JnMuHq	2016	5	12	Oceania	Australia	Enteritidis	Enteritidis	1425	39771
SAL_FA6522AA	NSW086	traces-0VRWbFp	2016	5	12	Oceania	Australia	Enteritidis	Enteritidis	1425	39770
SAL_FA6523AA	NSW090	traces-0jQKyPf	2016	5	12	Oceania	Australia	Enteritidis	Enteritidis	37848	39769
SAL_FA6524AA	NSW093	traces-0NUJeFj	2016	5	12	Oceania	Australia	Enteritidis	Enteritidis	1425	39757
SAL_FA6525AA	NSW116	traces-0pRwHdR	2016	5	12	Oceania	Australia	Enteritidis	Enteritidis	37847	39768
SAL_FA6526AA	NSW162	traces-0bYcQqn	2016	5	12	Oceania	Australia	Enteritidis	Enteritidis	1425	39767
SAL_FA6527AA	NSW163	traces-0lJsDtW	2016	5	12	Oceania	Australia	Enteritidis	Enteritidis	1425	39766
SAL_FA6529AA	NSW174	traces-0owSDgm	2016	5	12	Oceania	Australia	Enteritidis	Enteritidis	37849	39765
SAL_FA6530AA	NSW 165	traces-0AhamTE	2016	5	13	Oceania	Australia	Enteritidis	Enteritidis	1425	39764
SAL_FA6531AA	NSW080	traces-0jTVaau	2016	5	12	Oceania	Australia	Enteritidis	Enteritidis	1425	39763
SAL_FA6532AA	NSW079	traces-0PvGVGl	2016	5	12	Oceania	Australia	Enteritidis	Enteritidis	1425	39762
SAL_FA6533AA	NSW081	traces-0SqBZGX	2016	5	12	Oceania	Australia	Enteritidis	Enteritidis	1425	39758
SAL_FA6534AA	NSW082	traces-0TbNZrn	2016	5	12	Oceania	Australia	Enteritidis	Enteritidis	1425	39761
SAL_FA6535AA	NSW085	traces-0iixDeu	2016	5	12	Oceania	Australia	Enteritidis	Enteritidis	1425	39760
SAL_FA6536AA	NSW084	traces-0PqZGrp	2016	5	12	Oceania	Australia	Enteritidis	Enteritidis	1425	39787
SAL_FA6537AA	NSW087	traces-0DpCoOD	2016	5	12	Oceania	Australia	Enteritidis	Enteritidis	1425	39786
SAL_FA6538AA	NSW089	traces-0qKCeFC	2016	5	12	Oceania	Australia	Enteritidis	Enteritidis	1425	39757
SAL_FA6539AA	NSW091	traces-0hseeLw	2016	5	12	Oceania	Australia	Enteritidis	Enteritidis	1425	39785
SAL_FA6540AA	NSW092	traces-0ajIZEk	2016	5	12	Oceania	Australia	Enteritidis	Enteritidis	1425	39784
SAL_FA6541AA	NSW088	traces-0DoZmbP	2016	5	12	Oceania	Australia	Enteritidis	Enteritidis	1425	39757
SAL_FA6542AA	NSW105	traces-0GvzhrG	2016	5	12	Oceania	Australia	Enteritidis	Enteritidis	7942	39788
SAL_FA6543AA	NSW164	traces-0SOoIzC	2016	5	12	Oceania	Australia	Enteritidis	Enteritidis	1425	39798
SAL_FA6544AA	NSW153	traces-0SSDedo	2016	5	12	Oceania	Australia	Enteritidis	Enteritidis	1425	39797
SAL_FA6546AA	NSW103	traces-0BYaPQf	2016	5	12	Oceania	Australia	Enteritidis	Enteritidis	1425	39795
SAL_FA6549AA	NSW117	traces-0LtXzMx	2016	5	12	Oceania	Australia	Enteritidis	Enteritidis	7942	39788
SAL_FA6551AA	NSW152	traces-0pZCBHb	2016	5	12	Oceania	Australia	Enteritidis	Enteritidis	1425	39818
SAL_FA6552AA	NSW096	traces-0aTxKDh	2016	5	12	Oceania	Australia	Enteritidis	Enteritidis	1425	39817
SAL_FA6553AA	NSW095	traces-0IUbQMf	2016	5	12	Oceania	Australia	Enteritidis	Enteritidis	1425	39816
SAL_FA6554AA	NSW104	traces-0gOLUHh	2016	5	12	Oceania	Australia	Enteritidis	Enteritidis	1425	39815
SAL_FA6556AA	NSW139	traces-0DJKCtc	2016	5	12	Oceania	Australia	Enteritidis	Enteritidis	1425	39813
SAL_FA6558AA	NSW140	traces-0AnfFGC	2016	5	12	Oceania	Australia	Enteritidis	Enteritidis	1425	39838
SAL_FA6559AA	NSW151	traces-0kBiOoA	2016	5	12	Oceania	Australia	Enteritidis	Enteritidis	1425	39837
SAL_FA6560AA	NSW177	traces-0TlxdUL	2016	5	12	Oceania	Australia	Enteritidis	Enteritidis	37843	39836
SAL_FA6561AA	NSW176	traces-0ztyOLD	2016	5	12	Oceania	Australia	Enteritidis non-motile	Enteritidis	1425	39835
SAL_FA6562AA	NSW175	traces-0YLhwui	2016	5	12	Oceania	Australia	Enteritidis	Enteritidis	1425	39834
SAL_FA6563AA	NSW186	traces-0nqojSH	2016	5	12	Oceania	Australia	Enteritidis	Enteritidis	37736	39876
SAL_FA6569AA	2016K-0279	SRR3499803				North America	United States	Enteritidis (Predicted)	Enteritidis	1425	39864

SAL_FA6570AA	2016K-0278	SRR3499802				North America	United States	Enteritidis (Predicted)	Enteritidis	1425	39869
SAL_FA6571AA	2016K-0277	SRR3499801				North America	United States	Enteritidis (Predicted)	Enteritidis	1425	39863
SAL_FA6572AA	2016K-0275	SRR3499799				North America	United States	Enteritidis (Predicted)	Enteritidis	1425	39868
SAL_FA6573AA	2016K-0274	SRR3499798				North America	United States	Enteritidis (Predicted)	Enteritidis	1425	165
SAL_FA6574AA	2016K-0285	SRR3499797				North America	United States	Enteritidis (Predicted)	Enteritidis	1425	39867
SAL_FA6575AA	2016K-0284	SRR3499796				North America	United States	Enteritidis (Predicted)	Enteritidis	1425	39866
SAL_FA6576AA	2016K-0283	SRR3499795				North America	United States	Enteritidis (Predicted)	Enteritidis	1425	39862
SAL_FA6577AA	2016K-0282	SRR3499794				North America	United States	Enteritidis (Predicted)	Enteritidis	1425	39861
SAL_FA6578AA	2016K-0281	SRR3499793	2016	1		North America	United States	Enteritidis (Predicted)	Enteritidis	1425	36319
SAL_FA6579AA	2016K-0280	SRR3499792				North America	United States	Enteritidis (Predicted)	Enteritidis	1425	39860
SAL_FA6582AA	PNUSAS002041	SRR3499789	2016	4		North America	United States	Enteritidis	Enteritidis	37133	39859
SAL_FA6583AA	PNUSAS002040	SRR3499788	2016	4		North America	United States	Enteritidis	Enteritidis	1425	39856
SAL_FA6586AA	PNUSAS002106	SRR3499785	2016	3		North America	United States	Enteritidis	Enteritidis	1425	39853
SAL_FA6588AA	PNUSAS002103	SRR3499783	2016	1		North America	United States	Enteritidis	Enteritidis	1425	35158
SAL_FA6589AA	PNUSAS002101	SRR3499782	2016	1		North America	United States	Enteritidis	Enteritidis	1425	39858
SAL_FA6590AA	PNUSAS002100	SRR3499781	2015	11		North America	United States	Enteritidis	Enteritidis	1425	39857
SAL_FA6592AA	PNUSAS002140	SRR3499779	2016	4		North America	United States	Enteritidis	Enteritidis	1425	39850
SAL_FA6596AA	PNUSAS002138	SRR3499775				North America	United States	Enteritidis (Predicted)	Enteritidis	1425	180
SAL_FA6599AA	PNUSAS002065	SRR3499772				North America	United States	Enteritidis (Predicted)	Enteritidis	1425	39810
SAL_FA6600AA	PNUSAS002064	SRR3499771				North America	United States	Enteritidis (Predicted)	Enteritidis	1425	180
SAL_FA6601AA	PNUSAS002063	SRR3499770				North America	United States	Enteritidis (Predicted)	Enteritidis	1425	39808
SAL_FA6602AA	PNUSAS002062	SRR3499769				North America	United States	Enteritidis (Predicted)	Enteritidis	1425	39807
SAL_FA6603AA	PNUSAS002061	SRR3499768				North America	United States	Enteritidis (Predicted)	Enteritidis	1425	39809
SAL_FA6604AA	PNUSAS002060	SRR3499767				North America	United States	Enteritidis (Predicted)	Enteritidis	1425	180
SAL_FA6605AA	PNUSAS002059	SRR3499766				North America	United States	Enteritidis (Predicted)	Enteritidis	1425	39806
SAL_FA6606AA	PNUSAS002058	SRR3499765				North America	United States	Enteritidis (Predicted)	Enteritidis	1425	180
SAL_FA6607AA	PNUSAS002057	SRR3499764	2016	3		North America	United States	Enteritidis	Enteritidis	1425	39805
SAL_FA6610AA	PNUSAS002130	SRR3499745	2016	4		North America	United States	Enteritidis	Enteritidis	1425	39802
SAL_FA6611AA	PNUSAS002129	SRR3499744	2016	3		North America	United States	Enteritidis	Enteritidis	1425	39783
SAL_FA6618AA	PNUSAS001785	SRR3499736	2016	2		North America	United States	Enteritidis	Enteritidis	1425	39799
SAL_FA6621AA	PNUSAS002143	SRR3499733	2016	4		North America	United States	Enteritidis	Enteritidis	1425	39779
SAL_FA6622AA	PNUSAS002142	SRR3499732	2016	4		North America	United States	Enteritidis	Enteritidis	1425	39778
SAL_FA6624AA	PNUSAS002066	SRR3499730	2016	4		North America	United States	Enteritidis	Enteritidis	1425	39776
SAL_FA6661AA	2016K-0276	SRR3499800				North America	United States	Enteritidis (Predicted)	Enteritidis	1425	39790
SAL_FA6663AA	RKI_15-03486	traces-0WJBIhz	2015			Europe	Germany	Enteritidis	Enteritidis	1425	39875
SAL_FA6664AA	RKI_15-03455	traces-0GoIukp	2015			Europe	Germany	Enteritidis	Enteritidis	1425	39875
SAL_FA6665AA	RKI_15-03487	traces-0KXjcUv	2015			Europe	Germany	Enteritidis	Enteritidis	1425	39875
SAL_FA6668AA	CVM-N27408	SRR3503030	2010			North America	United States	Enteritidis	Enteritidis	1425	39885
SAL_FA6674AA	CVM-N27396	SRR3502966	2010			North America	United States	Enteritidis	Enteritidis	1425	12469
SAL_FA6760AA	RKI_15-01563	traces-0eaumVN	2015			Europe	Germany	Enteritidis	Enteritidis	3961	39904
SAL_FA6761AA	RKI_15-03374	traces-0BTfEXp	2015			Europe	Germany	Enteritidis	Enteritidis	1425	39903
SAL_FA6762AA	RKI_14-04275	traces-0qBZIrz	2014			Europe	Germany	Enteritidis	Enteritidis	1425	39905
SAL_FA6763AA	RKI_15-03456	traces-0TVyhxt	2015			Europe	Germany	Enteritidis	Enteritidis	1425	39875
SAL_FA6764AA	RKI_15-03375	traces-0LEeHds	2015			Europe	Germany	Enteritidis	Enteritidis	1425	39875
SAL_FA6765AA	RKI_15-03373	traces-0DrgpKV	2015			Europe	Germany	Enteritidis	Enteritidis	1425	39875
SAL_FA6803AA	PNUSAS001776	SRR3521385				North America	United States	Enteritidis (Predicted)	Enteritidis	1425	39946
SAL_FA6817AA	I2016006669	SRR3509923	2016	5		North America	United States	Enteritidis	Enteritidis	1425	39932
SAL_FA6818AA	I2016006642	SRR3509922	2016	5		North America	United States	Enteritidis	Enteritidis	1425	39931
SAL_FA6819AA	I2016006566	SRR3571304	2016	5		North America	United States	Enteritidis	Enteritidis	1425	39930
SAL_FA6820AA	I2016006553	SRR3509919	2016	4		North America	United States	Enteritidis	Enteritidis	1425	39929
SAL_FA6821AA	E2016006813	SRR3571301	2016	5		North America	United States	Enteritidis	Enteritidis	1425	39928
SAL_FA6830AA	CVM-N28367	SRR3509889	2010			North America	United States	Enteritidis	Enteritidis	1425	39919
SAL_FA6835AA	CVM-N27419	SRR3509882	2010			North America	United States	Enteritidis	Enteritidis	1425	39910
SAL_FA6837AA	CVM-N27417	SRR3509874	2010			North America	United States	Enteritidis	Enteritidis	1425	39909
SAL_FA6846AA	NSW187	traces-0FUdwGL	2016	5	18	Oceania	Australia	Enteritidis	Enteritidis	1425	39993
SAL_FA6849AA	NSW048	traces-0nkkKQp	2016	5	18			Enteritidis	Enteritidis	1425	39991
SAL_FA6850AA	NSW049	traces-0gEizau	2016	5	18	Oceania	Australia	Enteritidis	Enteritidis	1425	39990
SAL_FA6851AA	NSW050	traces-0HLJYvN	2016	5	18	Oceania	Australia	Enteritidis	Enteritidis	1425	16325
SAL_FA6852AA	NSW051	traces-0MJgsan	2016	5	18	Oceania	Australia	Enteritidis	Enteritidis	1425	39989

SAL_FA6853AA	NSW052	traces-0OVexSJ	2016	5	18	Oceania	Australia	Enteritidis	Enteritidis	1425	651
SAL_FA6855AA	NSW054	traces-0XgKUPm	2016	5	18	Oceania	Australia	Enteritidis	Enteritidis	1425	39987
SAL_FA6856AA	NSW29-055	traces-0efxQoM	2016	5	18	Oceania	Australia	Enteritidis	Enteritidis	1425	39986
SAL_FA6857AA	NSW29-056	traces-0tPDBPV	2016	5	18	Oceania	Australia	Enteritidis	Enteritidis	27796	39985
SAL_FA6858AA	NSW29-057	traces-0aeFODc	2016	5	18	Oceania	Australia	Enteritidis	Enteritidis	1425	39984
SAL_FA6859AA	NSW29-058	traces-0wmAwhM	2016	5	18	Oceania	Australia	Enteritidis	Enteritidis	38220	39983
SAL_FA6860AA	NSW29-059	traces-0TzZDEz	2016	5	18	Oceania	Australia	Enteritidis	Enteritidis	1425	39982
SAL_FA6861AA	NSW29-060	traces-0YNwRsS	2016	5	18	Oceania	Australia	Enteritidis	Enteritidis	1425	39981
SAL_FA6862AA	NSW29-061	traces-0LqDcbl	2016	5	18	Oceania	Australia	Enteritidis	Enteritidis	28689	39980
SAL_FA6865AA	NSW29-064	traces-0bNEGjC	2016	5	18	Oceania	Australia	Enteritidis	Enteritidis	1425	39977
SAL_FA6869AA	NSW29-068	traces-0isVqBY	2016	5	18			Enteritidis	Enteritidis	1425	40008
SAL_FA6872AA	NSW29-071	traces-0wNIEAs	2016	5	18	Oceania	Australia	Enteritidis	Enteritidis	1425	40005
SAL_FA6873AA	NSW29-072	traces-0sOxEJe	2016	5	18	Oceania	Australia	Enteritidis	Enteritidis	1425	7578
SAL_FA6874AA	NSW29-072	traces-0RwjTYb	2016	5	18	Oceania	Australia	Enteritidis	Enteritidis	1425	7578
SAL_FA6875AA	NSW29-073	traces-0NpChRa	2016	5	18	Oceania	Australia	Enteritidis	Enteritidis	1425	40004
SAL_FA6877AA	NSW29-075	traces-0rIRjPd	2016	5	18	Oceania	Australia	Enteritidis	Enteritidis	1425	40002
SAL_FA6878AA	NSW29-086	traces-0QPChTK	2016	5	18	Oceania	Australia	Enteritidis	Enteritidis	38332	40001
SAL_FA6879AA	NSW29-087	traces-0mcovUs	2016	5	18	Oceania	Australia	Enteritidis	Enteritidis	37843	40000
SAL_FA6880AA	NSW29-088	traces-0DYSdcc	2016	5	18	Oceania	Australia	Enteritidis	Enteritidis	1425	39999
SAL_FA6881AA	NSW29-089	traces-0KAawPl	2016	5	18	Oceania	Australia	Enteritidis	Enteritidis	7942	39788
SAL_FA6882AA	NSW12-072	traces-0Cifnuc	2016	5	18	Oceania	Australia	Enteritidis	Enteritidis	7942	39998
SAL_FA6885AA	NSW12-077	traces-0ckAkzJ	2016	5	18	Oceania	Australia	Enteritidis	Enteritidis	1425	39995
SAL_FA6900AA	RKI_15-03580	traces-0YpbnDL	2015			Europe	Germany	Enteritidis	Enteritidis	1425	40023
SAL_FA6901AA	RKI_15-03489	traces-0xyhvWF	2015			Europe	Germany	Enteritidis	Enteritidis	1425	39875
SAL_FA6902AA	RKI_15-03367	traces-0ZJkesv	2015			Europe	Germany	Enteritidis	Enteritidis	1425	39875
SAL_FA6903AA	RKI_15-03579_2	traces-0xvkjrf	2015			Europe	Germany	Enteritidis	Enteritidis	1425	40025
SAL_FA6904AA	RKI_15-03552	traces-0zXCrNj	2015			Europe	Germany	Enteritidis	Enteritidis	1425	39875
SAL_FA6905AA	RKI_15-03440	traces-0BvhXaE	2015			Europe	Germany	Enteritidis	Enteritidis	1425	39875
SAL_FA6906AA	RKI_15-03370	traces-0jQaHtq	2015			Europe	Germany	Enteritidis	Enteritidis	1425	39875
SAL_FA6907AA	RKI_15-03614	traces-0MBfFGG	2015			Europe	Germany	Enteritidis	Enteritidis	1425	39875
SAL_FA6908AA	RKI_15-03538	traces-0zQxlVL	2015			Europe	Germany	Enteritidis	Enteritidis	1425	40024
SAL_FA6909AA	RKI_15-03637	traces-0SUxbKa	2015			Europe	Germany	Enteritidis	Enteritidis	1425	40026
SAL_FA6910AA	RKI_15-03654	traces-0ujMbsn	2015			Europe	Germany	Enteritidis	Enteritidis	1425	40027
SAL_FA6924AA	CVM-N28611	SRR3547960	2010			North America	United States	Enteritidis	Enteritidis	1425	40030
SAL_FA6926AA	CVM-N28608	SRR3547958	2010			North America	United States	Enteritidis	Enteritidis	1425	40030
SAL_FA6961AA	H14330_0431	traces-0dquTvZ				Europe	United Kingdom	Enteritidis	Enteritidis	3888	2066
SAL_FA6962AA	H14346_0537	traces-0UwPlna				Europe	United Kingdom	Enteritidis	Enteritidis	3888	40066
SAL_FA6963AA	L00851-15	traces-0RBsedb				Europe	United Kingdom	Enteritidis (Predicted)	Enteritidis	1425	40042
SAL_FA6964AA	L00786-15	traces-0HhYcrH				Europe	United Kingdom	Enteritidis (Predicted)	Enteritidis	1425	40065
SAL_FA6965AA	L00999-15	traces-0wusDwN				Europe	United Kingdom	Enteritidis (Predicted)	Enteritidis	3888	40064
SAL_FA6966AA	L01100-13	traces-0VvBGsX				Europe	United Kingdom	Enteritidis (Predicted)	Enteritidis	3888	40063
SAL_FA6967AA	L01381-10	traces-0srtMpj				Europe	United Kingdom	Enteritidis (Predicted)	Enteritidis	1425	40062
SAL_FA6968AA	L01382-10	traces-0JZPjay				Europe	United Kingdom	Enteritidis (Predicted)	Enteritidis	1425	40061
SAL_FA6969AA	S00018-15	traces-0mKAsOB				Europe	United Kingdom	Enteritidis (Predicted)	Enteritidis	3888	40060
SAL_FA6970AA	S00095-14	traces-0dvePJQ				Europe	United Kingdom	Enteritidis (Predicted)	Enteritidis	3888	40059
SAL_FA6971AA	S00099-14	traces-0hQndUx				Europe	United Kingdom	Enteritidis (Predicted)	Enteritidis	3888	40058
SAL_FA6972AA	S00964-05	traces-0vIGpqG				Europe	United Kingdom	Enteritidis (Predicted)	Enteritidis	1425	40057
SAL_FA6973AA	S00432_16	traces-0skdtcu				Europe	United Kingdom	Enteritidis (Predicted)	Enteritidis	1425	40056
SAL_FA6974AA	S01041-15	traces-0EoORsu				Europe	United Kingdom	Enteritidis (Predicted)	Enteritidis	1425	40055
SAL_FA6975AA	S01909-15	traces-0OlZgtL				Europe	United Kingdom	Enteritidis (Predicted)	Enteritidis	1425	40054
SAL_FA6976AA	S02006-15	traces-0npRCMd				Europe	United Kingdom	Enteritidis (Predicted)	Enteritidis	1425	40053
SAL_FA6977AA	S02037-10	traces-0qJkjLv				Europe	United Kingdom	Enteritidis (Predicted)	Enteritidis	1425	40052
SAL_FA6978AA	S02014-15	traces-0TaNUHC				Europe	United Kingdom	Enteritidis (Predicted)	Enteritidis	1425	503
SAL_FA6979AA	S02159-15	traces-0CayCCr				Europe	United Kingdom	Enteritidis (Predicted)	Enteritidis	1425	503
SAL_FA6980AA	S02161-15	traces-0BngJhd				Europe	United Kingdom	Enteritidis (Predicted)	Enteritidis	1425	40051
SAL_FA6981AA	S02213-15	traces-0xvPFBm				Europe	United Kingdom	Enteritidis (Predicted)	Enteritidis	1425	40043
SAL_FA6982AA	S02160-15	traces-0AdhToW				Europe	United Kingdom	Enteritidis (Predicted)	Enteritidis	1425	503
SAL_FA6983AA	S02222-15	traces-0APlnzr				Europe	United Kingdom	Enteritidis (Predicted)	Enteritidis	1425	40050
SAL_FA6984AA	S02225-15	traces-0VBhArw				Europe	United Kingdom	Enteritidis (Predicted)	Enteritidis	1425	40044

SAL_FA6985AA	S02227-15	traces-0BIfAhv	Europe	United Kingdom	Enteritidis (Predicted)	Enteritidis	1425	40049
SAL_FA6986AA	S02273-15	traces-0dkCYLF	Europe	United Kingdom	Enteritidis (Predicted)	Enteritidis	1425	40048
SAL_FA6987AA	S02228-15	traces-0Idanhs	Europe	United Kingdom	Enteritidis (Predicted)	Enteritidis	1425	503
SAL_FA6988AA	S02440-15	traces-0ZpxmUZ	Europe	United Kingdom	Enteritidis (Predicted)	Enteritidis	1425	40047
SAL_FA6989AA	S02356-15	traces-0Hlcety	Europe	United Kingdom	Enteritidis (Predicted)	Enteritidis	1425	40045
SAL_FA6990AA	S02498-15	traces-0gvLxwh	Europe	United Kingdom	Enteritidis (Predicted)	Enteritidis	1425	40046
SAL_FA6991AA	S02665-15	traces-0sMwhys	Europe	United Kingdom	Enteritidis (Predicted)	Enteritidis	1425	40079
SAL_FA6993AA	S02576-15	traces-0eKSlan	Europe	United Kingdom	Enteritidis (Predicted)	Enteritidis	1425	40067
SAL_FA6994AA	S02669-15	traces-0mTURhY	Europe	United Kingdom	Enteritidis (Predicted)	Enteritidis	1425	40068
SAL_FA6995AA	S02674-15	traces-0LLlShN	Europe	United Kingdom	Enteritidis (Predicted)	Enteritidis	1425	503
SAL_FA6996AA	S02675-15	traces-0SMoUVK	Europe	United Kingdom	Enteritidis (Predicted)	Enteritidis	1425	503
SAL_FA6997AA	S02677-15	traces-0hZHozR	Europe	United Kingdom	Enteritidis (Predicted)	Enteritidis	1425	40070
SAL_FA6998AA	S02679-15	traces-0EIeKUZ	Europe	United Kingdom	Enteritidis (Predicted)	Enteritidis	1425	40069
SAL_FA6999AA	S02683-15	traces-0zFhYEF	Europe	United Kingdom	Enteritidis (Predicted)	Enteritidis	1425	40071
SAL_FA7000AA	S02681-15	traces-0Jknxvu	Europe	United Kingdom	Enteritidis (Predicted)	Enteritidis	38379	40072
SAL_FA7001AA	S02687-15	traces-0IPtpmU	Europe	United Kingdom	Enteritidis (Predicted)	Enteritidis	1425	40078
SAL_FA7002AA	S02690-15	traces-0XuPeLq	Europe	United Kingdom	Enteritidis (Predicted)	Enteritidis	1425	40073
SAL_FA7003AA	S02857-15	traces-0OCjshO	Europe	United Kingdom	Enteritidis (Predicted)	Enteritidis	1425	40075
SAL_FA7004AA	S03145-11	traces-0oPDuSP	Europe	United Kingdom	Enteritidis (Predicted)	Enteritidis	1425	40074
SAL_FA7005AA	S03570-15	traces-0TTPdsV	Europe	United Kingdom	Enteritidis (Predicted)	Enteritidis	38616	40076
SAL_FA7006AA	S03573-15	traces-0YaVEcy	Europe	United Kingdom	Enteritidis (Predicted)	Enteritidis	38616	40076
SAL_FA7007AA	S03734-07	traces-0rVUsSM	Europe	United Kingdom	Enteritidis (Predicted)	Enteritidis	1425	40080
SAL_FA7008AA	S04084-15	traces-0IeWVHN	Europe	United Kingdom	Enteritidis (Predicted)	Enteritidis	38616	40077
SAL_FA7009AA	S04085-15	traces-0VySHAx	Europe	United Kingdom	Enteritidis (Predicted)	Enteritidis	1425	503
SAL_FA7010AA	S04740-11	traces-0tUFehg	Europe	United Kingdom	Enteritidis (Predicted)	Enteritidis	1425	40118
SAL_FA7012AA	S05532-04	traces-0EcXsTj	Europe	United Kingdom	Enteritidis	Enteritidis	3888	40116
SAL_FA7013AA	S05859-09	traces-0IszkNX	Europe	United Kingdom	Enteritidis (Predicted)	Enteritidis	1425	40115
SAL_FA7017AA	S15-000930	traces-0FVVlIx	Europe	United Kingdom	Enteritidis (Predicted)	Enteritidis	1425	40114
SAL_FA7018AA	S15-001327	traces-0zMJTEy	Europe	United Kingdom	Enteritidis (Predicted)	Enteritidis	1425	40113
SAL_FA7019AA	S15-001649	traces-0gifXFk	Europe	United Kingdom	Enteritidis (Predicted)	Enteritidis	1425	40112
SAL_FA7020AA	S15-002286	traces-0MyRYtP	Europe	United Kingdom	Enteritidis (Predicted)	Enteritidis	1425	503
SAL_FA7021AA	S15-002695	traces-0HPIZfF	Europe	United Kingdom	Enteritidis (Predicted)	Enteritidis	1425	40111
SAL_FA7022AA	S15-1151	traces-0SITcUx	Europe	United Kingdom	Enteritidis (Predicted)	Enteritidis	1425	40110
SAL_FA7023AA	S15-1217	traces-0mWWgyX	Europe	United Kingdom	Enteritidis (Predicted)	Enteritidis	1425	40109
SAL_FA7025AA	S15-1364L	traces-0qOuLMr	Europe	United Kingdom	Enteritidis (Predicted)	Enteritidis	1425	40108
SAL_FA7026AA	S15-1765	traces-0njMsCH	Europe	United Kingdom	Enteritidis (Predicted)	Enteritidis	1425	40107
SAL_FA7028AA	L00758-15	traces-0WqOepE	Europe	United Kingdom	Enteritidis (Predicted)	Enteritidis	1425	40106
SAL_FA7029AA	L00416-10	traces-0nanEbU	Europe	United Kingdom	Enteritidis (Predicted)	Enteritidis	1425	40105
SAL_FA7030AA	L00759-15	traces-0nehBfu	Europe	United Kingdom	Enteritidis (Predicted)	Enteritidis	1425	40104
SAL_FA7031AA	L00781-15	traces-0RErxnT	Europe	United Kingdom	Enteritidis (Predicted)	Enteritidis	1425	40103
SAL_FA7032AA	S00023-14	traces-0fGKjSZ	Europe	United Kingdom	Enteritidis (Predicted)	Enteritidis	3888	40102
SAL_FA7033AA	L02106-15	traces-0IyrNFC	Europe	United Kingdom	Enteritidis (Predicted)	Enteritidis	1425	40101
SAL_FA7034AA	S00172-05	traces-0YHOYzc	Europe	United Kingdom	Enteritidis (Predicted)	Enteritidis	1425	40100
SAL_FA7035AA	S00994-15	traces-0XfjcNm	Europe	United Kingdom	Enteritidis (Predicted)	Enteritidis	1425	40099
SAL_FA7036AA	L01193-15	traces-0qFcitd	Europe	United Kingdom	Enteritidis (Predicted)	Enteritidis	1425	503
SAL_FA7037AA	S00854-15	traces-0HMtOSo	Europe	United Kingdom	Enteritidis (Predicted)	Enteritidis	1425	40098
SAL_FA7038AA	S02007-15	traces-0qnBGfK	Europe	United Kingdom	Enteritidis (Predicted)	Enteritidis	1425	40097
SAL_FA7039AA	S01239-12	traces-0KYESKn	Europe	United Kingdom	Enteritidis (Predicted)	Enteritidis	3888	40096
SAL_FA7040AA	S00993-15	traces-0JoQVvD	Europe	United Kingdom	Enteritidis (Predicted)	Enteritidis	1425	40095
SAL_FA7041AA	S02013-15	traces-0gHesNJ	Europe	United Kingdom	Enteritidis (Predicted)	Enteritidis	1425	40094
SAL_FA7042AA	S02215-15	traces-0lAQvNh	Europe	United Kingdom	Enteritidis (Predicted)	Enteritidis	1425	40093
SAL_FA7043AA	S02217-15	traces-0JiFcOa	Europe	United Kingdom	Enteritidis (Predicted)	Enteritidis	1425	40092
SAL_FA7044AA	S02226-15	traces-0paAnSR	Europe	United Kingdom	Enteritidis (Predicted)	Enteritidis	1425	40091
SAL_FA7045AA	S02229-15	traces-0eunffe	Europe	United Kingdom	Enteritidis (Predicted)	Enteritidis	1425	40090
SAL_FA7046AA	S02274-15	traces-0Emmmim	Europe	United Kingdom	Enteritidis (Predicted)	Enteritidis	1425	40089
SAL_FA7047AA	S02220-15	traces-0UVmJmi	Europe	United Kingdom	Enteritidis (Predicted)	Enteritidis	1425	40088
SAL_FA7048AA	S02158-15	traces-0KqNFTS	Europe	United Kingdom	Enteritidis (Predicted)	Enteritidis	1425	503
SAL_FA7049AA	S02276-15	traces-0UJQNaR	Europe	United Kingdom	Enteritidis (Predicted)	Enteritidis	1425	40087
SAL_FA7050AA	S02441-15	traces-0jBeGCk	Europe	United Kingdom	Enteritidis (Predicted)	Enteritidis	1425	40086

SAL_FA7051AA	S02355-15	traces-0BuzZnH			Europe	United Kingdom	Enteritidis (Predicted)	Enteritidis	1425	40085
SAL_FA7052AA	S02457-15	traces-0rHYeHr			Europe	United Kingdom	Enteritidis (Predicted)	Enteritidis	1425	40084
SAL_FA7053AA	S02463-15	traces-0gQtKGu			Europe	United Kingdom	Enteritidis (Predicted)	Enteritidis	1425	40083
SAL_FA7054AA	S02508-15	traces-0YLeXYH			Europe	United Kingdom	Enteritidis (Predicted)	Enteritidis	1425	40082
SAL_FA7055AA	S02671-15	traces-0LBLBYy			Europe	United Kingdom	Enteritidis (Predicted)	Enteritidis	1425	40081
SAL_FA7056AA	S02670-15	traces-0uBvONG			Europe	United Kingdom	Enteritidis (Predicted)	Enteritidis	1425	40130
SAL_FA7057AA	S02511-15	traces-0zxJEqB			Europe	United Kingdom	Enteritidis (Predicted)	Enteritidis	1425	40129
SAL_FA7058AA	S02668-15	traces-0XUotob			Europe	United Kingdom	Enteritidis (Predicted)	Enteritidis	1425	40128
SAL_FA7060AA	S02672-15	traces-0DtnCaG			Europe	United Kingdom	Enteritidis (Predicted)	Enteritidis	1425	40127
SAL_FA7061AA	S02680-15	traces-0JSBRgM			Europe	United Kingdom	Enteritidis (Predicted)	Enteritidis	1425	40126
SAL_FA7062AA	S02682-15	traces-0rarDnC			Europe	United Kingdom	Enteritidis (Predicted)	Enteritidis	1425	40160
SAL_FA7063AA	S02673-15	traces-0YkWfbJ			Europe	United Kingdom	Enteritidis (Predicted)	Enteritidis	1425	503
SAL_FA7064AA	S02678-15	traces-0IvbuKf			Europe	United Kingdom	Enteritidis (Predicted)	Enteritidis	1425	40159
SAL_FA7065AA	S02676-15	traces-0vLuRrp			Europe	United Kingdom	Enteritidis (Predicted)	Enteritidis	1425	40158
SAL_FA7067AA	S02684-15	traces-0isXhMd			Europe	United Kingdom	Enteritidis (Predicted)	Enteritidis	1425	40157
SAL_FA7068AA	S02686-15	traces-0FoeaYc			Europe	United Kingdom	Enteritidis (Predicted)	Enteritidis	1425	40125
SAL_FA7069AA	S02688-15	traces-0BlEJce			Europe	United Kingdom	Enteritidis (Predicted)	Enteritidis	1425	40119
SAL_FA7070AA	S02691-15	traces-0JYCFCr			Europe	United Kingdom	Enteritidis (Predicted)	Enteritidis	1425	40124
SAL_FA7071AA	S02832-15	traces-0HBIKHF			Europe	United Kingdom	Enteritidis (Predicted)	Enteritidis	1425	40123
SAL_FA7072AA	S02689-15	traces-0BKcdFd			Europe	United Kingdom	Enteritidis (Predicted)	Enteritidis	1425	40120
SAL_FA7073AA	S02833-15	traces-0LGMSia			Europe	United Kingdom	Enteritidis (Predicted)	Enteritidis	1425	40121
SAL_FA7074AA	S02858-15	traces-0eEArUv			Europe	United Kingdom	Enteritidis (Predicted)	Enteritidis	1425	40122
SAL_FA7075AA	S02860-15	traces-0kxtEud			Europe	United Kingdom	Enteritidis (Predicted)	Enteritidis	1425	40145
SAL_FA7076AA	S02856-15	traces-0WrkUnT			Europe	United Kingdom	Enteritidis (Predicted)	Enteritidis	1425	40144
SAL_FA7077AA	S03342-11	traces-0FQiamq			Europe	United Kingdom	Enteritidis (Predicted)	Enteritidis	1425	40143
SAL_FA7078AA	S03351-15	traces-0PrHXVP			Europe	United Kingdom	Enteritidis (Predicted)	Enteritidis	1425	40131
SAL_FA7079AA	S03669-15	traces-0KkfquQ			Europe	United Kingdom	Enteritidis (Predicted)	Enteritidis	1425	40142
SAL_FA7080AA	S03571-15	traces-0OIHvNv			Europe	United Kingdom	Enteritidis (Predicted)	Enteritidis	38616	40156
SAL_FA7081AA	S03742-15	traces-0DOnKNv			Europe	United Kingdom	Enteritidis (Predicted)	Enteritidis	1425	40141
SAL_FA7082AA	S03572-15	traces-0ioKFpb			Europe	United Kingdom	Enteritidis (Predicted)	Enteritidis	38616	40076
SAL_FA7083AA	S02862-15	traces-0YbYsEn			Europe	United Kingdom	Enteritidis (Predicted)	Enteritidis	1425	503
SAL_FA7084AA	S04055-15	traces-0GbpqeE			Europe	United Kingdom	Enteritidis (Predicted)	Enteritidis	1425	40155
SAL_FA7085AA	S04086-15	traces-0htNKHg			Europe	United Kingdom	Enteritidis (Predicted)	Enteritidis	38616	40076
SAL_FA7086AA	S05-0398	traces-0YZEFOx			Europe	United Kingdom	Enteritidis (Predicted)	Enteritidis	38613	48250
SAL_FA7088AA	S05658-06	traces-0ZXOtHO			Europe	United Kingdom	Enteritidis (Predicted)	Enteritidis	1425	40152
SAL_FA7089AA	S05-000397	traces-0qmhaph			Europe	United Kingdom	Enteritidis (Predicted)	Enteritidis	1425	40140
SAL_FA7090AA	S06824-06	traces-0eQVNfK			Europe	United Kingdom	Enteritidis (Predicted)	Enteritidis	1425	40139
SAL_FA7093AA	S15-002261	traces-0pQoZaF			Europe	United Kingdom	Enteritidis (Predicted)	Enteritidis	1425	503
SAL_FA7094AA	S15-002694	traces-0GNILXJ			Europe	United Kingdom	Enteritidis (Predicted)	Enteritidis	1425	40138
SAL_FA7095AA	S15-1150	traces-0ONXCsH			Europe	United Kingdom	Enteritidis (Predicted)	Enteritidis	1425	40137
SAL_FA7096AA	S15-001578	traces-0eEnzzS			Europe	United Kingdom	Enteritidis (Predicted)	Enteritidis	1425	40136
SAL_FA7097AA	S15-001577	traces-0SeREfn			Europe	United Kingdom	Enteritidis (Predicted)	Enteritidis	1425	40112
SAL_FA7098AA	S15-1223	traces-0Eoyxjh			Europe	United Kingdom	Enteritidis (Predicted)	Enteritidis	-7006	40135
SAL_FA7099AA	S15-1154	traces-0LDpTAm			Europe	United Kingdom	Enteritidis (Predicted)	Enteritidis	1425	40132
SAL_FA7102AA	S15-1664	traces-0NhGSMS			Europe	United Kingdom	Enteritidis (Predicted)	Enteritidis	1425	40133
SAL_FA7107AA	L00853-15	traces-0SEgMZU			Europe	United Kingdom	Enteritidis (Predicted)	Enteritidis	1425	40162
SAL_FA7108AA	L00855-15	traces-0EmYNaw			Europe	United Kingdom	Enteritidis (Predicted)	Enteritidis	1425	40148
SAL_FA7109AA	S02859-15	traces-0OPPuvE			Europe	United Kingdom	Enteritidis (Predicted)	Enteritidis	1425	503
SAL_FA7110AA	S02861-15	traces-0BofoLc			Europe	United Kingdom	Enteritidis (Predicted)	Enteritidis	1425	40147
SAL_FA7111AA	S03-005205	traces-0Jcwduz			Europe	United Kingdom	Enteritidis (Predicted)	Enteritidis	1425	40146
SAL_FA7112AA	S15-003806	traces-0Ecpsuu			Europe	United Kingdom	Enteritidis (Predicted)	Enteritidis	1425	503
SAL_FA7128AA	I2016007212	SRR3571338	2016	5	North America	United States	Enteritidis	Enteritidis	1425	40916
SAL_FA7129AA	I2016007211	SRR3571313	2016	5	North America	United States	Enteritidis	Enteritidis	1425	40916
SAL_FA7130AA	I2016007000	SRR3571312	2016	5	North America	United States	Enteritidis	Enteritidis	1425	40916
SAL_FA7131AA	I2016006999	SRR3571311	2016	5	North America	United States	Enteritidis	Enteritidis	1425	40916
SAL_FA7138AA	E2016007253	SRR3571302	2016	5	North America	United States	Enteritidis	Enteritidis	34197	41685
SAL_FA7139AA	E2016006696	SRR3571300	2016	4	North America	United States	Enteritidis	Enteritidis	1425	1171
SAL_FA7140AA	I2016006732	SRR3571281	2016	5	North America	United States	Enteritidis	Enteritidis	1425	29454
SAL_FA7141AA	E2016007250	SRR3571280	2016	5	North America	United States	Enteritidis	Enteritidis	1425	41684

SAL_FA7142AA	PNUSAS002043	SRR3572573				North America	United States	Enteritidis (Predicted)	Enteritidis	27863	40225
SAL_FA7143AA	PNUSAS002199	SRR3572571	2016	3		North America	United States	Enteritidis	Enteritidis	1425	40224
SAL_FA7144AA	PNUSAS002108	SRR3572570	2016	3		North America	United States	Enteritidis	Enteritidis	1425	40226
SAL_FA7146AA	PNUSAS002197	SRR3572527	2016	2		North America	United States	Enteritidis	Enteritidis	1425	617
SAL_FA7147AA	PNUSAS002102	SRR3572506	2016	1		North America	United States	Enteritidis	Enteritidis	37741	41683
SAL_FA7149AA	PNUSAS002195	SRR3572456	2016	1		North America	United States	Enteritidis	Enteritidis	1425	6087
SAL_FA7161AA	PNUSAS002135	SRR3571906	2016	3		North America	United States	Enteritidis	Enteritidis	1425	180
SAL_FA7167AA	PNUSAS002208	SRR3571791				North America	United States	Enteritidis (Predicted)	Enteritidis	34197	41671
SAL_FA7168AA	PNUSAS002207	SRR3571790				North America	United States	Enteritidis (Predicted)	Enteritidis	1425	41670
SAL_FA7180AA	PNUSAS002252	SRR3573841	2016	4		North America	United States	Enteritidis	Enteritidis	1425	40210
SAL_FA7181AA	PNUSAS002251	SRR3573820	2016	4		North America	United States	Enteritidis	Enteritidis	1425	41660
SAL_FA7182AA	PNUSAS002250	SRR3573799	2016	4		North America	United States	Enteritidis	Enteritidis	1425	40209
SAL_FA7183AA	PNUSAS002249	SRR3573778	2016	4		North America	United States	Enteritidis	Enteritidis	1425	40208
SAL_FA7184AA	PNUSAS002247	SRR3573700	2016	4		North America	United States	Enteritidis	Enteritidis	1425	41658
SAL_FA7185AA	PNUSAS002246	SRR3573696				North America	United States	Enteritidis (Predicted)	Enteritidis	1425	41659
SAL_FA7186AA	PNUSAS002194	SRR3573618	2016	4		North America	United States	Enteritidis	Enteritidis	1425	447
SAL_FA7187AA	PNUSAS002245	SRR3573617				North America	United States	Enteritidis (Predicted)	Enteritidis	1425	41658
SAL_FA7188AA	PNUSAS002244	SRR3573595				North America	United States	Enteritidis (Predicted)	Enteritidis	1425	40177
SAL_FA7189AA	PNUSAS002243	SRR3573518				North America	United States	Enteritidis (Predicted)	Enteritidis	1425	36675
SAL_FA7227AA	PNUSAS002107	SRR3572568	2016	3		North America	United States	Enteritidis	Enteritidis	1425	40224
SAL_FA7300AA	MS150314	traces-0bQoJsY	2015			Europe	Ireland	Enteritidis	Enteritidis	1425	40252
SAL_FA7301AA	MS150319	traces-0rxwIPL	2015			Europe	Ireland	Enteritidis	Enteritidis	1425	40251
SAL_FA7303AA	MS150321	traces-0OCzqNk	2015			Europe	Ireland	Enteritidis	Enteritidis	1425	25188
SAL_FA7312AA	110976	SRR3585376	2015	5	11	Europe	United Kingdom	Enteritidis	Enteritidis	1425	2345
SAL_FA7313AA	168519	SRR3585375	2015	9	25	Europe	United Kingdom	Enteritidis	Enteritidis	1425	40270
SAL_FA7314AA	185894	SRR3585367	2015	11	11	Europe	United Kingdom	Enteritidis	Enteritidis	38719	40269
SAL_FA7315AA	190569	SRR3585365	2015	11	24	Europe	United Kingdom	Enteritidis	Enteritidis	38773	40268
SAL_FA7316AA	183605	SRR3585364	2015	11	2	Europe	United Kingdom	Enteritidis	Enteritidis	1425	40267
SAL_FA7317AA	178732	SRR3585363	2015	10	13	Europe	United Kingdom	Enteritidis	Enteritidis	1425	40266
SAL_FA7319AA	160806	SRR3585360	2015	9	10	Europe	United Kingdom	Enteritidis	Enteritidis	1425	40264
SAL_FA7320AA	160846	SRR3585359	2015	9	14	Europe	United Kingdom	Enteritidis	Enteritidis	1425	40263
SAL_FA7321AA	153189	SRR3585358	2015	8	27	Europe	United Kingdom	Enteritidis	Enteritidis	1425	40262
SAL_FA7322AA	120578	SRR3585357	2015	6	2	Europe	United Kingdom	Enteritidis	Enteritidis	1425	40261
SAL_FA7323AA	154869	SRR3585356	2015	9	1	Europe	United Kingdom	Enteritidis	Enteritidis	1425	40260
SAL_FA7324AA	179844	SRR3585355	2015	10	23	Europe	United Kingdom	Enteritidis	Enteritidis	26775	40259
SAL_FA7325AA	137147	SRR3585354	2015	7	23	Europe	United Kingdom	Enteritidis	Enteritidis	1425	40258
SAL_FA7326AA	134034	SRR3585352	2015	7	8	Europe	United Kingdom	Enteritidis	Enteritidis	1425	40257
SAL_FA7327AA	164715	SRR3585351	2015	9	21	Europe	United Kingdom	Enteritidis	Enteritidis	1425	40256
SAL_FA7329AA	152413	SRR3585348	2015	8	25	Europe	United Kingdom	Enteritidis	Enteritidis	37741	40254
SAL_FA7330AA	143553	SRR3585347	2015	8	10	Europe	United Kingdom	Enteritidis	Enteritidis	1425	40253
SAL_FA7342AA	PNUSAS000838	SRR3593758	2015	8		North America	United States	Poona	Enteritidis	1425	40306
SAL_FA7358AA	PNUSAS002289	SRR3593742				North America	United States	Enteritidis (Predicted)	Enteritidis	1425	40291
SAL_FA7359AA	PNUSAS002272	SRR3593741	2016	4		North America	United States	Enteritidis	Enteritidis	1425	40290
SAL_FA7360AA	PNUSAS002271	SRR3593680	2016	4		North America	United States	Enteritidis	Enteritidis	1425	40289
SAL_FA7431AA	CFSAN050983	SRR3606935	2016	5		North America	United States	Enteritidis	Enteritidis	1425	40426
SAL_FA7432AA	CFSAN051204	SRR3606934	2016	5		North America	United States	Enteritidis	Enteritidis	1425	40425
SAL_FA7433AA	CFSAN051203	SRR3606933	2016	5		North America	United States	Enteritidis	Enteritidis	1425	40425
SAL_FA7443AA	CFSAN032618	SRR3606913	2014	5		North America	United States	Enteritidis (Predicted)	Enteritidis	1425	40416
SAL_FA7451AA	CFSAN032622	SRR3606816	2014	5		North America	United States	Enteritidis (Predicted)	Enteritidis	1425	40410
SAL_FA7452AA	CFSAN032640	SRR3606815	2014	4		North America	United States	Enteritidis (Predicted)	Enteritidis	1425	40405
SAL_FA7453AA	CFSAN032623	SRR3606814	2014	5		North America	United States	Enteritidis (Predicted)	Enteritidis	1425	40408
SAL_FA7454AA	CFSAN032641	SRR3606813	2014	4		North America	United States	Enteritidis (Predicted)	Enteritidis	1425	40405
SAL_FA7456AA	CFSAN032642	SRR3606811	2014	4		North America	United States	Enteritidis (Predicted)	Enteritidis	1425	40404
SAL_FA7457AA	CFSAN032625	SRR3606810	2014	5		North America	United States	Enteritidis (Predicted)	Enteritidis	1425	8869
SAL_FA7469AA	CFSAN032583	SRR3606753	2014	5		North America	United States	Enteritidis (Predicted)	Enteritidis	1425	40393
SAL_FA7470AA	CFSAN032584	SRR3606752	2014	5		North America	United States	Enteritidis (Predicted)	Enteritidis	1425	40392
SAL_FA7473AA	CFSAN032587	SRR3606749	2014	5		North America	United States	Enteritidis (Predicted)	Enteritidis	1425	40389
SAL_FA7474AA	CFSAN032588	SRR3606748	2014	5		North America	United States	Enteritidis (Predicted)	Enteritidis	1425	40388
SAL_FA7479AA	CFSAN032593	SRR3606743	2014	5		North America	United States	Enteritidis (Predicted)	Enteritidis	1425	40383

SAL_FA7481AA	CFSAN032595	SRR3606740	2014	5	North America	United States	Enteritidis (Predicted)	Enteritidis	1425	40381
SAL_FA7483AA	CFSAN032597	SRR3606738	2014	5	North America	United States	Enteritidis (Predicted)	Enteritidis	1425	40379
SAL_FA7488AA	CFSAN032602	SRR3606732	2014	5	North America	United States	Enteritidis (Predicted)	Enteritidis	27901	40375
SAL_FA7489AA	CFSAN032606	SRR3606731	2014	5	North America	United States	Enteritidis (Predicted)	Enteritidis	1425	40373
SAL_FA7498AA	CFSAN035328	SRR3606722	2015	6	North America	United States	Enteritidis (Predicted)	Enteritidis	1425	40364
SAL_FA7509AA	CVM-N28729	SRR3606699	2010		North America	United States	Enteritidis	Enteritidis	1425	40356
SAL_FA7589AA	FSIS1606696	SRR3618595	2016		North America	United States	Enteritidis	Enteritidis	1425	41619
SAL_FA7611AA	E2016007564	SRR3634484	2016	5	North America	United States	Enteritidis	Enteritidis	1425	40487
SAL_FA7612AA	E2016007407	SRR3622483	2016	4	North America	United States	Enteritidis	Enteritidis	37741	40486
SAL_FA7641AA	CFSAN031222	SRR3622957	2014	5	North America	United States	Enteritidis (Predicted)	Enteritidis	1425	40520
SAL_FA7651AA	CFSAN031232	SRR3622941	2014	5	North America	United States	Enteritidis (Predicted)	Enteritidis	38857	40510
SAL_FA7652AA	CFSAN031233	SRR3622940	2014	5	North America	United States	Enteritidis (Predicted)	Enteritidis	1425	40509
SAL_FA7654AA	CFSAN031235	SRR3622938	2014	5	North America	United States	Enteritidis (Predicted)	Enteritidis	1425	40507
SAL_FA7664AA	CFSAN031245	SRR3622928	2014	5	North America	United States	Enteritidis (Predicted)	Enteritidis	1425	40498
SAL_FA7699AA	PNUSAS002346	SRR3632932	2016	4	North America	United States	Enteritidis	Enteritidis	1425	40563
SAL_FA7705AA	I2016007658	SRR3634510	2016	5	North America	United States	Enteritidis	Enteritidis	1425	40641
SAL_FA7706AA	I2016007594	SRR3634509	2016	5	North America	United States	Enteritidis	Enteritidis	26493	40640
SAL_FA7707AA	I2016007589	SRR3634508	2016	5	North America	United States	Enteritidis	Enteritidis	1425	40639
SAL_FA7708AA	E2016008135	SRR3634506	2016	5	North America	United States	Enteritidis	Enteritidis	1425	40592
SAL_FA7709AA	E2016007926	SRR3634505	2016	5	North America	United States	Enteritidis	Enteritidis	1425	40591
SAL_FA7710AA	ADRDL_ISUVDL19	SRR3634504	2010		North America	United States	Enteritidis (Predicted)	Enteritidis	1425	40590
SAL_FA7711AA	ADRDL_ISUVDL18	SRR3634503	2010		North America	United States	Enteritidis (Predicted)	Enteritidis	8116	40638
SAL_FA7712AA	ADRDL2011011290	SRR3634502	2011	4	North America	United States	Enteritidis (Predicted)	Enteritidis	1425	40637
SAL_FA7713AA	ADRDL2010037089	SRR3634501	2010	12	North America	United States	Enteritidis (Predicted)	Enteritidis	1425	40586
SAL_FA7714AA	ADRDL2010034399	SRR3634499	2010	11	North America	United States	Enteritidis (Predicted)	Enteritidis	1425	40585
SAL_FA7715AA	ADRDL2010034164	SRR3634498	2010	11	North America	United States	Enteritidis (Predicted)	Enteritidis	1425	40584
SAL_FA7716AA	ADRDL2010034161	SRR3634497	2010	11	North America	United States	Enteritidis (Predicted)	Enteritidis	1425	40583
SAL_FA7717AA	ADRDL2010034159	SRR3634496	2010	11	North America	United States	Enteritidis (Predicted)	Enteritidis	1425	40582
SAL_FA7718AA	ADRDL2010032613	SRR3634494	2010	11	North America	United States	Enteritidis (Predicted)	Enteritidis	1425	40581
SAL_FA7719AA	ADRDL2010031844	SRR3634493	2010	11	North America	United States	Enteritidis (Predicted)	Enteritidis	1425	40636
SAL_FA7720AA	ADRDL2010013903	SRR3634492	2010	5	North America	United States	Enteritidis (Predicted)	Enteritidis	1425	40635
SAL_FA7721AA	ADRDL2010013410	SRR3634491	2010	5	North America	United States	Enteritidis (Predicted)	Enteritidis	1425	40634
SAL_FA7722AA	ADRDL2010013409	SRR3634489	2010	5	North America	United States	Enteritidis (Predicted)	Enteritidis	1425	12129
SAL_FA7723AA	ADRDL2010013408	SRR3634488	2010	5	North America	United States	Enteritidis (Predicted)	Enteritidis	1425	12116
SAL_FA7724AA	ADRDL2010011928	SRR3634487	2010	4	North America	United States	Enteritidis (Predicted)	Enteritidis	1425	40580
SAL_FA7725AA	ADRDL2010011927	SRR3634486	2010	4	North America	United States	Enteritidis (Predicted)	Enteritidis	1425	40579
SAL_FA7752AA	PNUSAS002414	SRR3642947	2016	4	North America	United States	Enteritidis	Enteritidis	1425	40608
SAL_FA7753AA	PNUSAS002413	SRR3642946	2016	4	North America	United States	Enteritidis	Enteritidis	1425	40609
SAL_FA7759AA	PNUSAS002351	SRR3642938	2016	2	North America	United States	Enteritidis	Enteritidis	1425	40603
SAL_FA7763AA	FDA00003652	SRR3641795	2012	5	North America	United States	Enteritidis	Enteritidis	1425	40600
SAL_FA7764AA	FDA00003653	SRR3641794	2012	5	North America	United States	Enteritidis	Enteritidis	1425	40623
SAL_FA7765AA	FDA00003654	SRR3641793	2012	5	North America	United States	Enteritidis	Enteritidis	1425	40622
SAL_FA7766AA	FDA00003655	SRR3641792	2012	5	North America	United States	Enteritidis	Enteritidis	1425	40599
SAL_FA7767AA	FDA00003656	SRR3641791	2012	5	North America	United States	Enteritidis	Enteritidis	1425	40598
SAL_FA7768AA	FDA00003657	SRR3641790	2012	5	North America	United States	Enteritidis	Enteritidis	1425	40597
SAL_FA7769AA	FDA00003658	SRR3641789	2012	5	North America	United States	Enteritidis	Enteritidis	1425	40593
SAL_FA7770AA	FDA00003659	SRR3641788	2012	5	North America	United States	Enteritidis	Enteritidis	1425	40596
SAL_FA7771AA	FDA00003660	SRR3641778	2012	5	North America	United States	Enteritidis	Enteritidis	1425	40595
SAL_FA7772AA	FDA00003661	SRR3641777	2012	5	North America	United States	Enteritidis	Enteritidis	1425	40594
SAL_FA7773AA	FDA00003662	SRR3641776	2012	5	North America	United States	Enteritidis	Enteritidis	1425	40593
SAL_FA7774AA	FDA00003663	SRR3641775	2012	5	North America	United States	Enteritidis	Enteritidis	1425	40588
SAL_FA7781AA	FDA00002085	SRR3647313	2004	5	North America	United States	Enteritidis	Enteritidis	1425	40657
SAL_FA7782AA	FDA00002082	SRR3647312	2004	5	North America	United States	Enteritidis	Enteritidis	1425	40656
SAL_FA7783AA	FDA00000050	SRR3647311	2001	4	North America	United States	Enteritidis	Enteritidis	1425	40657
SAL_FA7794AA	ADRDL_ISUVDL21	SRR3649689	2010		North America	United States	Enteritidis (Predicted)	Enteritidis	8116	40679
SAL_FA7795AA	ADRDL_ISUVDL20	SRR3649688	2010		North America	United States	Enteritidis (Predicted)	Enteritidis	1425	40678
SAL_FA7818AA	ADRDL2010015944	SRR3649720	2010	6	North America	United States	Enteritidis (Predicted)	Enteritidis	1425	40695
SAL_FA7819AA	ADRDL2010015727	SRR3649719	2010	5	North America	United States	Enteritidis (Predicted)	Enteritidis	1425	40694
SAL_FA7820AA	ADRDL2010014849	SRR3649718	2010	5	North America	United States	Enteritidis (Predicted)	Enteritidis	1425	40693

SAL_FA7821AA	ADRDL2010014806	SRR3649703	2010	5	North America	United States	Enteritidis (Predicted)	Enteritidis	1425	40692
SAL_FA7822AA	ADRDL2010014848	SRR3649702	2010	5	North America	United States	Enteritidis (Predicted)	Enteritidis	1425	40691
SAL_FA7823AA	ADRDL2010014360	SRR3649701	2010	5	North America	United States	Enteritidis (Predicted)	Enteritidis	1425	40690
SAL_FA7824AA	ADRDL2010014357	SRR3649700	2010	5	North America	United States	Enteritidis (Predicted)	Enteritidis	-6845	40689
SAL_FA7825AA	ADRDL2010017460	SRR3649699	2010	6	North America	United States	Enteritidis (Predicted)	Enteritidis	1425	40688
SAL_FA7826AA	ADRDL2010016499	SRR3649698	2010	6	North America	United States	Enteritidis (Predicted)	Enteritidis	1425	40684
SAL_FA7827AA	ADRDL2012001037	SRR3649697	2012	1	North America	United States	Enteritidis (Predicted)	Enteritidis	1425	40683
SAL_FA7829AA	ADRDL2012028707	SRR3649693	2012	8	North America	United States	Enteritidis (Predicted)	Enteritidis	-6844	40681
SAL_FA7830AA	ADRDL2011030426	SRR3649692	2011	9	North America	United States	Enteritidis (Predicted)	Enteritidis	1425	40680
SAL_FA7859AA	I2016008288-1	SRR3743173	2016	6	North America	United States	Enteritidis	Enteritidis	1425	40732
SAL_FA7860AA	I2016008251-1	SRR3743174	2016	5	North America	United States	Enteritidis	Enteritidis	1425	40728
SAL_FA7861AA	I2016008250-2	SRR3696038	2016	5	North America	United States	Enteritidis	Enteritidis	1425	40731
SAL_FA7862AA	I2016007916	SRR3696037	2016	5	North America	United States	Enteritidis	Enteritidis	1425	40730
SAL_FA7863AA	E2016008572	SRR3696036	2016	6	North America	United States	Enteritidis	Enteritidis	1425	40729
SAL_FA7864AA	E2016008452	SRR3674946	2016	5	North America	United States	Enteritidis	Enteritidis	1425	40728
SAL_FA7865AA	E2016008445	SRR3660530	2016	6	North America	United States	Enteritidis	Enteritidis	1425	40727
SAL_FA7866AA	E2016008278	SRR3660529	2016	5	North America	United States	Enteritidis	Enteritidis	1425	40726
SAL_FA7867AA	E2016008277	SRR3660528	2016	5	North America	United States	Enteritidis	Enteritidis	1425	40725
SAL_FA7868AA	E2016008276	SRR3660527	2016	5	North America	United States	Enteritidis	Enteritidis	1425	40724
SAL_FA7869AA	E2016008275	SRR3660151	2016	5	North America	United States	Enteritidis	Enteritidis	1425	40724
SAL_FA7870AA	E2016008274	SRR3660150	2016	5	North America	United States	Enteritidis	Enteritidis	1425	40724
SAL_FA7871AA	E2016008273	SRR3660149	2016	5	North America	United States	Enteritidis	Enteritidis	1425	40724
SAL_FA7872AA	E2016008211	SRR3660132	2016	5	North America	United States	Enteritidis	Enteritidis	37741	40723
SAL_FA7873AA	E2016008173	SRR3696018	2016	5	North America	United States	Enteritidis	Enteritidis	1425	40722
SAL_FA7874AA	E2016008169-1	SRR3660130	2016	5	North America	United States	Enteritidis	Enteritidis	1425	40721
SAL_FA7876AA	FDA00003644	SRR3659834	2012	5	North America	United States	Enteritidis	Enteritidis	1425	40593
SAL_FA7877AA	FDA00003646	SRR3659833	2012	5	North America	United States	Enteritidis	Enteritidis	-6847	40719
SAL_FA7878AA	FDA00003647	SRR3659832	2012	5	North America	United States	Enteritidis	Enteritidis	1425	40718
SAL_FA7879AA	FDA00003648	SRR3659474	2012	5	North America	United States	Enteritidis	Enteritidis	1425	40717
SAL_FA7880AA	FDA00003649	SRR3659473	2012	5	North America	United States	Enteritidis	Enteritidis	1425	40593
SAL_FA7881AA	FDA00003650	SRR3659472	2012	5	North America	United States	Enteritidis	Enteritidis	1425	40716
SAL_FA7882AA	FDA00003651	SRR3659471	2012	5	North America	United States	Enteritidis	Enteritidis	1425	40715
SAL_FA7884AA	FSIS1606784	SRR3667097	2016		North America	United States	Enteritidis	Enteritidis	1425	40764
SAL_FA7920AA	15MN00053	SRR3662623	2015	1	North America	United States	Enteritidis	Enteritidis	1425	40743
SAL_FA7927AA	PNUSAS002388	SRR3667941	2016	5	North America	United States	Enteritidis	Enteritidis	1425	40808
SAL_FA7929AA	PNUSAS002386	SRR3667939			North America	United States	Enteritidis (Predicted)	Enteritidis	1425	40808
SAL_FA7930AA	PNUSAS002385	SRR3667938			North America	United States	Enteritidis (Predicted)	Enteritidis	1425	40808
SAL_FA7931AA	PNUSAS002376	SRR3667937	2016	5	North America	United States	Enteritidis	Enteritidis	1425	40609
SAL_FA7944AA	PNUSAS002472	SRR3667924	2016	4	North America	United States	Enteritidis	Enteritidis	1425	40802
SAL_FA7945AA	PNUSAS002471	SRR3667923	2016	4	North America	United States	Enteritidis	Enteritidis	1425	40801
SAL_FA7946AA	PNUSAS002470	SRR3667901	2016	3	North America	United States	Enteritidis	Enteritidis	1425	180
SAL_FA7947AA	PNUSAS002469	SRR3667900	2016	3	North America	United States	Enteritidis	Enteritidis	1425	40800
SAL_FA7949AA	PNUSAS002452	SRR3667858	2016	5	North America	United States	Enteritidis	Enteritidis	1425	40798
SAL_FA7955AA	PNUSAS002446	SRR3667851	2016	4	North America	United States	Enteritidis	Enteritidis	1425	180
SAL_FA7974AA	2016K-0430	SRR3667831			North America	United States	Enteritidis (Predicted)	Enteritidis	1425	40787
SAL_FA7976AA	2016K-0436	SRR3667829			North America	United States	Enteritidis (Predicted)	Enteritidis	27863	40785
SAL_FA7979AA	2016K-0440	SRR3667826			North America	United States	Enteritidis (Predicted)	Enteritidis	27863	40225
SAL_FA7980AA	2016K-0431	SRR3667825			North America	United States	Enteritidis (Predicted)	Enteritidis	1425	40784
SAL_FA7982AA	2016K-0435	SRR3667823			North America	United States	Enteritidis (Predicted)	Enteritidis	1425	40782
SAL_FA7983AA	2016K-0429	SRR3667822			North America	United States	Enteritidis (Predicted)	Enteritidis	27863	40783
SAL_FA7984AA	2016K-0432	SRR3667821			North America	United States	Enteritidis (Predicted)	Enteritidis	1425	39779
SAL_FA7988AA	2016K-0427	SRR3667817			North America	United States	Enteritidis (Predicted)	Enteritidis	1425	180
SAL_FA7990AA	2016K-0422	SRR3667815			North America	United States	Enteritidis (Predicted)	Enteritidis	1425	180
SAL_FA7991AA	2016K-0423	SRR3667814			North America	United States	Enteritidis (Predicted)	Enteritidis	37133	177
SAL_FA8012AA	2013AM-0984	SRR3676331			North America	United States	Enteritidis (Predicted)	Enteritidis	28412	40874
SAL_FA8024AA	E2016008184	SRR3696033	2016	5	North America	United States	Enteritidis	Enteritidis	1425	40865
SAL_FA8027AA	ADRDL2011019869	SRR3674699	2011	6	North America	United States	Enteritidis (Predicted)	Enteritidis	1425	40862
SAL_FA8028AA	ADRDL2011012612	SRR3674685	2011	4	North America	United States	Enteritidis (Predicted)	Enteritidis	1425	40868
SAL_FA8029AA	ADRDL2011010185	SRR3674684	2011	3	North America	United States	Enteritidis (Predicted)	Enteritidis	1425	40861

SAL_FA8030AA	ADRDL2010029485	SRR3674673	2010	10	North America	United States	Enteritidis (Predicted)	Enteritidis	1425	40860
SAL_FA8031AA	ADRDL2010025661	SRR3674672	2010	9	North America	United States	Enteritidis (Predicted)	Enteritidis	1425	40859
SAL_FA8032AA	ADRDL2010023741	SRR3674666	2010	8	North America	United States	Enteritidis (Predicted)	Enteritidis	1425	40858
SAL_FA8033AA	ADRDL2010023513	SRR3674665	2010	8	North America	United States	Enteritidis (Predicted)	Enteritidis	1425	40857
SAL_FA8034AA	ADRDL2010023423	SRR3674664	2010	8	North America	United States	Enteritidis (Predicted)	Enteritidis	1425	40856
SAL_FA8035AA	ADRDL2010023411	SRR3674663	2010	8	North America	United States	Enteritidis (Predicted)	Enteritidis	1425	12124
SAL_FA8036AA	ADRDL2010023303	SRR3674662	2010	8	North America	United States	Enteritidis (Predicted)	Enteritidis	1425	40855
SAL_FA8053AA	PNUSAS002445	SRR3670104			North America	United States	Enteritidis (Predicted)	Enteritidis	1425	40842
SAL_FA8059AA	PNUSAS002486	SRR3669929	2016	5	North America	United States	Enteritidis	Enteritidis	1425	40837
SAL_FA8060AA	PNUSAS002485	SRR3669928	2016	5	North America	United States	Enteritidis	Enteritidis	1425	40837
SAL_FA8061AA	PNUSAS002484	SRR3669925	2016	5	North America	United States	Enteritidis	Enteritidis	1425	40836
SAL_FA8062AA	PNUSAS002248	SRR3669924	2016	4	North America	United States	Enteritidis	Enteritidis	1425	40835
SAL_FA8113AA	2016K-0494	SRR3708005			North America	United States	Enteritidis (Predicted)	Enteritidis	27863	40917
SAL_FA8114AA	2016K-0470	SRR3708004			North America	United States	Enteritidis (Predicted)	Enteritidis	27863	40225
SAL_FA8119AA	2016K-0467	SRR3707999			North America	United States	Enteritidis (Predicted)	Enteritidis	37133	177
SAL_FA8122AA	CFSAN048079	SRR3707431	2015	9	North America	United States	Enteritidis (Predicted)	Enteritidis	1425	40912
SAL_FA8127AA	I2016008677	SRR3707421	2016	6	North America	United States	Enteritidis	Enteritidis	1425	40905
SAL_FA8128AA	E2016008726	SRR3707420	2016	6	North America	United States	Enteritidis	Enteritidis	1425	40909
SAL_FA8129AA	E2016008707	SRR3707419	2016	6	North America	United States	Enteritidis	Enteritidis	1425	40904
SAL_FA8130AA	I2016008661	SRR3707418	2016	6	North America	United States	Enteritidis	Enteritidis	1425	40903
SAL_FA8131AA	E2016008453	SRR3707417	2016	6	North America	United States	Enteritidis	Enteritidis	1425	40916
SAL_FA8139AA	FSIS1606888	SRR3710249	2016		North America	United States	Enteritidis	Enteritidis	1425	40922
SAL_FA8144AA	FSIS1606877	SRR3710223	2016		North America	United States	Enteritidis	Enteritidis	1425	36028
SAL_FA8178AA	PNUSAS002542	SRR3713627	2016	5	North America	United States	Enteritidis	Enteritidis	1425	6087
SAL_FA8206AA	FDA00003941	SRR3721547	2011	9	Asia	China	Enteritidis	Enteritidis	1425	41510
SAL_FA8212AA	CFSAN051893	SRR3721487	2016	6	North America	United States	Enteritidis	Enteritidis	1425	41535
SAL_FA8213AA	CFSAN051896	SRR3721486	2016	6	North America	United States	Enteritidis (Predicted)	Enteritidis	1425	41534
SAL_FA8216AA	E2016009047	SRR3721482	2016	6	North America	United States	Enteritidis	Enteritidis	1425	41531
SAL_FA8221AA	ARIM-SE1258-05	SRR3721476	2005	5	South America	Argentina	Enteritidis	Enteritidis	1425	41526
SAL_FA8381AA	PNUSAS002541	SRR3713626	2016	5	North America	United States	Enteritidis	Enteritidis	1425	6087
SAL_FA8389AA	PNUSAS002488	SRR3713617			North America	United States	Enteritidis (Predicted)	Enteritidis	27863	47457
SAL_FA8393AA	PNUSAS002504	SRR3713613	2016	5	North America	United States	Enteritidis	Enteritidis	1425	47454
SAL_FA8394AA	PNUSAS002503	SRR3713612	2016	5	North America	United States	Enteritidis	Enteritidis	1425	47448
SAL_FA8395AA	PNUSAS002502	SRR3713611	2016	5	North America	United States	Enteritidis	Enteritidis	1425	47453
SAL_FA8396AA	PNUSAS002501	SRR3713610	2016	5	North America	United States	Enteritidis	Enteritidis	1425	47452
SAL_FA8397AA	PNUSAS002483	SRR3713608	2016	5	North America	United States	Enteritidis	Enteritidis	1425	180
SAL_FA8398AA	PNUSAS002482	SRR3713607	2016	5	North America	United States	Enteritidis	Enteritidis	1425	180
SAL_FA8399AA	PNUSAS002481	SRR3713606			North America	United States	Enteritidis (Predicted)	Enteritidis	37133	47451
SAL_FA8400AA	PNUSAS002480	SRR3713605	2016	5	North America	United States	Enteritidis	Enteritidis	1425	47450
SAL_FA8401AA	PNUSAS002479	SRR3713604	2016	5	North America	United States	Enteritidis	Enteritidis	1425	47449
SAL_FA8402AA	PNUSAS002500	SRR3713603	2016	5	North America	United States	Enteritidis	Enteritidis	1425	47448
SAL_FA8405AA	PNUSAS002522	SRR3713600	2016	5	North America	United States	Enteritidis	Enteritidis	1425	1026
SAL_FA8411AA	PNUSAS002496	SRR3713594	2016	4	North America	United States	Enteritidis	Enteritidis	1425	47440
SAL_FA8425AA	PNUSAS002521	SRR3713567	2016	5	North America	United States	Enteritidis	Enteritidis	1425	47428
SAL_FA8459AA	FSIS1606833	SRR3710738	2016		North America	United States	Enteritidis	Enteritidis	1425	41754
SAL_FA8474AA	PNUSAS002222	SRR3732324	2016	4	North America	United States	Enteritidis	Enteritidis	1425	47396
SAL_FA8475AA	PNUSAS002221	SRR3680154	2016	4	North America	United States	Enteritidis	Enteritidis	1425	180
SAL_FA8483AA	PNUSAS002378	SRR3732328	2016	5	North America	United States	Enteritidis	Enteritidis	1425	41758
SAL_FA8484AA	PNUSAS002377	SRR3732327	2016	5	North America	United States	Enteritidis	Enteritidis	1425	40609
SAL_FA8485AA	PNUSAS002375	SRR3680143			North America	United States	Enteritidis	Enteritidis	1425	47389
SAL_FA8486AA	PNUSAS002374	SRR3732314	2016	4	North America	United States	Enteritidis	Enteritidis	1425	47388
SAL_FA8503AA	CVM N38233	SRR3665310	2011		North America	United States	Enteritidis	Enteritidis	1425	47372
SAL_FA8513AA	CVM N37947	SRR3665300	2011		North America	United States	Enteritidis	Enteritidis	1425	47363
SAL_FA8524AA	CVM N37936	SRR3665289	2011		North America	United States	Enteritidis	Enteritidis	1425	47354
SAL_FA8525AA	CVM N37935	SRR3665288	2011		North America	United States	Typhimurium	Enteritidis	1425	47353
SAL_FA8536AA	CVM N37924	SRR3665277	2011		North America	United States	Enteritidis	Enteritidis	1425	47342
SAL_FA8541AA	CVM N37919	SRR3665272	2011		North America	United States	Kentucky	Enteritidis	1425	47337
SAL_FA8551AA	CVM N37909	SRR3665262	2011		North America	United States	Enteritidis	Enteritidis	42202	47329
SAL_FA8556AA	CVM N37904	SRR3665257	2011		North America	United States	Enteritidis	Enteritidis	1425	47324

SAL_FA8558AA	CVM N37902	SRR3665255	2011		North America	United States	Enteritidis	Enteritidis	1425	47322
SAL_FA8607AA	CVM N32752	SRR3665206	2011		North America	United States	Enteritidis	Enteritidis	1425	47274
SAL_FA8616AA	CVM N32062	SRR3665197	2011		North America	United States	Enteritidis	Enteritidis	1425	47265
SAL_FA8673AA	CVM N31405	SRR3665140	2011		North America	United States	Enteritidis	Enteritidis	1425	47211
SAL_FA8681AA	CVM N31397	SRR3665132	2011		North America	United States	Enteritidis	Enteritidis	1425	47203
SAL_FA8682AA	CVM N31396	SRR3665131	2011		North America	United States	Enteritidis	Enteritidis	1425	47202
SAL_FA8698AA	CVM N31380	SRR3665115	2011		North America	United States	Enteritidis	Enteritidis	1425	47546
SAL_FA8713AA	CVM N30691	SRR3665100	2011		North America	United States	Enteritidis	Enteritidis	1425	47187
SAL_FA8729AA	CVM N30675	SRR3665084	2011		North America	United States	Enteritidis	Enteritidis	1425	47172
SAL_FA8745AA	CVM N30659	SRR3665068	2011		North America	United States	Enteritidis	Enteritidis	1425	47157
SAL_FA8746AA	CVM N30658	SRR3665067	2011		North America	United States	Enteritidis	Enteritidis	1425	47156
SAL_FA8765AA	CVM N30639	SRR3665048	2011		North America	United States	Enteritidis	Enteritidis	1425	47137
SAL_FA8769AA	CVM N29385	SRR3665044	2011		North America	United States	Enteritidis	Enteritidis	1425	47133
SAL_FA8794AA	CVM N29358	SRR3665019	2011		North America	United States	Enteritidis	Enteritidis	1425	47111
SAL_FA8824AA	CVM N29328	SRR3664989	2011		North America	United States	Enteritidis	Enteritidis	1425	47082
SAL_FA8865AA	CVM N42517	SRR3664948	2012		North America	United States	Enteritidis	Enteritidis	1425	47002
SAL_FA8866AA	CVM N42516	SRR3664947	2012		North America	United States	Enteritidis	Enteritidis	1425	47045
SAL_FA8877AA	CVM N42504	SRR3664936	2012		North America	United States	Enteritidis	Enteritidis	1425	46993
SAL_FA8886AA	CVM N42495	SRR3664927	2012		North America	United States	Enteritidis	Enteritidis	1425	46986
SAL_FA8920AA	CVM N42461	SRR3664893	2012		North America	United States	Enteritidis	Enteritidis	1425	46953
SAL_FA8942AA	CVM N42329	SRR3664871	2012		North America	United States	Enteritidis	Enteritidis	1425	46930
SAL_FA8971AA	CVM N41912	SRR3664842	2012		North America	United States	Enteritidis	Enteritidis	1425	46904
SAL_FA9002AA	CVM N41742	SRR3664811	2012		North America	United States	Enteritidis	Enteritidis	1425	14207
SAL_FA9019AA	CVM N40937	SRR3664794	2012		North America	United States	Enteritidis	Enteritidis	1425	46854
SAL_FA9024AA	CVM N40931	SRR3664789	2012		North America	United States	Enteritidis	Enteritidis	1425	46850
SAL_FA9027AA	CVM N40928	SRR3664786	2012		North America	United States	Enteritidis	Enteritidis	1425	46858
SAL_FA9028AA	CVM N40927	SRR3664785	2012		North America	United States	Enteritidis	Enteritidis	1425	46847
SAL_FA9054AA	CVM N40402	SRR3664759	2012		North America	United States	Enteritidis	Enteritidis	1425	46825
SAL_FA9056AA	CVM N40400	SRR3664757	2012		North America	United States	Enteritidis	Enteritidis	1425	46824
SAL_FA9057AA	CVM N40399	SRR3664756	2012		North America	United States	Iiia 18:Z4,Z23:-	Enteritidis	1425	13122
SAL_FA9063AA	CVM N40393	SRR3664750	2012		North America	United States	Enteritidis	Enteritidis	1425	46819
SAL_FA9066AA	CVM N40390	SRR3664747	2012		North America	United States	Enteritidis	Enteritidis	1425	46816
SAL_FA9067AA	CVM N40389	SRR3664746	2012		North America	United States	Enteritidis	Enteritidis	1425	46814
SAL_FA9082AA	CVM N40372	SRR3664731	2012		North America	United States	I 9,12:Non-Motile	Enteritidis	1425	46455
SAL_FA9104AA	CVM N39853	SRR3664709	2012		North America	United States	Iiia 18:Z4,Z23:-	Enteritidis	1425	46430
SAL_FA9108AA	CVM N39849	SRR3664705	2012		North America	United States	Typhimurium Var. 5-	Enteritidis	1425	46427
SAL_FA9140AA	CVM N38916	SRR3664673	2012		North America	United States	Enteritidis	Enteritidis	1425	46378
SAL_FA9148AA	CVM N38908	SRR3664665	2012		North America	United States	Enteritidis	Enteritidis	1425	46370
SAL_FA9154AA	CVM N38902	SRR3664659	2012		North America	United States	Enteritidis	Enteritidis	1425	46326
SAL_FA9156AA	CVM N38895	SRR3664652	2012		North America	United States	Enteritidis	Enteritidis	1425	46324
SAL_FA9179AA	CVM N38872	SRR3664629	2012		North America	United States	Enteritidis	Enteritidis	1425	46203
SAL_FA9196AA	CVM N38854	SRR3664612	2012		North America	United States	Enteritidis	Enteritidis	1425	46187
SAL_FA9200AA	CVM N38850	SRR3664608	2012		North America	United States	Enteritidis	Enteritidis	1425	46184
SAL_FA9205AA	CVM N38901	SRR3664658	2012		North America	United States	Enteritidis	Enteritidis	1425	41766
SAL_FA9207AA	CVM N38899	SRR3664656	2012		North America	United States	Enteritidis	Enteritidis	1425	41764
SAL_FA9210AA	0000-0058-2895	SRR3618628	2016		North America	United States	Enteritidis (Predicted)	Enteritidis	1425	2668
SAL_FA9212AA	NY64344217	SRR3536615	2016	4	North America	United States	Enteritidis	Enteritidis	1425	41759
SAL_FA9213AA	NY64333822	SRR3536614	2016	4	North America	United States	Enteritidis	Enteritidis	1425	41758
SAL_FA9214AA	NY64333783	SRR3536613	2016	4	North America	United States	Enteritidis	Enteritidis	1425	41757
SAL_FA9215AA	NY64333741	SRR3536612	2016	4	North America	United States	Enteritidis	Enteritidis	1425	40609
SAL_FA9217AA	NY64248047	SRR3479907	2016	4	North America	United States	Enteritidis	Enteritidis	1425	41757
SAL_FA9218AA	NY64247955	SRR3479906	2016	4	North America	United States	Enteritidis	Enteritidis	1425	41757
SAL_FA9219AA	NY64247851	SRR3479905	2016	4	North America	United States	Enteritidis	Enteritidis	1425	180
SAL_FA9220AA	NY64247811	SRR3479904	2016	4	North America	United States	Enteritidis	Enteritidis	1425	46183
SAL_FA9221AA	NY64247773	SRR3479903	2016	4	North America	United States	Enteritidis	Enteritidis	1425	46182
SAL_FA9222AA	NY64247713	SRR3479902	2016	3	North America	United States	Enteritidis	Enteritidis	1425	41758
SAL_FA9223AA	NY64247673	SRR3479901	2016	4	North America	United States	Enteritidis	Enteritidis	1425	46181
SAL_FA9229AA	NY63966755	SRR3457618	2016	4	North America	United States	Enteritidis	Enteritidis	1425	46175
SAL_FA9230AA	NY63966716	SRR3457617	2016	4	North America	United States	Enteritidis	Enteritidis	1425	46366

SAL_FA9231AA	NY63966674	SRR3457616	2016	4	North America	United States	Enteritidis	Enteritidis	1425	41758
SAL_FA9232AA	NY63966636	SRR3457615	2016	3	North America	United States	Enteritidis	Enteritidis	1425	41758
SAL_FA9233AA	NY63966598	SRR3457614	2016	3	North America	United States	Enteritidis	Enteritidis	1425	4151
SAL_FA9234AA	NY63966560	SRR3457613	2016	3	North America	United States	Enteritidis	Enteritidis	1425	40289
SAL_FA9235AA	NY63966522	SRR3457612	2016	3	North America	United States	Enteritidis	Enteritidis	1425	46175
SAL_FA9236AA	NY63966472	SRR3457611	2016	4	North America	United States	Enteritidis	Enteritidis	1425	46174
SAL_FA9237AA	NY63966434	SRR3457610	2016	3	North America	United States	Enteritidis	Enteritidis	1425	41758
SAL_FA9241AA	2016K-0209	SRR3392790			North America	United States	Enteritidis (Predicted)	Enteritidis	1425	717
SAL_FA9279AA	CFSAN046997	SRR3225393	2015	9	North America	United States	Enteritidis (Predicted)	Enteritidis	1425	4174
SAL_FA9280AA	CFSAN046996	SRR3225392	2015	9	North America	United States	Enteritidis (Predicted)	Enteritidis	1425	46143
SAL_FA9306AA	AZ-TG92668	SRR3224203	2015	9	North America	United States	Enteritidis	Enteritidis	1425	4174
SAL_FA9355AA	E2016003571	SRR3223817	2016	3	North America	United States	Enteritidis	Enteritidis	1425	45918
SAL_FA9356AA	E2016003547	SRR3223816	2016	3	North America	United States	Enteritidis	Enteritidis	1425	617
SAL_FA9357AA	E2016003457	SRR3223815	2016	3	North America	United States	Enteritidis	Enteritidis	1425	613
SAL_FA9358AA	E2016003446	SRR3223814	2016	3	North America	United States	Enteritidis	Enteritidis	1425	45917
SAL_FA9359AA	E2016003440	SRR3223813	2016	3	North America	United States	Enteritidis	Enteritidis	1425	45916
SAL_FA9360AA	E2016003423	SRR3223812	2016	2	North America	United States	Enteritidis	Enteritidis	34197	45921
SAL_FA9361AA	E2016003239	SRR3223811	2016	2	North America	United States	Enteritidis	Enteritidis	1425	45915
SAL_FA9362AA	E2016003221	SRR3223810	2016	2	North America	United States	Enteritidis	Enteritidis	1425	617
SAL_FA9363AA	E2016003179	SRR3223809	2016	2	North America	United States	Enteritidis	Enteritidis	1425	45911
SAL_FA9400AA	AZ-TG74416	SRR3223149	2005		North America	United States	Enteritidis	Enteritidis	1425	45872
SAL_FA9428AA	AZ-TG92664	SRR3219250	2015	4	North America	United States	Enteritidis	Enteritidis	1425	4169
SAL_FA9429AA	AZ-TG92660	SRR3219249	2015	4	North America	United States	Enteritidis	Enteritidis	1425	4169
SAL_FA9449AA	CVM-N19727	SRR3219139	2008		North America	United States	Enteritidis	Enteritidis	1425	45809
SAL_FA9450AA	CVM-N18616	SRR3219137	2008		North America	United States	Enteritidis	Enteritidis	1425	45807
SAL_FA9451AA	AZ-TG92700	SRR3219135	2015	9	North America	United States	Enteritidis	Enteritidis	1425	45806
SAL_FA9452AA	AZ-TG92696	SRR3219133	2015	9	North America	United States	Enteritidis	Enteritidis	1425	45805
SAL_FA9453AA	AZ-TG92692	SRR3219127	2015	9	North America	United States	Enteritidis	Enteritidis	1425	4174
SAL_FA9454AA	AZ-TG92688	SRR3219125	2015	9	North America	United States	Enteritidis	Enteritidis	1425	45804
SAL_FA9455AA	AZ-TG92684	SRR3219124	2015	9	North America	United States	Enteritidis	Enteritidis	1425	4174
SAL_FA9456AA	AZ-TG92680	SRR3219123	2015	9	North America	United States	Enteritidis	Enteritidis	1425	4174
SAL_FA9457AA	AZ-TG92676	SRR3219121	2015	9	North America	United States	Enteritidis	Enteritidis	1425	4174
SAL_FA9458AA	AZ-TG92672	SRR3219120	2015	9	North America	United States	Enteritidis	Enteritidis	1425	46021
SAL_FA9478AA	I2016002671	SRR3217352	2016	2	North America	United States	Enteritidis	Enteritidis	1425	45779
SAL_FA9479AA	E2016003173	SRR3217351	2016	2	North America	United States	Enteritidis	Enteritidis	1425	45784
SAL_FA9480AA	E2016003171	SRR3217350	2016	2	North America	United States	Enteritidis	Enteritidis	42123	45783
SAL_FA9481AA	E2016002889	SRR3217349	2016	2	North America	United States	I 9,12:-:-	Enteritidis	42123	45782
SAL_FA9482AA	E2016002887	SRR3217348	2016	2	North America	United States	Enteritidis	Enteritidis	1425	45781
SAL_FA9483AA	E2016002765	SRR3217347	2016	2	North America	United States	Enteritidis	Enteritidis	1425	45780
SAL_FA9484AA	E2016002752	SRR3217346	2016	2	North America	United States	Enteritidis	Enteritidis	1425	45779
SAL_FA9485AA	E2016002728	SRR3217345	2016	2	North America	United States	Enteritidis	Enteritidis	1425	45778
SAL_FA9486AA	E2016002711	SRR3217344	2016	2	North America	United States	Enteritidis	Enteritidis	1425	40905
SAL_FA9512AA	E2015011861	SRR3135177	2015	8	North America	United States	Enteritidis	Enteritidis	1425	45534
SAL_FA9536AA	CDPHFDLB-F14M01191b	SRR3110376	2014	7	North America	United States	Enteritidis	Enteritidis	1425	45512
SAL_FA9537AA	CDPHFDLB-F14M01191a	SRR3110375	2014	7	North America	United States	Enteritidis	Enteritidis	1425	45511
SAL_FA9548AA	E2016000287	SRR3110337	2015	12	North America	United States	Enteritidis	Enteritidis	1425	45499
SAL_FA9549AA	E2016000456	SRR3110336	2015	12	North America	United States	Enteritidis	Enteritidis	1425	45498
SAL_FA9550AA	E2016000376	SRR3110335	2016	1	North America	United States	Enteritidis	Enteritidis	26775	45497
SAL_FA9614AA	CVM-N23792	SRR3098645	2010		North America	United States	Enteritidis	Enteritidis	1425	45460
SAL_FA9641AA	WAPHL-SAL-A00966	SRR3057230	2009		North America	United States	Enteritidis	Enteritidis	33238	45436
SAL_FA9644AA	WAPHL-SAL-A00963	SRR3057227	2009		North America	United States	Enteritidis	Enteritidis	1425	45434
SAL_GA0110AA	NY59589192	SRR2734630					Enteritidis (Predicted)	Enteritidis	1425	45084
SAL_GA0111AA	NY59589171	SRR2734629					Enteritidis (Predicted)	Enteritidis	1425	45089
SAL_GA0112AA	NY59550709	SRR2734585	2015	9	North America	United States	Enteritidis	Enteritidis	1425	45088
SAL_GA0113AA	NY59550630	SRR2734584	2015	8	North America	United States	Enteritidis	Enteritidis	1425	3333
SAL_GA0114AA	NY59550216	SRR2734583	2015	9	North America	United States	Enteritidis	Enteritidis	1425	45085
SAL_GA0115AA	NY59550178	SRR2734559	2015	9	North America	United States	Enteritidis	Enteritidis	1425	45084
SAL_GA0117AA	VNB1352-sc-2280688	ERR1069321					Enteritidis (Predicted)	Enteritidis	1425	44855
SAL_GA0119AA	VNB1343-sc-2280684	ERR1069319					Enteritidis (Predicted)	Enteritidis	1425	45087

SAL_GA0122AA	VNB1284-sc-2280679	ERR1069316		Enteritidis	42109	45081
SAL_GA0127AA	VNB1216-sc-2280670	ERR1069311	Enteritidis (Predicted)	Enteritidis	1425	44867
SAL_GA0131AA	VNB1125-sc-2280662	ERR1069307	Enteritidis (Predicted)	Enteritidis	1425	45073
SAL_GA0133AA	VNB1106-sc-2280658	ERR1069305	Enteritidis (Predicted)	Enteritidis	1425	45071
SAL_GA0134AA	VNB1094-sc-2280656	ERR1069304	Enteritidis (Predicted)	Enteritidis	1425	45068
SAL_GA0136AA	VNB1048-sc-2280653	ERR1069302	Enteritidis (Predicted)	Enteritidis	1425	45067
SAL_GA0137AA	VNB1039-sc-2280651	ERR1069301	Enteritidis (Predicted)	Enteritidis	1425	45065
SAL_GA0138AA	VNB1028-sc-2280648	ERR1069300	Enteritidis (Predicted)	Enteritidis	1425	44867
SAL_GA0139AA	VNB1010-sc-2280646	ERR1069299	Enteritidis (Predicted)	Enteritidis	1425	45064
SAL_GA0140AA	VNB985-sc-2280644	ERR1069298	Enteritidis (Predicted)	Enteritidis	1425	45063
SAL_GA0142AA	VNB899-sc-2280639	ERR1069296	Enteritidis (Predicted)	Enteritidis	1425	45062
SAL_GA0147AA	VNB789-sc-2280629	ERR1069291	Enteritidis (Predicted)	Enteritidis	1425	45057
SAL_GA0149AA	VNB719-sc-2280625	ERR1069289		Enteritidis	42115	45056
SAL_GA0150AA	VNB713-sc-2280623	ERR1069288	Enteritidis (Predicted)	Enteritidis	1425	45055
SAL_GA0151AA	VNB703-sc-2280621	ERR1069287	Enteritidis (Predicted)	Enteritidis	1425	45054
SAL_GA0152AA	VNB631-sc-2280619	ERR1069286	Enteritidis (Predicted)	Enteritidis	1425	45053
SAL_GA0155AA	VNB498-sc-2280613	ERR1069283	Enteritidis (Predicted)	Enteritidis	1425	45049
SAL_GA0157AA	VNB246-sc-2280609	ERR1069281	Enteritidis (Predicted)	Enteritidis	1425	45047
SAL_GA0158AA	VNB203-sc-2280607	ERR1069280	Enteritidis (Predicted)	Enteritidis	1425	45045
SAL_GA0159AA	VNB162-sc-2280604	ERR1069279	Enteritidis (Predicted)	Enteritidis	1425	45046
SAL_GA0160AA	VNB160-sc-2280602	ERR1069278	Enteritidis (Predicted)	Enteritidis	1425	45043
SAL_GA0161AA	VNB114-sc-2280599	ERR1069277	Enteritidis (Predicted)	Enteritidis	1425	45044
SAL_GA0163AA	VNB53-sc-2280595	ERR1069275	Enteritidis (Predicted)	Enteritidis	1425	45042
SAL_GA0164AA	VNB24-sc-2280591	ERR1069274	Enteritidis (Predicted)	Enteritidis	1425	45041
SAL_GA0165AA	2659-sc-2280589	ERR1069273	Enteritidis (Predicted)	Enteritidis	1425	45040
SAL_GA0166AA	2651-sc-2280588	ERR1069272	Enteritidis (Predicted)	Enteritidis	1425	45039
SAL_GA0167AA	2646-sc-2280586	ERR1069271	Enteritidis (Predicted)	Enteritidis	1425	45038
SAL_GA0169AA	2614-sc-2280582	ERR1069269	Enteritidis (Predicted)	Enteritidis	1425	45036
SAL_GA0170AA	2540-sc-2280580	ERR1069268	Enteritidis (Predicted)	Enteritidis	1425	45035
SAL_GA0171AA	2521-sc-2280578	ERR1069267	Enteritidis (Predicted)	Enteritidis	1425	45031
SAL_GA0175AA	2355-sc-2280570	ERR1069263	Enteritidis (Predicted)	Enteritidis	1425	44875
SAL_GA0176AA	2263-sc-2280568	ERR1069262	Enteritidis (Predicted)	Enteritidis	1425	45029
SAL_GA0177AA	2251-sc-2280566	ERR1069261	Enteritidis (Predicted)	Enteritidis	1425	45028
SAL_GA0178AA	2177-sc-2280563	ERR1069260	Enteritidis (Predicted)	Enteritidis	1425	45026
SAL_GA0179AA	2164-sc-2280562	ERR1069259	Enteritidis (Predicted)	Enteritidis	1425	45027
SAL_GA0181AA	2097-sc-2280558	ERR1069257	Enteritidis (Predicted)	Enteritidis	1425	45024
SAL_GA0182AA	2093-sc-2280557	ERR1069256	Enteritidis (Predicted)	Enteritidis	1425	45023
SAL_GA0183AA	2086-sc-2280556	ERR1069255	Enteritidis (Predicted)	Enteritidis	1425	45022
SAL_GA0184AA	1992-sc-2280555	ERR1069254	Enteritidis (Predicted)	Enteritidis	1425	45021
SAL_GA0185AA	1937-sc-2280554	ERR1069253	Enteritidis (Predicted)	Enteritidis	1425	45020
SAL_GA0186AA	1935-sc-2280553	ERR1069252	Enteritidis (Predicted)	Enteritidis	1425	44875
SAL_GA0187AA	1915-sc-2280552	ERR1069251	Enteritidis (Predicted)	Enteritidis	1425	45019
SAL_GA0188AA	1909-sc-2280551	ERR1069250	Enteritidis (Predicted)	Enteritidis	1425	45018
SAL_GA0189AA	1852-sc-2280550	ERR1069249	Enteritidis (Predicted)	Enteritidis	1425	45017
SAL_GA0190AA	1829-sc-2280549	ERR1069248	Enteritidis (Predicted)	Enteritidis	1425	45016
SAL_GA0191AA	1821-sc-2280548	ERR1069247	Enteritidis (Predicted)	Enteritidis	1425	45015
SAL_GA0193AA	1783-sc-2280546	ERR1069245	Enteritidis (Predicted)	Enteritidis	1425	45013
SAL_GA0194AA	1731-sc-2280545	ERR1069244	Enteritidis (Predicted)	Enteritidis	1425	45012
SAL_GA0195AA	1725-sc-2280544	ERR1069243	Enteritidis (Predicted)	Enteritidis	1425	45011
SAL_GA0197AA	MT16C_2_3-sc-2280811	ERR1069241	Enteritidis (Predicted)	Enteritidis	1425	44992
SAL_GA0220AA	MT28C_3_1-sc-2280826	ERR1046282	Enteritidis (Predicted)	Enteritidis	1425	44996
SAL_GA0221AA	MT28C_2_1-sc-2280824	ERR1046281	Enteritidis (Predicted)	Enteritidis	1425	44995
SAL_GA0222AA	MT28C_1_1-sc-2280821	ERR1046280	Enteritidis (Predicted)	Enteritidis	1425	44994
SAL_GA0223AA	MT25C_2_1-sc-2280819	ERR1046279	Enteritidis (Predicted)	Enteritidis	1425	44875
SAL_GA0224AA	MT25C_1_1-sc-2280817	ERR1046278	Enteritidis (Predicted)	Enteritidis	1425	44875
SAL_GA0226AA	MT16C_2_1-sc-2280809	ERR1046276	Enteritidis (Predicted)	Enteritidis	1425	44992
SAL_GA0241AA	CT08C_2_1-sc-2280780	ERR1046261	Enteritidis (Predicted)	Enteritidis	1425	44980
SAL_GA0249AA	CG12C_3_1-sc-2280772	ERR1046253	Enteritidis (Predicted)	Enteritidis	1425	44973
SAL_GA0250AA	CG12C_1_1-sc-2280771	ERR1046252	Enteritidis (Predicted)	Enteritidis	1425	44970

SAL_GA0251AA	CG11C_2_1-sc-2280770	ERR1046251	Enteritidis (Predicted)	Enteritidis	1425	44969
SAL_GA0252AA	CG07C_1_1-sc-2280769	ERR1046250	Enteritidis (Predicted)	Enteritidis	1425	44968
SAL_GA0280AA	72_V_027-sc-2280740	ERR1046222	Enteritidis (Predicted)	Enteritidis	1425	44944
SAL_GA0290AA	72_G_172-sc-2280729	ERR1046212	Enteritidis (Predicted)	Enteritidis	1425	44855
SAL_GA0330AA	KH_46-sc-2280650	ERR1046172	Enteritidis (Predicted)	Enteritidis	1425	44904
SAL_GA0331AA	Hue_99-sc-2280649	ERR1046171	Enteritidis (Predicted)	Enteritidis	1425	44903
SAL_GA0332AA	Hue_96-sc-2280647	ERR1046170	Enteritidis (Predicted)	Enteritidis	1425	44902
SAL_GA0334AA	Hue_52-sc-2280643	ERR1046168	Enteritidis (Predicted)	Enteritidis	1425	45255
SAL_GA0350AA	VNSC2508-sc-2280612	ERR1046152	Enteritidis (Predicted)	Enteritidis	1425	44889
SAL_GA0360AA	VNS20361-sc-2280590	ERR1046142	Enteritidis (Predicted)	Enteritidis	1425	44882
SAL_GA0372AA	VNB1680-sc-2280561	ERR1046130	Enteritidis (Predicted)	Enteritidis	1425	44867
SAL_GA0373AA	VNB1663-sc-2280560	ERR1046129	Enteritidis (Predicted)	Enteritidis	1425	44865
SAL_GA0374AA	VNB1662-sc-2280721	ERR1046128	Enteritidis (Predicted)	Enteritidis	1425	44875
SAL_GA0375AA	VNB1661-sc-2280719	ERR1046127	Enteritidis (Predicted)	Enteritidis	1425	44866
SAL_GA0377AA	VNB1572-sc-2280715	ERR1046125	Enteritidis (Predicted)	Enteritidis	1425	44863
SAL_GA0378AA	VNB1560-sc-2280712	ERR1046124	Enteritidis (Predicted)	Enteritidis	1425	44862
SAL_GA0380AA	VNB1523-sc-2280708	ERR1046122	Enteritidis (Predicted)	Enteritidis	1425	44860
SAL_GA0384AA	VNB1489-sc-2280699	ERR1046118	Enteritidis (Predicted)	Enteritidis	1425	44855
SAL_GA0385AA	VNB1434-sc-2280697	ERR1046117	Enteritidis (Predicted)	Enteritidis	1425	44856
SAL_GA0386AA	VNB1430-sc-2280695	ERR1046116	Enteritidis (Predicted)	Enteritidis	1425	44855
SAL_GA0387AA	VNB1425-sc-2280692	ERR1046115	Enteritidis (Predicted)	Enteritidis	1425	44854
SAL_GA0388AA	VNB1370-sc-2280691	ERR1046114	Enteritidis (Predicted)	Enteritidis	1425	44853
SAL_GA0415AA	gha60207-sc-2235410	ERR1010174	Enteritidis	Enteritidis	1425	44850
SAL_GA0416AA	gha114900-sc-2235407	ERR1010173	Enteritidis	Enteritidis	1425	44785
SAL_GA0437AA	gha601338-sc-2235365	ERR1010152	Enteritidis	Enteritidis	1425	44844
SAL_GA0447AA	gha63084-sc-2235342	ERR1010142	Typhi	Enteritidis	1425	44496
SAL_GA0448AA	gha61020-sc-2235340	ERR1010141	Enteritidis	Enteritidis	1425	44841
SAL_GA0460AA	gha113562-sc-2235315	ERR1010129	Enteritidis	Enteritidis	1425	44483
SAL_GA0461AA	gha86779-sc-2235313	ERR1010128	Enteritidis	Enteritidis	1425	44837
SAL_GA0487AA	gha114062-sc-2235395	ERR1010101	Enteritidis	Enteritidis	1425	44826
SAL_GA0497AA	sen00489-sc-2235375	ERR1010091	Typhimurium	Enteritidis	1425	44488
SAL_GA0506AA	gha113599-sc-2235201	ERR1010082	Enteritidis	Enteritidis	1425	44496
SAL_GA0524AA	gha113480-sc-2235165	ERR1010064	Enteritidis	Enteritidis	1425	44805
SAL_GA0529AA	gha112732-sc-2235153	ERR1010059	Enteritidis	Enteritidis	1425	44801
SAL_GA0552AA	gha114900-sc-2235106	ERR1010036	Enteritidis	Enteritidis	1425	44785
SAL_GA0556AA	gha113491-sc-2235099	ERR1010032	Enteritidis	Enteritidis	1425	44681
SAL_GA0560AA	gha115116-sc-2235090	ERR1010028	Enteritidis	Enteritidis	1425	44779
SAL_GA0561AA	gha113573-sc-2235088	ERR1010027	Enteritidis	Enteritidis	1425	44780
SAL_GA0587AA	bkf00121-sc-2235206	ERR1010000	Enteritidis	Enteritidis	1425	44619
SAL_GA0640AA	pw1547_3-sc-2235654	ERR998598	Enteritidis (Predicted)	Enteritidis	7942	44735
SAL_GA0722AA	gha113718-sc-2235614	ERR998502	Enteritidis	Enteritidis	1425	44582
SAL_GA0735AA	gha113491-sc-2235580	ERR998484	Enteritidis	Enteritidis	1425	44681
SAL_GA0766AA	gha83911-sc-2235537	ERR984964	Enteritidis	Enteritidis	1425	44631
SAL_GA0836AA	gha83911-sc-2235418	ERR984884	Enteritidis	Enteritidis	1425	44631
SAL_GA0847AA	gha114146-sc-2235352	ERR984873	Enteritidis	Enteritidis	1425	44621
SAL_GA0849AA	bkf00121-sc-2235348	ERR984871	Enteritidis	Enteritidis	1425	44619
SAL_GA0875AA	gha200805-sc-2235295	ERR984845	Enteritidis	Enteritidis	1425	44605
SAL_GA0882AA	gha400816-sc-2235288	ERR984838	Enteritidis (Predicted)	Enteritidis	1425	44600
SAL_GA0898AA	gha114606-sc-2235272	ERR984822	Enteritidis	Enteritidis	1425	44588
SAL_GA0908AA	gha113718-sc-2235255	ERR984812	Enteritidis	Enteritidis	1425	44582
SAL_GA0917AA	gha114543-sc-2235237	ERR984803	Enteritidis	Enteritidis	1425	44574
SAL_GA0935AA	bkf01708-sc-2235192	ERR984785	Enteritidis	Enteritidis	1425	44556
SAL_GA0945AA	gha300240-sc-2235168	ERR984775	Enteritidis	Enteritidis	1425	44544
SAL_GA0963AA	rsa639626-sc-2235121	ERR984757	Enteritidis	Enteritidis	1425	44527
SAL_GA0982AA	gnb00128-sc-2235081	ERR984738	Typhimurium	Enteritidis	1425	44510
SAL_GA0997AA	gha113599-sc-2235296	ERR984719	Enteritidis	Enteritidis	1425	44496
SAL_GA1007AA	sen00489-sc-2235156	ERR984709	Typhimurium	Enteritidis	1425	44488
SAL_GA1012AA	gha113562-sc-2235089	ERR984704	Enteritidis	Enteritidis	1425	44483
SAL_GA1154AA	AviproE-sc-2140669	ERR744295	Typhimurium	Enteritidis	8185	44362

SAL_GA1170AA	FieldSE-sc-2140653	ERR744279					Typhimurium	Enteritidis	1425	44351
SAL_GA1210AA	Gallivac-sc-2140593	ERR744238					Typhimurium	Enteritidis	1425	44316
SAL_GA1260AA	S01045_06-sc-2140532	ERR744171					Typhimurium	Enteritidis	1425	44206
SAL_GA1288AA	S4022_12-sc-2140711	ERR744329					Typhimurium	Enteritidis	1425	44178
SAL_GA1290AA	S355_13-sc-2140709	ERR744327					Typhimurium	Enteritidis	1425	44177
SAL_GA1292AA	S668_06-sc-2140706	ERR744325					Typhimurium	Enteritidis	1425	44175
SAL_GA1324AA	PO1334-sc-1993724	ERR731400					Typhi	Enteritidis	41670	44155
SAL_GA1562AA	NY-swgs1581	SRR1774123	2014	12	North America	United States	Enteritidis	Enteritidis	1425	36671
SAL_GA1621AA	SWUN 3736	SRR1946847	2010		Unresolved		Onarimon	Enteritidis	41673	44077
SAL_GA1622AA	SWUN 3733	SRR1946844	2010		Unresolved		Blegdam	Enteritidis	41673	44077
SAL_GA1623AA	SWUN 3712	SRR1946842	2010		Unresolved		Enterica	Enteritidis	41673	44077
SAL_GA1723AA	SAL3385	SRR1840657	2005				Enteritidis	Enteritidis	1425	43970
SAL_GA1724AA	SAL3369	SRR1840656	2006				Enteritidis	Enteritidis	1425	43968
SAL_GA1725AA	SAL3364	SRR1840655	2006				Enteritidis	Enteritidis	1425	43967
SAL_GA1726AA	SAL3360	SRR1840654	2005				Enteritidis	Enteritidis	1425	43966
SAL_GA1744AA	BCW_2429	SRR1840636			North America	United States	Enteritidis	Enteritidis	26543	43949
SAL_GA1769AA	BCW_2126	SRR1840611	2010		North America	United States	Enteritidis	Enteritidis	41626	43925
SAL_GA1819AA	BCW_2577	SRR1814454					Enteritidis (Predicted)	Enteritidis	1425	43879
SAL_GA1986AA	BCW_4684	SRR1816355					Enteritidis (Predicted)	Enteritidis	41007	43694
SAL_GA1987AA	BCW_4683	SRR1816354					Enteritidis (Predicted)	Enteritidis	8384	43693
SAL_GA1988AA	BCW_4682	SRR1816353					Enteritidis (Predicted)	Enteritidis	8384	43692
SAL_GA1989AA	BCW_4681	SRR1816352					Enteritidis (Predicted)	Enteritidis	8384	42977
SAL_GA1990AA	BCW_4680	SRR1816351					Enteritidis (Predicted)	Enteritidis	1425	42991
SAL_GA1991AA	BCW_4679	SRR1816350					Enteritidis (Predicted)	Enteritidis	1425	43677
SAL_GA1992AA	BCW_4678	SRR1816349					Enteritidis (Predicted)	Enteritidis	1425	42991
SAL_GA1993AA	BCW_4677	SRR1816348					Enteritidis (Predicted)	Enteritidis	8384	43691
SAL_GA1994AA	BCW_4676	SRR1816347					Enteritidis (Predicted)	Enteritidis	8384	43690
SAL_GA1995AA	BCW_4675	SRR1816346					Enteritidis (Predicted)	Enteritidis	8384	43689
SAL_GA1996AA	BCW_4674	SRR1816345					Enteritidis (Predicted)	Enteritidis	1425	43273
SAL_GA1997AA	BCW_4673	SRR1816344					Enteritidis (Predicted)	Enteritidis	1425	42991
SAL_GA1998AA	BCW_4672	SRR1816343					Enteritidis (Predicted)	Enteritidis	8384	43688
SAL_GA1999AA	BCW_4671	SRR1816342					Enteritidis (Predicted)	Enteritidis	1425	43687
SAL_GA2000AA	BCW_4670	SRR1816341					Enteritidis (Predicted)	Enteritidis	41546	43681
SAL_GA2001AA	BCW_4669	SRR1816340					Enteritidis (Predicted)	Enteritidis	8384	43680
SAL_GA2002AA	BCW_4668	SRR1816339					Enteritidis (Predicted)	Enteritidis	1425	43686
SAL_GA2003AA	BCW_4667	SRR1816338					Enteritidis (Predicted)	Enteritidis	1425	43679
SAL_GA2004AA	BCW_4666	SRR1816337					Enteritidis (Predicted)	Enteritidis	1425	43685
SAL_GA2005AA	BCW_4665	SRR1816336					Enteritidis (Predicted)	Enteritidis	1425	43678
SAL_GA2006AA	BCW_4664	SRR1816335					Enteritidis (Predicted)	Enteritidis	41494	43684
SAL_GA2007AA	BCW_4663	SRR1816334					Enteritidis (Predicted)	Enteritidis	1425	43677
SAL_GA2008AA	BCW_4662	SRR1816333					Enteritidis (Predicted)	Enteritidis	1425	42975
SAL_GA2009AA	BCW_4661	SRR1816332					Enteritidis (Predicted)	Enteritidis	1425	42989
SAL_GA2010AA	BCW_4660	SRR1816331					Enteritidis (Predicted)	Enteritidis	1425	42987
SAL_GA2012AA	BCW_4658	SRR1816329					Enteritidis (Predicted)	Enteritidis	1425	42182
SAL_GA2013AA	BCW_4657	SRR1816328					Enteritidis (Predicted)	Enteritidis	1425	42181
SAL_GA2014AA	BCW_4656	SRR1816327					Enteritidis (Predicted)	Enteritidis	1425	42975
SAL_GA2015AA	BCW_4654	SRR1816326					Enteritidis (Predicted)	Enteritidis	8384	43675
SAL_GA2016AA	BCW_4653	SRR1816325					Enteritidis (Predicted)	Enteritidis	1425	42180
SAL_GA2017AA	BCW_4652	SRR1816324					Enteritidis (Predicted)	Enteritidis	1425	42991
SAL_GA2018AA	BCW_4651	SRR1816323					Enteritidis (Predicted)	Enteritidis	1425	42974
SAL_GA2070AA	BCW_3070	SRR1815499					Enteritidis (Predicted)	Enteritidis	1425	42976
SAL_GA2092AA	BCW_3048	SRR1815477					Enteritidis (Predicted)	Enteritidis	1425	41062
SAL_GA2098AA	BCW_2906	SRR1815424					Enteritidis (Predicted)	Enteritidis	1425	42552
SAL_GA2249AA	BCW_5906	SRR1814936					Enteritidis (Predicted)	Enteritidis	1425	43499
SAL_GA2258AA	BCW_5897	SRR1814927					Enteritidis (Predicted)	Enteritidis	1425	43490
SAL_GA2300AA	BCW_5854	SRR1814885					Enteritidis (Predicted)	Enteritidis	1425	43448
SAL_GA2301AA	BCW_5853	SRR1814884					Enteritidis (Predicted)	Enteritidis	1425	43449
SAL_GA2344AA	BCW_2570	SRR1814453					Enteritidis (Predicted)	Enteritidis	1425	43404
SAL_GA2345AA	BCW_2569	SRR1814452					Enteritidis (Predicted)	Enteritidis	1425	43405

SAL_GA2346AA	BCW_2568	SRR1814451				Enteritidis (Predicted)	Enteritidis	1425	43406
SAL_GA2347AA	BCW_2566	SRR1814450				Enteritidis (Predicted)	Enteritidis	1425	43404
SAL_GA2349AA	BCW_2559	SRR1814448				Enteritidis (Predicted)	Enteritidis	1425	43402
SAL_GA2350AA	BCW_2557	SRR1814447				Enteritidis (Predicted)	Enteritidis	1425	43401
SAL_GA2351AA	BCW_2556	SRR1814446				Enteritidis (Predicted)	Enteritidis	1425	43400
SAL_GA2352AA	BCW_2553	SRR1814445				Enteritidis (Predicted)	Enteritidis	1425	43399
SAL_GA2353AA	BCW_2551	SRR1814444				Enteritidis (Predicted)	Enteritidis	1425	43398
SAL_GA2354AA	BCW_2550	SRR1814443				Enteritidis (Predicted)	Enteritidis	1425	43397
SAL_GA2355AA	BCW_2549	SRR1814442				Enteritidis (Predicted)	Enteritidis	41467	43396
SAL_GA2356AA	BCW_2544	SRR1814441				Enteritidis (Predicted)	Enteritidis	41148	43395
SAL_GA2388AA	BCW_2448	SRR1814407				Enteritidis (Predicted)	Enteritidis	1425	43745
SAL_GA2400AA	BCW_2020	SRR1814324				Enteritidis (Predicted)	Enteritidis	8384	43274
SAL_GA2401AA	BCW_2019	SRR1814323				Enteritidis (Predicted)	Enteritidis	1425	43273
SAL_GA2403AA	BCW_2017	SRR1814321				Enteritidis (Predicted)	Enteritidis	8384	43271
SAL_GA2404AA	BCW_2016	SRR1814320				Enteritidis (Predicted)	Enteritidis	8384	43270
SAL_GA2405AA	BCW_2015	SRR1814319				Enteritidis (Predicted)	Enteritidis	8384	43269
SAL_GA2406AA	BCW_2014	SRR1814318				Enteritidis (Predicted)	Enteritidis	26543	43268
SAL_GA2407AA	BCW_2013	SRR1814317				Enteritidis (Predicted)	Enteritidis	26543	43267
SAL_GA2419AA	BCW_1996	SRR1814305				Enteritidis (Predicted)	Enteritidis	26543	43255
SAL_GA2423AA	BCW_1992	SRR1814301				Enteritidis (Predicted)	Enteritidis	26543	43250
SAL_GA2425AA	BCW_1990	SRR1814299				Enteritidis (Predicted)	Enteritidis	41141	43249
SAL_GA2427AA	BCW_1988	SRR1814297				Enteritidis (Predicted)	Enteritidis	1425	43247
SAL_GA2428AA	BCW_1987	SRR1814296				Enteritidis (Predicted)	Enteritidis	1425	43246
SAL_GA2429AA	BCW_1986	SRR1814295				Enteritidis (Predicted)	Enteritidis	1425	43245
SAL_GA2430AA	BCW_1985	SRR1814294				Enteritidis (Predicted)	Enteritidis	1425	43244
SAL_GA2431AA	BCW_1984	SRR1814293				Enteritidis (Predicted)	Enteritidis	1425	43243
SAL_GA2432AA	BCW_1983	SRR1814292				Enteritidis (Predicted)	Enteritidis	1425	43242
SAL_GA2440AA	BCW_1975	SRR1814284				Enteritidis (Predicted)	Enteritidis	1425	43234
SAL_GA2742AA	CRJJGF_00171	SRR1686645	2004	North America	United States	Enteritidis	Enteritidis	1425	13840
SAL_GA2775AA	CRJJGF_00137	SRR1686612	2002	North America	United States	Enteritidis	Enteritidis	1425	41919
SAL_GA2781AA	CRJJGF_00131	SRR1686606	2005	North America	United States	Enteritidis	Enteritidis	40459	41913
SAL_GA2782AA	CRJJGF_00130	SRR1686605	2006	North America	United States	Enteritidis	Enteritidis	1425	41912
SAL_GA2783AA	CRJJGF_00129	SRR1686604	1999	North America	United States	Enteritidis	Enteritidis	1425	41911
SAL_GA2855AA	CRJJGF_00056	SRR1686531	2004	North America	United States	Enteritidis	Enteritidis	27433	41841
SAL_GA2898AA	CRJJGF_00013	SRR1686488	2010	North America	United States	Enteritidis	Enteritidis	1425	13017
SAL_GA2899AA	CRJJGF_00012	SRR1686487	1998	North America	United States	Enteritidis	Enteritidis	1425	42644
SAL_GA2900AA	CRJJGF_00011	SRR1686486	2002	North America	United States	Enteritidis	Enteritidis	1425	42645
SAL_GA2901AA	CRJJGF_00010	SRR1686485	2002	North America	United States	Enteritidis	Enteritidis	26543	42973
SAL_GA2902AA	CRJJGF_00009	SRR1686484	2010	North America	United States	Enteritidis	Enteritidis	1425	42651
SAL_GA2903AA	CRJJGF_00008	SRR1686483	2007	North America	United States	Enteritidis	Enteritidis	1425	42650
SAL_GA2904AA	CRJJGF_00007	SRR1686482	2005	North America	United States	Enteritidis	Enteritidis	1425	42643
SAL_GA2905AA	CRJJGF_00006	SRR1686481	2008	North America	United States	Enteritidis	Enteritidis	1425	42643
SAL_GA2906AA	CRJJGF_00005	SRR1686480	2001	North America	United States	Enteritidis	Enteritidis	1425	43319
SAL_GA2907AA	CRJJGF_00004	SRR1686479	2008	North America	United States	Enteritidis	Enteritidis	1425	42971
SAL_GA2991AA	60277	SRR1544563	2002	North America	United States	Enteritidis	Enteritidis	1425	41919
SAL_GA2992AA	UC06	SRR1544580	2004			Enteritidis (Predicted)	Enteritidis	26543	42999
SAL_GA2994AA	Stk # 1280	SRR1544562	1965	Europe	Slovakia	Nitra	Enteritidis	1425	42996
SAL_GA2995AA	353410	SRR1536816				Enteritidis (Predicted)	Enteritidis	1425	42997
SAL_GA2996AA	77320	SRR1536814	2004	North America	United States	Enteritidis	Enteritidis	1425	13840
SAL_GA2997AA	81748	SRR1536813	2005	North America	United States	Enteritidis	Enteritidis	40459	41913
SAL_GA2998AA	82631	SRR1536812	2006	North America	United States	Enteritidis	Enteritidis	1425	41912
SAL_GA2999AA	96-0186	SRR1536811	1996	North America	United States	Nitra	Enteritidis	26543	42995
SAL_GA3000AA	J0915	SRR1544561	2001	North America	United States	Enteritidis	Enteritidis	1425	42994
SAL_GA3001AA	UC12	SRR1544559	2002	North America	United States	Enteritidis	Enteritidis	1425	42993
SAL_GA3002AA	UC02	SRR1544558	2004	North America	United States	Enteritidis	Enteritidis	26543	42992
SAL_GA3003AA	1102933A	SRR1544578	2011	North America	United States	Enteritidis	Enteritidis	1425	42991
SAL_GA3004AA	0811210F	SRR1544577	2008	North America	United States	Enteritidis	Enteritidis	41007	42990
SAL_GA3005AA	0701376-4	SRR1544556	2007	North America	United States	Enteritidis	Enteritidis	1425	42989
SAL_GA3006AA	502571	SRR1544576	2005	North America	United States	Enteritidis	Enteritidis	8384	42988

SAL_GA3007AA	389382	SRR1544575	2002	North America	United States	Enteritidis	Enteritidis	1425	42987
SAL_GA3008AA	93-7741	SRR1544574	1993	North America	United States	Enteritidis	Enteritidis	1425	42182
SAL_GA3009AA	93-6175B	SRR1544552	1993	North America	United States	Enteritidis	Enteritidis	1425	42180
SAL_GA3010AA	34986	SRR1544572	1998			Enteritidis	Enteritidis	1425	42644
SAL_GA3011AA	353306	SRR1544551				Enteritidis (Predicted)	Enteritidis	1425	42645
SAL_GA3012AA	S-277	SRR1544550	2008			Enteritidis	Enteritidis	1425	42643
SAL_GA3013AA	J0903	SRR1544571	2001	North America	United States	Enteritidis	Enteritidis	1425	42986
SAL_GA3014AA	J0828	SRR1544549	2001			Enteritidis	Enteritidis	1425	42985
SAL_GA3015AA	76-0331	SRR1544548	1976	Asia	Saudi Arabia	Enteritidis	Enteritidis	1425	42984
SAL_GA3016AA	2010K-1554	SRR1544547	2010	North America	United States	Enteritidis	Enteritidis	1425	42983
SAL_GA3017AA	353273	SRR1544546				Enteritidis (Predicted)	Enteritidis	1425	42982
SAL_GA3018AA	353266	SRR1544545				Enteritidis (Predicted)	Enteritidis	1425	12130
SAL_GA3019AA	353262	SRR1544544				Enteritidis (Predicted)	Enteritidis	1425	41911
SAL_GA3020AA	UC13	SRR1544543	2009	North America	United States	Enteritidis	Enteritidis	1425	42981
SAL_GA3021AA	UC11	SRR1544579	2001	North America	United States	Enteritidis	Enteritidis	1425	42980
SAL_GA3022AA	UC10	SRR1544519	2004	North America	United States	Enteritidis	Enteritidis	26543	42979
SAL_GA3023AA	UC03	SRR1544518	2005	North America	United States	Enteritidis	Enteritidis	26543	42978
SAL_GA3024AA	9810102B	SRR1536831	1998	North America	United States	Enteritidis	Enteritidis	8384	42977
SAL_GA3025AA	0804789B	SRR1544539	2008	North America	United States	Enteritidis	Enteritidis	1425	42975
SAL_GA3026AA	98-9534	SRR1544567	1998	North America	United States	Enteritidis	Enteritidis	1425	42976
SAL_GA3027AA	93-7922A	SRR1536830	1993	North America	United States	Enteritidis	Enteritidis	1425	42974
SAL_GA3028AA	93-2836A	SRR1544581	1993	North America	United States	Enteritidis	Enteritidis	1425	42181
SAL_GA3029AA	97569	SRR1544537	2010	North America	United States	Enteritidis	Enteritidis	1425	13017
SAL_GA3030AA	61979	SRR1544536	2002	North America	United States	Enteritidis	Enteritidis	26543	42973
SAL_GA3031AA	98961	SRR1544565	2010	North America	United States	Enteritidis	Enteritidis	1425	42651
SAL_GA3032AA	85366	SRR1544535	2007	North America	United States	Enteritidis	Enteritidis	1425	42650
SAL_GA3033AA	78296	SRR1536828	2005	North America	United States	Enteritidis	Enteritidis	1425	42643
SAL_GA3034AA	55795	SRR1536827	2001	North America	United States	Enteritidis	Enteritidis	1425	42972
SAL_GA3035AA	S-380	SRR1536826	2008			Enteritidis	Enteritidis	1425	42971
SAL_GA3037AA	2010K-1832	SRR1536806				Enteritidis	Enteritidis	1425	42969
SAL_GA3038AA	352039	SRR1532725				Enteritidis (Predicted)	Enteritidis	3888	16445
SAL_GA3039AA	352060	SRR1532737				Enteritidis (Predicted)	Enteritidis	3888	42966
SAL_GA3040AA	352013	SRR1532734				Enteritidis (Predicted)	Enteritidis	1425	42967
SAL_GA3041AA	352034	SRR1532724				Enteritidis (Predicted)	Enteritidis	1425	42965
SAL_GA3042AA	2010K-0268	SRR1532723	2008	Asia	Thailand	Enteritidis	Enteritidis	1425	42964
SAL_GA3043AA	2010K-0264	SRR1532722	2008	Asia	Thailand	Enteritidis	Enteritidis	1425	42963
SAL_GA3044AA	2010K-0263	SRR1532719	2008	Asia	Thailand	Enteritidis	Enteritidis	1425	42962
SAL_GA3045AA	2009K1324	SRR1532718	2009	North America	United States	Enteritidis	Enteritidis	1425	42961
SAL_GA3046AA	2009K-0479	SRR1532717		Africa	Uganda	Enteritidis	Enteritidis	1424	42960
SAL_GA3047AA	2009K-0477	SRR1532716		Africa	Uganda	Enteritidis	Enteritidis	1424	42959
SAL_GA3048AA	04-0307	SRR1532714	2004	North America	United States	Enteritidis	Enteritidis	1425	42958
SAL_GA3049AA	351981	SRR1532732				Enteritidis (Predicted)	Enteritidis	26543	42957
SAL_GA3050AA	93-0063	SRR1532731	1993	South America	Puerto Rico	Enteritidis	Enteritidis	1425	42956
SAL_GA3051AA	84-1226	SRR1532712	1984	North America	United States	Enteritidis	Enteritidis	1425	42955
SAL_GA3052AA	351967	SRR1532710				Enteritidis (Predicted)	Enteritidis	1425	42953
SAL_GA3053AA	351960	SRR1532709				Enteritidis (Predicted)	Enteritidis	1425	42952
SAL_GA3054AA	351939	SRR1532707				Enteritidis (Predicted)	Enteritidis	3888	42954
SAL_GA3055AA	351956	SRR1532578				Enteritidis (Predicted)	Enteritidis	3888	42951
SAL_GA3056AA	2010K-0329	SRR1532577	2010	Africa	Mauritius	Enteritidis	Enteritidis	1425	42950
SAL_GA3057AA	2010K-0313	SRR1532576	2010	Africa	Mauritius	Enteritidis	Enteritidis	1425	42949
SAL_GA3058AA	2010K-0303	SRR1532730	2008	Asia	Thailand	Enteritidis	Enteritidis	1425	42947
SAL_GA3059AA	351916	SRR1532575				Enteritidis (Predicted)	Enteritidis	1425	42948
SAL_GA3060AA	351884	SRR1532574				Enteritidis (Predicted)	Enteritidis	1425	42946
SAL_GA3061AA	351880	SRR1532573				Enteritidis (Predicted)	Enteritidis	27775	42945
SAL_GA3062AA	351907	SRR1532580				Enteritidis (Predicted)	Enteritidis	1425	42941
SAL_GA3063AA	351900	SRR1532729				Enteritidis (Predicted)	Enteritidis	1425	42940
SAL_GA3064AA	351896	SRR1532728				Enteritidis (Predicted)	Enteritidis	1425	42939
SAL_GA3065AA	2010K-0271	SRR1532572	2008	Asia	Thailand	Enteritidis	Enteritidis	1425	42938
SAL_GA3066AA	2010K-0267	SRR1532727	2008	Asia	Thailand	Enteritidis	Enteritidis	1425	42937

SAL_GA3067AA	2010K-0262	SRR1532706	2008	Asia	Thailand	Enteritidis	Enteritidis	1425	42936
SAL_GA3069AA	2009K-1651	SRR1532571	2009	North America	United States	Enteritidis	Enteritidis	1425	42935
SAL_GA3070AA	2009K-0958	SRR1532570	2009	North America	United States	Enteritidis	Enteritidis	1425	42933
SAL_GA3071AA	351838	SRR1532726				Enteritidis (Predicted)	Enteritidis	1425	42932
SAL_GA3091AA	SAL3373	SRR1132889	2007			Enteritidis	Enteritidis	1425	42943
SAL_GA3092AA	SAL3367	SRR1132890	2006			Enteritidis	Enteritidis	1425	42919
SAL_GA3093AA	SAL3366	SRR1132891	2006			Enteritidis	Enteritidis	26543	42918
SAL_GA3094AA	SAL3357	SRR1132892	2005			Enteritidis	Enteritidis	1425	42917
SAL_GA3095AA	SAL3354	SRR1132893	2001			Enteritidis	Enteritidis	27446	42916
SAL_GA3096AA	SAL3350	SRR1132894	2002			Enteritidis	Enteritidis	1425	42915
SAL_GA3097AA	SAL3349	SRR1132895	2002			Enteritidis	Enteritidis	1425	42913
SAL_GA3098AA	SAL3347	SRR1132896	2002			Enteritidis	Enteritidis	1425	42914
SAL_GA3118AA	BCW_3418	SRR1122490	2004	North America	United States	Enteritidis	Enteritidis	1425	9568
SAL_GA3119AA	BCW_3417	SRR1122491	2004	North America	United States	Enteritidis	Enteritidis	1425	42893
SAL_GA3120AA	NCTR-SF923	SRR1122492	2009	North America	United States	Enteritidis	Enteritidis	1425	42892
SAL_GA3132AA	NCTR-SF475	SRR1122504	1999	North America	United States	Typhimurium	Enteritidis	1425	42881
SAL_GA3150AA	SAL3399	SRR1122544	2010			Enteritidis	Enteritidis	1425	42857
SAL_GA3151AA	SAL3398	SRR1122545	2010			Enteritidis	Enteritidis	1425	42863
SAL_GA3152AA	SAL3397	SRR1122546	2010			Enteritidis	Enteritidis	1425	42862
SAL_GA3153AA	SAL3388	SRR1122547	2009			Enteritidis	Enteritidis	1425	42861
SAL_GA3154AA	SAL3386	SRR1122548	2009			Enteritidis	Enteritidis	1425	42856
SAL_GA3155AA	SAL3384	SRR1122549	2005			Enteritidis	Enteritidis	1425	42855
SAL_GA3156AA	SAL3383	SRR1122550	2004			Enteritidis	Enteritidis	1425	42854
SAL_GA3157AA	SAL3382	SRR1122551	2009			Enteritidis	Enteritidis	1425	42853
SAL_GA3158AA	SAL3380	SRR1122552	2009			Enteritidis	Enteritidis	1425	42852
SAL_GA3159AA	SAL3378	SRR1122553	2003			Enteritidis	Enteritidis	1425	42851
SAL_GA3160AA	SAL3377	SRR1122554	2003			Enteritidis	Enteritidis	1425	42860
SAL_GA3161AA	SAL3376	SRR1122555	2008			Enteritidis	Enteritidis	1425	42850
SAL_GA3162AA	SAL3375	SRR1122556	2007			Enteritidis	Enteritidis	1425	42849
SAL_GA3163AA	SAL3374	SRR1122557	2007			Enteritidis	Enteritidis	1425	42848
SAL_GA3164AA	CFSAN005945	SRR1122558	2006			Enteritidis	Enteritidis	1425	42847
SAL_GA3165AA	CFSAN005942	SRR1122559	2006			Enteritidis	Enteritidis	1425	42846
SAL_GA3166AA	CFSAN005936	SRR1122560	2005			Enteritidis	Enteritidis	1425	42841
SAL_GA3167AA	CFSAN005928	SRR1122561	2002			Enteritidis	Enteritidis	1425	42845
SAL_GA3168AA	CFSAN005925	SRR1122562	2001			Enteritidis	Enteritidis	1425	42844
SAL_GA3173AA	CFSAN005903	SRR1122568	2002			Typhimurium	Enteritidis	1425	42837
SAL_GA3186AA	N37936	SRR1122581	2011	North America	United States	Enteritidis	Enteritidis	1425	42825
SAL_GA3191AA	N37909	SRR1122586	2011	North America	United States	Enteritidis	Enteritidis	1425	42820
SAL_GA3210AA	N30639	SRR1122605	2011	North America	United States	Enteritidis	Enteritidis	1425	42805
SAL_GA3211AA	N29385	SRR1122606	2011	North America	United States	Enteritidis	Enteritidis	1425	42803
SAL_GA3255AA	N23853	SRR1122651	2010	North America	United States	Enteritidis	Enteritidis	1425	42761
SAL_GA3263AA	BCW_2048	SRR1122659	2010	North America	United States	Enteritidis	Enteritidis	1425	42754
SAL_GA3269AA	BCW_2039	SRR1122665	2010	North America	United States	Enteritidis	Enteritidis	1425	42748
SAL_GA3340AA	BCW_1588	SRR1122738				Enteritidis	Enteritidis	1425	42685
SAL_GA3374AA	BCW_2426	SRR1118722				Enteritidis	Enteritidis	26543	42655
SAL_GA3379AA	CFSAN005841	SRR1106564	2010			Enteritidis	Enteritidis	1425	42651
SAL_GA3380AA	CFSAN005840	SRR1106565	2007			Enteritidis	Enteritidis	1425	42650
SAL_GA3381AA	CFSAN005839	SRR1106566	2007			9,12:-:-	Enteritidis	1425	13017
SAL_GA3382AA	CFSAN005838	SRR1106567	2006			Enteritidis	Enteritidis	1425	42649
SAL_GA3383AA	CFSAN005837	SRR1106568	2010			Enteritidis	Enteritidis	1425	13017
SAL_GA3384AA	CFSAN005836	SRR1106569	2002			Enteritidis	Enteritidis	26543	42648
SAL_GA3385AA	CFSAN005835	SRR1106570	1999			Enteritidis	Enteritidis	1425	41911
SAL_GA3387AA	CFSAN005833	SRR1106572	2002			Enteritidis	Enteritidis	1425	42645
SAL_GA3388AA	CFSAN005832	SRR1106573	1998			Enteritidis	Enteritidis	1425	42644
SAL_GA3389AA	CFSAN005831	SRR1106574	2005			Enteritidis	Enteritidis	1425	42643
SAL_GA3492AA	BCW_2877	SRR1106360				Enteritidis	Enteritidis	1425	42552
SAL_GA3505AA	BCW_2860	SRR1106373				Enteritidis	Enteritidis	1425	42552
SAL_GA3515AA	BCW_2844	SRR1106383				Enteritidis	Enteritidis	1425	42544
SAL_GA3626AA	BCW_2687	SRR1106494	2011	Asia	Iran	Enteritidis	Enteritidis	1425	42454

SAL_GA3628AA	BCW_2685	SRR1106496	2009		Africa	Mauritius	Enteritidis	Enteritidis	3888	44275
SAL_GA3629AA	BCW_2684	SRR1106497	2009		Europe	Denmark	Enteritidis	Enteritidis	1425	42452
SAL_GA3630AA	BCW_2682	SRR1106498	2006		Asia	Turkey	Enteritidis	Enteritidis	1425	42451
SAL_GA3631AA	BCW_2681	SRR1106499	2005		Unresolved	Congo	Enteritidis	Enteritidis	1424	42448
SAL_GA3632AA	BCW_2680	SRR1106500	2002		Asia	Taiwan	Enteritidis	Enteritidis	1425	42450
SAL_GA3633AA	BCW_2679	SRR1106501	1999		Asia	China	Enteritidis	Enteritidis	1425	42449
SAL_GA3662AA	CFSAN005976	SRR1060521	2010				Enteritidis	Enteritidis	1425	42426
SAL_GA3663AA	CFSAN005975	SRR1060522	2010				Enteritidis	Enteritidis	1425	44271
SAL_GA3664AA	CFSAN005974	SRR1060523	2010				Enteritidis	Enteritidis	1425	42425
SAL_GA3665AA	CFSAN005973	SRR1060524	2010				Enteritidis	Enteritidis	1425	44270
SAL_GA3666AA	CFSAN005972	SRR1060525	2010				Enteritidis	Enteritidis	1425	42424
SAL_GA3667AA	CFSAN005971	SRR1060526	2010				Enteritidis	Enteritidis	1425	14380
SAL_GA3668AA	CFSAN005970	SRR1060527	2010				Enteritidis	Enteritidis	1425	42423
SAL_GA3669AA	CFSAN005969	SRR1060528	2010				Enteritidis	Enteritidis	1425	42422
SAL_GA3670AA	CFSAN005967	SRR1060529	2009				Enteritidis	Enteritidis	1425	42421
SAL_GA3688AA	BCW_2195	SRR1060547	2011		North America	United States	Enteritidis	Enteritidis	1425	42406
SAL_GA3795AA	CDC SSU7998	SRR1060654			North America	United States	Enteritidis	Enteritidis	1425	42318
SAL_GA3847AA	BCW_1587	SRR1060706					Enteritidis	Enteritidis	1425	42278
SAL_GA3898AA	cSen_S8	ERR330010					Enteritidis (Predicted)	Enteritidis	1425	42239
SAL_GA3899AA	Sen_S3	ERR330009					Enteritidis (Predicted)	Enteritidis	1425	44227
SAL_GA4196AA	SQ0229	SRR863224	2013		North America	United States	Enteritidis	Enteritidis	1425	42182
SAL_GA4197AA	SQ0228	SRR863223	2013		North America	United States	Enteritidis	Enteritidis	1425	42181
SAL_GA4198AA	SQ0227	SRR863221	2013		North America	United States	Enteritidis	Enteritidis	1425	42180
SAL_GA4204AA	Sent-0907R11860	ERR277243					Enteritidis (Predicted)	Enteritidis	1425	42177
SAL_GA4205AA	Sent-0905R11615	ERR277242					Enteritidis (Predicted)	Enteritidis	1425	42176
SAL_GA4206AA	Sent-0910R12287	ERR277241					Enteritidis (Predicted)	Enteritidis	1425	42174
SAL_GA4207AA	Sent-0910R12234	ERR277240					Enteritidis (Predicted)	Enteritidis	1425	42175
SAL_GA4208AA	Sent-0910R12018	ERR277239					Enteritidis (Predicted)	Enteritidis	1425	42173
SAL_GA4209AA	Sent-0909R12091	ERR277238					Enteritidis (Predicted)	Enteritidis	1425	42172
SAL_GA4210AA	Sent-0905R11609	ERR277237					Enteritidis (Predicted)	Enteritidis	3888	42171
SAL_GA4211AA	Sent-0905R11565	ERR277236					Enteritidis (Predicted)	Enteritidis	1425	42170
SAL_GA4326AA	PNUSAS002421	SRR3732333	2016	4	North America	United States	Enteritidis	Enteritidis	1425	447
SAL_GA4331AA	PNUSAS002373	SRR3732313			North America	United States	Enteritidis (Predicted)	Enteritidis	1425	42204
SAL_GA4337AA	PNUSAS002641	SRR3732286	2016	5	North America	United States	Enteritidis	Enteritidis	1425	42199
SAL_GA4340AA	PNUSAS002666	SRR3732283	2016	6	North America	United States	Enteritidis	Enteritidis	1425	44222
SAL_GA4345AA	PNUSAS002661	SRR3732278			North America	United States	Enteritidis (Predicted)	Enteritidis	1425	42190
SAL_GA4356AA	4248_low_coverage_salmon	ERR1513238					Enteritidis (Predicted)	Enteritidis	1425	42233
SAL_GA4382AA	CVM-N23629	SRR3740193	2009		North America	United States	Enteritidis	Enteritidis	1425	42225
SAL_GA4384AA	CVM-N23580	SRR3740191	2009		North America	United States	I 9,12:Nonmotile	Enteritidis	1425	42223
SAL_GA4393AA	2016K-0493	SRR3743987			North America	United States	Enteritidis (Predicted)	Enteritidis	27863	40225
SAL_GA4394AA	2016K-0492	SRR3743986			North America	United States	Enteritidis (Predicted)	Enteritidis	27863	43070
SAL_GA4401AA	PNUSAS002491	SRR3743978	2016	5	North America	United States	Enteritidis	Enteritidis	27863	43297
SAL_GA4403AA	PNUSAS002639	SRR3743976	2016	6	North America	United States	Enteritidis	Enteritidis	1425	180
SAL_GA4404AA	PNUSAS002638	SRR3743975			North America	United States	Enteritidis	Enteritidis	1425	43295
SAL_GA4405AA	PNUSAS002637	SRR3743974	2016	6	North America	United States	Enteritidis	Enteritidis	1425	43294
SAL_GA4406AA	PNUSAS002630	SRR3743973	2016	6	North America	United States	Enteritidis	Enteritidis	27863	40225
SAL_GA4407AA	PNUSAS002629	SRR3743972	2016	5	North America	United States	Enteritidis	Enteritidis	1425	2668
SAL_GA4408AA	PNUSAS002594	SRR3743971	2016	5	North America	United States	Enteritidis	Enteritidis	1425	40609
SAL_GA4409AA	PNUSAS002563	SRR3743970	2016	5	North America	United States	Enteritidis	Enteritidis	1425	43064
SAL_GA4410AA	PNUSAS002562	SRR3743969	2016	5	North America	United States	Enteritidis	Enteritidis	1425	10271
SAL_GA4411AA	PNUSAS002561	SRR3743968			North America	United States	Enteritidis (Predicted)	Enteritidis	1425	43063
SAL_GA4412AA	PNUSAS002508	SRR3743967	2016	5	North America	United States	Enteritidis	Enteritidis	1425	180
SAL_GA4413AA	PNUSAS002507	SRR3743966			North America	United States	Enteritidis (Predicted)	Enteritidis	1425	43062
SAL_GA4414AA	PNUSAS002506	SRR3743965			North America	United States	Enteritidis (Predicted)	Enteritidis	27863	43293
SAL_GA4415AA	PNUSAS002505	SRR3743964			North America	United States	Enteritidis (Predicted)	Enteritidis	1425	4151
SAL_GA4416AA	PNUSAS002560	SRR3743962			North America	United States	Enteritidis (Predicted)	Enteritidis	1425	39779
SAL_GA4426AA	2016K-0466	SRR3743950			North America	United States	Enteritidis (Predicted)	Enteritidis	37133	177
SAL_GA4427AA	PNUSAS002678	SRR3743949	2016	6	North America	United States	Enteritidis	Enteritidis	1425	43055
SAL_GA4430AA	PNUSAS002675	SRR3743946	2016	6	North America	United States	Enteritidis	Enteritidis	1425	43052

SAL_GA4437AA	PNUSAS002670	SRR3743939			North America	United States	Enteritidis (Predicted)	Enteritidis	27863	43044
SAL_GA4445AA	PNUSAS002310	SRR3743930	2016	4	North America	United States	Enteritidis	Enteritidis	1425	43039
SAL_GA4446AA	PNUSAS002477	SRR3743929	2016	4	North America	United States	Enteritidis	Enteritidis	1425	180
SAL_GA4454AA	PNUSAS002215	SRR3743920	2016	4	North America	United States	Enteritidis	Enteritidis	27863	43034
SAL_GA4455AA	PNUSAS002214	SRR3743919	2016	4	North America	United States	Enteritidis	Enteritidis	1425	43033
SAL_GA4456AA	PNUSAS002213	SRR3743918	2016	3	North America	United States	Enteritidis	Enteritidis	3888	43032
SAL_GA4458AA	PNUSAS002209	SRR3743916	2016	3	North America	United States	Enteritidis	Enteritidis	1425	43030
SAL_GA4480AA	NY-N13667	SRR3745542	2007		North America	United States	Enteritidis	Enteritidis	1425	30959
SAL_GA4481AA	PNUSAS001778	SRR3745466			North America	United States	Enteritidis (Predicted)	Enteritidis	1425	617
SAL_GA4487AA	I2016009066	SRR3928693	2016	6	North America	United States	Enteritidis	Enteritidis	1425	43276
SAL_GA4545AA	CVM-N23613	SRR3765177	2009		North America	United States	Typhimurium Var. O:5-	Enteritidis	1425	42853
SAL_GA4673AA	5	traces-0UlIxwb					Enteritidis (Predicted)	Enteritidis	1425	44372
SAL_GA4674AA	6	traces-0QabEEc					Enteritidis (Predicted)	Enteritidis	1425	44374
SAL_GA4722AA	FSIS1607046	SRR3924743	2016		North America	United States	Enteritidis	Enteritidis	1425	45726
SAL_GA4739AA	PNUSAS002551	SRR3898626			North America	United States	Enteritidis (Predicted)	Enteritidis	37133	45711
SAL_GA4740AA	PNUSAS002550	SRR3898622			North America	United States	Enteritidis (Predicted)	Enteritidis	27924	45710
SAL_GA4741AA	PNUSAS002549	SRR3898621			North America	United States	Enteritidis (Predicted)	Enteritidis	1425	45709
SAL_GA4742AA	PNUSAS002688	SRR3898618			North America	United States	Enteritidis (Predicted)	Enteritidis	37133	177
SAL_GA4743AA	PNUSAS002687	SRR3898617			North America	United States	Enteritidis (Predicted)	Enteritidis	37133	45708
SAL_GA4769AA	PNUSAS002836	SRR3898516	2016	6	North America	United States	Enteritidis	Enteritidis	1425	45684
SAL_GA4771AA	PNUSAS002834	SRR3898482	2016	6	North America	United States	Enteritidis	Enteritidis	1425	45683
SAL_GA4781AA	PNUSAS002799	SRR3898177			North America	United States	Enteritidis (Predicted)	Enteritidis	1425	45672
SAL_GA4809AA	PNUSAS002779	SRR3897929	2016	5	North America	United States	Enteritidis	Enteritidis	3888	45643
SAL_GA4851AA	I2016009808	SRR3824141	2016	6	North America	United States	Enteritidis	Enteritidis	1425	45554
SAL_GA4852AA	I2016009149	SRR3824044	2016	6	North America	United States	Enteritidis	Enteritidis	1425	40903
SAL_GA4853AA	I2016008662-12	SRR3824043	2016	6	North America	United States	Enteritidis	Enteritidis	1425	45553
SAL_GA4854AA	E2016009720	SRR3824138	2016	6	North America	United States	Enteritidis	Enteritidis	1425	45552
SAL_GA4855AA	E2016009483	SRR3824040	2016	6	North America	United States	Enteritidis	Enteritidis	1425	45551
SAL_GA4856AA	E2016009919	SRR3824039	2016	6	North America	United States	Enteritidis	Enteritidis	1425	45803
SAL_GA4857AA	E2016009870	SRR3824133	2016	6	North America	United States	Enteritidis	Enteritidis	1425	45802
SAL_GA4858AA	E2016009850	SRR3824132	2016	6	North America	United States	Enteritidis	Enteritidis	34197	45550
SAL_GA4859AA	E2016009771	SRR3824131	2016	6	North America	United States	Enteritidis	Enteritidis	1425	45549
SAL_GA4860AA	E2016009765	SRR3824034	2016	6	North America	United States	Enteritidis	Enteritidis	1425	45546
SAL_GA4861AA	E2016009735	SRR3824033	2016	6	North America	United States	Enteritidis	Enteritidis	27863	45795
SAL_GA4862AA	E2016009469	SRR3824128	2016	6	North America	United States	Enteritidis	Enteritidis	1425	45545
SAL_GA4863AA	CFSAN052648	SRR3824093	2016	6	North America	United States	Enteritidis	Enteritidis	1425	45544
SAL_GA4865AA	CFSAN052649	SRR3824094	2016	6	North America	United States	Enteritidis	Enteritidis	1425	45543
SAL_GA4900AA	FSIS1607118	SRR3928714	2016		North America	United States	Enteritidis	Enteritidis	1425	45631
SAL_GA4916AA	E2016010140	SRR3928752	2016	6	North America	United States	Enteritidis	Enteritidis	1425	45620
SAL_GA4917AA	E2016010134	SRR3928751	2016	6	North America	United States	Enteritidis	Enteritidis	1425	45619
SAL_GA4959AA	CFSAN052937	SRR3929083	2016	7	North America	United States	Enteritidis	Enteritidis	1425	45619
SAL_GA4960AA	CFSAN052936	SRR3929082	2016	7	North America	United States	Enteritidis	Enteritidis	1425	45619
SAL_GA5023AA	PNUSAS002821	SRR3930407	2016	6	North America	United States	Enteritidis	Enteritidis	1425	46091
SAL_GA5025AA	PNUSAS002820	SRR3930405	2016	5	North America	United States	Enteritidis	Enteritidis	1425	46089
SAL_GA5026AA	PNUSAS002819	SRR3930404	2016	6	North America	United States	Enteritidis	Enteritidis	1425	46088
SAL_GA5027AA	PNUSAS002818	SRR3930402	2016	5	North America	United States	Enteritidis	Enteritidis	37133	46087
SAL_GA5028AA	PNUSAS002817	SRR3930401	2016	5	North America	United States	Enteritidis	Enteritidis	1425	46085
SAL_GA5029AA	PNUSAS002816	SRR3930399	2016	5	North America	United States	Enteritidis	Enteritidis	1425	46084
SAL_GA5030AA	PNUSAS002815	SRR3930398	2016	5	North America	United States	Enteritidis	Enteritidis	1425	2668
SAL_GA5031AA	PNUSAS002814	SRR3930397	2016	5	North America	United States	Enteritidis	Enteritidis	1425	46083
SAL_GA5032AA	PNUSAS002813	SRR3930394	2016	5	North America	United States	Enteritidis	Enteritidis	27863	43297
SAL_GA5033AA	PNUSAS002812	SRR3930393	2016	5	North America	United States	Enteritidis	Enteritidis	1425	46082
SAL_GA5034AA	PNUSAS002811	SRR3930392	2016	5	North America	United States	Enteritidis	Enteritidis	1425	46076
SAL_GA5035AA	PNUSAS002810	SRR3930391	2016	5	North America	United States	Enteritidis	Enteritidis	27863	46080
SAL_GA5036AA	PNUSAS002559	SRR3930390	2016	5	North America	United States	Enteritidis	Enteritidis	27863	46081
SAL_GA5037AA	PNUSAS002558	SRR3930389	2016	5	North America	United States	Enteritidis	Enteritidis	27863	46079
SAL_GA5038AA	PNUSAS002557	SRR3930388	2016	5	North America	United States	Enteritidis	Enteritidis	27863	46078
SAL_GA5039AA	PNUSAS002556	SRR3930387	2016	5	North America	United States	Enteritidis	Enteritidis	1425	46077
SAL_GA5040AA	PNUSAS002555	SRR3930385	2016	4	North America	United States	Enteritidis	Enteritidis	1425	46075

SAL_GA5041AA	PNUSAS002554	SRR3930384	2016	5	North America	United States	Enteritidis	Enteritidis	1425	46077
SAL_GA5042AA	PNUSAS002553	SRR3930383	2016	5	North America	United States	Enteritidis	Enteritidis	1425	46074
SAL_GA5043AA	PNUSAS002552	SRR3930382	2016	4	North America	United States	Enteritidis	Enteritidis	1425	46071
SAL_GA5046AA	2016K-0511	SRR3930379			North America	United States	Enteritidis (Predicted)	Enteritidis	1425	46070
SAL_GA5052AA	PNUSAS002695	SRR3930368	2016	5	North America	United States	Enteritidis	Enteritidis	1425	1026
SAL_GA5053AA	PNUSAS002694	SRR3930367	2016	5	North America	United States	Enteritidis	Enteritidis	1425	46065
SAL_GA5054AA	PNUSAS002693	SRR3930366	2016	5	North America	United States	Enteritidis	Enteritidis	1425	46064
SAL_GA5065AA	PNUSAS002683	SRR3930355			North America	United States	Enteritidis (Predicted)	Enteritidis	1425	46060
SAL_GA5066AA	PNUSAS002682	SRR3930353			North America	United States	Enteritidis (Predicted)	Enteritidis	1425	46059
SAL_GA5086AA	PNUSAS002690	SRR3930269	2016	5	North America	United States	Enteritidis	Enteritidis	1425	46040
SAL_GA5087AA	PNUSAS002849	SRR3930268			North America	United States	Enteritidis (Predicted)	Enteritidis	1425	46039
SAL_GA5088AA	PNUSAS002610	SRR3930267			North America	United States	Enteritidis (Predicted)	Enteritidis	27944	46017
SAL_GA5089AA	PNUSAS002609	SRR3930266			North America	United States	Enteritidis (Predicted)	Enteritidis	27944	46017
SAL_GA5090AA	PNUSAS002608	SRR3930265			North America	United States	Enteritidis (Predicted)	Enteritidis	27944	46038
SAL_GA5091AA	PNUSAS002607	SRR3930264			North America	United States	Enteritidis (Predicted)	Enteritidis	27944	46017
SAL_GA5092AA	PNUSAS002606	SRR3930263			North America	United States	Enteritidis (Predicted)	Enteritidis	27944	46054
SAL_GA5093AA	PNUSAS002605	SRR3930262			North America	United States	Enteritidis (Predicted)	Enteritidis	1425	46016
SAL_GA5094AA	PNUSAS002604	SRR3930261			North America	United States	Enteritidis (Predicted)	Enteritidis	1425	46015
SAL_GA5095AA	PNUSAS002603	SRR3930260			North America	United States	Enteritidis (Predicted)	Enteritidis	27904	46164
SAL_GA5096AA	PNUSAS002602	SRR3930259			North America	United States	Enteritidis (Predicted)	Enteritidis	1425	39578
SAL_GA5097AA	PNUSAS002601	SRR3930258			North America	United States	Enteritidis (Predicted)	Enteritidis	27904	46014
SAL_GA5098AA	PNUSAS002848	SRR3930256			North America	United States	Enteritidis (Predicted)	Enteritidis	1425	46163
SAL_GA5099AA	PNUSAS002600	SRR3930255			North America	United States	Enteritidis (Predicted)	Enteritidis	1425	46005
SAL_GA5100AA	PNUSAS002598	SRR3930254			North America	United States	Enteritidis (Predicted)	Enteritidis	1425	46004
SAL_GA5101AA	PNUSAS002597	SRR3930253			North America	United States	Enteritidis (Predicted)	Enteritidis	1425	39578
SAL_GA5102AA	PNUSAS002596	SRR3930252			North America	United States	Enteritidis (Predicted)	Enteritidis	1425	46003
SAL_GA5114AA	PNUSAS002703	SRR3930240	2016	5	North America	United States	Enteritidis	Enteritidis	1425	45987
SAL_GA5118AA	PNUSAS002781	SRR3930236	2016	5	North America	United States	Enteritidis	Enteritidis	1425	45983
SAL_GA5121AA	PNUSAS002698	SRR3930233	2016	5	North America	United States	Enteritidis	Enteritidis	1425	45980
SAL_GA5124AA	PNUSAS002697	SRR3930230	2016	5	North America	United States	Enteritidis	Enteritidis	1425	46133
SAL_GA5125AA	PNUSAS002890	SRR3930229	2016	5	North America	United States	Enteritidis	Enteritidis	1425	45975
SAL_GA5128AA	PNUSAS002887	SRR3930226	2016	5	North America	United States	Enteritidis	Enteritidis	1425	45973
SAL_GA5130AA	PNUSAS002885	SRR3930224	2016	5	North America	United States	Enteritidis	Enteritidis	1425	45971
SAL_GA5143AA	PNUSAS002611	SRR3930211			North America	United States	Enteritidis (Predicted)	Enteritidis	27944	45954
SAL_GA5176AA	V-CLASP-D-44	SRR3933137	2013		North America	United States	Enteritidis	Enteritidis	1425	46287
SAL_GA5180AA	V-CLASP-D-40	SRR3933133	2013		North America	United States	Enteritidis	Enteritidis	42163	46282
SAL_GA5217AA	V-CLASP-D-05	SRR3933096	2013		North America	United States	Enteritidis	Enteritidis	1425	46216
SAL_GA5220AA	V-CLASP-D-02	SRR3933093	2012		North America	United States	Enteritidis	Enteritidis	1425	46214
SAL_GA5257AA	FDA00004494	SRR3938678	2008	10	Asia	China	Enteritidis	Enteritidis	1425	46804
SAL_GA5288AA	CFSAN042921	SRR4012587	2015	9	North America	United States	Enteritidis (Predicted)	Enteritidis	1425	46783
SAL_GA5289AA	CFSAN042920	SRR4012586	2015	9	North America	United States	Enteritidis (Predicted)	Enteritidis	1425	46783
SAL_GA5290AA	CFSAN042919	SRR3934265	2015	9	North America	United States	Enteritidis (Predicted)	Enteritidis	1425	46783
SAL_GA5291AA	CFSAN042918	SRR3934264	2015	9	North America	United States	Enteritidis (Predicted)	Enteritidis	1425	46785
SAL_GA5292AA	CFSAN042917	SRR4012583	2015	9	North America	United States	Enteritidis (Predicted)	Enteritidis	1425	46783
SAL_GA5293AA	CFSAN042916	SRR3934262	2015	9	North America	United States	Enteritidis (Predicted)	Enteritidis	1425	46783
SAL_GA5294AA	CFSAN042915	SRR4012580	2015	9	North America	United States	Enteritidis (Predicted)	Enteritidis	1425	46783
SAL_GA5400AA	PNUSAS002689	SRR3933711			North America	United States	Enteritidis (Predicted)	Enteritidis	1425	447
SAL_GA5406AA	PNUSAS002947	SRR3933706	2016	6	North America	United States	Enteritidis	Enteritidis	1425	46741
SAL_GA5407AA	PNUSAS002946	SRR3933705	2016	6	North America	United States	Enteritidis	Enteritidis	1425	2067
SAL_GA5409AA	PNUSAS002945	SRR3933704	2016	6	North America	United States	Enteritidis	Enteritidis	1425	46739
SAL_GA5410AA	PNUSAS002944	SRR3933703	2016	6	North America	United States	Enteritidis	Enteritidis	1425	46738
SAL_GA5411AA	PNUSAS002824	SRR3933702	2016	5	North America	United States	Enteritidis	Enteritidis	1425	46737
SAL_GA5412AA	PNUSAS002823	SRR3933701	2016	6	North America	United States	Enteritidis	Enteritidis	1425	46735
SAL_GA5413AA	PNUSAS002822	SRR3933700	2016	6	North America	United States	Enteritidis	Enteritidis	1425	46734
SAL_GA5436AA	PNUSAS002809	SRR3933690	2016	6	North America	United States	Enteritidis	Enteritidis	27863	46722
SAL_GA5437AA	PNUSAS002808	SRR3933689	2016	6	North America	United States	Enteritidis	Enteritidis	27863	40225
SAL_GA5438AA	PNUSAS002686	SRR3933688	2016	5	North America	United States	Enteritidis	Enteritidis	1425	46721
SAL_GA5456AA	PNUSAS002949	SRR3933673	2016	6	North America	United States	Enteritidis	Enteritidis	1425	46708
SAL_GA5458AA	PNUSAS002625	SRR3933671	2016	6	North America	United States	Enteritidis	Enteritidis	1425	44222

SAL_GA5465AA	PNUSAS002621	SRR3933667	2016	5	North America	United States	Enteritidis	Enteritidis	1425	46728
SAL_GA5469AA	PNUSAS002620	SRR3933666			North America	United States	Enteritidis	Enteritidis	1425	47027
SAL_GA5476AA	PNUSAS002618	SRR3933664			North America	United States	Enteritidis (Predicted)	Enteritidis	37741	46700
SAL_GA5483AA	PNUSAS002615	SRR3933662			North America	United States	Enteritidis (Predicted)	Enteritidis	1425	46694
SAL_GA5488AA	PNUSAS002612	SRR3933659	2016	5	North America	United States	Enteritidis	Enteritidis	1425	46690
SAL_GA5489AA	PNUSAS002584	SRR3933658	2016	5	North America	United States	Enteritidis	Enteritidis	1425	46689
SAL_GA5492AA	PNUSAS002581	SRR3933655	2016	5	North America	United States	Enteritidis	Enteritidis	27863	40225
SAL_GA5494AA	PNUSAS002579	SRR3933653	2016	5	North America	United States	Enteritidis	Enteritidis	1425	46684
SAL_GA5504AA	PNUSAS002574	SRR3933648	2016	5	North America	United States	Enteritidis	Enteritidis	1425	46678
SAL_GA5506AA	PNUSAS002692	SRR3933647			North America	United States	Enteritidis (Predicted)	Enteritidis	1425	46677
SAL_GA5509AA	PNUSAS002691	SRR3933645			North America	United States	Enteritidis (Predicted)	Enteritidis	1425	47023
SAL_GA5563AA	CVM N62976	SRR3933083	2015		North America	United States	Enteritidis	Enteritidis	1425	47016
SAL_GA5581AA	CVM N62524	SRR3933065	2015		North America	United States	Enteritidis	Enteritidis	27904	46621
SAL_GA5583AA	CVM N62522	SRR3933063	2015		North America	United States	Enteritidis	Enteritidis	1425	46620
SAL_GA5598AA	CVM N62507	SRR3933049	2015		North America	United States	Enteritidis	Enteritidis	1425	46611
SAL_GA5616AA	CVM N62486	SRR3933031	2015		North America	United States	Enteritidis	Enteritidis	1425	46596
SAL_GA5619AA	CVM N62480	SRR3933028	2015		North America	United States	Enteritidis	Enteritidis	1425	46593
SAL_GA5620AA	CVM N62479	SRR3933027	2015		North America	United States	Enteritidis	Enteritidis	1425	46588
SAL_GA5626AA	CVM N62472	SRR3933021	2015		North America	United States	Enteritidis	Enteritidis	1425	46585
SAL_GA5631AA	CVM N62467	SRR3933016	2015		North America	United States	Enteritidis	Enteritidis	1425	12362
SAL_GA5633AA	CVM N62464	SRR3933014	2015		North America	United States	Enteritidis	Enteritidis	1425	46580
SAL_GA5637AA	CVM N62460	SRR3933010	2015		North America	United States	Enteritidis	Enteritidis	1425	46578
SAL_GA5638AA	CVM N62459	SRR3933009	2015		North America	United States	Enteritidis	Enteritidis	1425	46575
SAL_GA5639AA	CVM N62458	SRR3933008	2015		North America	United States	Enteritidis	Enteritidis	1425	46574
SAL_GA5646AA	CVM N62451	SRR3933001	2015		North America	United States	Enteritidis	Enteritidis	1425	46625
SAL_GA5672AA	CVM N58676	SRR3932975	2015		North America	United States	Enteritidis	Enteritidis	1425	46544
SAL_GA5687AA	CVM N58661	SRR3932960	2015		North America	United States	Enteritidis	Enteritidis	1425	46528
SAL_GA5688AA	CVM N58660	SRR3932959	2015		North America	United States	Enteritidis	Enteritidis	1425	46527
SAL_GA5698AA	CVM N58650	SRR3932949	2015		North America	United States	Enteritidis	Enteritidis	1425	46517
SAL_GA5705AA	CVM N58643	SRR3932942	2015		North America	United States	Enteritidis	Enteritidis	27901	12216
SAL_GA5735AA	CVM N56556	SRR3932912	2015		North America	United States	Enteritidis	Enteritidis	1425	46489
SAL_GA5738AA	CVM N56553	SRR3932909	2015		North America	United States	Enteritidis	Enteritidis	1425	17527
SAL_GA5754AA	CVM N56229	SRR3932893	2015		North America	United States	Enteritidis	Enteritidis	1425	46470
SAL_GA5760AA	CVM N55863	SRR3932887	2015		North America	United States	Saintpaul	Enteritidis	1425	46465
SAL_GA5761AA	CVM N55860	SRR3932886	2015		North America	United States	Heidelberg	Enteritidis	1425	46464
SAL_GA5765AA	CVM N55847	SRR3932882	2015		North America	United States	Enteritidis	Enteritidis	1425	46461
SAL_GA5924AA	CFSAN053020	SRR3944461	2016	7	North America	United States	Enteritidis	Enteritidis	1425	47009
SAL_GA5925AA	CFSAN053019	SRR3944460	2016	7	North America	United States	Enteritidis	Enteritidis	1425	47008
SAL_GA5926AA	CFSAN053018	SRR3944459	2016	7	North America	United States	Enteritidis	Enteritidis	1425	47007
SAL_GA5952AA	FSIS1607056	SRR3953436	2016		North America	United States	Enteritidis	Enteritidis	1425	47604
SAL_GA5957AA	FSIS1607057	SRR3953320	2016		North America	United States	Enteritidis	Enteritidis	1425	47604
SAL_GA5985AA	PNUSAS002216	SRR3951366	2016	4	North America	United States	Enteritidis	Enteritidis	27863	43034
SAL_GA5987AA	PNUSAS002211	SRR3951364	2016	3	North America	United States	Enteritidis	Enteritidis	27863	47577
SAL_GA5989AA	PNUSAS002859	SRR3951360			North America	United States	Enteritidis (Predicted)	Enteritidis	1425	1026
SAL_GA5990AA	PNUSAS002858	SRR3951359			North America	United States	Enteritidis (Predicted)	Enteritidis	1425	1026
SAL_GA5999AA	E2016010873	SRR3948505	2016	5	North America	United States	Enteritidis	Enteritidis	1425	47569
SAL_GA6000AA	E2016010879	SRR3948504	2016	7	North America	United States	Enteritidis	Enteritidis	27924	47564
SAL_GA6017AA	I2016010304-1	SRR3948447	2016	7	North America	United States	Enteritidis	Enteritidis	1425	47552
SAL_GA6019AA	E2016010254	SRR3948445	2016	7	North America	United States	Enteritidis	Enteritidis	27901	47551
SAL_GA6020AA	E2016010234	SRR3948444	2016	7	North America	United States	Enteritidis	Enteritidis	1425	47550
SAL_GA6021AA	E2016010179	SRR3948442	2016	6	North America	United States	Enteritidis	Enteritidis	1425	47549
SAL_GA6022AA	I2016010355	SRR3948441	2016	7	North America	United States	Enteritidis	Enteritidis	1425	47534
SAL_GA6023AA	E2016010377	SRR3948440	2016	7	North America	United States	Enteritidis	Enteritidis	42205	47548
SAL_GA6047AA	FSIS1607197	SRR3981176	2016		North America	United States	Enteritidis	Enteritidis	1425	47689
SAL_GA6064AA	PNUSAS002955	SRR3979115	2016	6	North America	United States	Enteritidis	Enteritidis	1425	47676
SAL_GA6067AA	PNUSAS003018	SRR3979112	2016	6	North America	United States	Enteritidis	Enteritidis	1425	47673
SAL_GA6068AA	PNUSAS003017	SRR3979111	2016	3	North America	United States	Enteritidis	Enteritidis	1425	47672
SAL_GA6069AA	PNUSAS003015	SRR3978852			North America	United States	Enteritidis (Predicted)	Enteritidis	1425	47671
SAL_GA6071AA	PNUSAS003113	SRR3978823			North America	United States	Enteritidis (Predicted)	Enteritidis	27863	43297

SAL_GA6072AA	PNUSAS003119	SRR3978768				North America	United States	Enteritidis (Predicted)	Enteritidis	1425	47667
SAL_GA6073AA	PNUSAS003116	SRR3978747	2016	7		North America	United States	Enteritidis	Enteritidis	1425	47666
SAL_GA6074AA	PNUSAS003118	SRR3978701	2016	7		North America	United States	Enteritidis	Enteritidis	1425	47665
SAL_GA6075AA	PNUSAS003117	SRR3978686	2016	6		North America	United States	Enteritidis	Enteritidis	1425	180
SAL_GA6076AA	PNUSAS003120	SRR3978450	2016	7		North America	United States	Enteritidis	Enteritidis	1425	47664
SAL_GA6079AA	PNUSAS003002	SRR3978447	2016	6		North America	United States	Enteritidis	Enteritidis	1425	47661
SAL_GA6080AA	PNUSAS002948	SRR3978446	2016	6		North America	United States	Enteritidis	Enteritidis	1425	47660
SAL_GA6081AA	PNUSAS002825	SRR3978445	2016	6		North America	United States	Enteritidis	Enteritidis	1425	47659
SAL_GA6092AA	PNUSAS002869	SRR3978331				North America	United States	Enteritidis (Predicted)	Enteritidis	1425	10271
SAL_GA6093AA	PNUSAS002967	SRR3978330	2016	6		North America	United States	Enteritidis	Enteritidis	1425	47652
SAL_GA6094AA	PNUSAS002966	SRR3978329	2016	6		North America	United States	Enteritidis	Enteritidis	1425	180
SAL_GA6098AA	PNUSAS002965	SRR3978325	2016	6		North America	United States	Enteritidis	Enteritidis	1425	47651
SAL_GA6100AA	PNUSAS002963	SRR3978323	2016	6		North America	United States	Enteritidis	Enteritidis	1425	47645
SAL_GA6101AA	PNUSAS002962	SRR3978322	2016	6		North America	United States	Enteritidis	Enteritidis	1425	180
SAL_GA6103AA	PNUSAS002961	SRR3978312				North America	United States	Enteritidis (Predicted)	Enteritidis	1425	47643
SAL_GA6104AA	PNUSAS002960	SRR3978311				North America	United States	Enteritidis (Predicted)	Enteritidis	1425	47642
SAL_GA6105AA	PNUSAS002959	SRR3978310				North America	United States	Enteritidis (Predicted)	Enteritidis	1425	47641
SAL_GA6107AA	PNUSAS002957	SRR3978308				North America	United States	Enteritidis (Predicted)	Enteritidis	1425	40609
SAL_GA6108AA	PNUSAS002956	SRR3978291				North America	United States	Enteritidis (Predicted)	Enteritidis	1425	180
SAL_GA6120AA	PNUSAS002696	SRR3978279	2016	5		North America	United States	Enteritidis	Enteritidis	1425	47590
SAL_GA6121AA	PNUSAS002260	SRR3978278	2016	4		North America	United States	Enteritidis	Enteritidis	1425	47632
SAL_GA6161AA	NSW_SH34	traces-0wnSkrv	2016	7	31	Oceania	Australia	Enteritidis	Enteritidis	1425	39817
SAL_GA6252AA	LSS22_S18_run08_2016	traces-0gncOuK						Enteritidis (Predicted)	Enteritidis	1425	47725
SAL_GA6253AA	LSS21_S15_run08_2016	traces-0WvsMfD						Enteritidis (Predicted)	Enteritidis	1425	47724
SAL_GA6254AA	LSS23_S21_run08_2016	traces-0ZERlMs						Enteritidis (Predicted)	Enteritidis	1425	47723
SAL_GA6255AA	LSS20_S12_run08_2016	traces-0KtdWZW						Enteritidis (Predicted)	Enteritidis	1425	47722
SAL_GA6256AA	LSS21_S15_raw_run08_201	traces-0JwyeTa						Enteritidis (Predicted)	Enteritidis	1425	47745
SAL_GA6257AA	LSS22_S18_raw_run08_201	traces-0PHXeZf						Enteritidis (Predicted)	Enteritidis	1425	47742
SAL_GA6258AA	LSS23_S21_raw_run08_201	traces-0mJtKNn						Enteritidis (Predicted)	Enteritidis	1425	47743
SAL_GA6259AA	LSS20_S12_raw_run08_201	traces-0KiHIZM						Enteritidis (Predicted)	Enteritidis	1425	47744
SAL_GA6271AA	FSIS1607275	SRR3995880	2016			North America	United States	Enteritidis	Enteritidis	1425	47979
SAL_GA6367AA	2016K-0576	SRR3993252				North America	United States	Enteritidis (Predicted)	Enteritidis	1425	47819
SAL_GA6368AA	2016K-0575	SRR3993251				North America	United States	Enteritidis (Predicted)	Enteritidis	1425	47818
SAL_GA6369AA	2016K-0578	SRR3993250				North America	United States	Enteritidis (Predicted)	Enteritidis	27863	47817
SAL_GA6370AA	2016K-0574	SRR3993249				North America	United States	Enteritidis (Predicted)	Enteritidis	1425	47816
SAL_GA6371AA	2016K-0573	SRR3993248				North America	United States	Enteritidis (Predicted)	Enteritidis	1425	47815
SAL_GA6372AA	2016K-0577	SRR3993247				North America	United States	Enteritidis (Predicted)	Enteritidis	1425	47814
SAL_GA6378AA	FDA00010467	SRR3991001	2013	5		North America	United States	Enteritidis (Predicted)	Enteritidis	1425	47808
SAL_GA6384AA	E2016011232	SRR3990991	2016	7		North America	United States	Enteritidis	Enteritidis	1425	47802
SAL_GA6385AA	I2016011230	SRR3990990	2016	7		North America	United States	Enteritidis	Enteritidis	1425	47801
SAL_GA6386AA	E2016011226	SRR3990989	2016	7		North America	United States	Enteritidis	Enteritidis	1425	47800
SAL_GA6387AA	E2016011044	SRR3990988	2016	7		North America	United States	Enteritidis	Enteritidis	1425	47848
SAL_GA6388AA	E2016011020	SRR3990987	2016	7		North America	United States	Enteritidis	Enteritidis	1425	47847
SAL_GA6390AA	E2016011001	SRR3990984	2016	7		North America	United States	Enteritidis	Enteritidis	1425	47794
SAL_GA6412AA	PNUSAS003112	SRR3996865	2016	7		North America	United States	Enteritidis	Enteritidis	26849	47912
SAL_GA6414AA	PNUSAS003155	SRR3996861				North America	United States	Enteritidis (Predicted)	Enteritidis	1425	47910
SAL_GA6415AA	PNUSAS003154	SRR3996860	2016	5		North America	United States	Enteritidis	Enteritidis	1425	47910
SAL_GA6416AA	PNUSAS003159	SRR3996859	2016	5		North America	United States	Enteritidis	Enteritidis	1425	47910
SAL_GA6417AA	PNUSAS003158	SRR3996858	2016	5		North America	United States	Enteritidis	Enteritidis	1425	47910
SAL_GA6418AA	PNUSAS003157	SRR3996857				North America	United States	Enteritidis (Predicted)	Enteritidis	1425	47910
SAL_GA6419AA	PNUSAS003156	SRR3996856	2016	5		North America	United States	Enteritidis	Enteritidis	1425	47910
SAL_GA6481AA	PNUSAS003100	SRR3996684	2016	7		North America	United States	Serotype Pending	Enteritidis	1425	47838
SAL_GA6482AA	PNUSAS003099	SRR3996683				North America	United States	Enteritidis (Predicted)	Enteritidis	1425	47838
SAL_GA6483AA	PNUSAS003098	SRR3996682	2016	6		North America	United States	Serotype Pending	Enteritidis	1425	47837
SAL_GA6484AA	PNUSAS003097	SRR3996681				North America	United States	Enteritidis (Predicted)	Enteritidis	1425	47836
SAL_GA6488AA	PNUSAS002857	SRR3996677				North America	United States	Enteritidis (Predicted)	Enteritidis	27944	46017
SAL_GA6503AA	PNUSAS002681	SRR3996659				North America	United States	Enteritidis (Predicted)	Enteritidis	1425	47779
SAL_GA6537AA	PNUSAS003092	SRR3996625	2016	6		North America	United States	Enteritidis	Enteritidis	1425	47755
SAL_GA6550AA	PNUSAS003129	SRR4000315	2016	7		North America	United States	Enteritidis	Enteritidis	1425	48074

SAL_GA6551AA	PNUSAS003127	SRR4000314	2016	7	North America	United States	Enteritidis	Enteritidis	1425	48073
SAL_GA6553AA	PNUSAS002684	SRR4000312	2016	4	North America	United States	Enteritidis	Enteritidis	27863	48072
SAL_GA6554AA	PNUSAS003088	SRR4000311			North America	United States	Enteritidis (Predicted)	Enteritidis	1425	48071
SAL_GA6559AA	PNUSAS003188	SRR4000306			North America	United States	Enteritidis (Predicted)	Enteritidis	1425	48057
SAL_GA6564AA	PNUSAS003183	SRR4000281	2016	7	North America	United States	Enteritidis (Predicted)	Enteritidis	1425	6087
SAL_GA6569AA	PNUSAS003173	SRR4000276			North America	United States	Enteritidis (Predicted)	Enteritidis	1425	36319
SAL_GA6572AA	PNUSAS003170	SRR4000252			North America	United States	Enteritidis (Predicted)	Enteritidis	1425	48057
SAL_GA6588AA	2016K-0615	SRR4000205			North America	United States	Enteritidis (Predicted)	Enteritidis	1425	48044
SAL_GA6589AA	2016K-0617	SRR4000204			North America	United States	Enteritidis (Predicted)	Enteritidis	1425	48043
SAL_GA6600AA	2016K-0546	SRR4000193			North America	United States	Enteritidis (Predicted)	Enteritidis	1425	39779
SAL_GA6605AA	PNUSAS003014	SRR4000013	2016	1	North America	United States	Enteritidis	Enteritidis	1425	48035
SAL_GA6607AA	PNUSAS003111	SRR4000011	2016	6	North America	United States	Enteritidis	Enteritidis	1425	48011
SAL_GA6621AA	PNUSAS002968	SRR3999993			North America	United States	Enteritidis (Predicted)	Enteritidis	1425	48020
SAL_GA6625AA	PNUSAS003082	SRR3999989			North America	United States	Enteritidis (Predicted)	Enteritidis	27863	48017
SAL_GA6626AA	PNUSAS003080	SRR3999988			North America	United States	Enteritidis (Predicted)	Enteritidis	1425	48016
SAL_GA6627AA	PNUSAS003077	SRR3999987	2016	7	North America	United States	Enteritidis	Enteritidis	1425	48015
SAL_GA6628AA	PNUSAS003076	SRR3999986			North America	United States	Enteritidis (Predicted)	Enteritidis	1425	48014
SAL_GA6629AA	PNUSAS003013	SRR3999985			North America	United States	Enteritidis (Predicted)	Enteritidis	1425	39863
SAL_GA6632AA	PNUSAS002599	SRR3999974			North America	United States	Enteritidis (Predicted)	Enteritidis	1425	48012
SAL_GA6647AA	PNUSAS003044	SRR3999884	2016	6	North America	United States	Enteritidis	Enteritidis	1425	48011
SAL_GA6678AA	48bd2d00-bac1-11e5-9e10-3	ERR1556250						Enteritidis	42231	48123
SAL_GA6689AA	485f2e80-bac1-11e5-9e10-3	ERR1556239					Enteritidis (Predicted)	Enteritidis	1424	48226
SAL_GA6696AA	47d00de0-bac1-11e5-9e10-3	ERR1556232					Enteritidis (Predicted)	Enteritidis	1424	48223
SAL_GA6700AA	47a10ea0-bac1-11e5-9e10-3	ERR1556228						Enteritidis	42231	48219
SAL_GA6703AA	47839b90-bac1-11e5-9e10-3	ERR1556225						Enteritidis	42231	48216
SAL_GA6708AA	47469290-bac1-11e5-9e10-3	ERR1556220					Enteritidis (Predicted)	Enteritidis	1424	48215
SAL_GA6729AA	421eaff0-bac1-11e5-aed5-3c	ERR1556199					Enteritidis (Predicted)	Enteritidis	1424	48205
SAL_GA6741AA	416f5d20-bac1-11e5-aed5-3	ERR1556187					Enteritidis (Predicted)	Enteritidis	3801	48193
SAL_GA6754AA	41046650-bac1-11e5-aed5-3	ERR1556174					Enteritidis (Predicted)	Enteritidis	1425	48183
SAL_GA6760AA	40b3ae40-bac1-11e5-aed5-3	ERR1556168					Enteritidis (Predicted)	Enteritidis	1425	48161
SAL_GA6775AA	401d61b0-bac1-11e5-aed5-3	ERR1556153					Enteritidis (Predicted)	Enteritidis	1425	48172
SAL_GA6780AA	3fe8bd20-bac1-11e5-aed5-3	ERR1556148					Enteritidis (Predicted)	Enteritidis	1425	48170
SAL_GA6781AA	3fe25480-bac1-11e5-aed5-3	ERR1556147					Enteritidis (Predicted)	Enteritidis	1425	48169
SAL_GA6790AA	3f9fa630-bac1-11e5-aed5-3c	ERR1556138					Enteritidis (Predicted)	Enteritidis	1425	48161
SAL_GA6792AA	3f8bd010-bac1-11e5-aed5-3	ERR1556136					Enteritidis (Predicted)	Enteritidis	1425	48161
SAL_GA6814AA	3fc2be90-bac1-11e5-9e10-3	ERR1556114					Enteritidis (Predicted)	Enteritidis	1424	48134
SAL_GA6815AA	3fbc07d0-bac1-11e5-9e10-3	ERR1556113					Enteritidis (Predicted)	Enteritidis	1424	48143
SAL_GA6818AA	3f993d90-bac1-11e5-9e10-3	ERR1556110					Enteritidis (Predicted)	Enteritidis	1424	48140
SAL_GA6820AA	3f8bd010-bac1-11e5-9e10-3	ERR1556108					Enteritidis (Predicted)	Enteritidis	1424	48134
SAL_GA6824AA	3f6c3a20-bac1-11e5-9e10-3	ERR1556104					Enteritidis (Predicted)	Enteritidis	1424	48130
SAL_GA6825AA	3f65aa70-bac1-11e5-9e10-3	ERR1556103					Enteritidis (Predicted)	Enteritidis	1424	48137
SAL_GA6827AA	3f4f3c40-bac1-11e5-9e10-3c	ERR1556101					Enteritidis (Predicted)	Enteritidis	1424	48135
SAL_GA6829AA	3f2aeb60-bac1-11e5-9e10-3	ERR1556099					Enteritidis (Predicted)	Enteritidis	1424	48134
SAL_GA6832AA	3ed921e0-bac1-11e5-9e10-3	ERR1556096					Enteritidis (Predicted)	Enteritidis	1424	48131
SAL_GA6834AA	3eba9d60-bac1-11e5-9e10-3	ERR1556094					Enteritidis (Predicted)	Enteritidis	1424	48130
SAL_GA6837AA	3ea65210-bac1-11e5-9e10-3	ERR1556091					Enteritidis (Predicted)	Enteritidis	1424	48125
SAL_GA6840AA	3e648e20-bac1-11e5-9e10-3	ERR1556088						Enteritidis	42231	48123
SAL_GA6982AA	I2016011407	SRR4014403	2016	7	North America	United States	Enteritidis	Enteritidis	1425	48473
SAL_GA6983AA	I2016011392	SRR4014402	2016	7	North America	United States	Enteritidis	Enteritidis	1425	48472
SAL_GA6984AA	I2016011284	SRR4014401	2016	7	North America	United States	Enteritidis	Enteritidis	1425	48471
SAL_GA6985AA	E2016011589	SRR4022300	2016	7	North America	United States	Enteritidis	Enteritidis	1425	48470
SAL_GA6986AA	E2016011581	SRR4014399	2016	7	North America	United States	Enteritidis	Enteritidis	8003	48469
SAL_GA6987AA	E2016011520	SRR4014398	2016	7	North America	United States	Enteritidis	Enteritidis	1425	48468
SAL_GA6988AA	E2016011385	SRR4014397	2016	7	North America	United States	Enteritidis	Enteritidis	27924	48467
SAL_GA7176AA	WAPHL-SAL-A01090	SRR4011134	2009		North America	United States	Enteritidis	Enteritidis	1425	48316
SAL_GA7178AA	CFSAN025361	SRR4011123	2006		Europe	United Kingdom	Enteritidis	Enteritidis	1425	48314
SAL_GA7191AA	PNUSAS003128	SRR4017862	2016	7	North America	United States	Enteritidis	Enteritidis	1425	48487
SAL_GA7401AA	2016K-0622	SRR4019599			North America	United States	Enteritidis (Predicted)	Enteritidis	1425	48607
SAL_GA7402AA	2016K-0623	SRR4019598			North America	United States	Enteritidis (Predicted)	Enteritidis	1425	48606

SAL_GA7403AA	2016K-0621	SRR4019597			North America	United States	Enteritidis (Predicted)	Enteritidis	1425	48605
SAL_GA7417AA	CVM-N23645	SRR4019219	2009		North America	United States	Enteritidis	Enteritidis	1425	48595
SAL_GA7419AA	CVM-N23611	SRR4019217	2009		North America	United States	Typhimurium Var. O:5-	Enteritidis	1425	48594
SAL_GA7450AA	14S002	traces-0DytmRr					Enteritidis (Predicted)	Enteritidis	1425	48634
SAL_GA7457AA	S38	traces-0CujaGQ					Enteritidis (Predicted)	Enteritidis	1425	48637
SAL_GA7460AA	S49	traces-0PRilIJ					Enteritidis (Predicted)	Enteritidis	1425	48638
SAL_GA7469AA	PNUSAS003384	SRR4040231	2016	7	North America	United States	Enteritidis	Enteritidis	1425	48744
SAL_GA7471AA	PNUSAS003382	SRR4040229	2016	7	North America	United States	Enteritidis	Enteritidis	1425	47667
SAL_GA7474AA	PNUSAS003379	SRR4040134	2016	7	North America	United States	Enteritidis	Enteritidis	1425	48746
SAL_GA7488AA	PNUSAS003144	SRR4040099	2016	7	North America	United States	Enteritidis	Enteritidis	1425	48711
SAL_GA7490AA	PNUSAS003143	SRR4040097	2016	7	North America	United States	Enteritidis	Enteritidis	1425	48713
SAL_GA7491AA	PNUSAS003142	SRR4040096	2016	7	North America	United States	Enteritidis	Enteritidis	27924	48714
SAL_GA7492AA	PNUSAS003141	SRR4040095	2016	7	North America	United States	Enteritidis	Enteritidis	1425	48715
SAL_GA7493AA	PNUSAS003140	SRR4040094	2016	7	North America	United States	Enteritidis	Enteritidis	33238	48753
SAL_GA7494AA	PNUSAS003139	SRR4040093			North America	United States	Enteritidis (Predicted)	Enteritidis	1425	48764
SAL_GA7495AA	PNUSAS003138	SRR4040092	2016	7	North America	United States	Enteritidis	Enteritidis	1425	48716
SAL_GA7496AA	PNUSAS003137	SRR4040091			North America	United States	Enteritidis (Predicted)	Enteritidis	1425	36319
SAL_GA7497AA	PNUSAS003136	SRR4040090			North America	United States	Enteritidis (Predicted)	Enteritidis	1425	48754
SAL_GA7498AA	PNUSAS003135	SRR4040089	2016	7	North America	United States	Enteritidis	Enteritidis	1425	48717
SAL_GA7499AA	PNUSAS003134	SRR4040088	2016	7	North America	United States	Enteritidis	Enteritidis	1425	41757
SAL_GA7500AA	PNUSAS003133	SRR4040087	2016	7	North America	United States	Enteritidis	Enteritidis	1425	48755
SAL_GA7510AA	PNUSAS003191	SRR4039937	2016	7	North America	United States	Enteritidis	Enteritidis	1425	48722
SAL_GA7511AA	PNUSAS003190	SRR4039936			North America	United States	Enteritidis (Predicted)	Enteritidis	1425	48757
SAL_GA7527AA	PNUSAS003407	SRR4039920	2016	6	North America	United States	Enteritidis	Enteritidis	1425	48758
SAL_GA7528AA	PNUSAS003406	SRR4039919	2016	6	North America	United States	Enteritidis	Enteritidis	1425	48759
SAL_GA7543AA	CFSAN025372	SRR4035055	2006		Europe	United Kingdom	Enteritidis	Enteritidis	1425	48683
SAL_GA7552AA	CFSAN025363	SRR4035036	2006		Europe	United Kingdom	Enteritidis	Enteritidis	1425	48665
SAL_GA7553AA	PNUSAS003274	SRR4032975	2016	7	North America	United States	Enteritidis	Enteritidis	1425	48689
SAL_GA7555AA	PNUSAS003272	SRR4032973	2016	7	North America	United States	Enteritidis	Enteritidis	1425	48667
SAL_GA7556AA	PNUSAS003235	SRR4032972	2016	7	North America	United States	Enteritidis	Enteritidis	1425	48668
SAL_GA7557AA	PNUSAS003234	SRR4032971	2016	7	North America	United States	Enteritidis	Enteritidis	1425	47667
SAL_GA7558AA	PNUSAS003233	SRR4032970	2016	7	North America	United States	Enteritidis	Enteritidis	1425	48669
SAL_GA7559AA	PNUSAS003231	SRR4032969	2016	7	North America	United States	Enteritidis	Enteritidis	1425	1171
SAL_GA7563AA	PNUSAS003045	SRR4032965			North America	United States	Enteritidis (Predicted)	Enteritidis	1425	48011
SAL_GA7585AA	PNUSAS003030	SRR4032943			North America	United States	Enteritidis (Predicted)	Enteritidis	1425	48698
SAL_GA7586AA	PNUSAS003029	SRR4032942			North America	United States	Enteritidis (Predicted)	Enteritidis	1425	9330
SAL_GA7587AA	PNUSAS003028	SRR4032941	2016	6	North America	United States	Enteritidis	Enteritidis	1425	41535
SAL_GA7588AA	PNUSAS003027	SRR4032940	2016	6	North America	United States	Enteritidis	Enteritidis	1425	48699
SAL_GA7592AA	PNUSAS003023	SRR4032936			North America	United States	Enteritidis (Predicted)	Enteritidis	1425	48656
SAL_GA7648AA	I2016011233-4	SRR4039117	2016	7	North America	United States	Enteritidis	Enteritidis	1425	47801
SAL_GA7649AA	E2016011924	SRR4039116	2016	8	North America	United States	Enteritidis	Enteritidis	1425	48773
SAL_GA7650AA	E2016011826	SRR4039115	2016	8	North America	United States	Enteritidis	Enteritidis	27904	48772
SAL_GA7651AA	E2016011710	SRR4039114	2016	7	North America	United States	Enteritidis	Enteritidis	1425	48785
SAL_GA7660AA	0000-0058-4276	SRR3932422	2016		North America	United States	Enteritidis (Predicted)	Enteritidis	1425	48858
SAL_GA7662AA	CFSAN037735	SRR3931884	2012		South America	Peru	Enteritidis	Enteritidis	1425	48851
SAL_GA7663AA	CFSAN037734	SRR3931883	2012		South America	Peru	Enteritidis	Enteritidis	37736	48815
SAL_GA7783AA	2014AM-0092	SRR4044679			North America	United States	Enteritidis (Predicted)	Enteritidis	1425	49094
SAL_GA7793AA	2014AM-3138	SRR4044669			North America	United States	Enteritidis (Predicted)	Enteritidis	1425	49078
SAL_GA7796AA	2014AM-2860	SRR4044666			North America	United States	Enteritidis (Predicted)	Enteritidis	1425	6087
SAL_GA7817AA	2014AM-1931	SRR4044645			North America	United States	Enteritidis (Predicted)	Enteritidis	1425	49019
SAL_GA7839AA	2014AM-1287	SRR4044607			North America	United States	Enteritidis (Predicted)	Enteritidis	1425	48967
SAL_GA7845AA	2014AM-2419	SRR4044601			North America	United States	Enteritidis (Predicted)	Enteritidis	1425	48973
SAL_GA7852AA	2014AM-1471	SRR4044594			North America	United States	Enteritidis (Predicted)	Enteritidis	1425	49034
SAL_GA7871AA	2014AM-0031	SRR4044572			North America	United States	Enteritidis (Predicted)	Enteritidis	1425	48991
SAL_GA7895AA	2014AM-2432	SRR4044548			North America	United States	Enteritidis (Predicted)	Enteritidis	1425	18562
SAL_GA7898AA	2014AM-1262	SRR4044545			North America	United States	Enteritidis (Predicted)	Enteritidis	1425	15690
SAL_GA7919AA	2014AM-3014	SRR4044462			North America	United States	Enteritidis (Predicted)	Enteritidis	1425	48878
SAL_GA7921AA	2014AM-0326	SRR4044460			North America	United States	Enteritidis (Predicted)	Enteritidis	1425	48880
SAL_GA7930AA	2014AM-1221	SRR4044450			North America	United States	Enteritidis (Predicted)	Enteritidis	1425	48888

SAL_GA7936AA	2014AM-2250	SRR4044444			North America	United States	Enteritidis (Predicted)	Enteritidis	1425	48893
SAL_GA7940AA	2014AM-1380	SRR4044437			North America	United States	Enteritidis (Predicted)	Enteritidis	1425	48897
SAL_GA7943AA	2014AM-0119	SRR4044433			North America	United States	Enteritidis (Predicted)	Enteritidis	1425	48902
SAL_GA7960AA	2014AM-2749	SRR4044416			North America	United States	Enteritidis (Predicted)	Enteritidis	1425	48918
SAL_GA7962AA	2014AM-2382	SRR4044414			North America	United States	Enteritidis (Predicted)	Enteritidis	1425	48920
SAL_GA7984AA	2014AM-1070	SRR4044376			North America	United States	Enteritidis (Predicted)	Enteritidis	3888	48940
SAL_GA7988AA	2014AM-2576	SRR4044372			North America	United States	Enteritidis (Predicted)	Enteritidis	1425	48946
SAL_GA7991AA	2014AM-0970	SRR4044369			North America	United States	Enteritidis (Predicted)	Enteritidis	1425	48949
SAL_GA8027AA	FSIS1607405	SRR4050212	2016		North America	United States	Enteritidis	Enteritidis	1425	49196
SAL_GA8038AA	I2016011767	SRR4048576	2016	8	North America	United States	Enteritidis	Enteritidis	1425	49207
SAL_GA8042AA	FDA00010338	SRR4048542	2001	1	Asia	Taiwan	Enteritidis (Predicted)	Enteritidis	1425	49228
SAL_GA8043AA	FDA00010337	SRR4048541	2001	1	Asia	Taiwan	Enteritidis (Predicted)	Enteritidis	1425	49211
SAL_GA8044AA	FDA00010336	SRR4048540	2001	1	Asia	Taiwan	Enteritidis (Predicted)	Enteritidis	1425	49230
SAL_GA8073AA	PNUSAS003386	SRR4047008	2016	7	North America	United States	Enteritidis	Enteritidis	1425	49240
SAL_GA8088AA	PNUSAS003441	SRR4046993			North America	United States	Enteritidis (Predicted)	Enteritidis	1425	49135
SAL_GA8090AA	PNUSAS003437	SRR4046991			North America	United States	Enteritidis (Predicted)	Enteritidis	1425	6085
SAL_GA8096AA	PNUSAS003357	SRR4046985	2016	7	North America	United States	Enteritidis	Enteritidis	1425	49138
SAL_GA8152AA	WAPHLSALA01152	SRR4046495	2016	8	North America	United States	Enteritidis	Enteritidis	1425	49257
SAL_GA8153AA	ADRDL_R14	SRR4046494	2015	7	North America	United States	Enteritidis (Predicted)	Enteritidis	1425	49258
SAL_GA8193AA	PNUSAS003527	SRR4054690			North America	United States	Enteritidis (Predicted)	Enteritidis	1425	49214
SAL_GA8194AA	PNUSAS003525	SRR4054689	2016	7	North America	United States	Enteritidis	Enteritidis	1425	49254
SAL_GA8206AA	PNUSAS003440	SRR4054677			North America	United States	Enteritidis (Predicted)	Enteritidis	1425	49220
SAL_GA8221AA	ADRDL_R113	SRR4054819	2016	1	North America	United States	Enteritidis (Predicted)	Enteritidis	1425	49226
SAL_GA8292AA	157055	SRR4063757	2015	9	Europe	United Kingdom	Salmonella Enteritidis	Enteritidis	3888	1717
SAL_GA8294AA	199925	SRR4063755	2015	12	Europe	United Kingdom	Salmonella Enteritidis	Enteritidis	3888	3621
SAL_GA8295AA	172513	SRR4063754	2015	10	Europe	United Kingdom	Salmonella Enteritidis	Enteritidis	3888	49375
SAL_GA8296AA	180544	SRR4063753	2015	10	Europe	United Kingdom	Salmonella Enteritidis	Enteritidis	1425	49361
SAL_GA8297AA	205599	SRR4063752	2016	1	Europe	United Kingdom	Salmonella Enteritidis	Enteritidis	3888	3621
SAL_GA8298AA	137133	SRR4063751	2015	7	Europe	United Kingdom	Salmonella Enteritidis	Enteritidis	1425	1396
SAL_GA8299AA	193395	SRR4063750	2015	12	Europe	United Kingdom	Salmonella Enteritidis	Enteritidis	3888	3621
SAL_GA8300AA	155741	SRR4063749	2015	9	Europe	United Kingdom	Salmonella Enteritidis	Enteritidis	1425	49372
SAL_GA8301AA	106568	SRR4063748	2015	4	Europe	United Kingdom	Salmonella Enteritidis	Enteritidis	3888	49364
SAL_GA8302AA	143657	SRR4063747	2015	8	Europe	United Kingdom	Salmonella Enteritidis	Enteritidis	3888	3629
SAL_GA8303AA	136739	SRR4063746	2015	7	Europe	United Kingdom	Salmonella Enteritidis	Enteritidis	3888	3621
SAL_GA8304AA	107756	SRR4063745	2015	4	Europe	United Kingdom	Salmonella Enteritidis	Enteritidis	3888	49365
SAL_GA8305AA	179663	SRR4063744	2015	10	Europe	United Kingdom	Salmonella Enteritidis	Enteritidis	1425	49339
SAL_GA8306AA	190784	SRR4063743	2015	11	Europe	United Kingdom	Salmonella Enteritidis	Enteritidis	1425	49362
SAL_GA8307AA	197076	SRR4063742	2015	12	Europe	United Kingdom	Salmonella Enteritidis	Enteritidis	3888	3623
SAL_GA8308AA	204443	SRR4063741	2016	1	Europe	United Kingdom	Salmonella Enteritidis	Enteritidis	3888	49363
SAL_GA8309AA	134048	SRR4063740	2015	7	Europe	United Kingdom	Salmonella Enteritidis	Enteritidis	3888	49366
SAL_GA8310AA	186571	SRR4063739	2015	11	Europe	United Kingdom	Salmonella Enteritidis	Enteritidis	3888	49354
SAL_GA8311AA	142221	SRR4063738	2015	8	Europe	United Kingdom	Salmonella Enteritidis	Enteritidis	3888	49337
SAL_GA8312AA	207568	SRR4063737	2016	1	Europe	United Kingdom	Salmonella Enteritidis	Enteritidis	3888	49336
SAL_GA8313AA	170333	SRR4063736	2015	9	Europe	United Kingdom	Salmonella Enteritidis	Enteritidis	3888	49343
SAL_GA8314AA	157288	SRR4063735	2015	9	Europe	United Kingdom	Salmonella Enteritidis	Enteritidis	1425	49352
SAL_GA8315AA	184418	SRR4063734	2015	11	Europe	United Kingdom	Salmonella Enteritidis	Enteritidis	3888	49341
SAL_GA8316AA	227002	SRR4063733	2016	3	Europe	United Kingdom	Salmonella Enteritidis	Enteritidis	3888	43032
SAL_GA8317AA	146204	SRR4063732	2015	8	Europe	United Kingdom	Salmonella Enteritidis	Enteritidis	3888	49353
SAL_GA8318AA	143656	SRR4063731	2015	8	Europe	United Kingdom	Salmonella Enteritidis	Enteritidis	3888	3630
SAL_GA8319AA	119477	SRR4063730	2015	6	Europe	United Kingdom	Salmonella Enteritidis	Enteritidis	3888	49335
SAL_GA8320AA	155655	SRR4063729	2015	9	Europe	United Kingdom	Salmonella Enteritidis	Enteritidis	3888	2002
SAL_GA8321AA	160816	SRR4063728	2015	9	Europe	United Kingdom	Salmonella Enteritidis	Enteritidis	3888	49346
SAL_GA8322AA	210002	SRR4063727	2016	1	Europe	United Kingdom	Salmonella Enteritidis	Enteritidis	3888	49340
SAL_GA8323AA	116574	SRR4063726	2015	5	Europe	United Kingdom	Salmonella Enteritidis	Enteritidis	3888	3633
SAL_GA8324AA	208919	SRR4063725	2016	1	Europe	United Kingdom	Salmonella Enteritidis	Enteritidis	3888	2002
SAL_GA8325AA	143565	SRR4063724	2015	8	Europe	United Kingdom	Salmonella Enteritidis	Enteritidis	3888	49345
SAL_GA8326AA	196410	SRR4063723	2015	12	Europe	United Kingdom	Salmonella Enteritidis	Enteritidis	3888	3621
SAL_GA8327AA	146114	SRR4063722	2015	8	Europe	United Kingdom	Salmonella Enteritidis	Enteritidis	1425	49356
SAL_GA8328AA	153188	SRR4063721	2015	8	Europe	United Kingdom	Salmonella Enteritidis	Enteritidis	3900	49344

SAL_GA8329AA	184488	SRR4063720	2015	11		Europe	United Kingdom	Salmonella Enteritidis	Enteritidis	44479	49342
SAL_GA8330AA	133208	SRR4063719	2015	7		Europe	United Kingdom	Salmonella Enteritidis	Enteritidis	3888	49349
SAL_GA8331AA	161939	SRR4063718	2015	9		Europe	United Kingdom	Salmonella Enteritidis	Enteritidis	3888	49347
SAL_GA8332AA	45238	SRR4063717	2014	9		Europe	United Kingdom	Salmonella Enteritidis	Enteritidis	3888	28683
SAL_GA8333AA	109884	SRR4063716	2015	5		Europe	United Kingdom	Salmonella Enteritidis	Enteritidis	3888	49357
SAL_GA8334AA	149329	SRR4063715	2015	8		Europe	United Kingdom	Salmonella Enteritidis	Enteritidis	3888	49367
SAL_GA8335AA	193389	SRR4063714	2015	12		Europe	United Kingdom	Salmonella Enteritidis	Enteritidis	3888	49338
SAL_GA8336AA	161087	SRR4063713	2015	9		Europe	United Kingdom	Salmonella Enteritidis	Enteritidis	1425	49350
SAL_GA8337AA	168370	SRR4063712	2015	9		Europe	United Kingdom	Salmonella Enteritidis	Enteritidis	3982	49348
SAL_GA8338AA	160805	SRR4063711	2015	9		Europe	United Kingdom	Salmonella Enteritidis	Enteritidis	3888	49351
SAL_GA8339AA	222583	SRR4063710	2016	2		Europe	United Kingdom	Salmonella Enteritidis	Enteritidis	3888	49355
SAL_GA8340AA	205621	SRR4063709	2016	1		Europe	United Kingdom	Salmonella Enteritidis	Enteritidis	3888	49358
SAL_GA8341AA	119438	SRR4063708	2015	6		Europe	United Kingdom	Salmonella Enteritidis	Enteritidis	1425	49371
SAL_GA8342AA	169613	SRR4063707	2015	9		Europe	United Kingdom	Salmonella Enteritidis	Enteritidis	3888	49377
SAL_GA8343AA	122198	SRR4063706	2015	6		Europe	United Kingdom	Salmonella Enteritidis	Enteritidis	3888	49376
SAL_GA8344AA	224042	SRR4063705	2016	2		Europe	United Kingdom	Salmonella Enteritidis	Enteritidis	3888	2002
SAL_GA8345AA	202277	SRR4063704	2016	1		Europe	United Kingdom	Enteritidis	Enteritidis	3888	49374
SAL_GA8346AA	155644	SRR4063703	2015	9		Europe	United Kingdom	Salmonella Enteritidis	Enteritidis	1425	49368
SAL_GA8347AA	182866	SRR4063702	2015	11		Europe	United Kingdom	Salmonella Enteritidis	Enteritidis	3888	49369
SAL_GA8348AA	149393	SRR4063701	2015	8		Europe	United Kingdom	Salmonella Enteritidis	Enteritidis	3888	49373
SAL_GA8349AA	217038	SRR4063700	2016	2		Europe	United Kingdom	Salmonella Enteritidis	Enteritidis	3888	49359
SAL_GA8350AA	209719	SRR4063699	2016	1		Europe	United Kingdom	Salmonella Enteritidis	Enteritidis	3888	49359
SAL_GA8351AA	208718	SRR4063698	2016	1		Europe	United Kingdom	Salmonella Enteritidis	Enteritidis	3888	49370
SAL_GA8352AA	160811	SRR4063697	2015	9		Europe	United Kingdom	Salmonella Enteritidis	Enteritidis	1425	49360
SAL_GA8396AA	S/20160014	traces-0WdpQqP	2014	7	1	Europe	United Kingdom	Enteritidis	Enteritidis	3941	49388
SAL_GA8397AA	S/20160818	traces-0ZwFmHg	2016	7	31	Europe	United Kingdom	Enteritidis	Enteritidis	3888	49389
SAL_GA8480AA	E2016012184	SRR4095259	2016	8		North America	United States	Enteritidis	Enteritidis	7801	49495
SAL_GA8481AA	E2016012152	SRR4095258	2016	8		North America	United States	Enteritidis	Enteritidis	1425	49491
SAL_GA8482AA	E2016012118	SRR4095257	2016	8		North America	United States	Enteritidis	Enteritidis	33238	49476
SAL_GA8483AA	E2016012117	SRR4095256	2016	8		North America	United States	Enteritidis	Enteritidis	33238	49493
SAL_GA8493AA	ADRDL_R47	SRR4093302	2015	9		North America	United States	Enteritidis (Predicted)	Enteritidis	1425	17527
SAL_GA8510AA	227047	SRR4073160	2016	3		Europe	United Kingdom	Salmonella Enteritidis	Enteritidis	3888	49416
SAL_GA8511AA	215119	SRR4073159	2016	2		Europe	United Kingdom	Salmonella Enteritidis	Enteritidis	3888	2002
SAL_GA8512AA	161949	SRR4073158	2015	9		Europe	United Kingdom	Salmonella Enteritidis	Enteritidis	3888	49417
SAL_GA8513AA	127551	SRR4073157	2015	6		Europe	United Kingdom	Salmonella Enteritidis	Enteritidis	3888	49418
SAL_GA8514AA	166234	SRR4073156	2015	9		Europe	United Kingdom	Salmonella Enteritidis	Enteritidis	3888	49419
SAL_GA8515AA	161868	SRR4073155	2015	9		Europe	United Kingdom	Salmonella Enteritidis	Enteritidis	3888	49420
SAL_GA8516AA	199845	SRR4073154	2015	12		Europe	United Kingdom	Salmonella Enteritidis	Enteritidis	3888	49421
SAL_GA8517AA	205741	SRR4073153	2016	1		Europe	United Kingdom	Salmonella Enteritidis	Enteritidis	1425	49422
SAL_GA8518AA	195750	SRR4073152	2015	12		Europe	United Kingdom	Enteritidis	Enteritidis	3888	49456
SAL_GA8519AA	182178	SRR4073151	2015	11		Europe	United Kingdom	Salmonella Enteritidis	Enteritidis	1425	49484
SAL_GA8520AA	IA-2011107414	SRR4069571	2011	9		North America	United States	Enteritidis	Enteritidis	1425	49423
SAL_GA8528AA	IA-2010126614	SRR4069563	2010	10		North America	United States	Enteritidis	Enteritidis	1425	49428
SAL_GA8544AA	IA_2011111840	SRR4069544	2011	10		North America	United States	Enteritidis	Enteritidis	1425	49442
SAL_GA8545AA	IA_2011125013	SRR4069543	2011	11		North America	United States	Enteritidis	Enteritidis	1425	49443
SAL_GA8547AA	PNUSAS003447	SRR4100165	2016	6		North America	United States	Enteritidis	Enteritidis	3888	49509
SAL_GA8548AA	PNUSAS003446	SRR4100164	2016	6		North America	United States	Enteritidis	Enteritidis	1425	49520
SAL_GA8549AA	PNUSAS003445	SRR4100163	2016	6		North America	United States	Enteritidis	Enteritidis	1425	49521
SAL_GA8550AA	PNUSAS003444	SRR4100162	2016	6		North America	United States	Enteritidis	Enteritidis	1425	49508
SAL_GA8551AA	PNUSAS003443	SRR4100152				North America	United States	Enteritidis (Predicted)	Enteritidis	1425	49528
SAL_GA8552AA	PNUSAS003442	SRR4100151	2016	5		North America	United States	Enteritidis	Enteritidis	1425	49527
SAL_GA8554AA	PNUSAS003375	SRR4100149				North America	United States	Enteritidis (Predicted)	Enteritidis	1425	49525
SAL_GA8559AA	PNUSAS003370	SRR4100144				North America	United States	Enteritidis (Predicted)	Enteritidis	1425	49539
SAL_GA8582AA	PNUSAS003428	SRR4099288	2016	7		North America	United States	Enteritidis	Enteritidis	1425	49501
SAL_GA8599AA	FDA00009522	SRR4098705	2015	10		North America	United States	Enteritidis (Predicted)	Enteritidis	1425	49499
SAL_GA8624AA	PNUSAS003427	SRR4101379	2016	7		North America	United States	Enteritidis	Enteritidis	1425	49550
SAL_GA8625AA	PNUSAS003426	SRR4101378	2016	7		North America	United States	Enteritidis	Enteritidis	1425	47848
SAL_GA8626AA	PNUSAS003425	SRR4101377	2016	7		North America	United States	Enteritidis	Enteritidis	1425	49587
SAL_GA8627AA	PNUSAS003424	SRR4101376	2016	7		North America	United States	Enteritidis	Enteritidis	1425	49588

SAL_GA8628AA	PNUSAS003423	SRR4101375	2016	7		North America	United States	Enteritidis	Enteritidis	1425	49591
SAL_GA8629AA	PNUSAS003422	SRR4101374				North America	United States	Enteritidis (Predicted)	Enteritidis	1425	49566
SAL_GA8637AA	PNUSAS003533	SRR4101366	2016	7		North America	United States	Enteritidis	Enteritidis	1425	49570
SAL_GA8639AA	PNUSAS003518	SRR4101362	2016	7		North America	United States	Enteritidis	Enteritidis	1425	49546
SAL_GA8640AA	PNUSAS003517	SRR4101361				North America	United States	Enteritidis (Predicted)	Enteritidis	1425	49548
SAL_GA8652AA	PNUSAS003506	SRR4101350				North America	United States	Enteritidis	Enteritidis	1425	49559
SAL_GA8671AA	PNUSAS003263	SRR4101262				North America	United States	Enteritidis (Predicted)	Enteritidis	1425	49589
SAL_GA8679AA	H124521395	traces-0BUHFLa						Enteritidis	Enteritidis	1425	26091
SAL_GA8744AA	CFSAN025377	SRR4120114	2006			Europe	United Kingdom	Enteritidis	Enteritidis	1425	49647
SAL_GA8777AA	PNUSAS003472	SRR4176769	2016	7		North America	United States	Enteritidis	Enteritidis	1425	49686
SAL_GA8778AA	PNUSAS003471	SRR4176768	2016	7		North America	United States	Enteritidis	Enteritidis	1425	49687
SAL_GA8779AA	PNUSAS003470	SRR4176766	2016	7		North America	United States	Enteritidis	Enteritidis	1425	49723
SAL_GA8780AA	PNUSAS003469	SRR4176765	2016	7		North America	United States	Enteritidis	Enteritidis	45548	49688
SAL_GA8795AA	PNUSAS002685	SRR4176741				North America	United States	Enteritidis (Predicted)	Enteritidis	1425	49691
SAL_GA8807AA	PNUSAS003678	SRR4176728				North America	United States	Enteritidis (Predicted)	Enteritidis	34197	49744
SAL_GA8809AA	PNUSAS003676	SRR4176726				North America	United States	Enteritidis (Predicted)	Enteritidis	1425	49727
SAL_GA8818AA	E2016013470	SRR4175564	2016	8		North America	United States	Enteritidis	Enteritidis	1425	49709
SAL_GA8819AA	E2016013100	SRR4175563	2016	8		North America	United States	Enteritidis	Enteritidis	1425	49710
SAL_GA8828AA	I2016012753	SRR4175554	2016	8		North America	United States	Enteritidis	Enteritidis	1425	49740
SAL_GA8829AA	I2016012750	SRR4175553	2016	8		North America	United States	Enteritidis	Enteritidis	1425	49741
SAL_GA8830AA	I2016012340-1	SRR4175552	2016	8		North America	United States	Enteritidis	Enteritidis	1425	48785
SAL_GA8831AA	E2016012974	SRR4175551	2016	8		North America	United States	Enteritidis	Enteritidis	1425	49713
SAL_GA8832AA	E2016012932	SRR4175550	2016	8		North America	United States	Enteritidis	Enteritidis	1425	49735
SAL_GA8833AA	E2016012931	SRR4175549	2016	8		North America	United States	Enteritidis	Enteritidis	1425	49731
SAL_GA8834AA	E2016012926	SRR4175548	2016	8		North America	United States	Enteritidis	Enteritidis	1425	49728
SAL_GA8835AA	E2016012907	SRR4175547	2016	8		North America	United States	Enteritidis	Enteritidis	1425	49709
SAL_GA8836AA	E2016012846	SRR4175546	2016	8		North America	United States	Enteritidis	Enteritidis	1425	45549
SAL_GA8837AA	E2016012366	SRR4175545	2016	8		North America	United States	Enteritidis	Enteritidis	34197	49674
SAL_GA8838AA	E2016012283	SRR4175544	2016	8		North America	United States	Enteritidis	Enteritidis	1425	49714
SAL_GA8879AA	PNUSAS003729	SRR4190267	2016	8		North America	United States	Enteritidis	Enteritidis	1425	49782
SAL_GA8882AA	PNUSAS003623	SRR4190264	2016	7		North America	United States	Enteritidis	Enteritidis	1425	49254
SAL_GA8884AA	PNUSAS003622	SRR4190262	2016	7		North America	United States	Enteritidis	Enteritidis	1425	49804
SAL_GA8885AA	PNUSAS003474	SRR4190261	2016	7		North America	United States	Enteritidis	Enteritidis	1425	49813
SAL_GA8928AA	PNUSAS003270	SRR4190217				North America	United States	Enteritidis (Predicted)	Enteritidis	1425	49771
SAL_GA8949AA	S/20160704	traces-0oWFntY	2016	7	13	Europe	United Kingdom	Enteritidis	Enteritidis	3941	49815
SAL_GA8950AA	S/20160745	traces-0THVfbs	2016	7	22	Europe	United Kingdom	Enteritidis	Enteritidis	3888	3621
SAL_GA8951AA	S/20160748	traces-0wiaSUz	2016	7	23	Europe	United Kingdom	Enteritidis	Enteritidis	3888	49826
SAL_GA8952AA	S/20160753	traces-0pviIcq	2016	7	23	Europe	United Kingdom	Enteritidis	Enteritidis	3888	49825
SAL_GA8953AA	S/20160782	traces-0aoNTSr	2016	7	27	Europe	United Kingdom	Enteritidis	Enteritidis	3888	3621
SAL_GA8954AA	S/20160804	traces-0nhWWdk	2016	7	28	Europe	United Kingdom	Enteritidis	Enteritidis	3888	49824
SAL_GA8956AA	S/20160805	traces-0OcSAaw	2016	7	28	Europe	United Kingdom	Enteritidis	Enteritidis	3888	49823
SAL_GA8957AA	S/20160810	traces-0zUzShM	2016	7	29	Europe	United Kingdom	Enteritidis	Enteritidis	3888	49822
SAL_GA8958AA	S/20160812	traces-0QEjrqX	2016	7	29	Europe	United Kingdom	Enteritidis	Enteritidis	3888	49822
SAL_GA8959AA	S/20160813	traces-0VpfFmn	2016	8	2	Europe	United Kingdom	Enteritidis	Enteritidis	3888	49816
SAL_GA8960AA	S/20160814	traces-0qkKCGU	2016	7	29	Europe	United Kingdom	Enteritidis	Enteritidis	3941	49821
SAL_GA8961AA	S/20160820	traces-0WCjRLU	2016	8	1	Europe	United Kingdom	Enteritidis	Enteritidis	3941	49820
SAL_GA8962AA	S/20160825	traces-0CwSXYs	2016	8	1	Europe	United Kingdom	Enteritidis	Enteritidis	3888	2002
SAL_GA8965AA	S/20160831	traces-0DtiBwA	2016	8	3	Europe	United Kingdom	Enteritidis	Enteritidis	3888	49819
SAL_GA8967AA	S/20160836	traces-0eOKYIA	2016	8	4	Europe	United Kingdom	Enteritidis	Enteritidis	3888	49818
SAL_GA8969AA	S/20160837	traces-0TnwQiB	2016	8	4	Europe	United Kingdom	Enteritidis	Enteritidis	3888	3621
SAL_GA8970AA	S/20160840	traces-0VHJTyQ	2016	8	8	Europe	United Kingdom	Enteritidis	Enteritidis	3888	49817
SAL_GA8971AA	S/20160852	traces-0sooyuo	2016	8	6	Europe	United Kingdom	Enteritidis	Enteritidis	3888	2002
SAL_GA8974AA	S/20160863	traces-0NGmhyN	2016	8	5	Europe	United Kingdom	Enteritidis	Enteritidis	3888	49816
SAL_GA8975AA	S/20160865	traces-0MhlsLL	2016	8	11	Europe	United Kingdom	Enteritidis	Enteritidis	3888	3621
SAL_GA8976AA	S/20160866	traces-0uvRSMC	2016	8	4	Europe	United Kingdom	Enteritidis	Enteritidis	3888	2002
SAL_GA9067AA	E2016013647	SRR4237886	2016	8		North America	United States	Enteritidis	Enteritidis	1425	49943
SAL_GA9068AA	E2016013656	SRR4237885	2016	8		North America	United States	Enteritidis	Enteritidis	1425	49938
SAL_GA9134AA	C140686	traces-0YFBzwG	2014			Europe	Luxembourg	Enteritidis	Enteritidis	1425	49976
SAL_GA9143AA	14055902	traces-0QqHshV	2014			Europe	Luxembourg	Enteritidis	Enteritidis	1425	49970

SAL_GA9144AA	15043002	traces-0JMKXOm	2015			Europe	Luxembourg	Enteritidis	Enteritidis	1425	49958
SAL_GA9145AA	14049912	traces-0mjOduo	2014			Europe	Luxembourg	Enteritidis	Enteritidis	1425	49968
SAL_GA9147AA	C131717	traces-0DKvloM	2013			Europe	Luxembourg	Enteritidis	Enteritidis	1425	NaN
SAL_GA9158AA	15042024	traces-0AsyYtV	2015			Europe	Luxembourg	Enteritidis	Enteritidis	3888	49978
SAL_GA9175AA	CFSAN031538	SRR4243084	2005	1		Africa	Ethiopia	Enteritidis	Enteritidis	1425	50002
SAL_GA9176AA	CFSAN031537	SRR4243083	2005	1		Africa	Ethiopia	Enteritidis	Enteritidis	1425	49981
SAL_GA9177AA	CFSAN031536	SRR4243082	2005	1		Africa	Ethiopia	Enteritidis	Enteritidis	1425	50003
SAL_GA9178AA	CFSAN031535	SRR4243081	2005	1		Africa	Ethiopia	Enteritidis	Enteritidis	1425	49986
SAL_GA9200AA	CFSAN031513	SRR4243051	2005	1		Africa	Ethiopia	I:9,12:-:-	Enteritidis	1425	50014
SAL_GA9201AA	CFSAN031512	SRR4243050	2005	1		Africa	Ethiopia	I:9,12:-:-	Enteritidis	1425	49993
SAL_GA9212AA	14046049	traces-0nmMNMr	2014			Europe	Luxembourg	Enteritidis	Enteritidis	3888	24765
SAL_GA9214AA	15050407	traces-0msgoCu	2015			Europe	Luxembourg	Enteritidis	Enteritidis	1425	2028
SAL_GA9219AA	16026942	traces-0eWcLmd	2016			Europe	Luxembourg	Enteritidis	Enteritidis	1425	50031
SAL_GA9282AA	PNUSAS003865	SRR4244393	2016	7		North America	United States	Enteritidis	Enteritidis	1425	50076
SAL_GA9302AA	FSIS1607531	SRR4252419	2016			North America	United States	Enteritidis	Enteritidis	1425	50101
SAL_GA9335AA	S/20160924	traces-0dVBqVJ	2016	8	19	Europe	United Kingdom	Enteritidis	Enteritidis	3888	50134
SAL_GA9336AA	S/20160926	traces-0OktoVZ	2016	8	21	Europe	United Kingdom	Enteritidis	Enteritidis	3888	50136
SAL_GA9337AA	S/20160951	traces-0bToyWF	2016	8	26	Europe	United Kingdom	Enteritidis	Enteritidis	3888	50135
SAL_GA9347AA	E2016013694	SRR4256755	2016	8		North America	United States	Enteritidis	Enteritidis	1425	49943
SAL_GA9348AA	E2016013872-1	SRR4256617	2016	9		North America	United States	Enteritidis	Enteritidis	1425	50171
SAL_GA9349AA	E2016013873	SRR4256616	2016	9		North America	United States	Enteritidis	Enteritidis	1425	50154
SAL_GA9351AA	I2016013863	SRR4256615	2016	8		North America	United States	Enteritidis	Enteritidis	1425	50170
SAL_GA9352AA	I2016013970-1	SRR4256614	2016	9		North America	United States	Enteritidis	Enteritidis	1425	50161
SAL_GA9357AA	ADRDL_R162	SRR4256096	2016	3		North America	United States	Enteritidis (Predicted)	Enteritidis	1425	50172
SAL_GA9375AA	CFSAN055194	SRR4292689	2016	8		North America	United States	Enteritidis	Enteritidis	1425	50155
SAL_GA9382AA	CDPH_C73	SRR5481462						Enteritidis	Enteritidis	1425	12116
SAL_GA9393AA	PNUSAS003016	SRR4273717	2016	3		North America	United States	Enteritidis	Enteritidis	1425	50202
SAL_GA9395AA	PNUSAS003627	SRR4273715				North America	United States	Enteritidis (Predicted)	Enteritidis	1425	50204
SAL_GA9397AA	PNUSAS003625	SRR4273713				North America	United States	Enteritidis (Predicted)	Enteritidis	1425	50224
SAL_GA9398AA	PNUSAS003624	SRR4273712				North America	United States	Enteritidis (Predicted)	Enteritidis	1425	50217
SAL_GA9402AA	PNUSAS003698	SRR4273708	2016	7		North America	United States	Enteritidis	Enteritidis	1425	50207
SAL_GA9406AA	PNUSAS003694	SRR4273704	2016	7		North America	United States	Enteritidis	Enteritidis	1425	50208
SAL_GA9419AA	PNUSAS003860	SRR4273691				North America	United States	Enteritidis (Predicted)	Enteritidis	1425	49135
SAL_GA9421AA	PNUSAS003858	SRR4273689	2016	8		North America	United States	Enteritidis	Enteritidis	1425	50180
SAL_GA9422AA	PNUSAS003857	SRR4273688	2016	8		North America	United States	Enteritidis	Enteritidis	27863	50181
SAL_GA9423AA	PNUSAS003856	SRR4273687	2016	8		North America	United States	Enteritidis	Enteritidis	1425	50182
SAL_GA9430AA	PNUSAS003874	SRR5195279	2016	7		North America	United States	Enteritidis	Enteritidis	1425	50189
SAL_GA9497AA	CVM N62944	SRR4280620	2015			North America	United States	Enteritidis	Enteritidis	1425	50286
SAL_GA9515AA	CVM N62435	SRR4280602	2015			North America	United States	Enteritidis	Enteritidis	1425	50274
SAL_GA9516AA	CVM N62434	SRR4280601	2015			North America	United States	Enteritidis	Enteritidis	1425	50247
SAL_GA9524AA	CVM N59313	SRR4280593	2015			North America	United States	Enteritidis	Enteritidis	1425	50283
SAL_GA9527AA	CVM N59310	SRR4280590	2015			North America	United States	Enteritidis	Enteritidis	1425	17527
SAL_GA9542AA	CVM N58678	SRR4280575	2015			North America	United States	Enteritidis	Enteritidis	1425	50305
SAL_GA9561AA	FDA00003904	SRR4279932	2011	7		North America	United States	Enteritidis	Enteritidis	1425	50261
SAL_GA9588AA	FSIS1607716	SRR4288985	2016			North America	United States	Enteritidis	Enteritidis	1425	50294
SAL_GA9606AA	1091601542	traces-0LrwNgX				Europe	Netherlands	Enteritidis	Enteritidis	3888	2002
SAL_GA9607AA	1091601510	traces-0rQUdiu				Europe	Netherlands	Enteritidis	Enteritidis	3888	2002
SAL_GA9608AA	1091601413	traces-0jyrkls				Europe	Netherlands	Enteritidis	Enteritidis	3888	50297
SAL_GA9609AA	1091601343	traces-0qmckMD				Europe	Netherlands	Enteritidis	Enteritidis	3888	50289
SAL_GA9610AA	1091601248	traces-0rKtIeb				Europe	Netherlands	Enteritidis	Enteritidis	3888	2002
SAL_GA9611AA	1091601162	traces-0RRDNHA				Europe	Netherlands	Enteritidis	Enteritidis	3888	50290
SAL_GA9612AA	1091601118	traces-0jBqEif				Europe	Netherlands	Enteritidis	Enteritidis	3888	50307
SAL_GA9613AA	1091601072	traces-0rjpBCU				Europe	Netherlands	Enteritidis	Enteritidis	3888	2002
SAL_GA9614AA	1091601003	traces-0oPoflY				Europe	Netherlands	Enteritidis	Enteritidis	3888	2002
SAL_GA9615AA	1091600887	traces-0GIPtkh				Europe	Netherlands	Enteritidis	Enteritidis	3888	2002
SAL_GA9616AA	16EP000978	traces-0IuJGpK				Europe	Norway	Enteritidis	Enteritidis	3941	50288
SAL_GA9617AA	16EP000866	traces-0pJtqTp				Europe	Norway	Enteritidis	Enteritidis	3888	50309
SAL_GA9645AA	CFSAN001001	SRR4293084						Enteritidis	Enteritidis	27433	50417
SAL_GA9649AA	CFSAN000997	SRR4293058						Enteritidis	Enteritidis	1425	50424

SAL_GA9733AA	E2016014437	SRR4292712	2016	9		North America	United States	Enteritidis	Enteritidis	1425	50366
SAL_GA9752AA	IA-2011060617	SRR4292682	2011			North America	United States	Enteritidis (Predicted)	Enteritidis	1425	50390
SAL_GA9757AA	IA-2011088535	SRR4292677	2011			North America	United States	Enteritidis (Predicted)	Enteritidis	1425	50355
SAL_GA9764AA	NYVetLIRN-14	SRR4292618	2016	6		North America	United States	Enteritidis	Enteritidis	27924	50369
SAL_GA9798AA	PNUSAS003759	SRR4292015	2016	8		North America	United States	Enteritidis	Enteritidis	1425	50341
SAL_GA9799AA	PNUSAS003758	SRR4292014	2016	8		North America	United States	Enteritidis	Enteritidis	3888	2002
SAL_GA9800AA	PNUSAS003757	SRR4292013	2016	8		North America	United States	Enteritidis	Enteritidis	1425	50360
SAL_GA9802AA	PNUSAS003755	SRR4292011	2016	8		North America	United States	Enteritidis	Enteritidis	1425	50329
SAL_GA9803AA	PNUSAS003754	SRR4292010	2016	8		North America	United States	Enteritidis	Enteritidis	1425	50324
SAL_GA9805AA	PNUSAS003752	SRR4292008	2016	7		North America	United States	Enteritidis	Enteritidis	1425	50336
SAL_GA9812AA	PNUSAS003968	SRR4292001	2016	8		North America	United States	Enteritidis (Predicted)	Enteritidis	1425	49135
SAL_GA9813AA	PNUSAS003967	SRR4292000	2016	8		North America	United States	Enteritidis (Predicted)	Enteritidis	1425	49135
SAL_GA9814AA	PNUSAS003861	SRR4291999	2016	8		North America	United States	Enteritidis (Predicted)	Enteritidis	1425	49135
SAL_GA9854AA	16042880	traces-0AhDKEV	2016			Europe	Luxembourg	Enteritidis	Enteritidis	3888	81310
SAL_GA9855AA	PNUSAS003799	SRR4295155	2016	1		North America	United States	Enteritidis	Enteritidis	1425	50569
SAL_GA9940AA	PNUSAS003782	SRR4295068	2016	6		North America	United States	Typhimurium	Enteritidis	1425	50566
SAL_GA9950AA	PNUSAS003876	SRR4295058				North America	United States	Enteritidis (Predicted)	Enteritidis	1425	49709
SAL_GA9951AA	PNUSAS003875	SRR4295057				North America	United States	Enteritidis (Predicted)	Enteritidis	1425	49709
SAL_GA9958AA	PNUSAS004206	SRR4295050	2016	8		North America	United States	Enteritidis	Enteritidis	1425	50610
SAL_GA9959AA	PNUSAS003924	SRR4295049	2016	8		North America	United States	Enteritidis	Enteritidis	1425	50504
SAL_GA9962AA	PNUSAS004073	SRR4295046	2016	8		North America	United States	Enteritidis	Enteritidis	1425	50525
SAL_GA9963AA	PNUSAS003979	SRR4295045	2016	8		North America	United States	Enteritidis (Predicted)	Enteritidis	1425	49135
SAL_GA9964AA	PNUSAS004086	SRR4295044	2016	8		North America	United States	Enteritidis	Enteritidis	1425	50526
SAL_GA9968AA	PNUSAS004069	SRR4295040	2016	8		North America	United States	Enteritidis	Enteritidis	1425	50620
SAL_GA9969AA	PNUSAS004068	SRR4295039	2016	8		North America	United States	Enteritidis	Enteritidis	1425	50506
SAL_GA9976AA	PNUSAS003976	SRR4295032	2016	8		North America	United States	Enteritidis	Enteritidis	1425	50507
SAL_GA9983AA	PNUSAS003847	SRR4295025				North America	United States	Enteritidis (Predicted)	Enteritidis	1425	44222
SAL_HA0034AA	16042880-SAL	traces-0PagAiU				Europe	Luxembourg	Enteritidis	Enteritidis	3888	50501
SAL_HA0035AA	S/20160783	traces-0YghClG	2016	7	27			Enteritidis	Enteritidis	3888	3621
SAL_HA0036AA	S/20160795	traces-0LGKrlS	2016	7	27			Enteritidis	Enteritidis	3888	3621
SAL_HA0037AA	S/20160873	traces-0ukqJOA	2016	8	10			Enteritidis	Enteritidis	3888	50543
SAL_HA0038AA	S/20160876	traces-0HObPbc	2016	8	11			Enteritidis	Enteritidis	1425	50518
SAL_HA0039AA	S/20160879	traces-0rxPfhJ	2016	8	11			Enteritidis	Enteritidis	3888	50542
SAL_HA0040AA	S/20160904	traces-0HeqRtp	2016	8	13			Enteritidis	Enteritidis	3888	2002
SAL_HA0041AA	S/20160912	traces-0wJLNVX	2016	8	19			Enteritidis	Enteritidis	3888	50541
SAL_HA0042AA	S/20160968	traces-0IxqceW	2016	8	25			Enteritidis	Enteritidis	3888	50540
SAL_HA0043AA	S/20161024	traces-0xKSsTa	2016	9	2			Enteritidis	Enteritidis	3888	49818
SAL_HA0044AA	S/20161028	traces-0fdDEZX	2016	9	3			Enteritidis	Enteritidis	1425	50539
SAL_HA0045AA	S/20161055	traces-0AsfeHr	2016	9	7			Enteritidis	Enteritidis	3888	50538
SAL_HA0046AA	S/20160905	traces-0SRQfAf	2016	8	13			Enteritidis	Enteritidis	3888	2002
SAL_HA0062AA	PNUSAS004201	SRR4297073				North America	United States	Enteritidis (Predicted)	Enteritidis	1425	50669
SAL_HA0063AA	PNUSAS004200	SRR4297072	2016	4		North America	United States	Enteritidis	Enteritidis	34197	45550
SAL_HA0087AA	PNUSAS003774	SRR4297048	2016	8		North America	United States	Enteritidis	Enteritidis	1425	50662
SAL_HA0091AA	PNUSAS003770	SRR4297044	2016	8		North America	United States	Enteritidis	Enteritidis	1425	17527
SAL_HA0092AA	PNUSAS003923	SRR4297043	2016	7		North America	United States	Serotype Pending	Enteritidis	1425	47838
SAL_HA0116AA	IA-2009121617	SRR4301117	2009	8		North America	United States	Enteritidis	Enteritidis	1425	50678
SAL_HA0121AA	IA-2009099254	SRR4301112	2009	8		North America	United States	Enteritidis (Predicted)	Enteritidis	1425	50684
SAL_HA0169AA	I2016014963-1	SRR4340444	2016	9		North America	United States	Enteritidis	Enteritidis	1425	50731
SAL_HA0171AA	1091600823	traces-0KxIpSF				Europe	Netherlands	Enteritidis	Enteritidis	3888	2002
SAL_HA0172AA	1091600857	traces-0mSUaNJ				Europe	Netherlands	Enteritidis	Enteritidis	3888	2002
SAL_HA0174AA	1091600881	traces-0EerzbO				Europe	Netherlands	Enteritidis	Enteritidis	3888	49337
SAL_HA0175AA	1091600936	traces-0iZIIUR				Europe	Netherlands	Enteritidis	Enteritidis	3888	2002
SAL_HA0176AA	1091600937	traces-0xyzobu				Europe	Netherlands	Enteritidis	Enteritidis	3888	2002
SAL_HA0177AA	1091600938	traces-0aEIVyk				Europe	Netherlands	Enteritidis	Enteritidis	3888	49337
SAL_HA0178AA	1091600969	traces-0hreilX				Europe	Netherlands	Enteritidis	Enteritidis	3888	50714
SAL_HA0179AA	1091601041	traces-0wASplU				Europe	Netherlands	Enteritidis	Enteritidis	3888	50713
SAL_HA0180AA	1091601047	traces-0AyKmCj				Europe	Netherlands	Enteritidis	Enteritidis	3888	50712
SAL_HA0181AA	1091601061	traces-0zOeecB				Europe	Netherlands	Enteritidis	Enteritidis	3888	2002
SAL_HA0182AA	1091601070	traces-0GzAfvM				Europe	Netherlands	Enteritidis	Enteritidis	3888	2002

SAL_HA0183AA	1091601074	traces-0Elgqhy			Europe	Netherlands	Enteritidis	Enteritidis	3888	2002
SAL_HA0184AA	1091601093	traces-0fGcFPJ			Europe	Netherlands	Enteritidis	Enteritidis	3888	50711
SAL_HA0185AA	1091601102	traces-0dDyXdO			Europe	Netherlands	Enteritidis	Enteritidis	3888	50725
SAL_HA0186AA	1091601107	traces-0UyEQQj			Europe	Netherlands	Enteritidis	Enteritidis	3888	50724
SAL_HA0187AA	1091601124	traces-0aYTQXL			Europe	Netherlands	Enteritidis	Enteritidis	3888	50711
SAL_HA0188AA	1091601127	traces-0YFqGwj			Europe	Netherlands	Enteritidis	Enteritidis	3888	2002
SAL_HA0189AA	1091601157	traces-0fZhOFg			Europe	Netherlands	Enteritidis	Enteritidis	3888	50710
SAL_HA0190AA	1091601159	traces-0oJtqbN			Europe	Netherlands	Enteritidis	Enteritidis	3888	2002
SAL_HA0191AA	1091601198	traces-0tZLgke			Europe	Netherlands	Enteritidis	Enteritidis	3888	2002
SAL_HA0192AA	1091601201	traces-0PVKlRd			Europe	Netherlands	Enteritidis	Enteritidis	3888	2002
SAL_HA0193AA	1091601228	traces-0SdBjch			Europe	Netherlands	Enteritidis	Enteritidis	3888	2002
SAL_HA0194AA	1091601240	traces-0FgiALC			Europe	Netherlands	Enteritidis	Enteritidis	26384	50708
SAL_HA0195AA	1091601241	traces-0nCTWde			Europe	Netherlands	Enteritidis	Enteritidis	3888	50723
SAL_HA0196AA	1091601246	traces-0pYcocC			Europe	Netherlands	Enteritidis	Enteritidis	3888	2002
SAL_HA0197AA	1091601247	traces-0UdDGNM			Europe	Netherlands	Enteritidis	Enteritidis	3888	50709
SAL_HA0198AA	1091601270	traces-0tcEcnm			Europe	Netherlands	Enteritidis	Enteritidis	3888	2002
SAL_HA0199AA	1091601298	traces-0NZBwvm			Europe	Netherlands	Enteritidis	Enteritidis	3888	50722
SAL_HA0200AA	1091601305	traces-0xyhOFg			Europe	Netherlands	Enteritidis	Enteritidis	3888	50721
SAL_HA0201AA	1091601341	traces-0gyUMnx			Europe	Netherlands	Enteritidis	Enteritidis	3888	50720
SAL_HA0202AA	1091601351	traces-0aKBGmi			Europe	Netherlands	Enteritidis	Enteritidis	3888	50720
SAL_HA0203AA	1091601352	traces-0cgqzrc			Europe	Netherlands	Enteritidis	Enteritidis	3888	2002
SAL_HA0204AA	1091601383	traces-0CtjiWp			Europe	Netherlands	Enteritidis	Enteritidis	26384	50708
SAL_HA0205AA	1091601384	traces-0LLSAFV			Europe	Netherlands	Enteritidis	Enteritidis	3888	2002
SAL_HA0206AA	1091601395	traces-0KBgjzH			Europe	Netherlands	Enteritidis	Enteritidis	3888	2002
SAL_HA0207AA	16EP001503	traces-0iFZwUQ			Europe	Norway	Enteritidis	Enteritidis	3888	50719
SAL_HA0208AA	2015-01-4688-B3075	traces-0dBJwtj			Europe	Norway	Enteritidis	Enteritidis	3888	50718
SAL_HA0209AA	1091601416	traces-0nWYLbU			Europe	Netherlands	Enteritidis	Enteritidis	3888	2002
SAL_HA0210AA	1091601436	traces-0VJyrzH			Europe	Netherlands	Enteritidis	Enteritidis	3888	50729
SAL_HA0211AA	1091601443	traces-0EeLaxz			Europe	Netherlands	Enteritidis	Enteritidis	3888	50717
SAL_HA0212AA	1091601460	traces-0aBVvFh			Europe	Netherlands	Enteritidis	Enteritidis	3888	50707
SAL_HA0213AA	1091601461	traces-0RZrzho			Europe	Netherlands	Enteritidis	Enteritidis	3888	50707
SAL_HA0214AA	1091601466	traces-0zsZjbH			Europe	Netherlands	Enteritidis	Enteritidis	3888	50716
SAL_HA0215AA	1091601491	traces-0ynQecZ			Europe	Netherlands	Enteritidis	Enteritidis	3888	2002
SAL_HA0216AA	1091601505	traces-0pEnjNd			Europe	Netherlands	Enteritidis	Enteritidis	3888	50715
SAL_HA0217AA	1091601538	traces-0kqifgv			Europe	Netherlands	Enteritidis	Enteritidis	3888	2002
SAL_HA0218AA	1091601549	traces-0VEViXj			Europe	Netherlands	Enteritidis	Enteritidis	3888	2002
SAL_HA0219AA	1091601553	traces-0NrAXVB			Europe	Netherlands	Enteritidis	Enteritidis	3888	2002
SAL_HA0220AA	1091601557	traces-0FcjjuE			Europe	Netherlands	Enteritidis	Enteritidis	3888	50728
SAL_HA0221AA	1091601558	traces-0JvFtsR			Europe	Netherlands	Enteritidis	Enteritidis	3888	50727
SAL_HA0222AA	1091601559	traces-0SjXMmd			Europe	Netherlands	Enteritidis	Enteritidis	3888	2002
SAL_HA0223AA	1091601484	traces-0qSJTVO			Europe	Netherlands	Enteritidis	Enteritidis	3888	50726
SAL_HA0224AA	110368	traces-0hAireI			Europe	Finland	Enteritidis	Enteritidis	3888	50730
SAL_HA0255AA	PNUSAS004346	SRR4342982	2016	9	North America	United States	Enteritidis	Enteritidis	27446	50782
SAL_HA0256AA	PNUSAS004288	SRR4342981			North America	United States	Enteritidis (Predicted)	Enteritidis	1425	50794
SAL_HA0257AA	PNUSAS004129	SRR4342980	2016	8	North America	United States	Enteritidis	Enteritidis	1425	50778
SAL_HA0258AA	PNUSAS004128	SRR4342979	2016	9	North America	United States	Enteritidis	Enteritidis	27863	50763
SAL_HA0259AA	PNUSAS004127	SRR4342978	2016	8	North America	United States	Enteritidis	Enteritidis	27863	50795
SAL_HA0260AA	PNUSAS004126	SRR4342977	2016	8	North America	United States	Enteritidis	Enteritidis	1425	50832
SAL_HA0266AA	PNUSAS004082	SRR4342971	2016	8	North America	United States	Enteritidis	Enteritidis	1425	50765
SAL_HA0272AA	PNUSAS003894	SRR4342965	2016	8	North America	United States	Enteritidis	Enteritidis	1425	50789
SAL_HA0275AA	PNUSAS003893	SRR4342962	2016	7	North America	United States	Enteritidis	Enteritidis	1425	50808
SAL_HA0282AA	PNUSAS004185	SRR4342955	2016	9	North America	United States	Enteritidis	Enteritidis	1425	50801
SAL_HA0283AA	PNUSAS004184	SRR4342954	2016	9	North America	United States	Enteritidis	Enteritidis	1425	50770
SAL_HA0285AA	PNUSAS004183	SRR4342952			North America	United States	Enteritidis (Predicted)	Enteritidis	1425	50764
SAL_HA0287AA	PNUSAS004186	SRR4342950	2016	8	North America	United States	Enteritidis	Enteritidis	1425	50802
SAL_HA0288AA	PNUSAS004182	SRR4342949	2016	8	North America	United States	Enteritidis	Enteritidis	1425	50797
SAL_HA0289AA	PNUSAS004181	SRR4342948	2016	8	North America	United States	Enteritidis	Enteritidis	1425	50771
SAL_HA0291AA	PNUSAS004180	SRR4342946	2016	8	North America	United States	Enteritidis	Enteritidis	1425	50772
SAL_HA0293AA	PNUSAS004179	SRR4342944	2016	8	North America	United States	Enteritidis	Enteritidis	27944	46038

SAL_HA0294AA	PNUSAS004178	SRR4342943	2016	8	North America	United States	Enteritidis	Enteritidis	1425	50773
SAL_HA0295AA	PNUSAS004177	SRR4342942	2016	8	North America	United States	Enteritidis	Enteritidis	1425	48715
SAL_HA0302AA	PNUSAS003721	SRR4342935	2016	8	North America	United States	Bareilly	Enteritidis	1425	50789
SAL_HA0306AA	PNUSAS003717	SRR4342931	2016	8	North America	United States	Enteritidis	Enteritidis	1425	50822
SAL_HA0307AA	PNUSAS003716	SRR4342930	2016	8	North America	United States	Typhimurium	Enteritidis	1425	50747
SAL_HA0308AA	PNUSAS003715	SRR4342929			North America	United States	Enteritidis (Predicted)	Enteritidis	1425	50747
SAL_HA0319AA	PNUSAS003457	SRR4342918			North America	United States	Enteritidis (Predicted)	Enteritidis	1425	50750
SAL_HA0321AA	PNUSAS003455	SRR4342916	2016	7	North America	United States	Enteritidis	Enteritidis	1425	50752
SAL_HA0327AA	PNUSAS004334	SRR4342910	2016	8	North America	United States	Enteritidis	Enteritidis	1425	50757
SAL_HA0328AA	PNUSAS004333	SRR4342909	2016	8	North America	United States	Enteritidis	Enteritidis	1425	50813
SAL_HA0335AA	PNUSAS004325	SRR4342902	2016	8	North America	United States	Enteritidis	Enteritidis	1425	50738
SAL_HA0401AA	2016K-0846	SRR4298894			North America	United States	Enteritidis (Predicted)	Enteritidis	1425	50789
SAL_HA0402AA	2016K-0834	SRR4298893			North America	United States	Enteritidis (Predicted)	Enteritidis	1425	49727
SAL_HA0403AA	2016K-0840	SRR4298892			North America	United States	Enteritidis (Predicted)	Enteritidis	1425	50789
SAL_HA0404AA	2016K-0839	SRR4298891			North America	United States	Enteritidis (Predicted)	Enteritidis	1425	50860
SAL_HA0405AA	2016K-0835	SRR4298890			North America	United States	Enteritidis (Predicted)	Enteritidis	1425	50865
SAL_HA0407AA	2016K-0857	SRR4298888			North America	United States	Enteritidis (Predicted)	Enteritidis	1425	50851
SAL_HA0410AA	2016K-0856	SRR4298885			North America	United States	Enteritidis (Predicted)	Enteritidis	1425	50862
SAL_HA0416AA	CFSAN025424	SRR4319131	2006		Europe	United Kingdom	Enteritidis	Enteritidis	1425	50856
SAL_HA0430AA	PNUSAS003800	SRR4415956			North America	United States	Enteritidis (Predicted)	Enteritidis	1425	45084
SAL_HA0433AA	PNUSAS004039	SRR4415952	2016	8	North America	United States	Enteritidis	Enteritidis	1425	50897
SAL_HA0451AA	PNUSAS004015	SRR4415734			North America	United States	Enteritidis (Predicted)	Enteritidis	1425	49709
SAL_HA0452AA	PNUSAS004014	SRR4415733			North America	United States	Enteritidis	Enteritidis	1425	50906
SAL_HA0453AA	PNUSAS004013	SRR4414192			North America	United States	Enteritidis	Enteritidis	1425	50907
SAL_HA0472AA	PNUSAS004305	SRR4414128			North America	United States	Enteritidis (Predicted)	Enteritidis	1425	50893
SAL_HA0473AA	PNUSAS004304	SRR4414127			North America	United States	Enteritidis (Predicted)	Enteritidis	1425	50224
SAL_HA0474AA	PNUSAS004303	SRR4414126	2016	8	North America	United States	Enteritidis	Enteritidis	1425	50893
SAL_HA0475AA	PNUSAS004302	SRR4414125			North America	United States	Enteritidis (Predicted)	Enteritidis	1425	50893
SAL_HA0487AA	PNUSAS004231	SRR4414113	2016	8	North America	United States	Enteritidis	Enteritidis	1425	51277
SAL_HA0511AA	PNUSAS004385	SRR4414089	2016	8	North America	United States	Enteritidis	Enteritidis	1425	50747
SAL_HA0520AA	PNUSAS004376	SRR4414080			North America	United States	Enteritidis (Predicted)	Enteritidis	1425	51260
SAL_HA0521AA	PNUSAS004375	SRR4414079	2016	8	North America	United States	Enteritidis	Enteritidis	1425	51224
SAL_HA0522AA	PNUSAS004374	SRR4414078			North America	United States	Enteritidis (Predicted)	Enteritidis	1425	51272
SAL_HA0527AA	PNUSAS004368	SRR4414073	2016	8	North America	United States	Enteritidis	Enteritidis	1425	51263
SAL_HA0528AA	PNUSAS004367	SRR4414072	2016	8	North America	United States	Enteritidis	Enteritidis	1425	51273
SAL_HA0531AA	S16BD04187	traces-0LqRGYH			Europe	Belgium	Enteritidis	Enteritidis	3888	50912
SAL_HA0532AA	S16BD04298	traces-0vjDelh			Europe	Belgium	Enteritidis	Enteritidis	3888	2002
SAL_HA0535AA	S16BD04564	traces-0IKCFgU			Europe	Belgium	Enteritidis	Enteritidis	3941	50914
SAL_HA0536AA	S16BD04629	traces-0bmCBkz			Europe	Belgium	Enteritidis	Enteritidis	3941	50915
SAL_HA0537AA	S16BD05065	traces-0VCYamL			Europe	Belgium	Enteritidis	Enteritidis	3888	2002
SAL_HA0538AA	S16BD05414	traces-0zpogLn			Europe	Belgium	Enteritidis	Enteritidis	3888	2002
SAL_HA0539AA	S16BD05419	traces-0YgxwPp			Europe	Belgium	Enteritidis	Enteritidis	3888	2002
SAL_HA0540AA	S16BD05528	traces-0noeRUu			Europe	Belgium	Enteritidis	Enteritidis	3888	2002
SAL_HA0541AA	S16BD05443	traces-0dvQhTX			Europe	Belgium	Enteritidis	Enteritidis	3941	50916
SAL_HA0542AA	S343	traces-0KFPqMO			Europe	Sweden	Enteritidis	Enteritidis	3941	49815
SAL_HA0543AA	S365	traces-0YjbLIH			Europe	Sweden	Enteritidis	Enteritidis	3888	49337
SAL_HA0544AA	S398	traces-0nriHyD			Europe	Sweden	Enteritidis	Enteritidis	3888	50917
SAL_HA0545AA	S424	traces-0xJVRCg			Europe	Sweden	Enteritidis	Enteritidis	3888	50917
SAL_HA0546AA	S431	traces-0YtrCcE			Europe	Sweden	Enteritidis	Enteritidis	3888	2002
SAL_HA0547AA	S453	traces-0oiGvcb			Europe	Sweden	Enteritidis	Enteritidis	3941	50918
SAL_HA0548AA	2015-01-1680	traces-0rnFNxB			Europe	Norway	Enteritidis	Enteritidis	3888	50919
SAL_HA0549AA	16EP001346	traces-0jOCaLg			Europe	Norway	Enteritidis	Enteritidis	3888	50920
SAL_HA0550AA	16EP001384	traces-0LiTwfJ			Europe	Norway	Enteritidis	Enteritidis	3888	50921
SAL_HA0551AA	16EP001290	traces-0RAQIAN			Europe	Norway	Enteritidis	Enteritidis	3888	50538
SAL_HA0552AA	16EP001157	traces-0yXdkXm			Europe	Norway	Enteritidis	Enteritidis	3941	49815
SAL_HA0553AA	16EP001080	traces-0leTlht			Europe	Norway	Enteritidis	Enteritidis	3941	50922
SAL_HA0554AA	16EP001022	traces-0StIFta			Europe	Norway	Enteritidis	Enteritidis	3941	49815
SAL_HA0555AA	16EP001008	traces-0ZvNvwN			Europe	Norway	Enteritidis	Enteritidis	3888	50923
SAL_HA0556AA	serovar enteritidis	SRR4421135	2015	2	North America	Canada	Enteritidis	Enteritidis	1425	51225

SAL_HA0557AA	serovar enteritidis	SRR4421134	2015	2	North America	Canada	Enteritidis	Enteritidis	1425	51235
SAL_HA0558AA	serovar enteritidis	SRR4421133	2015	2	North America	Canada	Enteritidis	Enteritidis	1425	51130
SAL_HA0559AA	serovar enteritidis	SRR4421132	2015	2	North America	Canada	Enteritidis	Enteritidis	1425	51226
SAL_HA0560AA	serovar enteritidis	SRR4421131	2015	2	North America	Canada	Enteritidis	Enteritidis	1425	51244
SAL_HA0561AA	serovar enteritidis	SRR4421130	2015	2	North America	Canada	Enteritidis	Enteritidis	1425	51227
SAL_HA0562AA	serovar enteritidis	SRR4421129	2015	2	North America	Canada	Enteritidis	Enteritidis	1425	51235
SAL_HA0563AA	serovar enteritidis	SRR4421128	2015	2	North America	Canada	Enteritidis	Enteritidis	1425	51201
SAL_HA0564AA	serovar enteritidis	SRR4421127	2015	3	North America	Canada	Enteritidis	Enteritidis	1425	23956
SAL_HA0565AA	serovar enteritidis	SRR4421126	2015	2	North America	Canada	Enteritidis	Enteritidis	1425	51236
SAL_HA0571AA	PNUSAS004453	SRR4421095			North America	United States	Enteritidis (Predicted)	Enteritidis	1425	51185
SAL_HA0604AA	2014AM-0194	SRR4420944			North America	United States	Enteritidis (Predicted)	Enteritidis	1425	48902
SAL_HA0607AA	2014AM-2800	SRR4420941			North America	United States	Enteritidis (Predicted)	Enteritidis	1425	51219
SAL_HA0631AA	2014AM-2057	SRR4420916			North America	United States	Enteritidis (Predicted)	Enteritidis	1425	51192
SAL_HA0635AA	2014AM-2534	SRR4420911			North America	United States	Enteritidis (Predicted)	Enteritidis	1425	51161
SAL_HA0637AA	2014AM-0699	SRR4420909			North America	United States	Enteritidis (Predicted)	Enteritidis	1425	51135
SAL_HA0642AA	2014AM-1411	SRR4420904			North America	United States	Enteritidis (Predicted)	Enteritidis	26543	51208
SAL_HA0644AA	2014AM-1408	SRR4420902			North America	United States	Enteritidis (Predicted)	Enteritidis	26543	51163
SAL_HA0650AA	2014AM-1407	SRR4420896			North America	United States	Enteritidis (Predicted)	Enteritidis	1425	51127
SAL_HA0651AA	2014AM-1410	SRR4420895			North America	United States	Enteritidis (Predicted)	Enteritidis	1425	51200
SAL_HA0652AA	2014AM-1409	SRR4420861			North America	United States	Enteritidis (Predicted)	Enteritidis	1425	51199
SAL_HA0654AA	2014AM-0786	SRR4420859			North America	United States	Enteritidis (Predicted)	Enteritidis	1425	51141
SAL_HA0665AA	2014AM-0529	SRR4420838			North America	United States	Enteritidis (Predicted)	Enteritidis	1425	51149
SAL_HA0671AA	2014AM-0353	SRR4420824			North America	United States	Enteritidis (Predicted)	Enteritidis	1425	51182
SAL_HA0696AA	serovar enteritidis	SRR4420670	2015	2	North America	Canada	Enteritidis	Enteritidis	1425	51152
SAL_HA0697AA	serovar enteritidis	SRR4420669	2015	2	North America	Canada	Enteritidis	Enteritidis	1425	51130
SAL_HA0698AA	serovar enteritidis	SRR4420668	2015	2	North America	Canada	Enteritidis	Enteritidis	1425	51201
SAL_HA0699AA	serovar enteritidis	SRR4420667	2015	1	North America	Canada	Enteritidis	Enteritidis	1425	51125
SAL_HA0700AA	serovar enteritidis	SRR4420666	2015	1	North America	Canada	Enteritidis	Enteritidis	1425	51153
SAL_HA0701AA	serovar enteritidis	SRR4420665	2015	2	North America	Canada	Enteritidis	Enteritidis	1425	51130
SAL_HA0702AA	serovar enteritidis	SRR4420664	2015	2	North America	Canada	Enteritidis	Enteritidis	1425	23956
SAL_HA0703AA	serovar enteritidis	SRR4420663	2015	2	North America	Canada	Enteritidis	Enteritidis	1425	51168
SAL_HA0704AA	serovar enteritidis	SRR4420662	2015	1	North America	Canada	Enteritidis	Enteritidis	1425	51130
SAL_HA0705AA	serovar enteritidis	SRR4420661	2015	1	North America	Canada	Enteritidis	Enteritidis	1425	51131
SAL_HA0726AA	PNUSAS004491	SRR4418399			North America	United States	Enteritidis (Predicted)	Enteritidis	1425	48035
SAL_HA0729AA	PNUSAS004411	SRR4418396			North America	United States	Enteritidis (Predicted)	Enteritidis	1425	51134
SAL_HA0738AA	PNUSAS004410	SRR4418386			North America	United States	Enteritidis (Predicted)	Enteritidis	1425	50789
SAL_HA0739AA	PNUSAS004409	SRR4418385			North America	United States	Enteritidis (Predicted)	Enteritidis	1425	51083
SAL_HA0740AA	PNUSAS004542	SRR4418384			North America	United States	Enteritidis (Predicted)	Enteritidis	1425	51082
SAL_HA0741AA	PNUSAS004541	SRR4418383			North America	United States	Enteritidis (Predicted)	Enteritidis	1425	51081
SAL_HA0752AA	PNUSAS004442	SRR4418372			North America	United States	Enteritidis (Predicted)	Enteritidis	1425	51091
SAL_HA0764AA	2016K-0889	SRR4418360			North America	United States	Enteritidis (Predicted)	Enteritidis	1425	50789
SAL_HA0765AA	2016K-0888	SRR4418359			North America	United States	Enteritidis (Predicted)	Enteritidis	1425	50789
SAL_HA0766AA	2016K-0890	SRR4418358			North America	United States	Enteritidis (Predicted)	Enteritidis	1425	51081
SAL_HA0777AA	PNUSAS004405	SRR4418347			North America	United States	Enteritidis (Predicted)	Enteritidis	1425	51093
SAL_HA0841AA	FDA00008291	SRR4418787	2014	8	North America	United States	Enteritidis	Enteritidis	46583	51025
SAL_HA0862AA	PNUSAS004125	SRR4416110	2016	8	North America	United States	Enteritidis	Enteritidis	27863	50987
SAL_HA0863AA	PNUSAS004124	SRR4416109	2016	8	North America	United States	Enteritidis	Enteritidis	1425	51005
SAL_HA0865AA	PNUSAS004122	SRR4416107	2016	8	North America	United States	Enteritidis	Enteritidis	1425	50986
SAL_HA0866AA	PNUSAS004121	SRR4416106			North America	United States	Enteritidis (Predicted)	Enteritidis	1425	51007
SAL_HA0868AA	PNUSAS004119	SRR4416104			North America	United States	Enteritidis (Predicted)	Enteritidis	1425	50990
SAL_HA0873AA	PNUSAS004300	SRR4416099			North America	United States	Enteritidis (Predicted)	Enteritidis	1425	49135
SAL_HA0874AA	PNUSAS004299	SRR4416098			North America	United States	Enteritidis (Predicted)	Enteritidis	1425	50994
SAL_HA0884AA	PNUSAS004261	SRR4416023			North America	United States	Enteritidis	Enteritidis	1425	50968
SAL_HA0891AA	PNUSAS004254	SRR4416008	2016	8	North America	United States	Javiana	Enteritidis	1425	50970
SAL_HA0906AA	PNUSAS004292	SRR4415993			North America	United States	Enteritidis (Predicted)	Enteritidis	1425	1171
SAL_HA0926AA	PNUSAS003817	SRR4415973			North America	United States	Enteritidis (Predicted)	Enteritidis	1425	50957
SAL_HA0937AA	PNUSAS003805	SRR4415962			North America	United States	Enteritidis (Predicted)	Enteritidis	1425	50930
